# Systems pathology analysis identifies neurodegenerative nature of age‐related vitreoretinal interface diseases

**DOI:** 10.1111/acel.12809

**Published:** 2018-07-02

**Authors:** Tiina Öhman, Fitsum Tamene, Helka Göös, Sirpa Loukovaara, Markku Varjosalo

**Affiliations:** ^1^ Institute of Biotechnology and Helsinki Institute of Life Science University of Helsinki Helsinki Finland; ^2^ Unit of Vitreoretinal Surgery, Department of Ophthalmology University of Helsinki and Helsinki University Hospital Helsinki Finland

**Keywords:** aging, epiretinal membrane, macular hole, MS‐based quantitative proteomics, neurodegeneration, vitreous humor

## Abstract

Aging is a phenomenon that is associated with profound medical implications. Idiopathic epiretinal membrane (iEMR) and macular hole (MH) are the major vision‐threatening vitreoretinal diseases affecting millions of aging people globally, making these conditions an important public health issue. iERM is characterized by fibrous tissue developing on the surface of the macula, which leads to biomechanical and biochemical macular damage. MH is a small breakage in the macula and is associated with many ocular conditions. Although several individual factors and pathways are suggested, a systems pathology level understanding of the molecular mechanisms underlying these disorders is lacking. Therefore, we performed mass spectrometry‐based label‐free quantitative proteomics analysis of the vitreous proteomes from patients with iERM and MH to identify the key proteins, as well as the multiple interconnected biochemical pathways, contributing to the development of these diseases. We identified a total of 1,014 unique proteins, many of which are linked to inflammation and the complement cascade, revealing the inflammation processes in retinal diseases. Additionally, we detected a profound difference in the proteomes of iEMR and MH compared to those of diabetic retinopathy with macular edema and rhegmatogenous retinal detachment. A large number of neuronal proteins were present at higher levels in the iERM and MH vitreous, including neuronal adhesion molecules, nervous system development proteins, and signaling molecules, pointing toward the important role of neurodegenerative component in the pathogenesis of age‐related vitreoretinal diseases. Despite them having marked similarities, several unique vitreous proteins were identified in both iERM and MH, from which candidate targets for new diagnostic and therapeutic approaches can be provided.

## 1 INTRODUCTION

Population aging is a global phenomenon with profound medical implications. Tissue dysfunction associated with aging affects all vital organs, including the eyes. Various ocular structures are affected by aging, such as the macula, the functional center of the retina responsible for precise central vision. Idiopathic epiretinal membrane (iEMR) and macular hole (MH) are the major vision‐threatening vitreoretinal interface diseases that affect millions of aging people globally, making these conditions an important public health issue (Steel & Lotery, 2013). iERM is characterized by the growth of fibrocellular tissue along the inner surface of the retina (Snead, James, & Snead, 2008). Ultimately, an impaired repair and an excess of fibrosis in iERM eyes lead to biomechanical and biochemical macular damage, the development of retinal surface wrinkling with or without shallow tractional retinal detachment (RD), macular vascular distortion, the breakdown of the blood–retinal barrier at the retinal pigment epithelial (RPE) level, and vascular leakage. MH, however, is a full‐thickness defect of retinal tissue involving the anatomic fovea (Ho, Guyer, & Fine, 1998). Originally, MH was described in the trauma setting, but it has been associated with many ocular conditions, and the greater majority of MH cases are idiopathic (Chung & Byeon, 2017).

The exact pathogenic mechanisms underlying these two pathological conditions are still not known. An older age and the development of anomalous posterior vitreous detachment (PVD) are the generally accepted nonmodifiable key risk factors in the iERM pathogenesis (Cheung et al., 2017). In addition, a number of inflammatory and immunomodulatory processes, chronic oxidative insult, proteolysis, and cytoskeleton remodeling, have been implicated in its formation (Joshi, Agrawal, & Christoforidis, 2013; Pollreisz et al., 2013). Metabolically, the retina is the most oxygen‐consuming tissue in the human body (Arden, Sidman, Arap, & Schlingemann, 2005). Therefore, metabolic stress and an altered microvascular retinal blood flow, together with genetic and lifestyle‐related factors, such as smoking, could also play a role in iERM formation (Salminen et al., 2011). The pathogenesis of idiopathic age‐related MH remains unclear despite the existence of a list of theories, making a systems level understanding of the disease instrumental in developing therapeutic approaches. Currently, pars plana vitrectomy remains the primary treatment option for achieving MH closure and improvement and/or stabilization of visual acuity in iERM eyes.

The fundamental cell types involved in iERM are RPE, Müller cells, astrocytes, and microglia that begin proliferating and migrating onto the surface of the retina (Schumann et al., 2011; Zhao et al., 2013). Microglia, the main retinal immune cells (macrophages) especially play a key role both in degenerative and inflammatory retinal diseases (Fritsche et al., 2014; Karlstetter, Scholz, Rutar, Wong, & Provis, 2015). Additionally, other cell types present at the vitreoretinal interface, such as hyalocytes, may contribute to the ERM contraction (Kohno et al., 2009).

The protein composition of the vitreous humor is vital for its homeostasis. In healthy eyes, the homeostasis of the retinal extracellular matrix (ECM) is tightly regulated. However, it is altered in ocular disorders, and this offers a means of indirectly studying the events that take place at the retina (Miller, Budoff, Prenner, & Schwarzbauer, 2017; Monteiro et al., 2015). Mass spectrometry (MS)‐based quantitative proteomics provides a means for the determination of global proteome changes at the tissue and cellular levels, enabling a molecular level characterization of the pathophysiologies of complex eye disorders. Currently, most proteomic studies characterizing disease‐induced vitreous proteome changes have focused on proliferative and nonproliferative diabetic retinopathies (Kim et al., 2007; Loukovaara et al., 2015; Wang, Feng, Hu, Xie, & Wang, 2013), proliferative vitreoretinopathy (Garweg, Tappeneiner, & Halberstadt, 2013; Mitry, Fleck, Wright, Campbell, & Charteris, 2010), and age‐related macular degeneration (AMD; Koss et al., 2014), whereas iERM and MH remain less‐studied (Mandal et al., 2013; Pollreisz et al., 2013; Yu et al., 2014; Zhang et al., 2017).

In this study, we performed MS‐based label‐free quantitative proteomics analysis of the vitreous proteomes from patients with iERM and MH. The aim of this study was to obtain an in‐depth and global understanding of the complex and multifactorial molecular pathomechanisms underlying the two most typical age‐related vitreoretinal interface eye disorders and to show the differences in the vitreous proteome between eye‐related local degenerative conditions (MH being nonproliferative; ERM being proliferative) compared to the findings of systemic and eye‐related inflammatory diseases (diabetic patients with macular edema (DME). Understanding how to achieve primary prevention of retinal fibrosis is an important goal. Currently, there is no known way to pharmacologically impact this process, making this MS‐related investigation an important method to shed more light on the role of various proteins in this harmful process.

## 2 RESULTS AND DISCUSSION

### 2.1 Study plan and the patients’ preoperative analyses

**Figure 1:**
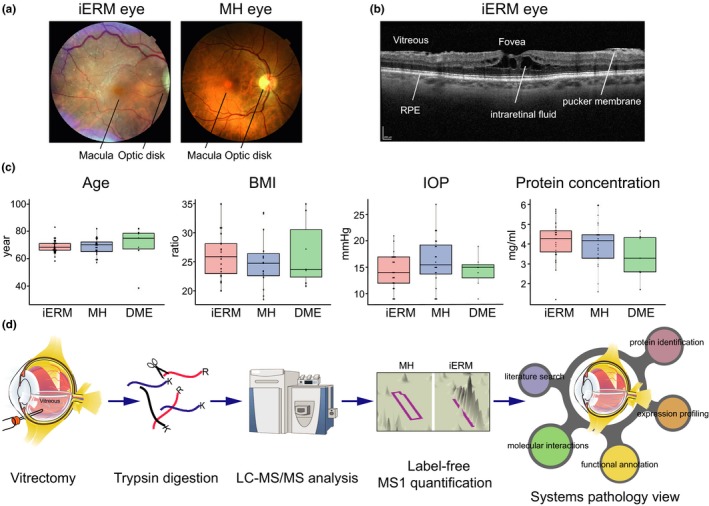
Patient characterization and quantitative proteomics pipeline. (a) Fundus photograph of the central and peripheral retina, optic disk, and macula from the patients with iERM or MH. The macula is located in the posterior pole of the eye. In the center of the macula, a shallow depression in the retina (the fovea) marks the area with the highest visual acuity. (b) An optical coherence tomography (OCT) scan through the fovea of the iERM eye reveals the abnormal organization of the retinal layers, including epiretinal fibrosis and secondary cystic macular edema. Key: RPE = retinal pigment epithelial cells; scale bar: 200 µm. (c) The demographics of the iERM, MH, and DME patients, showing the distributions of the age, body mass index (BMI), preoperative intraocular pressure (IOP), and protein concentration (mg/ml). No significance differences were found between the sample groups. (d) The experimental workflow used for identifying and quantifying the human vitreous proteins from the patients with iERM, MH or DME. Vitreous samples were collected in vitrectomy, proteins were extracted and digested with trypsin, and the resulting peptides were analyzed via LC‐MS/MS. The label‐free quantification was performed by using Progenesis LC‐MS analysis software, and the protein identification was performed using the SEQUEST search engine. Bioinformatics approaches were used to combine our proteome data with the existing knowledge in order to obtain a systems pathology view on the differences of the molecular etiologies of these eye diseases

Although both iERM and MH patients have similar visual disturbances and symptoms, including metamorphopsia, photopsia, blurred vision, and decreased visual acuity, these are different pathological conditions. iERM presents with a thin layer of scar tissue that forms on the posterior pole of the human anatomic macula, whereas MH manifests as a partial or full‐thickness loss of tissue in the central retina (Figure 1a). An optical coherence tomography (OCT) scan through the fovea of the iERM eye reveals the abnormal organization of the retinal layers including epiretinal fibrosis and secondary cystic macular edema (Figure 1b).

The transparent collagenous human vitreous is in close contact with the retina and lacks its own vasculature. Because of this close interaction, the physiological and pathological conditions of the retina are reflected directly on the protein composition of the vitreous (Monteiro et al., 2015). To obtain an in‐depth view of the vitreous humor proteome in the aging human eye, we collected and analyzed a total of 54 vitreous humor patient samples; 26 were obtained from iERM patients and 21 from MH patients. In addition, seven age‐matched type 2 diabetic retinopathy patients with macular edema (DME) were included in the analysis. Patients with prior surgical complications (such as cataract complications) were not enrolled in this study. The mean ages in the iERM, MH, and DME patient groups were highly similar, 68.7 ± 5.0 years, 68.6 ± 6.2 years, and 69.4 ± 15.0 years, respectively (Figure 1c). Neither the body mass index (BMI) proportion nor the intraocular pressure (IOP) varied significantly between the three sample groups (Figure 1c). The average protein concentration in the vitreous samples of the iERM, MH, and control‐DME was 4.2 ± 1.0 mg/ml, 4.1 ± 1.2 mg/ml, and 3.4 ± 1.1 mg/ml (mean ± *SD*), respectively (Figure 1c). Detailed patient demographics are shown in Supporting Information Table S1.

### 2.2 The analysis of the patients’ vitreous humor proteomes

**Figure 2:**
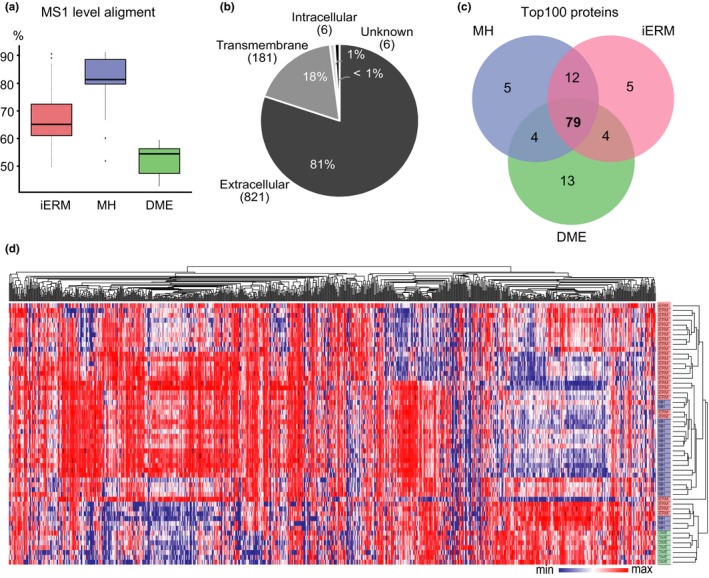
The vitreous proteomes from patients with iERM, MH, or DME show differential compositions between the diseases. (a) The MS1 spectra alignment percentages for the iERM, MH and DME sample MS analysis runs compared to those of the MH reference run. (b) Cellular localizations of the detected proteins were predicted using Phobius predictor software and were found to be predominantly either extracellular or transmembrane. (c) Venn diagram of 100 of the most abundant proteins in the iERM, MH and DME sample groups shows good overlap between the disease groups, whereas (d) the disease groups separate well after hierarchical clustering of the global quantitative proteomes. Heat‐map analysis of the hierarchical cluster analysis was performed using log2‐normalized MS1 intensities of 934 quantified vitreous proteins. The columns represent individual samples, and the rows represent the individual proteins. Each cell in the matrix represents the expression level of protein in an individual sample. Red and blue in the cells reflect the maximum and minimum expression levels, respectively

Vitreous samples from each patient were cleared from possible insoluble cellular fractions and analyzed for proteome composition. The proteins, which were digested into peptides, were analyzed by LC‐MS/MS, and the corresponding protein identity and abundance were obtained with the label‐free quantification of the MS1 (Figure 1d). The run from the MH group that had the greatest similarity to all of the other runs was automatically selected as the alignment reference. The median alignment percentage for the MH samples was 80.7%, showing high similarities between the samples (Figure 2a). The median alignment percentages for the iERM and DME sample groups compared to the MH reference run were 67.6% and 52.1%, respectively, indicating differences in the vitreous proteomes of the iERM and DME patients compared to the MH patients. On the protein level, we identified a total of 1,014 unique proteins with 4,323 nonconflicting peptides (Supporting Information Table S3). To assess the cellular localization of the identified proteins (Figure 2b, Supporting Information Table S3), we used Phobius software (Käll, Krogh, & Sonnhammer, 2004), and more than 99% of intravitreal proteins were identified either as being extracellular or as having a transmembrane domain. This corresponds well with the fact that most vitreous proteins are expected to be secreted or shed from the surrounding tissues (Joshi et al., 2013; Loukovaara et al., 2015).

Of the 1,014 identified proteins, 934 were quantified with an average MS1 intensity over 1 × 10^4^ in at least one of the sample groups (Supporting Information Table S4; MH, iERM, or DME). The most common proteins in each sample group were highly similar (Figure 2c), consisting of high abundance proteins such as serum albumin, transferrin, complement factors, and apolipoproteins. This result is highly comparable with that of previous publications about MH, iERM or DME vitreous (Loukovaara et al., 2015; Yu et al., 2014; Zhang et al., 2017). To profile the vitreous proteomes, or more precisely their possible quantitative differences, the hierarchical clustering analysis of the MS1 intensity of 934 quantified proteins was performed (Figure 2d). Clustering analysis aims to classify a mixed population into more homogenous groups based on available features. In spite of a high intergroup similarity on the level of the most abundant proteins, the clustering revealed three large separated protein clusters, which define the differential protein changes induced by the iERM, MH, and DME diseases.

To further validate the MS workflow, we performed a high‐throughput dot blot analysis for six proteins. The dot blot results correlated highly with the MS1 data (median: 0.65, Supporting Information Figure S1). The high correlation between the dot blot analysis and MS1 quantification of vitreous samples has also been previously shown (Loukovaara et al., 2015).

### 2.3 DME vitreous proteomes differ from the iERM and MH proteomes

**Figure 3:**
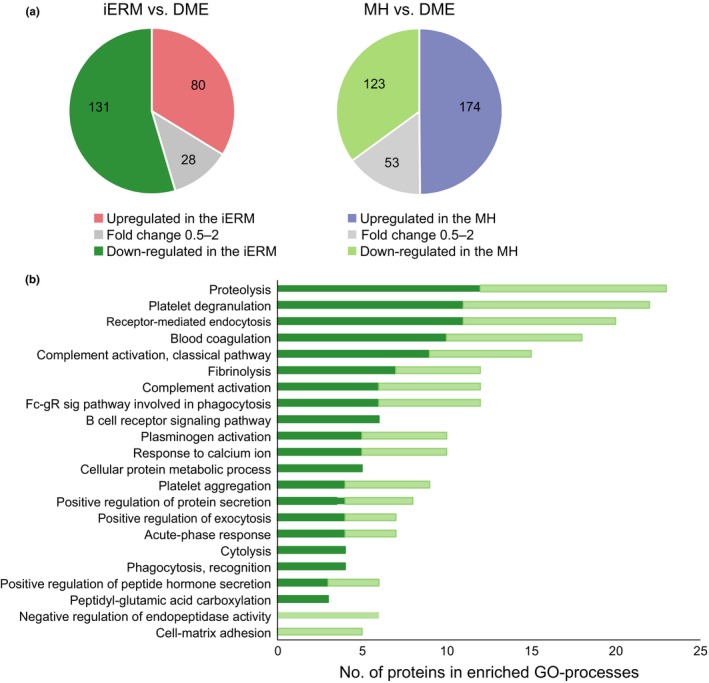
iERM and MH vitreous proteomes differ clearly from DME vitreous proteomes. (a) The abundance of 240 and 351 proteins differed statistically significantly (*q*‐value ≤0.05) between the iERM and DME groups (left panel) and between the MH and DME groups (right panel), respectively. Eighty proteins were present at higher levels and 131 proteins at lower levels in the iERM proteome (abundance ratio difference >2‐fold), and 174 proteins were present at higher levels and 123 proteins at lower levels in the MH proteome when compared to the DME proteome. (b) 131 and 123 proteins that were more abundant in the DME group were categorized according to their involvement in biological processes (Gene Ontology, Biological Processes terms) via DAVID bioinformatics resources. Dark green indicates proteins that were upregulated in DME compared to iERM (131), and light green indicates those upregulated in DME compared to MH (123)

When the iERM and MH proteomes were compared to the vitreous proteome of DME, a clear difference was detected among the groups (Figure 3a). In the iERM proteome, 80 proteins were present at a higher level and 131 proteins at a lower level compared to in the DME proteome (the abundance ratio >2, *q*‐value <0.05, Supporting Information Table S5). Likewise, in the MH proteome, 174 proteins were present at a higher level and 123 proteins at a lower level compared to in the DME proteome (Figure 3a, Supporting Information Table S6). When the differently expressed proteins were classified according to their involvement in different biological processes, we observed that the more abundant proteins in the DME group belonged to a limited number of biological processes, mainly to those of the immune system. More specifically, the proteins were associated with blood coagulation, fibrinolysis, and platelet aggregation linked to wound healing, as well as complement activation and phagocytosis linked to inflammatory processes (Figure 3b). This observation is consistent with our previous report that shows that inflammation and the complement cascade play a significant role in diabetic retinopathy and especially in its most advanced proliferative form (Loukovaara et al., 2015). Many proteins involved in the classical and alternative pathways of complement activation were also detected in the MH and iERM proteomes, indicating that inflammation may be a common denominator in both fibroproliferative and nonfibroproliferative retinal diseases.

**Figure 4:**
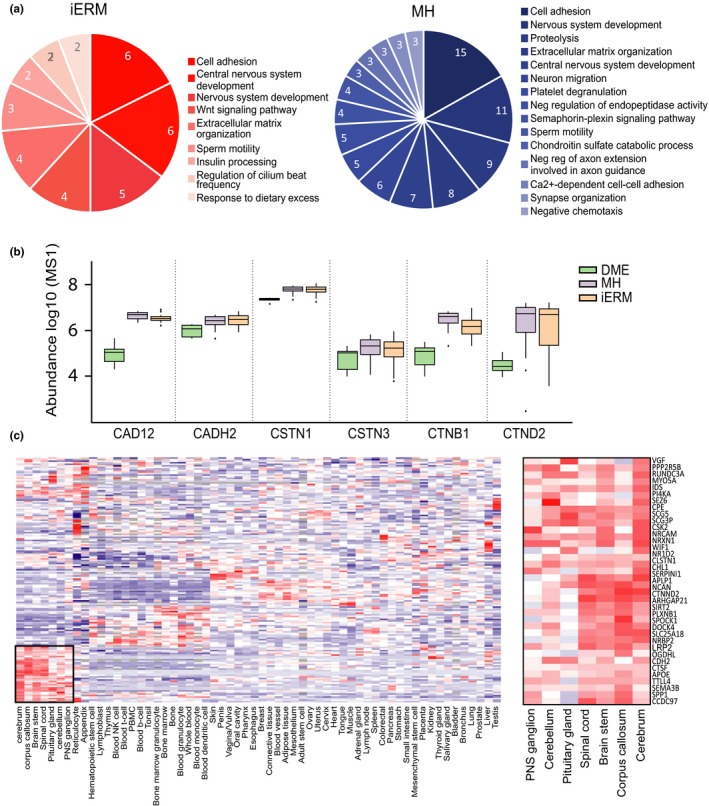
Systems level analysis highlights the interconnectivity of the upregulated proteins in iERM and MH. (a) Biological processes associated with the 80 and 174 proteins upregulated in the iERM and MH groups, respectively, compared to the DME group. (b) MS1 intensity (log‐scale) of the enriched group of adhesion molecules involved in the cadherin‐catenin complex illustrates their abundant presence in iERM and MH. (c) Hierarchical clustering of the iERM and MH sample upregulated proteins based on their gene expression profiles in healthy human tissues. The iERM and MH upregulated proteins are expressed clearly in separate clusters consisting of a majority of neuronal tissues

The proteins that were upregulated in iERM (*n* = 80) and MH (*n* = 174) showed a marked overlap (66 proteins) between the two groups. Similarly, these two retinal disease conditions shared the most frequently enriched biological processes (Figure 4a). The common proteins were associated with cell adhesion, cellular movement, nervous system development, cell signaling, and proteolysis (Table 1). This finding of multiple proteins being linked to cell adhesion, migration, and formation of the extracellular matrix supports the hypothesis that the development of macular damage requires cell migration from within the retina and extracellular matrix‐containing fibrous element of formation. It is notable to report that we detected six members of the neuronal cadherin and catenin family (CAD12, CADH2, CSTN1, CSTN3, CTNB1, CTND2) that were significantly upregulated in the iERM and MH samples (Figure 4b). The cadherin‐catenin complex is the main component of the intercellular adherens junction, which contributes to both tissue stability and dynamic cell movements (Mège & Ishiyama, 2017). Cadherin‐based adherens junctions are involved in various processes of neuronal development affecting neuronal progenitor cells including retinal stem cells. In the retina, under normal physiological circumstances, the cadherin complex contributes to RPE cell stability, whereas in some pathologic circumstances, it facilitates RPE cell motility and migration (Van Aken et al., 2003). In addition, the cadherin‐catenin complex plays a critical role in regulating Wnt signaling (Heuberger & Birchmeier, 2010), and interestingly, the Wnt signaling pathway was one of the main enriched processes detected in the iERM proteomes (Figure 4a).

**Table 1:** Sixty‐six common proteins that present with higher levels in the iERM and MH proteomes than in the DME proteome

GO processes	UniProt	Protein name	Description	Fold change compared to DME	
				MH	iERM
*Cell adhesion*	P46108	CRK	Adapter molecule crk	2.9	3.1
	P55289	CAD12	Cadherin‐12	25.7	32.2
	P19022	CADH2	Cadherin‐2	2.8	2.4
	O94985	CSTN1	Calsyntenin‐1	2.8	2.8
	Q9UQB3	CTND2	Catenin delta‐2	136.7	146.2
	P26006	ITA3	Integrin alpha‐3	33.3	11.9
	Q9UHB6	LIMA1	LIM domain and actin‐binding protein 1	9.9	2.9
	Q9P121	NTRI	Neurotrimin	2.6	2.1
	Q9HB19	PKHA2	PH domain‐containing family A member 2	10.2	14.8
	Q7Z7G0	TARSH	Target of Nesh‐SH3	4.6	6.4
*Cell motility*	Q9UFE4	CCD39	Coiled‐coil domain‐containing protein 39	3.0	2.2
	Q96DT5	DYH11	Dynein heavy chain 11, axonemal	25.4	38.0
	P58107	EPIPL	Epiplakin	23.7	26.3
	Q14533	KRT81	Keratin, type II cuticular Hb1	16.4	13.1
	P35908	K22E	Keratin, type II cytoskeletal 2 epidermal	2.3	3.1
	P20929	NEBU	Nebulin	17.1	5.9
	Q9H939	PPIP2	Pro‐Ser‐Thr phosphatase‐interacting prot 2	2.8	3.1
	Q9Y4F4	F179B	Protein FAM179B	2.3	4.4
	Q5T5U3	RHG21	Rho GTPase‐activating protein 21	6.3	6.8
	Q9HBV2	SACA1	Sperm acrosome membrane‐associated prot 1	3.4	4.3
	Q6Q759	SPG17	Sperm‐associated antigen 17	8.1	9.1
	P28290	SSFA2	Sperm‐specific antigen 2	2.5	3.4
	P32019	I5P2	Type II inositol polyphosphate‐5‐phosphatase	3.2	3.2
*Nervous system development*	Q16706	MA2A1	Alpha‐mannosidase 2	3.3	3.4
	P51693	APLP1	Amyloid‐like protein 1	3.9	3.4
	P02649	APOE	Apolipoprotein E	2.1	2.0
	P43251	BTD	Biotinidase	3.2	3.2
	Q86SQ4	GP126	G protein‐coupled receptor 126	23.3	12.3
	Q9Y287	ITM2B	Integral membrane protein 2B	77.4	73.9
	P41271	NBL1	Neuroblastoma suppressor of tumorigenicity 1	30.0	41.2
	P16519	NEC2	Neuroendocrine convertase 2	4.7	3.8
	Q92823	NRCAM	Neuronal cell adhesion molecule	5.1	3.9
	Q15818	NPTX1	Neuronal pentraxin‐1	9.6	17.9
	O14773	TPP1	Tripeptidyl‐peptidase 1	2.8	2.7
	P30291	WEE1	Wee1‐like protein kinase	4.2	4.6
*Cell signaling*	Q9HCE7	SMUF1	E3 ubiquitin‐protein ligase SMURF1	2.2	2.4
	Q14571	ITPR2	Inositol 1,4,5‐trisphosphate receptor type 2	244.9	320.2
	O15240	VGF	Neurosecretory protein VGF	10.6	9.5
	P48552	NRIP1	Nuclear receptor‐interacting protein 1	19.3	29.3
	Q9P219	DAPLE	Protein Daple	10.3	13.5
	Q15904	VAS1	V‐type proton ATPase subunit S1	2.4	2.2
*Proteolysis*	P16870	CBPE	Carboxypeptidase E	2.9	2.8
	Q9Y646	CBPQ	Carboxypeptidase Q	No detected in DME	
	Q9NZP8	C1RL	Complement C1r subcomponent‐like protein	24.8	10.0
	Q86UX2	ITIH5	Interalpha‐trypsin inhibitor heavy chain H5	19.6	22.1
	Q9H3G5	CPVL	Probable serine carboxypeptidase CPVL	3.1	5.0
	O75674	TM1L1	TOM1‐like protein 1	No detected in DME	
	Q9Y5W5	WIF1	Wnt inhibitory factor 1	2.6	3.3
*Other*	Q8IVF6	AN18A	Ankyrin repeat domain‐containing prot18A	3.5	3.2
	Q8NE71	ABCF1	ATP‐binding cassette subfamily F member 1	7.0	15.8
	Q9UBZ9	REV1	DNA repair protein REV1	4.9	5.0
	Q96HE7	ERO1A	ERO1‐like protein alpha	3.8	4.3
	O75063	XYLK	Glycosaminoglycan xylosylkinase	17.7	6.0
	Q9UPS6	SET1B	Histone‐lysine *N*‐methyltransferase SETD1B	28.0	44.1
	Q9H1K4	GHC2	Mitochondrial glutamate carrier 2	3.4	4.7
	Q02817	MUC2	Mucin‐2	89.5	21.2
	Q14995	NR1D2	Nuclear receptor subfamily 1D2	22.3	22.1
	O94880	PHF14	PHD finger protein 14	9.3	13.0
	Q6UX71	PXDC2	Plexin domain‐containing protein 2	3.1	3.0
	Q7Z5M8	AB12B	Protein ABHD12B	11.3	15.2
	Q92520	FAM3C	Protein FAM3C	6.1	4.8
	Q9BSG5	RTBDN	Retbindin	3.7	3.3
	Q8IXT5	RB12B	RNA‐binding protein 12B	2.5	2.3
	Q8WVM8	SCFD1	Sec1 family domain‐containing protein 1	61.6	11.7
	P04278	SHBG	Sex hormone‐binding globulin	2.4	2.2
	Q14679	TTLL4	Tubulin polyglutamylase TTLL4	7.6	6.7

The other interesting protein groups that were upregulated in the iERM and MH proteomes are involved in cell motility, including the proteins CCD39, DYH11, SPG17, and EPIPL, which regulate cilium movement and wound healing. In the retina, photoreceptors have unique sensory cilia that are essential for eye health. Patients who have defects in ciliary motility develop retinal degeneration (Brown & Witman, 2014). It has also been shown that primary cilia coordinate a series of signaling pathways and regulate cell migration during the development of and during the process of wound healing (Veland, Awan, Pedersen, Yoder, & Christensen, 2009).

Importantly, specific processes, such as Wnt signaling for iERM samples and the semaphorin‐plexin pathway for MH samples, were also detected in the iERM and MH proteomes (Figure 4a). Aberrant Wnt/beta catenin signaling has been implicated in major inflammatory and neurodegenerative disorders (Marchetti et al., 2013) and in a variety of human hereditary diseases. Currently, the modulation of Wnt signaling is actively studied in the fields of cancer, regenerative medicine, and wound healing (Clevers & Nusse, 2012). However, our understanding of the Wnt pathway is incomplete, and many questions in this field remain unanswered.

The semaphorin‐plexin pathway is also known to have functions in the neural and immune systems, as well as in angiogenesis (Worzfeld & Offermanns, 2014). The intravitreal semaphorin 3A concentration has been previously shown to be increased in patients suffering from diabetic retinopathy (Cerani et al., 2017). However, in the DME proteome in our study, semaphorins, including 3A, were detected at only very low levels, whereas several semaphorins (3A, 3B, 4B, 3F, 7A) and their binging partner, plexin B1, were present at higher levels in the MH proteome, suggesting an important role of semaphorin‐plexin signaling in MH but not in DME. Altogether, the role of the Wnt or semaphorin‐plexin signaling pathway in the MH or iERM processes has not been explored in great detail and therefore requires further study.

### 2.4 The iERM and MH vitreous proteomes display a surprisingly high abundance of neuronal proteins

**Figure 5:**
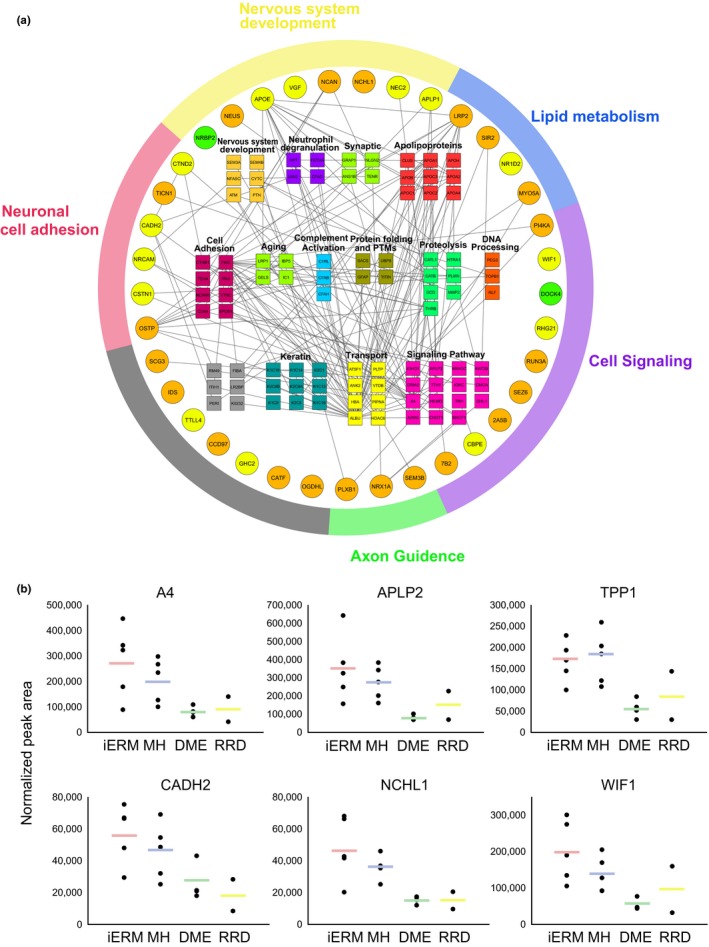
The iERM and MH vitreous proteomes display high abundance of neuronal proteins. (a) Interaction analysis of the differentially abundant neuronal proteins. Thirty‐seven neuronal proteins that were present at higher levels in the iERM and MH proteomes were analyzed using the PINA2 protein interaction public database (green nodes indicate proteins that were upregulated in the iERM samples; orange nodes indicate upregulated proteins in the MH samples; and yellow nodes indicate upregulated proteins in both of the samples). Interaction analysis reveals a total of 90 interacting proteins found in our vitreous analysis, and they are classified based on their biological processes. (b) SWATH MS analysis verifies the increased expression of neuronal proteins in the iERM and MH vitreous, including proteins associated with neurodegeneration (A4, APLP2, and TPP1), cell adhesion (CADH2 and NCHL1), or cell signaling (WIF1). The results are shown as the peak areas detected in the SWATH analysis. The spots represent individual samples, and the lines indicate the mean values

To assess the cellular origins of the upregulated proteins in iERM and MH, we analyzed gene expression profiles using the MediSapiens database, which includes the gene expression data from over 3,000 samples from 60 “healthy” human tissues (http://www.medisapiens.com). Only one clear cluster could be detected, showing the accumulation of neuronal origin proteins in the vitreous proteomes (Figure 4c). To further analyze these neuronal proteins, we used the PINA2 database to derive the protein–protein interactions for these proteins (Figure 5a). According to the PINA2 analysis, the 37 upregulated proteins found in the neuronal cluster have a total of 90 interactors that were identified in our vitreous proteome analysis (Figure 5a, Supporting Information Table S7). The interacting proteins formed several protein groups, the largest of which were the apolipoproteins, keratins, and signaling proteins. Additionally, several proteins involved in neuronal system development and cell adhesion were detected.

Network analysis showed a central role of apolipoproteins in the vitreous proteome (Figure 5a). Previously, APOA1, APOB, APOH, and APOC have been reported as biomarkers for diabetic retinopathy (Loukovaara et al., 2015; Sasongko et al., 2011). However, according to our study, APOE and APOB were more abundant in the iERM and MH samples than in the diabetic control samples, indicating the specific role of these apolipoproteins in iERM and MH conditions. Therefore, our findings suggest that APOB is not a suitable biomarker for diabetic retinopathy.

Moreover, various keratins have been identified as binding partners of upregulated neuronal proteins. Generally, keratins are broadly expressed in epithelial cells, but they have also been detected in normal nervous tissue (Iwatsuki & Suda, 2010). The detection of multiple different keratins in the proteome analysis indicates a specific role of keratins in age‐related eye diseases, even though some keratins can also be contaminants linked to sample preparation. Keratins are a class of intermediate filament proteins that participate in scar formation and wound healing (Martin, 1997). As the iERM is a condition in which a thin sheet of scar tissue grows on the surface of the retina, keratins could also play a role in the iERM process.

Interestingly, many proteins directly associated with neurodegeneration were also detected at higher abundances in the iERM and MH samples than in the DME samples, including amyloid‐like protein 1 and 2 (APLP1 and APLP2), integral membrane protein 2B (ITM2B), amyloid beta A4 protein (A4), and tripeptidyl‐peptidase 1 (TPP1). The presence of a member of the amyloid precursor protein gene family in vitreous the iERM and MH humor suggests that there could be a link between age‐related eye diseases and neurodegenerative diseases in the brain. Similar morphological and functional changes in microglial and neuronal activities, such as those reported in the brain of Alzheimer´s patient, may also occur in the retina (Krantic & Torriglia, 2014). Actually, neurodegenerative processes that have been characterized in central nervous system disorders have also been detected in ocular pathologies, such as glaucoma and age‐related macular degeneration (London, Benhar, & Schwartz, 2013).

### 2.5 SWATH MS analysis for validation of the data‐dependent global proteomics results

Sequential window acquisition of all theoretical mass spectra (SWATH MS) is a recently developed, massively parallel protein targeting technique that features high accuracy and high reproducibility in protein quantification (Gillet et al., 2012). To validate our findings, we performed SWATH MS analysis of vitreous samples from 5 iERM, 5 MH and 4 DME patients. In addition, two rhegmatogenous retinal detachment (RRD) patients were included in the analysis as a second control. A total of 403 proteins could be reliably quantified (Supporting Information Table S8). Clustering analysis showed nice separation of the iERM and MH proteomes from the DME and RRD controls (Supporting Information Figure S2). Several proteins were selected for validation, including proteins associated with neurodegeneration (A4, APLP2, and TPP1), cell adhesion (CADH2 and NCHL1) and cell signaling (WIF1), and these produced very similar expression profiles to those produced by the quantitative shotgun MS approach (Figure 5b). In addition, the SWATH analysis verified the increased expression of an additional 15 neuronal proteins in the iERM and MH vitreous (Supporting Information Figure S3), confirming our speculation about the neurogenesis background of these two diseases. The increased amount of neuronal proteins was not detected in the RDD control samples, which eliminates the possibility that the neuronal proteins being in the vitreous was due to unspecific leakage caused by retinal tearing.

### 2.6 Comparison of the iERM and MH proteomes

**Figure 6:**
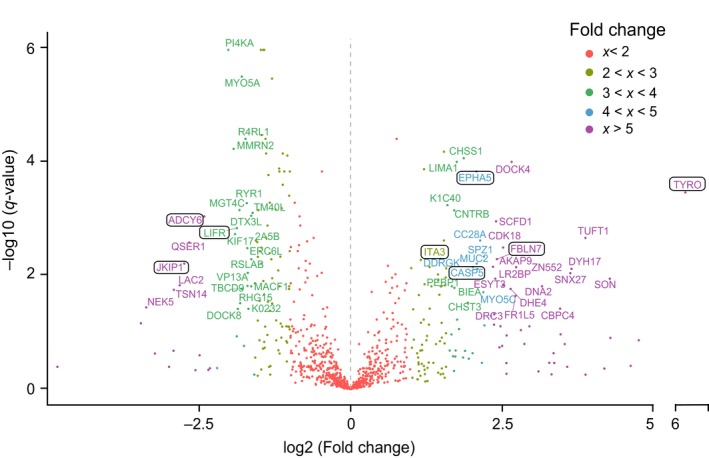
Comparison between iERM and MH reveals several potential iERM biomarkers. Volcano blot analysis of differentially expressed proteins in the iERM and MH samples. The short names of the proteins were given to proteins with a q‐value <0.05 and a fold change >3 (exception: ITA3, fold change: 2.8). Potential iERM biomarker candidates are highlighted

Next, we compared the iERM proteome to the MH proteome to assess for precise biomarkers of these diseases. Altogether, 160 proteins differed significantly between the iERM and MH groups (q‐value <0.05), with 53 proteins being upregulated in the iERM samples (abundance ratio >2) and 65 proteins being upregulated in the MH samples (Figure 6, Supporting Information Table S9). As these upregulated proteins in the iERM and MH groups represent different biological processes and could not show any enrichment pathways, we selected some highly expressed individual proteins that displayed a significantly different abundance in the iERM samples compared to the MH samples as potential biomarker candidates for a diagnosis and prognostic approach.

One of the novel biomarker candidates is tyrosinase (TYRO), which is the rate‐limiting enzyme oxidase responsible for melanin biosynthesis in the RPE of the eye. RPE cells contain different types of melanin granules (Boulton, 2014), making them the probable origin of an increased level of tyrosinase. Melanin has an important role in retinal development and protection against light‐induced oxidative stress, and melanin levels have been associated with AMD (Reinisalo, Putula, Mannermaa, Urtti, & Honkakoski, 2012). Our analysis showed that tyrosinase levels were 70‐fold higher in the iERM eyes than in the MH eyes and 40‐fold higher in the iERM eyes than in the DME eyes, indicating a specific role of tyrosinase in iERM formation.

Fibulin‐7 (FBLN7) is a cell adhesion molecule that is expressed in the retina also. Recently, it was shown to play a role in preventing AMD (Sardell et al., 2016). In addition, it has been found to negatively regulate angiogenesis (de Vega et al., 2014), which could be a mechanism for AMD regulation. FBLN7 interacts with beta1 integrin, which forms a heterodimer with alpha3 integrin (ITA3) which was found to be one of the upregulated proteins in our iERM samples. In the eye, integrin receptors have been closely associated with ocular surface inflammation, vitreolysis, and choroidal and preretinal angiogenesis. Alpha3beta1 integrin mediates the attachment of the vitreous to the retinal surface (Oliveira et al., 2002), which makes these adhesion molecules a potential therapeutic target for iERM.

Caspases are a family of protease enzymes with essential roles in programmed cell death and inflammation. During eye development, cell death allows for the selection of appropriate synaptic connections (Braunger, Demmer, & Tamm, 2014). Dysregulation of programmed cell death, however, has been linked to the pathogenesis of several retinal diseases, including RD and AMD (Kaarniranta, Tokarz, Koskela, Paterno, & Blasiak, 2017; Torriglia, Jaadane, & Lebon, 2016). Caspase‐5 (CASP‐5), a poorly characterized member of the caspase subfamily, was found to be highly upregulated in our iERM sample group. It interplays with caspase‐1 in inflammatory responses in RPE cells, but the functional roles of CASP‐5 in ocular inflammatory diseases are essentially unknown (Bian et al., 2011). EPHA5 receptor tyrosine kinase also plays a key role in the development of the eye and visual system (Pfeiffenberger et al., 2005). However, the association between EPHA5 and retinal dysfunctions has not been reported.

In regard to the MH eyes, three interesting signaling molecules, Janus kinase and microtubule‐interaction protein 1 (JAKMIP1), leukemia inhibitory factor receptor (LIFR), and adenylate cyclase type 6 (ADCY6), were presented at significantly higher levels in the MH proteome than in the iERM or the DME proteome, and they may therefore be considered as potential MH markers. JAKMIP1 interacts with Tyk2, a member of Janus kinase family, which has been shown to control the survival and proliferation of retinal cells (Samardzija et al., 2006). LIFR, together with its ligand, leukemia inhibitory factor (LIF), also participates in neuroprotection by activating an endogenous rescue pathway that protects viable photoreceptor cells (Bürgi, Samardzija, & Grimm, 2009). ADCY6 is a member of adenylate cyclase family that synthesizes cyclic adenosine monophosphate or cyclic AMP from adenosine triphosphate. Two members of the family, ADCY1 and ADCY8, work together to facilitate midline crossing of retinal axons, but the role of ADCY6 in retinal function has not been reported (Xu, Leinwand, Dell, Fried‐Cassorla, & Raper, 2010).

### 2.7 Concluding remarks

Systemic data of intravitreal biochemical factors and signaling pathways related to the age‐related retinal interface diseases iERM and MH are rather scarce. This study utilized label‐free quantitative MS to investigate the vitreous proteomes in iERM and MH eyes. Precise bioinformatics analysis was performed to reveal novel candidate protein groups and signaling pathways involved in the formation of iERM and MH, potentially guiding the development of further pharmacological treatments or therapies. In conclusion, our results illustrate the following: (a) Both iERM and MH have complicated pathological processes involving inflammation, extracellular matrix dysfunction and fibrosis; (b) the surprisingly large number of neuronal proteins analyzed in this study was more abundant in the vitreous proteome from the age‐related eye pathologies of iERM and MH than in those of DME and RRD, indicating the neurodegenerative background of these two age‐related pathologies; (c) Wnt signaling involved in the development of iERM and the semaphorin‐plexin pathway involved in the development of MH formation were novel findings; and (d) the identity of proteins differed significantly between the iERM and MH conditions, from which we identified a candidate list of possible targets for diagnostic, prognostic, and/or therapeutic approaches.

During the last decade, human vitreous proteomics has rapidly expanded the list of potential protein biomarkers and molecular disease pathways. The differences between the pathogeneses of iERM and MH were actively investigated to provide new potential biomarkers to be used for diagnostic and prognostic approaches. We could observe several highly upregulated proteins in the iERM and MH proteomes, which provides further insight into the pathologies of these two age‐related eye diseases. The vitreous proteome atlases of iERM and MH, which were generated in this study, could provide a plethora of information and form the basis of future diagnostic and therapeutic approaches. However, although we were able to provide some novel insight into the pathomechanisms of these two retinal interface diseases that could possibly guide the further development of efficient treatments, other studies are required to better understand these pathologies.

## 3 EXPERIMENTAL PROCEDURES

### 3.1 Patients

This study was conducted according to the Declaration of Helsinki and was approved by the Institutional Review Board of Helsinki University Central Hospital at the University of Helsinki in Finland. Signed informed consent was obtained from each participant before the sampling occurred. Confidentiality of the patient records was maintained when the clinical data were entered into a computer‐based standardized data entry for analysis.

The patients were admitted for primary vitrectomy for the management of the pathologies of iERM, MH or diabetic retinopathy with macular edema (DME) in the Unit of Vitreoretinal Surgery at Helsinki University Central Hospital in Helsinki, Finland. The diagnosis and detection of morphologic retinal pathological changes in each studied eye were confirmed using optical coherence tomography (OCT) prior to surgery. OCT (Stratus OCT; Zeiss) or the spectral domain‐OCT OptoVue RTVue V.5.1 device (OptoVue Inc.) was acquired under mydriatic circumstances. The scan pattern used on Optovue RTVue was a standardized macular protocol, retina MM6, which gives accurate thickness measurements in the fovea, perifovea (<3 mm), and parafovea (<6 mm). The measurements of the central retinal thickness in the innermost foveal area were recorded and analyzed pre‐ and postoperatively.

The exclusion criteria among the iERM and MH patients were vitreous hemorrhage and inflammation, other retinal inflammatory or retinal vascular disorders (retinitis, choroiditis, uveitis, or retinal vein occlusion), RD, AMD, or previous ocular trauma. One eye from the iERM group was excluded because of previous systemic borreliosis, and one eye from the MH group was excluded because of previous glaucoma surgery (trabeculectomy). A total of 56 eyes from 56 patients underwent primary transconjunctival microincision vitrectomy for iERM (*n* = 26), MH (*n* = 21), DME (*n* = 7), or RRD (*n* = 2) between 2006 and 2017. The clinical systemic and ocular characteristics of the study patients are given in Table 1.

### 3.2 Vitreous sample collection

Undiluted vitreous samples (up to 1,000 µl) were collected at the start of the standard three‐port pars plana vitrectomy (25‐gauge or 23‐gauge, Constellation Vision System, Alcon Instruments, Inc.) without an infusion of artificial fluid. The samples were collected by manual aspiration into a syringe via the vitrectomy with the cutting function activated. The samples were immediately frozen at −70°C until MS analysis.

In the vitrectomy, if the vitreous was attached to the posterior retina, PVD was induced by suction with the vitrectomy probe over the optic disk. To visualize and identify the posterior hyaloid epiretinal membranes and the internal limiting membrane, intravitreal vital dyes were used (chromovitrectomy). Diluted indocyanine green, MembraneBlue‐Dual^®^ or ILM‐blue^®^ (D.O.R.C. Zuidland, Netherland) dye‐assisted ERM ± ILM or plain ILM peeling was performed. Peeling of ERM and ILM was carried out using the pinch‐peel technique with fine‐tipped forceps. In all of the MH eyes, a fluid‐air exchange was performed with a subsequent gas exchange.

### 3.3 Sample preparation for the LC‐MS

The collected patient vitreous samples were centrifuged at 18,000 *g* for 15 min at 4°C to clear the samples from cellular debris. Average protein concentrations (mg/ml) of the vitreous samples were determined using the Bicinchoninic Acid (BCA) Protein Assay Kit (Pierce, Thermo Scientific) according to the manufacturer's instructions. 100 μg of total protein per sample was taken for LC‐MS analysis. Urea was added to a final concentration 1 M, and the proteins were reduced with tris(2‐carboxyethyl)phosphine and alkylated with iodoacetamide. The proteins were then digested to peptides with Sequencing Grade Modified Trypsin (Promega) using a 1:50 enzyme:protein ratio at 37°C o/n. The resulting tryptic peptides were purified with C18 microspin columns (Nest Group, Southborough, MA, USA) before they were subjected to LC‐MS/MS analysis.

### 3.4 LC‐MS/MS analysis

The LC‐MS/MS analysis was carried out with a Q Exactive ESI‐quadrupole‐orbitrap mass spectrometer coupled to an EASY‐nLC 1000 nanoflow LC (Thermo Fisher Scientific), using the Xcalibur version 3.1.66.10 (Thermo Scientific). The tryptic peptide sample mixture was loaded from autosampler into a C18‐packed precolumn (Acclaim PepMap™100 75 μm × 2 cm, 3 μm, 100 Å, Thermo Scientific) in buffer A (1% acetonitrile, 0.1% formic acid). Peptides were transferred onward to C18‐packed analytical column (Acclaim PepMap™100 75 μm × 15 cm, 2 μm, 100 Å, Thermo Scientific) and separated with a 120‐min linear gradient from 5% to 35% of buffer B (98% acetonitrile, 0.1% formic acid) at a flow rate of 300 nl/min. MS analysis was performed in a data‐dependent acquisition in a positive‐ion mode. MS spectra were acquired from m/z 200 to m/z 2,000 with a resolution of 70,000 with a full AGC target value of 1,000,000 ions and a maximal injection time of 100 ms in the profile mode. The 10 most abundant ions for which the charge states were 2+ to 7+ were selected for subsequent fragmentation (higher energy collisional dissociation (HCD), and MS/MS spectra were acquired with a resolution of 17,500 with an AGC target value of 5,000, a maximal injection time of 100 ms, and the lowest mass fixed at m/z 120, in the centroid mode. The dynamic exclusion duration was 30 s.

### 3.5 MS1 quantification and protein identification

Progenesis LC‐MS software (v4.1, Nonlinear Dynamics Limited, Tyne, UK) was used to obtain the MS1 intensities of the peptides for label‐free quantification. The run with the greatest similarity to all of the other runs was automatically selected as the alignment reference. All of the runs were then aligned to a reference run automatically and further adjusted manually. The retention time was limited to 10–130 min excluding the first and last 10 min of the recorded data. Only peptides with charge states from +2 to +7 were allowed. For the protein identification with the MS/MS‐scan, data acquired from Progenesis LC‐MS were searched against the human component of the UniProtKB database (release 2016_06 with 20,154 entries) using SEQUEST search engine in Proteome Discoverer™ software (version 1.4). Carbamidomethylation (+57.021464 Da) of cysteine residues was used as a static modification, and oxidation (+15.994491 Da) of methionine was used as a dynamic modification. Precursor mass tolerance and fragment mass tolerance were set to <15 ppm and 0.05 Da, respectively. A maximum of two missed cleavages were allowed. The results were filtered to a maximum false discovery rate of 0.05. For the protein identification, the peptide spectrum match was set to ≥2.

To validate the sample reproducibility in regard to the feature alignment and detection level, five samples were analyzed in technical replicates. The distribution of the MS1 feature alignment and MS1 quantitation level correlation showed extremely high reproducibility (Supporting Information Table S2).

### 3.6 Dot blot analysis

Five microgram of total protein from the vitreous samples was dot blotted to a nitrocellulose membrane using the Bio‐Rad 96‐well dot blot system (Bio‐Rad) according to the manufacturer's instructions. Six antibodies against the differentially expressed proteins between the samples were selected based on the availability of high‐quality antibodies suitable for Western blotting. Signals were visualized using the Amersham ECL Western blotting analysis system (GE Healthcare). The dot blots were analyzed and quantified using Dot Blot Analyzer for ImageJ30. For each of the proteins, the Pearson´s correlation was calculated between the corresponding MS1 and dot blot intensities.

### 3.7 Data processing

Statistical significance tests of abundance changes between the iERM, MH, and DME eyes were conducted with Student's *t* test. An abundance change with a *q*‐value of 0.05 or less was considered a significant change. *q*‐Values were used instead of conventional p‐values to maximize the power of the statistical test. Hierarchical cluster was performed by Pearson correlation (both samples and identifications; average linkage) using Morpheus software (https://software.broadinstitute.org/morpheus). The boxplots and volcano blot analysis were produced using R version 3.3.2. Gene Ontology (GO) annotations were obtained from DAVID bioinformatics resources (Huang, Sherman, & Lempicki, 2009). The cellular locations of the identified proteins, being either intracellular, transmembrane, or extracellular, were extracted from the Phobius predictor (Käll et al., 2004). The MediSapiens database (http://www.medisapiens.com) was used to study the gene expression levels across healthy human tissues (Kilpinen et al., 2008). The PINA2 protein interaction database was used to obtain the known protein–protein interactions.

### 3.8 SWATH analysis

The samples were analyzed both in the shotgun experiment for spectral library building and in the SWATH MS mode for quantitative analysis using a Sciex 6600 TripleTOFF MS coupled to an Eksigent nanoLC with a microelectrospray ionization source. MS was operated in the positive‐ion mode. In the shotgun experiment, information‐dependent acquisition (IDA) was implemented using a “top 30” method. Specifically, a 100 ms survey scan was performed in the m/z range of 100–1,500, and the top 30 ions above the intensity threshold of 150 counts were selected for subsequent MS/MS scans with an accumulation time of 250 ms. In the SWATH experiment, a 100 ms survey scan was performed in the m/z range of 400–1,250, followed by serial consecutive SWATH scans. Spectral library was performed using the human Uniprot database (release 04_2018 with 20,341 entries) supplemented with common contaminants using Paragon algorithm via ProteinPilot (v4.5, AB SCIEX; Shilov et al., 2007). The “Thorough ID” mode was selected, which automatically adjusts the mass tolerance to fit the high‐resolution MS and MS/MS data. Peak extraction of the SWATH data was performed using the SWATH micro app embedded in PeakView (ver2.0, AB SCIEX) with the following parameters: 75 ppm m/z tolerance for the targeted transition, five transitions selected per peptide, peptide identification FDR < 1%, and exclusion of shared peptides. Retention time calibration was performed based on PepCalMix (AB SCIEX) elution profiles. The results are shown as the total area normalized ion peak areas.

### 3.9 Data availability

The peptide raw data have been uploaded to the MassIVE public repository (https://massive.ucsd.edu.), the MassIVE ID MSV000081839.

## CONFLICT OF INTEREST

The authors declare no competing financial interests.

## AUTHOR CONTRIBUTIONS

T.Ö. was responsible for the experimental design and performance and for the data analysis; F.T. analyzed and interpreted the data; H.G. was responsible for the planning and discussion of the study and for the reviewing of the manuscript; S.L. collected the vitreous samples; T.Ö., S.L., and M.V. designed the study and wrote the article. All of the authors contributed to the writing process and approved the final version of the article.
