# Supplementary material for: Systems pathology analysis identifies neurodegenerative nature of age‐related vitreoretinal interface diseases
Source: Aging Cell. 2018 Jul 2;17(5):e12809. doi: 10.1111/acel.12809 (PMC6156470; doi:10.1111/acel.12809)

Supplemental Figure S1. Correlation between dot blot analysis and MS1 quantification of vitreous samples

Kininogen (P01042)

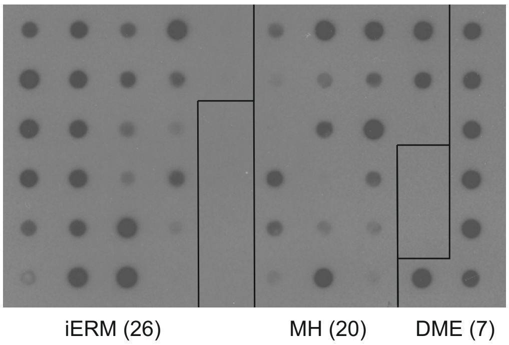

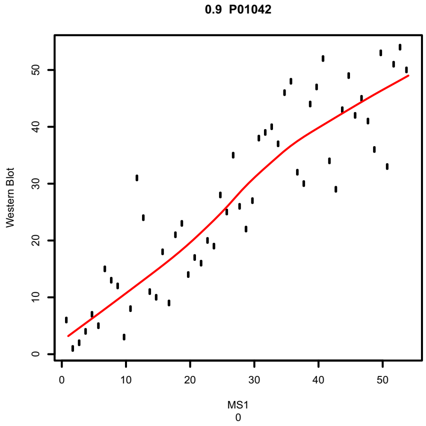

Complement C9 (P02748)

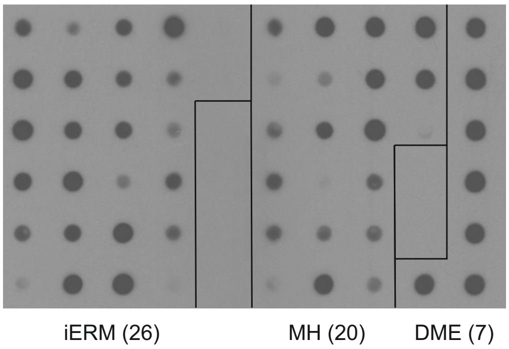

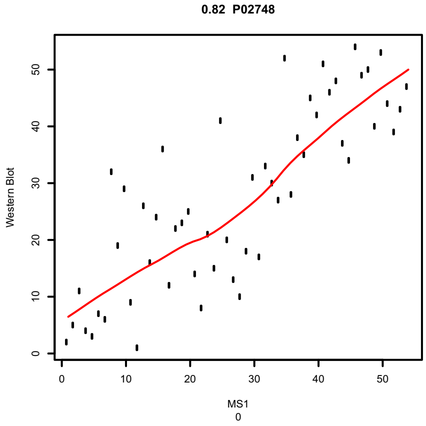

Beta-2-glycoprotein 1 (P02749)

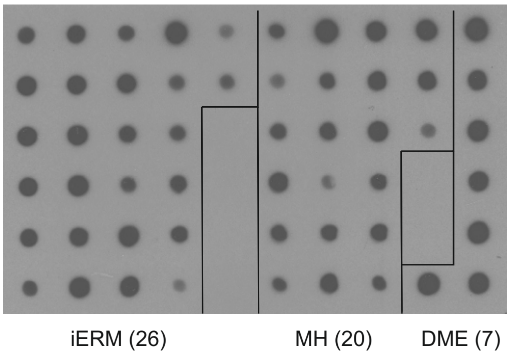

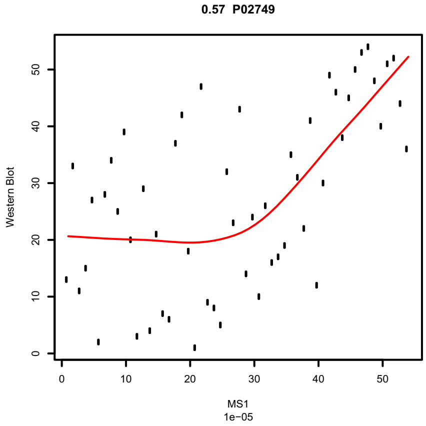

ITIH1 (P19827)

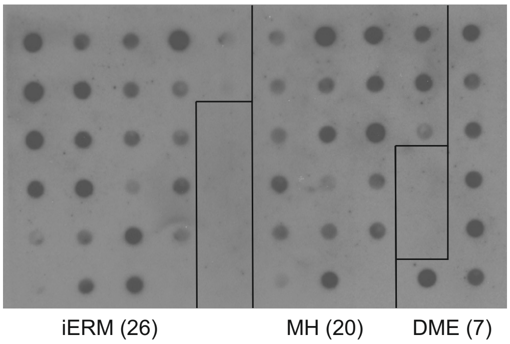

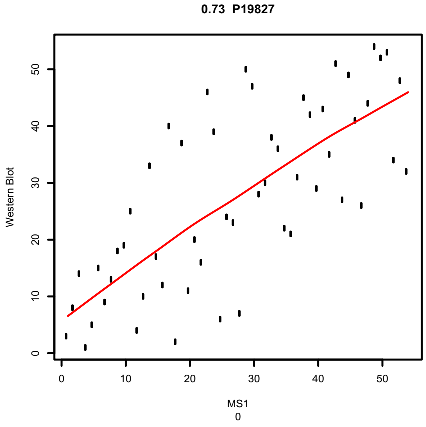

Serum amyloid A-4 protein (P35542)

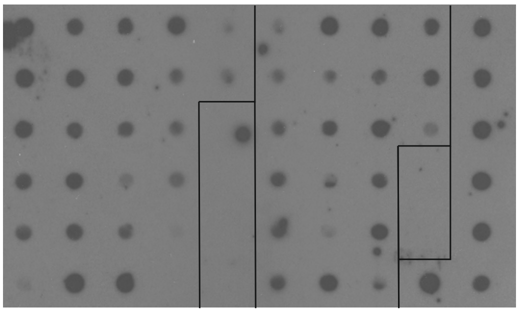

iERM (26) MH (20) DME (7)

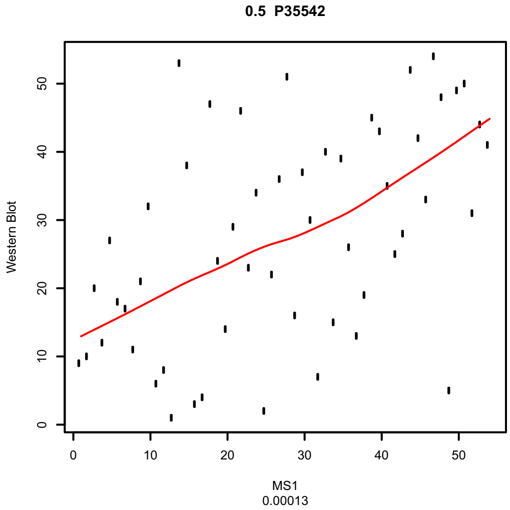

Plasminogen (P00747)

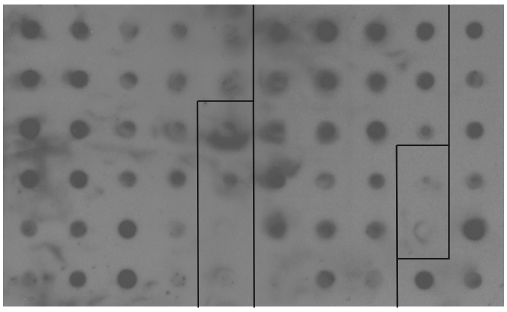

iERM (26) MH (20) DME (7)

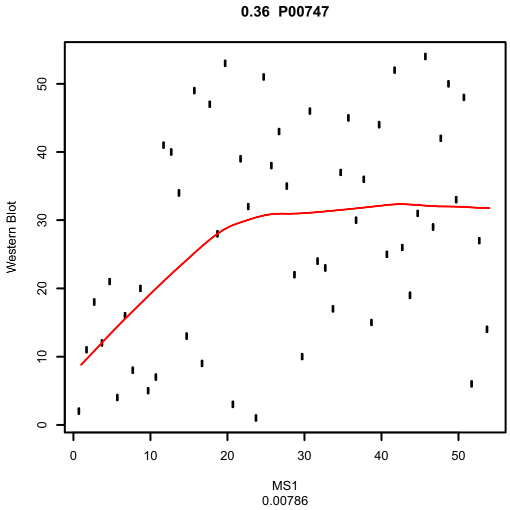

Supplement: Supplementary file 1 [file ACEL-17-e12809-s001.pdf]
