# Supplementary figures and images for: Systems pathology analysis identifies neurodegenerative nature of age‐related vitreoretinal interface diseases
Source: Aging Cell. 2018 Jul 2;17(5):e12809. doi: 10.1111/acel.12809 (PMC6156470; doi:10.1111/acel.12809)

Figure S2

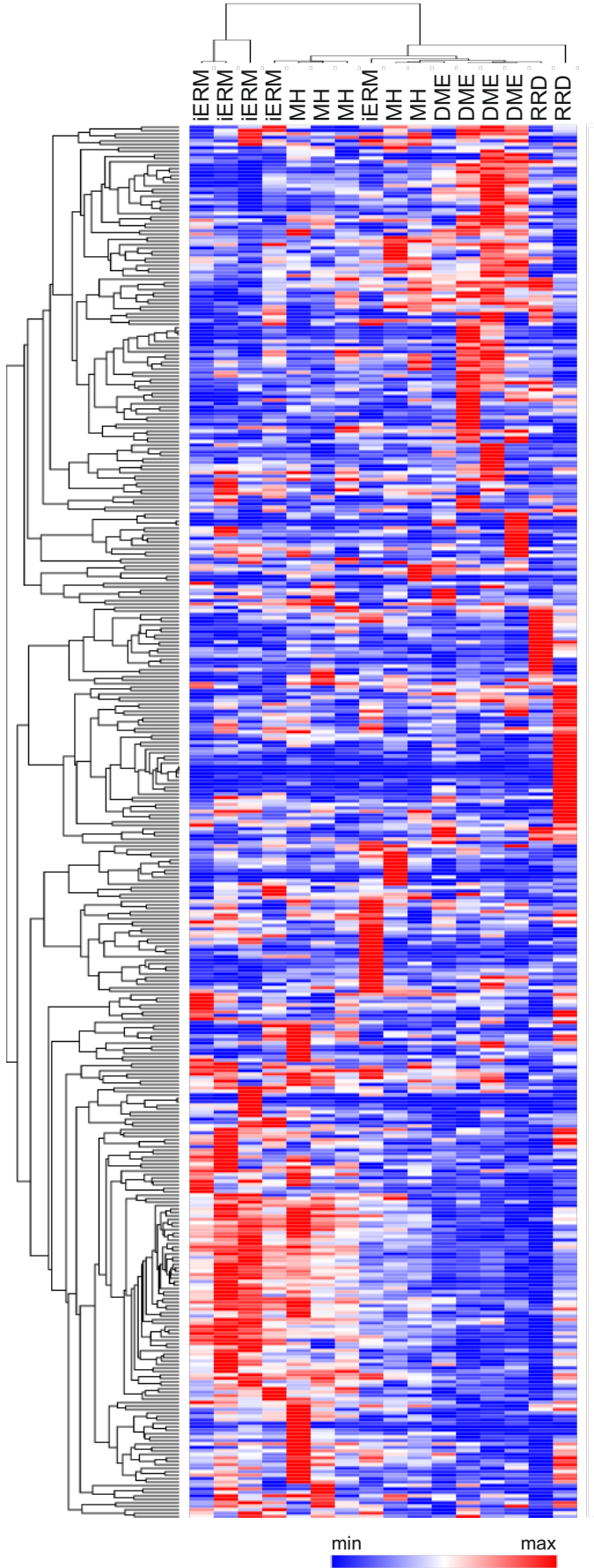

Supplement: Supplementary file 2 [file ACEL-17-e12809-s002.pdf]

Figure S3

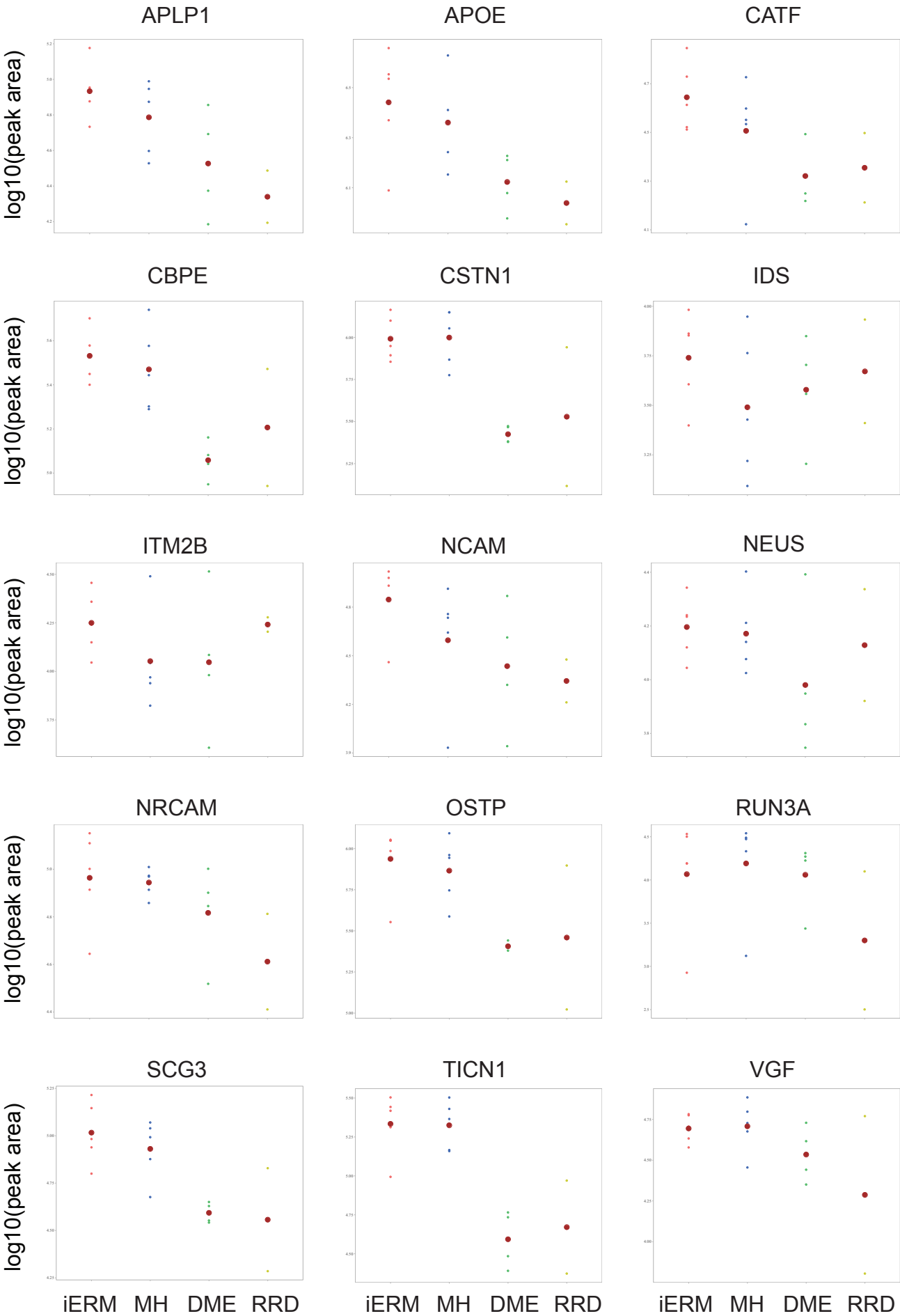

Supplement: Supplementary file 3 [file ACEL-17-e12809-s003.pdf]
