# Supplementary material for: Systems pathology analysis identifies neurodegenerative nature of age‐related vitreoretinal interface diseases
Source: Aging Cell. 2018 Jul 2;17(5):e12809. doi: 10.1111/acel.12809 (PMC6156470; doi:10.1111/acel.12809)
Supplement: Supplementary file 4 [file ACEL-17-e12809-s004.pdf]

Supplemental Table S1: Patient demographics and operated eye characteristics.
Sex: 1=female, 2=male; Diagnosis: pucker, macular hole; Protein concentration in vitreous (mg/ml)
Operated eye: 1=right, 2=left; Intraocular lens (IOL): 1=yes, 0= no (natural crystalline lens)
Cystoid macular edema (CME): 0=no, 1=yes
Preoperative laser photocoagulation: 0=no, 1=partial panretinal photocoagulation
Internal limiting membrane (ILM) peeling during vitrectomy: 0=no, 1=yes; Smoking: 0=no, 1=yes
American Society of Anesthesiologists physical status calssification system (ASA)(1-4)
Body Mass Index (BMI) kg/m2; Statin therapy: 0= no,1=yes; Hypertension: 0=no, 1=yes
Intraocular pressure (IOP) measured preoperatively (mmHg)
Optical coherence tomography measured preoperatively (µm)
Blood pressure, Systolic mmHg
Blood pressure, Diastolic mmHg
Alignment (%): Progenesis MS1 intensity aligment percentages

No.	Sex	Age	Diagnosis	Protein concentration (mg/ml)	Eye	IOL	CME/DME	preopLaser	ILM-rexis	Smoking	ASA	BMI	Statin	Hypertension	IOPpre (mmHg)	OCTpre (µm)	BPsyst	BPDiast	aligment %
Pucker_1	1	66	Pucker	4,5	1	1	1	0	1	0	2	20	0	0	14	367	126	75	65,5
Pucker_2	2	64	Pucker	2,7	1	1	0	0	1	0	2	26,6	0	1	17	350	137	78	57,9
Pucker_3	2	73	Pucker	3,6	1	0	0	0	1	0	2	27	0	0	12	426	167	78	50,2
Pucker_4	2	70	Pucker	3,7	2	0	0	0	1	0	2	27,1	0	1	17	340	152	88	64,6
Pucker_5	1	65	Pucker	3,1	1	0	1	0	1	0	2	30,8	0	1	17	397	165	85	73,3
Pucker_6	2	71	Pucker	4,3	2	1	0	0	1	0	2	21,6	0	0	20	508	164	89	66,9
Pucker_7	2	61	Pucker	3,2	2	0	0	0	0	0	1	28,1	0	0	13	493	181	83	65
Pucker_8	1	65	Pucker	4,6	2	0	0	0	1	0	2	21,4	0	0	16		124	75	61,2
Pucker_9	1	64	Pucker	4,5	2	0	1	0	1	0	1	23,2	0	0	13		164	93	64,5
Pucker_10	2	83	Pucker	1,2	1	1	0	0	1	0	3	24,5	1	1	9	373	126	73	53
Pucker_11	1	71	Pucker	4,7	2	0	0	0	1	0	3	23,7	1	0	18		160	105	64,7
Pucker_12	2	58	Pucker	4,7	1	0	0	0	1	0	3	29,8	0	1	14		163	102	55,8
Pucker_13	1	68	Pucker	3,5	2	0	0	0	1	0	3	22,5	1	0	14	446	146	86	69,7
Pucker_14	2	66	Pucker	3,9	1	1	0	1		0		25,8	0	0	12	402			62
Pucker_15	2	67	Pucker	3,5	2	0	0	0	1	0	2		0	0	13				74,7
Pucker_16	1	67	Pucker	5,8	1	0	0	0	1	0	2	34,9	0	1	17	441	170	85	65,2
Pucker_17	2	68	Pucker	4,3	1	1	0	0	1	0	2		1	1	11	370			61,2
Pucker_18	2	75	Pucker	5,6	1	1	1	1	1	0	3	25,4	1	0	9	575			50
Pucker_19	2	71	Pucker	4,3	1	1	0	0	1	0	3	28	0	1	9	320	185	89	51,5
Pucker_20	1	69	Pucker	4,1	2	0	1	0	1	0	2	22,6	0	0	17	370	141	92	89,2
Pucker_21	2	71	Pucker	4,6	1	1	0	0	1	0	1		0	0	11				86,9
Pucker_22	1	73	Pucker	5,7	1	0	1	0	1	0	2		0	0	16				90,7
Pucker_23	1	75	Pucker	5,1	1	1	1	0	1	0			1				170	55	86,5
Pucker_24	1	66	Pucker	4,0	1	0	0	0	0	0			0				167	89	69,7
Pucker_25	1	72	Pucker	4,7	2	1	0	0	0	0			1	1	16		207	92	78,3
Pucker_26	1	66	Pucker	4,9	1	0	1	0	1	0	1	30,8	0	0	21	404	170	95	67,4
MH_1	1	66	MH	3,8	1	1	1	0	1	0	1	23,3	0	0	15	350	118	68	79,9
MH_2	1	59	MH	4,2	1	0	1	0	1	0	1	23,2	0	0	14		165	102	79,8
MH_3	1	75	MH	3,5	2	0	1	0	1	0	2	26,8	1	0	17	461	177	93	87,1
MH_4	1	74	MH	4,0	1	1	1	0	1	0	3	33,2	1	1	9	270	139	70	88,9
MH_5	1	68	MH	5,0	2	0	0	0	1	0	2	22,8	0	0	19	288	149	85	88,7
MH_6	1	66	MH	2,9	1	0	1	0	1	0	2		0	1	16	270			83
MH_7	2	59	MH	5,1	1	0	1	0	1	0		30,5			27				52
MH_8	2	68	MH	3,3	1	0	0	0	1	0	2	24,7	1	0	9	428	142	92	90
MH_9	1	70	MH	3,7	2	0	0	0	1	0	1	25,9	0	0	20				REF
MH_10	1	75	MH	4,5	2	0	1	0	1	0			0	0	20				80,8
MH_11	1	70	MH	4,5	2	0	1	0	1	0			1	0	9				91,4
MH_12	1	57	MH	4,3	2	0	0	0	1	0	1	25,6	0	1	17		170	98	60,3
MH_13	2	70	MH	5,5	2	1	0	0	1	0			0	0	13				67
MH_14	1	71	MH	3,0	1	0	0	0	1	0	2	33,4	0	0	19	472	171	96	90
MH_15	1	71	MH	4,5	1	0	0	0	1	1	2	22,3	0	0	22	413	174	91	77,2
MH_16	1	64	MH	6,0	2	0	1	0	1	1	2	20,2	0	0	15	376	148	92	79,9
MH_17	1	63	MH	2,0	1	0	1	0	1	0		24,8	0		11		166	90	88,6
MH_18	1	65	MH	6,0	1	0	0	0	1	0			1	1	15		192	106	80,8
MH_19	1	82	MH	1,6			0						0						80,9
MH_20	2	76	MH	3,3	1	0	1	0	1	0		18,5	1	0	22		160	90	85,8
MH_21	1	72	MH	4,5	2	1	0	0	1	0	2	19,1	0	1	14	366	146	77	81,8
DME_1	1	38	DR	2,6	2	1	1	0	1	0	2	21,3	1	0	9	687	133	88	57,5
DME_2	1	78	DR	1,7	1	1	0	0	0	0	3	27,1	1	1	15		185	86	55,2
DME_3	2	82	DR	4,4	2	0	1	1	1	0	2	23,4	0	0	14		160	76	46,8
DME_4	2	79	DR	2,6	1	1	0	0	0	0	4	23,6	0	1	12	320	177	79	48,3
DME_5	2	68	DR	3,3	1	1	1	1	1	0	3	20,8	0	0	15	356	159	90	43,1
DME_6	1	66	DR	4,3	1	0	1	1	1	0	3	33,8	0	1	19	360	174	89	59,5
DME_7	1	75	DR	4,7	2	1	1	1	0	0	3	34,9	1	1	16		169	76	54,5
RRD_1	2	52	RRD	4,0	1	0	0	0	0	0	2	34,9	0	0	16		134	93	-
RRD_2	2	54	RRD	13,3	1	0	0	0	0	0	1	21,9	0	0	14		122	72	-
