# Supplementary material for: Systems pathology analysis identifies neurodegenerative nature of age‐related vitreoretinal interface diseases
Source: Aging Cell. 2018 Jul 2;17(5):e12809. doi: 10.1111/acel.12809 (PMC6156470; doi:10.1111/acel.12809)
Supplement: Supplementary file 5 [file ACEL-17-e12809-s005.pdf]

Supplemental Table S2: Reproducibility of the used analysis pipeline.
Selected samples has two technical runs (replicates). The retention times (RT) of each of the runs are aligned to the reference run. The table contains Pearson correlations of MS1 values (intensity) and alignment score difference between two replicates of

Samples	Correlation between replicates
**Sample_Pucker1**	0,999906229
**Sample_Pucker2**	0,999999138
**Sample_Pucker3**	0,999946116
**Sample_MH22**	0,999804508
**Sample_MH15**	0,999820937
**Average correlation**	**0,999895386**

Alignment score (replicate 1)	Alignment score (replicate 2)	Alignment score difference (replicate 1- replicate 2)
77,50 %	72,10 %	5,40 %
Reference	95,70 %	4,30 %
55,20 %	57,30 %	2,10 %
64,20 %	65,00 %	0,80 %
53,80 %	54,70 %	0,90 %
**Average alignment score difference**		**2,70 %**
