# Supplementary material for: Systems pathology analysis identifies neurodegenerative nature of age‐related vitreoretinal interface diseases
Source: Aging Cell. 2018 Jul 2;17(5):e12809. doi: 10.1111/acel.12809 (PMC6156470; doi:10.1111/acel.12809)
Supplement: Supplementary file 6 [file ACEL-17-e12809-s006.pdf]

Supplemental Table S3: The identified vitreous protein with a peptide-spectrum match score (PSM) ≥ 2. The cellular locations of identified proteins, being either intracellular, transmembrane or extracellular, were extracted from Phobius predictor.

Accession	Description	Coverage	# Unique Peptides	# Peptides	# PSMs	Localization
P02768	Serum albumin OS=Homo sapiens GN=ALB PE=1 SV=2 - [ALBU_HUMAN]	90,97	98	98	53147	Extracellular
P02787	Serotransferrin OS=Homo sapiens GN=TF PE=1 SV=3 - [TRFE_HUMAN]	76,22	66	68	12907	Extracellular
P01024	Complement C3 OS=Homo sapiens GN=C3 PE=1 SV=2 - [CO3_HUMAN]	60,55	98	98	7628	Extracellular
P02790	Hemopexin OS=Homo sapiens GN=HPX PE=1 SV=2 - [HEMO_HUMAN]	75,54	36	36	7313	Extracellular
P10745	Retinol-binding protein 3 OS=Homo sapiens GN=RBP3 PE=1 SV=2 - [RET3_HUMAN]	65,52	50	50	7221	Extracellular
P00450	Ceruloplasmin OS=Homo sapiens GN=CP PE=1 SV=1 - [CERU_HUMAN]	68,26	50	50	6420	Extracellular
P0C0L5	Complement C4-B OS=Homo sapiens GN=C4B PE=1 SV=2 - [CO4B_HUMAN]	53,44	3	71	5216	Extracellular
P0C0L4	Complement C4-A OS=Homo sapiens GN=C4A PE=1 SV=2 - [CO4A_HUMAN]	53,15	3	71	4968	Extracellular
P36955	Pigment epithelium-derived factor OS=Homo sapiens GN=SERPINF1 PE=1 SV=4 - [PEDF_HUMAN]	66,75	23	24	4952	Extracellular
P10909	Clusterin OS=Homo sapiens GN=CLU PE=1 SV=1 - [CLUS_HUMAN]	40,09	23	23	4855	Extracellular
P01857	Ig gamma-1 chain C region OS=Homo sapiens GN=IGHG1 PE=1 SV=1 - [IGHG1_HUMAN]	63,94	7	16	4218	Extracellular
P02774	Vitamin D-binding protein OS=Homo sapiens GN=GC PE=1 SV=1 - [VTDB_HUMAN]	76,58	34	34	3891	Extracellular
P01023	Alpha-2-macroglobulin OS=Homo sapiens GN=A2M PE=1 SV=3 - [A2MG_HUMAN]	57,19	66	66	3872	Extracellular
P01860	Ig gamma-3 chain C region OS=Homo sapiens GN=IGHG3 PE=1 SV=2 - [IGHG3_HUMAN]	54,11	6	16	3391	Extracellular
P06727	Apolipoprotein A-IV OS=Homo sapiens GN=APOA4 PE=1 SV=3 - [APOA4_HUMAN]	64,9	34	34	3027	Extracellular
P02647	Apolipoprotein A-I OS=Homo sapiens GN=APOA1 PE=1 SV=1 - [APOA1_HUMAN]	68,16	29	29	2986	Extracellular
P13645	Keratin, type I cytoskeletal 10 OS=Homo sapiens GN=KRT10 PE=1 SV=6 - [K1C10_HUMAN]	59,25	28	33	2867	Extracellular
P02766	Transthyretin OS=Homo sapiens GN=TTR PE=1 SV=1 - [TTHY_HUMAN]	77,55	15	15	2862	Extracellular
P04264	Keratin, type II cytoskeletal 1 OS=Homo sapiens GN=KRT1 PE=1 SV=6 - [K2C1_HUMAN]	51,09	30	35	2621	Extracellular
P00747	Plasminogen OS=Homo sapiens GN=PLG PE=1 SV=2 - [PLMN_HUMAN]	69,01	49	50	2594	Extracellular
P01859	Ig gamma-2 chain C region OS=Homo sapiens GN=IGHG2 PE=1 SV=2 - [IGHG2_HUMAN]	48,16	5	13	2488	Extracellular
P01861	Ig gamma-4 chain C region OS=Homo sapiens GN=IGHG4 PE=1 SV=1 - [IGHG4_HUMAN]	51,07	4	11	2383	Extracellular
P06396	Gelsolin OS=Homo sapiens GN=GSN PE=1 SV=1 - [GELS_HUMAN]	59,34	34	34	2311	Extracellular
P00751	Complement factor B OS=Homo sapiens GN=CFB PE=1 SV=2 - [CFAB_HUMAN]	50	33	33	2296	Extracellular
P01008	Antithrombin-III OS=Homo sapiens GN=SERPINC1 PE=1 SV=1 - [ANT3_HUMAN]	56,68	30	30	1951	Extracellular
Q06481	Amyloid-like protein 2 OS=Homo sapiens GN=APLP2 PE=1 SV=2 - [APLP2_HUMAN]	34,86	26	27	1858	Transmembrane
P02649	Apolipoprotein E OS=Homo sapiens GN=APOE PE=1 SV=1 - [APOE_HUMAN]	66,56	22	22	1854	Extracellular
P05155	Plasma protease C1 inhibitor OS=Homo sapiens GN=SERPING1 PE=1 SV=2 - [IC1_HUMAN]	32,8	15	15	1769	Extracellular
P01876	Ig alpha-1 chain C region OS=Homo sapiens GN=IGHA1 PE=1 SV=2 - [IGHA1_HUMAN]	54,96	6	14	1756	Extracellular
P00738	Haptoglobin OS=Homo sapiens GN=HP PE=1 SV=1 - [HPT_HUMAN]	61,82	27	27	1740	Extracellular
O94985	Calsyntenin-1 OS=Homo sapiens GN=CLSTN1 PE=1 SV=1 - [CSTN1_HUMAN]	34,25	29	29	1728	Transmembrane
P02765	Alpha-2-HS-glycoprotein OS=Homo sapiens GN=AHSG PE=1 SV=1 - [FETUA_HUMAN]	45,5	11	11	1721	Extracellular
P41222	Prostaglandin-H2 D-isomerase OS=Homo sapiens GN=PTGDS PE=1 SV=1 - [PTGDS_HUMAN]	51,58	8	8	1701	Extracellular
P01034	Cystatin-C OS=Homo sapiens GN=CST3 PE=1 SV=1 - [CYTC_HUMAN]	67,81	11	11	1559	Extracellular
P02652	Apolipoprotein A-II OS=Homo sapiens GN=APOA2 PE=1 SV=1 - [APOA2_HUMAN]	69	9	9	1558	Extracellular
P07339	Cathepsin D OS=Homo sapiens GN=CTSD PE=1 SV=1 - [CATD_HUMAN]	44,42	17	17	1551	Extracellular
P02763	Alpha-1-acid glycoprotein 1 OS=Homo sapiens GN=ORM1 PE=1 SV=1 - [A1AG1_HUMAN]	45,77	7	10	1531	Extracellular
Q9UBP4	Dickkopf-related protein 3 OS=Homo sapiens GN=DKK3 PE=1 SV=2 - [DKK3_HUMAN]	56,86	15	15	1510	Extracellular
P35908	Keratin, type II cytoskeletal 2 epidermal OS=Homo sapiens GN=KRT2 PE=1 SV=2 - [K22E_HUMAN]	62,75	22	29	1498	Extracellular
P00734	Prothrombin OS=Homo sapiens GN=F2 PE=1 SV=2 - [THRB_HUMAN]	52,73	24	24	1491	Extracellular
P04217	Alpha-1B-glycoprotein OS=Homo sapiens GN=A1BG PE=1 SV=4 - [A1BG_HUMAN]	57,78	17	17	1459	Extracellular
P35527	Keratin, type I cytoskeletal 9 OS=Homo sapiens GN=KRT9 PE=1 SV=3 - [K1C9_HUMAN]	52,49	24	25	1407	Extracellular
P01834	Ig kappa chain C region OS=Homo sapiens GN=IGKC PE=1 SV=1 - [IGKC_HUMAN]	80,19	6	6	1404	Extracellular
P01011	Alpha-1-antichymotrypsin OS=Homo sapiens GN=SERPINA3 PE=1 SV=2 - [AACT_HUMAN]	49,17	24	24	1376	Extracellular
P01009	Alpha-1-antitrypsin OS=Homo sapiens GN=SERPINA1 PE=1 SV=3 - [A1AT_HUMAN]	55,74	25	26	1324	Extracellular
P01877	Ig alpha-2 chain C region OS=Homo sapiens GN=IGHA2 PE=1 SV=3 - [IGHA2_HUMAN]	42,94	2	10	1321	Extracellular
P01042	Kininogen-1 OS=Homo sapiens GN=KNG1 PE=1 SV=2 - [KNG1_HUMAN]	35,09	21	21	1266	Extracellular
P02749	Beta-2-glycoprotein 1 OS=Homo sapiens GN=APOH PE=1 SV=3 - [APOH_HUMAN]	56,81	16	16	1259	Extracellular
Q13822	Ectonucleotide pyrophosphatase/phosphodiesterase family member 2 OS=Homo sapiens GN=ENPP2	40,21	28	28	1154	Transmembrane
P22352	Glutathione peroxidase 3 OS=Homo sapiens GN=GPX3 PE=1 SV=2 - [GPX3_HUMAN]	37,17	8	8	1127	Extracellular
Q12805	EGF-containing fibulin-like extracellular matrix protein 1 OS=Homo sapiens GN=EFEMP1 PE=1 SV=2	42,19	15	15	1115	Extracellular
P43652	Afamin OS=Homo sapiens GN=AFM PE=1 SV=1 - [AFAM_HUMAN]	41,07	24	24	1090	Extracellular
P10451	Osteopontin OS=Homo sapiens GN=SPP1 PE=1 SV=1 - [OSTP_HUMAN]	38,22	10	10	1082	Extracellular
P01019	Angiotensinogen OS=Homo sapiens GN=AGT PE=1 SV=1 - [ANGT_HUMAN]	37,94	13	13	1059	Extracellular
P19652	Alpha-1-acid glycoprotein 2 OS=Homo sapiens GN=ORM2 PE=1 SV=2 - [A1AG2_HUMAN]	45,77	7	10	1041	Extracellular
P05156	Complement factor I OS=Homo sapiens GN=CFI PE=1 SV=2 - [CFAI_HUMAN]	36,54	21	21	1037	Extracellular
P04196	Histidine-rich glycoprotein OS=Homo sapiens GN=HRG PE=1 SV=1 - [HRG_HUMAN]	25,9	16	16	1025	Extracellular
Q9Y5W5	Wnt inhibitory factor 1 OS=Homo sapiens GN=WIF1 PE=1 SV=3 - [WIF1_HUMAN]	34,04	11	11	1017	Extracellular
P19823	Inter-alpha-trypsin inhibitor heavy chain H2 OS=Homo sapiens GN=ITIH2 PE=1 SV=2 - [ITIH2_HUMA	30,55	19	19	977	Extracellular
Q9UBM4	Opticin OS=Homo sapiens GN=OPTC PE=1 SV=1 - [OPT_HUMAN]	45,78	13	13	973	Extracellular
P16870	Carboxypeptidase E OS=Homo sapiens GN=CPE PE=1 SV=1 - [CBPE_HUMAN]	49,58	20	20	927	Extracellular
Q9HCB6	Spondin-1 OS=Homo sapiens GN=SPON1 PE=1 SV=2 - [SPON1_HUMAN]	26,64	16	16	917	Extracellular
P04004	Vitronectin OS=Homo sapiens GN=VTN PE=1 SV=1 - [VTNC_HUMAN]	26,78	12	12	891	Extracellular
P02750	Leucine-rich alpha-2-glycoprotein OS=Homo sapiens GN=LRG1 PE=1 SV=2 - [A2GL_HUMAN]	45,24	11	12	887	Extracellular
P02748	Complement component C9 OS=Homo sapiens GN=C9 PE=1 SV=2 - [CO9_HUMAN]	44,19	19	19	871	Extracellular
P08603	Complement factor H OS=Homo sapiens GN=CFH PE=1 SV=4 - [CFAH_HUMAN]	35,99	27	31	857	Extracellular
Q9Y6R7	IgGFc-binding protein OS=Homo sapiens GN=FCGBP PE=1 SV=3 - [FCGBP_HUMAN]	18,7	47	47	824	Extracellular
Q14624	Inter-alpha-trypsin inhibitor heavy chain H4 OS=Homo sapiens GN=ITIH4 PE=1 SV=4 - [ITIH4_HUMA	25,48	19	19	775	Extracellular
P0CG05	Ig lambda-2 chain C regions OS=Homo sapiens GN=IGLC2 PE=1 SV=1 - [LAC2_HUMAN]	69,81	1	5	768	Extracellular
P05067	Amyloid beta A4 protein OS=Homo sapiens GN=APP PE=1 SV=3 - [A4_HUMAN]	33,38	18	19	670	Transmembrane
O75326	Semaphorin-7A OS=Homo sapiens GN=SEMA7A PE=1 SV=1 - [SEM7A_HUMAN]	36,04	20	20	665	Transmembrane
P61769	Beta-2-microglobulin OS=Homo sapiens GN=B2M PE=1 SV=1 - [B2MG_HUMAN]	42,86	5	5	635	Extracellular
B9A064	Immunoglobulin lambda-like polypeptide 5 OS=Homo sapiens GN=IGLL5 PE=2 SV=2 - [IGLL5_HUM/	38,32	2	6	573	Extracellular
P02675	Fibrinogen beta chain OS=Homo sapiens GN=FGB PE=1 SV=2 - [FIBB_HUMAN]	47,66	19	19	569	Extracellular
P02751	Fibronectin OS=Homo sapiens GN=FN1 PE=1 SV=4 - [FINC_HUMAN]	19,87	31	31	559	Extracellular
P02533	Keratin, type I cytoskeletal 14 OS=Homo sapiens GN=KRT14 PE=1 SV=4 - [K1C14_HUMAN]	37,29	3	19	559	Extracellular
P13647	Keratin, type II cytoskeletal 5 OS=Homo sapiens GN=KRT5 PE=1 SV=3 - [K2C5_HUMAN]	29,32	13	21	551	Extracellular
P19827	Inter-alpha-trypsin inhibitor heavy chain H1 OS=Homo sapiens GN=ITIH1 PE=1 SV=3 - [ITIH1_HUMA	25,69	16	16	551	Extracellular
P23142	Fibulin-1 OS=Homo sapiens GN=FBLN1 PE=1 SV=4 - [FBLN1_HUMAN]	33	15	15	547	Extracellular
P02679	Fibrinogen gamma chain OS=Homo sapiens GN=FGG PE=1 SV=3 - [FIBG_HUMAN]	33,11	13	13	538	Extracellular
Q7Z7G0	Target of Nesh-SH3 OS=Homo sapiens GN=ABI3BP PE=1 SV=1 - [TARSH_HUMAN]	17,95	15	15	510	Extracellular
O14773	Tripeptidyl-peptidase 1 OS=Homo sapiens GN=TPP1 PE=1 SV=2 - [TPP1_HUMAN]	36,23	11	11	505	Extracellular
P08697	Alpha-2-antiplasmin OS=Homo sapiens GN=SERPINF2 PE=1 SV=3 - [A2AP_HUMAN]	37,88	15	15	500	Extracellular
P02671	Fibrinogen alpha chain OS=Homo sapiens GN=FGA PE=1 SV=2 - [FIBA_HUMAN]	30,02	23	23	500	Extracellular
P08185	Corticosteroid-binding globulin OS=Homo sapiens GN=SERPINA6 PE=1 SV=1 - [CBG_HUMAN]	27,41	7	7	487	Extracellular
P08779	Keratin, type I cytoskeletal 16 OS=Homo sapiens GN=KRT16 PE=1 SV=4 - [K1C16_HUMAN]	44,82	9	21	485	Extracellular
P48668	Keratin, type II cytoskeletal 6C OS=Homo sapiens GN=KRT6C PE=1 SV=3 - [K2C6C_HUMAN]	32,27	10	20	477	Extracellular
P51884	Lumican OS=Homo sapiens GN=LUM PE=1 SV=2 - [LUM_HUMAN]	30,18	9	10	476	Extracellular
P02753	Retinol-binding protein 4 OS=Homo sapiens GN=RBP4 PE=1 SV=3 - [RET4_HUMAN]	45,77	8	8	470	Extracellular
O43505	Beta-1,4-glucuronyltransferase 1 OS=Homo sapiens GN=B4GAT1 PE=1 SV=1 - [B4GA1_HUMAN]	42,41	10	10	457	Extracellular
Q14515	SPARC-like protein 1 OS=Homo sapiens GN=SPARCL1 PE=1 SV=2 - [SPRL1_HUMAN]	37,8	18	18	427	Extracellular
Q92765	Secreted frizzled-related protein 3 OS=Homo sapiens GN=FRZB PE=1 SV=2 - [SFRP3_HUMAN]	16,92	5	5	415	Extracellular
P43251	Biotinidase OS=Homo sapiens GN=BTD PE=1 SV=2 - [BTD_HUMAN]	18,23	7	7	415	Extracellular
P24592	Insulin-like growth factor-binding protein 6 OS=Homo sapiens GN=IGFBP6 PE=1 SV=1 - [IBP6_HUM/	30	6	6	412	Extracellular
P51693	Amyloid-like protein 1 OS=Homo sapiens GN=APLP1 PE=1 SV=3 - [APLP1_HUMAN]	26,62	13	13	411	Transmembrane
P02760	Protein AMBP OS=Homo sapiens GN=AMBP PE=1 SV=1 - [AMBP_HUMAN]	30,4	9	9	409	Extracellular
Q96PD5	N-acetylmuramoyl-L-alanine amidase OS=Homo sapiens GN=PGLYRP2 PE=1 SV=1 - [PGRP2_HUM	38,19	13	13	385	Extracellular
Q9NQ79	Cartilage acidic protein 1 OS=Homo sapiens GN=CRTAC1 PE=1 SV=2 - [CRAC1_HUMAN]	29,35	16	16	377	Extracellular
Q04695	Keratin, type I cytoskeletal 17 OS=Homo sapiens GN=KRT17 PE=1 SV=2 - [K1C17_HUMAN]	28,94	3	15	362	Extracellular
P13611	Versican core protein OS=Homo sapiens GN=VCAN PE=1 SV=3 - [CSPG2_HUMAN]	4,06	11	11	352	Extracellular
P10643	Complement component C7 OS=Homo sapiens GN=C7 PE=1 SV=2 - [CO7_HUMAN]	25,86	14	14	332	Extracellular
Q16270	Insulin-like growth factor-binding protein 7 OS=Homo sapiens GN=IGFBP7 PE=1 SV=1 - [IBP7_HUM/	46,1	11	11	332	Extracellular
Q08629	Testican-1 OS=Homo sapiens GN=SPOCK1 PE=1 SV=1 - [TICN1_HUMAN]	34,62	10	11	328	Extracellular
P27169	Serum paraoxonase/arylesterase 1 OS=Homo sapiens GN=PON1 PE=1 SV=3 - [PON1_HUMAN]	39,72	9	9	296	Extracellular
O15537	Retinoschisin OS=Homo sapiens GN=RS1 PE=1 SV=2 - [XLRS1_HUMAN]	29,91	6	6	291	Extracellular
P08571	Monocyte differentiation antigen CD14 OS=Homo sapiens GN=CD14 PE=1 SV=2 - [CD14_HUMAN]	34,67	10	10	290	Extracellular

Q92823	Neuronal cell adhesion molecule OS=Homo sapiens GN=NRCAM PE=1 SV=3 - [NRCAM_HUMAN]	11,5	12	12	290	Transmembrane
P08727	Keratin, type I cytoskeletal 19 OS=Homo sapiens GN=KRT19 PE=1 SV=4 - [K1C19_HUMAN]	18,5	2	9	279	Extracellular
Q8IZJ3	C3 and PZP-like alpha-2-macroglobulin domain-containing protein 8 OS=Homo sapiens GN=CPAMD8	12,31	15	15	276	Extracellular
P00736	Complement C1r subcomponent OS=Homo sapiens GN=C1R PE=1 SV=2 - [C1R_HUMAN]	28,23	13	14	275	Extracellular
Q03591	Complement factor H-related protein 1 OS=Homo sapiens GN=CFHR1 PE=1 SV=2 - [FHR1_HUMAN]	59,09	3	11	268	Extracellular
P00748	Coagulation factor XII OS=Homo sapiens GN=F12 PE=1 SV=3 - [FA12_HUMAN]	16,91	8	8	268	Extracellular
Q9UHG2	ProSAAS OS=Homo sapiens GN=PCSK1N PE=1 SV=1 - [PCSK1_HUMAN]	31,92	5	5	262	Extracellular
Q8WXD2	Secretogranin-3 OS=Homo sapiens GN=SCG3 PE=1 SV=3 - [SCG3_HUMAN]	41,88	14	14	262	Extracellular
Q8N475	Follistatin-related protein 5 OS=Homo sapiens GN=FSTL5 PE=2 SV=2 - [FSTL5_HUMAN]	25,15	14	16	261	Extracellular
Q7Z794	Keratin, type II cytoskeletal 1b OS=Homo sapiens GN=KRT77 PE=2 SV=3 - [K2C1B_HUMAN]	13,15	5	8	247	Extracellular
P13646	Keratin, type I cytoskeletal 13 OS=Homo sapiens GN=KRT13 PE=1 SV=4 - [K1C13_HUMAN]	14,63	2	7	236	Extracellular
P07358	Complement component C8 beta chain OS=Homo sapiens GN=C8B PE=1 SV=3 - [CO8B_HUMAN]	38,41	17	17	227	Extracellular
P07357	Complement component C8 alpha chain OS=Homo sapiens GN=C8A PE=1 SV=2 - [CO8A_HUMAN]	21,58	8	8	226	Extracellular
P00746	Complement factor D OS=Homo sapiens GN=CFD PE=1 SV=5 - [CFAD_HUMAN]	52,96	8	8	213	Extracellular
P12109	Collagen alpha-1(VI) chain OS=Homo sapiens GN=COL6A1 PE=1 SV=3 - [CO6A1_HUMAN]	15,76	10	10	211	Extracellular
P01031	Complement C5 OS=Homo sapiens GN=C5 PE=1 SV=4 - [CO5_HUMAN]	11,99	16	16	210	Extracellular
P36222	Chitinase-3-like protein 1 OS=Homo sapiens GN=CHI3L1 PE=1 SV=2 - [CH3L1_HUMAN]	39,43	11	11	209	Extracellular
P07225	Vitamin K-dependent protein S OS=Homo sapiens GN=PROS1 PE=1 SV=1 - [PROS_HUMAN]	18,79	11	11	200	Extracellular
Q96S96	Phosphatidylethanolamine-binding protein 4 OS=Homo sapiens GN=PEBP4 PE=1 SV=3 - [PEBP4_H	32,6	5	5	199	Extracellular
P05546	Heparin cofactor 2 OS=Homo sapiens GN=SERPIND1 PE=1 SV=3 - [HEP2_HUMAN]	28,26	13	13	198	Extracellular
P10645	Chromogranin-A OS=Homo sapiens GN=CHGA PE=1 SV=7 - [CMGA_HUMAN]	24,95	7	8	198	Extracellular
Q15904	V-type proton ATPase subunit S1 OS=Homo sapiens GN=ATP6AP1 PE=1 SV=2 - [VAS1_HUMAN]	18,51	6	6	192	Transmembrane
P16035	Metalloproteinase inhibitor 2 OS=Homo sapiens GN=TIMP2 PE=1 SV=2 - [TIMP2_HUMAN]	33,64	7	7	191	Extracellular
O15240	Neurosecretory protein VGF OS=Homo sapiens GN=VGF PE=1 SV=2 - [VGF_HUMAN]	17,56	7	7	191	Extracellular
P06681	Complement C2 OS=Homo sapiens GN=C2 PE=1 SV=2 - [CO2_HUMAN]	22,74	13	13	190	Extracellular
P02656	Apolipoprotein C-III OS=Homo sapiens GN=APOC3 PE=1 SV=1 - [APOC3_HUMAN]	48,48	3	3	188	Extracellular
Q99972	Myocilin OS=Homo sapiens GN=MYOC PE=1 SV=2 - [MYOC_HUMAN]	22,82	9	9	187	Extracellular
P13671	Complement component C6 OS=Homo sapiens GN=C6 PE=1 SV=3 - [CO6_HUMAN]	18,74	11	11	186	Extracellular
Q12841	Follistatin-related protein 1 OS=Homo sapiens GN=FSTL1 PE=1 SV=1 - [FSTL1_HUMAN]	31,82	7	7	183	Extracellular
Q9BSG5	Retbindin OS=Homo sapiens GN=RTBDN PE=2 SV=2 - [RTBDN_HUMAN]	32,75	4	4	183	Extracellular
P05060	Secretogranin-1 OS=Homo sapiens GN=CHGB PE=1 SV=2 - [SCG1_HUMAN]	19,79	10	10	180	Extracellular
P55083	Microfibril-associated glycoprotein 4 OS=Homo sapiens GN=MFAP4 PE=1 SV=2 - [MFAP4_HUMAN]	22,35	4	4	179	Extracellular
P09871	Complement C1s subcomponent OS=Homo sapiens GN=C1S PE=1 SV=1 - [C1S_HUMAN]	15,99	9	9	174	Extracellular
Q5T7N2	LINE-1 type transposase domain-containing protein 1 OS=Homo sapiens GN=L1TD1 PE=1 SV=1 - [L	1,04	1	1	174	Extracellular
Q14118	Dystroglycan OS=Homo sapiens GN=DAG1 PE=1 SV=2 - [DAG1_HUMAN]	8,16	6	6	174	Transmembrane
P60709	Actin, cytoplasmic 1 OS=Homo sapiens GN=ACTB PE=1 SV=1 - [ACTB_HUMAN]	22,93	2	5	172	Extracellular
P02654	Apolipoprotein C-I OS=Homo sapiens GN=APOC1 PE=1 SV=1 - [APOC1_HUMAN]	26,51	4	4	168	Extracellular
Q9BU40	Chordin-like protein 1 OS=Homo sapiens GN=CHRDL1 PE=1 SV=1 - [CRDL1_HUMAN]	15,56	5	5	165	Extracellular
P39060	Collagen alpha-1(XVIII) chain OS=Homo sapiens GN=COL18A1 PE=1 SV=5 - [COIA1_HUMAN]	6,39	7	7	163	Extracellular
P07602	Prosaposin OS=Homo sapiens GN=PSAP PE=1 SV=2 - [SAP_HUMAN]	16,98	8	8	161	Extracellular
P05452	Tetranectin OS=Homo sapiens GN=CLEC3B PE=1 SV=3 - [TETN_HUMAN]	40,1	6	6	159	Extracellular
Q53EL9	Seizure protein 6 homolog OS=Homo sapiens GN=SEZ6 PE=1 SV=2 - [SEZ6_HUMAN]	15,79	10	10	152	Transmembrane
P36980	Complement factor H-related protein 2 OS=Homo sapiens GN=CFHR2 PE=1 SV=1 - [FHR2_HUMAN]	36,3	1	6	150	Extracellular
Q99969	Retinoic acid receptor responder protein 2 OS=Homo sapiens GN=RARRES2 PE=1 SV=1 - [RARR2	31,29	3	3	146	Extracellular
P19022	Cadherin-2 OS=Homo sapiens GN=CDH2 PE=1 SV=4 - [CADH2_HUMAN]	10,26	7	7	144	Transmembrane
O94915	Protein furry homolog-like OS=Homo sapiens GN=FRYL PE=1 SV=2 - [FRYL_HUMAN]	1,26	2	3	142	Extracellular
Q12860	Contactin-1 OS=Homo sapiens GN=CNTN1 PE=1 SV=1 - [CNTN1_HUMAN]	10,02	6	6	140	Transmembrane
Q5T011	Protein SZT2 OS=Homo sapiens GN=SZT2 PE=1 SV=3 - [SZT2_HUMAN]	0,47	1	2	139	Extracellular
O15195	Villin-like protein OS=Homo sapiens GN=VILL PE=2 SV=3 - [VILL_HUMAN]	4,32	2	2	135	Extracellular
Q9Y4C0	Neurexin-3 OS=Homo sapiens GN=NRXN3 PE=1 SV=4 - [NRX3A_HUMAN]	7,61	9	10	133	Transmembrane
P01766	Ig heavy chain V-III region BRO OS=Homo sapiens PE=1 SV=1 - [HV305_HUMAN]	30,83	2	4	132	Extracellular
P25311	Zinc-alpha-2-glycoprotein OS=Homo sapiens GN=AZGP1 PE=1 SV=2 - [ZA2G_HUMAN]	31,88	7	7	130	Extracellular
P07360	Complement component C8 gamma chain OS=Homo sapiens GN=C8G PE=1 SV=3 - [CO8G_HUMAN	40,1	6	6	129	Extracellular
P01620	Ig kappa chain V-III region SIE OS=Homo sapiens PE=1 SV=1 - [KV302_HUMAN]	31,19	2	2	126	not_matched
P07477	Trypsin-1 OS=Homo sapiens GN=PRSS1 PE=1 SV=1 - [TRY1_HUMAN]	8,1	1	1	33	Extracellular
Q9BXP8	Pappalysin-2 OS=Homo sapiens GN=PAPPA2 PE=1 SV=4 - [PAPP2_HUMAN]	11,17	14	14	118	Extracellular
Q9NR48	Histone-lysine N-methyltransferase ASH1L OS=Homo sapiens GN=ASH1L PE=1 SV=2 - [ASH1L_HU	0,44	1	2	118	Extracellular
P55058	Phospholipid transfer protein OS=Homo sapiens GN=PLTP PE=1 SV=1 - [PLTP_HUMAN]	15,62	6	6	114	Extracellular
Q16610	Extracellular matrix protein 1 OS=Homo sapiens GN=ECM1 PE=1 SV=2 - [ECM1_HUMAN]	23,33	9	9	111	Extracellular
O75787	Renin receptor OS=Homo sapiens GN=ATP6AP2 PE=1 SV=2 - [RENR_HUMAN]	24	7	7	110	Transmembrane
P02747	Complement C1q subcomponent subunit C OS=Homo sapiens GN=C1QC PE=1 SV=3 - [C1QC_HUM	13,88	3	3	109	Extracellular
O00533	Neural cell adhesion molecule L1-like protein OS=Homo sapiens GN=CHL1 PE=1 SV=4 - [NCHL1_HU	10,51	10	10	109	Transmembrane
P15586	N-acetylglucosamine-6-sulfatase OS=Homo sapiens GN=GNS PE=1 SV=3 - [GNS_HUMAN]	21,56	8	9	108	Extracellular
Q92520	Protein FAM3C OS=Homo sapiens GN=FAM3C PE=1 SV=1 - [FAM3C_HUMAN]	22,91	4	4	107	Extracellular
Q17R60	Interphotoreceptor matrix proteoglycan 1 OS=Homo sapiens GN=IMPG1 PE=1 SV=2 - [IMPG1_HUMA	11,29	8	8	107	Extracellular
Q96IY4	Carboxypeptidase B2 OS=Homo sapiens GN=CPB2 PE=1 SV=2 - [CBPB2_HUMAN]	30,02	8	8	107	Extracellular
Q7Z5M8	Protein ABHD12B OS=Homo sapiens GN=ABHD12B PE=2 SV=1 - [AB12B_HUMAN]	1,66	1	1	106	Extracellular
P98164	Low-density lipoprotein receptor-related protein 2 OS=Homo sapiens GN=LRP2 PE=1 SV=3 - [LRP2	1,83	7	7	106	Transmembrane
Q14767	Latent-transforming growth factor beta-binding protein 2 OS=Homo sapiens GN=LTBP2 PE=1 SV=3 -	5,16	7	7	105	Extracellular
P12035	Keratin, type II cytoskeletal 3 OS=Homo sapiens GN=KRT3 PE=1 SV=3 - [K2C3_HUMAN]	8,12	1	7	103	Extracellular
Q9BQ16	Testican-3 OS=Homo sapiens GN=SPOCK3 PE=1 SV=2 - [TICN3_HUMAN]	5,73	1	2	98	Extracellular
O76013	Keratin, type I cuticular Ha6 OS=Homo sapiens GN=KRT36 PE=2 SV=1 - [KRT36_HUMAN]	5,14	1	2	98	Extracellular
O60216	Double-strand-break repair protein rad21 homolog OS=Homo sapiens GN=RAD21 PE=1 SV=2 - [RAD	2,06	1	1	97	Extracellular
Q8WWN8	Arf-GAP with Rho-GAP domain, ANK repeat and PH domain-containing protein 3 OS=Homo sapiens (	1,04	1	1	96	Extracellular
O00468	Agrin OS=Homo sapiens GN=AGRN PE=1 SV=5 - [AGRIN_HUMAN]	7,98	12	12	96	Extracellular
Q7RTS7	Keratin, type II cytoskeletal 74 OS=Homo sapiens GN=KRT74 PE=1 SV=2 - [K2C74_HUMAN]	7,75	1	5	96	Extracellular
P68871	Hemoglobin subunit beta OS=Homo sapiens GN=HBB PE=1 SV=2 - [HBB_HUMAN]	88,44	7	12	95	Extracellular
Q08380	Galectin-3-binding protein OS=Homo sapiens GN=LGALS3BP PE=1 SV=1 - [LG3BP_HUMAN]	7,18	4	4	95	Extracellular
Q6UX71	Plexin domain-containing protein 2 OS=Homo sapiens GN=PLXDC2 PE=1 SV=1 - [PXDC2_HUMAN]	14,56	6	6	95	Transmembrane
O00391	Sulfhydryl oxidase 1 OS=Homo sapiens GN=QSOX1 PE=1 SV=3 - [QSOX1_HUMAN]	6,43	4	4	94	Transmembrane
P08253	72 kDa type IV collagenase OS=Homo sapiens GN=MMP2 PE=1 SV=2 - [MMP2_HUMAN]	15,15	8	8	93	Extracellular
P13987	CD59 glycoprotein OS=Homo sapiens GN=CD59 PE=1 SV=1 - [CD59_HUMAN]	18,75	2	2	92	Extracellular
P02458	Collagen alpha-1(II) chain OS=Homo sapiens GN=COL2A1 PE=1 SV=3 - [CO2A1_HUMAN]	4,77	6	6	91	Extracellular
P18065	Insulin-like growth factor-binding protein 2 OS=Homo sapiens GN=IGFBP2 PE=1 SV=2 - [IBP2_HUMA	18,77	4	4	91	Extracellular
P01617	Ig kappa chain V-II region TEW OS=Homo sapiens PE=1 SV=1 - [KV204_HUMAN]	17,7	2	2	91	not_matched
P07998	Ribonuclease pancreatic OS=Homo sapiens GN=RNASE1 PE=1 SV=4 - [RNAS1_HUMAN]	35,26	3	3	90	Extracellular
P25092	Heat-stable enterotoxin receptor OS=Homo sapiens GN=GUCY2C PE=1 SV=2 - [GUC2C_HUMAN]	1,49	1	1	90	Transmembrane
P08294	Extracellular superoxide dismutase [Cu-Zn] OS=Homo sapiens GN=SOD3 PE=1 SV=2 - [SODE_HUM	15,42	3	3	89	Extracellular
Q92752	Tenascin-R OS=Homo sapiens GN=TNR PE=1 SV=3 - [TENR_HUMAN]	9,94	10	10	89	Extracellular
Q16769	Glutaminyl-peptide cyclotransferase OS=Homo sapiens GN=QPCT PE=1 SV=1 - [QPCT_HUMAN]	16,62	4	4	88	Extracellular
Q6EMK4	Vasorin OS=Homo sapiens GN=VASN PE=1 SV=1 - [VASN_HUMAN]	5,94	3	3	88	Transmembrane
P35542	Serum amyloid A-4 protein OS=Homo sapiens GN=SAA4 PE=1 SV=2 - [SAA4_HUMAN]	30	4	4	87	Extracellular
P14618	Pyruvate kinase PKM OS=Homo sapiens GN=PKM PE=1 SV=4 - [KPYM_HUMAN]	10,73	5	5	86	Extracellular
Q9NNX1	Tuftelin OS=Homo sapiens GN=TUFT1 PE=1 SV=1 - [TUFT1_HUMAN]	5,9	1	1	85	Extracellular
P61916	Epididymal secretory protein E1 OS=Homo sapiens GN=NPC2 PE=1 SV=1 - [NPC2_HUMAN]	41,06	7	7	82	Extracellular
P07858	Cathepsin B OS=Homo sapiens GN=CTSB PE=1 SV=3 - [CATB_HUMAN]	10,32	3	3	81	Extracellular
A5A3E0	POTE ankyrin domain family member F OS=Homo sapiens GN=POTEF PE=1 SV=2 - [POTEF_HUMA	4,19	1	4	81	Transmembrane
P03952	Plasma kallikrein OS=Homo sapiens GN=KLKB1 PE=1 SV=1 - [KLKB1_HUMAN]	13,17	6	6	79	Extracellular
O14594	Neurocan core protein OS=Homo sapiens GN=NCAN PE=1 SV=3 - [NCAN_HUMAN]	5,3	5	6	77	Extracellular
P35858	Insulin-like growth factor-binding protein complex acid labile subunit OS=Homo sapiens GN=IGFALS I	22,48	9	9	77	Extracellular
P69905	Hemoglobin subunit alpha OS=Homo sapiens GN=HBA1 PE=1 SV=2 - [HBA_HUMAN]	83,8	9	9	76	Extracellular
Q13315	Serine-protein kinase ATM OS=Homo sapiens GN=ATM PE=1 SV=4 - [ATM_HUMAN]	0,39	1	1	76	Extracellular
Q02818	Nucleobindin-1 OS=Homo sapiens GN=NUCB1 PE=1 SV=4 - [NUCB1_HUMAN]	27,11	10	10	74	Extracellular
Q96GW7	Brevican core protein OS=Homo sapiens GN=BCAN PE=1 SV=2 - [PGCB_HUMAN]	6,92	4	5	73	Extracellular
Q86V88	Magnesium-dependent phosphatase 1 OS=Homo sapiens GN=MDP1 PE=1 SV=1 - [MGDP1_HUMAN	5,68	1	1	72	Extracellular
P05090	Apolipoprotein D OS=Homo sapiens GN=APOD PE=1 SV=1 - [APOD_HUMAN]	25,93	5	5	69	Extracellular
P19013	Keratin, type II cytoskeletal 4 OS=Homo sapiens GN=KRT4 PE=1 SV=4 - [K2C4_HUMAN]	7,49	1	4	69	Extracellular
Q13387	C-Jun-amino-terminal kinase-interacting protein 2 OS=Homo sapiens GN=MAPK8IP2 PE=1 SV=2 - [J	2,43	1	1	67	Extracellular
Q9NZP8	Complement C1r subcomponent-like protein OS=Homo sapiens GN=C1RL PE=1 SV=2 - [C1RL_HUM	7,39	2	3	66	Extracellular
Q562R1	Beta-actin-like protein 2 OS=Homo sapiens GN=ACTBL2 PE=1 SV=2 - [ACTBL_HUMAN]	9,04	1	2	66	Extracellular
P13591	Neural cell adhesion molecule 1 OS=Homo sapiens GN=NCAM1 PE=1 SV=3 - [NCAM1_HUMAN]	10,96	6	6	65	Transmembrane
P09486	SPARC OS=Homo sapiens GN=SPARC PE=1 SV=1 - [SPRC_HUMAN]	25,74	6	6	64	Extracellular
P02042	Hemoglobin subunit delta OS=Homo sapiens GN=HBD PE=1 SV=2 - [HBD_HUMAN]	57,82	4	9	62	Extracellular
P98160	Basement Transmembrane-specific heparan sulfate proteoglycan core protein OS=Homo sapiens GN	3,14	10	10	62	Extracellular
P35555	Fibrillin-1 OS=Homo sapiens GN=FBN1 PE=1 SV=3 - [FBN1_HUMAN]	1,92	4	4	61	Extracellular

Q6MZW2	Follistatin-related protein 4 OS=Homo sapiens GN=FSTL4 PE=2 SV=3 - [FSTL4_HUMAN]	8,08	3	6	61	Extracellular
P01717	Ig lambda chain V-IV region Hil OS=Homo sapiens PE=1 SV=1 - [LV403_HUMAN]	17,76	1	1	60	Extracellular
Q9UBR2	Cathepsin Z OS=Homo sapiens GN=CTSZ PE=1 SV=1 - [CATZ_HUMAN]	18,48	4	4	60	Extracellular
O00584	Ribonuclease T2 OS=Homo sapiens GN=RNASET2 PE=1 SV=2 - [RNT2_HUMAN]	19,92	4	4	59	Extracellular
P01765	Ig heavy chain V-III region TIL OS=Homo sapiens PE=1 SV=1 - [HV304_HUMAN]	22,61	1	3	58	Extracellular
Q86UD1	Out at first protein homolog OS=Homo sapiens GN=OAF PE=2 SV=1 - [OAF_HUMAN]	6,59	2	2	58	Extracellular
Q86UX2	Inter-alpha-trypsin inhibitor heavy chain H5 OS=Homo sapiens GN=ITIH5 PE=2 SV=2 - [ITIH5_HUMA	5,52	5	5	57	Extracellular
P22304	Iduronate 2-sulfatase OS=Homo sapiens GN=IDS PE=1 SV=1 - [IDS_HUMAN]	7,82	3	3	57	Extracellular
Q99574	Neuroserpin OS=Homo sapiens GN=SERPINI1 PE=1 SV=1 - [NEUS_HUMAN]	7,56	3	3	57	Extracellular
P20849	Collagen alpha-1(IX) chain OS=Homo sapiens GN=COL9A1 PE=1 SV=3 - [CO9A1_HUMAN]	1,52	1	1	57	Extracellular
Q14966	Zinc finger protein 638 OS=Homo sapiens GN=ZNF638 PE=1 SV=2 - [ZN638_HUMAN]	0,3	1	1	57	Extracellular
Q96NL6	Sodium channel and clathrin linker 1 OS=Homo sapiens GN=SCLT1 PE=1 SV=2 - [SCLT1_HUMAN]	2,33	2	2	56	Extracellular
P01033	Metalloproteinase inhibitor 1 OS=Homo sapiens GN=TIMP1 PE=1 SV=1 - [TIMP1_HUMAN]	16,91	3	3	55	Extracellular
Q96KN2	Beta-Ala-His dipeptidase OS=Homo sapiens GN=CNDP1 PE=1 SV=4 - [CNDP1_HUMAN]	22,88	9	9	55	Extracellular
P27797	Calreticulin OS=Homo sapiens GN=CALR PE=1 SV=1 - [CALR_HUMAN]	12,23	4	4	54	Extracellular
Q12830	Nucleosome-remodeling factor subunit BPTF OS=Homo sapiens GN=BPTF PE=1 SV=3 - [BPTF_HUI	0,43	1	1	54	Extracellular
Q9NPR2	Semaphorin-4B OS=Homo sapiens GN=SEMA4B PE=1 SV=3 - [SEM4B_HUMAN]	7,81	4	4	54	Transmembrane
P04433	Ig kappa chain V-III region VG (Fragment) OS=Homo sapiens PE=1 SV=1 - [KV309_HUMAN]	7,83	1	1	52	Extracellular
Q9BY67	Cell adhesion molecule 1 OS=Homo sapiens GN=CADM1 PE=1 SV=2 - [CADM1_HUMAN]	17,65	5	5	51	Transmembrane
Q9Y287	Integral Transmembrane protein 2B OS=Homo sapiens GN=ITM2B PE=1 SV=1 - [ITM2B_HUMAN]	3,38	1	1	50	Transmembrane
Q9BXJ4	Complement C1q tumor necrosis factor-related protein 3 OS=Homo sapiens GN=C1QTNF3 PE=1 SV	6,5	2	2	49	Extracellular
Q8NG11	Tetraspanin-14 OS=Homo sapiens GN=TSPAN14 PE=1 SV=1 - [TSN14_HUMAN]	2,96	1	1	49	Transmembrane
P06865	Beta-hexosaminidase subunit alpha OS=Homo sapiens GN=HEXA PE=1 SV=2 - [HEXA_HUMAN]	9,45	5	5	48	Extracellular
P23515	Oligodendrocyte-myelin glycoprotein OS=Homo sapiens GN=OMG PE=1 SV=2 - [OMGP_HUMAN]	18,86	6	6	48	Extracellular
Q13449	Limbic system-associated Transmembrane protein OS=Homo sapiens GN=LSAMP PE=1 SV=2 - [LS/	14,5	4	4	48	Transmembrane
Q86YA3	Protein ZGRF1 OS=Homo sapiens GN=ZGRF1 PE=2 SV=3 - [ZGRF1_HUMAN]	0,33	1	1	48	Transmembrane
P13521	Secretogranin-2 OS=Homo sapiens GN=SCG2 PE=1 SV=2 - [SCG2_HUMAN]	12,64	4	4	47	Extracellular
Q9P121	Neurotrimin OS=Homo sapiens GN=NTM PE=1 SV=1 - [NTRI_HUMAN]	6,69	2	2	46	Extracellular
Q86VD1	MORC family CW-type zinc finger protein 1 OS=Homo sapiens GN=MORC1 PE=2 SV=2 - [MORC1_H	1,83	2	2	46	Extracellular
P04406	Glyceraldehyde-3-phosphate dehydrogenase OS=Homo sapiens GN=GAPDH PE=1 SV=3 - [G3P_HU	22,99	5	5	46	Extracellular
Q92797	Symplekin OS=Homo sapiens GN=SYMPK PE=1 SV=2 - [SYMPK_HUMAN]	3,14	2	3	46	Extracellular
P01871	Ig mu chain C region OS=Homo sapiens GN=IGHM PE=1 SV=3 - [IGHM_HUMAN]	23,45	9	9	45	Extracellular
P01781	Ig heavy chain V-III region GAL OS=Homo sapiens PE=1 SV=1 - [HV320_HUMAN]	23,28	2	4	43	Extracellular
Q92743	Serine protease HTRA1 OS=Homo sapiens GN=HTRA1 PE=1 SV=1 - [HTRA1_HUMAN]	8,96	4	4	42	Extracellular
P16519	Neuroendocrine convertase 2 OS=Homo sapiens GN=PCSK2 PE=2 SV=2 - [NEC2_HUMAN]	3,92	2	2	42	Extracellular
Q96JP9	Cadherin-related family member 1 OS=Homo sapiens GN=CDHR1 PE=1 SV=2 - [CDHR1_HUMAN]	7,57	5	5	42	Transmembrane
Q9BZV3	Interphotoreceptor matrix proteoglycan 2 OS=Homo sapiens GN=IMPG2 PE=1 SV=3 - [IMPG2_HUM/	3,46	2	2	42	Transmembrane
Q96DT5	Dynein heavy chain 11, axonemal OS=Homo sapiens GN=DNAH11 PE=1 SV=4 - [DYH11_HUMAN]	0,91	2	3	41	Extracellular
Q99519	Sialidase-1 OS=Homo sapiens GN=NEU1 PE=1 SV=1 - [NEUR1_HUMAN]	7,23	3	3	41	Transmembrane
O75445	Usherin OS=Homo sapiens GN=USH2A PE=1 SV=3 - [USH2A_HUMAN]	0,21	1	1	40	Transmembrane
Q8TE73	Dynein heavy chain 5, axonemal OS=Homo sapiens GN=DNAH5 PE=1 SV=3 - [DYH5_HUMAN]	0,69	2	3	39	Extracellular
Q99523	Sortilin OS=Homo sapiens GN=SORT1 PE=1 SV=3 - [SORT_HUMAN]	1,56	1	1	39	Transmembrane
Q9BRK5	45 kDa calcium-binding protein OS=Homo sapiens GN=SDF4 PE=1 SV=1 - [CAB45_HUMAN]	25,97	7	7	38	Extracellular
O75503	Ceroid-lipofuscinosis neuronal protein 5 OS=Homo sapiens GN=CLN5 PE=1 SV=2 - [CLN5_HUMAN]	8,66	3	3	38	Transmembrane
A8MX76	Calpain-14 OS=Homo sapiens GN=CAPN14 PE=2 SV=2 - [CAN14_HUMAN]	3,95	1	2	37	Extracellular
Q14679	Tubulin polyglutamylase TTLL4 OS=Homo sapiens GN=TTLL4 PE=1 SV=2 - [TTLL4_HUMAN]	2	2	2	37	Extracellular
P61160	Actin-related protein 2 OS=Homo sapiens GN=ACTR2 PE=1 SV=1 - [ARP2_HUMAN]	5,84	1	2	37	Extracellular
Q96JB1	Dynein heavy chain 8, axonemal OS=Homo sapiens GN=DNAH8 PE=1 SV=2 - [DYH8_HUMAN]	0,62	1	2	37	Extracellular
Q9UP83	Conserved oligomeric Golgi complex subunit 5 OS=Homo sapiens GN=COG5 PE=1 SV=3 - [COG5_H	1,79	1	2	37	Extracellular
P22314	Ubiquitin-like modifier-activating enzyme 1 OS=Homo sapiens GN=UBA1 PE=1 SV=3 - [UBA1_HUM/	4,35	2	2	37	Transmembrane
Q9H668	CST complex subunit STN1 OS=Homo sapiens GN=OBFC1 PE=1 SV=2 - [STN1_HUMAN]	3,8	1	1	36	Extracellular
P22792	Carboxypeptidase N subunit 2 OS=Homo sapiens GN=CPN2 PE=1 SV=3 - [CPN2_HUMAN]	17,25	5	5	36	Extracellular
Q9HC10	Otoferlin OS=Homo sapiens GN=OTOF PE=1 SV=3 - [OTOF_HUMAN]	1,4	2	2	36	Transmembrane
A6NMB1	Sialic acid-binding Ig-like lectin 16 OS=Homo sapiens GN=SIGLEC16 PE=2 SV=3 - [SIG16_HUMAN]	1,46	1	1	36	Transmembrane
Q9H8L6	Multimerin-2 OS=Homo sapiens GN=MMRN2 PE=1 SV=2 - [MMRN2_HUMAN]	3,58	2	2	35	Extracellular
P05408	Neuroendocrine protein 7B2 OS=Homo sapiens GN=SCG5 PE=1 SV=2 - [7B2_HUMAN]	10,85	2	2	34	Extracellular
Q9UGM5	Fetuin-B OS=Homo sapiens GN=FETUB PE=1 SV=2 - [FETUB_HUMAN]	7,33	2	2	34	Extracellular
P04278	Sex hormone-binding globulin OS=Homo sapiens GN=SHBG PE=1 SV=2 - [SHBG_HUMAN]	12,94	3	3	33	Extracellular
P61626	Lysozyme C OS=Homo sapiens GN=LYZ PE=1 SV=1 - [LYSC_HUMAN]	32,43	3	3	33	Extracellular
P43320	Beta-crystallin B2 OS=Homo sapiens GN=CRYBB2 PE=1 SV=2 - [CRBB2_HUMAN]	58,54	10	10	33	Extracellular
P48058	Glutamate receptor 4 OS=Homo sapiens GN=GRIA4 PE=2 SV=2 - [GRIA4_HUMAN]	3,88	3	3	33	Transmembrane
Q99572	P2X purinoceptor 7 OS=Homo sapiens GN=P2RX7 PE=1 SV=4 - [P2RX7_HUMAN]	1,01	1	1	33	Transmembrane
O95445	Apolipoprotein M OS=Homo sapiens GN=APOM PE=1 SV=2 - [APOM_HUMAN]	15,43	3	3	32	Extracellular
Q5T6C5	Ataxin-7-like protein 2 OS=Homo sapiens GN=ATXN7L2 PE=3 SV=1 - [AT7L2_HUMAN]	1,11	1	1	32	Extracellular
Q9UHL4	Dipeptidyl peptidase 2 OS=Homo sapiens GN=DPP7 PE=1 SV=3 - [DPP2_HUMAN]	12,4	5	5	32	Extracellular
Q8IZT6	Abnormal spindle-like microcephaly-associated protein OS=Homo sapiens GN=ASPM PE=1 SV=2 - [/	0,66	1	2	32	Extracellular
Q15818	Neuronal pentraxin-1 OS=Homo sapiens GN=NPTX1 PE=2 SV=2 - [NPTX1_HUMAN]	3,7	1	1	32	Extracellular
A6NFK2	Glutaredoxin domain-containing cysteine-rich protein 2 OS=Homo sapiens GN=GRXCR2 PE=3 SV=1	6,45	1	1	32	Extracellular
P07686	Beta-hexosaminidase subunit beta OS=Homo sapiens GN=HEXB PE=1 SV=3 - [HEXB_HUMAN]	6,47	3	3	32	Extracellular
Q9BQT9	Calsyntenin-3 OS=Homo sapiens GN=CLSTN3 PE=1 SV=1 - [CSTN3_HUMAN]	1,88	1	1	32	Transmembrane
Q8NE71	ATP-binding cassette sub-family F member 1 OS=Homo sapiens GN=ABCF1 PE=1 SV=2 - [ABCF1_I	1,3	1	1	31	Extracellular
P01764	Ig heavy chain V-III region 23 OS=Homo sapiens GN=IGHV3-23 PE=1 SV=2 - [HV303_HUMAN]	15,38	1	3	31	Extracellular
O75093	Slit homolog 1 protein OS=Homo sapiens GN=SLIT1 PE=2 SV=4 - [SLIT1_HUMAN]	1,56	2	2	31	Extracellular
P01344	Insulin-like growth factor II OS=Homo sapiens GN=IGF2 PE=1 SV=1 - [IGF2_HUMAN]	13,89	2	2	31	Extracellular
Q8WVM8	Sec1 family domain-containing protein 1 OS=Homo sapiens GN=SCFD1 PE=1 SV=4 - [SCFD1_HUM.	1,71	1	1	31	Extracellular
Q8WZ42	Titin OS=Homo sapiens GN=TTN PE=1 SV=4 - [TITIN_HUMAN]	0,31	7	9	30	Extracellular
P17050	Alpha-N-acetylgalactosaminidase OS=Homo sapiens GN=NAGA PE=1 SV=2 - [NAGAB_HUMAN]	7,79	3	3	29	Extracellular
Q9UEF7	Klotho OS=Homo sapiens GN=KL PE=1 SV=2 - [KLOT_HUMAN]	0,89	1	1	29	Transmembrane
P32245	Melanocortin receptor 4 OS=Homo sapiens GN=MC4R PE=1 SV=2 - [MC4R_HUMAN]	6,33	1	1	28	Transmembrane
P49908	Selenoprotein P OS=Homo sapiens GN=SEPP1 PE=1 SV=3 - [SEPP1_HUMAN]	8,4	2	2	27	Extracellular
Q14055	Collagen alpha-2(IX) chain OS=Homo sapiens GN=COL9A2 PE=1 SV=2 - [CO9A2_HUMAN]	3,92	2	2	27	Extracellular
Q16513	Serine/threonine-protein kinase N2 OS=Homo sapiens GN=PKN2 PE=1 SV=1 - [PKN2_HUMAN]	3,25	2	2	27	Extracellular
Q14520	Hyaluronan-binding protein 2 OS=Homo sapiens GN=HABP2 PE=1 SV=1 - [HABP2_HUMAN]	8,57	5	5	27	Extracellular
Q86VR8	Four-jointed box protein 1 OS=Homo sapiens GN=FJX1 PE=2 SV=1 - [FJX1_HUMAN]	1,6	1	2	27	Extracellular
P04208	Ig lambda chain V-I region WAH OS=Homo sapiens PE=1 SV=1 - [LV106_HUMAN]	16,51	2	2	27	not_matched
O75882	Attractin OS=Homo sapiens GN=ATRN PE=1 SV=2 - [ATRN_HUMAN]	3,57	4	4	27	Transmembrane
O75674	TOM1-like protein 1 OS=Homo sapiens GN=TOM1L1 PE=1 SV=2 - [TM1L1_HUMAN]	2,1	1	1	26	Extracellular
P27216	Annexin A13 OS=Homo sapiens GN=ANXA13 PE=1 SV=3 - [ANX13_HUMAN]	4,43	1	1	26	Extracellular
P80748	Ig lambda chain V-III region LOI OS=Homo sapiens PE=1 SV=1 - [LV302_HUMAN]	7,21	1	1	26	Extracellular
Q96Q04	Serine/threonine-protein kinase LMTK3 OS=Homo sapiens GN=LMTK3 PE=1 SV=2 - [LMTK3_HUMA	0,75	1	1	26	Transmembrane
Q86Y38	Xylosyltransferase 1 OS=Homo sapiens GN=XYLT1 PE=1 SV=1 - [XYLT1_HUMAN]	1,98	1	1	25	Extracellular
Q9H3G5	Probable serine carboxypeptidase CPVL OS=Homo sapiens GN=CPVL PE=1 SV=2 - [CPVL_HUMAN	2,1	1	1	25	Extracellular
Q9NS87	Kinesin-like protein KIF15 OS=Homo sapiens GN=KIF15 PE=1 SV=1 - [KIF15_HUMAN]	0,86	1	1	25	Extracellular
Q86VW0	SEC14 domain and spectrin repeat-containing protein 1 OS=Homo sapiens GN=SESTD1 PE=1 SV=2	2,44	1	2	25	Extracellular
P22692	Insulin-like growth factor-binding protein 4 OS=Homo sapiens GN=IGFBP4 PE=1 SV=2 - [IBP4_HUM/	9,69	2	2	25	Extracellular
P51812	Ribosomal protein S6 kinase alpha-3 OS=Homo sapiens GN=RPS6KA3 PE=1 SV=1 - [KS6A3_HUMA	1,22	1	1	25	Extracellular
P41271	Neuroblastoma suppressor of tumorigenicity 1 OS=Homo sapiens GN=NBL1 PE=1 SV=2 - [NBL1_HU	3,87	1	1	24	Extracellular
P09104	Gamma-enolase OS=Homo sapiens GN=ENO2 PE=1 SV=3 - [ENOG_HUMAN]	5,76	2	2	24	Extracellular
O75145	Liprin-alpha-3 OS=Homo sapiens GN=PPFIA3 PE=1 SV=3 - [LIPA3_HUMAN]	3,43	2	3	24	Extracellular
Q9GZM5	Protein YIPF3 OS=Homo sapiens GN=YIPF3 PE=1 SV=1 - [YIPF3_HUMAN]	2,86	1	1	23	Transmembrane
P23471	Receptor-type tyrosine-protein phosphatase zeta OS=Homo sapiens GN=PTPRZ1 PE=1 SV=4 - [PTP	1,51	3	3	23	Transmembrane
Q5KU26	Collectin-12 OS=Homo sapiens GN=COLEC12 PE=1 SV=3 - [COL12_HUMAN]	2,43	2	2	23	Transmembrane
Q9BYJ0	Fibroblast growth factor-binding protein 2 OS=Homo sapiens GN=FGFBP2 PE=1 SV=1 - [FGFP2_HU	11,66	2	2	22	Extracellular
Q9UBX1	Cathepsin F OS=Homo sapiens GN=CTSF PE=1 SV=1 - [CATF_HUMAN]	8,47	4	4	22	Extracellular
Q7Z7G8	Vacuolar protein sorting-associated protein 13B OS=Homo sapiens GN=VPS13B PE=1 SV=2 - [VP13	0,37	1	1	22	Extracellular
P22914	Beta-crystallin S OS=Homo sapiens GN=CRYGS PE=1 SV=4 - [CRBS_HUMAN]	16,29	4	4	22	Extracellular
Q13510	Acid ceramidase OS=Homo sapiens GN=ASAH1 PE=1 SV=5 - [ASAH1_HUMAN]	5,57	2	2	22	Extracellular
P10253	Lysosomal alpha-glucosidase OS=Homo sapiens GN=GAA PE=1 SV=4 - [LYAG_HUMAN]	3,15	2	2	22	Transmembrane
O00115	Deoxyribonuclease-2-alpha OS=Homo sapiens GN=DNASE2 PE=1 SV=2 - [DNS2A_HUMAN]	3,33	1	1	21	Extracellular
O43790	Keratin, type II cuticular Hb6 OS=Homo sapiens GN=KRT86 PE=1 SV=1 - [KRT86_HUMAN]	1,44	1	1	21	Extracellular
Q5VT06	Centrosome-associated protein 350 OS=Homo sapiens GN=CEP350 PE=1 SV=1 - [CE350_HUMAN]	0,58	1	2	21	Extracellular
Q9P2S2	Neurexin-2 OS=Homo sapiens GN=NRXN2 PE=2 SV=1 - [NRX2A_HUMAN]	1,29	1	2	21	Transmembrane
Q4KMQ2	Anoctamin-6 OS=Homo sapiens GN=ANO6 PE=1 SV=2 - [ANO6_HUMAN]	1,54	2	2	21	Transmembrane
O95967	EGF-containing fibulin-like extracellular matrix protein 2 OS=Homo sapiens GN=EFEMP2 PE=1 SV=3	4,51	2	2	20	Extracellular
Q13275	Semaphorin-3F OS=Homo sapiens GN=SEMA3F PE=2 SV=2 - [SEM3F_HUMAN]	5,48	3	3	20	Extracellular

P24593	Insulin-like growth factor-binding protein 5 OS=Homo sapiens GN=IGFBP5 PE=1 SV=1 - [IBP5_HUM	5,15	1	1	20	Extracellular
P24821	Tenascin OS=Homo sapiens GN=TNC PE=1 SV=3 - [TENA_HUMAN]	1,68	4	4	20	Extracellular
P00338	L-lactate dehydrogenase A chain OS=Homo sapiens GN=LDHA PE=1 SV=2 - [LDHA_HUMAN]	3,61	1	1	20	Extracellular
Q99714	3-hydroxyacyl-CoA dehydrogenase type-2 OS=Homo sapiens GN=HSD17B10 PE=1 SV=3 - [HCD2_H	4,6	1	1	19	Extracellular
P12259	Coagulation factor V OS=Homo sapiens GN=F5 PE=1 SV=4 - [FA5_HUMAN]	1,84	3	3	19	Extracellular
Q96FE7	Phosphoinositide-3-kinase-interacting protein 1 OS=Homo sapiens GN=PIK3IP1 PE=1 SV=2 - [P3IP1	9,89	2	2	19	Transmembrane
O76074	cGMP-specific 3',5'-cyclic phosphodiesterase OS=Homo sapiens GN=PDE5A PE=1 SV=2 - [PDE5A_	0,57	1	1	18	Extracellular
P21246	Pleiotrophin OS=Homo sapiens GN=PTN PE=1 SV=1 - [PTN_HUMAN]	14,29	1	1	18	Extracellular
Q86SQ0	Pleckstrin homology-like domain family B member 2 OS=Homo sapiens GN=PHLDB2 PE=1 SV=2 - [F	1,68	2	2	18	Extracellular
O00566	U3 small nucleolar ribonucleoprotein protein MPP10 OS=Homo sapiens GN=MPHOSPH10 PE=1 SV=	1,91	1	1	18	Extracellular
Q9UM21	Alpha-1,3-mannosyl-glycoprotein 4-beta-N-acetylglucosaminyltransferase A OS=Homo sapiens GN=M	3,55	1	1	18	Extracellular
P04434	Ig kappa chain V-III region VH (Fragment) OS=Homo sapiens PE=4 SV=1 - [KV310_HUMAN]	23,28	2	2	18	not_matched
Q15846	Clusterin-like protein 1 OS=Homo sapiens GN=CLUL1 PE=2 SV=1 - [CLUL1_HUMAN]	9,01	2	2	17	Extracellular
P02100	Hemoglobin subunit epsilon OS=Homo sapiens GN=HBE1 PE=1 SV=2 - [HBE_HUMAN]	14,29	1	2	17	Extracellular
P48723	Heat shock 70 kDa protein 13 OS=Homo sapiens GN=HSPA13 PE=1 SV=1 - [HSP13_HUMAN]	8,28	3	3	17	Extracellular
P0C7P3	Schlafen family member 14 OS=Homo sapiens GN=SLFN14 PE=2 SV=2 - [SLN14_HUMAN]	2,41	2	2	17	Extracellular
Q9HC77	Centromere protein J OS=Homo sapiens GN=CENPJ PE=1 SV=2 - [CENPJ_HUMAN]	0,9	1	1	17	Extracellular
O43306	Adenylate cyclase type 6 OS=Homo sapiens GN=ADCY6 PE=1 SV=2 - [ADCY6_HUMAN]	0,68	1	1	17	Transmembrane
Q86UK0	ATP-binding cassette sub-family A member 12 OS=Homo sapiens GN=ABCA12 PE=1 SV=3 - [ABCA	0,35	1	1	17	Transmembrane
O60733	85/88 kDa calcium-independent phospholipase A2 OS=Homo sapiens GN=PLA2G6 PE=1 SV=2 - [PL	0,87	1	1	17	Transmembrane
P30291	Wee1-like protein kinase OS=Homo sapiens GN=WEE1 PE=1 SV=2 - [WEE1_HUMAN]	1,86	1	1	16	Extracellular
Q8TAG5	V-set and Transmembrane domain-containing protein 2A OS=Homo sapiens GN=VSTM2A PE=2 SV=	5,51	1	1	16	Extracellular
P02746	Complement C1q subcomponent subunit B OS=Homo sapiens GN=C1QB PE=1 SV=3 - [C1QB_HUM	11,86	4	4	16	Extracellular
Q92563	Testican-2 OS=Homo sapiens GN=SPOCK2 PE=1 SV=1 - [TICN2_HUMAN]	9,67	3	3	16	Extracellular
Q9Y5K1	Meiotic recombination protein SPO11 OS=Homo sapiens GN=SPO11 PE=2 SV=1 - [SPO11_HUMAN	4,29	1	1	16	Extracellular
Q9GZX5	Zinc finger protein 350 OS=Homo sapiens GN=ZNF350 PE=1 SV=3 - [ZN350_HUMAN]	1,69	1	1	16	Extracellular
O14791	Apolipoprotein L1 OS=Homo sapiens GN=APOL1 PE=1 SV=5 - [APOL1_HUMAN]	7,54	2	2	15	Extracellular
P57771	Regulator of G-protein signaling 8 OS=Homo sapiens GN=RGS8 PE=1 SV=1 - [RGS8_HUMAN]	11,67	1	1	15	Extracellular
A8MV23	Serpin E3 OS=Homo sapiens GN=SERPINE3 PE=2 SV=2 - [SERP3_HUMAN]	5,19	3	3	15	Extracellular
P03950	Angiogenin OS=Homo sapiens GN=ANG PE=1 SV=1 - [ANGI_HUMAN]	18,37	2	2	15	Extracellular
Q9HCJ0	Trinucleotide repeat-containing gene 6C protein OS=Homo sapiens GN=TNRC6C PE=1 SV=3 - [TNR	0,47	1	1	15	Extracellular
P49746	Thrombospondin-3 OS=Homo sapiens GN=THBS3 PE=1 SV=1 - [TSP3_HUMAN]	1,88	1	1	15	Extracellular
Q9NX58	Cell growth-regulating nucleolar protein OS=Homo sapiens GN=LYAR PE=1 SV=2 - [LYAR_HUMAN]	1,58	1	1	15	Extracellular
P17643	5,6-dihydroxyindole-2-carboxylic acid oxidase OS=Homo sapiens GN=TYRP1 PE=1 SV=2 - [TYRP1_	3,35	1	1	15	Transmembrane
P22680	Cholesterol 7-alpha-monooxygenase OS=Homo sapiens GN=CYP7A1 PE=1 SV=2 - [CP7A1_HUMAN	4,76	1	1	15	Transmembrane
P40189	Interleukin-6 receptor subunit beta OS=Homo sapiens GN=IL6ST PE=1 SV=2 - [IL6RB_HUMAN]	5,01	3	3	15	Transmembrane
P42356	Phosphatidylinositol 4-kinase alpha OS=Homo sapiens GN=PI4KA PE=1 SV=4 - [PI4KA_HUMAN]	0,43	1	1	15	Transmembrane
Q8TCU4	Alstrom syndrome protein 1 OS=Homo sapiens GN=ALMS1 PE=1 SV=3 - [ALMS1_HUMAN]	0,5	2	2	14	Extracellular
P17900	Ganglioside GM2 activator OS=Homo sapiens GN=GM2A PE=1 SV=4 - [SAP3_HUMAN]	17,1	2	2	14	Extracellular
Q06033	Inter-alpha-trypsin inhibitor heavy chain H3 OS=Homo sapiens GN=ITIH3 PE=1 SV=2 - [ITIH3_HUMA	1,69	1	1	14	Extracellular
Q9P2P6	StAR-related lipid transfer protein 9 OS=Homo sapiens GN=STARD9 PE=1 SV=3 - [STAR9_HUMAN]	0,36	1	1	14	Extracellular
Q7Z2W4	Zinc finger CCCH-type antiviral protein 1 OS=Homo sapiens GN=ZC3HAV1 PE=1 SV=3 - [ZCCHV_H	1,77	1	1	14	Extracellular
O95714	E3 ubiquitin-protein ligase HERC2 OS=Homo sapiens GN=HERC2 PE=1 SV=2 - [HERC2_HUMAN]	0,74	2	2	14	Extracellular
Q96KG7	Multiple epidermal growth factor-like domains protein 10 OS=Homo sapiens GN=MEGF10 PE=1 SV=1	1,32	1	1	14	Transmembrane
O94856	Neurofascin OS=Homo sapiens GN=NFASC PE=1 SV=4 - [NFASC_HUMAN]	2,6	3	3	14	Transmembrane
O60888	Protein CutA OS=Homo sapiens GN=CUTA PE=1 SV=2 - [CUTA_HUMAN]	10,06	1	1	14	Transmembrane
Q9Y646	Carboxypeptidase Q OS=Homo sapiens GN=CPQ PE=1 SV=1 - [CBPQ_HUMAN]	1,91	1	1	13	Extracellular
P00740	Coagulation factor IX OS=Homo sapiens GN=F9 PE=1 SV=2 - [FA9_HUMAN]	7,38	3	3	13	Extracellular
O75150	E3 ubiquitin-protein ligase BRE1B OS=Homo sapiens GN=RNF40 PE=1 SV=4 - [BRE1B_HUMAN]	1,1	1	1	13	Extracellular
P08493	Matrix Gla protein OS=Homo sapiens GN=MGP PE=1 SV=2 - [MGP_HUMAN]	10,68	1	1	13	Extracellular
P48681	Nestin OS=Homo sapiens GN=NES PE=1 SV=2 - [NEST_HUMAN]	1,05	1	1	13	Extracellular
Q96L92	Sorting nexin-27 OS=Homo sapiens GN=SNX27 PE=1 SV=2 - [SNX27_HUMAN]	3,88	1	1	13	Extracellular
Q8NCT3	Uncharacterized protein KIAA0895 OS=Homo sapiens GN=KIAA0895 PE=2 SV=4 - [K0895_HUMAN]	3,27	1	1	13	Extracellular
Q9Y4I1	Unconventional myosin-Va OS=Homo sapiens GN=MYO5A PE=1 SV=2 - [MYO5A_HUMAN]	0,59	1	1	13	Extracellular
P16070	CD44 antigen OS=Homo sapiens GN=CD44 PE=1 SV=3 - [CD44_HUMAN]	2,83	2	2	13	Transmembrane
P33908	Mannosyl-oligosaccharide 1,2-alpha-mannosidase IA OS=Homo sapiens GN=MAN1A1 PE=1 SV=3 -	4,9	3	3	13	Transmembrane
Q6NUM9	All-trans-retinol 13,14-reductase OS=Homo sapiens GN=RETSAT PE=1 SV=2 - [RETST_HUMAN]	1,97	1	1	12	Extracellular
P35222	Catenin beta-1 OS=Homo sapiens GN=CTNNB1 PE=1 SV=1 - [CTNB1_HUMAN]	0,9	1	1	12	Extracellular
Q0P6D6	Coiled-coil domain-containing protein 15 OS=Homo sapiens GN=CCDC15 PE=2 SV=2 - [CCD15_HU	5,57	3	3	12	Extracellular
Q5T9S5	Coiled-coil domain-containing protein 18 OS=Homo sapiens GN=CCDC18 PE=2 SV=1 - [CCD18_HU	2,34	2	2	12	Extracellular
Q9Y2U5	Mitogen-activated protein kinase kinase kinase 2 OS=Homo sapiens GN=MAP3K2 PE=1 SV=2 - [M3K	2,42	1	2	12	Extracellular
Q99784	Noelin OS=Homo sapiens GN=OLFM1 PE=1 SV=4 - [NOE1_HUMAN]	5,15	2	3	12	Extracellular
Q63HQ2	Pikachurin OS=Homo sapiens GN=EGFLAM PE=1 SV=2 - [EGFLA_HUMAN]	1,28	1	1	12	Extracellular
Q9HCI7	E3 ubiquitin-protein ligase MSL2 OS=Homo sapiens GN=MSL2 PE=1 SV=2 - [MSL2_HUMAN]	1,56	1	1	12	Extracellular
P01625	Ig kappa chain V-IV region Len OS=Homo sapiens PE=1 SV=2 - [KV402_HUMAN]	13,16	1	1	12	not_matched
Q12797	Aspartyl/asparaginyl beta-hydroxylase OS=Homo sapiens GN=ASPH PE=1 SV=3 - [ASPH_HUMAN]	2,37	1	1	12	Transmembrane
Q86WI1	Fibrocystin-L OS=Homo sapiens GN=PKHD1L1 PE=2 SV=2 - [PKHL1_HUMAN]	0,52	2	2	12	Transmembrane
O00461	Golgi integral Transmembrane protein 4 OS=Homo sapiens GN=GOLIM4 PE=1 SV=1 - [GOLI4_HUMA	1,87	1	1	12	Transmembrane
P84098	60S ribosomal protein L19 OS=Homo sapiens GN=RPL19 PE=1 SV=1 - [RL19_HUMAN]	5,1	1	1	11	Cytoplasm
P53004	Biliverdin reductase A OS=Homo sapiens GN=BLVRA PE=1 SV=2 - [BIEA_HUMAN]	5,41	1	1	11	Extracellular
P07711	Cathepsin L1 OS=Homo sapiens GN=CTSL PE=1 SV=2 - [CATL1_HUMAN]	4,2	1	1	11	Extracellular
P0C7V8	DDB1- and CUL4-associated factor 8-like protein 2 OS=Homo sapiens GN=DCAF8L2 PE=2 SV=2 - [D	3,96	2	2	11	Extracellular
Q9HC35	Echinoderm microtubule-associated protein-like 4 OS=Homo sapiens GN=EML4 PE=1 SV=3 - [EMAL	2,14	1	1	11	Extracellular
P38571	Lysosomal acid lipase/cholesteryl ester hydrolase OS=Homo sapiens GN=LIPA PE=1 SV=2 - [LICH_H	2,76	1	1	11	Extracellular
O60287	Nucleolar pre-ribosomal-associated protein 1 OS=Homo sapiens GN=URB1 PE=1 SV=4 - [NPA1P_HU	0,4	1	1	11	Extracellular
P51888	Prolargin OS=Homo sapiens GN=PRELP PE=1 SV=1 - [PRELP_HUMAN]	6,54	2	2	11	Extracellular
Q6PKX4	Docking protein 6 OS=Homo sapiens GN=DOK6 PE=1 SV=1 - [DOK6_HUMAN]	1,81	1	1	11	Extracellular
Q96KQ4	Apoptosis-stimulating of p53 protein 1 OS=Homo sapiens GN=PPP1R13B PE=1 SV=3 - [ASPP1_HU	1,65	1	1	11	Extracellular
P55061	Bax inhibitor 1 OS=Homo sapiens GN=TMBIM6 PE=1 SV=2 - [BI1_HUMAN]	3,38	1	1	11	Transmembrane
P19021	Peptidyl-glycine alpha-amidating monooxygenase OS=Homo sapiens GN=PAM PE=1 SV=2 - [AMD_H	3,19	2	2	11	Transmembrane
P25116	Proteinase-activated receptor 1 OS=Homo sapiens GN=F2R PE=1 SV=2 - [PAR1_HUMAN]	3,29	1	1	11	Transmembrane
P23467	Receptor-type tyrosine-protein phosphatase beta OS=Homo sapiens GN=PTPRB PE=1 SV=3 - [PTPF	0,35	1	1	11	Transmembrane
Q9BYH1	Seizure 6-like protein OS=Homo sapiens GN=SEZ6L PE=1 SV=1 - [SE6L1_HUMAN]	1,27	2	2	11	Transmembrane
Q15131	Cyclin-dependent kinase 10 OS=Homo sapiens GN=CDK10 PE=1 SV=1 - [CDK10_HUMAN]	5,83	2	2	10	Extracellular
Q8IWU5	Extracellular sulfatase Sulf-2 OS=Homo sapiens GN=SULF2 PE=1 SV=1 - [SULF2_HUMAN]	1,49	1	1	10	Extracellular
P05154	Plasma serine protease inhibitor OS=Homo sapiens GN=SERPINA5 PE=1 SV=3 - [IPSP_HUMAN]	2,96	1	1	10	Extracellular
Q8NCL4	Polypeptide N-acetylgalactosaminyltransferase 6 OS=Homo sapiens GN=GALNT6 PE=2 SV=2 - [GAL	2,57	1	1	10	Extracellular
P49788	Retinoic acid receptor responder protein 1 OS=Homo sapiens GN=RARRES1 PE=1 SV=2 - [TIG1_HU	4,08	1	1	10	Extracellular
Q9NRR4	Ribonuclease 3 OS=Homo sapiens GN=DROSHA PE=1 SV=2 - [RNC_HUMAN]	1,53	1	1	10	Extracellular
Q15582	Transforming growth factor-beta-induced protein ig-h3 OS=Homo sapiens GN=TGFBI PE=1 SV=1 - [E	5,71	3	3	10	Extracellular
P82094	TATA element modulatory factor OS=Homo sapiens GN=TMF1 PE=1 SV=2 - [TMF1_HUMAN]	2,56	2	2	10	Extracellular
Q8IZW8	Tensin-4 OS=Homo sapiens GN=TNS4 PE=1 SV=3 - [TENS4_HUMAN]	1,4	1	1	10	Extracellular
Q92859	Neogenin OS=Homo sapiens GN=NEO1 PE=1 SV=2 - [NEO1_HUMAN]	2,26	2	2	10	Transmembrane
P18428	Lipopolysaccharide-binding protein OS=Homo sapiens GN=LBP PE=1 SV=3 - [LBP_HUMAN]	2,29	1	1	9	Extracellular
Q96LB9	Peptidoglycan recognition protein 3 OS=Homo sapiens GN=PGLYRP3 PE=1 SV=1 - [PGRP3_HUMAI	4,69	2	2	9	Extracellular
Q9H707	Zinc finger protein 552 OS=Homo sapiens GN=ZNF552 PE=1 SV=2 - [ZN552_HUMAN]	5,16	1	1	9	Extracellular
P62304	Small nuclear ribonucleoprotein E OS=Homo sapiens GN=SNRPE PE=1 SV=1 - [RUXE_HUMAN]	27,17	1	1	9	Extracellular
Q14527	Helicase-like transcription factor OS=Homo sapiens GN=HLTF PE=1 SV=2 - [HLTF_HUMAN]	1,98	1	1	9	Extracellular
Q86WI3	Protein NLRC5 OS=Homo sapiens GN=NLRC5 PE=1 SV=3 - [NLRC5_HUMAN]	0,48	1	1	9	Extracellular
Q96SN8	CDK5 regulatory subunit-associated protein 2 OS=Homo sapiens GN=CDK5RAP2 PE=1 SV=5 - [CK5	1,27	2	2	9	Extracellular
Q9ULG1	DNA helicase INO80 OS=Homo sapiens GN=INO80 PE=1 SV=2 - [INO80_HUMAN]	0,96	1	1	9	Extracellular
Q8N3J6	Cell adhesion molecule 2 OS=Homo sapiens GN=CADM2 PE=2 SV=1 - [CADM2_HUMAN]	5,06	2	2	9	Transmembrane
O15031	Plexin-B2 OS=Homo sapiens GN=PLXNB2 PE=1 SV=3 - [PLXB2_HUMAN]	3,05	4	4	9	Transmembrane
Q24JP5	Transmembrane protein 132A OS=Homo sapiens GN=TMEM132A PE=1 SV=1 - [T132A_HUMAN]	6,26	4	4	9	Transmembrane
Q13367	AP-3 complex subunit beta-2 OS=Homo sapiens GN=AP3B2 PE=1 SV=2 - [AP3B2_HUMAN]	1,29	1	1	8	Extracellular
P02655	Apolipoprotein C-II OS=Homo sapiens GN=APOC2 PE=1 SV=1 - [APOC2_HUMAN]	8,91	1	1	8	Extracellular
Q96JB2	Conserved oligomeric Golgi complex subunit 3 OS=Homo sapiens GN=COG3 PE=1 SV=3 - [COG3_H	0,72	1	1	8	Extracellular
Q9C0G6	Dynein heavy chain 6, axonemal OS=Homo sapiens GN=DNAH6 PE=2 SV=3 - [DYH6_HUMAN]	0,55	1	1	8	Extracellular
Q03001	Dystonin OS=Homo sapiens GN=DST PE=1 SV=4 - [DYST_HUMAN]	0,09	1	1	8	Extracellular
P24043	Laminin subunit alpha-2 OS=Homo sapiens GN=LAMA2 PE=1 SV=4 - [LAMA2_HUMAN]	0,77	2	2	8	Extracellular
Q494U1	Pleckstrin homology domain-containing family N member 1 OS=Homo sapiens GN=PLEKHN1 PE=1 S	1,81	1	1	8	Extracellular
Q15185	Prostaglandin E synthase 3 OS=Homo sapiens GN=PTGES3 PE=1 SV=1 - [TEBP_HUMAN]	8,13	1	1	8	Extracellular
Q9H792	Pseudopodium-enriched atypical kinase 1 OS=Homo sapiens GN=PEAK1 PE=1 SV=4 - [PEAK1_HU	1,32	1	2	8	Extracellular
Q13214	Semaphorin-3B OS=Homo sapiens GN=SEMA3B PE=2 SV=1 - [SEM3B_HUMAN]	5,47	3	3	8	Extracellular
Q15911	Zinc finger homeobox protein 3 OS=Homo sapiens GN=ZFHX3 PE=1 SV=2 - [ZFHX3_HUMAN]	0,7	2	2	8	Extracellular

Q86WZ6	Zinc finger protein 227 OS=Homo sapiens GN=ZNF227 PE=1 SV=1 - [ZN227_HUMAN]	1,63	1	1	8	Extracellular
Q15293	Reticulocalbin-1 OS=Homo sapiens GN=RCN1 PE=1 SV=1 - [RCN1_HUMAN]	2,42	1	1	8	Extracellular
Q68DN1	Uncharacterized protein C2orf16 OS=Homo sapiens GN=C2orf16 PE=2 SV=3 - [CB016_HUMAN]	0,71	1	1	8	Extracellular
Q8TET4	Neutral alpha-glucosidase C OS=Homo sapiens GN=GANC PE=2 SV=3 - [GANC_HUMAN]	0,77	1	1	8	Extracellular
Q96L93	Kinesin-like protein KIF16B OS=Homo sapiens GN=KIF16B PE=1 SV=2 - [KI16B_HUMAN]	1,06	2	2	8	Extracellular
Q9H4D0	Calsyntenin-2 OS=Homo sapiens GN=CLSTN2 PE=1 SV=2 - [CSTN2_HUMAN]	1,68	1	1	8	Transmembrane
P26992	Ciliary neurotrophic factor receptor subunit alpha OS=Homo sapiens GN=CNTFR PE=1 SV=2 - [CNTF	3,49	1	1	8	Transmembrane
Q9P225	Dynein heavy chain 2, axonemal OS=Homo sapiens GN=DNAH2 PE=2 SV=3 - [DYH2_HUMAN]	0,54	2	3	8	Transmembrane
Q92621	Nuclear pore complex protein Nup205 OS=Homo sapiens GN=NUP205 PE=1 SV=3 - [NU205_HUMA	1,29	2	2	8	Transmembrane
O75752	UDP-GalNAc:beta-1,3-N-acetylgalactosaminyltransferase 1 OS=Homo sapiens GN=B3GALNT1 PE=2	4,83	1	1	8	Transmembrane
Q12907	Vesicular integral-Transmembrane protein VIP36 OS=Homo sapiens GN=LMAN2 PE=1 SV=1 - [LMAI	3,09	1	1	8	Transmembrane
Q12912	Lymphoid-restricted Transmembrane protein OS=Homo sapiens GN=LRMP PE=1 SV=3 - [LRMP_HU	1,44	1	1	8	Transmembrane
Q9UBP0	Spastin OS=Homo sapiens GN=SPAST PE=1 SV=1 - [SPAST_HUMAN]	2,44	1	1	8	Transmembrane
P46108	Adapter molecule crk OS=Homo sapiens GN=CRK PE=1 SV=2 - [CRK_HUMAN]	3,95	1	1	7	Extracellular
Q9Y574	Ankyrin repeat and SOCS box protein 4 OS=Homo sapiens GN=ASB4 PE=2 SV=1 - [ASB4_HUMAN]	3,05	1	1	7	Extracellular
P58107	Epiplakin OS=Homo sapiens GN=EPPK1 PE=1 SV=2 - [EPIPL_HUMAN]	1,24	1	1	7	Extracellular
Q659C4	La-related protein 1B OS=Homo sapiens GN=LARP1B PE=1 SV=2 - [LAR1B_HUMAN]	1,31	1	1	7	Extracellular
Q96NW7	Leucine-rich repeat-containing protein 7 OS=Homo sapiens GN=LRRC7 PE=1 SV=1 - [LRRC7_HUM/	0,52	1	1	7	Extracellular
Q9NU22	Midasin OS=Homo sapiens GN=MDN1 PE=1 SV=2 - [MDN1_HUMAN]	0,63	2	3	7	Extracellular
Q7L0Y3	Mitochondrial ribonuclease P protein 1 OS=Homo sapiens GN=TRMT10C PE=1 SV=2 - [MRRP1_HUI	6,7	2	2	7	Extracellular
Q02817	Mucin-2 OS=Homo sapiens GN=MUC2 PE=1 SV=2 - [MUC2_HUMAN]	0,46	2	2	7	Extracellular
Q9Y4F4	Protein FAM179B OS=Homo sapiens GN=FAM179B PE=1 SV=4 - [F179B_HUMAN]	1,34	2	2	7	Extracellular
Q5SRH9	Tetratricopeptide repeat protein 39A OS=Homo sapiens GN=TTC39A PE=2 SV=1 - [TT39A_HUMAN]	2,77	1	1	7	Extracellular
P40818	Ubiquitin carboxyl-terminal hydrolase 8 OS=Homo sapiens GN=USP8 PE=1 SV=1 - [UBP8_HUMAN]	1,79	1	1	7	Extracellular
O95970	Leucine-rich glioma-inactivated protein 1 OS=Homo sapiens GN=LGI1 PE=1 SV=1 - [LGI1_HUMAN]	1,97	1	1	7	Extracellular
P34931	Heat shock 70 kDa protein 1-like OS=Homo sapiens GN=HSPA1L PE=1 SV=2 - [HS71L_HUMAN]	2,03	1	1	7	Extracellular
Q08AI8	Uncharacterized protein C2orf54 OS=Homo sapiens GN=C2orf54 PE=2 SV=2 - [CB054_HUMAN]	2,01	1	1	7	Extracellular
P15291	Beta-1,4-galactosyltransferase 1 OS=Homo sapiens GN=B4GALT1 PE=1 SV=5 - [B4GT1_HUMAN]	14,07	3	3	7	Transmembrane
P51800	Chloride channel protein ClC-Ka OS=Homo sapiens GN=CLCNKA PE=1 SV=1 - [CLCKA_HUMAN]	2,62	1	1	7	Transmembrane
Q9H1K4	Mitochondrial glutamate carrier 2 OS=Homo sapiens GN=SLC25A18 PE=1 SV=1 - [GHC2_HUMAN]	6,98	1	1	7	Transmembrane
Q13948	Protein CASP OS=Homo sapiens GN=CUX1 PE=1 SV=2 - [CASP_HUMAN]	3,39	1	1	7	Transmembrane
Q9P212	1-phosphatidylinositol 4,5-bisphosphate phosphodiesterase epsilon-1 OS=Homo sapiens GN=PLCE1	1,22	2	2	6	Extracellular
Q9UNX3	60S ribosomal protein L26-like 1 OS=Homo sapiens GN=RPL26L1 PE=1 SV=1 - [RL26L_HUMAN]	6,9	1	1	6	Extracellular
Q9UHI8	A disintegrin and metalloproteinase with thrombospondin motifs 1 OS=Homo sapiens GN=ADAMTS1	5,27	2	2	6	Extracellular
Q14123	Calcium/calmodulin-dependent 3',5'-cyclic nucleotide phosphodiesterase 1C OS=Homo sapiens GN=I	1,55	1	1	6	Extracellular
Q6ZP82	Coiled-coil domain-containing protein 141 OS=Homo sapiens GN=CCDC141 PE=1 SV=2 - [CC141_H	1,59	2	2	6	Extracellular
Q9Y6G9	Cytoplasmic dynein 1 light intermediate chain 1 OS=Homo sapiens GN=DYNC1LI1 PE=1 SV=3 - [DC	4,78	1	1	6	Extracellular
Q9UBZ9	DNA repair protein REV1 OS=Homo sapiens GN=REV1 PE=1 SV=1 - [REV1_HUMAN]	0,8	1	1	6	Extracellular
O14498	Immunoglobulin superfamily containing leucine-rich repeat protein OS=Homo sapiens GN=ISLR PE=2	3,27	1	1	6	Extracellular
O95897	Noelin-2 OS=Homo sapiens GN=OLFM2 PE=1 SV=2 - [NOE2_HUMAN]	2,42	1	1	6	Extracellular
Q9BXG8	Spermatogenic leucine zipper protein 1 OS=Homo sapiens GN=SPZ1 PE=1 SV=2 - [SPZ1_HUMAN]	4,65	1	1	6	Extracellular
Q9H497	Torsin-3A OS=Homo sapiens GN=TOR3A PE=1 SV=1 - [TOR3A_HUMAN]	4,03	1	1	6	Extracellular
Q9UPT9	Ubiquitin carboxyl-terminal hydrolase 22 OS=Homo sapiens GN=USP22 PE=1 SV=2 - [UBP22_HUM/	4	1	1	6	Extracellular
O75962	Triple functional domain protein OS=Homo sapiens GN=TRIO PE=1 SV=2 - [TRIO_HUMAN]	1,19	3	4	6	Extracellular
P12883	Myosin-7 OS=Homo sapiens GN=MYH7 PE=1 SV=5 - [MYH7_HUMAN]	0,72	1	1	6	Extracellular
Q12955	Ankyrin-3 OS=Homo sapiens GN=ANK3 PE=1 SV=3 - [ANK3_HUMAN]	0,5	1	1	6	Extracellular
Q15113	Procollagen C-endopeptidase enhancer 1 OS=Homo sapiens GN=PCOLCE PE=1 SV=2 - [PCOC1_H	9,13	2	2	6	Extracellular
Q8IX29	F-box only protein 16 OS=Homo sapiens GN=FBXO16 PE=2 SV=1 - [FBX16_HUMAN]	2,74	1	1	6	Extracellular
Q8WXA3	RUN and FYVE domain-containing protein 2 OS=Homo sapiens GN=RUFY2 PE=1 SV=2 - [RUFY2_H	1,53	1	1	6	Extracellular
Q96AX9	E3 ubiquitin-protein ligase MIB2 OS=Homo sapiens GN=MIB2 PE=1 SV=3 - [MIB2_HUMAN]	0,59	1	1	6	Extracellular
Q99435	Protein kinase C-binding protein NELL2 OS=Homo sapiens GN=NELL2 PE=1 SV=1 - [NELL2_HUMA	4,78	3	3	6	Extracellular
Q9NQT6	Fascin-3 OS=Homo sapiens GN=FSCN3 PE=2 SV=1 - [FSCN3_HUMAN]	3,21	1	1	6	Extracellular
Q9P217	Zinc finger SWIM domain-containing protein 5 OS=Homo sapiens GN=ZSWIM5 PE=2 SV=2 - [ZSWM	0,68	1	1	6	Extracellular
Q02763	Angiopoietin-1 receptor OS=Homo sapiens GN=TEK PE=1 SV=2 - [TIE2_HUMAN]	0,98	1	1	6	Transmembrane
O15354	Prosaposin receptor GPR37 OS=Homo sapiens GN=GPR37 PE=1 SV=2 - [GPR37_HUMAN]	8,32	3	3	6	Transmembrane
P21333	Filamin-A OS=Homo sapiens GN=FLNA PE=1 SV=4 - [FLNA_HUMAN]	0,83	1	1	6	Transmembrane
Q16134	Electron transfer flavoprotein-ubiquinone oxidoreductase, mitochondrial OS=Homo sapiens GN=ETFI	2,92	1	1	6	Transmembrane
Q8N6H7	ADP-ribosylation factor GTPase-activating protein 2 OS=Homo sapiens GN=ARFGAP2 PE=1 SV=1 -	4,22	1	1	5	Extracellular
Q9UBS4	DnaJ homolog subfamily B member 11 OS=Homo sapiens GN=DNAJB11 PE=1 SV=1 - [DJB11_HUM	3,91	1	1	5	Extracellular
Q53RD9	Fibulin-7 OS=Homo sapiens GN=FBLN7 PE=2 SV=1 - [FBLN7_HUMAN]	5,01	1	1	5	Extracellular
Q13630	GDP-L-fucose synthase OS=Homo sapiens GN=TSTA3 PE=1 SV=1 - [FCL_HUMAN]	4,36	1	1	5	Extracellular
Q9UPS6	Histone-lysine N-methyltransferase SETD1B OS=Homo sapiens GN=SETD1B PE=1 SV=3 - [SET1B	0,66	1	2	5	Extracellular
Q8N3Z0	Inactive serine protease 35 OS=Homo sapiens GN=PRSS35 PE=2 SV=2 - [PRS35_HUMAN]	3,39	1	1	5	Extracellular
Q96Q89	Kinesin-like protein KIF20B OS=Homo sapiens GN=KIF20B PE=1 SV=3 - [KI20B_HUMAN]	0,55	1	1	5	Extracellular
Q9UIC8	Leucine carboxyl methyltransferase 1 OS=Homo sapiens GN=LCMT1 PE=1 SV=2 - [LCMT1_HUMAN	2,69	1	1	5	Extracellular
Q9P2M1	LRP2-binding protein OS=Homo sapiens GN=LRP2BP PE=1 SV=2 - [LR2BP_HUMAN]	5,48	1	1	5	Extracellular
P23368	NAD-dependent malic enzyme, mitochondrial OS=Homo sapiens GN=ME2 PE=1 SV=1 - [MAOM_HU	2,23	1	1	5	Extracellular
Q5VST9	Obscurin OS=Homo sapiens GN=OBSCN PE=1 SV=3 - [OBSCN_HUMAN]	0,23	1	2	5	Extracellular
Q9HB21	Pleckstrin homology domain-containing family A member 1 OS=Homo sapiens GN=PLEKHA1 PE=1 S	1,98	1	1	5	Extracellular
Q96LQ0	Protein phosphatase 1 regulatory subunit 36 OS=Homo sapiens GN=PPP1R36 PE=1 SV=1 - [PPR36	4,03	1	1	5	Extracellular
Q86T23	Putative ciliary rootlet coiled-coil protein-like 1 protein OS=Homo sapiens GN=CROCCP2 PE=5 SV=1	13,51	1	1	5	Extracellular
A8MXQ7	Putative IQ motif and ankyrin repeat domain-containing protein LOC642574 OS=Homo sapiens PE=5	1,99	1	1	5	Extracellular
P02549	Spectrin alpha chain, erythrocytic 1 OS=Homo sapiens GN=SPTA1 PE=1 SV=5 - [SPTA1_HUMAN]	0,5	1	1	5	Extracellular
Q12986	Transcriptional repressor NF-X1 OS=Homo sapiens GN=NFX1 PE=1 SV=2 - [NFX1_HUMAN]	0,63	1	1	5	Extracellular
Q86TN4	tRNA 2'-phosphotransferase 1 OS=Homo sapiens GN=TRPT1 PE=1 SV=2 - [TRPT1_HUMAN]	9,09	1	1	5	Extracellular
P51451	Tyrosine-protein kinase Blk OS=Homo sapiens GN=BLK PE=1 SV=3 - [BLK_HUMAN]	5,54	2	2	5	Extracellular
A8MQT2	Golgin subfamily A member 8B OS=Homo sapiens GN=GOLGA8B PE=2 SV=2 - [GOG8B_HUMAN]	4,15	2	2	5	Extracellular
O15169	Axin-1 OS=Homo sapiens GN=AXIN1 PE=1 SV=2 - [AXIN1_HUMAN]	2,09	1	1	5	Extracellular
Q9NWH7	Spermatogenesis-associated protein 6 OS=Homo sapiens GN=SPATA6 PE=1 SV=1 - [SPAT6_HUM/	4,3	1	1	5	Extracellular
Q9UK59	Lariat debranching enzyme OS=Homo sapiens GN=DBR1 PE=1 SV=2 - [DBR1_HUMAN]	2,76	1	1	5	Extracellular
Q8NF91	Nesprin-1 OS=Homo sapiens GN=SYNE1 PE=1 SV=4 - [SYNE1_HUMAN]	0,36	2	2	5	Transmembrane
Q9NVU7	Protein SDA1 homolog OS=Homo sapiens GN=SDAD1 PE=1 SV=3 - [SDA1_HUMAN]	2,04	1	1	5	Transmembrane
Q93050	V-type proton ATPase 116 kDa subunit a isoform 1 OS=Homo sapiens GN=ATP6V0A1 PE=1 SV=3 -	2,15	1	1	5	Transmembrane
O15399	Glutamate receptor ionotropic, NMDA 2D OS=Homo sapiens GN=GRIN2D PE=1 SV=2 - [NMDE4_HU	1,35	1	1	5	Transmembrane
A1L0T0	Acetolactate synthase-like protein OS=Homo sapiens GN=ILVBL PE=1 SV=2 - [ILVBL_HUMAN]	2,85	1	1	4	Extracellular
Q6ZTR5	Cilia- and flagella-associated protein 47 OS=Homo sapiens GN=CFAP47 PE=2 SV=4 - [CFA47_HUM	0,52	1	1	4	Extracellular
Q9P2M7	Cingulin OS=Homo sapiens GN=CGN PE=1 SV=2 - [CING_HUMAN]	1,17	1	1	4	Extracellular
Q8TBZ0	Coiled-coil domain-containing protein 110 OS=Homo sapiens GN=CCDC110 PE=1 SV=1 - [CC110_H	2,76	1	1	4	Extracellular
Q4VC31	Coiled-coil domain-containing protein 58 OS=Homo sapiens GN=CCDC58 PE=1 SV=1 - [CCD58_HUI	15,28	1	1	4	Extracellular
P81605	Dermcidin OS=Homo sapiens GN=DCD PE=1 SV=2 - [DCD_HUMAN]	20	2	2	4	Extracellular
Q99708	DNA endonuclease RBBP8 OS=Homo sapiens GN=RBBP8 PE=1 SV=2 - [COM1_HUMAN]	2,12	1	1	4	Extracellular
Q8WXX0	Dynein heavy chain 7, axonemal OS=Homo sapiens GN=DNAH7 PE=1 SV=2 - [DYH7_HUMAN]	0,72	2	2	4	Extracellular
Q9UKT6	F-box/LRR-repeat protein 21 OS=Homo sapiens GN=FBXL21 PE=1 SV=1 - [FXL21_HUMAN]	3,23	1	1	4	Extracellular
Q9P266	Junctional protein associated with coronary artery disease OS=Homo sapiens GN=KIAA1462 PE=1 S	0,96	1	1	4	Extracellular
Q5T749	Keratinocyte proline-rich protein OS=Homo sapiens GN=KPRP PE=1 SV=1 - [KPRP_HUMAN]	3,8	2	2	4	Extracellular
P52732	Kinesin-like protein KIF11 OS=Homo sapiens GN=KIF11 PE=1 SV=2 - [KIF11_HUMAN]	1,42	1	1	4	Extracellular
O60333	Kinesin-like protein KIF1B OS=Homo sapiens GN=KIF1B PE=1 SV=5 - [KIF1B_HUMAN]	1,54	2	2	4	Extracellular
Q9Y234	Lipoyltransferase 1, mitochondrial OS=Homo sapiens GN=LIPT1 PE=1 SV=1 - [LIPT_HUMAN]	3,75	1	1	4	Extracellular
Q8ND30	Liprin-beta-2 OS=Homo sapiens GN=PPFIBP2 PE=1 SV=3 - [LIPB2_HUMAN]	0,57	1	1	4	Extracellular
O75665	Oral-facial-digital syndrome 1 protein OS=Homo sapiens GN=OFD1 PE=1 SV=1 - [OFD1_HUMAN]	2,27	1	1	4	Extracellular
P50897	Palmitoyl-protein thioesterase 1 OS=Homo sapiens GN=PPT1 PE=1 SV=1 - [PPT1_HUMAN]	4,9	1	1	4	Extracellular
O75167	Phosphatase and actin regulator 2 OS=Homo sapiens GN=PHACTR2 PE=1 SV=2 - [PHAR2_HUMAN	2,05	1	1	4	Extracellular
Q15468	SCL-interrupting locus protein OS=Homo sapiens GN=STIL PE=1 SV=2 - [STIL_HUMAN]	1,01	1	1	4	Extracellular
Q9BRS2	Serine/threonine-protein kinase RIO1 OS=Homo sapiens GN=RIOK1 PE=1 SV=2 - [RIOK1_HUMAN]	7,22	3	3	4	Extracellular
Q8TF05	Serine/threonine-protein phosphatase 4 regulatory subunit 1 OS=Homo sapiens GN=PPP4R1 PE=1 S	2,74	1	2	4	Extracellular
Q9P0W8	Spermatogenesis-associated protein 7 OS=Homo sapiens GN=SPATA7 PE=1 SV=3 - [SPAT7_HUM/	1,17	1	1	4	Extracellular
O60347	TBC1 domain family member 12 OS=Homo sapiens GN=TBC1D12 PE=1 SV=3 - [TBC12_HUMAN]	3,61	2	2	4	Extracellular
Q9Y2I9	TBC1 domain family member 30 OS=Homo sapiens GN=TBC1D30 PE=1 SV=2 - [TBC30_HUMAN]	3,79	2	2	4	Extracellular
Q96RL7	Vacuolar protein sorting-associated protein 13A OS=Homo sapiens GN=VPS13A PE=1 SV=2 - [VP13	0,44	1	1	4	Extracellular
Q8TCN5	Zinc finger protein 507 OS=Homo sapiens GN=ZNF507 PE=1 SV=2 - [ZN507_HUMAN]	2,41	2	2	4	Extracellular
Q13129	Zinc finger protein Rlf OS=Homo sapiens GN=RLF PE=1 SV=2 - [RLF_HUMAN]	0,94	1	1	4	Extracellular
A2RUB6	Coiled-coil domain-containing protein 66 OS=Homo sapiens GN=CCDC66 PE=1 SV=4 - [CCD66_HUI	2,95	3	3	4	Extracellular
A8MUZ8	Putative zinc finger protein 705G OS=Homo sapiens GN=ZNF705G PE=2 SV=2 - [Z705G_HUMAN]	6	1	1	4	Extracellular
O75414	Nucleoside diphosphate kinase 6 OS=Homo sapiens GN=NME6 PE=1 SV=3 - [NDK6_HUMAN]	8,06	2	2	4	Extracellular
P0DJI8	Serum amyloid A-1 protein OS=Homo sapiens GN=SAA1 PE=1 SV=1 - [SAA1_HUMAN]	11,48	1	1	4	Extracellular

P14866	Heterogeneous nuclear ribonucleoprotein L OS=Homo sapiens GN=HNRNPL PE=1 SV=2 - [HNRPL_	0,85	1	1	4	Extracellular
P52746	Zinc finger protein 142 OS=Homo sapiens GN=ZNF142 PE=1 SV=4 - [ZN142_HUMAN]	0,36	1	1	4	Extracellular
Q02224	Centromere-associated protein E OS=Homo sapiens GN=CENPE PE=1 SV=2 - [CENPE_HUMAN]	0,41	1	1	4	Extracellular
Q05952	Nuclear transition protein 2 OS=Homo sapiens GN=TNP2 PE=1 SV=1 - [STP2_HUMAN]	15,22	1	1	4	Extracellular
Q96BH1	E3 ubiquitin-protein ligase RNF25 OS=Homo sapiens GN=RNF25 PE=1 SV=1 - [RNF25_HUMAN]	4,58	1	1	4	Extracellular
Q9BW85	Coiled-coil domain-containing protein 94 OS=Homo sapiens GN=CCDC94 PE=1 SV=1 - [CCD94_HU	4,02	1	1	4	Extracellular
O43292	Glycosylphosphatidylinositol anchor attachment 1 protein OS=Homo sapiens GN=GPAA1 PE=1 SV=3	2,09	1	1	4	Transmembrane
Q6UXB8	Peptidase inhibitor 16 OS=Homo sapiens GN=PI16 PE=1 SV=1 - [PI16_HUMAN]	5,18	2	2	4	Transmembrane
Q9BXS4	Transmembrane protein 59 OS=Homo sapiens GN=TMEM59 PE=1 SV=1 - [TMM59_HUMAN]	4,95	1	1	4	Transmembrane
Q8IV08	Phospholipase D3 OS=Homo sapiens GN=PLD3 PE=1 SV=1 - [PLD3_HUMAN]	2,45	1	1	4	Transmembrane
Q8NE01	Metal transporter CNNM3 OS=Homo sapiens GN=CNNM3 PE=1 SV=1 - [CNNM3_HUMAN]	1,56	1	1	4	Transmembrane
Q9C0I4	Thrombospondin type-1 domain-containing protein 7B OS=Homo sapiens GN=THSD7B PE=2 SV=2 -	1,43	1	1	4	Transmembrane
Q07002	Cyclin-dependent kinase 18 OS=Homo sapiens GN=CDK18 PE=1 SV=3 - [CDK18_HUMAN]	5,3	2	2	3	Cytoplasm
Q4KWH8	1-phosphatidylinositol 4,5-bisphosphate phosphodiesterase eta-1 OS=Homo sapiens GN=PLCH1 PE	1,42	1	2	3	Extracellular
Q99996	A-kinase anchor protein 9 OS=Homo sapiens GN=AKAP9 PE=1 SV=3 - [AKAP9_HUMAN]	0,77	2	2	3	Extracellular
Q8IVF6	Ankyrin repeat domain-containing protein 18A OS=Homo sapiens GN=ANKRD18A PE=2 SV=3 - [AN1	1,61	1	1	3	Extracellular
Q9UPS8	Ankyrin repeat domain-containing protein 26 OS=Homo sapiens GN=ANKRD26 PE=1 SV=3 - [ANR26	1,11	1	1	3	Extracellular
Q96QE3	ATPase family AAA domain-containing protein 5 OS=Homo sapiens GN=ATAD5 PE=1 SV=4 - [ATAD	0,6	1	1	3	Extracellular
Q7Z478	ATP-dependent RNA helicase DHX29 OS=Homo sapiens GN=DHX29 PE=1 SV=2 - [DHX29_HUMAN	1,53	1	2	3	Extracellular
Q68CP9	AT-rich interactive domain-containing protein 2 OS=Homo sapiens GN=ARID2 PE=1 SV=2 - [ARID2_	2,02	2	2	3	Extracellular
Q14865	AT-rich interactive domain-containing protein 5B OS=Homo sapiens GN=ARID5B PE=1 SV=3 - [ARI5	1,09	1	1	3	Extracellular
P04003	C4b-binding protein alpha chain OS=Homo sapiens GN=C4BPA PE=1 SV=2 - [C4BPA_HUMAN]	5,03	2	2	3	Extracellular
D6REC4	Cilia- and flagella-associated protein 99 OS=Homo sapiens GN=CFAP99 PE=3 SV=1 - [CFA99_HUM	2,83	1	1	3	Extracellular
P05160	Coagulation factor XIII B chain OS=Homo sapiens GN=F13B PE=1 SV=3 - [F13B_HUMAN]	3,63	1	1	3	Extracellular
Q8NEL0	Coiled-coil domain-containing protein 54 OS=Homo sapiens GN=CCDC54 PE=1 SV=2 - [CCD54_HU	6,4	1	1	3	Extracellular
Q02388	Collagen alpha-1(VII) chain OS=Homo sapiens GN=COL7A1 PE=1 SV=2 - [CO7A1_HUMAN]	0,27	1	1	3	Extracellular
P08123	Collagen alpha-2(I) chain OS=Homo sapiens GN=COL1A2 PE=1 SV=7 - [CO1A2_HUMAN]	2,78	2	2	3	Extracellular
Q15828	Cystatin-M OS=Homo sapiens GN=CST6 PE=1 SV=1 - [CYTM_HUMAN]	14,09	1	1	3	Extracellular
Q6ZMK1	Cysteine and histidine-rich protein 1 OS=Homo sapiens GN=CYHR1 PE=1 SV=2 - [CYHR1_HUMAN]	5,8	2	2	3	Extracellular
Q6IQ26	DENN domain-containing protein 5A OS=Homo sapiens GN=DENND5A PE=1 SV=2 - [DEN5A_HUM	1,55	1	1	3	Extracellular
P26358	DNA (cytosine-5)-methyltransferase 1 OS=Homo sapiens GN=DNMT1 PE=1 SV=2 - [DNMT1_HUMAN	0,87	1	1	3	Extracellular
Q03468	DNA excision repair protein ERCC-6 OS=Homo sapiens GN=ERCC6 PE=1 SV=1 - [ERCC6_HUMAN	1,07	1	1	3	Extracellular
Q9P2D7	Dynein heavy chain 1, axonemal OS=Homo sapiens GN=DNAH1 PE=2 SV=4 - [DYH1_HUMAN]	0,88	2	2	3	Extracellular
Q9H2F5	Enhancer of polycomb homolog 1 OS=Homo sapiens GN=EPC1 PE=1 SV=1 - [EPC1_HUMAN]	3,95	2	2	3	Extracellular
P14136	Glial fibrillary acidic protein OS=Homo sapiens GN=GFAP PE=1 SV=1 - [GFAP_HUMAN]	7,64	2	3	3	Extracellular
Q6IB77	Glycine N-acyltransferase OS=Homo sapiens GN=GLYAT PE=1 SV=3 - [GLYAT_HUMAN]	5,41	1	1	3	Extracellular
P62873	Guanine nucleotide-binding protein G(I)/G(S)/G(T) subunit beta-1 OS=Homo sapiens GN=GNB1 PE=	10,59	3	3	3	Extracellular
Q9Y4B4	Helicase ARIP4 OS=Homo sapiens GN=RAD54L2 PE=1 SV=4 - [ARIP4_HUMAN]	0,68	1	1	3	Extracellular
Q4G0P3	Hydrocephalus-inducing protein homolog OS=Homo sapiens GN=HYDIN PE=1 SV=3 - [HYDIN_HUM	0,29	2	2	3	Extracellular
P01743	Ig heavy chain V-I region HG3 OS=Homo sapiens PE=3 SV=1 - [HV102_HUMAN]	9,4	1	1	3	Extracellular
P17936	Insulin-like growth factor-binding protein 3 OS=Homo sapiens GN=IGFBP3 PE=1 SV=2 - [IBP3_HUM	5,84	1	1	3	Extracellular
Q496Y0	LON peptidase N-terminal domain and RING finger protein 3 OS=Homo sapiens GN=LONRF3 PE=1 S	3,95	2	2	3	Extracellular
Q14676	Mediator of DNA damage checkpoint protein 1 OS=Homo sapiens GN=MDC1 PE=1 SV=3 - [MDC1_H	0,86	1	1	3	Extracellular
Q7Z406	Myosin-14 OS=Homo sapiens GN=MYH14 PE=1 SV=2 - [MYH14_HUMAN]	1,8	2	2	3	Extracellular
P20929	Nebulin OS=Homo sapiens GN=NEB PE=1 SV=5 - [NEBU_HUMAN]	0,37	2	2	3	Extracellular
O75113	NEDD4-binding protein 1 OS=Homo sapiens GN=N4BP1 PE=1 SV=4 - [N4BP1_HUMAN]	3,01	1	1	3	Extracellular
Q8IVL0	Neuron navigator 3 OS=Homo sapiens GN=NAV3 PE=1 SV=3 - [NAV3_HUMAN]	0,92	2	2	3	Extracellular
O95428	Papilin OS=Homo sapiens GN=PAPLN PE=2 SV=4 - [PPN_HUMAN]	2,58	2	2	3	Extracellular
P68106	Peptidyl-prolyl cis-trans isomerase FKBP1B OS=Homo sapiens GN=FKBP1B PE=1 SV=2 - [FKB1B_H	15,74	1	1	3	Extracellular
Q96PX9	Pleckstrin homology domain-containing family G member 4B OS=Homo sapiens GN=PLEKHG4B PE	2,36	2	2	3	Extracellular
Q6PGQ7	Protein aurora borealis OS=Homo sapiens GN=BORA PE=1 SV=2 - [BORA_HUMAN]	2,15	1	1	3	Extracellular
Q86TB9	Protein PAT1 homolog 1 OS=Homo sapiens GN=PATL1 PE=1 SV=2 - [PATL1_HUMAN]	2,08	1	1	3	Extracellular
P20396	Pro-thyrotropin-releasing hormone OS=Homo sapiens GN=TRH PE=1 SV=1 - [TRH_HUMAN]	11,98	1	2	3	Extracellular
P15498	Proto-oncogene vav OS=Homo sapiens GN=VAV1 PE=1 SV=4 - [VAV_HUMAN]	1,78	1	1	3	Extracellular
Q9H5N1	Rab GTPase-binding effector protein 2 OS=Homo sapiens GN=RABEP2 PE=1 SV=2 - [RABE2_HUM	2,11	1	1	3	Extracellular
P49795	Regulator of G-protein signaling 19 OS=Homo sapiens GN=RGS19 PE=1 SV=1 - [RGS19_HUMAN]	10,6	1	1	3	Extracellular
O75916	Regulator of G-protein signaling 9 OS=Homo sapiens GN=RGS9 PE=1 SV=1 - [RGS9_HUMAN]	4,01	2	2	3	Extracellular
Q86UN2	Reticulon-4 receptor-like 1 OS=Homo sapiens GN=RTN4RL1 PE=1 SV=1 - [R4RL1_HUMAN]	3,4	1	1	3	Extracellular
Q13247	Serine/arginine-rich splicing factor 6 OS=Homo sapiens GN=SRSF6 PE=1 SV=2 - [SRSF6_HUMAN]	2,03	1	1	3	Extracellular
Q5T5P2	Sickle tail protein homolog OS=Homo sapiens GN=KIAA1217 PE=1 SV=2 - [SKT_HUMAN]	0,93	1	1	3	Extracellular
Q9NTI5	Sister chromatid cohesion protein PDS5 homolog B OS=Homo sapiens GN=PDS5B PE=1 SV=1 - [PD	0,41	1	1	3	Extracellular
Q9NRC6	Spectrin beta chain, non-erythrocytic 5 OS=Homo sapiens GN=SPTBN5 PE=1 SV=2 - [SPTN5_HUM	1,09	2	2	3	Extracellular
O00186	Syntaxin-binding protein 3 OS=Homo sapiens GN=STXBP3 PE=1 SV=2 - [STXB3_HUMAN]	2,7	1	1	3	Extracellular
P08631	Tyrosine-protein kinase HCK OS=Homo sapiens GN=HCK PE=1 SV=5 - [HCK_HUMAN]	6,65	2	2	3	Extracellular
Q92628	Uncharacterized protein KIAA0232 OS=Homo sapiens GN=KIAA0232 PE=1 SV=5 - [K0232_HUMAN]	1,43	1	1	3	Extracellular
P49750	YLP motif-containing protein 1 OS=Homo sapiens GN=YLPM1 PE=1 SV=3 - [YLPM1_HUMAN]	0,82	2	2	3	Extracellular
A0AVK6	Transcription factor E2F8 OS=Homo sapiens GN=E2F8 PE=1 SV=1 - [E2F8_HUMAN]	1,27	1	1	3	Extracellular
A2RRP1	Neuroblastoma-amplified sequence OS=Homo sapiens GN=NBAS PE=1 SV=2 - [NBAS_HUMAN]	1,01	1	2	3	Extracellular
E7EU14	Protein PPP5D1 OS=Homo sapiens GN=PPP5D1 PE=2 SV=2 - [PP5D1_HUMAN]	4,68	1	1	3	Extracellular
O60566	Mitotic checkpoint serine/threonine-protein kinase BUB1 beta OS=Homo sapiens GN=BUB1B PE=1 S	0,86	1	1	3	Extracellular
O75127	Pentatricopeptide repeat-containing protein 1, mitochondrial OS=Homo sapiens GN=PTCD1 PE=1 SV	2,43	1	1	3	Extracellular
P02771	Alpha-fetoprotein OS=Homo sapiens GN=AFP PE=1 SV=1 - [FETA_HUMAN]	1,15	1	1	3	Extracellular
P23528	Cofilin-1 OS=Homo sapiens GN=CFL1 PE=1 SV=3 - [COF1_HUMAN]	16,87	1	1	3	Extracellular
P48728	Aminomethyltransferase, mitochondrial OS=Homo sapiens GN=AMT PE=1 SV=1 - [GCST_HUMAN]	5,21	1	1	3	Extracellular
Q15796	Mothers against decapentaplegic homolog 2 OS=Homo sapiens GN=SMAD2 PE=1 SV=1 - [SMAD2_	2,36	1	1	3	Extracellular
Q3B7J2	Glucose-fructose oxidoreductase domain-containing protein 2 OS=Homo sapiens GN=GFOD2 PE=2 S	4,42	1	1	3	Extracellular
Q4KMZ1	IQ domain-containing protein C OS=Homo sapiens GN=IQCC PE=2 SV=2 - [IQCC_HUMAN]	6,65	2	2	3	Extracellular
Q5CZC0	Fibrous sheath-interacting protein 2 OS=Homo sapiens GN=FSIP2 PE=2 SV=4 - [FSIP2_HUMAN]	0,22	1	1	3	Extracellular
Q5TC84	Opioid growth factor receptor-like protein 1 OS=Homo sapiens GN=OGFRL1 PE=2 SV=1 - [OGRL1_H	4,88	2	2	3	Extracellular
Q6ZN30	Zinc finger protein basonuclin-2 OS=Homo sapiens GN=BNC2 PE=1 SV=1 - [BNC2_HUMAN]	0,55	1	1	3	Extracellular
Q8N2C7	Protein unc-80 homolog OS=Homo sapiens GN=UNC80 PE=2 SV=2 - [UNC80_HUMAN]	0,25	1	1	3	Extracellular
Q92917	G patch domain and KOW motifs-containing protein OS=Homo sapiens GN=GPKOW PE=1 SV=2 - [G	3,15	1	1	3	Extracellular
Q96PE3	Type I inositol 3,4-bisphosphate 4-phosphatase OS=Homo sapiens GN=INPP4A PE=1 SV=1 - [INP4A	1,43	1	1	3	Extracellular
Q96RR4	Calcium/calmodulin-dependent protein kinase kinase 2 OS=Homo sapiens GN=CAMKK2 PE=1 SV=2	1,87	1	1	3	Extracellular
Q9H161	Homeobox protein aristaless-like 4 OS=Homo sapiens GN=ALX4 PE=1 SV=2 - [ALX4_HUMAN]	4,62	1	1	3	Extracellular
Q9H7S9	Zinc finger protein 703 OS=Homo sapiens GN=ZNF703 PE=1 SV=1 - [ZN703_HUMAN]	2,03	1	1	3	Extracellular
Q9P215	Pogo transposable element with KRAB domain OS=Homo sapiens GN=POGK PE=1 SV=2 - [POGK_	1,64	1	1	3	Extracellular
Q9Y2D8	Afadin- and alpha-actinin-binding protein OS=Homo sapiens GN=SSX2IP PE=1 SV=3 - [ADIP_HUMA	1,47	1	1	3	Extracellular
Q9Y4K1	Absent in melanoma 1 protein OS=Homo sapiens GN=AIM1 PE=1 SV=3 - [AIM1_HUMAN]	1,22	1	1	3	Extracellular
Q9UBM8	Alpha-1,3-mannosyl-glycoprotein 4-beta-N-acetylglucosaminyltransferase C OS=Homo sapiens GN=M	4,6	2	2	3	Transmembrane
Q08554	Desmocollin-1 OS=Homo sapiens GN=DSC1 PE=1 SV=2 - [DSC1_HUMAN]	1,68	1	1	3	Transmembrane
Q9UF33	Ephrin type-A receptor 6 OS=Homo sapiens GN=EPHA6 PE=2 SV=3 - [EPHA6_HUMAN]	1,83	1	1	3	Transmembrane
P42702	Leukemia inhibitory factor receptor OS=Homo sapiens GN=LIFR PE=1 SV=1 - [LIFR_HUMAN]	1	1	1	3	Transmembrane
O75829	Leukocyte cell-derived chemotaxin 1 OS=Homo sapiens GN=LECT1 PE=1 SV=1 - [LECT1_HUMAN]	3,59	1	1	3	Transmembrane
Q8IUK5	Plexin domain-containing protein 1 OS=Homo sapiens GN=PLXDC1 PE=1 SV=2 - [PLDX1_HUMAN]	2,4	1	1	3	Transmembrane
Q5FWE3	Proline-rich Transmembrane protein 3 OS=Homo sapiens GN=PRRT3 PE=1 SV=3 - [PRRT3_HUMAN	1,83	1	1	3	Transmembrane
P21817	Ryanodine receptor 1 OS=Homo sapiens GN=RYR1 PE=1 SV=3 - [RYR1_HUMAN]	0,58	2	2	3	Transmembrane
Q8TBE7	Solute carrier family 35 member G2 OS=Homo sapiens GN=SLC35G2 PE=1 SV=3 - [S35G2_HUMAN	2,18	1	1	3	Transmembrane
Q5TH69	Brefeldin A-inhibited guanine nucleotide-exchange protein 3 OS=Homo sapiens GN=ARFGEF3 PE=1	0,78	1	1	3	Transmembrane
Q6ZUT9	DENN domain-containing protein 5B OS=Homo sapiens GN=DENND5B PE=1 SV=2 - [DEN5B_HUM	1,33	1	1	3	Transmembrane
Q9BSA9	Endosomal/lysomomal potassium channel TMEM175 OS=Homo sapiens GN=TMEM175 PE=1 SV=1	2,58	1	1	3	Transmembrane
P62829	60S ribosomal protein L23 OS=Homo sapiens GN=RPL23 PE=1 SV=1 - [RL23_HUMAN]	16,43	1	1	2	Cytoplasm
P56377	AP-1 complex subunit sigma-2 OS=Homo sapiens GN=AP1S2 PE=1 SV=1 - [AP1S2_HUMAN]	10,19	1	1	2	Cytoplasm
Q8NA47	Coiled-coil domain-containing protein 63 OS=Homo sapiens GN=CCDC63 PE=2 SV=1 - [CCD63_HU	3,2	1	2	2	Cytoplasm
Q9BZW7	Testis-specific gene 10 protein OS=Homo sapiens GN=TSGA10 PE=1 SV=1 - [TSG10_HUMAN]	2,01	1	1	2	Cytoplasm
P51178	1-phosphatidylinositol 4,5-bisphosphate phosphodiesterase delta-1 OS=Homo sapiens GN=PLCD1 P	2,65	1	1	2	Extracellular
Q9UGJ0	5'-AMP-activated protein kinase subunit gamma-2 OS=Homo sapiens GN=PRKAG2 PE=1 SV=1 - [AA	2,28	1	1	2	Extracellular
P02511	Alpha-crystallin B chain OS=Homo sapiens GN=CRYAB PE=1 SV=2 - [CRYAB_HUMAN]	4,57	1	1	2	Extracellular
Q9UJX3	Anaphase-promoting complex subunit 7 OS=Homo sapiens GN=ANAPC7 PE=1 SV=4 - [APC7_HUM	2,17	1	1	2	Extracellular
P53673	Beta-crystallin A4 OS=Homo sapiens GN=CRYBA4 PE=1 SV=3 - [CRBA4_HUMAN]	7,65	1	1	2	Extracellular
Q9UBD9	Cardiotrophin-like cytokine factor 1 OS=Homo sapiens GN=CLCF1 PE=1 SV=1 - [CLCF1_HUMAN]	10,22	1	1	2	Extracellular
P51878	Caspase-5 OS=Homo sapiens GN=CASP5 PE=1 SV=3 - [CASP5_HUMAN]	4,15	1	1	2	Extracellular
Q99618	Cell division cycle-associated protein 3 OS=Homo sapiens GN=CDCA3 PE=1 SV=1 - [CDCA3_HUM	4,85	1	1	2	Extracellular
Q5M9N0	Coiled-coil domain-containing protein 158 OS=Homo sapiens GN=CCDC158 PE=1 SV=2 - [CD158_H	1,26	1	1	2	Extracellular
Q8WXI2	Connector enhancer of kinase suppressor of ras 2 OS=Homo sapiens GN=CNKSR2 PE=1 SV=1 - [CN	2,13	1	1	2	Extracellular

Q14746	Conserved oligomeric Golgi complex subunit 2 OS=Homo sapiens GN=COG2 PE=1 SV=1 - [COG2_H	4,34	2	2	2	Extracellular
Q8NCM8	Cytoplasmic dynein 2 heavy chain 1 OS=Homo sapiens GN=DYNC2H1 PE=1 SV=4 - [DYHC2_HUMA	0,37	1	1	2	Extracellular
P53355	Death-associated protein kinase 1 OS=Homo sapiens GN=DAPK1 PE=1 SV=6 - [DAPK1_HUMAN]	0,77	1	1	2	Extracellular
Q68D51	DENN domain-containing protein 2C OS=Homo sapiens GN=DENND2C PE=1 SV=2 - [DEN2C_HUM	2,59	1	1	2	Extracellular
Q9Y620	DNA repair and recombination protein RAD54B OS=Homo sapiens GN=RAD54B PE=1 SV=1 - [RA54	1,1	1	1	2	Extracellular
Q92878	DNA repair protein RAD50 OS=Homo sapiens GN=RAD50 PE=1 SV=1 - [RAD50_HUMAN]	1,98	2	2	2	Extracellular
P51530	DNA replication ATP-dependent helicase/nuclease DNA2 OS=Homo sapiens GN=DNA2 PE=1 SV=3	0,85	1	1	2	Extracellular
Q92547	DNA topoisomerase 2-binding protein 1 OS=Homo sapiens GN=TOPBP1 PE=1 SV=3 - [TOPB1_HUM	1,84	2	2	2	Extracellular
O60313	Dynamin-like 120 kDa protein, mitochondrial OS=Homo sapiens GN=OPA1 PE=1 SV=3 - [OPA1_HUM	1,04	1	1	2	Extracellular
Q96J02	E3 ubiquitin-protein ligase Itchy homolog OS=Homo sapiens GN=ITCH PE=1 SV=2 - [ITCH_HUMAN]	3,88	2	2	2	Extracellular
Q9HCE0	Ectopic P granules protein 5 homolog OS=Homo sapiens GN=EPG5 PE=2 SV=2 - [EPG5_HUMAN]	0,66	2	2	2	Extracellular
A8K855	EF-hand calcium-binding domain-containing protein 7 OS=Homo sapiens GN=EFCAB7 PE=2 SV=1 -	3,66	1	1	2	Extracellular
P49448	Glutamate dehydrogenase 2, mitochondrial OS=Homo sapiens GN=GLUD2 PE=1 SV=2 - [DHE4_HUI	2,33	1	1	2	Extracellular
P09488	Glutathione S-transferase Mu 1 OS=Homo sapiens GN=GSTM1 PE=1 SV=3 - [GSTM1_HUMAN]	8,72	1	1	2	Extracellular
O75063	Glycosaminoglycan xylosylkinase OS=Homo sapiens GN=FAM20B PE=1 SV=1 - [XYLK_HUMAN]	2,93	1	1	2	Extracellular
Q4V328	GRIP1-associated protein 1 OS=Homo sapiens GN=GRIPAP1 PE=1 SV=1 - [GRAP1_HUMAN]	1,78	1	1	2	Extracellular
O95251	Histone acetyltransferase KAT7 OS=Homo sapiens GN=KAT7 PE=1 SV=1 - [KAT7_HUMAN]	3,11	1	1	2	Extracellular
Q9UBN7	Histone deacetylase 6 OS=Homo sapiens GN=HDAC6 PE=1 SV=2 - [HDAC6_HUMAN]	1,4	1	1	2	Extracellular
Q96KQ7	Histone-lysine N-methyltransferase EHMT2 OS=Homo sapiens GN=EHMT2 PE=1 SV=3 - [EHMT2_H	1,82	1	1	2	Extracellular
Q9BZ95	Histone-lysine N-methyltransferase NSD3 OS=Homo sapiens GN=WHSC1L1 PE=1 SV=1 - [NSD3_H	2,64	2	2	2	Extracellular
P01612	Ig kappa chain V-I region Mev OS=Homo sapiens PE=1 SV=1 - [KV120_HUMAN]	16,51	1	1	2	Extracellular
P01714	Ig lambda chain V-III region SH OS=Homo sapiens PE=1 SV=1 - [LV301_HUMAN]	16,67	1	1	2	Extracellular
Q5VZK9	Leucine-rich repeat-containing protein 16A OS=Homo sapiens GN=LRRC16A PE=1 SV=1 - [LR16A_H	1,46	2	2	2	Extracellular
Q9Y4F3	Meiosis arrest female protein 1 OS=Homo sapiens GN=KIAA0430 PE=1 SV=6 - [MARF1_HUMAN]	1,03	1	1	2	Extracellular
Q6P0N0	Mis18-binding protein 1 OS=Homo sapiens GN=MIS18BP1 PE=1 SV=1 - [M18BP_HUMAN]	3	2	2	2	Extracellular
Q969M1	Mitochondrial import receptor subunit TOM40B OS=Homo sapiens GN=TOMM40L PE=1 SV=1 - [TM4	4,87	1	1	2	Extracellular
P84022	Mothers against decapentaplegic homolog 3 OS=Homo sapiens GN=SMAD3 PE=1 SV=1 - [SMAD3_I	3,76	1	1	2	Extracellular
P35579	Myosin-9 OS=Homo sapiens GN=MYH9 PE=1 SV=4 - [MYH9_HUMAN]	0,41	1	1	2	Extracellular
Q8IXJ6	NAD-dependent protein deacetylase sirtuin-2 OS=Homo sapiens GN=SIRT2 PE=1 SV=2 - [SIR2_HUI	5,14	1	1	2	Extracellular
Q3BBV0	Neuroblastoma breakpoint family member 1 OS=Homo sapiens GN=NBPF1 PE=2 SV=1 - [NBPF1_H	1,81	1	1	2	Extracellular
Q9NSY0	Nuclear receptor-binding protein 2 OS=Homo sapiens GN=NRBP2 PE=2 SV=2 - [NRBP2_HUMAN]	3,19	1	1	2	Extracellular
Q9ULE6	Paladin OS=Homo sapiens GN=PALD1 PE=1 SV=3 - [PALD_HUMAN]	5,49	2	2	2	Extracellular
Q92569	Phosphatidylinositol 3-kinase regulatory subunit gamma OS=Homo sapiens GN=PIK3R3 PE=1 SV=2	3,9	1	1	2	Extracellular
Q7Z7M9	Polypeptide N-acetylgalactosaminyltransferase 5 OS=Homo sapiens GN=GALNT5 PE=1 SV=1 - [GAL	0,85	1	1	2	Extracellular
Q7L014	Probable ATP-dependent RNA helicase DDX46 OS=Homo sapiens GN=DDX46 PE=1 SV=2 - [DDX46	1,55	1	1	2	Extracellular
Q8NG31	Protein CASC5 OS=Homo sapiens GN=CASC5 PE=1 SV=3 - [CASC5_HUMAN]	0,64	1	1	2	Extracellular
P05109	Protein S100-A8 OS=Homo sapiens GN=S100A8 PE=1 SV=1 - [S10A8_HUMAN]	23,66	2	2	2	Extracellular
Q9UJV8	Purine-rich element-binding protein gamma OS=Homo sapiens GN=PURG PE=2 SV=1 - [PURG_HUM	4,9	1	1	2	Extracellular
Q8WXQ3	Putative uncharacterized protein encoded by LINC01599 OS=Homo sapiens GN=LINC01599 PE=2 S	4,63	1	1	2	Extracellular
Q14699	Raftlin OS=Homo sapiens GN=RFTN1 PE=1 SV=4 - [RFTN1_HUMAN]	1,9	1	1	2	Extracellular
Q5T481	RNA-binding protein 20 OS=Homo sapiens GN=RBM20 PE=1 SV=3 - [RBM20_HUMAN]	1,55	1	1	2	Extracellular
Q59EK9	RUN domain-containing protein 3A OS=Homo sapiens GN=RUNDC3A PE=1 SV=2 - [RUN3A_HUMAI	1,57	1	1	2	Extracellular
P10523	S-arrestin OS=Homo sapiens GN=SAG PE=1 SV=3 - [ARRS_HUMAN]	6,67	2	2	2	Extracellular
Q9P0W5	Schwannomin-interacting protein 1 OS=Homo sapiens GN=SCHIP1 PE=1 SV=1 - [SCHI1_HUMAN]	4,31	1	1	2	Extracellular
Q14563	Semaphorin-3A OS=Homo sapiens GN=SEMA3A PE=1 SV=1 - [SEM3A_HUMAN]	2,33	2	2	2	Extracellular
Q6P3R8	Serine/threonine-protein kinase Nek5 OS=Homo sapiens GN=NEK5 PE=2 SV=1 - [NEK5_HUMAN]	2,54	1	2	2	Extracellular
Q15173	Serine/threonine-protein phosphatase 2A 56 kDa regulatory subunit beta isoform OS=Homo sapiens (	1,01	1	1	2	Extracellular
Q96L03	Spermatogenesis-associated protein 17 OS=Homo sapiens GN=SPATA17 PE=2 SV=1 - [SPT17_HUI	3,88	1	1	2	Extracellular
P28290	Sperm-specific antigen 2 OS=Homo sapiens GN=SSFA2 PE=1 SV=3 - [SSFA2_HUMAN]	2,38	2	2	2	Extracellular
O95425	Supervillin OS=Homo sapiens GN=SVIL PE=1 SV=2 - [SVIL_HUMAN]	0,72	1	1	2	Extracellular
P49368	T-complex protein 1 subunit gamma OS=Homo sapiens GN=CCT3 PE=1 SV=4 - [TCPG_HUMAN]	5,14	2	2	2	Extracellular
Q9NXG2	THUMP domain-containing protein 1 OS=Homo sapiens GN=THUMPD1 PE=1 SV=2 - [THUM1_HUM,	6,23	1	1	2	Extracellular
Q6YHU6	Thyroid adenoma-associated protein OS=Homo sapiens GN=THADA PE=1 SV=1 - [THADA_HUMAN]	0,92	1	1	2	Extracellular
P48553	Trafficking protein particle complex subunit 10 OS=Homo sapiens GN=TRAPPC10 PE=1 SV=2 - [TPC	1,43	1	1	2	Extracellular
Q8NI51	Transcriptional repressor CTCFL OS=Homo sapiens GN=CTCFL PE=1 SV=2 - [CTCFL_HUMAN]	3,02	1	1	2	Extracellular
Q07283	Trichohyalin OS=Homo sapiens GN=TCHH PE=1 SV=2 - [TRHY_HUMAN]	1,03	1	2	2	Extracellular
P32019	Type II inositol 1,4,5-trisphosphate 5-phosphatase OS=Homo sapiens GN=INPP5B PE=1 SV=4 - [I5P	1,21	1	1	2	Extracellular
O00160	Unconventional myosin-If OS=Homo sapiens GN=MYO1F PE=1 SV=3 - [MYO1F_HUMAN]	2,73	2	2	2	Extracellular
P08670	Vimentin OS=Homo sapiens GN=VIM PE=1 SV=4 - [VIME_HUMAN]	2,15	1	1	2	Extracellular
Q02641	Voltage-dependent L-type calcium channel subunit beta-1 OS=Homo sapiens GN=CACNB1 PE=2 SV	3,51	1	1	2	Extracellular
Q5VZL5	Zinc finger MYM-type protein 4 OS=Homo sapiens GN=ZMYM4 PE=1 SV=1 - [ZMYM4_HUMAN]	1,03	1	1	2	Extracellular
O14628	Zinc finger protein 195 OS=Homo sapiens GN=ZNF195 PE=1 SV=2 - [ZN195_HUMAN]	3,34	1	1	2	Extracellular
Q2VY69	Zinc finger protein 284 OS=Homo sapiens GN=ZNF284 PE=2 SV=1 - [ZN284_HUMAN]	2,19	1	1	2	Extracellular
Q5JVG2	Zinc finger protein 484 OS=Homo sapiens GN=ZNF484 PE=1 SV=1 - [ZN484_HUMAN]	4,23	2	2	2	Extracellular
P62258	14-3-3 protein epsilon OS=Homo sapiens GN=YWHAE PE=1 SV=1 - [1433E_HUMAN]	4,71	1	1	2	Extracellular
P16885	1-phosphatidylinositol 4,5-bisphosphate phosphodiesterase gamma-2 OS=Homo sapiens GN=PLCG2	1,42	1	1	2	Extracellular
Q9ULD0	2-oxoglutarate dehydrogenase-like, mitochondrial OS=Homo sapiens GN=OGDHL PE=1 SV=3 - [OGI	2,08	1	1	2	Extracellular
Q13405	39S ribosomal protein L49, mitochondrial OS=Homo sapiens GN=MRPL49 PE=1 SV=1 - [RM49_HUM	3,61	1	1	2	Extracellular
O00763	Acetyl-CoA carboxylase 2 OS=Homo sapiens GN=ACACB PE=1 SV=3 - [ACACB_HUMAN]	0,69	1	1	2	Extracellular
Q9Y615	Actin-like protein 7A OS=Homo sapiens GN=ACTL7A PE=1 SV=1 - [ACL7A_HUMAN]	3,68	1	1	2	Extracellular
P82987	ADAMTS-like protein 3 OS=Homo sapiens GN=ADAMTSL3 PE=1 SV=4 - [ATL3_HUMAN]	0,59	1	1	2	Extracellular
Q9C0B1	Alpha-ketoglutarate-dependent dioxygenase FTO OS=Homo sapiens GN=FTO PE=1 SV=3 - [FTO_HU	1,58	1	1	2	Extracellular
Q7Z6G8	Ankyrin repeat and sterile alpha motif domain-containing protein 1B OS=Homo sapiens GN=ANKS1B	0,8	1	1	2	Extracellular
Q8N2N9	Ankyrin repeat domain-containing protein 36B OS=Homo sapiens GN=ANKRD36B PE=1 SV=4 - [AN3	0,81	1	1	2	Extracellular
Q01484	Ankyrin-2 OS=Homo sapiens GN=ANK2 PE=1 SV=4 - [ANK2_HUMAN]	0,33	1	1	2	Extracellular
P04114	Apolipoprotein B-100 OS=Homo sapiens GN=APOB PE=1 SV=2 - [APOB_HUMAN]	0,22	1	1	2	Extracellular
Q6PL18	ATPase family AAA domain-containing protein 2 OS=Homo sapiens GN=ATAD2 PE=1 SV=1 - [ATAD	1,01	1	1	2	Extracellular
Q8WXX7	Autism susceptibility gene 2 protein OS=Homo sapiens GN=AUTS2 PE=1 SV=1 - [AUTS2_HUMAN]	0,79	1	1	2	Extracellular
P05813	Beta-crystallin A3 OS=Homo sapiens GN=CRYBA1 PE=1 SV=4 - [CRBA1_HUMAN]	6,51	1	1	2	Extracellular
P60022	Beta-defensin 1 OS=Homo sapiens GN=DEFB1 PE=1 SV=1 - [DEFB1_HUMAN]	33,82	1	1	2	Extracellular
P13929	Beta-enolase OS=Homo sapiens GN=ENO3 PE=1 SV=5 - [ENOB_HUMAN]	5,53	1	1	2	Extracellular
Q96PL2	Beta-tectorin OS=Homo sapiens GN=TECTB PE=2 SV=1 - [TECTB_HUMAN]	2,74	1	1	2	Extracellular
Q9Y2F9	BTB/POZ domain-containing protein 3 OS=Homo sapiens GN=BTBD3 PE=2 SV=1 - [BTBD3_HUMAN	1,72	1	1	2	Extracellular
P62158	Calmodulin OS=Homo sapiens GN=CALM1 PE=1 SV=2 - [CALM_HUMAN]	11,41	1	1	2	Extracellular
Q9NP71	Carbohydrate-responsive element-binding protein OS=Homo sapiens GN=MLXIPL PE=1 SV=1 - [MLX	2,35	1	1	2	Extracellular
O60543	Cell death activator CIDE-A OS=Homo sapiens GN=CIDEA PE=1 SV=1 - [CIDEA_HUMAN]	6,85	1	1	2	Extracellular
Q69YH5	Cell division cycle-associated protein 2 OS=Homo sapiens GN=CDCA2 PE=1 SV=2 - [CDCA2_HUMA	1,47	1	1	2	Extracellular
Q8N137	Centrobin OS=Homo sapiens GN=CNTROB PE=1 SV=1 - [CNTRB_HUMAN]	2,1	1	1	2	Extracellular
Q9BV73	Centrosome-associated protein CEP250 OS=Homo sapiens GN=CEP250 PE=1 SV=2 - [CP250_HUM	0,41	1	1	2	Extracellular
Q86X52	Chondroitin sulfate synthase 1 OS=Homo sapiens GN=CHSY1 PE=1 SV=3 - [CHSS1_HUMAN]	1,62	1	1	2	Extracellular
Q8IYW2	Cilia- and flagella-associated protein 46 OS=Homo sapiens GN=CFAP46 PE=2 SV=3 - [CFA46_HUM	0,41	1	1	2	Extracellular
Q96MR6	Cilia- and flagella-associated protein 57 OS=Homo sapiens GN=CFAP57 PE=2 SV=3 - [CFA57_HUM	0,8	1	1	2	Extracellular
Q0VF96	Cingulin-like protein 1 OS=Homo sapiens GN=CGNL1 PE=1 SV=2 - [CGNL1_HUMAN]	1,61	1	1	2	Extracellular
Q8NCX0	Coiled-coil domain-containing protein 150 OS=Homo sapiens GN=CCDC150 PE=1 SV=2 - [CC150_H	1,63	1	1	2	Extracellular
A6NI56	Coiled-coil domain-containing protein 154 OS=Homo sapiens GN=CCDC154 PE=2 SV=4 - [CC154_H	1,63	1	1	2	Extracellular
Q8IWP9	Coiled-coil domain-containing protein 28A OS=Homo sapiens GN=CCDC28A PE=1 SV=1 - [CC28A_H	5,11	1	1	2	Extracellular
Q9UFE4	Coiled-coil domain-containing protein 39 OS=Homo sapiens GN=CCDC39 PE=2 SV=3 - [CCD39_HU	1,81	1	1	2	Extracellular
Q9UQ03	Coronin-2B OS=Homo sapiens GN=CORO2B PE=1 SV=4 - [COR2B_HUMAN]	2,08	1	1	2	Extracellular
Q96M20	Cyclic nucleotide-binding domain-containing protein 2 OS=Homo sapiens GN=CNBD2 PE=2 SV=2 - [(	3,65	1	1	2	Extracellular
P49589	Cysteine--tRNA ligase, cytoplasmic OS=Homo sapiens GN=CARS PE=1 SV=3 - [SYCC_HUMAN]	2,81	1	1	2	Extracellular
O43174	Cytochrome P450 26A1 OS=Homo sapiens GN=CYP26A1 PE=2 SV=2 - [CP26A_HUMAN]	2,21	1	1	2	Extracellular
Q14204	Cytoplasmic dynein 1 heavy chain 1 OS=Homo sapiens GN=DYNC1H1 PE=1 SV=5 - [DYHC1_HUMA	0,34	1	1	2	Extracellular
Q96MI9	Cytosolic carboxypeptidase 4 OS=Homo sapiens GN=AGBL1 PE=1 SV=2 - [CBPC4_HUMAN]	0,75	1	1	2	Extracellular
Q96HY6	DDRGK domain-containing protein 1 OS=Homo sapiens GN=DDRGK1 PE=1 SV=2 - [DDRGK_HUMA	3,5	1	1	2	Extracellular
Q14185	Dedicator of cytokinesis protein 1 OS=Homo sapiens GN=DOCK1 PE=1 SV=2 - [DOCK1_HUMAN]	0,8	1	1	2	Extracellular
Q9H7D0	Dedicator of cytokinesis protein 5 OS=Homo sapiens GN=DOCK5 PE=1 SV=3 - [DOCK5_HUMAN]	0,64	1	1	2	Extracellular
Q8NF50	Dedicator of cytokinesis protein 8 OS=Homo sapiens GN=DOCK8 PE=1 SV=3 - [DOCK8_HUMAN]	0,95	1	1	2	Extracellular
Q9BZ29	Dedicator of cytokinesis protein 9 OS=Homo sapiens GN=DOCK9 PE=1 SV=2 - [DOCK9_HUMAN]	0,53	1	1	2	Extracellular
P15924	Desmoplakin OS=Homo sapiens GN=DSP PE=1 SV=3 - [DESP_HUMAN]	0,7	1	1	2	Extracellular
Q2NKX8	DNA excision repair protein ERCC-6-like OS=Homo sapiens GN=ERCC6L PE=1 SV=1 - [ERC6L_HU	1,04	1	1	2	Extracellular
Q86Y56	Dynein assembly factor 5, axonemal OS=Homo sapiens GN=DNAAF5 PE=1 SV=4 - [DAAF5_HUMAN	1,29	1	1	2	Extracellular
Q96M86	Dynein heavy chain domain-containing protein 1 OS=Homo sapiens GN=DNHD1 PE=2 SV=2 - [DNHI	0,48	1	1	2	Extracellular
Q9H069	Dynein regulatory complex subunit 3 OS=Homo sapiens GN=DRC3 PE=2 SV=2 - [DRC3_HUMAN]	3,06	1	1	2	Extracellular
P13639	Elongation factor 2 OS=Homo sapiens GN=EEF2 PE=1 SV=4 - [EF2_HUMAN]	1,75	1	1	2	Extracellular
Q92611	ER degradation-enhancing alpha-mannosidase-like protein 1 OS=Homo sapiens GN=EDEM1 PE=1 S	2,74	1	1	2	Extracellular

Q96HE7	ERO1-like protein alpha OS=Homo sapiens GN=ERO1A PE=1 SV=2 - [ERO1A_HUMAN]	2,35	1	1	2	Extracellular
Q14152	Eukaryotic translation initiation factor 3 subunit A OS=Homo sapiens GN=EIF3A PE=1 SV=1 - [EIF3A	0,58	1	1	2	Extracellular
Q9Y2M0	Fanconi-associated nuclease 1 OS=Homo sapiens GN=FAN1 PE=1 SV=4 - [FAN1_HUMAN]	0,69	1	1	2	Extracellular
Q01469	Fatty acid-binding protein, epidermal OS=Homo sapiens GN=FABP5 PE=1 SV=3 - [FABP5_HUMAN]	10,37	1	1	2	Extracellular
B1AJZ9	Forkhead-associated domain-containing protein 1 OS=Homo sapiens GN=FHAD1 PE=2 SV=2 - [FHA	1,06	1	1	2	Extracellular
Q96PY5	Formin-like protein 2 OS=Homo sapiens GN=FMNL2 PE=1 SV=3 - [FMNL2_HUMAN]	2,12	1	1	2	Extracellular
P57678	Gem-associated protein 4 OS=Homo sapiens GN=GEMIN4 PE=1 SV=2 - [GEMI4_HUMAN]	2,08	1	1	2	Extracellular
Q2KHR3	Glutamine and serine-rich protein 1 OS=Homo sapiens GN=QSER1 PE=1 SV=3 - [QSER1_HUMAN]	0,52	1	1	2	Extracellular
P06737	Glycogen phosphorylase, liver form OS=Homo sapiens GN=PYGL PE=1 SV=4 - [PYGL_HUMAN]	2,01	1	1	2	Extracellular
Q6ISB3	Grainyhead-like protein 2 homolog OS=Homo sapiens GN=GRHL2 PE=1 SV=1 - [GRHL2_HUMAN]	2,4	1	1	2	Extracellular
Q8IWJ2	GRIP and coiled-coil domain-containing protein 2 OS=Homo sapiens GN=GCC2 PE=1 SV=4 - [GCC2	0,53	1	1	2	Extracellular
O95995	Growth arrest-specific protein 8 OS=Homo sapiens GN=GAS8 PE=1 SV=1 - [GAS8_HUMAN]	1,46	1	1	2	Extracellular
Q92794	Histone acetyltransferase KAT6A OS=Homo sapiens GN=KAT6A PE=1 SV=2 - [KAT6A_HUMAN]	0,7	1	1	2	Extracellular
O14686	Histone-lysine N-methyltransferase 2D OS=Homo sapiens GN=KMT2D PE=1 SV=2 - [KMT2D_HUMA	0,29	1	1	2	Extracellular
O96028	Histone-lysine N-methyltransferase NSD2 OS=Homo sapiens GN=WHSC1 PE=1 SV=1 - [NSD2_HUM	0,95	1	1	2	Extracellular
Q00056	Homeobox protein Hox-A4 OS=Homo sapiens GN=HOXA4 PE=2 SV=3 - [HXA4_HUMAN]	2,81	1	1	2	Extracellular
Q86YZ3	Hornerin OS=Homo sapiens GN=HRNR PE=1 SV=2 - [HORN_HUMAN]	0,84	1	1	2	Extracellular
P01611	Ig kappa chain V-I region Wes OS=Homo sapiens PE=1 SV=1 - [KV119_HUMAN]	16,67	1	1	2	Extracellular
Q8IWB1	Inositol 1,4,5-trisphosphate receptor-interacting protein OS=Homo sapiens GN=ITPRIP PE=1 SV=1 -	2,19	1	1	2	Extracellular
Q7Z2Y8	Interferon-induced very large GTPase 1 OS=Homo sapiens GN=GVINP1 PE=2 SV=2 - [GVIN1_HUMA	0,66	1	1	2	Extracellular
Q96N16	Janus kinase and microtubule-interacting protein 1 OS=Homo sapiens GN=JAKMIP1 PE=1 SV=1 - [JI	2,4	1	1	2	Extracellular
Q86VZ6	Juxtaposed with another zinc finger protein 1 OS=Homo sapiens GN=JAZF1 PE=1 SV=2 - [JAZF1_HU	9,47	1	1	2	Extracellular
Q9P2E2	Kinesin-like protein KIF17 OS=Homo sapiens GN=KIF17 PE=2 SV=3 - [KIF17_HUMAN]	1,55	1	1	2	Extracellular
Q14766	Latent-transforming growth factor beta-binding protein 1 OS=Homo sapiens GN=LTBP1 PE=1 SV=4 -	0,7	1	1	2	Extracellular
Q9NS15	Latent-transforming growth factor beta-binding protein 3 OS=Homo sapiens GN=LTBP3 PE=1 SV=4 -	1	1	1	2	Extracellular
P50458	LIM/homeobox protein Lhx2 OS=Homo sapiens GN=LHX2 PE=2 SV=2 - [LHX2_HUMAN]	2,96	1	1	2	Extracellular
P55001	Microfibrillar-associated protein 2 OS=Homo sapiens GN=MFAP2 PE=2 SV=1 - [MFAP2_HUMAN]	8,2	1	1	2	Extracellular
Q9UBU8	Mortality factor 4-like protein 1 OS=Homo sapiens GN=MORF4L1 PE=1 SV=2 - [MO4L1_HUMAN]	3,87	1	1	2	Extracellular
Q15746	Myosin light chain kinase, smooth muscle OS=Homo sapiens GN=MYLK PE=1 SV=4 - [MYLK_HUMA	1,36	1	1	2	Extracellular
Q9UHB4	NADPH-dependent diflavin oxidoreductase 1 OS=Homo sapiens GN=NDOR1 PE=1 SV=1 - [NDOR1	3,02	1	1	2	Extracellular
Q5VZE5	N-alpha-acetyltransferase 35, NatC auxiliary subunit OS=Homo sapiens GN=NAA35 PE=1 SV=1 - [NA	1,79	1	1	2	Extracellular
Q6P4R8	Nuclear factor related to kappa-B-binding protein OS=Homo sapiens GN=NFRKB PE=1 SV=2 - [NFRI	1,62	1	1	2	Extracellular
Q14980	Nuclear mitotic apparatus protein 1 OS=Homo sapiens GN=NUMA1 PE=1 SV=2 - [NUMA1_HUMAN]	0,8	1	1	2	Extracellular
P48552	Nuclear receptor-interacting protein 1 OS=Homo sapiens GN=NRIP1 PE=1 SV=2 - [NRIP1_HUMAN]	1,04	1	1	2	Extracellular
A8MXV4	Nucleoside diphosphate-linked moiety X motif 19 OS=Homo sapiens GN=NUDT19 PE=1 SV=1 - [NUI	2,93	1	1	2	Extracellular
O43913	Origin recognition complex subunit 5 OS=Homo sapiens GN=ORC5 PE=1 SV=1 - [ORC5_HUMAN]	3,91	1	1	2	Extracellular
Q8NB37	Parkinson disease 7 domain-containing protein 1 OS=Homo sapiens GN=PDDC1 PE=1 SV=1 - [PDD	10,91	1	1	2	Extracellular
Q9GZU2	Paternally-expressed gene 3 protein OS=Homo sapiens GN=PEG3 PE=1 SV=1 - [PEG3_HUMAN]	0,88	1	1	2	Extracellular
Q9BXM0	Periaxin OS=Homo sapiens GN=PRX PE=1 SV=2 - [PRAX_HUMAN]	1,03	1	1	2	Extracellular
Q8NDX1	PH and SEC7 domain-containing protein 4 OS=Homo sapiens GN=PSD4 PE=1 SV=2 - [PSD4_HUMA	2,08	1	1	2	Extracellular
P30086	Phosphatidylethanolamine-binding protein 1 OS=Homo sapiens GN=PEBP1 PE=1 SV=3 - [PEBP1_H	17,11	1	1	2	Extracellular
O00443	Phosphatidylinositol 4-phosphate 3-kinase C2 domain-containing subunit alpha OS=Homo sapiens GI	0,77	1	1	2	Extracellular
Q8WWB5	PIH1 domain-containing protein 2 OS=Homo sapiens GN=PIH1D2 PE=1 SV=1 - [PIHD2_HUMAN]	3,17	1	1	2	Extracellular
Q9HB19	Pleckstrin homology domain-containing family A member 2 OS=Homo sapiens GN=PLEKHA2 PE=1 S	1,65	1	1	2	Extracellular
Q9HAU0	Pleckstrin homology domain-containing family A member 5 OS=Homo sapiens GN=PLEKHA5 PE=1 S	1,08	1	1	2	Extracellular
Q8N945	PRELI domain-containing protein 2 OS=Homo sapiens GN=PRELID2 PE=2 SV=1 - [PRLD2_HUMAN]	6,88	1	1	2	Extracellular
Q9NY28	Probable polypeptide N-acetylgalactosaminyltransferase 8 OS=Homo sapiens GN=GALNT8 PE=2 SV	2,51	1	1	2	Extracellular
P07737	Profilin-1 OS=Homo sapiens GN=PFN1 PE=1 SV=2 - [PROF1_HUMAN]	10	1	1	2	Extracellular
Q9H939	Proline-serine-threonine phosphatase-interacting protein 2 OS=Homo sapiens GN=PSTPIP2 PE=1 SV	3,59	1	1	2	Extracellular
O43439	Protein CBFA2T2 OS=Homo sapiens GN=CBFA2T2 PE=1 SV=1 - [MTG8R_HUMAN]	4,3	1	1	2	Extracellular
Q9P219	Protein Daple OS=Homo sapiens GN=CCDC88C PE=1 SV=3 - [DAPLE_HUMAN]	0,39	1	1	2	Extracellular
Q8IYM0	Protein FAM186B OS=Homo sapiens GN=FAM186B PE=2 SV=2 - [F186B_HUMAN]	1,79	1	1	2	Extracellular
A8MYZ0	Protein FAM188B2 OS=Homo sapiens GN=FAM188B2 PE=3 SV=2 - [F1882_HUMAN]	3,61	1	1	2	Extracellular
Q8N485	Protein limb expression 1 homolog OS=Homo sapiens GN=LIX1 PE=2 SV=2 - [LIX1_HUMAN]	6,38	1	1	2	Extracellular
A8MPX8	Protein phosphatase 2C-like domain-containing protein 1 OS=Homo sapiens GN=PP2D1 PE=2 SV=2	2,38	1	1	2	Extracellular
P20848	Putative alpha-1-antitrypsin-related protein OS=Homo sapiens GN=SERPINA2 PE=1 SV=1 - [A1ATR	4,52	1	1	2	Extracellular
A6NCF6	Putative MAGE domain-containing protein MAGEA13P OS=Homo sapiens GN=MAGEA13P PE=5 SV	6,45	1	1	2	Extracellular
Q5T699	Putative uncharacterized protein C6orf183 OS=Homo sapiens GN=C6orf183 PE=5 SV=3 - [CF183_H	2,48	1	1	2	Extracellular
Q9H6N6	Putative uncharacterized protein MYH16 OS=Homo sapiens GN=MYH16 PE=1 SV=2 - [MYH16_HUM	1,82	1	1	2	Extracellular
P0CG00	Putative zinc finger and SCAN domain-containing protein 5D OS=Homo sapiens GN=ZSCAN5D PE=!	1,41	1	1	2	Extracellular
Q8TEU7	Rap guanine nucleotide exchange factor 6 OS=Homo sapiens GN=RAPGEF6 PE=1 SV=2 - [RPGF6	1	1	1	2	Extracellular
Q9HAU5	Regulator of nonsense transcripts 2 OS=Homo sapiens GN=UPF2 PE=1 SV=1 - [RENT2_HUMAN]	1,18	1	1	2	Extracellular
P12271	Retinaldehyde-binding protein 1 OS=Homo sapiens GN=RLBP1 PE=1 SV=2 - [RLBP1_HUMAN]	4,1	1	1	2	Extracellular
Q8TC12	Retinol dehydrogenase 11 OS=Homo sapiens GN=RDH11 PE=1 SV=2 - [RDH11_HUMAN]	3,46	1	1	2	Extracellular
Q53QZ3	Rho GTPase-activating protein 15 OS=Homo sapiens GN=ARHGAP15 PE=1 SV=2 - [RHG15_HUMA	1,05	1	1	2	Extracellular
Q8N392	Rho GTPase-activating protein 18 OS=Homo sapiens GN=ARHGAP18 PE=1 SV=3 - [RHG18_HUMA	2,56	1	1	2	Extracellular
Q5T5U3	Rho GTPase-activating protein 21 OS=Homo sapiens GN=ARHGAP21 PE=1 SV=1 - [RHG21_HUMA	0,92	1	1	2	Extracellular
Q9NZN5	Rho guanine nucleotide exchange factor 12 OS=Homo sapiens GN=ARHGEF12 PE=1 SV=1 - [ARHG	0,78	1	1	2	Extracellular
Q15418	Ribosomal protein S6 kinase alpha-1 OS=Homo sapiens GN=RPS6KA1 PE=1 SV=2 - [KS6A1_HUMA	1,09	1	1	2	Extracellular
Q8IXT5	RNA-binding protein 12B OS=Homo sapiens GN=RBM12B PE=1 SV=2 - [RB12B_HUMAN]	0,9	1	1	2	Extracellular
Q9NZJ4	Sacsin OS=Homo sapiens GN=SACS PE=1 SV=2 - [SACS_HUMAN]	0,13	1	1	2	Extracellular
Q96ES7	SAGA-associated factor 29 OS=Homo sapiens GN=SGF29 PE=1 SV=1 - [SGF29_HUMAN]	5,46	1	1	2	Extracellular
Q9HC62	Sentrin-specific protease 2 OS=Homo sapiens GN=SENP2 PE=1 SV=3 - [SENP2_HUMAN]	2,21	1	1	2	Extracellular
Q16181	Septin-7 OS=Homo sapiens GN=SEPT7 PE=1 SV=2 - [SEPT7_HUMAN]	2,52	1	1	2	Extracellular
P10124	Serglycin OS=Homo sapiens GN=SRGN PE=1 SV=3 - [SRGN_HUMAN]	3,16	1	1	2	Extracellular
Q9NQ38	Serine protease inhibitor Kazal-type 5 OS=Homo sapiens GN=SPINK5 PE=1 SV=2 - [ISK5_HUMAN]	1,22	1	1	2	Extracellular
O95747	Serine/threonine-protein kinase OSR1 OS=Homo sapiens GN=OXSR1 PE=1 SV=1 - [OXSR1_HUMAI	1,33	1	1	2	Extracellular
Q14140	SERTA domain-containing protein 2 OS=Homo sapiens GN=SERTAD2 PE=1 SV=1 - [SRTD2_HUMA	3,82	1	1	2	Extracellular
Q5TCZ1	SH3 and PX domain-containing protein 2A OS=Homo sapiens GN=SH3PXD2A PE=1 SV=1 - [SPD2A	0,79	1	1	2	Extracellular
Q9Y5X3	Sorting nexin-5 OS=Homo sapiens GN=SNX5 PE=1 SV=1 - [SNX5_HUMAN]	2,97	1	1	2	Extracellular
Q6Q759	Sperm-associated antigen 17 OS=Homo sapiens GN=SPAG17 PE=2 SV=1 - [SPG17_HUMAN]	0,54	1	1	2	Extracellular
Q9UM82	Spermatogenesis-associated protein 2 OS=Homo sapiens GN=SPATA2 PE=1 SV=2 - [SPAT2_HUMA	2,31	1	1	2	Extracellular
P38646	Stress-70 protein, mitochondrial OS=Homo sapiens GN=HSPA9 PE=1 SV=2 - [GRP75_HUMAN]	2,5	1	1	2	Extracellular
Q8IY18	Structural maintenance of chromosomes protein 5 OS=Homo sapiens GN=SMC5 PE=1 SV=2 - [SMC!	1	1	1	2	Extracellular
Q8IY92	Structure-specific endonuclease subunit SLX4 OS=Homo sapiens GN=SLX4 PE=1 SV=3 - [SLX4_HU	1,09	1	1	2	Extracellular
Q9ULW0	Targeting protein for Xklp2 OS=Homo sapiens GN=TPX2 PE=1 SV=2 - [TPX2_HUMAN]	2,28	1	1	2	Extracellular
Q9BYX2	TBC1 domain family member 2A OS=Homo sapiens GN=TBC1D2 PE=1 SV=3 - [TBD2A_HUMAN]	1,4	1	1	2	Extracellular
Q0IIM8	TBC1 domain family member 8B OS=Homo sapiens GN=TBC1D8B PE=1 SV=2 - [TBC8B_HUMAN]	1,79	1	1	2	Extracellular
Q6ZT07	TBC1 domain family member 9 OS=Homo sapiens GN=TBC1D9 PE=2 SV=2 - [TBCD9_HUMAN]	1,34	1	1	2	Extracellular
Q9NYB0	Telomeric repeat-binding factor 2-interacting protein 1 OS=Homo sapiens GN=TERF2IP PE=1 SV=1 -	3,51	1	1	2	Extracellular
P61812	Transforming growth factor beta-2 OS=Homo sapiens GN=TGFB2 PE=1 SV=1 - [TGFB2_HUMAN]	3,86	1	1	2	Extracellular
Q9BT92	Trichoplein keratin filament-binding protein OS=Homo sapiens GN=TCHP PE=1 SV=1 - [TCHP_HUM	2,21	1	1	2	Extracellular
B5MCY1	Tudor domain-containing protein 15 OS=Homo sapiens GN=TDRD15 PE=2 SV=1 - [TDR15_HUMAN]	0,52	1	1	2	Extracellular
Q9UIG0	Tyrosine-protein kinase BAZ1B OS=Homo sapiens GN=BAZ1B PE=1 SV=2 - [BAZ1B_HUMAN]	0,88	1	1	2	Extracellular
Q9UPU5	Ubiquitin carboxyl-terminal hydrolase 24 OS=Homo sapiens GN=USP24 PE=1 SV=3 - [UBP24_HUMA	0,57	1	1	2	Extracellular
Q9UHP3	Ubiquitin carboxyl-terminal hydrolase 25 OS=Homo sapiens GN=USP25 PE=1 SV=4 - [UBP25_HUMA	1,23	1	1	2	Extracellular
Q14376	UDP-glucose 4-epimerase OS=Homo sapiens GN=GALE PE=1 SV=2 - [GALE_HUMAN]	2,3	1	1	2	Extracellular
O60268	Uncharacterized protein KIAA0513 OS=Homo sapiens GN=KIAA0513 PE=2 SV=1 - [K0513_HUMAN]	6,08	1	1	2	Extracellular
Q6NV74	Uncharacterized protein KIAA1211-like OS=Homo sapiens GN=KIAA1211L PE=2 SV=3 - [K121L_HU	1,87	1	1	2	Extracellular
Q8IUG5	Unconventional myosin-XVIIIb OS=Homo sapiens GN=MYO18B PE=1 SV=1 - [MY18B_HUMAN]	0,7	1	1	2	Extracellular
Q8TEU8	WAP, Kazal, immunoglobulin, Kunitz and NTR domain-containing protein 2 OS=Homo sapiens GN=W	2,26	1	1	2	Extracellular
Q86UP3	Zinc finger homeobox protein 4 OS=Homo sapiens GN=ZFHX4 PE=1 SV=1 - [ZFHX4_HUMAN]	0,36	1	1	2	Extracellular
Q9UDV7	Zinc finger protein 282 OS=Homo sapiens GN=ZNF282 PE=2 SV=3 - [ZN282_HUMAN]	1,34	1	1	2	Extracellular
Q96ME7	Zinc finger protein 512 OS=Homo sapiens GN=ZNF512 PE=1 SV=2 - [ZN512_HUMAN]	1,94	1	1	2	Extracellular
A8MT19	Putative rhophilin-2-like protein RHPN2P1 OS=Homo sapiens GN=RHPN2P1 PE=5 SV=2 - [RHN2P_	1,72	1	1	2	Extracellular
H7BZ55	Putative ciliary rootlet coiled-coil protein 2 OS=Homo sapiens GN=CROCC2 PE=5 SV=3 - [CRCC2_H	1,45	2	2	2	Extracellular
O14978	Zinc finger protein 263 OS=Homo sapiens GN=ZNF263 PE=1 SV=2 - [ZN263_HUMAN]	1,61	1	1	2	Extracellular
O43196	MutS protein homolog 5 OS=Homo sapiens GN=MSH5 PE=1 SV=1 - [MSH5_HUMAN]	1,56	1	1	2	Extracellular
O43776	Asparagine--tRNA ligase, cytoplasmic OS=Homo sapiens GN=NARS PE=1 SV=1 - [SYNC_HUMAN]	2,92	1	1	2	Extracellular
O60814	Histone H2B type 1-K OS=Homo sapiens GN=HIST1H2BK PE=1 SV=3 - [H2B1K_HUMAN]	7,14	1	1	2	Extracellular
P0C7I6	Coiled-coil domain-containing protein 159 OS=Homo sapiens GN=CCDC159 PE=2 SV=1 - [CC159_H	3,64	1	1	2	Extracellular
P11532	Dystrophin OS=Homo sapiens GN=DMD PE=1 SV=3 - [DMD_HUMAN]	0,3	1	1	2	Extracellular
P34096	Ribonuclease 4 OS=Homo sapiens GN=RNASE4 PE=1 SV=3 - [RNAS4_HUMAN]	12,24	1	1	2	Extracellular
P52737	Zinc finger protein 136 OS=Homo sapiens GN=ZNF136 PE=1 SV=1 - [ZN136_HUMAN]	3,52	1	1	2	Extracellular
P59923	Zinc finger protein 445 OS=Homo sapiens GN=ZNF445 PE=1 SV=1 - [ZN445_HUMAN]	0,87	1	1	2	Extracellular

P80404	4-aminobutyrate aminotransferase, mitochondrial OS=Homo sapiens GN=ABAT PE=1 SV=3 - [GABT_	4,2	1	1	2	Extracellular
Q15599	Na(+)/H(+) exchange regulatory cofactor NHE-RF2 OS=Homo sapiens GN=SLC9A3R2 PE=1 SV=2 -	3,86	1	1	2	Extracellular
Q2M2Z5	Centrosomal protein kizuna OS=Homo sapiens GN=KIZ PE=1 SV=2 - [KIZ_HUMAN]	0,89	1	1	2	Extracellular
Q3KP66	Uncharacterized protein C1orf106 OS=Homo sapiens GN=C1orf106 PE=2 SV=2 - [CA106_HUMAN]	1,51	1	1	2	Extracellular
Q56UN5	Mitogen-activated protein kinase kinase kinase 19 OS=Homo sapiens GN=MAP3K19 PE=2 SV=1 - [M	0,75	1	1	2	Extracellular
Q5JXB2	Putative ubiquitin-conjugating enzyme E2 N-like OS=Homo sapiens GN=UBE2NL PE=1 SV=1 - [UE2I	6,54	1	1	2	Extracellular
Q5TEA3	Uncharacterized protein C20orf194 OS=Homo sapiens GN=C20orf194 PE=1 SV=1 - [CT194_HUMAN	3,06	2	2	2	Extracellular
Q5VYM1	Uncharacterized protein C9orf131 OS=Homo sapiens GN=C9orf131 PE=2 SV=3 - [CI131_HUMAN]	1,3	1	1	2	Extracellular
Q6ZMI0	Protein phosphatase 1 regulatory subunit 21 OS=Homo sapiens GN=PPP1R21 PE=1 SV=1 - [PPR21	1,03	1	1	2	Extracellular
Q6ZSC3	RNA-binding protein 43 OS=Homo sapiens GN=RBM43 PE=2 SV=1 - [RBM43_HUMAN]	2,52	1	1	2	Extracellular
Q7Z4H7	HAUS augmin-like complex subunit 6 OS=Homo sapiens GN=HAUS6 PE=1 SV=2 - [HAUS6_HUMAN	2,41	1	1	2	Extracellular
Q7Z7B0	Filamin-A-interacting protein 1 OS=Homo sapiens GN=FILIP1 PE=1 SV=1 - [FLIP1_HUMAN]	2,06	1	1	2	Extracellular
Q8IYI6	Exocyst complex component 8 OS=Homo sapiens GN=EXOC8 PE=1 SV=2 - [EXOC8_HUMAN]	1,52	1	1	2	Extracellular
Q8IYS1	Peptidase M20 domain-containing protein 2 OS=Homo sapiens GN=PM20D2 PE=1 SV=2 - [P20D2_H	5,73	1	1	2	Extracellular
Q8NBL1	Protein O-glucosyltransferase 1 OS=Homo sapiens GN=POGLUT1 PE=1 SV=1 - [PGLT1_HUMAN]	2,55	1	1	2	Extracellular
Q8NG27	E3 ubiquitin-protein ligase Praja-1 OS=Homo sapiens GN=PJA1 PE=1 SV=2 - [PJA1_HUMAN]	2,64	1	1	2	Extracellular
Q8TAW3	Zinc finger protein 671 OS=Homo sapiens GN=ZNF671 PE=2 SV=2 - [ZN671_HUMAN]	2,43	1	1	2	Extracellular
Q8WX94	NACHT, LRR and PYD domains-containing protein 7 OS=Homo sapiens GN=NLRP7 PE=1 SV=1 - [N	1,53	1	1	2	Extracellular
Q92485	Acid sphingomyelinase-like phosphodiesterase 3b OS=Homo sapiens GN=SMPDL3B PE=2 SV=2 - [A	2,2	1	1	2	Extracellular
Q92608	Dedicator of cytokinesis protein 2 OS=Homo sapiens GN=DOCK2 PE=1 SV=2 - [DOCK2_HUMAN]	1,37	1	2	2	Extracellular
Q92954	Proteoglycan 4 OS=Homo sapiens GN=PRG4 PE=1 SV=2 - [PRG4_HUMAN]	1,57	2	2	2	Extracellular
Q93008	Probable ubiquitin carboxyl-terminal hydrolase FAF-X OS=Homo sapiens GN=USP9X PE=1 SV=3 - [U	1,21	1	2	2	Extracellular
Q96EA4	Protein Spindly OS=Homo sapiens GN=SPDL1 PE=1 SV=2 - [SPDLY_HUMAN]	4,13	2	2	2	Extracellular
Q96M34	Uncharacterized protein C3orf30 OS=Homo sapiens GN=C3orf30 PE=2 SV=2 - [CC030_HUMAN]	2,61	1	1	2	Extracellular
Q9BTY2	Plasma alpha-L-fucosidase OS=Homo sapiens GN=FUCA2 PE=1 SV=2 - [FUCO2_HUMAN]	4,5	2	2	2	Extracellular
Q9BYG8	Gasdermin-C OS=Homo sapiens GN=GSDMC PE=2 SV=3 - [GSDMC_HUMAN]	2,56	1	1	2	Extracellular
Q9GZM7	Tubulointerstitial nephritis antigen-like OS=Homo sapiens GN=TINAGL1 PE=1 SV=1 - [TINAL_HUMA	8,57	2	2	2	Extracellular
Q9HA65	TBC1 domain family member 17 OS=Homo sapiens GN=TBC1D17 PE=1 SV=2 - [TBC17_HUMAN]	1,39	1	1	2	Extracellular
Q9NX20	39S ribosomal protein L16, mitochondrial OS=Homo sapiens GN=MRPL16 PE=1 SV=1 - [RM16_HUM	3,98	1	1	2	Extracellular
Q9UH77	Kelch-like protein 3 OS=Homo sapiens GN=KLHL3 PE=1 SV=2 - [KLHL3_HUMAN]	1,87	1	1	2	Extracellular
Q9UHQ1	Nuclear prelamin A recognition factor OS=Homo sapiens GN=NARF PE=1 SV=1 - [NARF_HUMAN]	3,95	1	1	2	Extracellular
Q9UJ41	Rab5 GDP/GTP exchange factor OS=Homo sapiens GN=RABGEF1 PE=1 SV=2 - [RABX5_HUMAN]	2,68	1	1	2	Extracellular
Q9Y2G9	Protein strawberry notch homolog 2 OS=Homo sapiens GN=SBNO2 PE=2 SV=3 - [SBNO2_HUMAN]	0,59	1	1	2	Extracellular
Q9Y600	Cysteine sulfinic acid decarboxylase OS=Homo sapiens GN=CSAD PE=1 SV=2 - [CSAD_HUMAN]	1,62	1	1	2	Extracellular
P06889	Ig lambda chain V-IV region MOL OS=Homo sapiens PE=1 SV=1 - [LV405_HUMAN]	23,58	1	1	2	not_matched
P08195	4F2 cell-surface antigen heavy chain OS=Homo sapiens GN=SLC3A2 PE=1 SV=3 - [4F2_HUMAN]	3,02	1	1	2	Transmembrane
Q32M45	Anoctamin-4 OS=Homo sapiens GN=ANO4 PE=2 SV=1 - [ANO4_HUMAN]	0,84	1	1	2	Transmembrane
Q9NWR8	Calcium uniporter regulatory subunit MCUb, mitochondrial OS=Homo sapiens GN=CCDC109B PE=1	5,36	1	1	2	Transmembrane
Q9P1Z9	Coiled-coil domain-containing protein 180 OS=Homo sapiens GN=CCDC180 PE=2 SV=2 - [CC180_H	1,82	2	2	2	Transmembrane
Q9Y672	Dolichyl pyrophosphate Man9GlcNAc2 alpha-1,3-glucosyltransferase OS=Homo sapiens GN=ALG6 P	2,56	1	1	2	Transmembrane
P53804	E3 ubiquitin-protein ligase TTC3 OS=Homo sapiens GN=TTC3 PE=1 SV=2 - [TTC3_HUMAN]	0,74	1	1	2	Transmembrane
P11488	Guanine nucleotide-binding protein G(t) subunit alpha-1 OS=Homo sapiens GN=GNAT1 PE=1 SV=5 -	4,29	1	1	2	Transmembrane
P50851	Lipopolysaccharide-responsive and beige-like anchor protein OS=Homo sapiens GN=LRBA PE=1 SV	0,45	1	1	2	Transmembrane
Q8NFT2	Metalloreductase STEAP2 OS=Homo sapiens GN=STEAP2 PE=1 SV=3 - [STEA2_HUMAN]	6,33	2	2	2	Transmembrane
Q9C000	NACHT, LRR and PYD domains-containing protein 1 OS=Homo sapiens GN=NLRP1 PE=1 SV=1 - [N	0,75	1	1	2	Transmembrane
O43157	Plexin-B1 OS=Homo sapiens GN=PLXNB1 PE=1 SV=3 - [PLXB1_HUMAN]	0,28	1	1	2	Transmembrane
Q07954	Prolow-density lipoprotein receptor-related protein 1 OS=Homo sapiens GN=LRP1 PE=1 SV=2 - [LRP	0,22	1	1	2	Transmembrane
Q96N87	Sodium-dependent neutral amino acid transporter B(0)AT3 OS=Homo sapiens GN=SLC6A18 PE=2 S	1,59	1	1	2	Transmembrane
Q9Y5W7	Sorting nexin-14 OS=Homo sapiens GN=SNX14 PE=1 SV=3 - [SNX14_HUMAN]	1,16	1	1	2	Transmembrane
O15455	Toll-like receptor 3 OS=Homo sapiens GN=TLR3 PE=1 SV=1 - [TLR3_HUMAN]	1,88	1	1	2	Transmembrane
Q5VU97	VWFA and cache domain-containing protein 1 OS=Homo sapiens GN=CACHD1 PE=2 SV=2 - [CAHD	1,1	1	1	2	Transmembrane
O60503	Adenylate cyclase type 9 OS=Homo sapiens GN=ADCY9 PE=1 SV=4 - [ADCY9_HUMAN]	0,96	1	1	2	Transmembrane
Q8WWZ7	ATP-binding cassette sub-family A member 5 OS=Homo sapiens GN=ABCA5 PE=2 SV=2 - [ABCA5_	0,43	1	1	2	Transmembrane
Q8N1M1	Bestrophin-3 OS=Homo sapiens GN=BEST3 PE=2 SV=1 - [BEST3_HUMAN]	2,84	1	1	2	Transmembrane
O60909	Beta-1,4-galactosyltransferase 2 OS=Homo sapiens GN=B4GALT2 PE=1 SV=1 - [B4GT2_HUMAN]	4,03	1	1	2	Transmembrane
Q86Z14	Beta-klotho OS=Homo sapiens GN=KLB PE=1 SV=1 - [KLOTB_HUMAN]	0,67	1	1	2	Transmembrane
P55289	Cadherin-12 OS=Homo sapiens GN=CDH12 PE=2 SV=2 - [CAD12_HUMAN]	2,02	1	1	2	Transmembrane
Q7LGC8	Carbohydrate sulfotransferase 3 OS=Homo sapiens GN=CHST3 PE=1 SV=3 - [CHST3_HUMAN]	2,71	1	1	2	Transmembrane
Q13740	CD166 antigen OS=Homo sapiens GN=ALCAM PE=1 SV=2 - [CD166_HUMAN]	1,37	1	1	2	Transmembrane
Q96N23	Cilia- and flagella-associated protein 54 OS=Homo sapiens GN=CFAP54 PE=2 SV=3 - [CFA54_HUM	0,52	1	1	2	Transmembrane
Q8N1I0	Dedicator of cytokinesis protein 4 OS=Homo sapiens GN=DOCK4 PE=1 SV=3 - [DOCK4_HUMAN]	0,81	1	1	2	Transmembrane
Q02413	Desmoglein-1 OS=Homo sapiens GN=DSG1 PE=1 SV=2 - [DSG1_HUMAN]	1,53	1	1	2	Transmembrane
P54756	Ephrin type-A receptor 5 OS=Homo sapiens GN=EPHA5 PE=1 SV=3 - [EPHA5_HUMAN]	2,31	1	1	2	Transmembrane
A0FGR9	Extended synaptotagmin-3 OS=Homo sapiens GN=ESYT3 PE=1 SV=1 - [ESYT3_HUMAN]	2,37	1	1	2	Transmembrane
A0AVI2	Fer-1-like protein 5 OS=Homo sapiens GN=FER1L5 PE=2 SV=2 - [FR1L5_HUMAN]	0,48	1	1	2	Transmembrane
Q2WGJ9	Fer-1-like protein 6 OS=Homo sapiens GN=FER1L6 PE=2 SV=2 - [FR1L6_HUMAN]	0,54	1	1	2	Transmembrane
P17302	Gap junction alpha-1 protein OS=Homo sapiens GN=GJA1 PE=1 SV=2 - [CXA1_HUMAN]	5,24	1	1	2	Transmembrane
P42262	Glutamate receptor 2 OS=Homo sapiens GN=GRIA2 PE=1 SV=3 - [GRIA2_HUMAN]	1,59	1	1	2	Transmembrane
P12314	High affinity immunoglobulin gamma Fc receptor I OS=Homo sapiens GN=FCGR1A PE=1 SV=2 - [FC	3,74	1	1	2	Transmembrane
Q14571	Inositol 1,4,5-trisphosphate receptor type 2 OS=Homo sapiens GN=ITPR2 PE=1 SV=2 - [ITPR2_HUM	0,26	1	1	2	Transmembrane
P26006	Integrin alpha-3 OS=Homo sapiens GN=ITGA3 PE=1 SV=5 - [ITA3_HUMAN]	1,43	1	1	2	Transmembrane
Q86UP2	Kinectin OS=Homo sapiens GN=KTN1 PE=1 SV=1 - [KTN1_HUMAN]	1,18	1	1	2	Transmembrane
P0C6S8	Leucine-rich repeat and immunoglobulin-like domain-containing nogo receptor-interacting protein 3 OS	4,56	1	1	2	Transmembrane
Q6UXK5	Leucine-rich repeat neuronal protein 1 OS=Homo sapiens GN=LRRN1 PE=1 SV=1 - [LRRN1_HUMAN	0,98	1	1	2	Transmembrane
Q7Z3B1	Neuronal growth regulator 1 OS=Homo sapiens GN=NEGR1 PE=1 SV=3 - [NEGR1_HUMAN]	3,67	1	1	2	Transmembrane
P26717	NKG2-C type II integral Transmembrane protein OS=Homo sapiens GN=KLRC2 PE=1 SV=2 - [NKG2	6,06	1	1	2	Transmembrane
Q8NGQ3	Olfactory receptor 1S2 OS=Homo sapiens GN=OR1S2 PE=3 SV=2 - [OR1S2_HUMAN]	6,46	1	1	2	Transmembrane
Q01814	Plasma Transmembrane calcium-transporting ATPase 2 OS=Homo sapiens GN=ATP2B2 PE=1 SV=2	0,88	1	1	2	Transmembrane
P16284	Platelet endothelial cell adhesion molecule OS=Homo sapiens GN=PECAM1 PE=1 SV=1 - [PECA1_H	2,85	1	1	2	Transmembrane
P98161	Polycystin-1 OS=Homo sapiens GN=PKD1 PE=1 SV=3 - [PKD1_HUMAN]	0,44	1	1	2	Transmembrane
P43115	Prostaglandin E2 receptor EP3 subtype OS=Homo sapiens GN=PTGER3 PE=2 SV=1 - [PE2R3_HUM	3,33	1	1	2	Transmembrane
Q5JWR5	Protein dopey-1 OS=Homo sapiens GN=DOPEY1 PE=2 SV=1 - [DOP1_HUMAN]	0,45	1	1	2	Transmembrane
Q9Y5E7	Protocadherin beta-2 OS=Homo sapiens GN=PCDHB2 PE=1 SV=1 - [PCDB2_HUMAN]	2,13	1	1	2	Transmembrane
P07949	Proto-oncogene tyrosine-protein kinase receptor Ret OS=Homo sapiens GN=RET PE=1 SV=3 - [RET	1,53	1	1	2	Transmembrane
Q9BQQ7	Receptor-transporting protein 3 OS=Homo sapiens GN=RTP3 PE=1 SV=1 - [RTP3_HUMAN]	4,74	1	1	2	Transmembrane
P10586	Receptor-type tyrosine-protein phosphatase F OS=Homo sapiens GN=PTPRF PE=1 SV=2 - [PTPRF_	0,52	1	1	2	Transmembrane
Q96LZ7	Regulator of microtubule dynamics protein 2 OS=Homo sapiens GN=RMDN2 PE=1 SV=2 - [RMD2_H	2,44	1	1	2	Transmembrane
Q96MT1	RING finger protein 145 OS=Homo sapiens GN=RNF145 PE=2 SV=2 - [RN145_HUMAN]	2,71	1	1	2	Transmembrane
Q15413	Ryanodine receptor 3 OS=Homo sapiens GN=RYR3 PE=1 SV=3 - [RYR3_HUMAN]	0,27	1	1	2	Transmembrane
P46721	Solute carrier organic anion transporter family member 1A2 OS=Homo sapiens GN=SLCO1A2 PE=2 S	2,54	1	1	2	Transmembrane
Q9HBV2	Sperm acrosome Transmembrane-associated protein 1 OS=Homo sapiens GN=SPACA1 PE=1 SV=1	2,38	1	1	2	Transmembrane
Q9NYW0	Taste receptor type 2 member 10 OS=Homo sapiens GN=TAS2R10 PE=1 SV=3 - [T2R10_HUMAN]	2,93	1	1	2	Transmembrane
Q8IUR5	Transmembrane and TPR repeat-containing protein 1 OS=Homo sapiens GN=TMTC1 PE=1 SV=3 - [T	1,81	1	1	2	Transmembrane
P14679	Tyrosinase OS=Homo sapiens GN=TYR PE=1 SV=3 - [TYRO_HUMAN]	2,27	1	1	2	Transmembrane
P19224	UDP-glucuronosyltransferase 1-6 OS=Homo sapiens GN=UGT1A6 PE=1 SV=2 - [UD16_HUMAN]	2,26	1	1	2	Transmembrane
Q9NQX4	Unconventional myosin-Vc OS=Homo sapiens GN=MYO5C PE=1 SV=2 - [MYO5C_HUMAN]	0,57	1	1	2	Transmembrane
O15056	Synaptojanin-2 OS=Homo sapiens GN=SYNJ2 PE=1 SV=3 - [SYNJ2_HUMAN]	1,07	1	1	2	Transmembrane
Q7Z7M0	Multiple epidermal growth factor-like domains protein 8 OS=Homo sapiens GN=MEGF8 PE=1 SV=2 -	0,56	1	1	2	Transmembrane
Q8N0W4	Neuroligin-4, X-linked OS=Homo sapiens GN=NLGN4X PE=1 SV=1 - [NLGNX_HUMAN]	1,59	1	1	2	Transmembrane
Q9BPW4	Apolipoprotein L4 OS=Homo sapiens GN=APOL4 PE=2 SV=3 - [APOL4_HUMAN]	5,98	2	2	2	Transmembrane
Q9HCJ2	Leucine-rich repeat-containing protein 4C OS=Homo sapiens GN=LRRC4C PE=1 SV=1 - [LRC4C_HU	1,56	1	1	2	Transmembrane
Q9NY46	Sodium channel protein type 3 subunit alpha OS=Homo sapiens GN=SCN3A PE=1 SV=2 - [SCN3A_H	1	1	1	2	Transmembrane
Q9UNW1	Multiple inositol polyphosphate phosphatase 1 OS=Homo sapiens GN=MINPP1 PE=1 SV=1 - [MINP1	2,26	1	1	2	Transmembrane
