# Supplementary material for: Systems pathology analysis identifies neurodegenerative nature of age‐related vitreoretinal interface diseases
Source: Aging Cell. 2018 Jul 2;17(5):e12809. doi: 10.1111/acel.12809 (PMC6156470; doi:10.1111/acel.12809)
Supplement: Supplementary file 7 [file ACEL-17-e12809-s007.pdf]

**Supplemental Table S4.** Average and fold differences of normalized MS1 intensities of the 934 quantified proteins. Statistical significant test of abundance changes between iERM, MH and DME eyes were conducted using Student's t-test. FC = fold differences.

| | | Average MS1 Intensities | | | iERM vs DME | | MH vs DME | | iERM vs MH | |
|---|---|---|---|---|---|---|---|---|---|---|
| **Accession** | **Description** | **iERM** | **MH** | **DME** | **FC** | **q-value** | **FC** | **q-value** | **FC** | **q-value** |
| P62258 | 14-3-3 protein epsilon OS=Homo sapiens GN=YWHAE PE=1 SV=1 - [1433E_HUMAN] | 274545 | 191784 | 480908 | 0,57 | 0,291274 | 0,40 | 0,064021 | 1,43 | 0,617431 |
| P51178 | 1-phosphatidylinositol 4,5-bisphosphate phosphodiesterase delta-1 OS=Homo sapiens GN=PL( | 866707 | 539368 | 729664 | 1,19 | 0,530083 | 0,74 | 0,089425 | 1,61 | 0,010923 |
| Q9P212 | 1-phosphatidylinositol 4,5-bisphosphate phosphodiesterase epsilon-1 OS=Homo sapiens GN=F | 5575551 | 7291016 | 3065990 | 1,82 | 0,055949 | 2,38 | 2,29E-06 | 0,76 | 0,036472 |
| Q9ULD0 | 2-oxoglutarate dehydrogenase-like, mitochondrial OS=Homo sapiens GN=OGDHL PE=1 SV=3 - | 6240174 | 6971886 | 3143241 | 1,99 | 0,131484 | 2,22 | 0,040467 | 0,90 | 0,720548 |
| Q13405 | 39S ribosomal protein L49, mitochondrial OS=Homo sapiens GN=MRPL49 PE=1 SV=1 - [RM49_ | 60826 | 120258 | 2317 | 26,25 | 0,216855 | 51,89 | 0,063632 | 0,51 | 0,231699 |
| Q99714 | 3-hydroxyacyl-CoA dehydrogenase type-2 OS=Homo sapiens GN=HSD17B10 PE=1 SV=3 - [HCD2 | 277329 | 391859 | 356297 | 0,78 | 0,620004 | 1,10 | 0,82175 | 0,71 | 0,359249 |
| Q9BRK5 | 45 kDa calcium-binding protein OS=Homo sapiens GN=SDF4 PE=1 SV=1 - [CAB45_HUMAN] | 168492 | 144190 | 34342 | 4,91 | 0,137946 | 4,20 | 0,093594 | 1,17 | 0,776033 |
| P08195 | 4F2 cell-surface antigen heavy chain OS=Homo sapiens GN=SLC3A2 PE=1 SV=3 - [4F2_HUMAN] | 178765 | 315296 | 463984 | 0,39 | 0,167537 | 0,68 | 0,418775 | 0,57 | 0,386254 |
| P17643 | 5,6-dihydroxyindole-2-carboxylic acid oxidase OS=Homo sapiens GN=TYRP1 PE=1 SV=2 - [TYRP | 28857 | 44778 | 4427 | 6,52 | 0,22756 | 10,11 | 0,093594 | 0,64 | 0,445197 |
| Q9UGJ0 | 5'-AMP-activated protein kinase subunit gamma-2 OS=Homo sapiens GN=PRKAG2 PE=1 SV=1 - | 13243 | 2660 | 48010 | 0,28 | 0,013791 | 0,06 | 0,000441 | 4,98 | 0,051539 |
| P84098 | 60S ribosomal protein L19 OS=Homo sapiens GN=RPL19 PE=1 SV=1 - [RL19_HUMAN] | 44608 | 86427 | 0 | #DIV/0! | 0,379484 | #DIV/0! | 0,116628 | 0,52 | 0,404952 |
| P62829 | 60S ribosomal protein L23 OS=Homo sapiens GN=RPL23 PE=1 SV=1 - [RL23_HUMAN] | 928532 | 340907 | 167372 | 5,55 | 0,054873 | 2,04 | 0,079071 | 2,72 | 0,012138 |
| Q9UNX3 | 60S ribosomal protein L26-like 1 OS=Homo sapiens GN=RPL26L1 PE=1 SV=1 - [RL26L_HUMAN] | 1578664 | 710865 | 1680748 | 0,94 | 0,920667 | 0,42 | 0,105283 | 2,22 | 0,20932 |
| P08253 | 72 kDa type IV collagenase OS=Homo sapiens GN=MMP2 PE=1 SV=2 - [MMP2_HUMAN] | 694645 | 805696 | 507034 | 1,37 | 0,453877 | 1,59 | 0,175955 | 0,86 | 0,631571 |
| O60733 | 85/88 kDa calcium-independent phospholipase A2 OS=Homo sapiens GN=PLA2G6 PE=1 SV=2 - | 44512 | 111940 | 194889 | 0,23 | 0,00145 | 0,57 | 0,117106 | 0,40 | 0,041687 |
| Q9UHI8 | A disintegrin and metalloproteinase with thrombospondin motifs 1 OS=Homo sapiens GN=AD/ | 45514 | 19512 | 25933 | 1,76 | 0,698175 | 0,75 | 0,72299 | 2,33 | 0,457069 |
| Q8TE59 | A disintegrin and metalloproteinase with thrombospondin motifs 19 OS=Homo sapiens GN=AD | 645169 | 846161 | 955768 | 0,68 | 0,698175 | 0,89 | 0,76948 | 0,76 | 0,761798 |
| Q8IZT6 | Abnormal spindle-like microcephaly-associated protein OS=Homo sapiens GN=ASPM PE=1 SV= | 1576815960 | 1580057027 | 1643415208 | 0,96 | 0,894535 | 0,96 | 0,868991 | 1,00 | 0,997748 |
| A1L0T0 | Acetolactate synthase-like protein OS=Homo sapiens GN=ILVBL PE=1 SV=2 - [ILVBL_HUMAN] | 1050778 | 821188 | 4291438 | 0,24 | 7,7E-05 | 0,19 | 7,72E-06 | 1,28 | 0,582136 |
| O00763 | Acetyl-CoA carboxylase 2 OS=Homo sapiens GN=ACACB PE=1 SV=3 - [ACACB_HUMAN] | 1437135 | 949651 | 157553 | 9,12 | 0,088325 | 6,03 | 0,03319 | 1,51 | 0,384524 |
| Q13510 | Acid ceramidase OS=Homo sapiens GN=ASAH1 PE=1 SV=5 - [ASAH1_HUMAN] | 332392 | 356654 | 194703 | 1,71 | 0,268137 | 1,83 | 0,072074 | 0,93 | 0,844452 |
| Q32M88 | Acid trehalase-like protein 1 OS=Homo sapiens GN=ATHL1 PE=1 SV=2 - [ATHL1_HUMAN] | 2638222 | 2810395 | 1697170 | 1,55 | 0,131484 | 1,66 | 0,086159 | 0,94 | 0,802075 |
| P62736 | Actin, aortic smooth muscle OS=Homo sapiens GN=ACTA2 PE=1 SV=1 - [ACTA_HUMAN] | 691018 | 337722 | 1414754 | 0,49 | 0,101945 | 0,24 | 0,011607 | 2,05 | 0,039363 |
| P60709 | Actin, cytoplasmic 1 OS=Homo sapiens GN=ACTB PE=1 SV=1 - [ACTB_HUMAN] | 331892 | 119430 | 838424 | 0,40 | 0,092778 | 0,14 | 0,003072 | 2,78 | 0,143293 |
| Q9Y615 | Actin-like protein 7A OS=Homo sapiens GN=ACTL7A PE=1 SV=1 - [ACL7A_HUMAN] | 28549 | 160579 | 49779 | 0,57 | 0,535422 | 3,23 | 0,552516 | 0,18 | 0,253714 |
| P61160 | Actin-related protein 2 OS=Homo sapiens GN=ACTR2 PE=1 SV=1 - [ARP2_HUMAN] | 27525386 | 33896755 | 105460593 | 0,26 | 0,019516 | 0,32 | 0,007389 | 0,81 | 0,824748 |
| Q13705 | Activin receptor type-2B OS=Homo sapiens GN=ACVR2B PE=1 SV=3 - [AVR2B_HUMAN] | 20424 | 35808 | 586 | 34,84 | 0,34549 | 61,09 | 0,216996 | 0,57 | 0,537618 |
| Q8WXI4 | Acyl-coenzyme A thioesterase 11 OS=Homo sapiens GN=ACOT11 PE=1 SV=1 - [ACO11_HUMAN | 1194094 | 3251295 | 7656737 | 0,16 | 1,99E-07 | 0,42 | 0,0703 | 0,37 | 0,193743 |
| P82987 | ADAMTS-like protein 3 OS=Homo sapiens GN=ADAMTSL3 PE=1 SV=4 - [ATL3_HUMAN] | 451653 | 432365 | 862354 | 0,52 | 0,045633 | 0,50 | 0,061407 | 1,04 | 0,943041 |
| P46108 | Adapter molecule crk OS=Homo sapiens GN=CRK PE=1 SV=2 - [CRK_HUMAN] | 4579245 | 4919238 | 1577169 | 2,90 | 0,000676 | 3,12 | 1,76E-05 | 0,93 | 0,663451 |
| Q8NFM4 | Adenylate cyclase type 4 OS=Homo sapiens GN=ADCY4 PE=1 SV=1 - [ADCY4_HUMAN] | 487825 | 789490 | 607419 | 0,80 | 0,797824 | 1,30 | 0,65444 | 0,62 | 0,391571 |
| O43306 | Adenylate cyclase type 6 OS=Homo sapiens GN=ADCY6 PE=1 SV=2 - [ADCY6_HUMAN] | 213099 | 1136476 | 61303 | 3,48 | 0,298847 | 18,54 | 0,028668 | 0,19 | 0,000907 |
| O60503 | Adenylate cyclase type 9 OS=Homo sapiens GN=ADCY9 PE=1 SV=4 - [ADCY9_HUMAN] | 1115442 | 1080514 | 2050152 | 0,54 | 0,16029 | 0,53 | 0,119323 | 1,03 | 0,956639 |
| Q8N6H7 | ADP-ribosylation factor GTPase-activating protein 2 OS=Homo sapiens GN=ARFGAP2 PE=1 SV= | 3079868 | 4030115 | 3900426 | 0,79 | 0,392996 | 1,03 | 0,889766 | 0,76 | 0,225987 |
| Q9Y6U3 | Adseverin OS=Homo sapiens GN=SCIN PE=1 SV=4 - [ADSV_HUMAN] | 15170 | 10 | 12 | 1289,40 | 0,691802 | 0,86 | 0,92741 | 1491,03 | 0,58392 |
| P43652 | Afamin OS=Homo sapiens GN=AFM PE=1 SV=1 - [AFAM_HUMAN] | 20939258 | 21182245 | 18632134 | 1,12 | 0,634554 | 1,14 | 0,604335 | 0,99 | 0,963841 |
| O00468 | Agrin OS=Homo sapiens GN=AGRN PE=1 SV=5 - [AGRIN_HUMAN] | 712537 | 937632 | 710597 | 1,00 | 0,992912 | 1,32 | 0,306893 | 0,76 | 0,233518 |
| Q99996 | A-kinase anchor protein 9 OS=Homo sapiens GN=AKAP9 PE=1 SV=3 - [AKAP9_HUMAN] | 10826785 | 2123410 | 1258187 | 8,61 | 0,083231 | 1,69 | 0,263242 | 5,10 | 0,006998 |
| Q6NUM9 | All-trans-retinol 13,14-reductase OS=Homo sapiens GN=RETSAT PE=1 SV=2 - [RETST_HUMAN] | 8044213 | 7838640 | 3421734 | 2,35 | 0,092616 | 2,29 | 0,037041 | 1,03 | 0,944533 |
| Q9UBM8 | Alpha-1,3-mannosyl-glycoprotein 4-beta-N-acetylglucosaminyltransferase C OS=Homo sapiens | 21850 | 77908 | 29157 | 0,75 | 0,698175 | 2,67 | 0,036969 | 0,28 | 0,000701 |
| P02763 | Alpha-1-acid glycoprotein 1 OS=Homo sapiens GN=ORM1 PE=1 SV=1 - [A1AG1_HUMAN] | 46101684 | 96112925 | 114646239 | 0,40 | 0,079371 | 0,84 | 0,656309 | 0,48 | 0,078257 |
| P19652 | Alpha-1-acid glycoprotein 2 OS=Homo sapiens GN=ORM2 PE=1 SV=2 - [A1AG2_HUMAN] | 54880008 | 94337869 | 133762700 | 0,41 | 0,014705 | 0,71 | 0,170789 | 0,58 | 0,115701 |
| P01011 | Alpha-1-antichymotrypsin OS=Homo sapiens GN=SERPINA3 PE=1 SV=2 - [AACT_HUMAN] | 44225095 | 44235192 | 55148237 | 0,80 | 0,189854 | 0,80 | 0,099039 | 1,00 | 0,998213 |
| P01009 | Alpha-1-antitrypsin OS=Homo sapiens GN=SERPINA1 PE=1 SV=3 - [A1AT_HUMAN] | 28828897 | 28216702 | 24830313 | 1,16 | 0,319084 | 1,14 | 0,392046 | 1,02 | 0,899768 |
| P04217 | Alpha-1B-glycoprotein OS=Homo sapiens GN=A1BG PE=1 SV=4 - [A1BG_HUMAN] | 66554368 | 66468450 | 97959998 | 0,68 | 0,059644 | 0,68 | 0,12867 | 1,00 | 0,997748 |
| Q13424 | Alpha-1-syntrophin OS=Homo sapiens GN=SNTA1 PE=1 SV=1 - [SNTA1_HUMAN] | 1044846 | 730455 | 520961 | 2,01 | 0,398143 | 1,40 | 0,590551 | 1,43 | 0,538047 |
| P08697 | Alpha-2-antiplasmin OS=Homo sapiens GN=SERPINF2 PE=1 SV=3 - [A2AP_HUMAN] | 8536413 | 6302824 | 9264868 | 0,92 | 0,740508 | 0,68 | 0,072238 | 1,35 | 0,186937 |
| P02765 | Alpha-2-HS-glycoprotein OS=Homo sapiens GN=AHSG PE=1 SV=1 - [FETUA_HUMAN] | 138444305 | 118005366 | 231591614 | 0,60 | 0,205111 | 0,51 | 0,020763 | 1,17 | 0,760808 |
| P01023 | Alpha-2-macroglobulin OS=Homo sapiens GN=A2M PE=1 SV=3 - [A2MG_HUMAN] | 121705266 | 147648177 | 125360579 | 0,97 | 0,932619 | 1,18 | 0,424861 | 0,82 | 0,405173 |
| Q9C0B1 | Alpha-ketoglutarate-dependent dioxygenase FTO OS=Homo sapiens GN=FTO PE=1 SV=3 - [FTO | 5222953 | 4817730 | 13021370 | 0,40 | 0,004268 | 0,37 | 0,022915 | 1,08 | 0,907395 |
| Q16706 | Alpha-mannosidase 2 OS=Homo sapiens GN=MAN2A1 PE=1 SV=2 - [MA2A1_HUMAN] | 5127108 | 5363524 | 1563133 | 3,28 | 0,028644 | 3,43 | 0,005695 | 0,96 | 0,894651 |
| P17050 | Alpha-N-acetylgalactosaminidase OS=Homo sapiens GN=NAGA PE=1 SV=2 - [NAGAB_HUMAN] | 23985 | 62147 | 25645 | 0,94 | 0,962372 | 2,42 | 0,180289 | 0,39 | 0,111204 |
| Q8TCU4 | Alstrom syndrome protein 1 OS=Homo sapiens GN=ALMS1 PE=1 SV=3 - [ALMS1_HUMAN] | 12140544 | 35070875 | 45510096 | 0,27 | 0,001396 | 0,77 | 0,293261 | 0,35 | 0,004675 |
| P05067 | Amyloid beta A4 protein OS=Homo sapiens GN=APP PE=1 SV=3 - [A4_HUMAN] | 57851109 | 54343863 | 42696786 | 1,35 | 0,304542 | 1,27 | 0,272794 | 1,06 | 0,779986 |
| P51693 | Amyloid-like protein 1 OS=Homo sapiens GN=APLP1 PE=1 SV=3 - [APLP1_HUMAN] | 6082322 | 5302804 | 1574277 | 3,86 | 0,018316 | 3,37 | 0,006292 | 1,15 | 0,631633 |
| Q06481 | Amyloid-like protein 2 OS=Homo sapiens GN=APLP2 PE=1 SV=2 - [APLP2_HUMAN] | 40815419 | 41861022 | 14213584 | 2,87 | 0,050096 | 2,95 | 0,003151 | 0,98 | 0,943871 |
| Q9UJX3 | Anaphase-promoting complex subunit 7 OS=Homo sapiens GN=ANAPC7 PE=1 SV=4 - [APC7_HU | 1384986 | 1155703 | 2644451 | 0,52 | 0,013948 | 0,44 | 0,002083 | 1,20 | 0,599238 |
| P03950 | Angiogenin OS=Homo sapiens GN=ANG PE=1 SV=1 - [ANGI_HUMAN] | 15172 | 13438 | 10201 | 1,49 | 0,698175 | 1,32 | 0,710734 | 1,13 | 0,883437 |
| Q02763 | Angiopoietin-1 receptor OS=Homo sapiens GN=TEK PE=1 SV=2 - [TIE2_HUMAN] | 150345 | 235710 | 127158 | 1,18 | 0,872085 | 1,85 | 0,112223 | 0,64 | 0,418681 |
| P01019 | Angiotensinogen OS=Homo sapiens GN=AGT PE=1 SV=1 - [ANGT_HUMAN] | 33111710 | 36943662 | 44319538 | 0,75 | 0,096548 | 0,83 | 0,437562 | 0,90 | 0,632359 |
| Q9P2G1 | Ankyrin repeat and IBR domain-containing protein 1 OS=Homo sapiens GN=ANKIB1 PE=1 SV=3 | 77470 | 7601 | 42 | 1840,61 | 0,39193 | 180,58 | 0,113598 | 10,19 | 0,233518 |
| Q9Y574 | Ankyrin repeat and SOCS box protein 4 OS=Homo sapiens GN=ASB4 PE=2 SV=1 - [ASB4_HUMA | 5074554 | 5858122 | 4629390 | 1,10 | 0,755336 | 1,27 | 0,241351 | 0,87 | 0,433923 |
| Q7Z6G8 | Ankyrin repeat and sterile alpha motif domain-containing protein 1B OS=Homo sapiens GN=AI | 118677 | 186796 | 184638 | 0,64 | 0,519457 | 1,01 | 0,975379 | 0,64 | 0,393409 |
| Q8IVF6 | Ankyrin repeat domain-containing protein 18A OS=Homo sapiens GN=ANKRD18A PE=2 SV=3 - | 331102 | 308070 | 95467 | 3,47 | 0,028644 | 3,23 | 0,012937 | 1,07 | 0,827233 |
| Q9UPS8 | Ankyrin repeat domain-containing protein 26 OS=Homo sapiens GN=ANKRD26 PE=1 SV=3 - [AN | 1308605 | 2706651 | 28934735 | 0,05 | 1,12E-10 | 0,09 | 6,34E-08 | 0,48 | 0,151495 |
| Q8N2N9 | Ankyrin repeat domain-containing protein 36B OS=Homo sapiens GN=ANKRD36B PE=1 SV=4 - | 2248 | 4226 | 18779 | 0,12 | 0,22176 | 0,23 | 0,322369 | 0,53 | 0,771102 |
| Q01484 | Ankyrin-2 OS=Homo sapiens GN=ANK2 PE=1 SV=4 - [ANK2_HUMAN] | 159263502 | 138111323 | 324402896 | 0,49 | 0,001624 | 0,43 | 0,000481 | 1,15 | 0,570405 |
| P27216 | Annexin A13 OS=Homo sapiens GN=ANXA13 PE=1 SV=3 - [ANX13_HUMAN] | 3213678 | 3348249 | 2013650 | 1,60 | 0,240687 | 1,66 | 0,286793 | 0,96 | 0,922335 |
| P08133 | Annexin A6 OS=Homo sapiens GN=ANXA6 PE=1 SV=3 - [ANXA6_HUMAN] | 66358 | 60679 | 57312 | 1,16 | 0,778124 | 1,06 | 0,903143 | 1,09 | 0,824748 |
| Q32M45 | Anoctamin-4 OS=Homo sapiens GN=ANO4 PE=2 SV=1 - [ANO4_HUMAN] | 12751963 | 19082862 | 19485842 | 0,65 | 0,267338 | 0,98 | 0,955241 | 0,67 | 0,236508 |
| P01008 | Antithrombin-III OS=Homo sapiens GN=SERPINC1 PE=1 SV=1 - [ANT3_HUMAN] | 90620732 | 93957702 | 115111337 | 0,79 | 0,070648 | 0,82 | 0,068617 | 0,96 | 0,797082 |
| P56377 | AP-1 complex subunit sigma-2 OS=Homo sapiens GN=AP1S2 PE=1 SV=1 - [AP1S2_HUMAN] | 1527206 | 537599 | 2876309 | 0,53 | 0,491098 | 0,19 | 0,095963 | 2,84 | 0,431957 |
| Q13367 | AP-3 complex subunit beta-2 OS=Homo sapiens GN=AP3B2 PE=1 SV=2 - [AP3B2_HUMAN] | 82385 | 160366 | 382919 | 0,22 | 0,097431 | 0,42 | 0,255958 | 0,51 | 0,452011 |
| Q8N7J2 | APC membrane recruitment protein 2 OS=Homo sapiens GN=AMER2 PE=1 SV=3 - [AMER2_HU | 213103 | 350732 | 123005 | 1,73 | 0,472106 | 2,85 | 0,530175 | 0,61 | 0,599238 |
| P02647 | Apolipoprotein A-I OS=Homo sapiens GN=APOA1 PE=1 SV=1 - [APOA1_HUMAN] | 231098987 | 198342325 | 214340141 | 1,08 | 0,702894 | 0,93 | 0,72299 | 1,17 | 0,429824 |
| P02652 | Apolipoprotein A-II OS=Homo sapiens GN=APOA2 PE=1 SV=1 - [APOA2_HUMAN] | 156867849 | 198994855 | 185509032 | 0,85 | 0,678654 | 1,07 | 0,896954 | 0,79 | 0,631571 |
| P06727 | Apolipoprotein A-IV OS=Homo sapiens GN=APOA4 PE=1 SV=3 - [APOA4_HUMAN] | 235955420 | 245693723 | 274899803 | 0,86 | 0,223309 | 0,89 | 0,216316 | 0,96 | 0,725698 |
| P04114 | Apolipoprotein B-100 OS=Homo sapiens GN=APOB PE=1 SV=2 - [APOB_HUMAN] | 471442 | 711266 | 51953 | 9,07 | 0,096301 | 13,69 | 0,032317 | 0,66 | 0,349383 |
| P02654 | Apolipoprotein C-I OS=Homo sapiens GN=APOC1 PE=1 SV=1 - [APOC1_HUMAN] | 1091229 | 670498 | 856975 | 1,27 | 0,499419 | 0,78 | 0,423502 | 1,63 | 0,059532 |
| P02655 | Apolipoprotein C-II OS=Homo sapiens GN=APOC2 PE=1 SV=1 - [APOC2_HUMAN] | 11183 | 454 | 30 | 373,53 | 0,537743 | 15,15 | 0,567938 | 24,65 | 0,390039 |
| P02656 | Apolipoprotein C-III OS=Homo sapiens GN=APOC3 PE=1 SV=1 - [APOC3_HUMAN] | 2933713 | 2429207 | 5127257 | 0,57 | 0,209923 | 0,47 | 0,101699 | 1,21 | 0,776033 |
| P05090 | Apolipoprotein D OS=Homo sapiens GN=APOD PE=1 SV=1 - [APOD_HUMAN] | 477131 | 561351 | 1482977 | 0,32 | 0,018316 | 0,38 | 0,009101 | 0,85 | 0,770502 |
| P02649 | Apolipoprotein E OS=Homo sapiens GN=APOE PE=1 SV=1 - [APOE_HUMAN] | 104475654 | 99376722 | 48756502 | 2,14 | 0,032433 | 2,04 | 0,007978 | 1,05 | 0,835891 |
| O14791 | Apolipoprotein L1 OS=Homo sapiens GN=APOL1 PE=1 SV=5 - [APOL1_HUMAN] | 28088 | 48969 | 213738 | 0,13 | 0,007187 | 0,23 | 0,065507 | 0,57 | 0,720548 |
| O95445 | Apolipoprotein M OS=Homo sapiens GN=APOM PE=1 SV=2 - [APOM_HUMAN] | 80801 | 126124 | 151276 | 0,53 | 0,10028 | 0,83 | 0,5864 | 0,64 | 0,073055 |
| Q8WWN8 | Arf-GAP with Rho-GAP domain, ANK repeat and PH domain-containing protein 3 OS=Homo sap | 4131239 | 5474109 | 11529953 | 0,36 | 0,0009 | 0,47 | 0,059311 | 0,75 | 0,584344 |
| P51689 | Arylsulfatase D OS=Homo sapiens GN=ARSD PE=1 SV=2 - [ARSD_HUMAN] | 271807 | 392868 | 581 | 467,77 | 0,131305 | 676,12 | 0,028524 | 0,69 | 0,474166 |
| Q12797 | Aspartyl/asparaginyl beta-hydroxylase OS=Homo sapiens GN=ASPH PE=1 SV=3 - [ASPH_HUMA | 169980 | 144590 | 23057 | 7,37 | 0,053074 | 6,27 | 0,015472 | 1,18 | 0,705613 |
| Q5T6C5 | Ataxin-7-like protein 2 OS=Homo sapiens GN=ATXN7L2 PE=3 SV=1 - [AT7L2_HUMAN] | 14963295 | 41385178 | 12919801 | 1,16 | 0,832285 | 3,20 | 7,25E-07 | 0,36 | 3,33E-05 |
| P24539 | ATP synthase F(0) complex subunit B1, mitochondrial OS=Homo sapiens GN=ATP5F1 PE=1 SV= | 28313 | 16155 | 451024 | 0,06 | 0,00574 | 0,04 | 0,009294 | 1,75 | 0,703585 |
| Q6PL18 | ATPase family AAA domain-containing protein 2 OS=Homo sapiens GN=ATAD2 PE=1 SV=1 - [AT | 15997 | 33183 | 98194 | 0,16 | 0,001242 | 0,34 | 0,040137 | 0,48 | 0,326329 |
| Q96QE3 | ATPase family AAA domain-containing protein 5 OS=Homo sapiens GN=ATAD5 PE=1 SV=4 - [AT | 200579 | 121279 | 102599 | 1,95 | 0,435432 | 1,18 | 0,808974 | 1,65 | 0,377567 |

| | | | | | | | | | | |
|---|---|---|---|---|---|---|---|---|---|---|
| Q86UK0 | ATP-binding cassette sub-family A member 12 OS=Homo sapiens GN=ABCA12 PE=1 SV=3 - [AB | 2070741 | 2021925 | 5355389 | 0,39 | 2,39E-05 | 0,38 | 5,74E-05 | 1,02 | 0,9455 |
| Q8WWZ7 | ATP-binding cassette sub-family A member 5 OS=Homo sapiens GN=ABCA5 PE=2 SV=2 - [ABCA | 18495 | 11068 | 0 | #DIV/0! | 0,422344 | #DIV/0! | 0,3306 | 1,67 | 0,705613 |
| Q8NE71 | ATP-binding cassette sub-family F member 1 OS=Homo sapiens GN=ABCF1 PE=1 SV=2 - [ABCF1 | 5873295 | 13299858 | 842580 | 6,97 | 0,043919 | 15,78 | 4,94E-06 | 0,44 | 0,00013 |
| Q92499 | ATP-dependent RNA helicase DDX1 OS=Homo sapiens GN=DDX1 PE=1 SV=2 - [DDX1_HUMAN] | 46653 | 66401 | 191671 | 0,24 | 0,067848 | 0,35 | 0,151012 | 0,70 | 0,599238 |
| Q7Z478 | ATP-dependent RNA helicase DHX29 OS=Homo sapiens GN=DHX29 PE=1 SV=2 - [DHX29_HUMA | 2445714 | 348618 | 6539846 | 0,37 | 0,04883 | 0,05 | 2,75E-13 | 7,02 | 0,091425 |
| Q68CP9 | AT-rich interactive domain-containing protein 2 OS=Homo sapiens GN=ARID2 PE=1 SV=2 - [ARI | 127483 | 197600 | 10725 | 11,89 | 0,365665 | 18,42 | 0,158586 | 0,65 | 0,599238 |
| Q14865 | AT-rich interactive domain-containing protein 5B OS=Homo sapiens GN=ARID5B PE=1 SV=3 - [A | 523078 | 349954 | 513653 | 1,02 | 0,973885 | 0,68 | 0,233434 | 1,49 | 0,291309 |
| O75882 | Attractin OS=Homo sapiens GN=ATRN PE=1 SV=2 - [ATRN_HUMAN] | 42756 | 50875 | 138604 | 0,31 | 0,040345 | 0,37 | 0,006497 | 0,84 | 0,816155 |
| P98160 | Basement membrane-specific heparan sulfate proteoglycan core protein OS=Homo sapiens GN | 662600 | 663619 | 673666 | 0,98 | 0,975586 | 0,99 | 0,969013 | 1,00 | 0,997748 |
| P55061 | Bax inhibitor 1 OS=Homo sapiens GN=TMBIM6 PE=1 SV=2 - [BI1_HUMAN] | 114467 | 47747 | 21617 | 5,30 | 0,145627 | 2,21 | 0,286793 | 2,40 | 0,115701 |
| O00512 | B-cell CLL/lymphoma 9 protein OS=Homo sapiens GN=BCL9 PE=1 SV=4 - [BCL9_HUMAN] | 64989 | 87279 | 428300 | 0,15 | 8,35E-05 | 0,20 | 0,004743 | 0,74 | 0,811877 |
| Q8N1M1 | Bestrophin-3 OS=Homo sapiens GN=BEST3 PE=2 SV=1 - [BEST3_HUMAN] | 714976 | 950103 | 1768668 | 0,40 | 0,095154 | 0,54 | 0,074491 | 0,75 | 0,646184 |
| P15291 | Beta-1,4-galactosyltransferase 1 OS=Homo sapiens GN=B4GALT1 PE=1 SV=5 - [B4GT1_HUMAN | 63173 | 44038 | 11380 | 5,55 | 0,317388 | 3,87 | 0,18662 | 1,43 | 0,651307 |
| O60909 | Beta-1,4-galactosyltransferase 2 OS=Homo sapiens GN=B4GALT2 PE=1 SV=1 - [B4GT2_HUMAN | 271787746 | 232112103 | 250321377 | 1,09 | 0,664559 | 0,93 | 0,460833 | 1,17 | 0,241913 |
| O43505 | Beta-1,4-glucuronyltransferase 1 OS=Homo sapiens GN=B4GAT1 PE=1 SV=1 - [B4GA1_HUMAN | 12219966 | 10542133 | 6465638 | 1,89 | 0,067876 | 1,63 | 0,048731 | 1,16 | 0,493353 |
| P02749 | Beta-2-glycoprotein 1 OS=Homo sapiens GN=APOH PE=1 SV=3 - [APOH_HUMAN] | 38509501 | 41906736 | 92297205 | 0,42 | 8,35E-05 | 0,45 | 4,64E-06 | 0,92 | 0,765009 |
| P61769 | Beta-2-microglobulin OS=Homo sapiens GN=B2M PE=1 SV=1 - [B2MG_HUMAN] | 14139240 | 11635091 | 5890110 | 2,40 | 0,150051 | 1,98 | 0,057158 | 1,22 | 0,590084 |
| P25098 | Beta-adrenergic receptor kinase 1 OS=Homo sapiens GN=ADRBK1 PE=1 SV=2 - [ARBK1_HUMA | 48963 | 33268 | 8851 | 5,53 | 0,544514 | 3,76 | 0,058591 | 1,47 | 0,767419 |
| Q96KN2 | Beta-Ala-His dipeptidase OS=Homo sapiens GN=CNDP1 PE=1 SV=4 - [CNDP1_HUMAN] | 7625293 | 10451413 | 8841886 | 0,86 | 0,519183 | 1,18 | 0,605795 | 0,73 | 0,186937 |
| P05813 | Beta-crystallin A3 OS=Homo sapiens GN=CRYBA1 PE=1 SV=4 - [CRBA1_HUMAN] | 51599 | 22050 | 106301 | 0,49 | 0,426602 | 0,21 | 0,026572 | 2,34 | 0,54414 |
| P53673 | Beta-crystallin A4 OS=Homo sapiens GN=CRYBA4 PE=1 SV=3 - [CRBA4_HUMAN] | 13452 | 738 | 84 | 159,77 | 0,577678 | 8,76 | 0,542444 | 18,24 | 0,433973 |
| P43320 | Beta-crystallin B2 OS=Homo sapiens GN=CRYBB2 PE=1 SV=2 - [CRBB2_HUMAN] | 676528 | 150657 | 632999 | 1,07 | 0,964498 | 0,24 | 0,081583 | 4,49 | 0,345161 |
| P22914 | Beta-crystallin S OS=Homo sapiens GN=CRYGS PE=1 SV=4 - [CRBS_HUMAN] | 223175 | 357664 | 226430 | 0,99 | 0,986179 | 1,58 | 0,556315 | 0,62 | 0,414519 |
| P60022 | Beta-defensin 1 OS=Homo sapiens GN=DEFB1 PE=1 SV=1 - [DEFB1_HUMAN] | 2869 | 1113 | 26186 | 0,11 | 0,055491 | 0,04 | 0,048186 | 2,58 | 0,344625 |
| P13929 | Beta-enolase OS=Homo sapiens GN=ENO3 PE=1 SV=5 - [ENOB_HUMAN] | 248241 | 261978 | 278595 | 0,89 | 0,832028 | 0,94 | 0,886088 | 0,95 | 0,926722 |
| P06865 | Beta-hexosaminidase subunit alpha OS=Homo sapiens GN=HEXA PE=1 SV=2 - [HEXA_HUMAN] | 47186269 | 47224227 | 169785781 | 0,28 | 0,000382 | 0,28 | 0,001679 | 1,00 | 0,998213 |
| P07686 | Beta-hexosaminidase subunit beta OS=Homo sapiens GN=HEXB PE=1 SV=3 - [HEXB_HUMAN] | 163220 | 228735 | 312400 | 0,52 | 0,523743 | 0,73 | 0,740073 | 0,71 | 0,631395 |
| Q86Z14 | Beta-klotho OS=Homo sapiens GN=KLB PE=1 SV=1 - [KLOTB_HUMAN] | 661 | 297 | 12263 | 0,05 | 0,038051 | 0,02 | 0,043669 | 2,22 | 0,582136 |
| P53004 | Biliverdin reductase A OS=Homo sapiens GN=BLVRA PE=1 SV=2 - [BIEA_HUMAN] | 3560070 | 1085335 | 337316 | 10,55 | 0,067201 | 3,22 | 0,049348 | 3,28 | 0,016436 |
| P43251 | Biotinidase OS=Homo sapiens GN=BTD PE=1 SV=2 - [BTD_HUMAN] | 8615634 | 8601553 | 2706339 | 3,18 | 0,018462 | 3,18 | 0,001353 | 1,00 | 0,997748 |
| Q96GW7 | Brevican core protein OS=Homo sapiens GN=BCAN PE=1 SV=2 - [PGCB_HUMAN] | 332714 | 300101 | 22336 | 14,90 | 0,372845 | 13,44 | 0,128472 | 1,11 | 0,928105 |
| Q9NRL2 | Bromodomain adjacent to zinc finger domain protein 1A OS=Homo sapiens GN=BAZ1A PE=1 SV | 15107 | 2542 | 172 | 87,64 | 0,451657 | 14,74 | 0,355846 | 5,94 | 0,34783 |
| Q9Y2F9 | BTB/POZ domain-containing protein 3 OS=Homo sapiens GN=BTBD3 PE=2 SV=1 - [BTBD3_HUM | 1515791 | 1710118 | 111887 | 13,55 | 0,179287 | 15,28 | 0,054508 | 0,89 | 0,86233 |
| Q8IZJ3 | C3 and PZP-like alpha-2-macroglobulin domain-containing protein 8 OS=Homo sapiens GN=CP | 1406369 | 1367351 | 937507 | 1,50 | 0,304542 | 1,46 | 0,308368 | 1,03 | 0,943131 |
| P04003 | C4b-binding protein alpha chain OS=Homo sapiens GN=C4BPA PE=1 SV=2 - [C4BPA_HUMAN] | 6307 | 1058 | 199827 | 0,03 | 0,096721 | 0,01 | 0,102872 | 5,96 | 0,113595 |
| P55289 | Cadherin-12 OS=Homo sapiens GN=CDH12 PE=2 SV=2 - [CAD12_HUMAN] | 3572765 | 4479107 | 139150 | 25,68 | 1,23E-05 | 32,19 | 5,34E-06 | 0,80 | 0,158017 |
| P19022 | Cadherin-2 OS=Homo sapiens GN=CDH2 PE=1 SV=4 - [CADH2_HUMAN] | 3203031 | 2695055 | 1132667 | 2,83 | 0,030144 | 2,38 | 0,029155 | 1,19 | 0,52281 |
| Q96JP9 | Cadherin-related family member 1 OS=Homo sapiens GN=CDHR1 PE=1 SV=2 - [CDHR1_HUMA | 152291 | 109816 | 306848 | 0,50 | 0,399006 | 0,36 | 0,287236 | 1,39 | 0,457069 |
| Q9NWR8 | Calcium uniporter regulatory subunit MCUb, mitochondrial OS=Homo sapiens GN=CCDC109B | 7815719 | 9210426 | 5458034 | 1,43 | 0,142219 | 1,69 | 0,037195 | 0,85 | 0,363614 |
| Q14123 | Calcium/calmodulin-dependent 3',5'-cyclic nucleotide phosphodiesterase 1C OS=Homo sapien | 1469334 | 1802469 | 2081783 | 0,71 | 0,133617 | 0,87 | 0,394198 | 0,82 | 0,253714 |
| A8MX76 | Calpain-14 OS=Homo sapiens GN=CAPN14 PE=2 SV=2 - [CAN14_HUMAN] | 15747986 | 12187174 | 15559119 | 1,01 | 0,983116 | 0,78 | 0,498231 | 1,29 | 0,52281 |
| P27797 | Calreticulin OS=Homo sapiens GN=CALR PE=1 SV=1 - [CALR_HUMAN] | 334753 | 375962 | 241139 | 1,39 | 0,383035 | 1,56 | 0,056546 | 0,89 | 0,645735 |
| O94985 | Calsyntenin-1 OS=Homo sapiens GN=CLSTN1 PE=1 SV=1 - [CSTN1_HUMAN] | 64267366 | 63911758 | 22608562 | 2,84 | 0,001958 | 2,83 | 0,000194 | 1,01 | 0,980727 |
| Q9H4D0 | Calsyntenin-2 OS=Homo sapiens GN=CLSTN2 PE=1 SV=2 - [CSTN2_HUMAN] | 34358 | 24551 | 283 | 121,58 | 0,115936 | 86,87 | 0,046705 | 1,40 | 0,584344 |
| Q9BQT9 | Calsyntenin-3 OS=Homo sapiens GN=CLSTN3 PE=1 SV=1 - [CSTN3_HUMAN] | 275291 | 248396 | 87827 | 3,13 | 0,200241 | 2,83 | 0,112223 | 1,11 | 0,854632 |
| Q8IYT2 | Cap-specific mRNA (nucleoside-2'-O-)-methyltransferase 2 OS=Homo sapiens GN=CMTR2 PE=1 | 23142 | 46481 | 65243 | 0,35 | 0,027159 | 0,71 | 0,325397 | 0,50 | 0,088724 |
| Q7LGC8 | Carbohydrate sulfotransferase 3 OS=Homo sapiens GN=CHST3 PE=1 SV=3 - [CHST3_HUMAN] | 93233 | 24369 | 1080296 | 0,09 | 0,013401 | 0,02 | 0,014011 | 3,83 | 0,03009 |
| Q96IY4 | Carboxypeptidase B2 OS=Homo sapiens GN=CPB2 PE=1 SV=2 - [CBPB2_HUMAN] | 511625 | 425822 | 1275283 | 0,40 | 0,000118 | 0,33 | 1,76E-05 | 1,20 | 0,429824 |
| P16870 | Carboxypeptidase E OS=Homo sapiens GN=CPE PE=1 SV=1 - [CBPE_HUMAN] | 20748199 | 19624878 | 7036285 | 2,95 | 0,027269 | 2,79 | 0,011607 | 1,06 | 0,86159 |
| P22792 | Carboxypeptidase N subunit 2 OS=Homo sapiens GN=CPN2 PE=1 SV=3 - [CPN2_HUMAN] | 245510 | 314947 | 686196 | 0,36 | 0,036905 | 0,46 | 0,20127 | 0,78 | 0,761798 |
| Q9Y646 | Carboxypeptidase Q OS=Homo sapiens GN=CPQ PE=1 SV=1 - [CBPQ_HUMAN] | 37151 | 28393 | 0 | #DIV/0! | 0,031418 | #DIV/0! | 0,001328 | 1,31 | 0,499238 |
| Q8N3K9 | Cardiomyopathy-associated protein 5 OS=Homo sapiens GN=CMYA5 PE=1 SV=3 - [CMYA5_HU | 160838 | 90007 | 57742 | 2,79 | 0,209923 | 1,56 | 0,75581 | 1,79 | 0,398845 |
| Q9UBD9 | Cardiotrophin-like cytokine factor 1 OS=Homo sapiens GN=CLCF1 PE=1 SV=1 - [CLCF1_HUMAN | 1166389 | 1764125 | 1310380 | 0,89 | 0,714654 | 1,35 | 0,27536 | 0,66 | 0,075216 |
| Q9NQ79 | Cartilage acidic protein 1 OS=Homo sapiens GN=CRTAC1 PE=1 SV=2 - [CRAC1_HUMAN] | 5114542 | 5733297 | 3148207 | 1,62 | 0,077983 | 1,82 | 0,000595 | 0,89 | 0,457069 |
| P31944 | Caspase-14 OS=Homo sapiens GN=CASP14 PE=1 SV=2 - [CASPE_HUMAN] | 32537 | 38366 | 5962 | 5,46 | 0,431463 | 6,44 | 0,063276 | 0,85 | 0,85388 |
| P51878 | Caspase-5 OS=Homo sapiens GN=CASP5 PE=1 SV=3 - [CASP5_HUMAN] | 941481 | 220558 | 149682 | 6,29 | 0,084303 | 1,47 | 0,52563 | 4,27 | 0,007629 |
| P35222 | Catenin beta-1 OS=Homo sapiens GN=CTNNB1 PE=1 SV=1 - [CTNB1_HUMAN] | 2039975 | 3899096 | 129234 | 15,79 | 0,06826 | 30,17 | 0,001353 | 0,52 | 0,028927 |
| Q9UQB3 | Catenin delta-2 OS=Homo sapiens GN=CTNND2 PE=1 SV=3 - [CTND2_HUMAN] | 5689028 | 6086147 | 41622 | 136,68 | 0,038741 | 146,22 | 0,013526 | 0,93 | 0,89911 |
| P07858 | Cathepsin B OS=Homo sapiens GN=CTSB PE=1 SV=3 - [CATB_HUMAN] | 770641 | 555090 | 1030785 | 0,75 | 0,353322 | 0,54 | 0,049194 | 1,39 | 0,096836 |
| P07339 | Cathepsin D OS=Homo sapiens GN=CTSD PE=1 SV=1 - [CATD_HUMAN] | 75996578 | 79012016 | 66261048 | 1,15 | 0,548655 | 1,19 | 0,376634 | 0,96 | 0,834401 |
| Q9UBX1 | Cathepsin F OS=Homo sapiens GN=CTSF PE=1 SV=1 - [CATF_HUMAN] | 11900122 | 31276874 | 4578743 | 2,60 | 0,313331 | 6,83 | 7,91E-08 | 0,38 | 7,07E-05 |
| P07711 | Cathepsin L1 OS=Homo sapiens GN=CTSL PE=1 SV=2 - [CATL1_HUMAN] | 103129 | 111495 | 33723 | 3,06 | 0,348533 | 3,31 | 0,244846 | 0,92 | 0,926722 |
| Q9UBR2 | Cathepsin Z OS=Homo sapiens GN=CTSZ PE=1 SV=1 - [CATZ_HUMAN] | 621399 | 580196 | 322904 | 1,92 | 0,260383 | 1,80 | 0,128472 | 1,07 | 0,8803 |
| A5YKK6 | CCR4-NOT transcription complex subunit 1 OS=Homo sapiens GN=CNOT1 PE=1 SV=2 - [CNOT1 | 3776375 | 5590493 | 6064509 | 0,62 | 0,207625 | 0,92 | 0,806559 | 0,68 | 0,240956 |
| Q13740 | CD166 antigen OS=Homo sapiens GN=ALCAM PE=1 SV=2 - [CD166_HUMAN] | 44977 | 358822 | 17424 | 2,58 | 0,784366 | 20,59 | 0,548804 | 0,13 | 0,404952 |
| P16070 | CD44 antigen OS=Homo sapiens GN=CD44 PE=1 SV=3 - [CD44_HUMAN] | 64234 | 50502 | 17361 | 3,70 | 0,076266 | 2,91 | 0,231141 | 1,27 | 0,606961 |
| P13987 | CD59 glycoprotein OS=Homo sapiens GN=CD59 PE=1 SV=1 - [CD59_HUMAN] | 439770 | 849960 | 213556 | 2,06 | 0,491518 | 3,98 | 0,015197 | 0,52 | 0,09559 |
| Q9BY67 | Cell adhesion molecule 1 OS=Homo sapiens GN=CADM1 PE=1 SV=2 - [CADM1_HUMAN] | 1729528 | 1964022 | 3978562 | 0,43 | 0,002371 | 0,49 | 1,53E-05 | 0,88 | 0,662223 |
| Q8N3J6 | Cell adhesion molecule 2 OS=Homo sapiens GN=CADM2 PE=2 SV=1 - [CADM2_HUMAN] | 55169 | 27929 | 2093 | 26,36 | 0,291274 | 13,35 | 0,061495 | 1,98 | 0,449275 |
| Q69YH5 | Cell division cycle-associated protein 2 OS=Homo sapiens GN=CDCA2 PE=1 SV=2 - [CDCA2_HU | 30573 | 2566 | 322195 | 0,09 | 0,023685 | 0,01 | 0,013944 | 11,92 | 0,404952 |
| Q99618 | Cell division cycle-associated protein 3 OS=Homo sapiens GN=CDCA3 PE=1 SV=1 - [CDCA3_HU | 85833 | 202271 | 42440 | 2,02 | 0,38177 | 4,77 | 0,000127 | 0,42 | 0,000687 |
| Q8N137 | Centrobin OS=Homo sapiens GN=CNTROB PE=1 SV=1 - [CNTRB_HUMAN] | 8405583 | 2568220 | 1576459 | 5,33 | 0,017437 | 1,63 | 0,283597 | 3,27 | 0,000709 |
| Q9BYV8 | Centrosomal protein of 41 kDa OS=Homo sapiens GN=CEP41 PE=1 SV=1 - [CEP41_HUMAN] | 94813792 | 94717550 | 102245271 | 0,93 | 0,897664 | 0,93 | 0,905487 | 1,00 | 0,998213 |
| Q9BV73 | Centrosome-associated protein CEP250 OS=Homo sapiens GN=CEP250 PE=1 SV=2 - [CP250_HU | 39188 | 42183 | 66153 | 0,59 | 0,701586 | 0,64 | 0,766922 | 0,93 | 0,975388 |
| O75503 | Ceroid-lipofuscinosis neuronal protein 5 OS=Homo sapiens GN=CLN5 PE=1 SV=2 - [CLN5_HUM | 116547 | 135771 | 60081 | 1,94 | 0,453877 | 2,26 | 0,109539 | 0,86 | 0,770502 |
| P00450 | Ceruloplasmin OS=Homo sapiens GN=CP PE=1 SV=1 - [CERU_HUMAN] | 432915472 | 392743517 | 404939371 | 1,07 | 0,50085 | 0,97 | 0,728964 | 1,10 | 0,161102 |
| P36222 | Chitinase-3-like protein 1 OS=Homo sapiens GN=CHI3L1 PE=1 SV=2 - [CH3L1_HUMAN] | 6515617 | 6819952 | 5572714 | 1,17 | 0,571859 | 1,22 | 0,241351 | 0,96 | 0,826513 |
| P51800 | Chloride channel protein ClC-Ka OS=Homo sapiens GN=CLCNKA PE=1 SV=1 - [CLCKA_HUMAN] | 192638 | 708193 | 368082 | 0,52 | 0,168661 | 1,92 | 0,540327 | 0,27 | 0,117475 |
| P22680 | Cholesterol 7-alpha-monooxygenase OS=Homo sapiens GN=CYP7A1 PE=1 SV=2 - [CP7A1_HUM | 13428965 | 29370485 | 30346431 | 0,44 | 0,054873 | 0,97 | 0,939778 | 0,46 | 0,079174 |
| Q86X52 | Chondroitin sulfate synthase 1 OS=Homo sapiens GN=CHSY1 PE=1 SV=3 - [CHSS1_HUMAN] | 6254952 | 1714291 | 2876489 | 2,17 | 0,092208 | 0,60 | 0,116386 | 3,65 | 8,55E-05 |
| Q9BU40 | Chordin-like protein 1 OS=Homo sapiens GN=CHRDL1 PE=1 SV=1 - [CRDL1_HUMAN] | 394675 | 357167 | 815945 | 0,48 | 0,102876 | 0,44 | 0,021587 | 1,11 | 0,859351 |
| P10645 | Chromogranin-A OS=Homo sapiens GN=CHGA PE=1 SV=7 - [CMGA_HUMAN] | 1530969 | 1636717 | 271500 | 5,64 | 0,090974 | 6,03 | 0,025632 | 0,94 | 0,898927 |
| Q6ZTR5 | Cilia- and flagella-associated protein 47 OS=Homo sapiens GN=CFAP47 PE=2 SV=4 - [CFA47_HU | 328383 | 245609 | 1134461 | 0,29 | 0,014093 | 0,22 | 0,009933 | 1,34 | 0,338818 |
| Q96N23 | Cilia- and flagella-associated protein 54 OS=Homo sapiens GN=CFAP54 PE=2 SV=3 - [CFA54_HU | 1613756 | 1815466 | 1498017 | 1,08 | 0,931299 | 1,21 | 0,639223 | 0,89 | 0,850054 |
| Q96MR6 | Cilia- and flagella-associated protein 57 OS=Homo sapiens GN=CFAP57 PE=2 SV=3 - [CFA57_HU | 2179435 | 4867951 | 633691 | 3,44 | 0,105191 | 7,68 | 0,00447 | 0,45 | 0,004626 |
| D6REC4 | Cilia- and flagella-associated protein 99 OS=Homo sapiens GN=CFAP99 PE=3 SV=1 - [CFA99_HU | 267315 | 275955 | 75625 | 3,53 | 0,453877 | 3,65 | 0,28471 | 0,97 | 0,975774 |
| Q9P2M7 | Cingulin OS=Homo sapiens GN=CGN PE=1 SV=2 - [CING_HUMAN] | 1147424 | 2099882 | 529615 | 2,17 | 0,095154 | 3,96 | 0,001353 | 0,55 | 0,003707 |
| Q0VF96 | Cingulin-like protein 1 OS=Homo sapiens GN=CGNL1 PE=1 SV=2 - [CGNL1_HUMAN] | 6423602 | 6000893 | 7334779 | 0,88 | 0,591682 | 0,82 | 0,418008 | 1,07 | 0,784223 |
| Q00610 | Clathrin heavy chain 1 OS=Homo sapiens GN=CLTC PE=1 SV=5 - [CLH1_HUMAN] | 689611 | 1191460 | 99753 | 6,91 | 0,10028 | 11,94 | 0,186418 | 0,58 | 0,404952 |
| P10909 | Clusterin OS=Homo sapiens GN=CLU PE=1 SV=1 - [CLUS_HUMAN] | 547098582 | 528845131 | 533301225 | 1,03 | 0,860746 | 0,99 | 0,929663 | 1,03 | 0,760808 |
| Q15846 | Clusterin-like protein 1 OS=Homo sapiens GN=CLUL1 PE=2 SV=1 - [CLUL1_HUMAN] | 373713 | 219522 | 389451 | 0,96 | 0,931299 | 0,56 | 0,101699 | 1,70 | 0,116358 |
| P00740 | Coagulation factor IX OS=Homo sapiens GN=F9 PE=1 SV=2 - [FA9_HUMAN] | 67410 | 64567 | 264160 | 0,26 | 0,000118 | 0,24 | 0,000322 | 1,04 | 0,936932 |
| P12259 | Coagulation factor V OS=Homo sapiens GN=F5 PE=1 SV=4 - [FA5_HUMAN] | 431791 | 494672 | 109523 | 3,94 | 0,112776 | 4,52 | 0,021713 | 0,87 | 0,751588 |
| P00748 | Coagulation factor XII OS=Homo sapiens GN=F12 PE=1 SV=3 - [FA12_HUMAN] | 4467312 | 4627326 | 7466825 | 0,60 | 0,101858 | 0,62 | 0,145975 | 0,97 | 0,944533 |
| P05160 | Coagulation factor XIII B chain OS=Homo sapiens GN=F13B PE=1 SV=3 - [F13B_HUMAN] | 36105 | 7809 | 57352 | 0,63 | 0,448353 | 0,14 | 0,000514 | 4,62 | 0,07556 |
| Q8TBZ0 | Coiled-coil domain-containing protein 110 OS=Homo sapiens GN=CCDC110 PE=1 SV=1 - [CC110 | 242462155 | 298976363 | 304421061 | 0,80 | 0,59504 | 0,98 | 0,968824 | 0,81 | 0,662535 |
| Q6ZP82 | Coiled-coil domain-containing protein 141 OS=Homo sapiens GN=CCDC141 PE=1 SV=2 - [CC14 | 84345 | 245190 | 50502 | 1,67 | 0,421993 | 4,86 | 0,051821 | 0,34 | 0,002575 |
| Q0P6D6 | Coiled-coil domain-containing protein 15 OS=Homo sapiens GN=CCDC15 PE=2 SV=2 - [CCD15_ | 1246373 | 1324123 | 1253951 | 0,99 | 0,987293 | 1,06 | 0,857316 | 0,94 | 0,862436 |
| Q8NCX0 | Coiled-coil domain-containing protein 150 OS=Homo sapiens GN=CCDC150 PE=1 SV=2 - [CC150 | 142648 | 41272 | 42770 | 3,34 | 0,283544 | 0,96 | 0,955241 | 3,46 | 0,087517 |
| A6NI56 | Coiled-coil domain-containing protein 154 OS=Homo sapiens GN=CCDC154 PE=2 SV=4 - [CC154 | 96170 | 109200 | 128470 | 0,75 | 0,698175 | 0,85 | 0,704462 | 0,88 | 0,866059 |

| | | | | | | | | | | |
|---|---|---|---|---|---|---|---|---|---|---|
| Q5M9N0 | Coiled-coil domain-containing protein 158 OS=Homo sapiens GN=CCDC158 PE=1 SV=2 - [CD15 | 609989 | 516737 | 883470 | 0,69 | 0,660598 | 0,58 | 0,498231 | 1,18 | 0,764817 |
| Q5BJE1 | Coiled-coil domain-containing protein 178 OS=Homo sapiens GN=CCDC178 PE=2 SV=3 - [CC17 | 10656195 | 4959628 | 27197 | 391,82 | 0,535422 | 182,36 | 0,395757 | 2,15 | 0,671602 |
| Q5T9S5 | Coiled-coil domain-containing protein 18 OS=Homo sapiens GN=CCDC18 PE=2 SV=1 - [CCD18_ | 998772 | 729461 | 3099642 | 0,32 | 0,000189 | 0,24 | 3E-05 | 1,37 | 0,475291 |
| Q9P1Z9 | Coiled-coil domain-containing protein 180 OS=Homo sapiens GN=CCDC180 PE=2 SV=2 - [CC18 | 36312317 | 34986270 | 13623129 | 2,67 | 0,092208 | 2,57 | 0,086503 | 1,04 | 0,936932 |
| Q8IWP9 | Coiled-coil domain-containing protein 28A OS=Homo sapiens GN=CCDC28A PE=1 SV=1 - [CC28 | 10334179 | 2347563 | 19222375 | 0,54 | 0,049831 | 0,12 | 6,33E-19 | 4,40 | 0,002407 |
| Q9UFE4 | Coiled-coil domain-containing protein 39 OS=Homo sapiens GN=CCDC39 PE=2 SV=3 - [CCD39_ | 2873277 | 2131724 | 956850 | 3,00 | 0,001933 | 2,23 | 0,00024 | 1,35 | 0,059532 |
| Q8NEL0 | Coiled-coil domain-containing protein 54 OS=Homo sapiens GN=CCDC54 PE=1 SV=2 - [CCD54_ | 1411242 | 1511339 | 5848602 | 0,24 | 0,003362 | 0,26 | 0,002102 | 0,93 | 0,944533 |
| Q4VC31 | Coiled-coil domain-containing protein 58 OS=Homo sapiens GN=CCDC58 PE=1 SV=1 - [CCD58_ | 2463413 | 3021821 | 5258950 | 0,47 | 0,105322 | 0,57 | 0,368896 | 0,82 | 0,795467 |
| Q96F63 | Coiled-coil domain-containing protein 97 OS=Homo sapiens GN=CCDC97 PE=1 SV=1 - [CCD97_ | 3360512 | 6735018 | 2803151 | 1,20 | 0,647018 | 2,40 | 0,000381 | 0,50 | 0,000147 |
| P02458 | Collagen alpha-1(II) chain OS=Homo sapiens GN=COL2A1 PE=1 SV=3 - [CO2A1_HUMAN] | 529190 | 356993 | 45740 | 11,57 | 0,037442 | 7,80 | 0,103857 | 1,48 | 0,39219 |
| P20849 | Collagen alpha-1(IX) chain OS=Homo sapiens GN=COL9A1 PE=1 SV=3 - [CO9A1_HUMAN] | 311222 | 486214 | 371029 | 0,84 | 0,582836 | 1,31 | 0,589146 | 0,64 | 0,220784 |
| P12109 | Collagen alpha-1(VI) chain OS=Homo sapiens GN=COL6A1 PE=1 SV=3 - [CO6A1_HUMAN] | 1476390 | 1315973 | 1314731 | 1,12 | 0,698175 | 1,00 | 0,995663 | 1,12 | 0,631571 |
| Q02388 | Collagen alpha-1(VII) chain OS=Homo sapiens GN=COL7A1 PE=1 SV=2 - [CO7A1_HUMAN] | 174072 | 22518 | 9777 | 17,80 | 0,221927 | 2,30 | 0,720079 | 7,73 | 0,078298 |
| P39060 | Collagen alpha-1(XVIII) chain OS=Homo sapiens GN=COL18A1 PE=1 SV=5 - [COIA1_HUMAN] | 1746845 | 2162617 | 1309672 | 1,33 | 0,288925 | 1,65 | 0,059364 | 0,81 | 0,278282 |
| P08123 | Collagen alpha-2(I) chain OS=Homo sapiens GN=COL1A2 PE=1 SV=7 - [CO1A2_HUMAN] | 63514 | 21664 | 1005 | 63,17 | 0,163986 | 21,55 | 0,075437 | 2,93 | 0,156797 |
| Q14055 | Collagen alpha-2(IX) chain OS=Homo sapiens GN=COL9A2 PE=1 SV=2 - [CO9A2_HUMAN] | 135718 | 194129 | 54925 | 2,47 | 0,189854 | 3,53 | 0,33362 | 0,70 | 0,597745 |
| Q5KU26 | Collectin-12 OS=Homo sapiens GN=COLEC12 PE=1 SV=3 - [COL12_HUMAN] | 85139 | 78781 | 7026 | 12,12 | 0,142699 | 11,21 | 0,056553 | 1,08 | 0,913688 |
| P02745 | Complement C1q subcomponent subunit A OS=Homo sapiens GN=C1QA PE=1 SV=2 - [C1QA_H | 55204 | 67812 | 3972 | 13,90 | 0,475059 | 17,07 | 0,117772 | 0,81 | 0,85388 |
| P02746 | Complement C1q subcomponent subunit B OS=Homo sapiens GN=C1QB PE=1 SV=3 - [C1QB_H | 85817 | 55632 | 35 | 2478,77 | 0,117392 | 1606,87 | 0,115518 | 1,54 | 0,506893 |
| P02747 | Complement C1q subcomponent subunit C OS=Homo sapiens GN=C1QC PE=1 SV=3 - [C1QC_H | 746983 | 543708 | 218737 | 3,41 | 0,095154 | 2,49 | 0,155338 | 1,37 | 0,434772 |
| Q9BXJ4 | Complement C1q tumor necrosis factor-related protein 3 OS=Homo sapiens GN=C1QTNF3 PE= | 242404 | 219225 | 1291 | 187,78 | 0,13518 | 169,83 | 0,024792 | 1,11 | 0,887972 |
| P00736 | Complement C1r subcomponent OS=Homo sapiens GN=C1R PE=1 SV=2 - [C1R_HUMAN] | 2502929 | 1674257 | 3164003 | 0,79 | 0,422813 | 0,53 | 0,050748 | 1,49 | 0,012894 |
| Q9NZP8 | Complement C1r subcomponent-like protein OS=Homo sapiens GN=C1RL PE=1 SV=2 - [C1RL_H | 65413 | 26464 | 2635 | 24,82 | 0,037599 | 10,04 | 0,042308 | 2,47 | 0,038203 |
| P09871 | Complement C1s subcomponent OS=Homo sapiens GN=C1S PE=1 SV=1 - [C1S_HUMAN] | 622419 | 547490 | 465741 | 1,34 | 0,5356 | 1,18 | 0,697634 | 1,14 | 0,70068 |
| P06681 | Complement C2 OS=Homo sapiens GN=C2 PE=1 SV=2 - [CO2_HUMAN] | 4033194 | 4172678 | 5625678 | 0,72 | 0,077983 | 0,74 | 0,018289 | 0,97 | 0,86656 |
| P01024 | Complement C3 OS=Homo sapiens GN=C3 PE=1 SV=2 - [CO3_HUMAN] | 214705954 | 204192921 | 306869834 | 0,70 | 0,058554 | 0,67 | 0,029527 | 1,05 | 0,834401 |
| P0C0L4 | Complement C4-A OS=Homo sapiens GN=C4A PE=1 SV=2 - [CO4A_HUMAN] | 213168444 | 193135678 | 268518808 | 0,79 | 0,146028 | 0,72 | 0,032773 | 1,10 | 0,538047 |
| P0C0L5 | Complement C4-B OS=Homo sapiens GN=C4B PE=1 SV=2 - [CO4B_HUMAN] | 12549474 | 9478770 | 8435110 | 1,49 | 0,229997 | 1,12 | 0,678249 | 1,32 | 0,240018 |
| P01031 | Complement C5 OS=Homo sapiens GN=C5 PE=1 SV=4 - [CO5_HUMAN] | 1626517 | 1748676 | 2439413 | 0,67 | 0,164629 | 0,72 | 0,135284 | 0,93 | 0,798687 |
| Q9NPY3 | Complement component C1q receptor OS=Homo sapiens GN=CD93 PE=1 SV=3 - [C1QR1_HUM | 29545 | 16030 | 0 | #DIV/0! | 0,12891 | #DIV/0! | 0,026996 | 1,84 | 0,335899 |
| P13671 | Complement component C6 OS=Homo sapiens GN=C6 PE=1 SV=3 - [CO6_HUMAN] | 2372108 | 2343994 | 3368564 | 0,70 | 0,197075 | 0,70 | 0,325499 | 1,01 | 0,980727 |
| P10643 | Complement component C7 OS=Homo sapiens GN=C7 PE=1 SV=2 - [CO7_HUMAN] | 1726957 | 1563257 | 5084386 | 0,34 | 3,62E-06 | 0,31 | 0,000449 | 1,10 | 0,828681 |
| P07357 | Complement component C8 alpha chain OS=Homo sapiens GN=C8A PE=1 SV=2 - [CO8A_HUMA | 2222823 | 2876612 | 2784263 | 0,80 | 0,251404 | 1,03 | 0,896603 | 0,77 | 0,208596 |
| P07358 | Complement component C8 beta chain OS=Homo sapiens GN=C8B PE=1 SV=3 - [CO8B_HUMAI | 4223584 | 5336674 | 3054800 | 1,38 | 0,228399 | 1,75 | 0,219615 | 0,79 | 0,425918 |
| P07360 | Complement component C8 gamma chain OS=Homo sapiens GN=C8G PE=1 SV=3 - [CO8G_HUI | 1907874 | 2201776 | 3970766 | 0,48 | 0,017169 | 0,55 | 0,038409 | 0,87 | 0,705633 |
| P02748 | Complement component C9 OS=Homo sapiens GN=C9 PE=1 SV=2 - [CO9_HUMAN] | 15127942 | 19035124 | 34989747 | 0,43 | 6,86E-05 | 0,54 | 0,003537 | 0,79 | 0,266723 |
| P00751 | Complement factor B OS=Homo sapiens GN=CFB PE=1 SV=2 - [CFAB_HUMAN] | 73334533 | 77634619 | 112032082 | 0,65 | 0,036414 | 0,69 | 0,021396 | 0,94 | 0,804356 |
| P00746 | Complement factor D OS=Homo sapiens GN=CFD PE=1 SV=5 - [CFAD_HUMAN] | 4133893 | 2756533 | 4807128 | 0,86 | 0,664993 | 0,57 | 0,072074 | 1,50 | 0,154882 |
| P08603 | Complement factor H OS=Homo sapiens GN=CFH PE=1 SV=4 - [CFAH_HUMAN] | 17785963 | 24457548 | 29786937 | 0,60 | 0,100458 | 0,82 | 0,266336 | 0,73 | 0,113968 |
| Q03591 | Complement factor H-related protein 1 OS=Homo sapiens GN=CFHR1 PE=1 SV=2 - [FHR1_HUM | 2621027 | 3045161 | 8952355 | 0,29 | 4,57E-05 | 0,34 | 1,93E-06 | 0,86 | 0,700135 |
| P36980 | Complement factor H-related protein 2 OS=Homo sapiens GN=CFHR2 PE=1 SV=1 - [FHR2_HUM | 65499 | 97495 | 505118 | 0,13 | 7,48E-07 | 0,19 | 2,39E-06 | 0,67 | 0,429824 |
| P05156 | Complement factor I OS=Homo sapiens GN=CFI PE=1 SV=2 - [CFAI_HUMAN] | 20714026 | 21461272 | 24955421 | 0,83 | 0,11815 | 0,86 | 0,259551 | 0,97 | 0,811877 |
| Q8WXI2 | Connector enhancer of kinase suppressor of ras 2 OS=Homo sapiens GN=CNKSR2 PE=1 SV=1 - | 173954235 | 156060013 | 159306738 | 1,09 | 0,835108 | 0,98 | 0,957583 | 1,11 | 0,80581 |
| Q14746 | Conserved oligomeric Golgi complex subunit 2 OS=Homo sapiens GN=COG2 PE=1 SV=1 - [COG | 4063257 | 1754031 | 860562 | 4,72 | 0,000353 | 2,04 | 0,117106 | 2,32 | 0,000134 |
| Q96JB2 | Conserved oligomeric Golgi complex subunit 3 OS=Homo sapiens GN=COG3 PE=1 SV=3 - [COG | 110648 | 258 | 3844 | 28,79 | 0,431226 | 0,07 | 0,085094 | 428,66 | 0,204724 |
| Q12860 | Contactin-1 OS=Homo sapiens GN=CNTN1 PE=1 SV=1 - [CNTN1_HUMAN] | 945733 | 906945 | 201906 | 4,68 | 0,073413 | 4,49 | 0,012508 | 1,04 | 0,93146 |
| P08185 | Corticosteroid-binding globulin OS=Homo sapiens GN=SERPINA6 PE=1 SV=1 - [CBG_HUMAN] | 11273920 | 9808540 | 15457606 | 0,73 | 0,07511 | 0,63 | 0,009609 | 1,15 | 0,429824 |
| Q9H668 | CST complex subunit STN1 OS=Homo sapiens GN=OBFC1 PE=1 SV=2 - [STN1_HUMAN] | 86511331 | 101361183 | 105833550 | 0,82 | 0,339992 | 0,96 | 0,827647 | 0,85 | 0,429824 |
| Q96M20 | Cyclic nucleotide-binding domain-containing protein 2 OS=Homo sapiens GN=CNBD2 PE=2 SV= | 4388767 | 4361720 | 2432150 | 1,80 | 0,137946 | 1,79 | 0,143204 | 1,01 | 0,989184 |
| Q15131 | Cyclin-dependent kinase 10 OS=Homo sapiens GN=CDK10 PE=1 SV=1 - [CDK10_HUMAN] | 245920 | 143386 | 172421 | 1,43 | 0,37617 | 0,83 | 0,65121 | 1,72 | 0,068083 |
| Q07002 | Cyclin-dependent kinase 18 OS=Homo sapiens GN=CDK18 PE=1 SV=3 - [CDK18_HUMAN] | 135425 | 23654 | 3205 | 42,26 | 0,037714 | 7,38 | 0,173939 | 5,73 | 0,003207 |
| P51959 | Cyclin-G1 OS=Homo sapiens GN=CCNG1 PE=1 SV=2 - [CCNG1_HUMAN] | 568793 | 645527 | 730890 | 0,78 | 0,712088 | 0,88 | 0,849639 | 0,88 | 0,834987 |
| P01034 | Cystatin-C OS=Homo sapiens GN=CST3 PE=1 SV=1 - [CYTC_HUMAN] | 133094180 | 171154135 | 66532281 | 2,00 | 0,202773 | 2,57 | 0,054497 | 0,78 | 0,443443 |
| Q15828 | Cystatin-M OS=Homo sapiens GN=CST6 PE=1 SV=1 - [CYTM_HUMAN] | 25458 | 11709 | 132349 | 0,19 | 0,00145 | 0,09 | 0,000241 | 2,17 | 0,338818 |
| Q6ZMK1 | Cysteine and histidine-rich protein 1 OS=Homo sapiens GN=CYHR1 PE=1 SV=2 - [CYHR1_HUMA | 245252 | 226253 | 73942 | 3,32 | 0,081289 | 3,06 | 0,022266 | 1,08 | 0,834506 |
| P49589 | Cysteine--tRNA ligase, cytoplasmic OS=Homo sapiens GN=CARS PE=1 SV=3 - [SYCC_HUMAN] | 6180970 | 7396977 | 10268436 | 0,60 | 0,00077 | 0,72 | 0,005829 | 0,84 | 0,134695 |
| O43174 | Cytochrome P450 26A1 OS=Homo sapiens GN=CYP26A1 PE=2 SV=2 - [CP26A_HUMAN] | 108011 | 272473 | 707278 | 0,15 | 0,000353 | 0,39 | 0,023865 | 0,40 | 0,004039 |
| Q14204 | Cytoplasmic dynein 1 heavy chain 1 OS=Homo sapiens GN=DYNC1H1 PE=1 SV=5 - [DYHC1_HUI | 6902460 | 5820063 | 7071751 | 0,98 | 0,962372 | 0,82 | 0,648171 | 1,19 | 0,70068 |
| Q9Y6G9 | Cytoplasmic dynein 1 light intermediate chain 1 OS=Homo sapiens GN=DYNC1LI1 PE=1 SV=3 - | 6155047 | 3807938 | 5267349 | 1,17 | 0,634554 | 0,72 | 0,186418 | 1,62 | 0,045618 |
| Q8NCM8 | Cytoplasmic dynein 2 heavy chain 1 OS=Homo sapiens GN=DYNC2H1 PE=1 SV=4 - [DYHC2_HUI | 206529 | 240851 | 139453 | 1,48 | 0,492273 | 1,73 | 0,370929 | 0,86 | 0,74924 |
| Q96MI9 | Cytosolic carboxypeptidase 4 OS=Homo sapiens GN=AGBL1 PE=1 SV=2 - [CBPC4_HUMAN] | 1265305 | 115153 | 385610 | 3,28 | 0,34549 | 0,30 | 0,002083 | 10,99 | 0,038203 |
| Q9BTE7 | DCN1-like protein 5 OS=Homo sapiens GN=DCUN1D5 PE=1 SV=1 - [DCNL5_HUMAN] | 2637642 | 3836290 | 3681393 | 0,72 | 0,472106 | 1,04 | 0,918089 | 0,69 | 0,386254 |
| P0C7V8 | DDB1- and CUL4-associated factor 8-like protein 2 OS=Homo sapiens GN=DCAF8L2 PE=2 SV=2 | 2561309 | 1279511 | 6764568 | 0,38 | 2,21E-05 | 0,19 | 3,65E-12 | 2,00 | 0,00739 |
| Q96HY6 | DDRGK domain-containing protein 1 OS=Homo sapiens GN=DDRGK1 PE=1 SV=2 - [DDRGK_HUI | 52799 | 13005 | 2808 | 18,80 | 0,046075 | 4,63 | 0,05203 | 4,06 | 0,007072 |
| P53355 | Death-associated protein kinase 1 OS=Homo sapiens GN=DAPK1 PE=1 SV=6 - [DAPK1_HUMAN | 948 | 27106 | 189439 | 0,01 | 0,131305 | 0,14 | 0,238929 | 0,03 | 0,404952 |
| Q14185 | Dedicator of cytokinesis protein 1 OS=Homo sapiens GN=DOCK1 PE=1 SV=2 - [DOCK1_HUMAN | 504278 | 379549 | 422123 | 1,19 | 0,672685 | 0,90 | 0,710878 | 1,33 | 0,336631 |
| Q8N1I0 | Dedicator of cytokinesis protein 4 OS=Homo sapiens GN=DOCK4 PE=1 SV=3 - [DOCK4_HUMAN | 4653239 | 737564 | 486392 | 9,57 | 0,014192 | 1,52 | 0,306986 | 6,31 | 9,92E-05 |
| Q9H7D0 | Dedicator of cytokinesis protein 5 OS=Homo sapiens GN=DOCK5 PE=1 SV=3 - [DOCK5_HUMAN | 11803 | 31518 | 0 | #DIV/0! | 0,432156 | #DIV/0! | 0,0961 | 0,37 | 0,186937 |
| Q96N67 | Dedicator of cytokinesis protein 7 OS=Homo sapiens GN=DOCK7 PE=1 SV=4 - [DOCK7_HUMAN | 953818 | 2757586 | 5499240 | 0,17 | 0,000123 | 0,50 | 0,059311 | 0,35 | 0,061888 |
| Q8NF50 | Dedicator of cytokinesis protein 8 OS=Homo sapiens GN=DOCK8 PE=1 SV=3 - [DOCK8_HUMAN | 9504 | 34517 | 522527 | 0,02 | 0,005543 | 0,07 | 0,015472 | 0,28 | 0,038663 |
| Q9BZ29 | Dedicator of cytokinesis protein 9 OS=Homo sapiens GN=DOCK9 PE=1 SV=2 - [DOCK9_HUMAN | 463276 | 620810 | 258329 | 1,79 | 0,155702 | 2,40 | 0,053336 | 0,75 | 0,266782 |
| Q15392 | Delta(24)-sterol reductase OS=Homo sapiens GN=DHCR24 PE=1 SV=2 - [DHC24_HUMAN] | 2656620 | 3401462 | 1816382 | 1,46 | 0,200241 | 1,87 | 0,034756 | 0,78 | 0,223973 |
| Q68D51 | DENN domain-containing protein 2C OS=Homo sapiens GN=DENND2C PE=1 SV=2 - [DEN2C_HU | 511991 | 363876 | 880327 | 0,58 | 0,110446 | 0,41 | 0,013085 | 1,41 | 0,345161 |
| Q6IQ26 | DENN domain-containing protein 5A OS=Homo sapiens GN=DENND5A PE=1 SV=2 - [DEN5A_HU | 2237114 | 4429906 | 2230130 | 1,00 | 0,995289 | 1,99 | 0,109421 | 0,51 | 0,003144 |
| O00115 | Deoxyribonuclease-2-alpha OS=Homo sapiens GN=DNASE2 PE=1 SV=2 - [DNS2A_HUMAN] | 181359 | 98509 | 16641 | 10,90 | 0,24035 | 5,92 | 0,015551 | 1,84 | 0,404952 |
| P81605 | Dermcidin OS=Homo sapiens GN=DCD PE=1 SV=2 - [DCD_HUMAN] | 30556 | 30394 | 2534 | 12,06 | 0,566375 | 11,99 | 0,461805 | 1,01 | 0,998057 |
| Q02413 | Desmoglein-1 OS=Homo sapiens GN=DSG1 PE=1 SV=2 - [DSG1_HUMAN] | 15725 | 25429 | 18214 | 0,86 | 0,856848 | 1,40 | 0,784714 | 0,62 | 0,620552 |
| Q9UBP4 | Dickkopf-related protein 3 OS=Homo sapiens GN=DKK3 PE=1 SV=2 - [DKK3_HUMAN] | 78901016 | 82018791 | 62741634 | 1,26 | 0,507745 | 1,31 | 0,145975 | 0,96 | 0,895228 |
| Q9UHL4 | Dipeptidyl peptidase 2 OS=Homo sapiens GN=DPP7 PE=1 SV=3 - [DPP2_HUMAN] | 265486 | 209448 | 29643 | 8,96 | 0,355982 | 7,07 | 0,120463 | 1,27 | 0,802075 |
| Q8TDJ6 | DmX-like protein 2 OS=Homo sapiens GN=DMXL2 PE=1 SV=2 - [DMXL2_HUMAN] | 9725 | 21912 | 104835 | 0,09 | 0,002184 | 0,21 | 0,018081 | 0,44 | 0,236508 |
| P26358 | DNA (cytosine-5)-methyltransferase 1 OS=Homo sapiens GN=DNMT1 PE=1 SV=2 - [DNMT1_HU | 3952428 | 8587929 | 6529996 | 0,61 | 0,17278 | 1,32 | 0,102066 | 0,46 | 0,000251 |
| Q99708 | DNA endonuclease RBBP8 OS=Homo sapiens GN=RBBP8 PE=1 SV=2 - [COM1_HUMAN] | 1407277 | 642 | 6708 | 209,80 | 0,101159 | 0,10 | 0,094159 | 2191,50 | 0,00509 |
| Q03468 | DNA excision repair protein ERCC-6 OS=Homo sapiens GN=ERCC6 PE=1 SV=1 - [ERCC6_HUMAN | 150955 | 50599 | 112290 | 1,34 | 0,540904 | 0,45 | 0,051525 | 2,98 | 0,007448 |
| Q2NKX8 | DNA excision repair protein ERCC-6-like OS=Homo sapiens GN=ERCC6L PE=1 SV=1 - [ERC6L_HU | 56117 | 174657 | 31338 | 1,79 | 0,593664 | 5,57 | 0,020541 | 0,32 | 0,005152 |
| Q9Y620 | DNA repair and recombination protein RAD54B OS=Homo sapiens GN=RAD54B PE=1 SV=1 - [R | 196465 | 243982 | 308438 | 0,64 | 0,266129 | 0,79 | 0,496927 | 0,81 | 0,615747 |
| Q92878 | DNA repair protein RAD50 OS=Homo sapiens GN=RAD50 PE=1 SV=1 - [RAD50_HUMAN] | 945816 | 1385599 | 1285826 | 0,74 | 0,185826 | 1,08 | 0,629674 | 0,68 | 0,010151 |
| Q9UBZ9 | DNA repair protein REV1 OS=Homo sapiens GN=REV1 PE=1 SV=1 - [REV1_HUMAN] | 714503 | 724574 | 145228 | 4,92 | 0,01261 | 4,99 | 0,000961 | 0,99 | 0,963841 |
| P51530 | DNA replication ATP-dependent helicase/nuclease DNA2 OS=Homo sapiens GN=DNA2 PE=1 SV | 47842 | 5354 | 8671 | 5,52 | 0,189854 | 0,62 | 0,518961 | 8,94 | 0,015453 |
| Q92547 | DNA topoisomerase 2-binding protein 1 OS=Homo sapiens GN=TOPBP1 PE=1 SV=3 - [TOPB1_H | 293717 | 334998 | 120434 | 2,44 | 0,13485 | 2,78 | 0,069704 | 0,88 | 0,739178 |
| Q9UBS4 | DnaJ homolog subfamily B member 11 OS=Homo sapiens GN=DNAJB11 PE=1 SV=1 - [DJB11_HU | 84637 | 85011 | 56541 | 1,50 | 0,752097 | 1,50 | 0,546584 | 1,00 | 0,997748 |
| Q9Y672 | Dolichyl pyrophosphate Man9GlcNAc2 alpha-1,3-glucosyltransferase OS=Homo sapiens GN=AI | 424904 | 392906 | 255205 | 1,66 | 0,493413 | 1,54 | 0,093594 | 1,08 | 0,883437 |
| Q14184 | Double C2-like domain-containing protein beta OS=Homo sapiens GN=DOC2B PE=1 SV=1 - [DO | 89085 | 113521 | 38290 | 2,33 | 0,296773 | 2,96 | 0,057411 | 0,78 | 0,546777 |
| O60216 | Double-strand-break repair protein rad21 homolog OS=Homo sapiens GN=RAD21 PE=1 SV=2 - | 34546990 | 57749017 | 46782395 | 0,74 | 0,31883 | 1,23 | 0,206233 | 0,60 | 0,005498 |
| O60313 | Dynamin-like 120 kDa protein, mitochondrial OS=Homo sapiens GN=OPA1 PE=1 SV=3 - [OPA1_ | 3401948 | 3313066 | 530660 | 6,41 | 0,231308 | 6,24 | 0,032267 | 1,03 | 0,970787 |
| Q86Y56 | Dynein assembly factor 5, axonemal OS=Homo sapiens GN=DNAAF5 PE=1 SV=4 - [DAAF5_HUM | 11378 | 6619 | 1811 | 6,28 | 0,521994 | 3,66 | 0,616734 | 1,72 | 0,724652 |
| Q9P2D7 | Dynein heavy chain 1, axonemal OS=Homo sapiens GN=DNAH1 PE=2 SV=4 - [DYH1_HUMAN] | 5038014 | 4625391 | 4017943 | 1,25 | 0,514976 | 1,15 | 0,673058 | 1,09 | 0,772596 |
| Q96DT5 | Dynein heavy chain 11, axonemal OS=Homo sapiens GN=DNAH11 PE=1 SV=4 - [DYH11_HUMA | 6820149 | 10184163 | 268176 | 25,43 | 0,009634 | 37,98 | 0,001441 | 0,67 | 0,141547 |
| Q9UFH2 | Dynein heavy chain 17, axonemal OS=Homo sapiens GN=DNAH17 PE=1 SV=2 - [DYH17_HUMAI | 238653 | 19061 | 43965 | 5,43 | 0,165625 | 0,43 | 0,293261 | 12,52 | 0,007614 |
| Q9P225 | Dynein heavy chain 2, axonemal OS=Homo sapiens GN=DNAH2 PE=2 SV=3 - [DYH2_HUMAN] | 12402 | 24498 | 0 | #DIV/0! | 0,392996 | #DIV/0! | 0,357392 | 0,51 | 0,571469 |
| Q8TE73 | Dynein heavy chain 5, axonemal OS=Homo sapiens GN=DNAH5 PE=1 SV=3 - [DYH5_HUMAN] | 237656 | 275463 | 259089 | 0,92 | 0,865407 | 1,06 | 0,896603 | 0,86 | 0,749905 |

| | | | | | | | | | | |
|---|---|---|---|---|---|---|---|---|---|---|
| Q9C0G6 | Dynein heavy chain 6, axonemal OS=Homo sapiens GN=DNAH6 PE=2 SV=3 - [DYH6_HUMAN] | 2355682 | 2579159 | 2815288 | 0,84 | 0,512361 | 0,92 | 0,724397 | 0,91 | 0,743111 |
| Q8WXX0 | Dynein heavy chain 7, axonemal OS=Homo sapiens GN=DNAH7 PE=1 SV=2 - [DYH7_HUMAN] | 19235 | 51686 | 108 | 177,98 | 0,298847 | 478,26 | 0,106318 | 0,37 | 0,147316 |
| Q96M86 | Dynein heavy chain domain-containing protein 1 OS=Homo sapiens GN=DNHD1 PE=2 SV=2 - [D | 369697 | 315784 | 657439 | 0,56 | 0,534602 | 0,48 | 0,198703 | 1,17 | 0,900556 |
| Q9H069 | Dynein regulatory complex subunit 3 OS=Homo sapiens GN=DRC3 PE=2 SV=2 - [DRC3_HUMAN | 320216 | 61954 | 84342 | 3,80 | 0,251404 | 0,73 | 0,767941 | 5,17 | 0,048114 |
| Q03001 | Dystonin OS=Homo sapiens GN=DST PE=1 SV=4 - [DYST_HUMAN] | 145116 | 145958 | 8743 | 16,60 | 0,155702 | 16,69 | 0,28096 | 0,99 | 0,997748 |
| Q14118 | Dystroglycan OS=Homo sapiens GN=DAG1 PE=1 SV=2 - [DAG1_HUMAN] | 1339990 | 1293464 | 1585110 | 0,85 | 0,558367 | 0,82 | 0,321008 | 1,04 | 0,919815 |
| Q14258 | E3 ubiquitin/ISG15 ligase TRIM25 OS=Homo sapiens GN=TRIM25 PE=1 SV=2 - [TRI25_HUMAN] | 1052369 | 1442301 | 413691 | 2,54 | 0,422344 | 3,49 | 0,065603 | 0,73 | 0,571469 |
| O75150 | E3 ubiquitin-protein ligase BRE1B OS=Homo sapiens GN=RNF40 PE=1 SV=4 - [BRE1B_HUMAN] | 485860 | 748400 | 308573 | 1,57 | 0,211336 | 2,43 | 0,096029 | 0,65 | 0,141547 |
| Q8TDB6 | E3 ubiquitin-protein ligase DTX3L OS=Homo sapiens GN=DTX3L PE=1 SV=1 - [DTX3L_HUMAN] | 17005 | 53022 | 18135 | 0,94 | 0,93824 | 2,92 | 0,039722 | 0,32 | 0,000885 |
| Q96J02 | E3 ubiquitin-protein ligase Itchy homolog OS=Homo sapiens GN=ITCH PE=1 SV=2 - [ITCH_HUM | 834986 | 888614 | 796309 | 1,05 | 0,856068 | 1,12 | 0,545806 | 0,94 | 0,777346 |
| Q9HCE7 | E3 ubiquitin-protein ligase SMURF1 OS=Homo sapiens GN=SMURF1 PE=1 SV=2 - [SMUF1_HUM | 33828231 | 36811710 | 15473308 | 2,19 | 0,03762 | 2,38 | 0,015551 | 0,92 | 0,743237 |
| P53804 | E3 ubiquitin-protein ligase TTC3 OS=Homo sapiens GN=TTC3 PE=1 SV=2 - [TTC3_HUMAN] | 1747013 | 616662 | 1896409 | 0,92 | 0,882754 | 0,33 | 0,03902 | 2,83 | 0,068072 |
| Q9HC35 | Echinoderm microtubule-associated protein-like 4 OS=Homo sapiens GN=EML4 PE=1 SV=3 - [E | 8637339 | 17332211 | 10119597 | 0,85 | 0,752097 | 1,71 | 0,011607 | 0,50 | 0,003713 |
| Q13822 | Ectonucleotide pyrophosphatase/phosphodiesterase family member 2 OS=Homo sapiens GN= | 22141292 | 27623781 | 14481310 | 1,53 | 0,191881 | 1,91 | 0,042859 | 0,80 | 0,276928 |
| Q9HCE0 | Ectopic P granules protein 5 homolog OS=Homo sapiens GN=EPG5 PE=2 SV=2 - [EPG5_HUMAN | 13662 | 1372 | 0 | #DIV/0! | 0,635951 | #DIV/0! | 0,433696 | 9,96 | 0,549834 |
| A8K855 | EF-hand calcium-binding domain-containing protein 7 OS=Homo sapiens GN=EFCAB7 PE=2 SV= | 582342 | 329389 | 242691 | 2,40 | 0,522865 | 1,36 | 0,674738 | 1,77 | 0,520082 |
| Q12805 | EGF-containing fibulin-like extracellular matrix protein 1 OS=Homo sapiens GN=EFEMP1 PE=1 | 36172522 | 64307594 | 41767332 | 0,87 | 0,69131 | 1,54 | 0,002239 | 0,56 | 0,001315 |
| O95967 | EGF-containing fibulin-like extracellular matrix protein 2 OS=Homo sapiens GN=EFEMP2 PE=1 | 11191 | 19770 | 3532 | 3,17 | 0,514976 | 5,60 | 0,051025 | 0,57 | 0,363594 |
| P13639 | Elongation factor 2 OS=Homo sapiens GN=EEF2 PE=1 SV=4 - [EF2_HUMAN] | 29658 | 2886 | 25703 | 1,15 | 0,932619 | 0,11 | 0,076833 | 10,28 | 0,346599 |
| Q9H2F5 | Enhancer of polycomb homolog 1 OS=Homo sapiens GN=EPC1 PE=1 SV=1 - [EPC1_HUMAN] | 612308 | 519246 | 2115838 | 0,29 | 0,00404 | 0,25 | 0,003375 | 1,18 | 0,760808 |
| P54756 | Ephrin type-A receptor 5 OS=Homo sapiens GN=EPHA5 PE=1 SV=3 - [EPHA5_HUMAN] | 91257544 | 21631146 | 23743593 | 3,84 | 0,030748 | 0,91 | 0,72299 | 4,22 | 0,000147 |
| Q9UF33 | Ephrin type-A receptor 6 OS=Homo sapiens GN=EPHA6 PE=2 SV=3 - [EPHA6_HUMAN] | 3673786 | 9009759 | 14200826 | 0,26 | 1,56E-06 | 0,63 | 0,13797 | 0,41 | 0,011134 |
| P61916 | Epididymal secretory protein E1 OS=Homo sapiens GN=NPC2 PE=1 SV=1 - [NPC2_HUMAN] | 676895 | 493724 | 282859 | 2,39 | 0,387214 | 1,75 | 0,336192 | 1,37 | 0,615518 |
| P58107 | Epiplakin OS=Homo sapiens GN=EPPK1 PE=1 SV=2 - [EPIPL_HUMAN] | 18463802 | 20499266 | 778990 | 23,70 | 0,026138 | 26,32 | 0,009916 | 0,90 | 0,805932 |
| Q92611 | ER degradation-enhancing alpha-mannosidase-like protein 1 OS=Homo sapiens GN=EDEM1 PE | 2736914 | 1992962 | 2933501 | 0,93 | 0,759705 | 0,68 | 0,023624 | 1,37 | 0,101656 |
| Q96HE7 | ERO1-like protein alpha OS=Homo sapiens GN=ERO1A PE=1 SV=2 - [ERO1A_HUMAN] | 3003385 | 3396555 | 794614 | 3,78 | 0,031802 | 4,27 | 0,00024 | 0,88 | 0,64579 |
| Q14152 | Eukaryotic translation initiation factor 3 subunit A OS=Homo sapiens GN=EIF3A PE=1 SV=1 - [EI | 150943 | 205299 | 5245 | 28,78 | 0,103602 | 39,14 | 0,015575 | 0,74 | 0,507382 |
| A0FGR9 | Extended synaptotagmin-3 OS=Homo sapiens GN=ESYT3 PE=1 SV=1 - [ESYT3_HUMAN] | 656600 | 125457 | 94220 | 6,97 | 0,11322 | 1,33 | 0,545623 | 5,23 | 0,011339 |
| Q16610 | Extracellular matrix protein 1 OS=Homo sapiens GN=ECM1 PE=1 SV=2 - [ECM1_HUMAN] | 798960 | 689701 | 714087 | 1,12 | 0,788088 | 0,97 | 0,934819 | 1,16 | 0,693974 |
| Q8IWU5 | Extracellular sulfatase Sulf-2 OS=Homo sapiens GN=SULF2 PE=1 SV=1 - [SULF2_HUMAN] | 40115 | 36858 | 14171 | 2,83 | 0,204477 | 2,60 | 0,148659 | 1,09 | 0,8803 |
| P08294 | Extracellular superoxide dismutase [Cu-Zn] OS=Homo sapiens GN=SOD3 PE=1 SV=2 - [SODE_HU | 745189 | 877340 | 55691 | 13,38 | 0,095154 | 15,75 | 0,0374 | 0,85 | 0,760808 |
| Q9Y2M0 | Fanconi-associated nuclease 1 OS=Homo sapiens GN=FAN1 PE=1 SV=4 - [FAN1_HUMAN] | 920769 | 1474039 | 527565 | 1,75 | 0,358017 | 2,79 | 0,069475 | 0,62 | 0,161102 |
| Q01469 | Fatty acid-binding protein, epidermal OS=Homo sapiens GN=FABP5 PE=1 SV=3 - [FABP5_HUMA | 13437 | 61854 | 60514 | 0,22 | 0,002686 | 1,02 | 0,987728 | 0,22 | 0,42591 |
| Q8TF61 | F-box only protein 41 OS=Homo sapiens GN=FBXO41 PE=2 SV=5 - [FBX41_HUMAN] | 3949261 | 5179783 | 3545165 | 1,11 | 0,854571 | 1,46 | 0,620224 | 0,76 | 0,657758 |
| Q9UKT6 | F-box/LRR-repeat protein 21 OS=Homo sapiens GN=FBXL21 PE=1 SV=1 - [FXL21_HUMAN] | 645639 | 637389 | 426544 | 1,51 | 0,209923 | 1,49 | 0,186418 | 1,01 | 0,963841 |
| A0AVI2 | Fer-1-like protein 5 OS=Homo sapiens GN=FER1L5 PE=2 SV=2 - [FR1L5_HUMAN] | 24866 | 3771 | 0 | #DIV/0! | 0,105191 | #DIV/0! | 0,342339 | 6,59 | 0,022848 |
| Q2WGJ9 | Fer-1-like protein 6 OS=Homo sapiens GN=FER1L6 PE=2 SV=2 - [FR1L6_HUMAN] | 139366498 | 165153416 | 275816956 | 0,51 | 0,005908 | 0,60 | 0,00447 | 0,84 | 0,539437 |
| Q9UGM5 | Fetuin-B OS=Homo sapiens GN=FETUB PE=1 SV=2 - [FETUB_HUMAN] | 347893 | 455885 | 1163765 | 0,30 | 0,00061 | 0,39 | 0,02689 | 0,76 | 0,66165 |
| P35555 | Fibrillin-1 OS=Homo sapiens GN=FBN1 PE=1 SV=3 - [FBN1_HUMAN] | 430451 | 334503 | 134917 | 3,19 | 0,092659 | 2,48 | 0,030508 | 1,29 | 0,442915 |
| P02671 | Fibrinogen alpha chain OS=Homo sapiens GN=FGA PE=1 SV=2 - [FIBA_HUMAN] | 4192344 | 3546865 | 14904251 | 0,28 | 0,006816 | 0,24 | 0,007868 | 1,18 | 0,513622 |
| P02675 | Fibrinogen beta chain OS=Homo sapiens GN=FGB PE=1 SV=2 - [FIBB_HUMAN] | 6745870 | 5032237 | 31952949 | 0,21 | 0,004849 | 0,16 | 0,004471 | 1,34 | 0,503117 |
| P02679 | Fibrinogen gamma chain OS=Homo sapiens GN=FGG PE=1 SV=3 - [FIBG_HUMAN] | 19790041 | 11616118 | 42118814 | 0,47 | 0,027159 | 0,28 | 0,003864 | 1,70 | 0,015453 |
| Q9BYJ0 | Fibroblast growth factor-binding protein 2 OS=Homo sapiens GN=FGFBP2 PE=1 SV=1 - [FGFP2_ | 90311 | 135714 | 479261 | 0,19 | 0,000979 | 0,28 | 0,011584 | 0,67 | 0,314301 |
| Q86WI1 | Fibrocystin-L OS=Homo sapiens GN=PKHD1L1 PE=2 SV=2 - [PKHL1_HUMAN] | 4713766 | 1734585 | 1630598 | 2,89 | 0,106501 | 1,06 | 0,842 | 2,72 | 0,009544 |
| P02751 | Fibronectin OS=Homo sapiens GN=FN1 PE=1 SV=4 - [FINC_HUMAN] | 36573293 | 65555579 | 119838606 | 0,31 | 4,1E-06 | 0,55 | 0,000357 | 0,56 | 0,000907 |
| P23142 | Fibulin-1 OS=Homo sapiens GN=FBLN1 PE=1 SV=4 - [FBLN1_HUMAN] | 13490222 | 14484282 | 11614748 | 1,16 | 0,384591 | 1,25 | 0,129142 | 0,93 | 0,607378 |
| Q53RD9 | Fibulin-7 OS=Homo sapiens GN=FBLN7 PE=2 SV=1 - [FBLN7_HUMAN] | 26774512 | 5019928 | 9828165 | 2,72 | 0,199006 | 0,51 | 0,045101 | 5,33 | 0,00513 |
| Q12841 | Follistatin-related protein 1 OS=Homo sapiens GN=FSTL1 PE=1 SV=1 - [FSTL1_HUMAN] | 26043744 | 51648101 | 100742484 | 0,26 | 5,79E-05 | 0,51 | 2,23E-05 | 0,50 | 0,013693 |
| Q6MZW2 | Follistatin-related protein 4 OS=Homo sapiens GN=FSTL4 PE=2 SV=3 - [FSTL4_HUMAN] | 4692216 | 4636784 | 7891122 | 0,59 | 0,101518 | 0,59 | 0,085094 | 1,01 | 0,980727 |
| Q8N475 | Follistatin-related protein 5 OS=Homo sapiens GN=FSTL5 PE=2 SV=2 - [FSTL5_HUMAN] | 2270960 | 1894820 | 1412356 | 1,61 | 0,246555 | 1,34 | 0,461805 | 1,20 | 0,570405 |
| Q9H334 | Forkhead box protein P1 OS=Homo sapiens GN=FOXP1 PE=1 SV=1 - [FOXP1_HUMAN] | 229034 | 339629 | 315072 | 0,73 | 0,518508 | 1,08 | 0,903143 | 0,67 | 0,437937 |
| B1AJZ9 | Forkhead-associated domain-containing protein 1 OS=Homo sapiens GN=FHAD1 PE=2 SV=2 - [ | 359254 | 396628 | 270845 | 1,33 | 0,737246 | 1,46 | 0,453273 | 0,91 | 0,870819 |
| Q96PY5 | Formin-like protein 2 OS=Homo sapiens GN=FMNL2 PE=1 SV=3 - [FMNL2_HUMAN] | 1752579 | 1173551 | 4181980 | 0,42 | 0,000353 | 0,28 | 7,25E-07 | 1,49 | 0,207137 |
| P48051 | G protein-activated inward rectifier potassium channel 2 OS=Homo sapiens GN=KCNJ6 PE=1 S | 539608 | 1381760 | 741909 | 0,73 | 0,514871 | 1,86 | 0,015727 | 0,39 | 0,000169 |
| Q08380 | Galectin-3-binding protein OS=Homo sapiens GN=LGALS3BP PE=1 SV=1 - [LG3BP_HUMAN] | 849867 | 1050466 | 765718 | 1,11 | 0,805725 | 1,37 | 0,334987 | 0,81 | 0,425918 |
| P17900 | Ganglioside GM2 activator OS=Homo sapiens GN=GM2A PE=1 SV=4 - [SAP3_HUMAN] | 54820 | 51668 | 13525 | 4,05 | 0,372845 | 3,82 | 0,113937 | 1,06 | 0,944831 |
| P17302 | Gap junction alpha-1 protein OS=Homo sapiens GN=GJA1 PE=1 SV=2 - [CXA1_HUMAN] | 2137551 | 1995230 | 1298599 | 1,65 | 0,049618 | 1,54 | 0,072074 | 1,07 | 0,739178 |
| Q13630 | GDP-L-fucose synthase OS=Homo sapiens GN=TSTA3 PE=1 SV=1 - [FCL_HUMAN] | 1112686 | 1750635 | 2753773 | 0,40 | 0,004518 | 0,64 | 0,178653 | 0,64 | 0,275429 |
| P06396 | Gelsolin OS=Homo sapiens GN=GSN PE=1 SV=1 - [GELS_HUMAN] | 95647566 | 75151612 | 86612186 | 1,10 | 0,537964 | 0,87 | 0,140258 | 1,27 | 0,01939 |
| P57678 | Gem-associated protein 4 OS=Homo sapiens GN=GEMIN4 PE=1 SV=2 - [GEMI4_HUMAN] | 18744 | 8573 | 29633 | 0,63 | 0,645892 | 0,29 | 0,143651 | 2,19 | 0,483721 |
| P14136 | Glial fibrillary acidic protein OS=Homo sapiens GN=GFAP PE=1 SV=1 - [GFAP_HUMAN] | 29167 | 4901 | 1094 | 26,66 | 0,614287 | 4,48 | 0,498231 | 5,95 | 0,538047 |
| P14314 | Glucosidase 2 subunit beta OS=Homo sapiens GN=PRKCSH PE=1 SV=2 - [GLU2B_HUMAN] | 20016 | 35348 | 4922 | 4,07 | 0,257174 | 7,18 | 0,226373 | 0,57 | 0,422389 |
| P49448 | Glutamate dehydrogenase 2, mitochondrial OS=Homo sapiens GN=GLUD2 PE=1 SV=2 - [DHE4_ | 178367 | 28595 | 188552 | 0,95 | 0,932619 | 0,15 | 0,000203 | 6,24 | 0,017175 |
| P42262 | Glutamate receptor 2 OS=Homo sapiens GN=GRIA2 PE=1 SV=3 - [GRIA2_HUMAN] | 171661 | 117243 | 561970 | 0,31 | 0,00077 | 0,21 | 9,09E-05 | 1,46 | 0,449275 |
| P48058 | Glutamate receptor 4 OS=Homo sapiens GN=GRIA4 PE=2 SV=2 - [GRIA4_HUMAN] | 94796 | 132926 | 61122 | 1,55 | 0,535422 | 2,17 | 0,214467 | 0,71 | 0,445197 |
| Q2KHR3 | Glutamine and serine-rich protein 1 OS=Homo sapiens GN=QSER1 PE=1 SV=3 - [QSER1_HUMA | 3964 | 25087 | 30691 | 0,13 | 0,058268 | 0,82 | 0,766922 | 0,16 | 0,002618 |
| Q16769 | Glutaminyl-peptide cyclotransferase OS=Homo sapiens GN=QPCT PE=1 SV=1 - [QPCT_HUMAN | 718616 | 842885 | 135722 | 5,29 | 0,056003 | 6,21 | 0,001833 | 0,85 | 0,64015 |
| A6NFK2 | Glutaredoxin domain-containing cysteine-rich protein 2 OS=Homo sapiens GN=GRXCR2 PE=3 S | 5769930 | 6080115 | 664832 | 8,68 | 0,050096 | 9,15 | 0,000714 | 0,95 | 0,903565 |
| P22352 | Glutathione peroxidase 3 OS=Homo sapiens GN=GPX3 PE=1 SV=2 - [GPX3_HUMAN] | 48637707 | 51872238 | 27560264 | 1,76 | 0,00446 | 1,88 | 0,002196 | 0,94 | 0,651698 |
| P09488 | Glutathione S-transferase Mu 1 OS=Homo sapiens GN=GSTM1 PE=1 SV=3 - [GSTM1_HUMAN] | 508262 | 1334508 | 1834704 | 0,28 | 0,000339 | 0,73 | 0,171578 | 0,38 | 0,002169 |
| P04406 | Glyceraldehyde-3-phosphate dehydrogenase OS=Homo sapiens GN=GAPDH PE=1 SV=3 - [G3P_ | 512691 | 376446 | 335463 | 1,53 | 0,346397 | 1,12 | 0,720079 | 1,36 | 0,296746 |
| Q6IB77 | Glycine N-acyltransferase OS=Homo sapiens GN=GLYAT PE=1 SV=3 - [GLYAT_HUMAN] | 1240980 | 1561830 | 281035 | 4,42 | 0,116696 | 5,56 | 0,015197 | 0,79 | 0,56951 |
| P06737 | Glycogen phosphorylase, liver form OS=Homo sapiens GN=PYGL PE=1 SV=4 - [PYGL_HUMAN] | 1458662 | 2623132 | 79771 | 18,29 | 0,096672 | 32,88 | 0,017724 | 0,56 | 0,145114 |
| O75063 | Glycosaminoglycan xylosylkinase OS=Homo sapiens GN=FAM20B PE=1 SV=1 - [XYLK_HUMAN] | 854683 | 290223 | 48227 | 17,72 | 0,037714 | 6,02 | 0,029527 | 2,94 | 0,014852 |
| O43292 | Glycosylphosphatidylinositol anchor attachment 1 protein OS=Homo sapiens GN=GPAA1 PE=1 | 3655649 | 2033784 | 1161047 | 3,15 | 0,068372 | 1,75 | 0,151012 | 1,80 | 0,072763 |
| O00461 | Golgi integral membrane protein 4 OS=Homo sapiens GN=GOLIM4 PE=1 SV=1 - [GOLI4_HUMA | 22449 | 17920 | 0 | #DIV/0! | 0,422118 | #DIV/0! | 0,20127 | 1,25 | 0,862436 |
| Q8NBJ4 | Golgi membrane protein 1 OS=Homo sapiens GN=GOLM1 PE=1 SV=1 - [GOLM1_HUMAN] | 50169 | 45546 | 17092 | 2,94 | 0,259201 | 2,66 | 0,228997 | 1,10 | 0,887972 |
| Q86SQ4 | G-protein coupled receptor 126 OS=Homo sapiens GN=GPR126 PE=1 SV=3 - [GP126_HUMAN] | 173406 | 91246 | 7430 | 23,34 | 0,028703 | 12,28 | 0,026131 | 1,90 | 0,107863 |
| Q6ISB3 | Grainyhead-like protein 2 homolog OS=Homo sapiens GN=GRHL2 PE=1 SV=1 - [GRHL2_HUMAI | 21906 | 17028 | 0 | #DIV/0! | 0,265232 | #DIV/0! | 0,151012 | 1,29 | 0,785156 |
| Q9H706 | GRB2-associated and regulator of MAPK protein 1 OS=Homo sapiens GN=GAREM1 PE=1 SV=2 - | 308120 | 115941 | 1174665 | 0,26 | 0,01209 | 0,10 | 0,001292 | 2,66 | 0,161102 |
| Q8IWJ2 | GRIP and coiled-coil domain-containing protein 2 OS=Homo sapiens GN=GCC2 PE=1 SV=4 - [G | 155456 | 440735 | 38288 | 4,06 | 0,133411 | 11,51 | 0,188248 | 0,35 | 0,137949 |
| Q4V328 | GRIP1-associated protein 1 OS=Homo sapiens GN=GRIPAP1 PE=1 SV=1 - [GRAP1_HUMAN] | 478930 | 668736 | 1100143 | 0,44 | 0,231165 | 0,61 | 0,555511 | 0,72 | 0,71381 |
| O95995 | Growth arrest-specific protein 8 OS=Homo sapiens GN=GAS8 PE=1 SV=1 - [GAS8_HUMAN] | 20500 | 21551 | 126280 | 0,16 | 0,00553 | 0,17 | 0,007222 | 0,95 | 0,962787 |
| P62873 | Guanine nucleotide-binding protein G(I)/G(S)/G(T) subunit beta-1 OS=Homo sapiens GN=GNB1 | 2581 | 667 | 86134 | 0,03 | 0,033898 | 0,01 | 0,04096 | 3,87 | 0,291309 |
| P11488 | Guanine nucleotide-binding protein G(t) subunit alpha-1 OS=Homo sapiens GN=GNAT1 PE=1 S | 210 | 0 | 75750 | 0,00 | 0,130816 | 0,00 | 0,143651 | #DIV/0! | 0,590084 |
| P00738 | Haptoglobin OS=Homo sapiens GN=HP PE=1 SV=1 - [HPT_HUMAN] | 36621369 | 29300130 | 60237349 | 0,61 | 0,266739 | 0,49 | 0,136563 | 1,25 | 0,693485 |
| P00739 | Haptoglobin-related protein OS=Homo sapiens GN=HPR PE=2 SV=2 - [HPTR_HUMAN] | 52245326 | 58806772 | 41841515 | 1,25 | 0,600897 | 1,41 | 0,371433 | 0,89 | 0,743111 |
| P48723 | Heat shock 70 kDa protein 13 OS=Homo sapiens GN=HSPA13 PE=1 SV=1 - [HSP13_HUMAN] | 60357271 | 49144727 | 37689379 | 1,60 | 0,199098 | 1,30 | 0,328244 | 1,23 | 0,43145 |
| P25092 | Heat-stable enterotoxin receptor OS=Homo sapiens GN=GUCY2C PE=1 SV=2 - [GUC2C_HUMAI | 16467404 | 29803585 | 47667557 | 0,35 | 0,000195 | 0,63 | 0,014665 | 0,55 | 0,007448 |
| P69905 | Hemoglobin subunit alpha OS=Homo sapiens GN=HBA1 PE=1 SV=2 - [HBA_HUMAN] | 1216046 | 376605 | 42122572 | 0,03 | 0,023614 | 0,01 | 0,029527 | 3,23 | 0,263928 |
| P68871 | Hemoglobin subunit beta OS=Homo sapiens GN=HBB PE=1 SV=2 - [HBB_HUMAN] | 4893086 | 3601152 | 188088670 | 0,03 | 0,032231 | 0,02 | 0,042222 | 1,36 | 0,685574 |
| P02042 | Hemoglobin subunit delta OS=Homo sapiens GN=HBD PE=1 SV=2 - [HBD_HUMAN] | 2338527 | 1399607 | 1754498 | 1,33 | 0,52784 | 0,80 | 0,662949 | 1,67 | 0,009458 |
| P02100 | Hemoglobin subunit epsilon OS=Homo sapiens GN=HBE1 PE=1 SV=2 - [HBE_HUMAN] | 512854 | 321954 | 181279 | 2,83 | 0,062368 | 1,78 | 0,009081 | 1,59 | 0,086134 |
| P02008 | Hemoglobin subunit zeta OS=Homo sapiens GN=HBZ PE=1 SV=2 - [HBAZ_HUMAN] | 31284 | 0 | 13571 | 2,31 | 0,743866 | 0,00 | 0,143651 | #DIV/0! | 0,401568 |
| P02790 | Hemopexin OS=Homo sapiens GN=HPX PE=1 SV=2 - [HEMO_HUMAN] | 983216173 | 894860945 | 1208769160 | 0,81 | 0,293215 | 0,74 | 0,085094 | 1,10 | 0,66165 |
| P05546 | Heparin cofactor 2 OS=Homo sapiens GN=SERPIND1 PE=1 SV=3 - [HEP2_HUMAN] | 2479759 | 2434448 | 1713203 | 1,45 | 0,279241 | 1,42 | 0,395757 | 1,02 | 0,962787 |
| P12314 | High affinity immunoglobulin gamma Fc receptor I OS=Homo sapiens GN=FCGR1A PE=1 SV=2 - | 179341 | 212474 | 234950 | 0,76 | 0,540904 | 0,90 | 0,720079 | 0,84 | 0,664729 |
| P04196 | Histidine-rich glycoprotein OS=Homo sapiens GN=HRG PE=1 SV=1 - [HRG_HUMAN] | 30531431 | 24688178 | 33155577 | 0,92 | 0,69131 | 0,74 | 0,114676 | 1,24 | 0,267236 |
| Q92831 | Histone acetyltransferase KAT2B OS=Homo sapiens GN=KAT2B PE=1 SV=3 - [KAT2B_HUMAN] | 756045 | 1122715 | 895810 | 0,84 | 0,698175 | 1,25 | 0,512499 | 0,67 | 0,167299 |
| Q92794 | Histone acetyltransferase KAT6A OS=Homo sapiens GN=KAT6A PE=1 SV=2 - [KAT6A_HUMAN] | 1193352 | 1502010 | 956916 | 1,25 | 0,502589 | 1,57 | 0,137718 | 0,79 | 0,346062 |

| | | | | | | | | | | |
|---|---|---|---|---|---|---|---|---|---|---|
| O95251 | Histone acetyltransferase KAT7 OS=Homo sapiens GN=KAT7 PE=1 SV=1 - [KAT7_HUMAN] | 4083350 | 3818117 | 9603053 | 0,43 | 0,001242 | 0,40 | 3,9E-05 | 1,07 | 0,854632 |
| Q9UBN7 | Histone deacetylase 6 OS=Homo sapiens GN=HDAC6 PE=1 SV=2 - [HDAC6_HUMAN] | 1339885 | 3450512 | 32645 | 41,04 | 0,238767 | 105,70 | 0,010546 | 0,39 | 0,028927 |
| O14686 | Histone-lysine N-methyltransferase 2D OS=Homo sapiens GN=KMT2D PE=1 SV=2 - [KMT2D_HU | 72642 | 143334 | 0 | #DIV/0! | 0,210723 | #DIV/0! | 0,041864 | 0,51 | 0,21111 |
| Q96KQ7 | Histone-lysine N-methyltransferase EHMT2 OS=Homo sapiens GN=EHMT2 PE=1 SV=3 - [EHMT | 251292 | 197062 | 219659 | 1,14 | 0,828097 | 0,90 | 0,800643 | 1,28 | 0,642974 |
| O96028 | Histone-lysine N-methyltransferase NSD2 OS=Homo sapiens GN=WHSC1 PE=1 SV=1 - [NSD2_H | 590717 | 991269 | 302018 | 1,96 | 0,20158 | 3,28 | 0,001353 | 0,60 | 0,01314 |
| Q9BZ95 | Histone-lysine N-methyltransferase NSD3 OS=Homo sapiens GN=WHSC1L1 PE=1 SV=1 - [NSD3 | 60091 | 59374 | 5913 | 10,16 | 0,233807 | 10,04 | 0,176439 | 1,01 | 0,9919 |
| Q9UPS6 | Histone-lysine N-methyltransferase SETD1B OS=Homo sapiens GN=SETD1B PE=1 SV=3 - [SET1B | 466084 | 735319 | 16659 | 27,98 | 0,033898 | 44,14 | 0,003377 | 0,63 | 0,154471 |
| Q9BYW2 | Histone-lysine N-methyltransferase SETD2 OS=Homo sapiens GN=SETD2 PE=1 SV=3 - [SETD2_H | 2729824 | 6245095 | 20354142 | 0,13 | 4,32E-08 | 0,31 | 6,23E-07 | 0,44 | 0,008106 |
| Q00056 | Homeobox protein Hox-A4 OS=Homo sapiens GN=HOXA4 PE=2 SV=3 - [HXA4_HUMAN] | 93728 | 189509 | 2707 | 34,62 | 0,064755 | 69,99 | 0,001206 | 0,49 | 0,017246 |
| Q86YZ3 | Hornerin OS=Homo sapiens GN=HRNR PE=1 SV=2 - [HORN_HUMAN] | 9922 | 12198 | 10162 | 0,98 | 0,987305 | 1,20 | 0,770801 | 0,81 | 0,854632 |
| Q14520 | Hyaluronan-binding protein 2 OS=Homo sapiens GN=HABP2 PE=1 SV=1 - [HABP2_HUMAN] | 104468 | 159481 | 101075 | 1,03 | 0,90167 | 1,58 | 0,068147 | 0,66 | 0,002169 |
| P22304 | Iduronate 2-sulfatase OS=Homo sapiens GN=IDS PE=1 SV=1 - [IDS_HUMAN] | 276940 | 340803 | 52376 | 5,29 | 0,095154 | 6,51 | 0,048703 | 0,81 | 0,651307 |
| P01876 | Ig alpha-1 chain C region OS=Homo sapiens GN=IGHA1 PE=1 SV=2 - [IGHA1_HUMAN] | 36542909 | 48083717 | 104616219 | 0,35 | 0,000679 | 0,46 | 0,029527 | 0,76 | 0,523485 |
| P01877 | Ig alpha-2 chain C region OS=Homo sapiens GN=IGHA2 PE=1 SV=3 - [IGHA2_HUMAN] | 92959913 | 127376309 | 286502854 | 0,32 | 0,014871 | 0,44 | 0,070228 | 0,73 | 0,615518 |
| P01857 | Ig gamma-1 chain C region OS=Homo sapiens GN=IGHG1 PE=1 SV=1 - [IGHG1_HUMAN] | 661438811 | 679699726 | 523450657 | 1,26 | 0,63163 | 1,30 | 0,125709 | 0,97 | 0,944831 |
| P01859 | Ig gamma-2 chain C region OS=Homo sapiens GN=IGHG2 PE=1 SV=2 - [IGHG2_HUMAN] | 61518052 | 110977664 | 93466100 | 0,66 | 0,00993 | 1,19 | 0,542514 | 0,55 | 0,00291 |
| P01860 | Ig gamma-3 chain C region OS=Homo sapiens GN=IGHG3 PE=1 SV=2 - [IGHG3_HUMAN] | 11235699 | 7967563 | 7360173 | 1,53 | 0,475059 | 1,08 | 0,770437 | 1,41 | 0,39244 |
| P01861 | Ig gamma-4 chain C region OS=Homo sapiens GN=IGHG4 PE=1 SV=1 - [IGHG4_HUMAN] | 35134886 | 32102723 | 43063919 | 0,82 | 0,582836 | 0,75 | 0,292056 | 1,09 | 0,830077 |
| P01764 | Ig heavy chain V-III region 23 OS=Homo sapiens GN=IGHV3-23 PE=1 SV=2 - [HV303_HUMAN] | 117424 | 214392 | 655346 | 0,18 | 5,9E-09 | 0,33 | 0,000398 | 0,55 | 0,097725 |
| P01766 | Ig heavy chain V-III region BRO OS=Homo sapiens PE=1 SV=1 - [HV305_HUMAN] | 2532771 | 4532643 | 3644537 | 0,69 | 0,133411 | 1,24 | 0,548804 | 0,56 | 0,017574 |
| P01781 | Ig heavy chain V-III region GAL OS=Homo sapiens PE=1 SV=1 - [HV320_HUMAN] | 153027 | 159202 | 224121 | 0,68 | 0,634554 | 0,71 | 0,542444 | 0,96 | 0,962787 |
| P01765 | Ig heavy chain V-III region TIL OS=Homo sapiens PE=1 SV=1 - [HV304_HUMAN] | 2224265 | 2701980 | 3821914 | 0,58 | 0,216018 | 0,71 | 0,433648 | 0,82 | 0,705633 |
| P01762 | Ig heavy chain V-III region TRO OS=Homo sapiens PE=1 SV=1 - [HV301_HUMAN] | 45855 | 53609 | 129 | 355,44 | 0,476677 | 415,55 | 0,128059 | 0,86 | 0,903097 |
| P01763 | Ig heavy chain V-III region WEA OS=Homo sapiens PE=1 SV=1 - [HV302_HUMAN] | 195198 | 128735 | 42588 | 4,58 | 0,046075 | 3,02 | 0,101699 | 1,52 | 0,253714 |
| P01834 | Ig kappa chain C region OS=Homo sapiens GN=IGKC PE=1 SV=1 - [IGKC_HUMAN] | 137664642 | 151595684 | 158179787 | 0,87 | 0,698175 | 0,96 | 0,798656 | 0,91 | 0,760808 |
| P01612 | Ig kappa chain V-I region Mev OS=Homo sapiens PE=1 SV=1 - [KV120_HUMAN] | 252711 | 322957 | 422387 | 0,60 | 0,554941 | 0,76 | 0,250212 | 0,78 | 0,760808 |
| P01611 | Ig kappa chain V-I region Wes OS=Homo sapiens PE=1 SV=1 - [KV119_HUMAN] | 531439 | 1018899 | 2698117 | 0,20 | 0,000353 | 0,38 | 0,005986 | 0,52 | 0,331604 |
| P01614 | Ig kappa chain V-II region Cum OS=Homo sapiens PE=1 SV=1 - [KV201_HUMAN] | 1426651 | 1997231 | 1645348 | 0,87 | 0,73707 | 1,21 | 0,408184 | 0,71 | 0,243466 |
| P06310 | Ig kappa chain V-II region RPMI 6410 OS=Homo sapiens PE=4 SV=1 - [KV206_HUMAN] | 15216 | 5358 | 50073 | 0,30 | 0,104504 | 0,11 | 0,032261 | 2,84 | 0,124086 |
| P01622 | Ig kappa chain V-III region Ti OS=Homo sapiens PE=1 SV=1 - [KV304_HUMAN] | 5117978 | 4809507 | 7231463 | 0,71 | 0,133617 | 0,67 | 0,014738 | 1,06 | 0,808152 |
| P04433 | Ig kappa chain V-III region VG (Fragment) OS=Homo sapiens PE=1 SV=1 - [KV309_HUMAN] | 794265 | 831065 | 361783 | 2,20 | 0,645892 | 2,30 | 0,112223 | 0,96 | 0,963841 |
| P06312 | Ig kappa chain V-IV region (Fragment) OS=Homo sapiens GN=IGKV4-1 PE=4 SV=1 - [KV401_HU | 50974 | 67913 | 195550 | 0,26 | 0,007231 | 0,35 | 0,00484 | 0,75 | 0,658435 |
| P06316 | Ig lambda chain V-I region BL2 OS=Homo sapiens PE=2 SV=1 - [LV107_HUMAN] | 7854 | 1903 | 29114 | 0,27 | 0,277122 | 0,07 | 0,16194 | 4,13 | 0,158017 |
| P01700 | Ig lambda chain V-I region HA OS=Homo sapiens PE=1 SV=1 - [LV102_HUMAN] | 318378 | 328035 | 610263 | 0,52 | 0,472106 | 0,54 | 0,143651 | 0,97 | 0,980727 |
| P80748 | Ig lambda chain V-III region LOI OS=Homo sapiens PE=1 SV=1 - [LV302_HUMAN] | 158713 | 353049 | 1867 | 85,02 | 0,123049 | 189,12 | 0,024653 | 0,45 | 0,070179 |
| P01714 | Ig lambda chain V-III region SH OS=Homo sapiens PE=1 SV=1 - [LV301_HUMAN] | 11272 | 27665 | 15578 | 0,72 | 0,691802 | 1,78 | 0,396353 | 0,41 | 0,03101 |
| P01717 | Ig lambda chain V-IV region Hil OS=Homo sapiens PE=1 SV=1 - [LV403_HUMAN] | 2451495 | 2533647 | 2689512 | 0,91 | 0,701376 | 0,94 | 0,811425 | 0,97 | 0,892675 |
| P0CG05 | Ig lambda-2 chain C regions OS=Homo sapiens GN=IGLC2 PE=1 SV=1 - [LAC2_HUMAN] | 30530 | 215635 | 455035 | 0,07 | 8,94E-06 | 0,47 | 0,129142 | 0,14 | 0,014852 |
| P01871 | Ig mu chain C region OS=Homo sapiens GN=IGHM PE=1 SV=3 - [IGHM_HUMAN] | 331139 | 996538 | 1026957 | 0,32 | 0,09034 | 0,97 | 0,98485 | 0,33 | 0,554664 |
| Q9Y6R7 | IgGFc-binding protein OS=Homo sapiens GN=FCGBP PE=1 SV=3 - [FCGBP_HUMAN] | 12800603 | 14628262 | 20955434 | 0,61 | 0,326441 | 0,70 | 0,240249 | 0,88 | 0,808152 |
| B9A064 | Immunoglobulin lambda-like polypeptide 5 OS=Homo sapiens GN=IGLL5 PE=2 SV=2 - [IGLL5_H | 41121041 | 48334586 | 53710106 | 0,77 | 0,239516 | 0,90 | 0,556703 | 0,85 | 0,429824 |
| O14498 | Immunoglobulin superfamily containing leucine-rich repeat protein OS=Homo sapiens GN=ISLI | 35998 | 10639 | 42871 | 0,84 | 0,802413 | 0,25 | 0,095488 | 3,38 | 0,059727 |
| Q9UPR0 | Inactive phospholipase C-like protein 2 OS=Homo sapiens GN=PLCL2 PE=1 SV=2 - [PLCL2_HUM | 73529 | 148752 | 20660 | 3,56 | 0,11322 | 7,20 | 0,055575 | 0,49 | 0,079978 |
| Q8N3Z0 | Inactive serine protease 35 OS=Homo sapiens GN=PRSS35 PE=2 SV=2 - [PRS35_HUMAN] | 18732 | 1617 | 441 | 42,50 | 0,343132 | 3,67 | 0,507926 | 11,58 | 0,161102 |
| Q14571 | Inositol 1,4,5-trisphosphate receptor type 2 OS=Homo sapiens GN=ITPR2 PE=1 SV=2 - [ITPR2_H | 134674 | 176048 | 550 | 244,94 | 0,000509 | 320,19 | 7,56E-05 | 0,76 | 0,212131 |
| Q8IWB1 | Inositol 1,4,5-trisphosphate receptor-interacting protein OS=Homo sapiens GN=ITPRIP PE=1 SV | 28626 | 49944 | 11961 | 2,39 | 0,581097 | 4,18 | 0,218791 | 0,57 | 0,458619 |
| P01344 | Insulin-like growth factor II OS=Homo sapiens GN=IGF2 PE=1 SV=1 - [IGF2_HUMAN] | 70840 | 90706 | 467774 | 0,15 | 1,99E-07 | 0,19 | 9,11E-07 | 0,78 | 0,515097 |
| P18065 | Insulin-like growth factor-binding protein 2 OS=Homo sapiens GN=IGFBP2 PE=1 SV=2 - [IBP2_H | 563412 | 419007 | 607673 | 0,93 | 0,80494 | 0,69 | 0,143651 | 1,34 | 0,236508 |
| P17936 | Insulin-like growth factor-binding protein 3 OS=Homo sapiens GN=IGFBP3 PE=1 SV=2 - [IBP3_H | 25988 | 24951 | 4233 | 6,14 | 0,225565 | 5,89 | 0,224973 | 1,04 | 0,962787 |
| P22692 | Insulin-like growth factor-binding protein 4 OS=Homo sapiens GN=IGFBP4 PE=1 SV=2 - [IBP4_H | 357504 | 177271 | 71398 | 5,01 | 0,140529 | 2,48 | 0,262857 | 2,02 | 0,186937 |
| P24593 | Insulin-like growth factor-binding protein 5 OS=Homo sapiens GN=IGFBP5 PE=1 SV=1 - [IBP5_H | 121698 | 136694 | 11303 | 10,77 | 0,112776 | 12,09 | 0,154044 | 0,89 | 0,874941 |
| P24592 | Insulin-like growth factor-binding protein 6 OS=Homo sapiens GN=IGFBP6 PE=1 SV=1 - [IBP6_H | 5532426 | 5490173 | 3709593 | 1,49 | 0,22176 | 1,48 | 0,216167 | 1,01 | 0,980727 |
| Q16270 | Insulin-like growth factor-binding protein 7 OS=Homo sapiens GN=IGFBP7 PE=1 SV=1 - [IBP7_H | 7018466 | 7241742 | 2911794 | 2,41 | 0,058672 | 2,49 | 0,065978 | 0,97 | 0,938084 |
| P35858 | Insulin-like growth factor-binding protein complex acid labile subunit OS=Homo sapiens GN=IG | 542217 | 555495 | 889020 | 0,61 | 0,050362 | 0,62 | 0,070228 | 0,98 | 0,944831 |
| Q9Y287 | Integral membrane protein 2B OS=Homo sapiens GN=ITM2B PE=1 SV=1 - [ITM2B_HUMAN] | 1728182 | 1648716 | 22315 | 77,44 | 0,037714 | 73,88 | 0,020452 | 1,05 | 0,936464 |
| P26006 | Integrin alpha-3 OS=Homo sapiens GN=ITGA3 PE=1 SV=5 - [ITA3_HUMAN] | 617077 | 220599 | 18529 | 33,30 | 0,008941 | 11,91 | 0,036293 | 2,80 | 0,005079 |
| P19827 | Inter-alpha-trypsin inhibitor heavy chain H1 OS=Homo sapiens GN=ITIH1 PE=1 SV=3 - [ITIH1_H | 12263795 | 8014836 | 22543183 | 0,54 | 0,101261 | 0,36 | 0,002849 | 1,53 | 0,324346 |
| P19823 | Inter-alpha-trypsin inhibitor heavy chain H2 OS=Homo sapiens GN=ITIH2 PE=1 SV=2 - [ITIH2_H | 61113338 | 59972758 | 49339226 | 1,24 | 0,329494 | 1,22 | 0,286793 | 1,02 | 0,926443 |
| Q06033 | Inter-alpha-trypsin inhibitor heavy chain H3 OS=Homo sapiens GN=ITIH3 PE=1 SV=2 - [ITIH3_H | 54720 | 22706 | 17408 | 3,14 | 0,514871 | 1,30 | 0,766922 | 2,41 | 0,422389 |
| Q14624 | Inter-alpha-trypsin inhibitor heavy chain H4 OS=Homo sapiens GN=ITIH4 PE=1 SV=4 - [ITIH4_H | 9790530 | 7757702 | 16037370 | 0,61 | 0,018316 | 0,48 | 0,000776 | 1,26 | 0,32795 |
| Q86UX2 | Inter-alpha-trypsin inhibitor heavy chain H5 OS=Homo sapiens GN=ITIH5 PE=2 SV=2 - [ITIH5_H | 121068 | 136493 | 6166 | 19,63 | 0,016956 | 22,14 | 0,014938 | 0,89 | 0,767903 |
| Q7Z2Y8 | Interferon-induced very large GTPase 1 OS=Homo sapiens GN=GVINP1 PE=2 SV=2 - [GVIN1_HU | 528083 | 553919 | 70024 | 7,54 | 0,453877 | 7,91 | 0,051753 | 0,95 | 0,963841 |
| P40189 | Interleukin-6 receptor subunit beta OS=Homo sapiens GN=IL6ST PE=1 SV=2 - [IL6RB_HUMAN] | 487004 | 302921 | 424074 | 1,15 | 0,605955 | 0,71 | 0,152028 | 1,61 | 0,016453 |
| Q17R60 | Interphotoreceptor matrix proteoglycan 1 OS=Homo sapiens GN=IMPG1 PE=1 SV=2 - [IMPG1_ | 560464 | 558921 | 975874 | 0,57 | 0,421993 | 0,57 | 0,392046 | 1,00 | 0,997748 |
| Q9BZV3 | Interphotoreceptor matrix proteoglycan 2 OS=Homo sapiens GN=IMPG2 PE=1 SV=3 - [IMPG2_ | 2026871 | 1917837 | 1694916 | 1,20 | 0,689499 | 1,13 | 0,789021 | 1,06 | 0,907395 |
| Q96N16 | Janus kinase and microtubule-interacting protein 1 OS=Homo sapiens GN=JAKMIP1 PE=1 SV=1 | 267418 | 1793281 | 831787 | 0,32 | 0,001229 | 2,16 | 0,325366 | 0,15 | 0,006151 |
| Q9P266 | Junctional protein associated with coronary artery disease OS=Homo sapiens GN=KIAA1462 PE | 499281 | 1084900 | 652741 | 0,76 | 0,536432 | 1,66 | 0,011426 | 0,46 | 7,07E-05 |
| Q86VZ6 | Juxtaposed with another zinc finger protein 1 OS=Homo sapiens GN=JAZF1 PE=1 SV=2 - [JAZF1 | 2716052287 | 1810425378 | 3039985889 | 0,89 | 0,706095 | 0,60 | 0,026914 | 1,50 | 0,135734 |
| Q9BW62 | Katanin p60 ATPase-containing subunit A-like 1 OS=Homo sapiens GN=KATNAL1 PE=1 SV=1 - [K | 1253 | 277364 | 4163 | 0,30 | 0,450949 | 66,62 | 0,463783 | 0,00 | 0,228806 |
| P13645 | Keratin, type I cytoskeletal 10 OS=Homo sapiens GN=KRT10 PE=1 SV=6 - [K1C10_HUMAN] | 91617695 | 127100527 | 32840928 | 2,79 | 0,055626 | 3,87 | 0,025974 | 0,72 | 0,266723 |
| P13646 | Keratin, type I cytoskeletal 13 OS=Homo sapiens GN=KRT13 PE=1 SV=4 - [K1C13_HUMAN] | 198659239 | 268445336 | 330410367 | 0,60 | 0,027722 | 0,81 | 0,376191 | 0,74 | 0,16513 |
| P02533 | Keratin, type I cytoskeletal 14 OS=Homo sapiens GN=KRT14 PE=1 SV=4 - [K1C14_HUMAN] | 4408543 | 11195835 | 646634 | 6,82 | 0,373773 | 17,31 | 0,363706 | 0,39 | 0,404952 |
| P08779 | Keratin, type I cytoskeletal 16 OS=Homo sapiens GN=KRT16 PE=1 SV=4 - [K1C16_HUMAN] | 6101443 | 7318798 | 2314097 | 2,64 | 0,129473 | 3,16 | 0,029011 | 0,83 | 0,613593 |
| Q04695 | Keratin, type I cytoskeletal 17 OS=Homo sapiens GN=KRT17 PE=1 SV=2 - [K1C17_HUMAN] | 169749 | 92539 | 19549 | 8,68 | 0,50085 | 4,73 | 0,33135 | 1,83 | 0,651698 |
| P08727 | Keratin, type I cytoskeletal 19 OS=Homo sapiens GN=KRT19 PE=1 SV=4 - [K1C19_HUMAN] | 520817 | 397898 | 448942 | 1,16 | 0,690415 | 0,89 | 0,75701 | 1,31 | 0,399143 |
| Q6A162 | Keratin, type I cytoskeletal 40 OS=Homo sapiens GN=KRT40 PE=1 SV=2 - [K1C40_HUMAN] | 18684961 | 6171587 | 3934075 | 4,75 | 0,015153 | 1,57 | 0,206997 | 3,03 | 0,000573 |
| P35527 | Keratin, type I cytoskeletal 9 OS=Homo sapiens GN=KRT9 PE=1 SV=3 - [K1C9_HUMAN] | 36622262 | 64972086 | 23649578 | 1,55 | 0,367522 | 2,75 | 0,334987 | 0,56 | 0,338818 |
| Q14533 | Keratin, type II cuticular Hb1 OS=Homo sapiens GN=KRT81 PE=1 SV=3 - [KRT81_HUMAN] | 140213 | 112520 | 8575 | 16,35 | 0,023959 | 13,12 | 0,021204 | 1,25 | 0,590084 |
| P04264 | Keratin, type II cytoskeletal 1 OS=Homo sapiens GN=KRT1 PE=1 SV=6 - [K2C1_HUMAN] | 90799456 | 133608618 | 34887485 | 2,60 | 0,092723 | 3,83 | 0,134772 | 0,68 | 0,385799 |
| Q7Z794 | Keratin, type II cytoskeletal 1b OS=Homo sapiens GN=KRT77 PE=2 SV=3 - [K2C1B_HUMAN] | 14776 | 16266 | 1739 | 8,50 | 0,408184 | 9,35 | 0,300508 | 0,91 | 0,938084 |
| P35908 | Keratin, type II cytoskeletal 2 epidermal OS=Homo sapiens GN=KRT2 PE=1 SV=2 - [K22E_HUMA | 74189472 | 98798214 | 32102736 | 2,31 | 0,037714 | 3,08 | 0,028688 | 0,75 | 0,268183 |
| P12035 | Keratin, type II cytoskeletal 3 OS=Homo sapiens GN=KRT3 PE=1 SV=3 - [K2C3_HUMAN] | 583711 | 919761 | 1464463 | 0,40 | 0,019923 | 0,63 | 0,102066 | 0,63 | 0,077953 |
| P13647 | Keratin, type II cytoskeletal 5 OS=Homo sapiens GN=KRT5 PE=1 SV=3 - [K2C5_HUMAN] | 1458110 | 2239245 | 127284 | 11,46 | 0,120964 | 17,59 | 0,119323 | 0,65 | 0,468311 |
| P02538 | Keratin, type II cytoskeletal 6A OS=Homo sapiens GN=KRT6A PE=1 SV=3 - [K2C6A_HUMAN] | 1319170 | 699575 | 205268 | 6,43 | 0,530083 | 3,41 | 0,313813 | 1,89 | 0,64579 |
| P04259 | Keratin, type II cytoskeletal 6B OS=Homo sapiens GN=KRT6B PE=1 SV=5 - [K2C6B_HUMAN] | 52183 | 22197 | 17367 | 3,00 | 0,752097 | 1,28 | 0,723017 | 2,35 | 0,73561 |
| Q5T749 | Keratinocyte proline-rich protein OS=Homo sapiens GN=KPRP PE=1 SV=1 - [KPRP_HUMAN] | 19155 | 48022 | 2079 | 9,21 | 0,217422 | 23,10 | 0,511408 | 0,40 | 0,554664 |
| P52732 | Kinesin-like protein KIF11 OS=Homo sapiens GN=KIF11 PE=1 SV=2 - [KIF11_HUMAN] | 1313431 | 1028676 | 1093230 | 1,20 | 0,726163 | 0,94 | 0,879622 | 1,28 | 0,513622 |
| Q9NS87 | Kinesin-like protein KIF15 OS=Homo sapiens GN=KIF15 PE=1 SV=1 - [KIF15_HUMAN] | 2037984 | 2311712 | 8020411 | 0,25 | 2,44E-06 | 0,29 | 1,81E-07 | 0,88 | 0,770502 |
| Q9P2E2 | Kinesin-like protein KIF17 OS=Homo sapiens GN=KIF17 PE=2 SV=3 - [KIF17_HUMAN] | 47777 | 179214 | 358072 | 0,13 | 0,000189 | 0,50 | 0,064864 | 0,27 | 0,001859 |
| O60333 | Kinesin-like protein KIF1B OS=Homo sapiens GN=KIF1B PE=1 SV=5 - [KIF1B_HUMAN] | 910783 | 1330284 | 1407077 | 0,65 | 0,187756 | 0,95 | 0,793703 | 0,68 | 0,136999 |
| Q96Q89 | Kinesin-like protein KIF20B OS=Homo sapiens GN=KIF20B PE=1 SV=3 - [KI20B_HUMAN] | 238219 | 326232 | 373292 | 0,64 | 0,197837 | 0,87 | 0,743321 | 0,73 | 0,393409 |
| P01042 | Kininogen-1 OS=Homo sapiens GN=KNG1 PE=1 SV=2 - [KNG1_HUMAN] | 48817639 | 43589414 | 94797310 | 0,51 | 0,004407 | 0,46 | 0,000563 | 1,12 | 0,728258 |
| P24043 | Laminin subunit alpha-2 OS=Homo sapiens GN=LAMA2 PE=1 SV=4 - [LAMA2_HUMAN] | 7019363 | 11187509 | 8954953 | 0,78 | 0,422118 | 1,25 | 0,120786 | 0,63 | 0,006151 |
| Q659C4 | La-related protein 1B OS=Homo sapiens GN=LARP1B PE=1 SV=2 - [LAR1B_HUMAN] | 338443 | 575558 | 1526883 | 0,22 | 0,104504 | 0,38 | 0,206497 | 0,59 | 0,189563 |
| Q14766 | Latent-transforming growth factor beta-binding protein 1 OS=Homo sapiens GN=LTBP1 PE=1 S | 4692765 | 4866728 | 3668369 | 1,28 | 0,209923 | 1,33 | 0,101594 | 0,96 | 0,824748 |
| Q14767 | Latent-transforming growth factor beta-binding protein 2 OS=Homo sapiens GN=LTBP2 PE=1 S | 227209 | 271799 | 24684 | 9,20 | 0,205233 | 11,01 | 0,115048 | 0,84 | 0,799729 |
| Q9NS15 | Latent-transforming growth factor beta-binding protein 3 OS=Homo sapiens GN=LTBP3 PE=1 S | 99524599 | 97168548 | 78221591 | 1,27 | 0,342046 | 1,24 | 0,46207 | 1,02 | 0,938084 |
| Q9UIC8 | Leucine carboxyl methyltransferase 1 OS=Homo sapiens GN=LCMT1 PE=1 SV=2 - [LCMT1_HUM | 272784 | 82052 | 164693 | 1,66 | 0,649664 | 0,50 | 0,137144 | 3,32 | 0,208596 |
| P02750 | Leucine-rich alpha-2-glycoprotein OS=Homo sapiens GN=LRG1 PE=1 SV=2 - [A2GL_HUMAN] | 17945345 | 18315030 | 23206090 | 0,77 | 0,316086 | 0,79 | 0,405329 | 0,98 | 0,952288 |
| Q9Y2L9 | Leucine-rich repeat and calponin homology domain-containing protein 1 OS=Homo sapiens GN | 340411 | 445955 | 668043 | 0,51 | 0,140624 | 0,67 | 0,087463 | 0,76 | 0,55731 |

| | | | | | | | | | |
|---|---|---|---|---|---|---|---|---|---|
| P0C6S8 | Leucine-rich repeat and immunoglobulin-like domain-containing nogo receptor-interacting pro | 1416737 | 1326754 | 724195 | 1,96 | 0,436785 | 1,83 | 0,376634 | 1,07 | 0,926722 |
| Q6UXK5 | Leucine-rich repeat neuronal protein 1 OS=Homo sapiens GN=LRRN1 PE=1 SV=1 - [LRRN1_HUM | 18610 | 1684 | 0 | #DIV/0! | 0,265206 | #DIV/0! | 0,186418 | 11,05 | 0,108457 |
| Q5VZK9 | Leucine-rich repeat-containing protein 16A OS=Homo sapiens GN=LRRC16A PE=1 SV=1 - [LR16 | 354636 | 894206 | 340215 | 1,04 | 0,958683 | 2,63 | 0,020006 | 0,40 | 0,000519 |
| P42702 | Leukemia inhibitory factor receptor OS=Homo sapiens GN=LIFR PE=1 SV=1 - [LIFR_HUMAN] | 70400 | 258480 | 72166 | 0,98 | 0,969011 | 3,58 | 0,072074 | 0,27 | 0,001457 |
| Q9UHB6 | LIM domain and actin-binding protein 1 OS=Homo sapiens GN=LIMA1 PE=1 SV=1 - [LIMA1_HU | 2268924 | 673378 | 228998 | 9,91 | 0,002486 | 2,94 | 0,025259 | 3,37 | 9,92E-05 |
| P50458 | LIM/homeobox protein Lhx2 OS=Homo sapiens GN=LHX2 PE=2 SV=2 - [LHX2_HUMAN] | 5507969 | 7359067 | 4971806 | 1,11 | 0,714654 | 1,48 | 0,042762 | 0,75 | 0,086573 |
| Q13449 | Limbic system-associated membrane protein OS=Homo sapiens GN=LSAMP PE=1 SV=2 - [LSAM | 279411 | 214913 | 69770 | 4,00 | 0,293056 | 3,08 | 0,089799 | 1,30 | 0,699208 |
| Q5T7N2 | LINE-1 type transposase domain-containing protein 1 OS=Homo sapiens GN=L1TD1 PE=1 SV=1 | 39201868 | 60430749 | 180830646 | 0,22 | 2,69E-08 | 0,33 | 0,000939 | 0,65 | 0,324052 |
| P18428 | Lipopolysaccharide-binding protein OS=Homo sapiens GN=LBP PE=1 SV=3 - [LBP_HUMAN] | 20737 | 18702 | 24758 | 0,84 | 0,839118 | 0,76 | 0,75207 | 1,11 | 0,932695 |
| P50851 | Lipopolysaccharide-responsive and beige-like anchor protein OS=Homo sapiens GN=LRBA PE= | 25184 | 8457 | 3161 | 7,97 | 0,155702 | 2,68 | 0,404056 | 2,98 | 0,103726 |
| Q9Y234 | Lipoyltransferase 1, mitochondrial OS=Homo sapiens GN=LIPT1 PE=1 SV=1 - [LIPT_HUMAN] | 5506264 | 2907853 | 3039260 | 1,81 | 0,161969 | 0,96 | 0,842 | 1,89 | 0,013693 |
| Q6ZMR3 | L-lactate dehydrogenase A-like 6A OS=Homo sapiens GN=LDHAL6A PE=2 SV=1 - [LDH6A_HUM | 49996 | 70302 | 21644 | 2,31 | 0,371526 | 3,25 | 0,101663 | 0,71 | 0,429824 |
| Q496Y0 | LON peptidase N-terminal domain and RING finger protein 3 OS=Homo sapiens GN=LONRF3 P | 4122390 | 4289598 | 6933081 | 0,59 | 0,152077 | 0,62 | 0,197073 | 0,96 | 0,922335 |
| P98164 | Low-density lipoprotein receptor-related protein 2 OS=Homo sapiens GN=LRP2 PE=1 SV=3 - [L | 598869 | 651585 | 67478 | 8,88 | 0,064755 | 9,66 | 0,015727 | 0,92 | 0,862436 |
| Q9P2M1 | LRP2-binding protein OS=Homo sapiens GN=LRP2BP PE=1 SV=2 - [LR2BP_HUMAN] | 923272 | 161478 | 102809 | 8,98 | 0,117523 | 1,57 | 0,555956 | 5,72 | 0,014852 |
| P51884 | Lumican OS=Homo sapiens GN=LUM PE=1 SV=2 - [LUM_HUMAN] | 6097972 | 4893933 | 7065805 | 0,86 | 0,514871 | 0,69 | 0,119323 | 1,25 | 0,336017 |
| P38571 | Lysosomal acid lipase/cholesteryl ester hydrolase OS=Homo sapiens GN=LIPA PE=1 SV=2 - [LIC | 11656 | 12445 | 0 | #DIV/0! | 0,213936 | #DIV/0! | 0,137811 | 0,94 | 0,944533 |
| P10253 | Lysosomal alpha-glucosidase OS=Homo sapiens GN=GAA PE=1 SV=4 - [LYAG_HUMAN] | 211999 | 112689 | 85514 | 2,48 | 0,137946 | 1,32 | 0,555511 | 1,88 | 0,086134 |
| P61626 | Lysozyme C OS=Homo sapiens GN=LYZ PE=1 SV=1 - [LYSC_HUMAN] | 378916 | 593118 | 536420 | 0,71 | 0,615875 | 1,11 | 0,843222 | 0,64 | 0,127069 |
| Q86V88 | Magnesium-dependent phosphatase 1 OS=Homo sapiens GN=MDP1 PE=1 SV=1 - [MGDP1_HU | 419643 | 142954 | 263057 | 1,60 | 0,843225 | 0,54 | 0,040042 | 2,94 | 0,631571 |
| P33908 | Mannosyl-oligosaccharide 1,2-alpha-mannosidase IA OS=Homo sapiens GN=MAN1A1 PE=1 SV | 36968 | 22421 | 2480 | 14,90 | 0,124453 | 9,04 | 0,050322 | 1,65 | 0,391013 |
| Q9NS73 | MAP3K12-binding inhibitory protein 1 OS=Homo sapiens GN=MBIP PE=1 SV=2 - [MBIP1_HUM | 202428 | 92931 | 60674 | 3,34 | 0,422344 | 1,53 | 0,620257 | 2,18 | 0,366354 |
| P08493 | Matrix Gla protein OS=Homo sapiens GN=MGP PE=1 SV=2 - [MGP_HUMAN] | 42000 | 14093 | 9140 | 4,60 | 0,50144 | 1,54 | 0,685019 | 2,98 | 0,405173 |
| Q14676 | Mediator of DNA damage checkpoint protein 1 OS=Homo sapiens GN=MDC1 PE=1 SV=3 - [MD | 2292980 | 2771955 | 775825 | 2,96 | 0,223913 | 3,57 | 0,066868 | 0,83 | 0,699208 |
| Q9Y4F3 | Meiosis arrest female protein 1 OS=Homo sapiens GN=KIAA0430 PE=1 SV=6 - [MARF1_HUMAN | 2408 | 12260 | 147521 | 0,02 | 0,000652 | 0,08 | 0,007867 | 0,20 | 0,458619 |
| Q8NEH6 | Meiosis-specific nuclear structural protein 1 OS=Homo sapiens GN=MNS1 PE=2 SV=2 - [MNS1_ | 299872 | 187017 | 328250 | 0,91 | 0,901118 | 0,57 | 0,474926 | 1,60 | 0,338818 |
| Q9Y5K1 | Meiotic recombination protein SPO11 OS=Homo sapiens GN=SPO11 PE=2 SV=1 - [SPO11_HUM | 221746 | 391327 | 270291 | 0,82 | 0,647339 | 1,45 | 0,439296 | 0,57 | 0,091396 |
| Q8N344 | Mesoderm induction early response protein 2 OS=Homo sapiens GN=MIER2 PE=1 SV=2 - [MIEF | 157563 | 182424 | 70599 | 2,23 | 0,488548 | 2,58 | 0,233434 | 0,86 | 0,834401 |
| P01033 | Metalloproteinase inhibitor 1 OS=Homo sapiens GN=TIMP1 PE=1 SV=1 - [TIMP1_HUMAN] | 984406 | 642435 | 1868741 | 0,53 | 0,019923 | 0,34 | 0,001353 | 1,53 | 0,006735 |
| P16035 | Metalloproteinase inhibitor 2 OS=Homo sapiens GN=TIMP2 PE=1 SV=2 - [TIMP2_HUMAN] | 1625133 | 1669003 | 815130 | 1,99 | 0,134339 | 2,05 | 0,067066 | 0,97 | 0,944533 |
| Q8NFT2 | Metalloreductase STEAP2 OS=Homo sapiens GN=STEAP2 PE=1 SV=3 - [STEA2_HUMAN] | 6147549 | 4526755 | 5966016 | 1,03 | 0,923716 | 0,76 | 0,210253 | 1,36 | 0,172161 |
| Q9P267 | Methyl-CpG-binding domain protein 5 OS=Homo sapiens GN=MBD5 PE=1 SV=3 - [MBD5_HUM | 5338478 | 7070970 | 4074987 | 1,31 | 0,332661 | 1,74 | 0,01047 | 0,75 | 0,079978 |
| P55083 | Microfibril-associated glycoprotein 4 OS=Homo sapiens GN=MFAP4 PE=1 SV=2 - [MFAP4_HUM | 5803781 | 7594410 | 5099423 | 1,14 | 0,647339 | 1,49 | 0,11291 | 0,76 | 0,123459 |
| P55001 | Microfibrillar-associated protein 2 OS=Homo sapiens GN=MFAP2 PE=2 SV=1 - [MFAP2_HUMAN | 51380 | 47586 | 31369 | 1,64 | 0,434228 | 1,52 | 0,416358 | 1,08 | 0,872698 |
| Q9UPN3 | Microtubule-actin cross-linking factor 1, isoforms 1/2/3/5 OS=Homo sapiens GN=MACF1 PE=1 | 29129 | 90832 | 34273 | 0,85 | 0,835108 | 2,65 | 0,206997 | 0,32 | 0,015453 |
| Q9NU22 | Midasin OS=Homo sapiens GN=MDN1 PE=1 SV=2 - [MDN1_HUMAN] | 413737443 | 474647125 | 525766693 | 0,79 | 0,543991 | 0,90 | 0,80068 | 0,87 | 0,767419 |
| Q6P0N0 | Mis18-binding protein 1 OS=Homo sapiens GN=MIS18BP1 PE=1 SV=1 - [M18BP_HUMAN] | 827675 | 358680 | 381706 | 2,17 | 0,204477 | 0,94 | 0,918089 | 2,31 | 0,059727 |
| Q9H1K4 | Mitochondrial glutamate carrier 2 OS=Homo sapiens GN=SLC25A18 PE=1 SV=1 - [GHC2_HUMA | 19722057 | 26976893 | 5780657 | 3,41 | 0,043933 | 4,67 | 0,003369 | 0,73 | 0,225987 |
| Q969M1 | Mitochondrial import receptor subunit TOM40B OS=Homo sapiens GN=TOMM40L PE=1 SV=1 - | 311250 | 953897 | 595309 | 0,52 | 0,302711 | 1,60 | 0,112223 | 0,33 | 0,000785 |
| Q7L0Y3 | Mitochondrial ribonuclease P protein 1 OS=Homo sapiens GN=TRMT10C PE=1 SV=2 - [MRRP1_ | 357784 | 309701 | 49483 | 7,23 | 0,246555 | 6,26 | 0,002394 | 1,16 | 0,834401 |
| Q9Y2U5 | Mitogen-activated protein kinase kinase kinase 2 OS=Homo sapiens GN=MAP3K2 PE=1 SV=2 - | 169650379 | 198088037 | 322670625 | 0,53 | 5,79E-05 | 0,61 | 0,00484 | 0,86 | 0,404952 |
| P08571 | Monocyte differentiation antigen CD14 OS=Homo sapiens GN=CD14 PE=1 SV=2 - [CD14_HUM | 3593339 | 3381374 | 3364303 | 1,07 | 0,72754 | 1,01 | 0,981518 | 1,06 | 0,770408 |
| Q86VD1 | MORC family CW-type zinc finger protein 1 OS=Homo sapiens GN=MORC1 PE=2 SV=2 - [MORC | 1329998 | 1002108 | 1220231 | 1,09 | 0,787273 | 0,82 | 0,518961 | 1,33 | 0,306703 |
| Q9Y6X9 | MORC family CW-type zinc finger protein 2 OS=Homo sapiens GN=MORC2 PE=1 SV=2 - [MORC | 271648 | 99707 | 266509 | 1,02 | 0,989969 | 0,37 | 0,170789 | 2,72 | 0,513622 |
| Q9UBU8 | Mortality factor 4-like protein 1 OS=Homo sapiens GN=MORF4L1 PE=1 SV=2 - [MO4L1_HUMAN | 240043 | 270924 | 158233 | 1,52 | 0,123049 | 1,71 | 0,091725 | 0,89 | 0,524628 |
| P84022 | Mothers against decapentaplegic homolog 3 OS=Homo sapiens GN=SMAD3 PE=1 SV=1 - [SMA | 2497262 | 3201030 | 4239537 | 0,59 | 0,178571 | 0,76 | 0,440867 | 0,78 | 0,582792 |
| Q02817 | Mucin-2 OS=Homo sapiens GN=MUC2 PE=1 SV=2 - [MUC2_HUMAN] | 53439520 | 12623677 | 596829 | 89,54 | 0,037714 | 21,15 | 0,00787 | 4,23 | 0,006151 |
| Q9H8L6 | Multimerin-2 OS=Homo sapiens GN=MMRN2 PE=1 SV=2 - [MMRN2_HUMAN] | 363319 | 1384464 | 436302 | 0,83 | 0,80494 | 3,17 | 0,004621 | 0,26 | 5,86E-05 |
| Q96KG7 | Multiple epidermal growth factor-like domains protein 10 OS=Homo sapiens GN=MEGF10 PE= | 14092 | 26706 | 4792 | 2,94 | 0,532257 | 5,57 | 0,093862 | 0,53 | 0,32795 |
| Q99972 | Myocilin OS=Homo sapiens GN=MYOC PE=1 SV=2 - [MYOC_HUMAN] | 2797766 | 2460711 | 1929383 | 1,45 | 0,472106 | 1,28 | 0,558641 | 1,14 | 0,73832 |
| Q15746 | Myosin light chain kinase, smooth muscle OS=Homo sapiens GN=MYLK PE=1 SV=4 - [MYLK_HU | 447407 | 191986 | 1088989 | 0,41 | 0,005553 | 0,18 | 6,23E-06 | 2,33 | 0,014199 |
| Q9UKX3 | Myosin-13 OS=Homo sapiens GN=MYH13 PE=2 SV=2 - [MYH13_HUMAN] | 223650 | 163691 | 10024 | 22,31 | 0,058672 | 16,33 | 0,056981 | 1,37 | 0,536813 |
| Q7Z406 | Myosin-14 OS=Homo sapiens GN=MYH14 PE=1 SV=2 - [MYH14_HUMAN] | 12587102 | 19377903 | 17294142 | 0,73 | 0,358807 | 1,12 | 0,776168 | 0,65 | 0,200125 |
| P35579 | Myosin-9 OS=Homo sapiens GN=MYH9 PE=1 SV=4 - [MYH9_HUMAN] | 189554 | 102575 | 29 | 6493,85 | 0,155642 | 3514,07 | 0,107616 | 1,85 | 0,39244 |
| P15586 | N-acetylglucosamine-6-sulfatase OS=Homo sapiens GN=GNS PE=1 SV=3 - [GNS_HUMAN] | 9068413 | 7319244 | 10724403 | 0,85 | 0,541052 | 0,68 | 0,097467 | 1,24 | 0,429824 |
| Q96PD5 | N-acetylmuramoyl-L-alanine amidase OS=Homo sapiens GN=PGLYRP2 PE=1 SV=1 - [PGRP2_HU | 8877740 | 8729048 | 11130709 | 0,80 | 0,28917 | 0,78 | 0,17641 | 1,02 | 0,951038 |
| Q9C000 | NACHT, LRR and PYD domains-containing protein 1 OS=Homo sapiens GN=NLRP1 PE=1 SV=1 - | 385768 | 367343 | 480639 | 0,80 | 0,63274 | 0,76 | 0,539389 | 1,05 | 0,862436 |
| P23368 | NAD-dependent malic enzyme, mitochondrial OS=Homo sapiens GN=ME2 PE=1 SV=1 - [MAOM | 242915 | 474201 | 212093 | 1,15 | 0,838662 | 2,24 | 0,143204 | 0,51 | 0,078298 |
| Q8IXJ6 | NAD-dependent protein deacetylase sirtuin-2 OS=Homo sapiens GN=SIRT2 PE=1 SV=2 - [SIR2_ | 743588 | 947158 | 338425 | 2,20 | 0,098699 | 2,80 | 0,046734 | 0,79 | 0,42491 |
| Q9UHB4 | NADPH-dependent diflavin oxidoreductase 1 OS=Homo sapiens GN=NDOR1 PE=1 SV=1 - [NDO | 33402723 | 11484948 | 13183708 | 2,53 | 0,026966 | 0,87 | 0,496927 | 2,91 | 6,57E-05 |
| Q5VZE5 | N-alpha-acetyltransferase 35, NatC auxiliary subunit OS=Homo sapiens GN=NAA35 PE=1 SV=1 | 3133523 | 3261113 | 9695524 | 0,32 | 0,000504 | 0,34 | 0,00531 | 0,96 | 0,953087 |
| P20929 | Nebulin OS=Homo sapiens GN=NEB PE=1 SV=5 - [NEBU_HUMAN] | 35937405 | 12357649 | 2104757 | 17,07 | 0,00983 | 5,87 | 0,003477 | 2,91 | 0,002407 |
| O75113 | NEDD4-binding protein 1 OS=Homo sapiens GN=N4BP1 PE=1 SV=4 - [N4BP1_HUMAN] | 7826556 | 11067299 | 3402386 | 2,30 | 0,168653 | 3,25 | 0,001637 | 0,71 | 0,173932 |
| Q8NF91 | Nesprin-1 OS=Homo sapiens GN=SYNE1 PE=1 SV=4 - [SYNE1_HUMAN] | 1115624 | 1298754 | 1427333 | 0,78 | 0,28917 | 0,91 | 0,72299 | 0,86 | 0,518261 |
| P48681 | Nestin OS=Homo sapiens GN=NES PE=1 SV=2 - [NEST_HUMAN] | 4984952 | 13553178 | 15878663 | 0,31 | 0,000148 | 0,85 | 0,331142 | 0,37 | 1,05E-06 |
| P13591 | Neural cell adhesion molecule 1 OS=Homo sapiens GN=NCAM1 PE=1 SV=3 - [NCAM1_HUMAN | 2139630 | 1146834 | 637795 | 3,35 | 0,193902 | 1,80 | 0,095008 | 1,87 | 0,18901 |
| O00533 | Neural cell adhesion molecule L1-like protein OS=Homo sapiens GN=CHL1 PE=1 SV=4 - [NCHL1 | 690221 | 520523 | 79695 | 8,66 | 0,079371 | 6,53 | 0,040877 | 1,33 | 0,554664 |
| Q9ULB1 | Neurexin-1 OS=Homo sapiens GN=NRXN1 PE=2 SV=1 - [NRX1A_HUMAN] | 62510 | 55919 | 6112 | 10,23 | 0,097291 | 9,15 | 0,018081 | 1,12 | 0,834506 |
| Q9P2S2 | Neurexin-2 OS=Homo sapiens GN=NRXN2 PE=2 SV=1 - [NRX2A_HUMAN] | 128082 | 134087 | 10470 | 12,23 | 0,216091 | 12,81 | 0,072074 | 0,96 | 0,953579 |
| Q9Y4C0 | Neurexin-3 OS=Homo sapiens GN=NRXN3 PE=1 SV=4 - [NRX3A_HUMAN] | 763428 | 634279 | 324018 | 2,36 | 0,204089 | 1,96 | 0,174492 | 1,20 | 0,669595 |
| Q3BBV0 | Neuroblastoma breakpoint family member 1 OS=Homo sapiens GN=NBPF1 PE=2 SV=1 - [NBPF | 1519616 | 737821 | 924514 | 1,64 | 0,408184 | 0,80 | 0,699241 | 2,06 | 0,134695 |
| P41271 | Neuroblastoma suppressor of tumorigenicity 1 OS=Homo sapiens GN=NBL1 PE=1 SV=2 - [NBL1 | 170603 | 234399 | 5694 | 29,96 | 0,03894 | 41,17 | 0,004262 | 0,73 | 0,384056 |
| O14594 | Neurocan core protein OS=Homo sapiens GN=NCAN PE=1 SV=3 - [NCAN_HUMAN] | 1342405 | 1926666 | 155046 | 8,66 | 0,141522 | 12,43 | 0,003499 | 0,70 | 0,385799 |
| P16519 | Neuroendocrine convertase 2 OS=Homo sapiens GN=PCSK2 PE=2 SV=2 - [NEC2_HUMAN] | 617401 | 503946 | 131950 | 4,68 | 0,03356 | 3,82 | 0,014662 | 1,23 | 0,538047 |
| P05408 | Neuroendocrine protein 7B2 OS=Homo sapiens GN=SCG5 PE=1 SV=2 - [7B2_HUMAN] | 972110 | 1069617 | 57758 | 16,83 | 0,094259 | 18,52 | 0,025974 | 0,91 | 0,86656 |
| O94856 | Neurofascin OS=Homo sapiens GN=NFASC PE=1 SV=4 - [NFASC_HUMAN] | 51337 | 41067 | 9101 | 5,64 | 0,142219 | 4,51 | 0,216996 | 1,25 | 0,728258 |
| Q8NFZ4 | Neuroligin-2 OS=Homo sapiens GN=NLGN2 PE=1 SV=1 - [NLGN2_HUMAN] | 52377 | 10150 | 18289 | 2,86 | 0,376365 | 0,55 | 0,48322 | 5,16 | 0,073279 |
| Q8IVL0 | Neuron navigator 3 OS=Homo sapiens GN=NAV3 PE=1 SV=3 - [NAV3_HUMAN] | 19913592 | 21012178 | 27216569 | 0,73 | 0,221927 | 0,77 | 0,37222 | 0,95 | 0,888849 |
| Q92823 | Neuronal cell adhesion molecule OS=Homo sapiens GN=NRCAM PE=1 SV=3 - [NRCAM_HUMAN | 3324184 | 2524496 | 653637 | 5,09 | 0,021629 | 3,86 | 0,0274 | 1,32 | 0,396413 |
| Q15818 | Neuronal pentraxin-1 OS=Homo sapiens GN=NPTX1 PE=2 SV=2 - [NPTX1_HUMAN] | 459172 | 858915 | 48040 | 9,56 | 0,038741 | 17,88 | 0,029155 | 0,53 | 0,101033 |
| O15240 | Neurosecretory protein VGF OS=Homo sapiens GN=VGF PE=1 SV=2 - [VGF_HUMAN] | 1434844 | 1291751 | 135982 | 10,55 | 0,00983 | 9,50 | 0,007222 | 1,11 | 0,767903 |
| Q99574 | Neuroserpin OS=Homo sapiens GN=SERPINI1 PE=1 SV=1 - [NEUS_HUMAN] | 544657 | 517989 | 88590 | 6,15 | 0,09993 | 5,85 | 0,002069 | 1,05 | 0,926443 |
| Q9P121 | Neurotrimin OS=Homo sapiens GN=NTM PE=1 SV=1 - [NTRI_HUMAN] | 391764 | 314068 | 148381 | 2,64 | 0,028644 | 2,12 | 0,029011 | 1,25 | 0,368331 |
| P26717 | NKG2-C type II integral membrane protein OS=Homo sapiens GN=KLRC2 PE=1 SV=2 - [NKG2C_ | 3003308 | 1615699 | 2965419 | 1,01 | 0,986179 | 0,54 | 0,095105 | 1,86 | 0,223973 |
| Q99784 | Noelin OS=Homo sapiens GN=OLFM1 PE=1 SV=4 - [NOE1_HUMAN] | 39936 | 46472 | 39110 | 1,02 | 0,986072 | 1,19 | 0,74469 | 0,86 | 0,834401 |
| O95897 | Noelin-2 OS=Homo sapiens GN=OLFM2 PE=1 SV=2 - [NOE2_HUMAN] | 24837 | 20675 | 17324 | 1,43 | 0,649664 | 1,19 | 0,791177 | 1,20 | 0,785309 |
| Q6P4R8 | Nuclear factor related to kappa-B-binding protein OS=Homo sapiens GN=NFRKB PE=1 SV=2 - [N | 1380570 | 1093538 | 700581 | 1,97 | 0,149461 | 1,56 | 0,262893 | 1,26 | 0,362102 |
| Q14980 | Nuclear mitotic apparatus protein 1 OS=Homo sapiens GN=NUMA1 PE=1 SV=2 - [NUMA1_HUN | 7363564 | 12240482 | 3382516 | 2,18 | 0,348533 | 3,62 | 0,05582 | 0,60 | 0,208754 |
| Q92621 | Nuclear pore complex protein Nup205 OS=Homo sapiens GN=NUP205 PE=1 SV=3 - [NU205_HU | 1388028 | 2860975 | 1465437 | 0,95 | 0,866798 | 1,95 | 0,018284 | 0,49 | 7,68E-05 |
| Q5VU65 | Nuclear pore membrane glycoprotein 210-like OS=Homo sapiens GN=NUP210L PE=2 SV=1 - [P | 75522 | 31708 | 75791 | 1,00 | 0,995289 | 0,42 | 0,272798 | 2,38 | 0,137949 |
| Q14995 | Nuclear receptor subfamily 1 group D member 2 OS=Homo sapiens GN=NR1D2 PE=1 SV=3 - [N | 14192048 | 14080864 | 637060 | 22,28 | 0,003541 | 22,10 | 0,002319 | 1,01 | 0,98316 |
| Q9NSY0 | Nuclear receptor-binding protein 2 OS=Homo sapiens GN=NRBP2 PE=2 SV=2 - [NRBP2_HUMAN | 1159031 | 939506 | 513619 | 2,26 | 0,008577 | 1,83 | 0,010186 | 1,23 | 0,220553 |
| P48552 | Nuclear receptor-interacting protein 1 OS=Homo sapiens GN=NRIP1 PE=1 SV=2 - [NRIP1_HUM | 64019 | 97081 | 3317 | 19,30 | 0,036905 | 29,27 | 0,005695 | 0,66 | 0,21493 |
| Q02818 | Nucleobindin-1 OS=Homo sapiens GN=NUCB1 PE=1 SV=4 - [NUCB1_HUMAN] | 757845 | 545885 | 453469 | 1,67 | 0,153114 | 1,20 | 0,548804 | 1,39 | 0,137949 |
| O60287 | Nucleolar pre-ribosomal-associated protein 1 OS=Homo sapiens GN=URB1 PE=1 SV=4 - [NPA1 | 51385 | 120378 | 3629 | 14,16 | 0,197075 | 33,17 | 0,026566 | 0,43 | 0,061493 |
| P12270 | Nucleoprotein TPR OS=Homo sapiens GN=TPR PE=1 SV=3 - [TPR_HUMAN] | 15296160 | 23059297 | 25001341 | 0,61 | 0,323391 | 0,92 | 0,850234 | 0,66 | 0,338818 |
| A8MXV4 | Nucleoside diphosphate-linked moiety X motif 19 OS=Homo sapiens GN=NUDT19 PE=1 SV=1 - | 609452 | 786998 | 1378221 | 0,44 | 0,205821 | 0,57 | 0,39135 | 0,77 | 0,751549 |
| Q5VST9 | Obscurin OS=Homo sapiens GN=OBSCN PE=1 SV=3 - [OBSCN_HUMAN] | 1545015 | 889729 | 819546 | 1,89 | 0,092208 | 1,09 | 0,842 | 1,74 | 0,02519 |
| Q8NGQ3 | Olfactory receptor 1S2 OS=Homo sapiens GN=OR1S2 PE=3 SV=2 - [OR1S2_HUMAN] | 69548 | 486 | 9005 | 7,72 | 0,621304 | 0,05 | 0,022315 | 142,99 | 0,404952 |
| P23515 | Oligodendrocyte-myelin glycoprotein OS=Homo sapiens GN=OMG PE=1 SV=2 - [OMGP_HUMA | 539154 | 490382 | 120368 | 4,48 | 0,514871 | 4,07 | 0,203572 | 1,10 | 0,94009 |

| | | | | | | | | | | |
|---|---|---|---|---|---|---|---|---|---|---|
| Q9UBM4 | Opticin OS=Homo sapiens GN=OPTC PE=1 SV=1 - [OPT_HUMAN] | 29384737 | 30038322 | 27513563 | 1,07 | 0,769486 | 1,09 | 0,730168 | 0,98 | 0,936932 |
| O75665 | Oral-facial-digital syndrome 1 protein OS=Homo sapiens GN=OFD1 PE=1 SV=1 - [OFD1_HUMAI | 2575207 | 1451881 | 3485038 | 0,74 | 0,518508 | 0,42 | 0,101663 | 1,77 | 0,32795 |
| O43913 | Origin recognition complex subunit 5 OS=Homo sapiens GN=ORC5 PE=1 SV=1 - [ORC5_HUMAN | 247199 | 499071 | 484558 | 0,51 | 0,025325 | 1,03 | 0,889766 | 0,50 | 0,000391 |
| P10451 | Osteopontin OS=Homo sapiens GN=SPP1 PE=1 SV=1 - [OSTP_HUMAN] | 82893180 | 74526397 | 31751509 | 2,61 | 0,096721 | 2,35 | 0,024792 | 1,11 | 0,760808 |
| Q7RTW8 | Otoancorin OS=Homo sapiens GN=OTOA PE=1 SV=1 - [OTOAN_HUMAN] | 20364 | 7068 | 134465 | 0,15 | 0,005543 | 0,05 | 0,000175 | 2,88 | 0,538047 |
| Q9HC10 | Otoferlin OS=Homo sapiens GN=OTOF PE=1 SV=3 - [OTOF_HUMAN] | 128477849 | 187078386 | 247041238 | 0,52 | 0,019923 | 0,76 | 0,242784 | 0,69 | 0,186937 |
| Q86UD1 | Out at first protein homolog OS=Homo sapiens GN=OAF PE=2 SV=1 - [OAF_HUMAN] | 486671 | 339017 | 229745 | 2,12 | 0,148051 | 1,48 | 0,140006 | 1,44 | 0,232853 |
| Q7RTZ1 | Ovochymase-2 OS=Homo sapiens GN=OVCH2 PE=3 SV=2 - [OVCH2_HUMAN] | 2253252 | 1485789 | 3199565 | 0,70 | 0,104021 | 0,46 | 0,000147 | 1,52 | 0,057326 |
| Q9ULE6 | Paladin OS=Homo sapiens GN=PALD1 PE=1 SV=3 - [PALD_HUMAN] | 14173269 | 19412820 | 28566870 | 0,50 | 0,023685 | 0,68 | 0,27536 | 0,73 | 0,272879 |
| P50897 | Palmitoyl-protein thioesterase 1 OS=Homo sapiens GN=PPT1 PE=1 SV=1 - [PPT1_HUMAN] | 125330 | 151022 | 62301 | 2,01 | 0,336337 | 2,42 | 0,231672 | 0,83 | 0,728258 |
| O95428 | Papilin OS=Homo sapiens GN=PAPLN PE=2 SV=4 - [PPN_HUMAN] | 450620 | 30670 | 335318 | 1,34 | 0,913356 | 0,09 | 0,052462 | 14,69 | 0,555664 |
| Q13219 | Pappalysin-1 OS=Homo sapiens GN=PAPPA PE=1 SV=3 - [PAPP1_HUMAN] | 182998 | 111989 | 590291 | 0,31 | 0,00022 | 0,19 | 2E-05 | 1,63 | 0,079232 |
| Q9BXP8 | Pappalysin-2 OS=Homo sapiens GN=PAPPA2 PE=1 SV=4 - [PAPP2_HUMAN] | 6365204 | 7487395 | 5740697 | 1,11 | 0,755884 | 1,30 | 0,369603 | 0,85 | 0,555664 |
| Q8NB37 | Parkinson disease 7 domain-containing protein 1 OS=Homo sapiens GN=PDDC1 PE=1 SV=1 - [P | 2150337 | 2052017 | 1228060 | 1,75 | 0,535422 | 1,67 | 0,498231 | 1,05 | 0,951038 |
| Q9GZU2 | Paternally-expressed gene 3 protein OS=Homo sapiens GN=PEG3 PE=1 SV=1 - [PEG3_HUMAN] | 128542 | 89830 | 29835 | 4,31 | 0,10028 | 3,01 | 0,138188 | 1,43 | 0,42591 |
| Q6UXB8 | Peptidase inhibitor 16 OS=Homo sapiens GN=PI16 PE=1 SV=1 - [PI16_HUMAN] | 12112 | 14155 | 3040 | 3,98 | 0,529267 | 4,66 | 0,426324 | 0,86 | 0,905747 |
| Q96LB9 | Peptidoglycan recognition protein 3 OS=Homo sapiens GN=PGLYRP3 PE=1 SV=1 - [PGRP3_HUN | 100857914 | 92228812 | 75096059 | 1,34 | 0,131963 | 1,23 | 0,048994 | 1,09 | 0,482305 |
| P19021 | Peptidyl-glycine alpha-amidating monooxygenase OS=Homo sapiens GN=PAM PE=1 SV=2 - [AN | 204498 | 93636 | 65779 | 3,11 | 0,092616 | 1,42 | 0,33114 | 2,18 | 0,022033 |
| P68106 | Peptidyl-prolyl cis-trans isomerase FKBP1B OS=Homo sapiens GN=FKBP1B PE=1 SV=2 - [FKB1B | 121206764 | 99889069 | 93510817 | 1,30 | 0,23996 | 1,07 | 0,757562 | 1,21 | 0,276928 |
| Q9BXM0 | Periaxin OS=Homo sapiens GN=PRX PE=1 SV=2 - [PRAX_HUMAN] | 2713817 | 3196174 | 2578828 | 1,05 | 0,941804 | 1,24 | 0,489353 | 0,85 | 0,705613 |
| P41219 | Peripherin OS=Homo sapiens GN=PRPH PE=1 SV=2 - [PERI_HUMAN] | 42814 | 36882 | 106843 | 0,40 | 0,157 | 0,35 | 0,100254 | 1,16 | 0,899768 |
| Q8NDX1 | PH and SEC7 domain-containing protein 4 OS=Homo sapiens GN=PSD4 PE=1 SV=2 - [PSD4_HUI | 3776877 | 3978889 | 1996215 | 1,89 | 0,324536 | 1,99 | 0,176696 | 0,95 | 0,907698 |
| O94880 | PHD finger protein 14 OS=Homo sapiens GN=PHF14 PE=1 SV=2 - [PHF14_HUMAN] | 10644581 | 14864652 | 1145820 | 9,29 | 0,018316 | 12,97 | 0,021204 | 0,72 | 0,350184 |
| O75167 | Phosphatase and actin regulator 2 OS=Homo sapiens GN=PHACTR2 PE=1 SV=2 - [PHAR2_HUM | 737440 | 393538 | 1157319 | 0,64 | 0,39193 | 0,34 | 0,123005 | 1,87 | 0,135638 |
| P30086 | Phosphatidylethanolamine-binding protein 1 OS=Homo sapiens GN=PEBP1 PE=1 SV=3 - [PEBP1 | 75161 | 23600 | 93761 | 0,80 | 0,698175 | 0,25 | 0,062812 | 3,18 | 0,015453 |
| Q96S96 | Phosphatidylethanolamine-binding protein 4 OS=Homo sapiens GN=PEBP4 PE=1 SV=3 - [PEBP4 | 2458910 | 3001503 | 813290 | 3,02 | 0,081013 | 3,69 | 0,018225 | 0,82 | 0,545286 |
| Q92569 | Phosphatidylinositol 3-kinase regulatory subunit gamma OS=Homo sapiens GN=PIK3R3 PE=1 S | 125811 | 184529 | 148948 | 0,84 | 0,894535 | 1,24 | 0,784465 | 0,68 | 0,685914 |
| P42356 | Phosphatidylinositol 4-kinase alpha OS=Homo sapiens GN=PI4KA PE=1 SV=4 - [PI4KA_HUMAN | 672176 | 2723851 | 150921 | 4,45 | 0,28703 | 18,05 | 4,64E-06 | 0,25 | 1,05E-06 |
| O00443 | Phosphatidylinositol 4-phosphate 3-kinase C2 domain-containing subunit alpha OS=Homo sapi | 5851954 | 9626104 | 3644453 | 1,61 | 0,200241 | 2,64 | 0,000184 | 0,61 | 0,002493 |
| O75747 | Phosphatidylinositol 4-phosphate 3-kinase C2 domain-containing subunit gamma OS=Homo sa | 2518 | 2607 | 32391 | 0,08 | 0,102278 | 0,08 | 0,113937 | 0,97 | 0,980888 |
| Q00169 | Phosphatidylinositol transfer protein alpha isoform OS=Homo sapiens GN=PITPNA PE=1 SV=2 - | 687806 | 450340 | 91926 | 7,48 | 0,521878 | 4,90 | 0,158586 | 1,53 | 0,751144 |
| Q96FE7 | Phosphoinositide-3-kinase-interacting protein 1 OS=Homo sapiens GN=PIK3IP1 PE=1 SV=2 - [P | 508107 | 609729 | 553297 | 0,92 | 0,865407 | 1,10 | 0,713417 | 0,83 | 0,582136 |
| P55058 | Phospholipid transfer protein OS=Homo sapiens GN=PLTP PE=1 SV=1 - [PLTP_HUMAN] | 1421340 | 1137519 | 1249288 | 1,14 | 0,709168 | 0,91 | 0,790589 | 1,25 | 0,289237 |
| P36955 | Pigment epithelium-derived factor OS=Homo sapiens GN=SERPINF1 PE=1 SV=4 - [PEDF_HUMA | 810767185 | 784648119 | 531102258 | 1,53 | 0,06086 | 1,48 | 0,015693 | 1,03 | 0,843097 |
| Q8WWB5 | PIH1 domain-containing protein 2 OS=Homo sapiens GN=PIH1D2 PE=1 SV=1 - [PIHD2_HUMAN | 4427194 | 5441599 | 2538949 | 1,74 | 0,298847 | 2,14 | 0,11553 | 0,81 | 0,582136 |
| Q63HQ2 | Pikachurin OS=Homo sapiens GN=EGFLAM PE=1 SV=2 - [EGFLA_HUMAN] | 43873 | 47673 | 1522 | 28,83 | 0,104504 | 31,32 | 0,004262 | 0,92 | 0,884745 |
| Q8TC59 | Piwi-like protein 2 OS=Homo sapiens GN=PIWIL2 PE=1 SV=1 - [PIWL2_HUMAN] | 284433 | 189211 | 134983 | 2,11 | 0,125572 | 1,40 | 0,31341 | 1,50 | 0,134695 |
| P03952 | Plasma kallikrein OS=Homo sapiens GN=KLKB1 PE=1 SV=1 - [KLKB1_HUMAN] | 84732 | 73038 | 325372 | 0,26 | 0,000374 | 0,22 | 0,000903 | 1,16 | 0,808152 |
| Q01814 | Plasma membrane calcium-transporting ATPase 2 OS=Homo sapiens GN=ATP2B2 PE=1 SV=2 - | 13366 | 21074 | 7326 | 1,82 | 0,542161 | 2,88 | 0,270269 | 0,63 | 0,457069 |
| P05155 | Plasma protease C1 inhibitor OS=Homo sapiens GN=SERPING1 PE=1 SV=2 - [IC1_HUMAN] | 47334845 | 47819294 | 55001188 | 0,86 | 0,142699 | 0,87 | 0,138069 | 0,99 | 0,936932 |
| P05154 | Plasma serine protease inhibitor OS=Homo sapiens GN=SERPINA5 PE=1 SV=3 - [IPSP_HUMAN] | 335151 | 667023 | 181852 | 1,84 | 0,541052 | 3,67 | 0,010091 | 0,50 | 0,059532 |
| P00747 | Plasminogen OS=Homo sapiens GN=PLG PE=1 SV=2 - [PLMN_HUMAN] | 53683767 | 64740691 | 113958036 | 0,47 | 4,65E-05 | 0,57 | 0,000481 | 0,83 | 0,32795 |
| Q15195 | Plasminogen-like protein A OS=Homo sapiens GN=PLGLA PE=2 SV=1 - [PLGA_HUMAN] | 347858 | 328106 | 332926 | 1,04 | 0,912071 | 0,99 | 0,953574 | 1,06 | 0,870099 |
| P16284 | Platelet endothelial cell adhesion molecule OS=Homo sapiens GN=PECAM1 PE=1 SV=1 - [PECA | 139644 | 64491 | 2960 | 47,18 | 0,150051 | 21,79 | 0,173816 | 2,17 | 0,278282 |
| Q9HB21 | Pleckstrin homology domain-containing family A member 1 OS=Homo sapiens GN=PLEKHA1 P | 201891 | 216781 | 254144 | 0,79 | 0,266739 | 0,85 | 0,334987 | 0,93 | 0,745747 |
| Q9HB19 | Pleckstrin homology domain-containing family A member 2 OS=Homo sapiens GN=PLEKHA2 P | 1529527 | 2231554 | 150317 | 10,18 | 0,018966 | 14,85 | 8,89E-05 | 0,69 | 0,113968 |
| Q9HAU0 | Pleckstrin homology domain-containing family A member 5 OS=Homo sapiens GN=PLEKHA5 P | 230005 | 286413 | 73049 | 3,15 | 0,244656 | 3,92 | 0,028524 | 0,80 | 0,634648 |
| Q96PX9 | Pleckstrin homology domain-containing family G member 4B OS=Homo sapiens GN=PLEKHG4B | 1467218 | 4210157 | 2989672 | 0,49 | 0,077983 | 1,41 | 0,33114 | 0,35 | 0,000701 |
| Q494U1 | Pleckstrin homology domain-containing family N member 1 OS=Homo sapiens GN=PLEKHN1 P | 3988948 | 6785337 | 8751779 | 0,46 | 0,045253 | 0,78 | 0,484512 | 0,59 | 0,050036 |
| Q86SQ0 | Pleckstrin homology-like domain family B member 2 OS=Homo sapiens GN=PHLDB2 PE=1 SV=2 | 554741 | 579357 | 12212 | 45,43 | 0,114183 | 47,44 | 0,003325 | 0,96 | 0,944533 |
| P21246 | Pleiotrophin OS=Homo sapiens GN=PTN PE=1 SV=1 - [PTN_HUMAN] | 132167 | 246708 | 131637 | 1,00 | 0,995289 | 1,87 | 0,134659 | 0,54 | 0,173443 |
| Q6UX71 | Plexin domain-containing protein 2 OS=Homo sapiens GN=PLXDC2 PE=1 SV=1 - [PXDC2_HUMA | 507987 | 503540 | 165857 | 3,06 | 0,041889 | 3,04 | 0,020541 | 1,01 | 0,980727 |
| O43157 | Plexin-B1 OS=Homo sapiens GN=PLXNB1 PE=1 SV=3 - [PLXB1_HUMAN] | 24177 | 42610 | 106 | 227,65 | 0,39985 | 401,21 | 0,03999 | 0,57 | 0,431957 |
| O15031 | Plexin-B2 OS=Homo sapiens GN=PLXNB2 PE=1 SV=3 - [PLXB2_HUMAN] | 401539 | 162740 | 146028 | 2,75 | 0,08837 | 1,11 | 0,751038 | 2,47 | 0,007072 |
| Q9BWT3 | Poly(A) polymerase gamma OS=Homo sapiens GN=PAPOLG PE=1 SV=2 - [PAPOG_HUMAN] | 18877 | 3399 | 821 | 23,00 | 0,196335 | 4,14 | 0,373634 | 5,55 | 0,078257 |
| Q86SE9 | Polycomb group RING finger protein 5 OS=Homo sapiens GN=PCGF5 PE=1 SV=1 - [PCGF5_HUM | 96409 | 58146 | 1132 | 85,18 | 0,064178 | 51,37 | 0,011607 | 1,66 | 0,291309 |
| P98161 | Polycystin-1 OS=Homo sapiens GN=PKD1 PE=1 SV=3 - [PKD1_HUMAN] | 10322 | 528 | 10985 | 0,94 | 0,957479 | 0,05 | 0,000514 | 19,54 | 0,125304 |
| Q9BY77 | Polymerase delta-interacting protein 3 OS=Homo sapiens GN=POLDIP3 PE=1 SV=2 - [PDIP3_HU | 16340 | 37309 | 53814 | 0,30 | 0,036905 | 0,69 | 0,552117 | 0,44 | 0,207137 |
| Q7Z7M9 | Polypeptide N-acetylgalactosaminyltransferase 5 OS=Homo sapiens GN=GALNT5 PE=1 SV=1 - [ | 10374624 | 21628170 | 48220448 | 0,22 | 0,000151 | 0,45 | 0,061495 | 0,48 | 0,143097 |
| Q8NCL4 | Polypeptide N-acetylgalactosaminyltransferase 6 OS=Homo sapiens GN=GALNT6 PE=2 SV=2 - [ | 1524396 | 1840620 | 1588106 | 0,96 | 0,902239 | 1,16 | 0,600747 | 0,83 | 0,391571 |
| Q8N945 | PRELI domain-containing protein 2 OS=Homo sapiens GN=PRELID2 PE=2 SV=1 - [PRLD2_HUMA | 92221901 | 41309964 | 163344177 | 0,56 | 0,022565 | 0,25 | 3E-07 | 2,23 | 0,005308 |
| Q7L014 | Probable ATP-dependent RNA helicase DDX46 OS=Homo sapiens GN=DDX46 PE=1 SV=2 - [DDX | 186712 | 87185 | 234934 | 0,79 | 0,737246 | 0,37 | 0,117658 | 2,14 | 0,233867 |
| Q9Y2G3 | Probable phospholipid-transporting ATPase IF OS=Homo sapiens GN=ATP11B PE=1 SV=2 - [AT1 | 1400 | 650 | 38493 | 0,04 | 0,137946 | 0,02 | 0,145817 | 2,16 | 0,675213 |
| Q9NY28 | Probable polypeptide N-acetylgalactosaminyltransferase 8 OS=Homo sapiens GN=GALNT8 PE= | 1891643 | 4428773 | 123273 | 15,35 | 0,096301 | 35,93 | 0,103572 | 0,43 | 0,138187 |
| Q9H3G5 | Probable serine carboxypeptidase CPVL OS=Homo sapiens GN=CPVL PE=1 SV=2 - [CPVL_HUMA | 1781747 | 2912808 | 582089 | 3,06 | 0,0367 | 5,00 | 0,000268 | 0,61 | 0,010978 |
| Q02809 | Procollagen-lysine,2-oxoglutarate 5-dioxygenase 1 OS=Homo sapiens GN=PLOD1 PE=1 SV=2 - [ | 14487 | 1983 | 18616 | 0,78 | 0,80494 | 0,11 | 0,0961 | 7,30 | 0,160222 |
| P51888 | Prolargin OS=Homo sapiens GN=PRELP PE=1 SV=1 - [PRELP_HUMAN] | 64880 | 57694 | 717 | 90,53 | 0,357258 | 80,50 | 0,100254 | 1,12 | 0,922328 |
| Q5FWE3 | Proline-rich transmembrane protein 3 OS=Homo sapiens GN=PRRT3 PE=1 SV=3 - [PRRT3_HUM | 316768123 | 259953746 | 448219751 | 0,71 | 0,064784 | 0,58 | 0,000265 | 1,22 | 0,267351 |
| Q9H939 | Proline-serine-threonine phosphatase-interacting protein 2 OS=Homo sapiens GN=PSTPIP2 PE | 250094 | 274472 | 89461 | 2,80 | 0,008577 | 3,07 | 0,007059 | 0,91 | 0,699208 |
| Q07954 | Prolow-density lipoprotein receptor-related protein 1 OS=Homo sapiens GN=LRP1 PE=1 SV=2 - | 5208 | 9155 | 35770 | 0,15 | 0,05582 | 0,26 | 0,095488 | 0,57 | 0,565439 |
| Q9UHG2 | ProSAAS OS=Homo sapiens GN=PCSK1N PE=1 SV=1 - [PCSK1_HUMAN] | 4539273 | 5524971 | 7504598 | 0,60 | 0,046075 | 0,74 | 0,186875 | 0,82 | 0,404883 |
| P07602 | Prosaposin OS=Homo sapiens GN=PSAP PE=1 SV=2 - [SAP_HUMAN] | 2066934 | 4667868 | 3826122 | 0,54 | 0,103602 | 1,22 | 0,182469 | 0,44 | 0,000147 |
| O15354 | Prosaposin receptor GPR37 OS=Homo sapiens GN=GPR37 PE=1 SV=2 - [GPR37_HUMAN] | 46875404 | 100084565 | 119182703 | 0,39 | 0,00032 | 0,84 | 0,312878 | 0,47 | 0,000147 |
| Q15185 | Prostaglandin E synthase 3 OS=Homo sapiens GN=PTGES3 PE=1 SV=1 - [TEBP_HUMAN] | 1132285 | 1106913 | 2233891 | 0,51 | 0,000146 | 0,50 | 5,98E-05 | 1,02 | 0,900556 |
| P43115 | Prostaglandin E2 receptor EP3 subtype OS=Homo sapiens GN=PTGER3 PE=2 SV=1 - [PE2R3_HU | 17573 | 3104 | 254929 | 0,07 | 0,155614 | 0,01 | 0,145975 | 5,66 | 0,173604 |
| P41222 | Prostaglandin-H2 D-isomerase OS=Homo sapiens GN=PTGDS PE=1 SV=1 - [PTGDS_HUMAN] | 522242177 | 606791405 | 316899128 | 1,65 | 0,082727 | 1,91 | 0,007777 | 0,86 | 0,405315 |
| Q7Z5M8 | Protein ABHD12B OS=Homo sapiens GN=ABHD12B PE=2 SV=1 - [AB12B_HUMAN] | 1153211 | 1551678 | 101815 | 11,33 | 0,018462 | 15,24 | 0,000234 | 0,74 | 0,263928 |
| P02760 | Protein AMBP OS=Homo sapiens GN=AMBP PE=1 SV=1 - [AMBP_HUMAN] | 6211489 | 9438425 | 24570836 | 0,25 | 2,44E-06 | 0,38 | 7,85E-05 | 0,66 | 0,253714 |
| Q6PGQ7 | Protein aurora borealis OS=Homo sapiens GN=BORA PE=1 SV=2 - [BORA_HUMAN] | 146280 | 362305 | 36415 | 4,02 | 0,263786 | 9,95 | 0,001353 | 0,40 | 0,005152 |
| Q8NG31 | Protein CASC5 OS=Homo sapiens GN=CASC5 PE=1 SV=3 - [CASC5_HUMAN] | 207312 | 179632 | 47118 | 4,40 | 0,019923 | 3,81 | 0,068147 | 1,15 | 0,703585 |
| Q13948 | Protein CASP OS=Homo sapiens GN=CUX1 PE=1 SV=2 - [CASP_HUMAN] | 559075 | 1573362 | 377103 | 1,48 | 0,601901 | 4,17 | 0,028524 | 0,36 | 0,003739 |
| O43439 | Protein CBFA2T2 OS=Homo sapiens GN=CBFA2T2 PE=1 SV=1 - [MTG8R_HUMAN] | 40915161 | 46166688 | 40515300 | 1,01 | 0,975586 | 1,14 | 0,573197 | 0,89 | 0,589112 |
| O60888 | Protein CutA OS=Homo sapiens GN=CUTA PE=1 SV=2 - [CUTA_HUMAN] | 134209 | 220571 | 31445 | 4,27 | 0,347073 | 7,01 | 0,093594 | 0,61 | 0,405173 |
| Q9P219 | Protein Daple OS=Homo sapiens GN=CCDC88C PE=1 SV=3 - [DAPLE_HUMAN] | 22332321 | 29192412 | 2159148 | 10,34 | 0,021788 | 13,52 | 0,019346 | 0,77 | 0,458619 |
| Q5JWR5 | Protein dopey-1 OS=Homo sapiens GN=DOPEY1 PE=2 SV=1 - [DOP1_HUMAN] | 716947 | 1227064 | 456544 | 1,57 | 0,772434 | 2,69 | 0,489144 | 0,58 | 0,614036 |
| Q9Y4F4 | Protein FAM179B OS=Homo sapiens GN=FAM179B PE=1 SV=4 - [F179B_HUMAN] | 1352790 | 2561655 | 577221 | 2,34 | 0,038051 | 4,44 | 0,00031 | 0,53 | 0,000519 |
| Q8IYM0 | Protein FAM186B OS=Homo sapiens GN=FAM186B PE=2 SV=2 - [F186B_HUMAN] | 211572 | 196783 | 354719 | 0,60 | 0,740926 | 0,55 | 0,65444 | 1,08 | 0,975774 |
| Q92520 | Protein FAM3C OS=Homo sapiens GN=FAM3C PE=1 SV=1 - [FAM3C_HUMAN] | 2614736 | 2036908 | 426201 | 6,13 | 0,000649 | 4,78 | 0,000529 | 1,28 | 0,21493 |
| O94915 | Protein furry homolog-like OS=Homo sapiens GN=FRYL PE=1 SV=2 - [FRYL_HUMAN] | 45105200 | 30200144 | 95849305 | 0,47 | 0,105191 | 0,32 | 0,014938 | 1,49 | 0,597402 |
| Q8N485 | Protein limb expression 1 homolog OS=Homo sapiens GN=LIX1 PE=2 SV=2 - [LIX1_HUMAN] | 3965378 | 1618360 | 1371958 | 2,89 | 0,211307 | 1,18 | 0,539389 | 2,45 | 0,073055 |
| Q86TB9 | Protein PAT1 homolog 1 OS=Homo sapiens GN=PATL1 PE=1 SV=2 - [PATL1_HUMAN] | 296023 | 501366 | 184255 | 1,61 | 0,581097 | 2,72 | 0,126778 | 0,59 | 0,208596 |
| Q96LQ0 | Protein phosphatase 1 regulatory subunit 36 OS=Homo sapiens GN=PPP1R36 PE=1 SV=1 - [PPR | 852635 | 663490 | 473523 | 1,80 | 0,06964 | 1,40 | 0,135284 | 1,29 | 0,186937 |
| A8MPX8 | Protein phosphatase 2C-like domain-containing protein 1 OS=Homo sapiens GN=PP2D1 PE=2 S | 2989993 | 3446440 | 5669932 | 0,53 | 1,23E-05 | 0,61 | 1,64E-06 | 0,87 | 0,200125 |
| P05109 | Protein S100-A8 OS=Homo sapiens GN=S100A8 PE=1 SV=1 - [S10A8_HUMAN] | 12982 | 76561 | 2442 | 5,32 | 0,540904 | 31,35 | 0,573067 | 0,17 | 0,463937 |
| Q9NVU7 | Protein SDA1 homolog OS=Homo sapiens GN=SDAD1 PE=1 SV=3 - [SDA1_HUMAN] | 1208500 | 2403723 | 21067 | 57,37 | 0,095154 | 114,10 | 0,031838 | 0,50 | 0,123459 |
| P18583 | Protein SON OS=Homo sapiens GN=SON PE=1 SV=4 - [SON_HUMAN] | 31518 | 1625 | 1935 | 16,29 | 0,137946 | 0,84 | 0,87651 | 19,40 | 0,011339 |
| Q5T011 | Protein SZT2 OS=Homo sapiens GN=SZT2 PE=1 SV=3 - [SZT2_HUMAN] | 2005941 | 2223660 | 1205382 | 1,66 | 0,341268 | 1,84 | 0,120028 | 0,90 | 0,777346 |
| Q9GZM5 | Protein YIPF3 OS=Homo sapiens GN=YIPF3 PE=1 SV=1 - [YIPF3_HUMAN] | 109024 | 119195 | 34102 | 3,20 | 0,209923 | 3,50 | 0,092559 | 0,91 | 0,878951 |
| Q86YA3 | Protein ZGRF1 OS=Homo sapiens GN=ZGRF1 PE=2 SV=3 - [ZGRF1_HUMAN] | 177060 | 150751 | 49012 | 3,61 | 0,10028 | 3,08 | 0,04174 | 1,17 | 0,703585 |
| P25116 | Proteinase-activated receptor 1 OS=Homo sapiens GN=F2R PE=1 SV=2 - [PAR1_HUMAN] | 213337687 | 216718929 | 208826860 | 1,02 | 0,934127 | 1,04 | 0,903143 | 0,98 | 0,962787 |

| | | | | | | | | | |
|---|---|---|---|---|---|---|---|---|---|
| P00734 | Prothrombin OS=Homo sapiens GN=F2 PE=1 SV=2 - [THRB_HUMAN] | 24043988 | 32216375 | 63237379 | 0,38 | 0,000364 | 0,51 | 0,004728 | 0,75 | 0,336631 |
| P20396 | Pro-thyrotropin-releasing hormone OS=Homo sapiens GN=TRH PE=1 SV=1 - [TRH_HUMAN] | 2459 | 12297 | 272381 | 0,01 | 0,021147 | 0,05 | 0,038531 | 0,20 | 0,429824 |
| Q9Y5E7 | Protocadherin beta-2 OS=Homo sapiens GN=PCDHB2 PE=1 SV=1 - [PCDB2_HUMAN] | 2744221 | 7368655 | 1249171 | 2,20 | 0,269424 | 5,90 | 2,03E-10 | 0,37 | 1,05E-06 |
| P07949 | Proto-oncogene tyrosine-protein kinase receptor Ret OS=Homo sapiens GN=RET PE=1 SV=3 - [ | 38913 | 35361 | 49242 | 0,79 | 0,743866 | 0,72 | 0,738394 | 1,10 | 0,928539 |
| P15498 | Proto-oncogene vav OS=Homo sapiens GN=VAV1 PE=1 SV=4 - [VAV_HUMAN] | 29128253 | 77090456 | 54628943 | 0,53 | 0,153221 | 1,41 | 0,029527 | 0,38 | 3,9E-05 |
| Q9H792 | Pseudopodium-enriched atypical kinase 1 OS=Homo sapiens GN=PEAK1 PE=1 SV=4 - [PEAK1_H | 173566878 | 175737884 | 280949669 | 0,62 | 0,09034 | 0,63 | 0,126757 | 0,99 | 0,980727 |
| Q9UJV8 | Purine-rich element-binding protein gamma OS=Homo sapiens GN=PURG PE=2 SV=1 - [PURG_ | 1303944 | 1250982 | 844539 | 1,54 | 0,456932 | 1,48 | 0,408184 | 1,04 | 0,918353 |
| P20848 | Putative alpha-1-antitrypsin-related protein OS=Homo sapiens GN=SERPINA2 PE=1 SV=1 - [A1A | 15245268 | 12281433 | 15403045 | 0,99 | 0,979783 | 0,80 | 0,423394 | 1,24 | 0,422389 |
| Q9BYX7 | Putative beta-actin-like protein 3 OS=Homo sapiens GN=POTEKP PE=5 SV=1 - [ACTBM_HUMAN | 71292 | 31480 | 234301 | 0,30 | 0,180286 | 0,13 | 0,105283 | 2,26 | 0,16551 |
| Q86T23 | Putative ciliary rootlet coiled-coil protein-like 1 protein OS=Homo sapiens GN=CROCCP2 PE=5 | 433563 | 289734 | 76191 | 5,69 | 0,252054 | 3,80 | 0,035889 | 1,50 | 0,542341 |
| A8MXQ7 | Putative IQ motif and ankyrin repeat domain-containing protein LOC642574 OS=Homo sapiens | 79725 | 155525 | 181589 | 0,44 | 0,110268 | 0,86 | 0,800181 | 0,51 | 0,233573 |
| A6NCF6 | Putative MAGE domain-containing protein MAGEA13P OS=Homo sapiens GN=MAGEA13P PE=5 | 1104 | 809 | 80194 | 0,01 | 0,097531 | 0,01 | 0,112174 | 1,37 | 0,767903 |
| Q9C0F0 | Putative Polycomb group protein ASXL3 OS=Homo sapiens GN=ASXL3 PE=2 SV=3 - [ASXL3_HUM | 81326 | 87230 | 2763 | 29,43 | 0,345889 | 31,57 | 0,11291 | 0,93 | 0,944831 |
| Q5T699 | Putative uncharacterized protein C6orf183 OS=Homo sapiens GN=C6orf183 PE=5 SV=3 - [CF18 | 356132 | 1060455 | 487 | 731,42 | 0,539775 | 2177,94 | 0,20127 | 0,34 | 0,278282 |
| Q8WXQ3 | Putative uncharacterized protein encoded by LINC01599 OS=Homo sapiens GN=LINC01599 PE | 325811 | 922170 | 84805 | 3,84 | 0,491518 | 10,87 | 0,014938 | 0,35 | 0,027965 |
| Q9H6N6 | Putative uncharacterized protein MYH16 OS=Homo sapiens GN=MYH16 PE=1 SV=2 - [MYH16_ | 2790048 | 2826874 | 1625092 | 1,72 | 0,150051 | 1,74 | 0,117106 | 0,99 | 0,964637 |
| P0CG00 | Putative zinc finger and SCAN domain-containing protein 5D OS=Homo sapiens GN=ZSCAN5D | 31003 | 34909 | 0 | #DIV/0! | 0,308395 | #DIV/0! | 0,043636 | 0,89 | 0,899113 |
| Q96N64 | PWWP domain-containing protein 2A OS=Homo sapiens GN=PWWP2A PE=1 SV=2 - [PWP2A_H | 49212 | 20414 | 2231265 | 0,02 | 4,21E-07 | 0,01 | 2,26E-06 | 2,41 | 0,720548 |
| P14618 | Pyruvate kinase PKM OS=Homo sapiens GN=PKM PE=1 SV=4 - [KPYM_HUMAN] | 180606 | 168877 | 98046 | 1,84 | 0,435639 | 1,72 | 0,316581 | 1,07 | 0,903565 |
| Q9H5N1 | Rab GTPase-binding effector protein 2 OS=Homo sapiens GN=RABEP2 PE=1 SV=2 - [RABE2_HU | 15247 | 13450 | 318079 | 0,05 | 0,002247 | 0,04 | 0,005217 | 1,13 | 0,926443 |
| Q14699 | Raftlin OS=Homo sapiens GN=RFTN1 PE=1 SV=4 - [RFTN1_HUMAN] | 10134 | 28148 | 237319 | 0,04 | 7,48E-07 | 0,12 | 0,000514 | 0,36 | 0,452011 |
| Q2PPJ7 | Ral GTPase-activating protein subunit alpha-2 OS=Homo sapiens GN=RALGAPA2 PE=1 SV=2 - [ | 1107763 | 1852063 | 3006316 | 0,37 | 3,02E-05 | 0,62 | 0,00447 | 0,60 | 0,008106 |
| Q8TEU7 | Rap guanine nucleotide exchange factor 6 OS=Homo sapiens GN=RAPGEF6 PE=1 SV=2 - [RPGF6 | 111032 | 128130 | 151393 | 0,73 | 0,740508 | 0,85 | 0,860012 | 0,87 | 0,875524 |
| Q96S79 | Ras-like protein family member 10B OS=Homo sapiens GN=RASL10B PE=2 SV=1 - [RSLAB_HUM | 303757 | 984700 | 448384 | 0,68 | 0,541052 | 2,20 | 0,237692 | 0,31 | 0,008961 |
| Q9BQQ7 | Receptor-transporting protein 3 OS=Homo sapiens GN=RTP3 PE=1 SV=1 - [RTP3_HUMAN] | 427654 | 1136483 | 854 | 500,95 | 0,321135 | 1331,28 | 0,035775 | 0,38 | 0,079174 |
| P23471 | Receptor-type tyrosine-protein phosphatase zeta OS=Homo sapiens GN=PTPRZ1 PE=1 SV=4 - [ | 406875 | 268915 | 485389 | 0,84 | 0,72754 | 0,55 | 0,237019 | 1,51 | 0,322182 |
| P49795 | Regulator of G-protein signaling 19 OS=Homo sapiens GN=RGS19 PE=1 SV=1 - [RGS19_HUMAN | 564990 | 588655 | 671619 | 0,84 | 0,748312 | 0,88 | 0,65444 | 0,96 | 0,944533 |
| P57771 | Regulator of G-protein signaling 8 OS=Homo sapiens GN=RGS8 PE=1 SV=1 - [RGS8_HUMAN] | 24804567 | 33120141 | 28712705 | 0,86 | 0,519693 | 1,15 | 0,321008 | 0,75 | 0,052444 |
| O75916 | Regulator of G-protein signaling 9 OS=Homo sapiens GN=RGS9 PE=1 SV=1 - [RGS9_HUMAN] | 10442534 | 23055310 | 3994036 | 2,61 | 0,518508 | 5,77 | 0,043152 | 0,45 | 0,124086 |
| Q96LZ7 | Regulator of microtubule dynamics protein 2 OS=Homo sapiens GN=RMDN2 PE=1 SV=2 - [RMD | 292309 | 810130 | 507564 | 0,58 | 0,419763 | 1,60 | 0,128158 | 0,36 | 0,006151 |
| Q9HAU5 | Regulator of nonsense transcripts 2 OS=Homo sapiens GN=UPF2 PE=1 SV=1 - [RENT2_HUMAN | 13546 | 15190 | 10800 | 1,25 | 0,820737 | 1,41 | 0,638192 | 0,89 | 0,898212 |
| O75787 | Renin receptor OS=Homo sapiens GN=ATP6AP2 PE=1 SV=2 - [RENR_HUMAN] | 880537 | 992340 | 262531 | 3,35 | 0,058672 | 3,78 | 0,044419 | 0,89 | 0,760808 |
| Q9BSG5 | Retbindin OS=Homo sapiens GN=RTBDN PE=2 SV=2 - [RTBDN_HUMAN] | 4419329 | 4012345 | 1203110 | 3,67 | 0,034898 | 3,33 | 0,024168 | 1,10 | 0,778565 |
| Q86UN2 | Reticulon-4 receptor-like 1 OS=Homo sapiens GN=RTN4RL1 PE=1 SV=1 - [R4RL1_HUMAN] | 36017563 | 119876831 | 38581223 | 0,93 | 0,926472 | 3,11 | 0,001193 | 0,30 | 3,9E-05 |
| P12271 | Retinaldehyde-binding protein 1 OS=Homo sapiens GN=RLBP1 PE=1 SV=2 - [RLBP1_HUMAN] | 593376 | 209805 | 352979 | 1,68 | 0,355353 | 0,59 | 0,223221 | 2,83 | 0,015406 |
| P49788 | Retinoic acid receptor responder protein 1 OS=Homo sapiens GN=RARRES1 PE=1 SV=2 - [TIG1_ | 40858 | 21522 | 82680 | 0,49 | 0,225506 | 0,26 | 0,051733 | 1,90 | 0,276928 |
| Q99969 | Retinoic acid receptor responder protein 2 OS=Homo sapiens GN=RARRES2 PE=1 SV=1 - [RARR | 2372136 | 2651619 | 1702339 | 1,39 | 0,209923 | 1,56 | 0,104277 | 0,89 | 0,615518 |
| Q8TC12 | Retinol dehydrogenase 11 OS=Homo sapiens GN=RDH11 PE=1 SV=2 - [RDH11_HUMAN] | 3038926 | 3782868 | 4899282 | 0,62 | 0,009037 | 0,77 | 0,122794 | 0,80 | 0,232853 |
| P10745 | Retinol-binding protein 3 OS=Homo sapiens GN=RBP3 PE=1 SV=2 - [RET3_HUMAN] | 719227913 | 907461772 | 586010479 | 1,23 | 0,493413 | 1,55 | 0,05491 | 0,79 | 0,193261 |
| P02753 | Retinol-binding protein 4 OS=Homo sapiens GN=RBP4 PE=1 SV=3 - [RET4_HUMAN] | 7024880 | 9271382 | 8257963 | 0,85 | 0,656092 | 1,12 | 0,538782 | 0,76 | 0,241913 |
| O15537 | Retinoschisin OS=Homo sapiens GN=RS1 PE=1 SV=2 - [XLRS1_HUMAN] | 10679148 | 16474452 | 21527452 | 0,50 | 0,077983 | 0,77 | 0,229306 | 0,65 | 0,189563 |
| Q53QZ3 | Rho GTPase-activating protein 15 OS=Homo sapiens GN=ARHGAP15 PE=1 SV=2 - [RHG15_HUM | 52238 | 184767 | 0 | #DIV/0! | 0,321135 | #DIV/0! | 0,054473 | 0,28 | 0,030709 |
| Q8N392 | Rho GTPase-activating protein 18 OS=Homo sapiens GN=ARHGAP18 PE=1 SV=3 - [RHG18_HUM | 465234 | 420545 | 1135455 | 0,41 | 0,000404 | 0,37 | 1,93E-06 | 1,11 | 0,743237 |
| Q5T5U3 | Rho GTPase-activating protein 21 OS=Homo sapiens GN=ARHGAP21 PE=1 SV=1 - [RHG21_HUN | 14913218 | 15869361 | 2348797 | 6,35 | 0,004248 | 6,76 | 0,009933 | 0,94 | 0,8601 |
| Q9HCE6 | Rho guanine nucleotide exchange factor 10-like protein OS=Homo sapiens GN=ARHGEF10L PE | 464347 | 532736 | 849305 | 0,55 | 0,181543 | 0,63 | 0,233434 | 0,87 | 0,784223 |
| Q9NZN5 | Rho guanine nucleotide exchange factor 12 OS=Homo sapiens GN=ARHGEF12 PE=1 SV=1 - [AR | 435638 | 399867 | 153048 | 2,85 | 0,22842 | 2,61 | 0,050322 | 1,09 | 0,871414 |
| Q9NRR4 | Ribonuclease 3 OS=Homo sapiens GN=DROSHA PE=1 SV=2 - [RNC_HUMAN] | 9891086 | 12565742 | 7764061 | 1,27 | 0,62636 | 1,62 | 0,453082 | 0,79 | 0,614036 |
| P07998 | Ribonuclease pancreatic OS=Homo sapiens GN=RNASE1 PE=1 SV=4 - [RNAS1_HUMAN] | 3671754 | 3449856 | 2878153 | 1,28 | 0,7799 | 1,20 | 0,708905 | 1,06 | 0,938084 |
| O00584 | Ribonuclease T2 OS=Homo sapiens GN=RNASET2 PE=1 SV=2 - [RNT2_HUMAN] | 254257 | 166171 | 143400 | 1,77 | 0,266739 | 1,16 | 0,70104 | 1,53 | 0,198485 |
| Q15418 | Ribosomal protein S6 kinase alpha-1 OS=Homo sapiens GN=RPS6KA1 PE=1 SV=2 - [KS6A1_HUN | 78142 | 118762 | 5439 | 14,37 | 0,239625 | 21,83 | 0,033126 | 0,66 | 0,457069 |
| P51812 | Ribosomal protein S6 kinase alpha-3 OS=Homo sapiens GN=RPS6KA3 PE=1 SV=1 - [KS6A3_HUN | 2042234 | 1762939 | 2546058 | 0,80 | 0,425815 | 0,69 | 0,190928 | 1,16 | 0,652245 |
| Q96MT1 | RING finger protein 145 OS=Homo sapiens GN=RNF145 PE=2 SV=2 - [RN145_HUMAN] | 24811 | 31488 | 516777 | 0,05 | 7,48E-05 | 0,06 | 0,00037 | 0,79 | 0,74924 |
| Q92541 | RNA polymerase-associated protein RTF1 homolog OS=Homo sapiens GN=RTF1 PE=1 SV=4 - [R | 352848 | 294757 | 1095850 | 0,32 | 0,004043 | 0,27 | 0,003208 | 1,20 | 0,779986 |
| Q8IXT5 | RNA-binding protein 12B OS=Homo sapiens GN=RBM12B PE=1 SV=2 - [RB12B_HUMAN] | 19619288 | 17589896 | 7776232 | 2,52 | 0,000679 | 2,26 | 7,85E-05 | 1,12 | 0,445197 |
| Q5T481 | RNA-binding protein 20 OS=Homo sapiens GN=RBM20 PE=1 SV=3 - [RBM20_HUMAN] | 2128991 | 2633657 | 2964550 | 0,72 | 0,567641 | 0,89 | 0,651887 | 0,81 | 0,642974 |
| Q59EK9 | RUN domain-containing protein 3A OS=Homo sapiens GN=RUNDC3A PE=1 SV=2 - [RUN3A_HU | 69474 | 114872 | 0 | #DIV/0! | 0,164293 | #DIV/0! | 0,009933 | 0,60 | 0,283833 |
| P21817 | Ryanodine receptor 1 OS=Homo sapiens GN=RYR1 PE=1 SV=3 - [RYR1_HUMAN] | 64834 | 212527 | 87524 | 0,74 | 0,648378 | 2,43 | 0,102872 | 0,31 | 0,000529 |
| Q15413 | Ryanodine receptor 3 OS=Homo sapiens GN=RYR3 PE=1 SV=3 - [RYR3_HUMAN] | 9468459 | 24488080 | 18710972 | 0,51 | 0,187756 | 1,31 | 0,19518 | 0,39 | 0,001315 |
| Q9NZJ4 | Sacsin OS=Homo sapiens GN=SACS PE=1 SV=2 - [SACS_HUMAN] | 7514 | 21747 | 184784 | 0,04 | 0,000798 | 0,12 | 0,013085 | 0,35 | 0,582136 |
| Q96ES7 | SAGA-associated factor 29 OS=Homo sapiens GN=SGF29 PE=1 SV=1 - [SGF29_HUMAN] | 7712188 | 2793330 | 1344593 | 5,74 | 0,07148 | 2,08 | 0,233434 | 2,76 | 0,021744 |
| P10523 | S-arrestin OS=Homo sapiens GN=SAG PE=1 SV=3 - [ARRS_HUMAN] | 754 | 1242 | 62103 | 0,01 | 0,019923 | 0,02 | 0,02957 | 0,61 | 0,615518 |
| P0C7P3 | Schlafen family member 14 OS=Homo sapiens GN=SLFN14 PE=2 SV=2 - [SLN14_HUMAN] | 4731746 | 10401692 | 8450106 | 0,56 | 0,092616 | 1,23 | 0,471995 | 0,45 | 0,000651 |
| Q9P0W5 | Schwannomin-interacting protein 1 OS=Homo sapiens GN=SCHIP1 PE=1 SV=1 - [SCHI1_HUMAI | 25809 | 23142 | 63671 | 0,41 | 0,12076 | 0,36 | 0,084637 | 1,12 | 0,885555 |
| Q15468 | SCL-interrupting locus protein OS=Homo sapiens GN=STIL PE=1 SV=2 - [STIL_HUMAN] | 265496 | 80136 | 1034868 | 0,26 | 0,009835 | 0,08 | 6,32E-06 | 3,31 | 0,266723 |
| Q8WVM8 | Sec1 family domain-containing protein 1 OS=Homo sapiens GN=SCFD1 PE=1 SV=4 - [SCFD1_HU | 3281523 | 621465 | 53311 | 61,55 | 0,021137 | 11,66 | 0,015693 | 5,28 | 0,001109 |
| Q86VW0 | SEC14 domain and spectrin repeat-containing protein 1 OS=Homo sapiens GN=SESTD1 PE=1 S\ | 89426 | 48030 | 29533 | 3,03 | 0,355982 | 1,63 | 0,523299 | 1,86 | 0,391571 |
| Q92765 | Secreted frizzled-related protein 3 OS=Homo sapiens GN=FRZB PE=1 SV=2 - [SFRP3_HUMAN] | 18583739 | 21976295 | 26446321 | 0,70 | 0,105191 | 0,83 | 0,376191 | 0,85 | 0,419918 |
| P05060 | Secretogranin-1 OS=Homo sapiens GN=CHGB PE=1 SV=2 - [SCG1_HUMAN] | 2187259 | 1988692 | 680747 | 3,21 | 0,123049 | 2,92 | 0,053164 | 1,10 | 0,834401 |
| P13521 | Secretogranin-2 OS=Homo sapiens GN=SCG2 PE=1 SV=2 - [SCG2_HUMAN] | 878467 | 760302 | 289742 | 3,03 | 0,092208 | 2,62 | 0,059311 | 1,16 | 0,707494 |
| Q8WXD2 | Secretogranin-3 OS=Homo sapiens GN=SCG3 PE=1 SV=3 - [SCG3_HUMAN] | 7181171 | 9745678 | 1170881 | 6,13 | 0,160748 | 8,32 | 0,009933 | 0,74 | 0,483498 |
| Q9BYH1 | Seizure 6-like protein OS=Homo sapiens GN=SEZ6L PE=1 SV=1 - [SE6L1_HUMAN] | 11260 | 9133 | 0 | #DIV/0! | 0,229997 | #DIV/0! | 0,113618 | 1,23 | 0,808152 |
| Q53EL9 | Seizure protein 6 homolog OS=Homo sapiens GN=SEZ6 PE=1 SV=2 - [SEZ6_HUMAN] | 1882071 | 1549716 | 442665 | 4,25 | 0,054873 | 3,50 | 0,015557 | 1,21 | 0,590084 |
| P49908 | Selenoprotein P OS=Homo sapiens GN=SEPP1 PE=1 SV=3 - [SEPP1_HUMAN] | 108249 | 90308 | 63746 | 1,70 | 0,614765 | 1,42 | 0,705349 | 1,20 | 0,834401 |
| Q14563 | Semaphorin-3A OS=Homo sapiens GN=SEMA3A PE=1 SV=1 - [SEM3A_HUMAN] | 10131 | 17923 | 65 | 154,97 | 0,316348 | 274,17 | 0,040029 | 0,57 | 0,38273 |
| Q13214 | Semaphorin-3B OS=Homo sapiens GN=SEMA3B PE=2 SV=1 - [SEM3B_HUMAN] | 16810 | 18084 | 609 | 27,59 | 0,209923 | 29,69 | 0,02022 | 0,93 | 0,926722 |
| Q13275 | Semaphorin-3F OS=Homo sapiens GN=SEMA3F PE=2 SV=2 - [SEM3F_HUMAN] | 162414 | 156871 | 65738 | 2,47 | 0,343132 | 2,39 | 0,113618 | 1,04 | 0,957902 |
| Q9NPR2 | Semaphorin-4B OS=Homo sapiens GN=SEMA4B PE=1 SV=3 - [SEM4B_HUMAN] | 372742 | 295423 | 32785 | 11,37 | 0,090974 | 9,01 | 0,035608 | 1,26 | 0,64799 |
| O75326 | Semaphorin-7A OS=Homo sapiens GN=SEMA7A PE=1 SV=1 - [SEM7A_HUMAN] | 5969374 | 5212406 | 2182541 | 2,74 | 0,108564 | 2,39 | 0,054497 | 1,15 | 0,725698 |
| Q9HC62 | Sentrin-specific protease 2 OS=Homo sapiens GN=SENP2 PE=1 SV=3 - [SENP2_HUMAN] | 24566630 | 19544834 | 32230330 | 0,76 | 0,24558 | 0,61 | 0,003485 | 1,26 | 0,32795 |
| Q16181 | Septin-7 OS=Homo sapiens GN=SEPT7 PE=1 SV=2 - [SEPT7_HUMAN] | 29657 | 224897 | 98955 | 0,30 | 0,279241 | 2,27 | 0,623122 | 0,13 | 0,21111 |
| Q6UWY2 | Serine protease 57 OS=Homo sapiens GN=PRSS57 PE=1 SV=2 - [PRS57_HUMAN] | 123189 | 248593 | 3722 | 33,10 | 0,06826 | 66,79 | 0,00072 | 0,50 | 0,015453 |
| Q92743 | Serine protease HTRA1 OS=Homo sapiens GN=HTRA1 PE=1 SV=1 - [HTRA1_HUMAN] | 584543 | 580916 | 1201225 | 0,49 | 0,004043 | 0,48 | 0,002079 | 1,01 | 0,980727 |
| Q9NQ38 | Serine protease inhibitor Kazal-type 5 OS=Homo sapiens GN=SPINK5 PE=1 SV=2 - [ISK5_HUMA | 81468761 | 32322798 | 58596913 | 1,39 | 0,490907 | 0,55 | 0,066409 | 2,52 | 0,015453 |
| Q13247 | Serine/arginine-rich splicing factor 6 OS=Homo sapiens GN=SRSF6 PE=1 SV=2 - [SRSF6_HUMAN | 29028 | 49095 | 9664 | 3,00 | 0,581097 | 5,08 | 0,10814 | 0,59 | 0,484209 |
| Q16513 | Serine/threonine-protein kinase N2 OS=Homo sapiens GN=PKN2 PE=1 SV=1 - [PKN2_HUMAN] | 367193 | 455900 | 607034 | 0,60 | 0,313331 | 0,75 | 0,567938 | 0,81 | 0,567721 |
| Q6P3R8 | Serine/threonine-protein kinase Nek5 OS=Homo sapiens GN=NEK5 PE=2 SV=1 - [NEK5_HUMAI | 2528 | 26185 | 28860 | 0,09 | 0,059599 | 0,91 | 0,909956 | 0,10 | 0,036427 |
| O95747 | Serine/threonine-protein kinase OSR1 OS=Homo sapiens GN=OXSR1 PE=1 SV=1 - [OXSR1_HUN | 354896 | 209719 | 196 | 1811,54 | 0,055491 | 1070,49 | 0,014938 | 1,69 | 0,266041 |
| Q9BRS2 | Serine/threonine-protein kinase RIO1 OS=Homo sapiens GN=RIOK1 PE=1 SV=2 - [RIOK1_HUMA | 32312077 | 26982286 | 20235742 | 1,60 | 0,207648 | 1,33 | 0,270946 | 1,20 | 0,494551 |
| P57059 | Serine/threonine-protein kinase SIK1 OS=Homo sapiens GN=SIK1 PE=1 SV=2 - [SIK1_HUMAN] | 4524899 | 3808076 | 6538459 | 0,69 | 0,240211 | 0,58 | 0,087102 | 1,19 | 0,668684 |
| Q15173 | Serine/threonine-protein phosphatase 2A 56 kDa regulatory subunit beta isoform OS=Homo sa | 14944 | 48695 | 0 | #DIV/0! | 0,160564 | #DIV/0! | 0,0079 | 0,31 | 0,003283 |
| Q8TF05 | Serine/threonine-protein phosphatase 4 regulatory subunit 1 OS=Homo sapiens GN=PPP4R1 P | 178798 | 126967 | 777754 | 0,23 | 2,69E-08 | 0,16 | 1,47E-07 | 1,41 | 0,483968 |
| Q13315 | Serine-protein kinase ATM OS=Homo sapiens GN=ATM PE=1 SV=4 - [ATM_HUMAN] | 9321298 | 17850356 | 26041955 | 0,36 | 0,008671 | 0,69 | 0,137907 | 0,52 | 0,086573 |
| P02787 | Serotransferrin OS=Homo sapiens GN=TF PE=1 SV=3 - [TRFE_HUMAN] | 2991882936 | 1765912759 | 2097086346 | 1,43 | 0,053074 | 0,84 | 0,111579 | 1,69 | 3,9E-05 |
| A8MV23 | Serpin E3 OS=Homo sapiens GN=SERPINE3 PE=2 SV=2 - [SERP3_HUMAN] | 45689 | 25098 | 1705 | 26,79 | 0,22931 | 14,72 | 0,316275 | 1,82 | 0,495235 |
| Q14140 | SERTA domain-containing protein 2 OS=Homo sapiens GN=SERTAD2 PE=1 SV=1 - [SRTD2_HUM | 802520 | 973700 | 322666 | 2,49 | 0,138943 | 3,02 | 0,091725 | 0,82 | 0,631571 |
| P02768 | Serum albumin OS=Homo sapiens GN=ALB PE=1 SV=2 - [ALBU_HUMAN] | 75502417661 | 74106970287 | 80692540173 | 0,94 | 0,7799 | 0,92 | 0,697634 | 1,02 | 0,948073 |
| P0DJI9 | Serum amyloid A-2 protein OS=Homo sapiens GN=SAA2 PE=1 SV=1 - [SAA2_HUMAN] | 56676 | 14042 | 78119 | 0,73 | 0,715489 | 0,18 | 0,038661 | 4,04 | 0,233518 |
| P35542 | Serum amyloid A-4 protein OS=Homo sapiens GN=SAA4 PE=1 SV=2 - [SAA4_HUMAN] | 64033 | 20599 | 197438 | 0,32 | 0,031418 | 0,10 | 3,94E-05 | 3,11 | 0,208754 |
| P27169 | Serum paraoxonase/arylesterase 1 OS=Homo sapiens GN=PON1 PE=1 SV=3 - [PON1_HUMAN] | 3747150 | 3611278 | 3296016 | 1,14 | 0,76164 | 1,10 | 0,882225 | 1,04 | 0,949415 |
| P04278 | Sex hormone-binding globulin OS=Homo sapiens GN=SHBG PE=1 SV=2 - [SHBG_HUMAN] | 8335991 | 7618348 | 3492956 | 2,39 | 0,021629 | 2,18 | 0,038629 | 1,09 | 0,738442 |

| | | | | | | | | | | |
|---|---|---|---|---|---|---|---|---|---|---|
| Q5TCZ1 | SH3 and PX domain-containing protein 2A OS=Homo sapiens GN=SH3PXD2A PE=1 SV=1 - [SPD | 227679 | 364489 | 611809 | 0,37 | 0,345889 | 0,60 | 0,531073 | 0,62 | 0,63529 |
| Q99519 | Sialidase-1 OS=Homo sapiens GN=NEU1 PE=1 SV=1 - [NEUR1_HUMAN] | 303651 | 513079 | 8708 | 34,87 | 0,117625 | 58,92 | 0,009036 | 0,59 | 0,204724 |
| Q5T5P2 | Sickle tail protein homolog OS=Homo sapiens GN=KIAA1217 PE=1 SV=2 - [SKT_HUMAN] | 5759608 | 6181444 | 5926970 | 0,97 | 0,894535 | 1,04 | 0,743851 | 0,93 | 0,693485 |
| O43166 | Signal-induced proliferation-associated 1-like protein 1 OS=Homo sapiens GN=SIPA1L1 PE=1 S | 175125 | 75687 | 71279 | 2,46 | 0,514871 | 1,06 | 0,950609 | 2,31 | 0,391571 |
| Q9NTI5 | Sister chromatid cohesion protein PDS5 homolog B OS=Homo sapiens GN=PDS5B PE=1 SV=1 - | 33260 | 35648 | 69512 | 0,48 | 0,092208 | 0,51 | 0,101594 | 0,93 | 0,900556 |
| O75093 | Slit homolog 1 protein OS=Homo sapiens GN=SLIT1 PE=2 SV=4 - [SLIT1_HUMAN] | 359310 | 366295 | 162272 | 2,21 | 0,174887 | 2,26 | 0,112742 | 0,98 | 0,963841 |
| Q96NL6 | Sodium channel and clathrin linker 1 OS=Homo sapiens GN=SCLT1 PE=1 SV=2 - [SCLT1_HUMAN | 3721772 | 9142708 | 3299192 | 1,13 | 0,768673 | 2,77 | 0,000203 | 0,41 | 3,38E-06 |
| Q96N87 | Sodium-dependent neutral amino acid transporter B(0)AT3 OS=Homo sapiens GN=SLC6A18 PE | 1280404 | 930736 | 4078457 | 0,31 | 0,019923 | 0,23 | 0,002102 | 1,38 | 0,743111 |
| Q8TBE7 | Solute carrier family 35 member G2 OS=Homo sapiens GN=SLC35G2 PE=1 SV=3 - [S35G2_HUM | 43520 | 56416 | 10749 | 4,05 | 0,209923 | 5,25 | 0,015693 | 0,77 | 0,568178 |
| P46721 | Solute carrier organic anion transporter family member 1A2 OS=Homo sapiens GN=SLCO1A2 P | 31214867 | 33558606 | 18286878 | 1,71 | 0,100458 | 1,84 | 0,028668 | 0,93 | 0,765009 |
| Q99523 | Sortilin OS=Homo sapiens GN=SORT1 PE=1 SV=3 - [SORT_HUMAN] | 523373 | 462440 | 651871 | 0,80 | 0,687386 | 0,71 | 0,474926 | 1,13 | 0,751144 |
| Q9Y5W7 | Sorting nexin-14 OS=Homo sapiens GN=SNX14 PE=1 SV=3 - [SNX14_HUMAN] | 20516 | 759 | 4248 | 4,83 | 0,450949 | 0,18 | 0,176377 | 27,01 | 0,137949 |
| Q96L92 | Sorting nexin-27 OS=Homo sapiens GN=SNX27 PE=1 SV=2 - [SNX27_HUMAN] | 1275112 | 103225 | 1122922 | 1,14 | 0,849509 | 0,09 | 5,26E-07 | 12,35 | 0,00908 |
| Q9Y5X3 | Sorting nexin-5 OS=Homo sapiens GN=SNX5 PE=1 SV=1 - [SNX5_HUMAN] | 102952 | 70525 | 62417 | 1,65 | 0,12363 | 1,13 | 0,648876 | 1,46 | 0,044563 |
| P09486 | SPARC OS=Homo sapiens GN=SPARC PE=1 SV=1 - [SPRC_HUMAN] | 320296 | 172323 | 755598 | 0,42 | 0,030677 | 0,23 | 0,003422 | 1,86 | 0,018172 |
| Q14515 | SPARC-like protein 1 OS=Homo sapiens GN=SPARCL1 PE=1 SV=2 - [SPRL1_HUMAN] | 6953710 | 6913341 | 3267296 | 2,13 | 0,166532 | 2,12 | 0,082675 | 1,01 | 0,9919 |
| P02549 | Spectrin alpha chain, erythrocytic 1 OS=Homo sapiens GN=SPTA1 PE=1 SV=5 - [SPTA1_HUMAN | 14965135 | 44265807 | 3317173 | 4,51 | 0,343132 | 13,34 | 0,001361 | 0,34 | 0,002407 |
| Q9NRC6 | Spectrin beta chain, non-erythrocytic 5 OS=Homo sapiens GN=SPTBN5 PE=1 SV=2 - [SPTN5_HU | 20426724 | 24035548 | 21150700 | 0,97 | 0,894535 | 1,14 | 0,579249 | 0,85 | 0,388965 |
| Q9HBV2 | Sperm acrosome membrane-associated protein 1 OS=Homo sapiens GN=SPACA1 PE=1 SV=1 - | 511559 | 641452 | 149297 | 3,43 | 0,038741 | 4,30 | 0,000135 | 0,80 | 0,340688 |
| Q6Q759 | Sperm-associated antigen 17 OS=Homo sapiens GN=SPAG17 PE=2 SV=1 - [SPG17_HUMAN] | 599958 | 675633 | 74195 | 8,09 | 0,038741 | 9,11 | 0,005672 | 0,89 | 0,760808 |
| Q96L03 | Spermatogenesis-associated protein 17 OS=Homo sapiens GN=SPATA17 PE=2 SV=1 - [SPT17_H | 34409 | 379043 | 410723 | 0,08 | 0,007364 | 0,92 | 0,926664 | 0,09 | 0,069387 |
| Q9UM82 | Spermatogenesis-associated protein 2 OS=Homo sapiens GN=SPATA2 PE=1 SV=2 - [SPAT2_HU | 53583 | 43296 | 133637 | 0,40 | 0,355982 | 0,32 | 0,044316 | 1,24 | 0,89911 |
| P0DKV0 | Spermatogenesis-associated protein 31C1 OS=Homo sapiens GN=SPATA31C1 PE=2 SV=1 - [S31 | 5809862 | 3784574 | 5737479 | 1,01 | 0,986072 | 0,66 | 0,296353 | 1,54 | 0,362102 |
| Q9P0W8 | Spermatogenesis-associated protein 7 OS=Homo sapiens GN=SPATA7 PE=1 SV=3 - [SPAT7_HU | 146190 | 225253 | 818 | 178,76 | 0,071249 | 275,44 | 0,001559 | 0,65 | 0,223973 |
| Q9BXG8 | Spermatogenic leucine zipper protein 1 OS=Homo sapiens GN=SPZ1 PE=1 SV=2 - [SPZ1_HUMA | 7827952 | 1704541 | 979850 | 7,99 | 0,059779 | 1,74 | 0,325499 | 4,59 | 0,004626 |
| P28290 | Sperm-specific antigen 2 OS=Homo sapiens GN=SSFA2 PE=1 SV=3 - [SSFA2_HUMAN] | 2271704 | 3162191 | 918250 | 2,47 | 5,79E-05 | 3,44 | 0,000903 | 0,72 | 0,008941 |
| Q9HCB6 | Spondin-1 OS=Homo sapiens GN=SPON1 PE=1 SV=2 - [SPON1_HUMAN] | 132096817 | 108111169 | 141015001 | 0,94 | 0,690472 | 0,77 | 0,014681 | 1,22 | 0,099352 |
| Q8NCJ5 | SPRY domain-containing protein 3 OS=Homo sapiens GN=SPRYD3 PE=1 SV=2 - [SPRY3_HUMAN | 39763974 | 39662015 | 26122107 | 1,52 | 0,428437 | 1,52 | 0,451595 | 1,00 | 0,997748 |
| Q9NY15 | Stabilin-1 OS=Homo sapiens GN=STAB1 PE=1 SV=3 - [STAB1_HUMAN] | 451979 | 815647 | 85555 | 5,28 | 0,279241 | 9,53 | 0,170789 | 0,55 | 0,391571 |
| Q9P2P6 | StAR-related lipid transfer protein 9 OS=Homo sapiens GN=STARD9 PE=1 SV=3 - [STAR9_HUMA | 2225654 | 3047024 | 1151132 | 1,93 | 0,009509 | 2,65 | 0,005695 | 0,73 | 0,038707 |
| Q8IY18 | Structural maintenance of chromosomes protein 5 OS=Homo sapiens GN=SMC5 PE=1 SV=2 - [S | 35818 | 53287 | 0 | #DIV/0! | 0,645892 | #DIV/0! | 0,468041 | 0,67 | 0,834987 |
| Q8IY92 | Structure-specific endonuclease subunit SLX4 OS=Homo sapiens GN=SLX4 PE=1 SV=3 - [SLX4_H | 569010 | 1028135 | 4684532 | 0,12 | 4,21E-07 | 0,22 | 5,34E-06 | 0,55 | 0,242331 |
| O00391 | Sulfhydryl oxidase 1 OS=Homo sapiens GN=QSOX1 PE=1 SV=3 - [QSOX1_HUMAN] | 316654 | 369401 | 46300 | 6,84 | 0,058268 | 7,98 | 0,048179 | 0,86 | 0,743237 |
| O95425 | Supervillin OS=Homo sapiens GN=SVIL PE=1 SV=2 - [SVIL_HUMAN] | 14436217 | 17967813 | 22318134 | 0,65 | 0,055949 | 0,81 | 0,376191 | 0,80 | 0,353755 |
| Q92797 | Symplekin OS=Homo sapiens GN=SYMPK PE=1 SV=2 - [SYMPK_HUMAN] | 1892036 | 344677 | 179432 | 10,54 | 0,343132 | 1,92 | 0,423394 | 5,49 | 0,183992 |
| O00186 | Syntaxin-binding protein 3 OS=Homo sapiens GN=STXBP3 PE=1 SV=2 - [STXB3_HUMAN] | 1642118 | 2323407 | 1562995 | 1,05 | 0,945543 | 1,49 | 0,260504 | 0,71 | 0,416319 |
| Q5T5C0 | Syntaxin-binding protein 5 OS=Homo sapiens GN=STXBP5 PE=1 SV=1 - [STXB5_HUMAN] | 4575 | 1263 | 18772 | 0,24 | 0,22176 | 0,07 | 0,125709 | 3,62 | 0,278282 |
| Q7Z7G0 | Target of Nesh-SH3 OS=Homo sapiens GN=ABI3BP PE=1 SV=1 - [TARSH_HUMAN] | 8434473 | 11666609 | 1831771 | 4,60 | 0,013135 | 6,37 | 0,03095 | 0,72 | 0,331604 |
| Q9ULW0 | Targeting protein for Xklp2 OS=Homo sapiens GN=TPX2 PE=1 SV=2 - [TPX2_HUMAN] | 65198 | 54511 | 10821 | 6,03 | 0,118161 | 5,04 | 0,166747 | 1,20 | 0,767903 |
| Q9NYW0 | Taste receptor type 2 member 10 OS=Homo sapiens GN=TAS2R10 PE=1 SV=3 - [T2R10_HUMAI | 2163851 | 729167 | 2184188 | 0,99 | 0,987682 | 0,33 | 0,112966 | 2,97 | 0,010827 |
| Q9Y2I9 | TBC1 domain family member 30 OS=Homo sapiens GN=TBC1D30 PE=1 SV=2 - [TBC30_HUMAN | 168172943 | 171023529 | 249633557 | 0,67 | 0,123893 | 0,69 | 0,148962 | 0,98 | 0,963841 |
| Q0IIM8 | TBC1 domain family member 8B OS=Homo sapiens GN=TBC1D8B PE=1 SV=2 - [TBC8B_HUMAN | 6450642 | 5419233 | 9529911 | 0,68 | 0,288669 | 0,57 | 0,089746 | 1,19 | 0,699995 |
| Q6ZT07 | TBC1 domain family member 9 OS=Homo sapiens GN=TBC1D9 PE=2 SV=2 - [TBCD9_HUMAN] | 253799 | 880723 | 218081 | 1,16 | 0,873697 | 4,04 | 0,112619 | 0,29 | 0,017109 |
| O43435 | T-box transcription factor TBX1 OS=Homo sapiens GN=TBX1 PE=1 SV=1 - [TBX1_HUMAN] | 547305 | 901663 | 1693157 | 0,32 | 7,7E-05 | 0,53 | 0,006227 | 0,61 | 0,088199 |
| P49368 | T-complex protein 1 subunit gamma OS=Homo sapiens GN=CCT3 PE=1 SV=4 - [TCPG_HUMAN] | 945646 | 2565663 | 740812 | 1,28 | 0,754948 | 3,46 | 0,054497 | 0,37 | 0,012465 |
| Q9NYB0 | Telomeric repeat-binding factor 2-interacting protein 1 OS=Homo sapiens GN=TERF2IP PE=1 S | 7819 | 2111 | 131412 | 0,06 | 0,001154 | 0,02 | 0,002167 | 3,70 | 0,212131 |
| P24821 | Tenascin OS=Homo sapiens GN=TNC PE=1 SV=3 - [TENA_HUMAN] | 120767 | 54007 | 35513 | 3,40 | 0,113536 | 1,52 | 0,511504 | 2,24 | 0,067782 |
| Q92752 | Tenascin-R OS=Homo sapiens GN=TNR PE=1 SV=3 - [TENR_HUMAN] | 1204689 | 1059391 | 1101144 | 1,09 | 0,84972 | 0,96 | 0,905487 | 1,14 | 0,767903 |
| Q68CZ2 | Tensin-3 OS=Homo sapiens GN=TNS3 PE=1 SV=2 - [TENS3_HUMAN] | 105557 | 157478 | 1169060 | 0,09 | 0,000137 | 0,13 | 0,003391 | 0,67 | 0,808152 |
| Q08629 | Testican-1 OS=Homo sapiens GN=SPOCK1 PE=1 SV=1 - [TICN1_HUMAN] | 7145650 | 8126187 | 3986185 | 1,79 | 0,179219 | 2,04 | 0,011068 | 0,88 | 0,63007 |
| Q92563 | Testican-2 OS=Homo sapiens GN=SPOCK2 PE=1 SV=1 - [TICN2_HUMAN] | 128206 | 186155 | 8337 | 15,38 | 0,371767 | 22,33 | 0,0729 | 0,69 | 0,631633 |
| Q9BQ16 | Testican-3 OS=Homo sapiens GN=SPOCK3 PE=1 SV=2 - [TICN3_HUMAN] | 13556 | 1580 | 337 | 40,19 | 0,598465 | 4,68 | 0,324453 | 8,58 | 0,488757 |
| Q86VY4 | Testis-specific Y-encoded-like protein 5 OS=Homo sapiens GN=TSPYL5 PE=1 SV=2 - [TSYL5_HUN | 393739 | 247345 | 1302462 | 0,30 | 5,79E-05 | 0,19 | 3E-07 | 1,59 | 0,147316 |
| P05452 | Tetranectin OS=Homo sapiens GN=CLEC3B PE=1 SV=3 - [TETN_HUMAN] | 3696631 | 2684542 | 2596952 | 1,42 | 0,422344 | 1,03 | 0,891328 | 1,38 | 0,278282 |
| Q8NG11 | Tetraspanin-14 OS=Homo sapiens GN=TSPAN14 PE=1 SV=1 - [TSN14_HUMAN] | 1268893 | 9584468 | 1757186 | 0,72 | 0,687454 | 5,45 | 0,211872 | 0,13 | 0,017839 |
| Q8NDW8 | Tetratricopeptide repeat protein 21A OS=Homo sapiens GN=TTC21A PE=2 SV=3 - [TT21A_HUM | 1676417 | 1878699 | 1502989 | 1,12 | 0,645892 | 1,25 | 0,371187 | 0,89 | 0,55886 |
| Q5SRH9 | Tetratricopeptide repeat protein 39A OS=Homo sapiens GN=TTC39A PE=2 SV=1 - [TT39A_HUM | 194193 | 262847 | 56842 | 3,42 | 0,540328 | 4,62 | 0,066868 | 0,74 | 0,722921 |
| Q6I9Y2 | THO complex subunit 7 homolog OS=Homo sapiens GN=THOC7 PE=1 SV=3 - [THOC7_HUMAN] | 22659150 | 26689996 | 38288944 | 0,59 | 0,002792 | 0,70 | 0,018289 | 0,85 | 0,337618 |
| Q9NXG2 | THUMP domain-containing protein 1 OS=Homo sapiens GN=THUMPD1 PE=1 SV=2 - [THUM1_H | 417592 | 352730 | 302334 | 1,38 | 0,39193 | 1,17 | 0,705349 | 1,18 | 0,594883 |
| Q6YHU6 | Thyroid adenoma-associated protein OS=Homo sapiens GN=THADA PE=1 SV=1 - [THADA_HUM | 2754254 | 3384567 | 3206840 | 0,86 | 0,645892 | 1,06 | 0,84746 | 0,81 | 0,428422 |
| P05543 | Thyroxine-binding globulin OS=Homo sapiens GN=SERPINA7 PE=1 SV=2 - [THBG_HUMAN] | 16584 | 6785 | 121 | 136,55 | 0,25729 | 55,87 | 0,095008 | 2,44 | 0,340345 |
| Q8WZ42 | Titin OS=Homo sapiens GN=TTN PE=1 SV=4 - [TITIN_HUMAN] | 25038847 | 26923662 | 130465382 | 0,19 | 2,47E-10 | 0,21 | 7,99E-09 | 0,93 | 0,745747 |
| O15455 | Toll-like receptor 3 OS=Homo sapiens GN=TLR3 PE=1 SV=1 - [TLR3_HUMAN] | 9573 | 3416 | 455866 | 0,02 | 0,03894 | 0,01 | 0,049409 | 2,80 | 0,548857 |
| O75674 | TOM1-like protein 1 OS=Homo sapiens GN=TOM1L1 PE=1 SV=2 - [TM1L1_HUMAN] | 90054 | 78317 | 0 | #DIV/0! | 0,003493 | #DIV/0! | 0,018289 | 1,15 | 0,724652 |
| Q9H497 | Torsin-3A OS=Homo sapiens GN=TOR3A PE=1 SV=1 - [TOR3A_HUMAN] | 3106844 | 2364707 | 4420768 | 0,70 | 0,209923 | 0,53 | 0,008696 | 1,31 | 0,385799 |
| P48553 | Trafficking protein particle complex subunit 10 OS=Homo sapiens GN=TRAPPC10 PE=1 SV=2 - [ | 777330 | 883782 | 75721 | 10,27 | 0,027169 | 11,67 | 0,050748 | 0,88 | 0,781877 |
| Q8NI51 | Transcriptional repressor CTCFL OS=Homo sapiens GN=CTCFL PE=1 SV=2 - [CTCFL_HUMAN] | 2007176 | 4524294 | 5976859 | 0,34 | 0,001242 | 0,76 | 0,423394 | 0,44 | 0,015453 |
| P61812 | Transforming growth factor beta-2 OS=Homo sapiens GN=TGFB2 PE=1 SV=1 - [TGFB2_HUMAN | 1110 | 547 | 21289 | 0,05 | 0,000581 | 0,03 | 0,001353 | 2,03 | 0,631395 |
| Q15582 | Transforming growth factor-beta-induced protein ig-h3 OS=Homo sapiens GN=TGFBI PE=1 SV= | 2416909 | 2376919 | 4255789 | 0,57 | 0,025186 | 0,56 | 0,049348 | 1,02 | 0,963841 |
| P49770 | Translation initiation factor eIF-2B subunit beta OS=Homo sapiens GN=EIF2B2 PE=1 SV=3 - [EI | 14854 | 37142 | 19081 | 0,78 | 0,752985 | 1,95 | 0,633961 | 0,40 | 0,39244 |
| Q8IUR5 | Transmembrane and TPR repeat-containing protein 1 OS=Homo sapiens GN=TMTC1 PE=1 SV= | 8932101 | 11568837 | 21872622 | 0,41 | 8,35E-05 | 0,53 | 0,018109 | 0,77 | 0,429824 |
| Q24JP5 | Transmembrane protein 132A OS=Homo sapiens GN=TMEM132A PE=1 SV=1 - [T132A_HUMAN | 5664553 | 7854394 | 7670946 | 0,74 | 0,024955 | 1,02 | 0,849639 | 0,72 | 0,000147 |
| Q9BXS4 | Transmembrane protein 59 OS=Homo sapiens GN=TMEM59 PE=1 SV=1 - [TMM59_HUMAN] | 147228 | 292202 | 16556 | 8,89 | 0,180826 | 17,65 | 0,022869 | 0,50 | 0,11504 |
| P02766 | Transthyretin OS=Homo sapiens GN=TTR PE=1 SV=1 - [TTHY_HUMAN] | 403799280 | 306740800 | 219795326 | 1,84 | 0,037714 | 1,40 | 0,08523 | 1,32 | 0,112739 |
| Q07283 | Trichohyalin OS=Homo sapiens GN=TCHH PE=1 SV=2 - [TRHY_HUMAN] | 153144986 | 171968358 | 108907441 | 1,41 | 0,209923 | 1,58 | 0,094159 | 0,89 | 0,590084 |
| Q9BT92 | Trichoplein keratin filament-binding protein OS=Homo sapiens GN=TCHP PE=1 SV=1 - [TCHP_H | 1320477 | 1130782 | 570209 | 2,32 | 0,209923 | 1,98 | 0,098957 | 1,17 | 0,705613 |
| Q9HCJ0 | Trinucleotide repeat-containing gene 6C protein OS=Homo sapiens GN=TNRC6C PE=1 SV=3 - [T | 77374013 | 74363103 | 71932882 | 1,08 | 0,79086 | 1,03 | 0,903143 | 1,04 | 0,894651 |
| O14773 | Tripeptidyl-peptidase 1 OS=Homo sapiens GN=TPP1 PE=1 SV=2 - [TPP1_HUMAN] | 9511448 | 9140646 | 3399327 | 2,80 | 0,021788 | 2,69 | 0,002468 | 1,04 | 0,892006 |
| Q86TN4 | tRNA 2'-phosphotransferase 1 OS=Homo sapiens GN=TRPT1 PE=1 SV=2 - [TRPT1_HUMAN] | 31781855 | 37789897 | 21902399 | 1,45 | 0,260383 | 1,73 | 0,083625 | 0,84 | 0,456221 |
| P00761 | Trypsin OS=Sus scrofa PE=1 SV=1 - [TRYP_PIG] | 308816546 | 347219336 | 106327077 | 2,90 | 0,046829 | 3,27 | 0,009933 | 0,89 | 0,703585 |
| Q14679 | Tubulin polyglutamylase TTLL4 OS=Homo sapiens GN=TTLL4 PE=1 SV=2 - [TTLL4_HUMAN] | 2579064 | 2270408 | 340498 | 7,57 | 0,002277 | 6,67 | 0,038409 | 1,14 | 0,724652 |
| B5MCY1 | Tudor domain-containing protein 15 OS=Homo sapiens GN=TDRD15 PE=2 SV=1 - [TDR15_HUM | 247321 | 109088 | 7557 | 32,73 | 0,133975 | 14,44 | 0,065978 | 2,27 | 0,207137 |
| Q9NNX1 | Tuftelin OS=Homo sapiens GN=TUFT1 PE=1 SV=1 - [TUFT1_HUMAN] | 486602980 | 33160534 | 4265973 | 114,07 | 0,056414 | 7,77 | 0,005672 | 14,67 | 0,002169 |
| P32019 | Type II inositol 1,4,5-trisphosphate 5-phosphatase OS=Homo sapiens GN=INPP5B PE=1 SV=4 - | 2745187 | 2800737 | 862724 | 3,18 | 0,033006 | 3,25 | 0,030234 | 0,98 | 0,959949 |
| P14679 | Tyrosinase OS=Homo sapiens GN=TYR PE=1 SV=3 - [TYRO_HUMAN] | 201837 | 2796 | 4926 | 40,97 | 0,042885 | 0,57 | 0,600677 | 72,19 | 0,000518 |
| Q9UIG0 | Tyrosine-protein kinase BAZ1B OS=Homo sapiens GN=BAZ1B PE=1 SV=2 - [BAZ1B_HUMAN] | 660971 | 945795 | 429092 | 1,54 | 0,419763 | 2,20 | 0,085094 | 0,70 | 0,237188 |
| P51451 | Tyrosine-protein kinase Blk OS=Homo sapiens GN=BLK PE=1 SV=3 - [BLK_HUMAN] | 2566147 | 5228898 | 8432298 | 0,30 | 2,69E-08 | 0,62 | 0,196387 | 0,49 | 0,079232 |
| P08631 | Tyrosine-protein kinase HCK OS=Homo sapiens GN=HCK PE=1 SV=5 - [HCK_HUMAN] | 53036 | 23514 | 28128 | 1,89 | 0,582273 | 0,84 | 0,84947 | 2,26 | 0,359249 |
| P54578 | Ubiquitin carboxyl-terminal hydrolase 14 OS=Homo sapiens GN=USP14 PE=1 SV=3 - [UBP14_H | 22811 | 2350 | 1321 | 17,27 | 0,508283 | 1,78 | 0,708508 | 9,71 | 0,344625 |
| Q9UPT9 | Ubiquitin carboxyl-terminal hydrolase 22 OS=Homo sapiens GN=USP22 PE=1 SV=2 - [UBP22_H | 4506525 | 5063150 | 16386539 | 0,28 | 8,35E-05 | 0,31 | 1,73E-05 | 0,89 | 0,811919 |
| Q9UPU5 | Ubiquitin carboxyl-terminal hydrolase 24 OS=Homo sapiens GN=USP24 PE=1 SV=3 - [UBP24_H | 10617252 | 10552749 | 20461872 | 0,52 | 0,009634 | 0,52 | 0,007868 | 1,01 | 0,991743 |
| Q9UHP3 | Ubiquitin carboxyl-terminal hydrolase 25 OS=Homo sapiens GN=USP25 PE=1 SV=4 - [UBP25_H | 424002 | 495239 | 10108 | 41,95 | 0,194929 | 48,99 | 0,034756 | 0,86 | 0,818528 |
| P40818 | Ubiquitin carboxyl-terminal hydrolase 8 OS=Homo sapiens GN=USP8 PE=1 SV=1 - [UBP8_HUM | 3823680 | 3030823 | 1958452 | 1,95 | 0,34549 | 1,55 | 0,489045 | 1,26 | 0,637322 |
| P22314 | Ubiquitin-like modifier-activating enzyme 1 OS=Homo sapiens GN=UBA1 PE=1 SV=3 - [UBA1_ | 6116521 | 6416472 | 5133522 | 1,19 | 0,698175 | 1,25 | 0,632595 | 0,95 | 0,926722 |
| O75752 | UDP-GalNAc:beta-1,3-N-acetylgalactosaminyltransferase 1 OS=Homo sapiens GN=B3GALNT1 | 15701 | 13976 | 593 | 26,48 | 0,348533 | 23,57 | 0,286799 | 1,12 | 0,926722 |
| P19224 | UDP-glucuronosyltransferase 1-6 OS=Homo sapiens GN=UGT1A6 PE=1 SV=2 - [UD16_HUMAN] | 71266 | 83094 | 28929 | 2,46 | 0,406106 | 2,87 | 0,170789 | 0,86 | 0,802535 |
| Q92628 | Uncharacterized protein KIAA0232 OS=Homo sapiens GN=KIAA0232 PE=1 SV=5 - [K0232_HUM | 29562 | 95197 | 9176 | 3,22 | 0,493413 | 10,37 | 0,071602 | 0,31 | 0,038564 |
| O60268 | Uncharacterized protein KIAA0513 OS=Homo sapiens GN=KIAA0513 PE=2 SV=1 - [K0513_HUM | 1093326 | 1018509 | 1545801 | 0,71 | 0,476677 | 0,66 | 0,140006 | 1,07 | 0,903339 |
| Q8NCT3 | Uncharacterized protein KIAA0895 OS=Homo sapiens GN=KIAA0895 PE=2 SV=4 - [K0895_HUM | 7451168 | 10143524 | 31499897 | 0,24 | 0,010921 | 0,32 | 0,020202 | 0,73 | 0,728258 |
| Q6NV74 | Uncharacterized protein KIAA1211-like OS=Homo sapiens GN=KIAA1211L PE=2 SV=3 - [K121L_ | 37894 | 36264 | 39528 | 0,96 | 0,957479 | 0,92 | 0,896954 | 1,04 | 0,944533 |

| | | | | | | | | | | |
|---|---|---|---|---|---|---|---|---|---|---|
| O43795 | Unconventional myosin-Ib OS=Homo sapiens GN=MYO1B PE=1 SV=3 - [MYO1B_HUMAN] | 2136917 | 1530274 | 1841964 | 1,16 | 0,621544 | 0,83 | 0,567938 | 1,40 | 0,21493 |
| O00160 | Unconventional myosin-If OS=Homo sapiens GN=MYO1F PE=1 SV=3 - [MYO1F_HUMAN] | 693609 | 669420 | 302947 | 2,29 | 0,356184 | 2,21 | 0,249837 | 1,04 | 0,959949 |
| Q9Y4I1 | Unconventional myosin-Va OS=Homo sapiens GN=MYO5A PE=1 SV=2 - [MYO5A_HUMAN] | 314925 | 1093701 | 382147 | 0,82 | 0,709689 | 2,86 | 0,012922 | 0,29 | 3,12E-06 |
| Q9NQX4 | Unconventional myosin-Vc OS=Homo sapiens GN=MYO5C PE=1 SV=2 - [MYO5C_HUMAN] | 181693 | 39805 | 183835 | 0,99 | 0,987002 | 0,22 | 0,000769 | 4,56 | 0,019619 |
| Q9UKN7 | Unconventional myosin-XV OS=Homo sapiens GN=MYO15A PE=1 SV=2 - [MYO15_HUMAN] | 127664 | 432033 | 3889663 | 0,03 | 0,005448 | 0,11 | 0,021204 | 0,30 | 0,173443 |
| Q8IUG5 | Unconventional myosin-XVIIIb OS=Homo sapiens GN=MYO18B PE=1 SV=1 - [MY18B_HUMAN] | 64077 | 22841 | 118636 | 0,54 | 0,158479 | 0,19 | 0,003069 | 2,81 | 0,015453 |
| O75445 | Usherin OS=Homo sapiens GN=USH2A PE=1 SV=3 - [USH2A_HUMAN] | 310258 | 434342 | 2027622 | 0,15 | 0,00477 | 0,21 | 0,022311 | 0,71 | 0,767419 |
| Q96RL7 | Vacuolar protein sorting-associated protein 13A OS=Homo sapiens GN=VPS13A PE=1 SV=2 - [V | 218190 | 709629 | 685528 | 0,32 | 0,07148 | 1,04 | 0,948728 | 0,31 | 0,015203 |
| Q7Z7G8 | Vacuolar protein sorting-associated protein 13B OS=Homo sapiens GN=VPS13B PE=1 SV=2 - [V | 10138934 | 8102177 | 4409710 | 2,30 | 0,095154 | 1,84 | 0,103736 | 1,25 | 0,445197 |
| Q8N1B4 | Vacuolar protein sorting-associated protein 52 homolog OS=Homo sapiens GN=VPS52 PE=1 SV | 94101 | 54880 | 388732 | 0,24 | 0,000339 | 0,14 | 6,32E-06 | 1,71 | 0,329587 |
| Q6EMK4 | Vasorin OS=Homo sapiens GN=VASN PE=1 SV=1 - [VASN_HUMAN] | 459100 | 364785 | 372484 | 1,23 | 0,691802 | 0,98 | 0,968752 | 1,26 | 0,512572 |
| P30291 | Wee1-like protein kinase OS=Homo sapiens GN=WEE1 PE=1 SV=2 - [WEE1_HUMAN] | 580708 | 640030 | 139693 | 4,16 | 0,02108 | 4,58 | 0,001617 | 0,91 | 0,743111 |
| P13611 | Versican core protein OS=Homo sapiens GN=VCAN PE=1 SV=3 - [CSPG2_HUMAN] | 11149234 | 13545022 | 2278223 | 4,89 | 0,123327 | 5,95 | 0,0274 | 0,82 | 0,665381 |
| Q12907 | Vesicular integral-membrane protein VIP36 OS=Homo sapiens GN=LMAN2 PE=1 SV=1 - [LMAN | 12918 | 7899 | 32 | 402,87 | 0,110268 | 246,34 | 0,101699 | 1,64 | 0,429824 |
| O15195 | Villin-like protein OS=Homo sapiens GN=VILL PE=2 SV=3 - [VILL_HUMAN] | 64054082 | 37755995 | 81769390 | 0,78 | 0,491518 | 0,46 | 0,004471 | 1,70 | 0,141008 |
| P02774 | Vitamin D-binding protein OS=Homo sapiens GN=GC PE=1 SV=1 - [VTDB_HUMAN] | 220045914 | 376548612 | 571288684 | 0,39 | 0,000679 | 0,66 | 0,072074 | 0,58 | 0,058568 |
| P04070 | Vitamin K-dependent protein C OS=Homo sapiens GN=PROC PE=1 SV=1 - [PROC_HUMAN] | 14770 | 138577 | 204534 | 0,07 | 0,019374 | 0,68 | 0,730626 | 0,11 | 0,236508 |
| P07225 | Vitamin K-dependent protein S OS=Homo sapiens GN=PROS1 PE=1 SV=1 - [PROS_HUMAN] | 3093378 | 1854466 | 1591403 | 1,94 | 0,116113 | 1,17 | 0,355925 | 1,67 | 0,030527 |
| P04004 | Vitronectin OS=Homo sapiens GN=VTN PE=1 SV=1 - [VTNC_HUMAN] | 31202788 | 30146302 | 47332152 | 0,66 | 0,072618 | 0,64 | 0,081583 | 1,04 | 0,926443 |
| Q9Y5W5 | Wnt inhibitory factor 1 OS=Homo sapiens GN=WIF1 PE=1 SV=3 - [WIF1_HUMAN] | 32093829 | 40875035 | 12215893 | 2,63 | 0,028644 | 3,35 | 0,009609 | 0,79 | 0,326329 |
| Q02641 | Voltage-dependent L-type calcium channel subunit beta-1 OS=Homo sapiens GN=CACNB1 PE= | 948298 | 1254404 | 1569448 | 0,60 | 0,074145 | 0,80 | 0,129142 | 0,76 | 0,157347 |
| Q8TAG5 | V-set and transmembrane domain-containing protein 2A OS=Homo sapiens GN=VSTM2A PE=2 | 80782 | 82614 | 822 | 98,29 | 0,114452 | 100,52 | 0,028524 | 0,98 | 0,975293 |
| Q93050 | V-type proton ATPase 116 kDa subunit a isoform 1 OS=Homo sapiens GN=ATP6V0A1 PE=1 SV= | 142482 | 43640 | 895208 | 0,16 | 0,054873 | 0,05 | 0,022266 | 3,26 | 0,479154 |
| Q15904 | V-type proton ATPase subunit S1 OS=Homo sapiens GN=ATP6AP1 PE=1 SV=2 - [VAS1_HUMAN] | 58898362 | 53520426 | 24568060 | 2,40 | 0,033296 | 2,18 | 0,007681 | 1,10 | 0,705613 |
| Q86Y38 | Xylosyltransferase 1 OS=Homo sapiens GN=XYLT1 PE=1 SV=1 - [XYLT1_HUMAN] | 62338 | 45156 | 3854 | 16,17 | 0,173241 | 11,72 | 0,079138 | 1,38 | 0,632359 |
| P49750 | YLP motif-containing protein 1 OS=Homo sapiens GN=YLPM1 PE=1 SV=3 - [YLPM1_HUMAN] | 60900 | 168630 | 11425 | 5,33 | 0,240687 | 14,76 | 0,002509 | 0,36 | 0,003225 |
| Q7Z2W4 | Zinc finger CCCH-type antiviral protein 1 OS=Homo sapiens GN=ZC3HAV1 PE=1 SV=3 - [ZCCHV_ | 893640 | 1385891 | 405932 | 2,20 | 0,083657 | 3,41 | 0,001353 | 0,64 | 0,022179 |
| Q15911 | Zinc finger homeobox protein 3 OS=Homo sapiens GN=ZFHX3 PE=1 SV=2 - [ZFHX3_HUMAN] | 59974 | 111970 | 335825 | 0,18 | 0,000105 | 0,33 | 0,04339 | 0,54 | 0,492429 |
| Q86UP3 | Zinc finger homeobox protein 4 OS=Homo sapiens GN=ZFHX4 PE=1 SV=1 - [ZFHX4_HUMAN] | 381034 | 423208 | 206573 | 1,84 | 0,150051 | 2,05 | 0,050307 | 0,90 | 0,724652 |
| Q5VZL5 | Zinc finger MYM-type protein 4 OS=Homo sapiens GN=ZMYM4 PE=1 SV=1 - [ZMYM4_HUMAN] | 21499 | 38132 | 12119 | 1,77 | 0,748312 | 3,15 | 0,177821 | 0,56 | 0,492429 |
| O14628 | Zinc finger protein 195 OS=Homo sapiens GN=ZNF195 PE=1 SV=2 - [ZN195_HUMAN] | 8656923 | 7366716 | 92950626 | 0,09 | 5,92E-10 | 0,08 | 7,99E-09 | 1,18 | 0,728589 |
| Q86WZ6 | Zinc finger protein 227 OS=Homo sapiens GN=ZNF227 PE=1 SV=1 - [ZN227_HUMAN] | 2113675 | 5902922 | 1977203 | 1,07 | 0,885474 | 2,99 | 0,00074 | 0,36 | 1,05E-06 |
| Q2VY69 | Zinc finger protein 284 OS=Homo sapiens GN=ZNF284 PE=2 SV=1 - [ZN284_HUMAN] | 360839 | 237250 | 605338 | 0,60 | 0,146028 | 0,39 | 0,022425 | 1,52 | 0,384056 |
| Q96SE7 | Zinc finger protein 347 OS=Homo sapiens GN=ZNF347 PE=1 SV=2 - [ZN347_HUMAN] | 65293 | 95735 | 1101819 | 0,06 | 0,064755 | 0,09 | 0,089191 | 0,68 | 0,642974 |
| Q02386 | Zinc finger protein 45 OS=Homo sapiens GN=ZNF45 PE=2 SV=2 - [ZNF45_HUMAN] | 1041158 | 663040 | 1836787 | 0,57 | 0,015012 | 0,36 | 0,000239 | 1,57 | 0,020218 |
| Q96JG9 | Zinc finger protein 469 OS=Homo sapiens GN=ZNF469 PE=2 SV=3 - [ZN469_HUMAN] | 967556 | 1647842 | 1318801 | 0,73 | 0,679617 | 1,25 | 0,740073 | 0,59 | 0,39244 |
| Q5JVG2 | Zinc finger protein 484 OS=Homo sapiens GN=ZNF484 PE=1 SV=1 - [ZN484_HUMAN] | 22762660 | 7180295 | 48081892 | 0,47 | 0,101401 | 0,15 | 2,28E-12 | 3,17 | 0,108567 |
| Q8TCN5 | Zinc finger protein 507 OS=Homo sapiens GN=ZNF507 PE=1 SV=2 - [ZN507_HUMAN] | 6961748 | 18715960 | 13783857 | 0,51 | 0,110446 | 1,36 | 0,579249 | 0,37 | 0,021442 |
| Q96ME7 | Zinc finger protein 512 OS=Homo sapiens GN=ZNF512 PE=1 SV=2 - [ZN512_HUMAN] | 2281909 | 6822903 | 853431 | 2,67 | 0,5356 | 7,99 | 0,014381 | 0,33 | 0,013693 |
| Q9H707 | Zinc finger protein 552 OS=Homo sapiens GN=ZNF552 PE=1 SV=2 - [ZN552_HUMAN] | 455369141 | 68545638 | 29804070 | 15,28 | 0,08728 | 2,30 | 0,023448 | 6,64 | 0,007108 |
| Q8TF20 | Zinc finger protein 721 OS=Homo sapiens GN=ZNF721 PE=2 SV=2 - [ZN721_HUMAN] | 94576 | 118035 | 1642880 | 0,06 | 1,23E-05 | 0,07 | 0,000184 | 0,80 | 0,86233 |
| Q13129 | Zinc finger protein Rlf OS=Homo sapiens GN=RLF PE=1 SV=2 - [RLF_HUMAN] | 548446 | 659353 | 541792 | 1,01 | 0,986072 | 1,22 | 0,649832 | 0,83 | 0,633636 |
| P25311 | Zinc-alpha-2-glycoprotein OS=Homo sapiens GN=AZGP1 PE=1 SV=2 - [ZA2G_HUMAN] | 1559848 | 1563700 | 1615800 | 0,97 | 0,886353 | 0,97 | 0,890139 | 1,00 | 0,994917 |
