# Supplementary material for: Systems pathology analysis identifies neurodegenerative nature of age‐related vitreoretinal interface diseases
Source: Aging Cell. 2018 Jul 2;17(5):e12809. doi: 10.1111/acel.12809 (PMC6156470; doi:10.1111/acel.12809)
Supplement: Supplementary file 8 [file ACEL-17-e12809-s008.pdf]

Supplemental Table S5: Significantly differed proteins between iERM and DME proteomes, q-value < 0.05.
80 proteins were present at higher level (red) and 131 proteins at lower level (yellow) in the iERM proteome when compared to DME proteome, the abundance ratio >2.

Accession	Description	Average of MS1 Intensities iERM	MH	DME	Fold differences iERM vs DME
Q9Y646	Carboxypeptidase Q OS=Homo sapiens GN=CPQ PE=1 SV=1 - [CBPQ_HUMAN]	37151	28393	0	#DIV/0!
O75674	TOM1-like protein 1 OS=Homo sapiens GN=TOM1L1 PE=1 SV=2 - [TM1L1_HUMAN]	90054	78317	0	#DIV/0!
Q14571	Inositol 1,4,5-trisphosphate receptor type 2 OS=Homo sapiens GN=ITPR2 PE=1 SV=2 - [ITPR2_HUMAN]	134674	176048	550	244,94
Q9UQB3	Catenin delta-2 OS=Homo sapiens GN=CTNND2 PE=1 SV=3 - [CTND2_HUMAN]	5689028	6086147	41622	136,68
Q02817	Mucin-2 OS=Homo sapiens GN=MUC2 PE=1 SV=2 - [MUC2_HUMAN]	53439520	12623677	596829	89,54
Q9Y287	Integral membrane protein 2B OS=Homo sapiens GN=ITM2B PE=1 SV=1 - [ITM2B_HUMAN]	1728182	1648716	22315	77,44
Q8WVM8	Sec1 family domain-containing protein 1 OS=Homo sapiens GN=SCFD1 PE=1 SV=4 - [SCFD1_HUMAN]	3281523	621465	53311	61,55
Q07002	Cyclin-dependent kinase 18 OS=Homo sapiens GN=CDK18 PE=1 SV=3 - [CDK18_HUMAN]	135425	23654	3205	42,26
P14679	Tyrosinase OS=Homo sapiens GN=TYR PE=1 SV=3 - [TYRO_HUMAN]	201837	2796	4926	40,97
P26006	Integrin alpha-3 OS=Homo sapiens GN=ITGA3 PE=1 SV=5 - [ITA3_HUMAN]	617077	220599	18529	33,30
P41271	Neuroblastoma suppressor of tumorigenicity 1 OS=Homo sapiens GN=NBL1 PE=1 SV=2 - [NBL1_HUMAN]	170603	234399	5694	29,96
Q9UPS6	Histone-lysine N-methyltransferase SETD1B OS=Homo sapiens GN=SETD1B PE=1 SV=3 - [SET1B_HUMAN]	466084	735319	16659	27,98
P55289	Cadherin-12 OS=Homo sapiens GN=CDH12 PE=2 SV=2 - [CAD12_HUMAN]	3572765	4479107	139150	25,68
Q96DT5	Dynein heavy chain 11, axonemal OS=Homo sapiens GN=DNAH11 PE=1 SV=4 - [DYH11_HUMAN]	6820149	10184163	268176	25,43
Q9NZP8	Complement C1r subcomponent-like protein OS=Homo sapiens GN=C1RL PE=1 SV=2 - [C1RL_HUMAN]	65413	26464	2635	24,82
P58107	Epiplakin OS=Homo sapiens GN=EPPK1 PE=1 SV=2 - [EPIPL_HUMAN]	18463802	20499266	778990	23,70
Q86SQ4	G-protein coupled receptor 126 OS=Homo sapiens GN=GPR126 PE=1 SV=3 - [GP126_HUMAN]	173406	91246	7430	23,34
Q14995	Nuclear receptor subfamily 1 group D member 2 OS=Homo sapiens GN=NR1D2 PE=1 SV=3 - [NR1D2_HUMA	14192048	14080864	637060	22,28
Q86UX2	Inter-alpha-trypsin inhibitor heavy chain H5 OS=Homo sapiens GN=ITIH5 PE=2 SV=2 - [ITIH5_HUMAN]	121068	136493	6166	19,63
P48552	Nuclear receptor-interacting protein 1 OS=Homo sapiens GN=NRIP1 PE=1 SV=2 - [NRIP1_HUMAN]	64019	97081	3317	19,30
Q96HY6	DDRGK domain-containing protein 1 OS=Homo sapiens GN=DDRGK1 PE=1 SV=2 - [DDRGK_HUMAN]	52799	13005	2808	18,80
O75063	Glycosaminoglycan xylosylkinase OS=Homo sapiens GN=FAM20B PE=1 SV=1 - [XYLK_HUMAN]	854683	290223	48227	17,72
P20929	Nebulin OS=Homo sapiens GN=NEB PE=1 SV=5 - [NEBU_HUMAN]	35937405	12357649	2104757	17,07
Q14533	Keratin, type II cuticular Hb1 OS=Homo sapiens GN=KRT81 PE=1 SV=3 - [KRT81_HUMAN]	140213	112520	8575	16,35
P02458	Collagen alpha-1(II) chain OS=Homo sapiens GN=COL2A1 PE=1 SV=3 - [CO2A1_HUMAN]	529190	356993	45740	11,57
Q7Z5M8	Protein ABHD12B OS=Homo sapiens GN=ABHD12B PE=2 SV=1 - [AB12B_HUMAN]	1153211	1551678	101815	11,33
O15240	Neurosecretory protein VGF OS=Homo sapiens GN=VGF PE=1 SV=2 - [VGF_HUMAN]	1434844	1291751	135982	10,55
Q9P219	Protein Daple OS=Homo sapiens GN=CCDC88C PE=1 SV=3 - [DAPLE_HUMAN]	22332321	29192412	2159148	10,34
P48553	Trafficking protein particle complex subunit 10 OS=Homo sapiens GN=TRAPPC10 PE=1 SV=2 - [TPC10_HUM/	777330	883782	75721	10,27
Q9HB19	Pleckstrin homology domain-containing family A member 2 OS=Homo sapiens GN=PLEKHA2 PE=1 SV=2 - [PI	1529527	2231554	150317	10,18
Q9UHB6	LIM domain and actin-binding protein 1 OS=Homo sapiens GN=LIMA1 PE=1 SV=1 - [LIMA1_HUMAN]	2268924	673378	228998	9,91
Q8N1I0	Dedicator of cytokinesis protein 4 OS=Homo sapiens GN=DOCK4 PE=1 SV=3 - [DOCK4_HUMAN]	4653239	737564	486392	9,57
Q15818	Neuronal pentraxin-1 OS=Homo sapiens GN=NPTX1 PE=2 SV=2 - [NPTX1_HUMAN]	459172	858915	48040	9,56
O94880	PHD finger protein 14 OS=Homo sapiens GN=PHF14 PE=1 SV=2 - [PHF14_HUMAN]	10644581	14864652	1145820	9,29
Q6Q759	Sperm-associated antigen 17 OS=Homo sapiens GN=SPAG17 PE=2 SV=1 - [SPG17_HUMAN]	599958	675633	74195	8,09
Q14679	Tubulin polyglutamylase TTLL4 OS=Homo sapiens GN=TTLL4 PE=1 SV=2 - [TTLL4_HUMAN]	2579064	2270408	340498	7,57
Q8NE71	ATP-binding cassette sub-family F member 1 OS=Homo sapiens GN=ABCF1 PE=1 SV=2 - [ABCF1_HUMAN]	5873295	13299858	842580	6,97
Q5T5U3	Rho GTPase-activating protein 21 OS=Homo sapiens GN=ARHGAP21 PE=1 SV=1 - [RHG21_HUMAN]	14913218	15869361	2348797	6,35
Q92520	Protein FAM3C OS=Homo sapiens GN=FAM3C PE=1 SV=1 - [FAM3C_HUMAN]	2614736	2036908	426201	6,13
Q8N137	Centrobin OS=Homo sapiens GN=CNTROB PE=1 SV=1 - [CNTRB_HUMAN]	8405583	2568220	1576459	5,33
Q92823	Neuronal cell adhesion molecule OS=Homo sapiens GN=NRCAM PE=1 SV=3 - [NRCAM_HUMAN]	3324184	2524496	653637	5,09
Q9UBZ9	DNA repair protein REV1 OS=Homo sapiens GN=REV1 PE=1 SV=1 - [REV1_HUMAN]	714503	724574	145228	4,92
Q6A162	Keratin, type I cytoskeletal 40 OS=Homo sapiens GN=KRT40 PE=1 SV=2 - [K1C40_HUMAN]	18684961	6171587	3934075	4,75
Q14746	Conserved oligomeric Golgi complex subunit 2 OS=Homo sapiens GN=COG2 PE=1 SV=1 - [COG2_HUMAN]	4063257	1754031	860562	4,72
P16519	Neuroendocrine convertase 2 OS=Homo sapiens GN=PCSK2 PE=2 SV=2 - [NEC2_HUMAN]	617401	503946	131950	4,68
Q7Z7G0	Target of Nesh-SH3 OS=Homo sapiens GN=ABI3BP PE=1 SV=1 - [TARSH_HUMAN]	8434473	11666609	1831771	4,60
P01763	Ig heavy chain V-III region WEA OS=Homo sapiens PE=1 SV=1 - [HV302_HUMAN]	195198	128735	42588	4,58
Q8NG31	Protein CASC5 OS=Homo sapiens GN=CASC5 PE=1 SV=3 - [CASC5_HUMAN]	207312	179632	47118	4,40
P30291	Wee1-like protein kinase OS=Homo sapiens GN=WEE1 PE=1 SV=2 - [WEE1_HUMAN]	580708	640030	139693	4,16
P51693	Amyloid-like protein 1 OS=Homo sapiens GN=APLP1 PE=1 SV=3 - [APLP1_HUMAN]	6082322	5302804	1574277	3,86
P54756	Ephrin type-A receptor 5 OS=Homo sapiens GN=EPHA5 PE=1 SV=3 - [EPHA5_HUMAN]	91257544	21631146	23743593	3,84
Q96HE7	ERO1-like protein alpha OS=Homo sapiens GN=ERO1A PE=1 SV=2 - [ERO1A_HUMAN]	3003385	3396555	794614	3,78
Q9BSG5	Retbindin OS=Homo sapiens GN=RTBDN PE=2 SV=2 - [RTBDN_HUMAN]	4419329	4012345	1203110	3,67
Q8IVF6	Ankyrin repeat domain-containing protein 18A OS=Homo sapiens GN=ANKRD18A PE=2 SV=3 - [AN18A_HUN	331102	308070	95467	3,47
Q9HBV2	Sperm acrosome membrane-associated protein 1 OS=Homo sapiens GN=SPACA1 PE=1 SV=1 - [SACA1_HUM	511559	641452	149297	3,43
Q9H1K4	Mitochondrial glutamate carrier 2 OS=Homo sapiens GN=SLC25A18 PE=1 SV=1 - [GHC2_HUMAN]	19722057	26976893	5780657	3,41
Q16706	Alpha-mannosidase 2 OS=Homo sapiens GN=MAN2A1 PE=1 SV=2 - [MA2A1_HUMAN]	5127108	5363524	1563133	3,28
P43251	Biotinidase OS=Homo sapiens GN=BTD PE=1 SV=2 - [BTD_HUMAN]	8615634	8601553	2706339	3,18
P32019	Type II inositol 1,4,5-trisphosphate 5-phosphatase OS=Homo sapiens GN=INPP5B PE=1 SV=4 - [I5P2_HUMAI	2745187	2800737	862724	3,18
Q6UX71	Plexin domain-containing protein 2 OS=Homo sapiens GN=PLXDC2 PE=1 SV=1 - [PXDC2_HUMAN]	507987	503540	165857	3,06
Q9H3G5	Probable serine carboxypeptidase CPVL OS=Homo sapiens GN=CPVL PE=1 SV=2 - [CPVL_HUMAN]	1781747	2912808	582089	3,06
Q9UFE4	Coiled-coil domain-containing protein 39 OS=Homo sapiens GN=CCDC39 PE=2 SV=3 - [CCD39_HUMAN]	2873277	2131724	956850	3,00
P16870	Carboxypeptidase E OS=Homo sapiens GN=CPE PE=1 SV=1 - [CBPE_HUMAN]	20748199	19624878	7036285	2,95
P46108	Adapter molecule crk OS=Homo sapiens GN=CRK PE=1 SV=2 - [CRK_HUMAN]	4579245	4919238	1577169	2,90
O94985	Calsyntenin-1 OS=Homo sapiens GN=CLSTN1 PE=1 SV=1 - [CSTN1_HUMAN]	64267366	63911758	22608562	2,84
P19022	Cadherin-2 OS=Homo sapiens GN=CDH2 PE=1 SV=4 - [CADH2_HUMAN]	3203031	2695055	1132667	2,83
O14773	Tripeptidyl-peptidase 1 OS=Homo sapiens GN=TPP1 PE=1 SV=2 - [TPP1_HUMAN]	9511448	9140646	3399327	2,80
Q9H939	Proline-serine-threonine phosphatase-interacting protein 2 OS=Homo sapiens GN=PSTPIP2 PE=1 SV=4 - [PPI	250094	274472	89461	2,80
Q9P121	Neurotrimin OS=Homo sapiens GN=NTM PE=1 SV=1 - [NTRI_HUMAN]	391764	314068	148381	2,64
Q9Y5W5	Wnt inhibitory factor 1 OS=Homo sapiens GN=WIF1 PE=1 SV=3 - [WIF1_HUMAN]	32093829	40875035	12215893	2,63
Q9UHB4	NADPH-dependent diflavin oxidoreductase 1 OS=Homo sapiens GN=NDOR1 PE=1 SV=1 - [NDOR1_HUMAN]	33402723	11484948	13183708	2,53
Q8IXT5	RNA-binding protein 12B OS=Homo sapiens GN=RBM12B PE=1 SV=2 - [RB12B_HUMAN]	19619288	17589896	7776232	2,52
P28290	Sperm-specific antigen 2 OS=Homo sapiens GN=SSFA2 PE=1 SV=3 - [SSFA2_HUMAN]	2271704	3162191	918250	2,47

Q15904	V-type proton ATPase subunit S1 OS=Homo sapiens GN=ATP6AP1 PE=1 SV=2 - [VAS1_HUMAN]	58898362	53520426	24568060	2,40
P04278	Sex hormone-binding globulin OS=Homo sapiens GN=SHBG PE=1 SV=2 - [SHBG_HUMAN]	8335991	7618348	3492956	2,39
Q9Y4F4	Protein FAM179B OS=Homo sapiens GN=FAM179B PE=1 SV=4 - [F179B_HUMAN]	1352790	2561655	577221	2,34
P35908	Keratin, type II cytoskeletal 2 epidermal OS=Homo sapiens GN=KRT2 PE=1 SV=2 - [K22E_HUMAN]	74189472	98798214	32102736	2,31
Q9NSY0	Nuclear receptor-binding protein 2 OS=Homo sapiens GN=NRBP2 PE=2 SV=2 - [NRBP2_HUMAN]	1159031	939506	513619	2,26
Q9HCE7	E3 ubiquitin-protein ligase SMURF1 OS=Homo sapiens GN=SMURF1 PE=1 SV=2 - [SMUF1_HUMAN]	33828231	36811710	15473308	2,19
P02649	Apolipoprotein E OS=Homo sapiens GN=APOE PE=1 SV=1 - [APOE_HUMAN]	104475654	99376722	48756502	2,14
Q9P2P6	StAR-related lipid transfer protein 9 OS=Homo sapiens GN=STARD9 PE=1 SV=3 - [STAR9_HUMAN]	2225654	3047024	1151132	1,93
P02766	Transthyretin OS=Homo sapiens GN=TTR PE=1 SV=1 - [TTHY_HUMAN]	403799280	306740800	219795326	1,84
P22352	Glutathione peroxidase 3 OS=Homo sapiens GN=GPX3 PE=1 SV=2 - [GPX3_HUMAN]	48637707	51872238	27560264	1,76
P17302	Gap junction alpha-1 protein OS=Homo sapiens GN=GJA1 PE=1 SV=2 - [CXA1_HUMAN]	2137551	1995230	1298599	1,65
Q24JP5	Transmembrane protein 132A OS=Homo sapiens GN=TMEM132A PE=1 SV=1 - [T132A_HUMAN]	5664553	7854394	7670946	0,74
P01859	Ig gamma-2 chain C region OS=Homo sapiens GN=IGHG2 PE=1 SV=2 - [IGHG2_HUMAN]	61518052	110977664	93466100	0,66
P00751	Complement factor B OS=Homo sapiens GN=CFB PE=1 SV=2 - [CFAB_HUMAN]	73334533	77634619	112032082	0,65
Q8TC12	Retinol dehydrogenase 11 OS=Homo sapiens GN=RDH11 PE=1 SV=2 - [RDH11_HUMAN]	3038926	3782868	4899282	0,62
Q14624	Inter-alpha-trypsin inhibitor heavy chain H4 OS=Homo sapiens GN=ITIH4 PE=1 SV=4 - [ITIH4_HUMAN]	9790530	7757702	16037370	0,61
Q9UHG2	ProSAAS OS=Homo sapiens GN=PCSK1N PE=1 SV=1 - [PCSK1_HUMAN]	4539273	5524971	7504598	0,60
P49589	Cysteine--tRNA ligase, cytoplasmic OS=Homo sapiens GN=CARS PE=1 SV=3 - [SYCC_HUMAN]	6180970	7396977	10268436	0,60
P13646	Keratin, type I cytoskeletal 13 OS=Homo sapiens GN=KRT13 PE=1 SV=4 - [K1C13_HUMAN]	198659239	268445336	330410367	0,60
Q6I9Y2	THO complex subunit 7 homolog OS=Homo sapiens GN=THOC7 PE=1 SV=3 - [THOC7_HUMAN]	22659150	26689996	38288944	0,59
Q15582	Transforming growth factor-beta-induced protein ig-h3 OS=Homo sapiens GN=TGFBI PE=1 SV=1 - [BGH3_HU	2416909	2376919	4255789	0,57
Q02386	Zinc finger protein 45 OS=Homo sapiens GN=ZNF45 PE=2 SV=2 - [ZNF45_HUMAN]	1041158	663040	1836787	0,57
Q8N945	PRELI domain-containing protein 2 OS=Homo sapiens GN=PRELID2 PE=2 SV=1 - [PRLD2_HUMAN]	92221901	41309964	163344177	0,56
Q8IWP9	Coiled-coil domain-containing protein 28A OS=Homo sapiens GN=CCDC28A PE=1 SV=1 - [CC28A_HUMAN]	10334179	2347563	19222375	0,54
A8MPX8	Protein phosphatase 2C-like domain-containing protein 1 OS=Homo sapiens GN=PP2D1 PE=2 SV=2 - [PP2D1	2989993	3446440	5669932	0,53
P01033	Metalloproteinase inhibitor 1 OS=Homo sapiens GN=TIMP1 PE=1 SV=1 - [TIMP1_HUMAN]	984406	642435	1868741	0,53
Q9Y2U5	Mitogen-activated protein kinase kinase kinase 2 OS=Homo sapiens GN=MAP3K2 PE=1 SV=2 - [M3K2_HUM.	169650379	198088037	322670625	0,53
P82987	ADAMTS-like protein 3 OS=Homo sapiens GN=ADAMTSL3 PE=1 SV=4 - [ATL3_HUMAN]	451653	432365	862354	0,52
Q9UJX3	Anaphase-promoting complex subunit 7 OS=Homo sapiens GN=ANAPC7 PE=1 SV=4 - [APC7_HUMAN]	1384986	1155703	2644451	0,52
Q9HC10	Otoferlin OS=Homo sapiens GN=OTOF PE=1 SV=3 - [OTOF_HUMAN]	128477849	187078386	247041238	0,52
Q9UPU5	Ubiquitin carboxyl-terminal hydrolase 24 OS=Homo sapiens GN=USP24 PE=1 SV=3 - [UBP24_HUMAN]	10617252	10552749	20461872	0,52
P01042	Kininogen-1 OS=Homo sapiens GN=KNG1 PE=1 SV=2 - [KNG1_HUMAN]	48817639	43589414	94797310	0,51
O43913	Origin recognition complex subunit 5 OS=Homo sapiens GN=ORC5 PE=1 SV=1 - [ORC5_HUMAN]	247199	499071	484558	0,51
Q15185	Prostaglandin E synthase 3 OS=Homo sapiens GN=PTGES3 PE=1 SV=1 - [TEBP_HUMAN]	1132285	1106913	2233891	0,51
Q2WGJ9	Fer-1-like protein 6 OS=Homo sapiens GN=FER1L6 PE=2 SV=2 - [FR1L6_HUMAN]	139366498	165153416	275816956	0,51
Q9ULE6	Paladin OS=Homo sapiens GN=PALD1 PE=1 SV=3 - [PALD_HUMAN]	14173269	19412820	28566870	0,50
Q01484	Ankyrin-2 OS=Homo sapiens GN=ANK2 PE=1 SV=4 - [ANK2_HUMAN]	159263502	138111323	324402896	0,49
Q92743	Serine protease HTRA1 OS=Homo sapiens GN=HTRA1 PE=1 SV=1 - [HTRA1_HUMAN]	584543	580916	1201225	0,49
P07360	Complement component C8 gamma chain OS=Homo sapiens GN=C8G PE=1 SV=3 - [CO8G_HUMAN]	1907874	2201776	3970766	0,48
P00747	Plasminogen OS=Homo sapiens GN=PLG PE=1 SV=2 - [PLMN_HUMAN]	53683767	64740691	113958036	0,47
P02679	Fibrinogen gamma chain OS=Homo sapiens GN=FGG PE=1 SV=3 - [FIBG_HUMAN]	19790041	11616118	42118814	0,47
Q494U1	Pleckstrin homology domain-containing family N member 1 OS=Homo sapiens GN=PLEKHN1 PE=1 SV=2 - [P	3988948	6785337	8751779	0,46
Q9BY67	Cell adhesion molecule 1 OS=Homo sapiens GN=CADM1 PE=1 SV=2 - [CADM1_HUMAN]	1729528	1964022	3978562	0,43
P02748	Complement component C9 OS=Homo sapiens GN=C9 PE=1 SV=2 - [CO9_HUMAN]	15127942	19035124	34989747	0,43
O95251	Histone acetyltransferase KAT7 OS=Homo sapiens GN=KAT7 PE=1 SV=1 - [KAT7_HUMAN]	4083350	3818117	9603053	0,43
P09486	SPARC OS=Homo sapiens GN=SPARC PE=1 SV=1 - [SPRC_HUMAN]	320296	172323	755598	0,42
Q96PY5	Formin-like protein 2 OS=Homo sapiens GN=FMNL2 PE=1 SV=3 - [FMNL2_HUMAN]	1752579	1173551	4181980	0,42
P02749	Beta-2-glycoprotein 1 OS=Homo sapiens GN=APOH PE=1 SV=3 - [APOH_HUMAN]	38509501	41906736	92297205	0,42
Q15746	Myosin light chain kinase, smooth muscle OS=Homo sapiens GN=MYLK PE=1 SV=4 - [MYLK_HUMAN]	447407	191986	1088989	0,41
P19652	Alpha-1-acid glycoprotein 2 OS=Homo sapiens GN=ORM2 PE=1 SV=2 - [A1AG2_HUMAN]	54880008	94337869	133762700	0,41
Q8N392	Rho GTPase-activating protein 18 OS=Homo sapiens GN=ARHGAP18 PE=1 SV=3 - [RHG18_HUMAN]	465234	420545	1135455	0,41
Q8IUR5	Transmembrane and TPR repeat-containing protein 1 OS=Homo sapiens GN=TMTC1 PE=1 SV=3 - [TMTC1_H	8932101	11568837	21872622	0,41
Q13630	GDP-L-fucose synthase OS=Homo sapiens GN=TSTA3 PE=1 SV=1 - [FCL_HUMAN]	1112686	1750635	2753773	0,40
Q96IY4	Carboxypeptidase B2 OS=Homo sapiens GN=CPB2 PE=1 SV=2 - [CBPB2_HUMAN]	511625	425822	1275283	0,40
Q9C0B1	Alpha-ketoglutarate-dependent dioxygenase FTO OS=Homo sapiens GN=FTO PE=1 SV=3 - [FTO_HUMAN]	5222953	4817730	13021370	0,40
P12035	Keratin, type II cytoskeletal 3 OS=Homo sapiens GN=KRT3 PE=1 SV=3 - [K2C3_HUMAN]	583711	919761	1464463	0,40
O15354	Prosaposin receptor GPR37 OS=Homo sapiens GN=GPR37 PE=1 SV=2 - [GPR37_HUMAN]	46875404	100084565	119182703	0,39
Q86UK0	ATP-binding cassette sub-family A member 12 OS=Homo sapiens GN=ABCA12 PE=1 SV=3 - [ABCAC_HUMAN	2070741	2021925	5355389	0,39
P02774	Vitamin D-binding protein OS=Homo sapiens GN=GC PE=1 SV=1 - [VTDB_HUMAN]	220045914	376548612	571288684	0,39
P00734	Prothrombin OS=Homo sapiens GN=F2 PE=1 SV=2 - [THRB_HUMAN]	24043988	32216375	63237379	0,38
P0C7V8	DDB1- and CUL4-associated factor 8-like protein 2 OS=Homo sapiens GN=DCAF8L2 PE=2 SV=2 - [DC8L2_HUI	2561309	1279511	6764568	0,38
Q7Z478	ATP-dependent RNA helicase DHX29 OS=Homo sapiens GN=DHX29 PE=1 SV=2 - [DHX29_HUMAN]	2445714	348618	6539846	0,37
Q2PPJ7	Ral GTPase-activating protein subunit alpha-2 OS=Homo sapiens GN=RALGAPA2 PE=1 SV=2 - [RGPA2_HUM/	1107763	1852063	3006316	0,37
Q8WWN8	Arf-GAP with Rho-GAP domain, ANK repeat and PH domain-containing protein 3 OS=Homo sapiens GN=AR/	4131239	5474109	11529953	0,36
Q13315	Serine-protein kinase ATM OS=Homo sapiens GN=ATM PE=1 SV=4 - [ATM_HUMAN]	9321298	17850356	26041955	0,36
P22792	Carboxypeptidase N subunit 2 OS=Homo sapiens GN=CPN2 PE=1 SV=3 - [CPN2_HUMAN]	245510	314947	686196	0,36
Q8IYT2	Cap-specific mRNA (nucleoside-2'-O-)-methyltransferase 2 OS=Homo sapiens GN=CMTR2 PE=1 SV=2 - [CMT	23142	46481	65243	0,35
P01876	Ig alpha-1 chain C region OS=Homo sapiens GN=IGHA1 PE=1 SV=2 - [IGHA1_HUMAN]	36542909	48083717	104616219	0,35
P25092	Heat-stable enterotoxin receptor OS=Homo sapiens GN=GUCY2C PE=1 SV=2 - [GUC2C_HUMAN]	16467404	29803585	47667557	0,35
P10643	Complement component C7 OS=Homo sapiens GN=C7 PE=1 SV=2 - [CO7_HUMAN]	1726957	1563257	5084386	0,34
Q8NI51	Transcriptional repressor CTCFL OS=Homo sapiens GN=CTCFL PE=1 SV=2 - [CTCFL_HUMAN]	2007176	4524294	5976859	0,34
P01877	Ig alpha-2 chain C region OS=Homo sapiens GN=IGHA2 PE=1 SV=3 - [IGHA2_HUMAN]	92959913	127376309	286502854	0,32
P35542	Serum amyloid A-4 protein OS=Homo sapiens GN=SAA4 PE=1 SV=2 - [SAA4_HUMAN]	64033	20599	197438	0,32
O43435	T-box transcription factor TBX1 OS=Homo sapiens GN=TBX1 PE=1 SV=1 - [TBX1_HUMAN]	547305	901663	1693157	0,32
Q5VZE5	N-alpha-acetyltransferase 35, NatC auxiliary subunit OS=Homo sapiens GN=NAA35 PE=1 SV=1 - [NAA35_HU	3133523	3261113	9695524	0,32
Q5T9S5	Coiled-coil domain-containing protein 18 OS=Homo sapiens GN=CCDC18 PE=2 SV=1 - [CCD18_HUMAN]	998772	729461	3099642	0,32
Q92541	RNA polymerase-associated protein RTF1 homolog OS=Homo sapiens GN=RTF1 PE=1 SV=4 - [RTF1_HUMAN	352848	294757	1095850	0,32
P05090	Apolipoprotein D OS=Homo sapiens GN=APOD PE=1 SV=1 - [APOD_HUMAN]	477131	561351	1482977	0,32
Q96N16	Janus kinase and microtubule-interacting protein 1 OS=Homo sapiens GN=JAKMIP1 PE=1 SV=1 - [JKIP1_HUN	267418	1793281	831787	0,32
Q96N87	Sodium-dependent neutral amino acid transporter B(0)AT3 OS=Homo sapiens GN=SLC6A18 PE=2 SV=2 - [S6	1280404	930736	4078457	0,31
P48681	Nestin OS=Homo sapiens GN=NES PE=1 SV=2 - [NEST_HUMAN]	4984952	13553178	15878663	0,31

Q13219	Pappalysin-1 OS=Homo sapiens GN=PAPPA PE=1 SV=3 - [PAPP1_HUMAN]	182998	111989	590291	0,31
O75882	Attractin OS=Homo sapiens GN=ATRN PE=1 SV=2 - [ATRN_HUMAN]	42756	50875	138604	0,31
P42262	Glutamate receptor 2 OS=Homo sapiens GN=GRIA2 PE=1 SV=3 - [GRIA2_HUMAN]	171661	117243	561970	0,31
P02751	Fibronectin OS=Homo sapiens GN=FN1 PE=1 SV=4 - [FINC_HUMAN]	36573293	65555579	119838606	0,31
P51451	Tyrosine-protein kinase Blk OS=Homo sapiens GN=BLK PE=1 SV=3 - [BLK_HUMAN]	2566147	5228898	8432298	0,30
Q9BY77	Polymerase delta-interacting protein 3 OS=Homo sapiens GN=POLDIP3 PE=1 SV=2 - [PDIP3_HUMAN]	16340	37309	53814	0,30
Q86VY4	Testis-specific Y-encoded-like protein 5 OS=Homo sapiens GN=TSPYL5 PE=1 SV=2 - [TSYL5_HUMAN]	393739	247345	1302462	0,30
Q9UGM5	Fetuin-B OS=Homo sapiens GN=FETUB PE=1 SV=2 - [FETUB_HUMAN]	347893	455885	1163765	0,30
Q03591	Complement factor H-related protein 1 OS=Homo sapiens GN=CFHR1 PE=1 SV=2 - [FHR1_HUMAN]	2621027	3045161	8952355	0,29
Q6ZTR5	Cilia- and flagella-associated protein 47 OS=Homo sapiens GN=CFAP47 PE=2 SV=4 - [CFA47_HUMAN]	328383	245609	1134461	0,29
Q9H2F5	Enhancer of polycomb homolog 1 OS=Homo sapiens GN=EPC1 PE=1 SV=1 - [EPC1_HUMAN]	612308	519246	2115838	0,29
P02671	Fibrinogen alpha chain OS=Homo sapiens GN=FGA PE=1 SV=2 - [FIBA_HUMAN]	4192344	3546865	14904251	0,28
P06865	Beta-hexosaminidase subunit alpha OS=Homo sapiens GN=HEXA PE=1 SV=2 - [HEXA_HUMAN]	47186269	47224227	169785781	0,28
P09488	Glutathione S-transferase Mu 1 OS=Homo sapiens GN=GSTM1 PE=1 SV=3 - [GSTM1_HUMAN]	508262	1334508	1834704	0,28
Q9UGJ0	5'-AMP-activated protein kinase subunit gamma-2 OS=Homo sapiens GN=PRKAG2 PE=1 SV=1 - [AAKG2_HUI	13243	2660	48010	0,28
Q9UPT9	Ubiquitin carboxyl-terminal hydrolase 22 OS=Homo sapiens GN=USP22 PE=1 SV=2 - [UBP22_HUMAN]	4506525	5063150	16386539	0,28
Q8TCU4	Alstrom syndrome protein 1 OS=Homo sapiens GN=ALMS1 PE=1 SV=3 - [ALMS1_HUMAN]	12140544	35070875	45510096	0,27
Q9H706	GRB2-associated and regulator of MAPK protein 1 OS=Homo sapiens GN=GAREM1 PE=1 SV=2 - [GARE1_HU	308120	115941	1174665	0,26
P61160	Actin-related protein 2 OS=Homo sapiens GN=ACTR2 PE=1 SV=1 - [ARP2_HUMAN]	27525386	33896755	105460593	0,26
P06312	Ig kappa chain V-IV region (Fragment) OS=Homo sapiens GN=IGKV4-1 PE=4 SV=1 - [KV401_HUMAN]	50974	67913	195550	0,26
P03952	Plasma kallikrein OS=Homo sapiens GN=KLKB1 PE=1 SV=1 - [KLKB1_HUMAN]	84732	73038	325372	0,26
Q9UF33	Ephrin type-A receptor 6 OS=Homo sapiens GN=EPHA6 PE=2 SV=3 - [EPHA6_HUMAN]	3673786	9009759	14200826	0,26
Q12841	Follistatin-related protein 1 OS=Homo sapiens GN=FSTL1 PE=1 SV=1 - [FSTL1_HUMAN]	26043744	51648101	100742484	0,26
Q15468	SCL-interrupting locus protein OS=Homo sapiens GN=STIL PE=1 SV=2 - [STIL_HUMAN]	265496	80136	1034868	0,26
P00740	Coagulation factor IX OS=Homo sapiens GN=F9 PE=1 SV=2 - [FA9_HUMAN]	67410	64567	264160	0,26
Q9NS87	Kinesin-like protein KIF15 OS=Homo sapiens GN=KIF15 PE=1 SV=1 - [KIF15_HUMAN]	2037984	2311712	8020411	0,25
P02760	Protein AMBP OS=Homo sapiens GN=AMBP PE=1 SV=1 - [AMBP_HUMAN]	6211489	9438425	24570836	0,25
A1L0T0	Acetolactate synthase-like protein OS=Homo sapiens GN=ILVBL PE=1 SV=2 - [ILVBL_HUMAN]	1050778	821188	4291438	0,24
Q8N1B4	Vacuolar protein sorting-associated protein 52 homolog OS=Homo sapiens GN=VPS52 PE=1 SV=1 - [VPS52_I	94101	54880	388732	0,24
Q8NEL0	Coiled-coil domain-containing protein 54 OS=Homo sapiens GN=CCDC54 PE=1 SV=2 - [CCD54_HUMAN]	1411242	1511339	5848602	0,24
Q8NCT3	Uncharacterized protein KIAA0895 OS=Homo sapiens GN=KIAA0895 PE=2 SV=4 - [K0895_HUMAN]	7451168	10143524	31499897	0,24
Q8TF05	Serine/threonine-protein phosphatase 4 regulatory subunit 1 OS=Homo sapiens GN=PPP4R1 PE=1 SV=1 - [P	178798	126967	777754	0,23
O60733	85/88 kDa calcium-independent phospholipase A2 OS=Homo sapiens GN=PLA2G6 PE=1 SV=2 - [PLPL9_HUM	44512	111940	194889	0,23
Q01469	Fatty acid-binding protein, epidermal OS=Homo sapiens GN=FABP5 PE=1 SV=3 - [FABP5_HUMAN]	13437	61854	60514	0,22
Q5T7N2	LINE-1 type transposase domain-containing protein 1 OS=Homo sapiens GN=L1TD1 PE=1 SV=1 - [LITD1_HUI	39201868	60430749	180830646	0,22
Q7Z7M9	Polypeptide N-acetylgalactosaminyltransferase 5 OS=Homo sapiens GN=GALNT5 PE=1 SV=1 - [GALT5_HUM/	10374624	21628170	48220448	0,22
P02675	Fibrinogen beta chain OS=Homo sapiens GN=FGB PE=1 SV=2 - [FIBB_HUMAN]	6745870	5032237	31952949	0,21
P01611	Ig kappa chain V-I region Wes OS=Homo sapiens PE=1 SV=1 - [KV119_HUMAN]	531439	1018899	2698117	0,20
Q15828	Cystatin-M OS=Homo sapiens GN=CST6 PE=1 SV=1 - [CYTM_HUMAN]	25458	11709	132349	0,19
Q8WZ42	Titin OS=Homo sapiens GN=TTN PE=1 SV=4 - [TITIN_HUMAN]	25038847	26923662	130465382	0,19
Q9BYJ0	Fibroblast growth factor-binding protein 2 OS=Homo sapiens GN=FGFBP2 PE=1 SV=1 - [FGFP2_HUMAN]	90311	135714	479261	0,19
P01764	Ig heavy chain V-III region 23 OS=Homo sapiens GN=IGHV3-23 PE=1 SV=2 - [HV303_HUMAN]	117424	214392	655346	0,18
Q15911	Zinc finger homeobox protein 3 OS=Homo sapiens GN=ZFHX3 PE=1 SV=2 - [ZFHX3_HUMAN]	59974	111970	335825	0,18
Q96N67	Dedicator of cytokinesis protein 7 OS=Homo sapiens GN=DOCK7 PE=1 SV=4 - [DOCK7_HUMAN]	953818	2757586	5499240	0,17
Q6PL18	ATPase family AAA domain-containing protein 2 OS=Homo sapiens GN=ATAD2 PE=1 SV=1 - [ATAD2_HUMAI	15997	33183	98194	0,16
O95995	Growth arrest-specific protein 8 OS=Homo sapiens GN=GAS8 PE=1 SV=1 - [GAS8_HUMAN]	20500	21551	126280	0,16
Q8WXI4	Acyl-coenzyme A thioesterase 11 OS=Homo sapiens GN=ACOT11 PE=1 SV=1 - [ACO11_HUMAN]	1194094	3251295	7656737	0,16
O75445	Usherin OS=Homo sapiens GN=USH2A PE=1 SV=3 - [USH2A_HUMAN]	310258	434342	2027622	0,15
O43174	Cytochrome P450 26A1 OS=Homo sapiens GN=CYP26A1 PE=2 SV=2 - [CP26A_HUMAN]	108011	272473	707278	0,15
O00512	B-cell CLL/lymphoma 9 protein OS=Homo sapiens GN=BCL9 PE=1 SV=4 - [BCL9_HUMAN]	64989	87279	428300	0,15
Q7RTW8	Otoancorin OS=Homo sapiens GN=OTOA PE=1 SV=1 - [OTOAN_HUMAN]	20364	7068	134465	0,15
P01344	Insulin-like growth factor II OS=Homo sapiens GN=IGF2 PE=1 SV=1 - [IGF2_HUMAN]	70840	90706	467774	0,15
Q9BYW2	Histone-lysine N-methyltransferase SETD2 OS=Homo sapiens GN=SETD2 PE=1 SV=3 - [SETD2_HUMAN]	2729824	6245095	20354142	0,13
Q9P2E2	Kinesin-like protein KIF17 OS=Homo sapiens GN=KIF17 PE=2 SV=3 - [KIF17_HUMAN]	47777	179214	358072	0,13
O14791	Apolipoprotein L1 OS=Homo sapiens GN=APOL1 PE=1 SV=5 - [APOL1_HUMAN]	28088	48969	213738	0,13
P36980	Complement factor H-related protein 2 OS=Homo sapiens GN=CFHR2 PE=1 SV=1 - [FHR2_HUMAN]	65499	97495	505118	0,13
Q8IY92	Structure-specific endonuclease subunit SLX4 OS=Homo sapiens GN=SLX4 PE=1 SV=3 - [SLX4_HUMAN]	569010	1028135	4684532	0,12
Q69YH5	Cell division cycle-associated protein 2 OS=Homo sapiens GN=CDCA2 PE=1 SV=2 - [CDCA2_HUMAN]	30573	2566	322195	0,09
O14628	Zinc finger protein 195 OS=Homo sapiens GN=ZNF195 PE=1 SV=2 - [ZN195_HUMAN]	8656923	7366716	92950626	0,09
Q8TDJ6	DmX-like protein 2 OS=Homo sapiens GN=DMXL2 PE=1 SV=2 - [DMXL2_HUMAN]	9725	21912	104835	0,09
Q68CZ2	Tensin-3 OS=Homo sapiens GN=TNS3 PE=1 SV=2 - [TENS3_HUMAN]	105557	157478	1169060	0,09
Q7LGC8	Carbohydrate sulfotransferase 3 OS=Homo sapiens GN=CHST3 PE=1 SV=3 - [CHST3_HUMAN]	93233	24369	1080296	0,09
Q96L03	Spermatogenesis-associated protein 17 OS=Homo sapiens GN=SPATA17 PE=2 SV=1 - [SPT17_HUMAN]	34409	379043	410723	0,08
P04070	Vitamin K-dependent protein C OS=Homo sapiens GN=PROC PE=1 SV=1 - [PROC_HUMAN]	14770	138577	204534	0,07
P0CG05	Ig lambda-2 chain C regions OS=Homo sapiens GN=IGLC2 PE=1 SV=1 - [LAC2_HUMAN]	30530	215635	455035	0,07
P24539	ATP synthase F(0) complex subunit B1, mitochondrial OS=Homo sapiens GN=ATP5F1 PE=1 SV=2 - [AT5F1_H	28313	16155	451024	0,06
Q9NYB0	Telomeric repeat-binding factor 2-interacting protein 1 OS=Homo sapiens GN=TERF2IP PE=1 SV=1 - [TE2IP_I	7819	2111	131412	0,06
Q8TF20	Zinc finger protein 721 OS=Homo sapiens GN=ZNF721 PE=2 SV=2 - [ZN721_HUMAN]	94576	118035	1642880	0,06
Q86Z14	Beta-klotho OS=Homo sapiens GN=KLB PE=1 SV=1 - [KLOTB_HUMAN]	661	297	12263	0,05
P61812	Transforming growth factor beta-2 OS=Homo sapiens GN=TGFB2 PE=1 SV=1 - [TGFB2_HUMAN]	1110	547	21289	0,05
Q96MT1	RING finger protein 145 OS=Homo sapiens GN=RNF145 PE=2 SV=2 - [RN145_HUMAN]	24811	31488	516777	0,05
Q9H5N1	Rab GTPase-binding effector protein 2 OS=Homo sapiens GN=RABEP2 PE=1 SV=2 - [RABE2_HUMAN]	15247	13450	318079	0,05
Q9UPS8	Ankyrin repeat domain-containing protein 26 OS=Homo sapiens GN=ANKRD26 PE=1 SV=3 - [ANR26_HUMAI	1308605	2706651	28934735	0,05
Q14699	Raftlin OS=Homo sapiens GN=RFTN1 PE=1 SV=4 - [RFTN1_HUMAN]	10134	28148	237319	0,04
Q9NZJ4	Sacsin OS=Homo sapiens GN=SACS PE=1 SV=2 - [SACS_HUMAN]	7514	21747	184784	0,04
Q9UKN7	Unconventional myosin-XV OS=Homo sapiens GN=MYO15A PE=1 SV=2 - [MYO15_HUMAN]	127664	432033	3889663	0,03
P62873	Guanine nucleotide-binding protein G(I)/G(S)/G(T) subunit beta-1 OS=Homo sapiens GN=GNB1 PE=1 SV=3 -	2581	667	86134	0,03
P69905	Hemoglobin subunit alpha OS=Homo sapiens GN=HBA1 PE=1 SV=2 - [HBA_HUMAN]	1216046	376605	42122572	0,03
P68871	Hemoglobin subunit beta OS=Homo sapiens GN=HBB PE=1 SV=2 - [HBB_HUMAN]	4893086	3601152	188088670	0,03
Q96N64	PWWP domain-containing protein 2A OS=Homo sapiens GN=PWWP2A PE=1 SV=2 - [PWP2A_HUMAN]	49212	20414	2231265	0,02
O15455	Toll-like receptor 3 OS=Homo sapiens GN=TLR3 PE=1 SV=1 - [TLR3_HUMAN]	9573	3416	455866	0,02

Q8NF50	Dedicator of cytokinesis protein 8 OS=Homo sapiens GN=DOCK8 PE=1 SV=3 - [DOCK8_HUMAN]	9504	34517	522527	0,02
Q9Y4F3	Meiosis arrest female protein 1 OS=Homo sapiens GN=KIAA0430 PE=1 SV=6 - [MARF1_HUMAN]	2408	12260	147521	0,02
P10523	S-arrestin OS=Homo sapiens GN=SAG PE=1 SV=3 - [ARRS_HUMAN]	754	1242	62103	0,01
P20396	Pro-thyrotropin-releasing hormone OS=Homo sapiens GN=TRH PE=1 SV=1 - [TRH_HUMAN]	2459	12297	272381	0,01
