# Supplementary material for: Systems pathology analysis identifies neurodegenerative nature of age‐related vitreoretinal interface diseases
Source: Aging Cell. 2018 Jul 2;17(5):e12809. doi: 10.1111/acel.12809 (PMC6156470; doi:10.1111/acel.12809)
Supplement: Supplementary file 9 [file ACEL-17-e12809-s009.pdf]

Supplementary Table S6: Significantly differed proteins between MH and DME proteomes, q-value < 0.05.
174 proteins were present at higher level (red) and 123 proteins at lower level (yellow) in the MH proteome when compared to DME proteome, the abundance ratio >2.

		Average of MS1 Intensities			Fold differences
**Accession**	**Description**	**iERM**	**MH**	**DME**	**MH vs DME**
Q9Y646	Carboxypeptidase Q OS=Homo sapiens GN=CPQ PE=1 SV=1 - [CBPQ_HUMAN]	37151	28393	0	#DIV/0!
Q15173	Serine/threonine-protein phosphatase 2A 56 kDa regulatory subunit beta isoform OS=Homo sapiens GN=PPP2R5B	14944	48695	0	#DIV/0!
Q59EK9	RUN domain-containing protein 3A OS=Homo sapiens GN=RUNDC3A PE=1 SV=2 - [RUN3A_HUMAN]	69474	114872	0	#DIV/0!
O75674	TOM1-like protein 1 OS=Homo sapiens GN=TOM1L1 PE=1 SV=2 - [TM1L1_HUMAN]	90054	78317	0	#DIV/0!
Q9NPY3	Complement component C1q receptor OS=Homo sapiens GN=CD93 PE=1 SV=3 - [C1QR1_HUMAN]	29545	16030	0	#DIV/0!
O14686	Histone-lysine N-methyltransferase 2D OS=Homo sapiens GN=KMT2D PE=1 SV=2 - [KMT2D_HUMAN]	72642	143334	0	#DIV/0!
P0CG00	Putative zinc finger and SCAN domain-containing protein 5D OS=Homo sapiens GN=ZSCAN5D PE=5 SV=1 - [ZSA5D_	31003	34909	0	#DIV/0!
Q9BQQ7	Receptor-transporting protein 3 OS=Homo sapiens GN=RTP3 PE=1 SV=1 - [RTP3_HUMAN]	427654	1136483	854	1331,28
O95747	Serine/threonine-protein kinase OSR1 OS=Homo sapiens GN=OXSR1 PE=1 SV=1 - [OXSR1_HUMAN]	354896	209719	196	1070,49
P51689	Arylsulfatase D OS=Homo sapiens GN=ARSD PE=1 SV=2 - [ARSD_HUMAN]	271807	392868	581	676,12
O43157	Plexin-B1 OS=Homo sapiens GN=PLXNB1 PE=1 SV=3 - [PLXB1_HUMAN]	24177	42610	106	401,21
Q14571	Inositol 1,4,5-trisphosphate receptor type 2 OS=Homo sapiens GN=ITPR2 PE=1 SV=2 - [ITPR2_HUMAN]	134674	176048	550	320,19
Q9P0W8	Spermatogenesis-associated protein 7 OS=Homo sapiens GN=SPATA7 PE=1 SV=3 - [SPAT7_HUMAN]	146190	225253	818	275,44
Q14563	Semaphorin-3A OS=Homo sapiens GN=SEMA3A PE=1 SV=1 - [SEM3A_HUMAN]	10131	17923	65	274,17
P80748	Ig lambda chain V-III region LOI OS=Homo sapiens PE=1 SV=1 - [LV302_HUMAN]	158713	353049	1867	189,12
Q9BXJ4	Complement C1q tumor necrosis factor-related protein 3 OS=Homo sapiens GN=C1QTNF3 PE=1 SV=1 - [C1QT3_HU	242404	219225	1291	169,83
Q9UQB3	Catenin delta-2 OS=Homo sapiens GN=CTNND2 PE=1 SV=3 - [CTND2_HUMAN]	5689028	6086147	41622	146,22
Q9NVU7	Protein SDA1 homolog OS=Homo sapiens GN=SDAD1 PE=1 SV=3 - [SDA1_HUMAN]	1208500	2403723	21067	114,10
Q9UBN7	Histone deacetylase 6 OS=Homo sapiens GN=HDAC6 PE=1 SV=2 - [HDAC6_HUMAN]	1339885	3450512	32645	105,70
Q8TAG5	V-set and transmembrane domain-containing protein 2A OS=Homo sapiens GN=VSTM2A PE=2 SV=3 - [VTM2A_HU	80782	82614	822	100,52
Q9H4D0	Calsyntenin-2 OS=Homo sapiens GN=CLSTN2 PE=1 SV=2 - [CSTN2_HUMAN]	34358	24551	283	86,87
Q9Y287	Integral membrane protein 2B OS=Homo sapiens GN=ITM2B PE=1 SV=1 - [ITM2B_HUMAN]	1728182	1648716	22315	73,88
Q00056	Homeobox protein Hox-A4 OS=Homo sapiens GN=HOXA4 PE=2 SV=3 - [HXA4_HUMAN]	93728	189509	2707	69,99
Q6UWY2	Serine protease 57 OS=Homo sapiens GN=PRSS57 PE=1 SV=2 - [PRS57_HUMAN]	123189	248593	3722	66,79
Q99519	Sialidase-1 OS=Homo sapiens GN=NEU1 PE=1 SV=1 - [NEUR1_HUMAN]	303651	513079	8708	58,92
Q86SE9	Polycomb group RING finger protein 5 OS=Homo sapiens GN=PCGF5 PE=1 SV=1 - [PCGF5_HUMAN]	96409	58146	1132	51,37
Q9UHP3	Ubiquitin carboxyl-terminal hydrolase 25 OS=Homo sapiens GN=USP25 PE=1 SV=4 - [UBP25_HUMAN]	424002	495239	10108	48,99
Q86SQ0	Pleckstrin homology-like domain family B member 2 OS=Homo sapiens GN=PHLDB2 PE=1 SV=2 - [PHLB2_HUMAN]	554741	579357	12212	47,44
Q9UPS6	Histone-lysine N-methyltransferase SETD1B OS=Homo sapiens GN=SETD1B PE=1 SV=3 - [SET1B_HUMAN]	466084	735319	16659	44,14
P41271	Neuroblastoma suppressor of tumorigenicity 1 OS=Homo sapiens GN=NBL1 PE=1 SV=2 - [NBL1_HUMAN]	170603	234399	5694	41,17
Q14152	Eukaryotic translation initiation factor 3 subunit A OS=Homo sapiens GN=EIF3A PE=1 SV=1 - [EIF3A_HUMAN]	150943	205299	5245	39,14
Q96DT5	Dynein heavy chain 11, axonemal OS=Homo sapiens GN=DNAH11 PE=1 SV=4 - [DYH11_HUMAN]	6820149	10184163	268176	37,98
O60287	Nucleolar pre-ribosomal-associated protein 1 OS=Homo sapiens GN=URB1 PE=1 SV=4 - [NPA1P_HUMAN]	51385	120378	3629	33,17
P06737	Glycogen phosphorylase, liver form OS=Homo sapiens GN=PYGL PE=1 SV=4 - [PYGL_HUMAN]	1458662	2623132	79771	32,88
P55289	Cadherin-12 OS=Homo sapiens GN=CDH12 PE=2 SV=2 - [CAD12_HUMAN]	3572765	4479107	139150	32,19
Q63HQ2	Pikachurin OS=Homo sapiens GN=EGFLAM PE=1 SV=2 - [EGFLA_HUMAN]	43873	47673	1522	31,32
P35222	Catenin beta-1 OS=Homo sapiens GN=CTNNB1 PE=1 SV=1 - [CTNB1_HUMAN]	2039975	3899096	129234	30,17
Q13214	Semaphorin-3B OS=Homo sapiens GN=SEMA3B PE=2 SV=1 - [SEM3B_HUMAN]	16810	18084	609	29,69
P48552	Nuclear receptor-interacting protein 1 OS=Homo sapiens GN=NRIP1 PE=1 SV=2 - [NRIP1_HUMAN]	64019	97081	3317	29,27
P58107	Epiplakin OS=Homo sapiens GN=EPPK1 PE=1 SV=2 - [EPIPL_HUMAN]	18463802	20499266	778990	26,32
Q86UX2	Inter-alpha-trypsin inhibitor heavy chain H5 OS=Homo sapiens GN=ITIH5 PE=2 SV=2 - [ITIH5_HUMAN]	121068	136493	6166	22,14
Q14995	Nuclear receptor subfamily 1 group D member 2 OS=Homo sapiens GN=NR1D2 PE=1 SV=3 - [NR1D2_HUMAN]	14192048	14080864	637060	22,10
Q15418	Ribosomal protein S6 kinase alpha-1 OS=Homo sapiens GN=RPS6KA1 PE=1 SV=2 - [KS6A1_HUMAN]	78142	118762	5439	21,83
Q02817	Mucin-2 OS=Homo sapiens GN=MUC2 PE=1 SV=2 - [MUC2_HUMAN]	53439520	12623677	596829	21,15
O43306	Adenylate cyclase type 6 OS=Homo sapiens GN=ADCY6 PE=1 SV=2 - [ADCY6_HUMAN]	213099	1136476	61303	18,54
P05408	Neuroendocrine protein 7B2 OS=Homo sapiens GN=SCG5 PE=1 SV=2 - [7B2_HUMAN]	972110	1069617	57758	18,52
P42356	Phosphatidylinositol 4-kinase alpha OS=Homo sapiens GN=PI4KA PE=1 SV=4 - [PI4KA_HUMAN]	672176	2723851	150921	18,05
Q15818	Neuronal pentraxin-1 OS=Homo sapiens GN=NPTX1 PE=2 SV=2 - [NPTX1_HUMAN]	459172	858915	48040	17,88
Q9BXS4	Transmembrane protein 59 OS=Homo sapiens GN=TMEM59 PE=1 SV=1 - [TMM59_HUMAN]	147228	292202	16556	17,65
Q8NE71	ATP-binding cassette sub-family F member 1 OS=Homo sapiens GN=ABCF1 PE=1 SV=2 - [ABCF1_HUMAN]	5873295	13299858	842580	15,78
P08294	Extracellular superoxide dismutase [Cu-Zn] OS=Homo sapiens GN=SOD3 PE=1 SV=2 - [SODE_HUMAN]	745189	877340	55691	15,75
Q7Z5M8	Protein ABHD12B OS=Homo sapiens GN=ABHD12B PE=2 SV=1 - [AB12B_HUMAN]	1153211	1551678	101815	15,24
Q9HB19	Pleckstrin homology domain-containing family A member 2 OS=Homo sapiens GN=PLEKHA2 PE=1 SV=2 - [PKHA2_H	1529527	2231554	150317	14,85
P49750	YLP motif-containing protein 1 OS=Homo sapiens GN=YLPM1 PE=1 SV=3 - [YLPM1_HUMAN]	60900	168630	11425	14,76
P04114	Apolipoprotein B-100 OS=Homo sapiens GN=APOB PE=1 SV=2 - [APOB_HUMAN]	471442	711266	51953	13,69
Q9P219	Protein Daple OS=Homo sapiens GN=CCDC88C PE=1 SV=3 - [DAPLE_HUMAN]	22332321	29192412	2159148	13,52
P02549	Spectrin alpha chain, erythrocytic 1 OS=Homo sapiens GN=SPTA1 PE=1 SV=5 - [SPTA1_HUMAN]	14965135	44265807	3317173	13,34
Q14533	Keratin, type II cuticular Hb1 OS=Homo sapiens GN=KRT81 PE=1 SV=3 - [KRT81_HUMAN]	140213	112520	8575	13,12
O94880	PHD finger protein 14 OS=Homo sapiens GN=PHF14 PE=1 SV=2 - [PHF14_HUMAN]	10644581	14864652	1145820	12,97
O14594	Neurocan core protein OS=Homo sapiens GN=NCAN PE=1 SV=3 - [NCAN_HUMAN]	1342405	1926666	155046	12,43
Q86SQ4	G-protein coupled receptor 126 OS=Homo sapiens GN=GPR126 PE=1 SV=3 - [GP126_HUMAN]	173406	91246	7430	12,28
P26006	Integrin alpha-3 OS=Homo sapiens GN=ITGA3 PE=1 SV=5 - [ITA3_HUMAN]	617077	220599	18529	11,91
Q8WVM8	Sec1 family domain-containing protein 1 OS=Homo sapiens GN=SCFD1 PE=1 SV=4 - [SCFD1_HUMAN]	3281523	621465	53311	11,66
Q8WXQ3	Putative uncharacterized protein encoded by LINC01599 OS=Homo sapiens GN=LINC01599 PE=2 SV=1 - [CN183_HU	325811	922170	84805	10,87
Q9NZP8	Complement C1r subcomponent-like protein OS=Homo sapiens GN=C1RL PE=1 SV=2 - [C1RL_HUMAN]	65413	26464	2635	10,04
Q6PGQ7	Protein aurora borealis OS=Homo sapiens GN=BORA PE=1 SV=2 - [BORA_HUMAN]	146280	362305	36415	9,95
P98164	Low-density lipoprotein receptor-related protein 2 OS=Homo sapiens GN=LRP2 PE=1 SV=3 - [LRP2_HUMAN]	598869	651585	67478	9,66
O15240	Neurosecretory protein VGF OS=Homo sapiens GN=VGF PE=1 SV=2 - [VGF_HUMAN]	1434844	1291751	135982	9,50
Q9ULB1	Neurexin-1 OS=Homo sapiens GN=NRXN1 PE=2 SV=1 - [NRX1A_HUMAN]	62510	55919	6112	9,15
A6NFK2	Glutaredoxin domain-containing cysteine-rich protein 2 OS=Homo sapiens GN=GRXCR2 PE=3 SV=1 - [GRCR2_HUM	5769930	6080115	664832	9,15
Q6Q759	Sperm-associated antigen 17 OS=Homo sapiens GN=SPAG17 PE=2 SV=1 - [SPG17_HUMAN]	599958	675633	74195	9,11
Q9NPR2	Semaphorin-4B OS=Homo sapiens GN=SEMA4B PE=1 SV=3 - [SEM4B_HUMAN]	372742	295423	32785	9,01
Q8WXD2	Secretogranin-3 OS=Homo sapiens GN=SCG3 PE=1 SV=3 - [SCG3_HUMAN]	7181171	9745678	1170881	8,32
Q96ME7	Zinc finger protein 512 OS=Homo sapiens GN=ZNF512 PE=1 SV=2 - [ZN512_HUMAN]	2281909	6822903	853431	7,99
O00391	Sulfhydryl oxidase 1 OS=Homo sapiens GN=QSOX1 PE=1 SV=3 - [QSOX1_HUMAN]	316654	369401	46300	7,98
Q9NNX1	Tuftelin OS=Homo sapiens GN=TUFT1 PE=1 SV=1 - [TUFT1_HUMAN]	486602980	33160534	4265973	7,77
Q96MR6	Cilia- and flagella-associated protein 57 OS=Homo sapiens GN=CFAP57 PE=2 SV=3 - [CFA57_HUMAN]	2179435	4867951	633691	7,68
Q9UBX1	Cathepsin F OS=Homo sapiens GN=CTSF PE=1 SV=1 - [CATF_HUMAN]	11900122	31276874	4578743	6,83

Q5T5U3	Rho GTPase-activating protein 21 OS=Homo sapiens GN=ARHGAP21 PE=1 SV=1 - [RHG21_HUMAN]	14913218	15869361	2348797	6,76
Q14679	Tubulin polyglutamylase TTLL4 OS=Homo sapiens GN=TTLL4 PE=1 SV=2 - [TTLL4_HUMAN]	2579064	2270408	340498	6,67
O00533	Neural cell adhesion molecule L1-like protein OS=Homo sapiens GN=CHL1 PE=1 SV=4 - [NCHL1_HUMAN]	690221	520523	79695	6,53
P22304	Iduronate 2-sulfatase OS=Homo sapiens GN=IDS PE=1 SV=1 - [IDS_HUMAN]	276940	340803	52376	6,51
Q7Z7G0	Target of Nesh-SH3 OS=Homo sapiens GN=ABI3BP PE=1 SV=1 - [TARSH_HUMAN]	8434473	11666609	1831771	6,37
Q12797	Aspartyl/asparaginyl beta-hydroxylase OS=Homo sapiens GN=ASPH PE=1 SV=3 - [ASPH_HUMAN]	169980	144590	23057	6,27
Q7L0Y3	Mitochondrial ribonuclease P protein 1 OS=Homo sapiens GN=TRMT10C PE=1 SV=2 - [MRRP1_HUMAN]	357784	309701	49483	6,26
O60313	Dynamin-like 120 kDa protein, mitochondrial OS=Homo sapiens GN=OPA1 PE=1 SV=3 - [OPA1_HUMAN]	3401948	3313066	530660	6,24
Q16769	Glutaminyl-peptide cyclotransferase OS=Homo sapiens GN=QPCT PE=1 SV=1 - [QPCT_HUMAN]	718616	842885	135722	6,21
P10645	Chromogranin-A OS=Homo sapiens GN=CHGA PE=1 SV=7 - [CMGA_HUMAN]	1530969	1636717	271500	6,03
O00763	Acetyl-CoA carboxylase 2 OS=Homo sapiens GN=ACACB PE=1 SV=3 - [ACACB_HUMAN]	1437135	949651	157553	6,03
O75063	Glycosaminoglycan xylosylkinase OS=Homo sapiens GN=FAM20B PE=1 SV=1 - [XYLK_HUMAN]	854683	290223	48227	6,02
P13611	Versican core protein OS=Homo sapiens GN=VCAN PE=1 SV=3 - [CSPG2_HUMAN]	11149234	13545022	2278223	5,95
O00115	Deoxyribonuclease-2-alpha OS=Homo sapiens GN=DNASE2 PE=1 SV=2 - [DNS2A_HUMAN]	181359	98509	16641	5,92
Q9Y5E7	Protocadherin beta-2 OS=Homo sapiens GN=PCDHB2 PE=1 SV=1 - [PCDB2_HUMAN]	2744221	7368655	1249171	5,90
P20929	Nebulin OS=Homo sapiens GN=NEB PE=1 SV=5 - [NEBU_HUMAN]	35937405	12357649	2104757	5,87
Q99574	Neuroserpin OS=Homo sapiens GN=SERPINI1 PE=1 SV=1 - [NEUS_HUMAN]	544657	517989	88590	5,85
O75916	Regulator of G-protein signaling 9 OS=Homo sapiens GN=RGS9 PE=1 SV=1 - [RGS9_HUMAN]	10442534	23055310	3994036	5,77
Q2NKX8	DNA excision repair protein ERCC-6-like OS=Homo sapiens GN=ERCC6L PE=1 SV=1 - [ERC6L_HUMAN]	56117	174657	31338	5,57
Q6IB77	Glycine N-acyltransferase OS=Homo sapiens GN=GLYAT PE=1 SV=3 - [GLYAT_HUMAN]	1240980	1561830	281035	5,56
Q8TBE7	Solute carrier family 35 member G2 OS=Homo sapiens GN=SLC35G2 PE=1 SV=3 - [S35G2_HUMAN]	43520	56416	10749	5,25
Q9H3G5	Probable serine carboxypeptidase CPVL OS=Homo sapiens GN=CPVL PE=1 SV=2 - [CPVL_HUMAN]	1781747	2912808	582089	5,00
Q9UBZ9	DNA repair protein REV1 OS=Homo sapiens GN=REV1 PE=1 SV=1 - [REV1_HUMAN]	714503	724574	145228	4,99
Q92520	Protein FAM3C OS=Homo sapiens GN=FAM3C PE=1 SV=1 - [FAM3C_HUMAN]	2614736	2036908	426201	4,78
Q99618	Cell division cycle-associated protein 3 OS=Homo sapiens GN=CDCA3 PE=1 SV=1 - [CDCA3_HUMAN]	85833	202271	42440	4,77
Q9H1K4	Mitochondrial glutamate carrier 2 OS=Homo sapiens GN=SLC25A18 PE=1 SV=1 - [GHC2_HUMAN]	19722057	26976893	5780657	4,67
P30291	Wee1-like protein kinase OS=Homo sapiens GN=WEE1 PE=1 SV=2 - [WEE1_HUMAN]	580708	640030	139693	4,58
P12259	Coagulation factor V OS=Homo sapiens GN=F5 PE=1 SV=4 - [FA5_HUMAN]	431791	494672	109523	4,52
Q12860	Contactin-1 OS=Homo sapiens GN=CNTN1 PE=1 SV=1 - [CNTN1_HUMAN]	945733	906945	201906	4,49
Q9Y4F4	Protein FAM179B OS=Homo sapiens GN=FAM179B PE=1 SV=4 - [F179B_HUMAN]	1352790	2561655	577221	4,44
Q9HBV2	Sperm acrosome membrane-associated protein 1 OS=Homo sapiens GN=SPACA1 PE=1 SV=1 - [SACA1_HUMAN]	511559	641452	149297	4,30
Q96HE7	ERO1-like protein alpha OS=Homo sapiens GN=ERO1A PE=1 SV=2 - [ERO1A_HUMAN]	3003385	3396555	794614	4,27
Q13948	Protein CASP OS=Homo sapiens GN=CUX1 PE=1 SV=2 - [CASP_HUMAN]	559075	1573362	377103	4,17
P13987	CD59 glycoprotein OS=Homo sapiens GN=CD59 PE=1 SV=1 - [CD59_HUMAN]	439770	849960	213556	3,98
Q9P2M7	Cingulin OS=Homo sapiens GN=CGN PE=1 SV=2 - [CING_HUMAN]	1147424	2099882	529615	3,96
Q9HAU0	Pleckstrin homology domain-containing family A member 5 OS=Homo sapiens GN=PLEKHA5 PE=1 SV=1 - [PKHA5_H	230005	286413	73049	3,92
P13645	Keratin, type I cytoskeletal 10 OS=Homo sapiens GN=KRT10 PE=1 SV=6 - [K1C10_HUMAN]	91617695	127100527	32840928	3,87
Q92823	Neuronal cell adhesion molecule OS=Homo sapiens GN=NRCAM PE=1 SV=3 - [NRCAM_HUMAN]	3324184	2524496	653637	3,86
P16519	Neuroendocrine convertase 2 OS=Homo sapiens GN=PCSK2 PE=2 SV=2 - [NEC2_HUMAN]	617401	503946	131950	3,82
Q86T23	Putative ciliary rootlet coiled-coil protein-like 1 protein OS=Homo sapiens GN=CROCCP2 PE=5 SV=1 - [CROL1_HUM	433563	289734	76191	3,80
O75787	Renin receptor OS=Homo sapiens GN=ATP6AP2 PE=1 SV=2 - [RENR_HUMAN]	880537	992340	262531	3,78
Q96S96	Phosphatidylethanolamine-binding protein 4 OS=Homo sapiens GN=PEBP4 PE=1 SV=3 - [PEBP4_HUMAN]	2458910	3001503	813290	3,69
P05154	Plasma serine protease inhibitor OS=Homo sapiens GN=SERPINA5 PE=1 SV=3 - [IPSP_HUMAN]	335151	667023	181852	3,67
Q53EL9	Seizure protein 6 homolog OS=Homo sapiens GN=SEZ6 PE=1 SV=2 - [SEZ6_HUMAN]	1882071	1549716	442665	3,50
P28290	Sperm-specific antigen 2 OS=Homo sapiens GN=SSFA2 PE=1 SV=3 - [SSFA2_HUMAN]	2271704	3162191	918250	3,44
Q16706	Alpha-mannosidase 2 OS=Homo sapiens GN=MAN2A1 PE=1 SV=2 - [MA2A1_HUMAN]	5127108	5363524	1563133	3,43
Q7Z2W4	Zinc finger CCCH-type antiviral protein 1 OS=Homo sapiens GN=ZC3HAV1 PE=1 SV=3 - [ZCCHV_HUMAN]	893640	1385891	405932	3,41
P51693	Amyloid-like protein 1 OS=Homo sapiens GN=APLP1 PE=1 SV=3 - [APLP1_HUMAN]	6082322	5302804	1574277	3,37
Q9Y5W5	Wnt inhibitory factor 1 OS=Homo sapiens GN=WIF1 PE=1 SV=3 - [WIF1_HUMAN]	32093829	40875035	12215893	3,35
Q9BSG5	Retbindin OS=Homo sapiens GN=RTBDN PE=2 SV=2 - [RTBDN_HUMAN]	4419329	4012345	1203110	3,33
O96028	Histone-lysine N-methyltransferase NSD2 OS=Homo sapiens GN=WHSC1 PE=1 SV=1 - [NSD2_HUMAN]	590717	991269	302018	3,28
O75113	NEDD4-binding protein 1 OS=Homo sapiens GN=N4BP1 PE=1 SV=4 - [N4BP1_HUMAN]	7826556	11067299	3402386	3,25
P32019	Type II inositol 1,4,5-trisphosphate 5-phosphatase OS=Homo sapiens GN=INPP5B PE=1 SV=4 - [I5P2_HUMAN]	2745187	2800737	862724	3,25
Q8IVF6	Ankyrin repeat domain-containing protein 18A OS=Homo sapiens GN=ANKRD18A PE=2 SV=3 - [AN18A_HUMAN]	331102	308070	95467	3,23
P53004	Biliverdin reductase A OS=Homo sapiens GN=BLVRA PE=1 SV=2 - [BIEA_HUMAN]	3560070	1085335	337316	3,22
Q5T6C5	Ataxin-7-like protein 2 OS=Homo sapiens GN=ATXN7L2 PE=3 SV=1 - [AT7L2_HUMAN]	14963295	41385178	12919801	3,20
P43251	Biotinidase OS=Homo sapiens GN=BTD PE=1 SV=2 - [BTD_HUMAN]	8615634	8601553	2706339	3,18
Q9H8L6	Multimerin-2 OS=Homo sapiens GN=MMRN2 PE=1 SV=2 - [MMRN2_HUMAN]	363319	1384464	436302	3,17
P08779	Keratin, type I cytoskeletal 16 OS=Homo sapiens GN=KRT16 PE=1 SV=4 - [K1C16_HUMAN]	6101443	7318798	2314097	3,16
P46108	Adapter molecule crk OS=Homo sapiens GN=CRK PE=1 SV=2 - [CRK_HUMAN]	4579245	4919238	1577169	3,12
Q86UN2	Reticulon-4 receptor-like 1 OS=Homo sapiens GN=RTN4RL1 PE=1 SV=1 - [R4RL1_HUMAN]	36017563	119876831	38581223	3,11
P35908	Keratin, type II cytoskeletal 2 epidermal OS=Homo sapiens GN=KRT2 PE=1 SV=2 - [K22E_HUMAN]	74189472	98798214	32102736	3,08
Q86YA3	Protein ZGRF1 OS=Homo sapiens GN=ZGRF1 PE=2 SV=3 - [ZGRF1_HUMAN]	177060	150751	49012	3,08
Q9H939	Proline-serine-threonine phosphatase-interacting protein 2 OS=Homo sapiens GN=PSTPIP2 PE=1 SV=4 - [PPIP2_HU	250094	274472	89461	3,07
Q6ZMK1	Cysteine and histidine-rich protein 1 OS=Homo sapiens GN=CYHR1 PE=1 SV=2 - [CYHR1_HUMAN]	245252	226253	73942	3,06
Q6UX71	Plexin domain-containing protein 2 OS=Homo sapiens GN=PLXDC2 PE=1 SV=1 - [PXDC2_HUMAN]	507987	503540	165857	3,04
Q86WZ6	Zinc finger protein 227 OS=Homo sapiens GN=ZNF227 PE=1 SV=1 - [ZN227_HUMAN]	2113675	5902922	1977203	2,99
Q06481	Amyloid-like protein 2 OS=Homo sapiens GN=APLP2 PE=1 SV=2 - [APLP2_HUMAN]	40815419	41861022	14213584	2,95
Q9UHB6	LIM domain and actin-binding protein 1 OS=Homo sapiens GN=LIMA1 PE=1 SV=1 - [LIMA1_HUMAN]	2268924	673378	228998	2,94
Q8TDB6	E3 ubiquitin-protein ligase DTX3L OS=Homo sapiens GN=DTX3L PE=1 SV=1 - [DTX3L_HUMAN]	17005	53022	18135	2,92
Q9Y4I1	Unconventional myosin-Va OS=Homo sapiens GN=MYO5A PE=1 SV=2 - [MYO5A_HUMAN]	314925	1093701	382147	2,86
O94985	Calsyntenin-1 OS=Homo sapiens GN=CLSTN1 PE=1 SV=1 - [CSTN1_HUMAN]	64267366	63911758	22608562	2,83
Q8IXJ6	NAD-dependent protein deacetylase sirtuin-2 OS=Homo sapiens GN=SIRT2 PE=1 SV=2 - [SIR2_HUMAN]	743588	947158	338425	2,80
P16870	Carboxypeptidase E OS=Homo sapiens GN=CPE PE=1 SV=1 - [CBPE_HUMAN]	20748199	19624878	7036285	2,79
Q96NL6	Sodium channel and clathrin linker 1 OS=Homo sapiens GN=SCLT1 PE=1 SV=2 - [SCLT1_HUMAN]	3721772	9142708	3299192	2,77
O14773	Tripeptidyl-peptidase 1 OS=Homo sapiens GN=TPP1 PE=1 SV=2 - [TPP1_HUMAN]	9511448	9140646	3399327	2,69
Q9UBM8	Alpha-1,3-mannosyl-glycoprotein 4-beta-N-acetylglucosaminyltransferase C OS=Homo sapiens GN=MGAT4C PE=2 S	21850	77908	29157	2,67
Q9P2P6	StAR-related lipid transfer protein 9 OS=Homo sapiens GN=STARD9 PE=1 SV=3 - [STAR9_HUMAN]	2225654	3047024	1151132	2,65
O00443	Phosphatidylinositol 4-phosphate 3-kinase C2 domain-containing subunit alpha OS=Homo sapiens GN=PIK3C2A PE=	5851954	9626104	3644453	2,64
Q5VZK9	Leucine-rich repeat-containing protein 16A OS=Homo sapiens GN=LRRC16A PE=1 SV=1 - [LR16A_HUMAN]	354636	894206	340215	2,63
P35555	Fibrillin-1 OS=Homo sapiens GN=FBN1 PE=1 SV=3 - [FBN1_HUMAN]	430451	334503	134917	2,48
Q96F63	Coiled-coil domain-containing protein 97 OS=Homo sapiens GN=CCDC97 PE=1 SV=1 - [CCD97_HUMAN]	3360512	6735018	2803151	2,40
P19022	Cadherin-2 OS=Homo sapiens GN=CDH2 PE=1 SV=4 - [CADH2_HUMAN]	3203031	2695055	1132667	2,38
Q9HCE7	E3 ubiquitin-protein ligase SMURF1 OS=Homo sapiens GN=SMURF1 PE=1 SV=2 - [SMUF1_HUMAN]	33828231	36811710	15473308	2,38
Q9P212	1-phosphatidylinositol 4,5-bisphosphate phosphodiesterase epsilon-1 OS=Homo sapiens GN=PLCE1 PE=1 SV=3 - [PL	5575551	7291016	3065990	2,38
P10451	Osteopontin OS=Homo sapiens GN=SPP1 PE=1 SV=1 - [OSTP_HUMAN]	82893180	74526397	31751509	2,35

Q9H707	Zinc finger protein 552 OS=Homo sapiens GN=ZNF552 PE=1 SV=2 - [ZN552_HUMAN]	455369141	68545638	29804070	2,30
Q6NUM9	All-trans-retinol 13,14-reductase OS=Homo sapiens GN=RETSAT PE=1 SV=2 - [RETST_HUMAN]	8044213	7838640	3421734	2,29
Q8IXT5	RNA-binding protein 12B OS=Homo sapiens GN=RBM12B PE=1 SV=2 - [RB12B_HUMAN]	19619288	17589896	7776232	2,26
Q9UFE4	Coiled-coil domain-containing protein 39 OS=Homo sapiens GN=CCDC39 PE=2 SV=3 - [CCD39_HUMAN]	2873277	2131724	956850	2,23
Q9ULD0	2-oxoglutarate dehydrogenase-like, mitochondrial OS=Homo sapiens GN=OGDHL PE=1 SV=3 - [OGDHL_HUMAN]	6240174	6971886	3143241	2,22
P04278	Sex hormone-binding globulin OS=Homo sapiens GN=SHBG PE=1 SV=2 - [SHBG_HUMAN]	8335991	7618348	3492956	2,18
Q15904	V-type proton ATPase subunit S1 OS=Homo sapiens GN=ATP6AP1 PE=1 SV=2 - [VAS1_HUMAN]	58898362	53520426	24568060	2,18
Q9P121	Neurotrimin OS=Homo sapiens GN=NTM PE=1 SV=1 - [NTRI_HUMAN]	391764	314068	148381	2,12
Q08629	Testican-1 OS=Homo sapiens GN=SPOCK1 PE=1 SV=1 - [TICN1_HUMAN]	7145650	8126187	3986185	2,04
P02649	Apolipoprotein E OS=Homo sapiens GN=APOE PE=1 SV=1 - [APOE_HUMAN]	104475654	99376722	48756502	2,04
Q92621	Nuclear pore complex protein Nup205 OS=Homo sapiens GN=NUP205 PE=1 SV=3 - [NU205_HUMAN]	1388028	2860975	1465437	1,95
P41222	Prostaglandin-H2 D-isomerase OS=Homo sapiens GN=PTGDS PE=1 SV=1 - [PTGDS_HUMAN]	522242177	606791405	316899128	1,91
Q13822	Ectonucleotide pyrophosphatase/phosphodiesterase family member 2 OS=Homo sapiens GN=ENPP2 PE=1 SV=3 - [I	22141292	27623781	14481310	1,91
P22352	Glutathione peroxidase 3 OS=Homo sapiens GN=GPX3 PE=1 SV=2 - [GPX3_HUMAN]	48637707	51872238	27560264	1,88
Q15392	Delta(24)-sterol reductase OS=Homo sapiens GN=DHCR24 PE=1 SV=2 - [DHC24_HUMAN]	2656620	3401462	1816382	1,87
P48051	G protein-activated inward rectifier potassium channel 2 OS=Homo sapiens GN=KCNJ6 PE=1 SV=1 - [KCNJ6_HUMAI	539608	1381760	741909	1,86
P46721	Solute carrier organic anion transporter family member 1A2 OS=Homo sapiens GN=SLCO1A2 PE=2 SV=1 - [SO1A2_I	31214867	33558606	18286878	1,84
Q9NSY0	Nuclear receptor-binding protein 2 OS=Homo sapiens GN=NRBP2 PE=2 SV=2 - [NRBP2_HUMAN]	1159031	939506	513619	1,83
Q9NQ79	Cartilage acidic protein 1 OS=Homo sapiens GN=CRTAC1 PE=1 SV=2 - [CRAC1_HUMAN]	5114542	5733297	3148207	1,82
P02100	Hemoglobin subunit epsilon OS=Homo sapiens GN=HBE1 PE=1 SV=2 - [HBE_HUMAN]	512854	321954	181279	1,78
Q9P267	Methyl-CpG-binding domain protein 5 OS=Homo sapiens GN=MBD5 PE=1 SV=3 - [MBD5_HUMAN]	5338478	7070970	4074987	1,74
Q9HC35	Echinoderm microtubule-associated protein-like 4 OS=Homo sapiens GN=EML4 PE=1 SV=3 - [EMAL4_HUMAN]	8637339	17332211	10119597	1,71
Q9NWR8	Calcium uniporter regulatory subunit MCUb, mitochondrial OS=Homo sapiens GN=CCDC109B PE=1 SV=2 - [MCUB_I	7815719	9210426	5458034	1,69
Q9P266	Junctional protein associated with coronary artery disease OS=Homo sapiens GN=KIAA1462 PE=1 SV=3 - [JCAD_HU	499281	1084900	652741	1,66
O43505	Beta-1,4-glucuronyltransferase 1 OS=Homo sapiens GN=B4GAT1 PE=1 SV=1 - [B4GA1_HUMAN]	12219966	10542133	6465638	1,63
Q12805	EGF-containing fibulin-like extracellular matrix protein 1 OS=Homo sapiens GN=EFEMP1 PE=1 SV=2 - [FBLN3_HUMA	36172522	64307594	41767332	1,54
P50458	LIM/homeobox protein Lhx2 OS=Homo sapiens GN=LHX2 PE=2 SV=2 - [LHX2_HUMAN]	5507969	7359067	4971806	1,48
P36955	Pigment epithelium-derived factor OS=Homo sapiens GN=SERPINF1 PE=1 SV=4 - [PEDF_HUMAN]	810767185	784648119	531102258	1,48
P15498	Proto-oncogene vav OS=Homo sapiens GN=VAV1 PE=1 SV=4 - [VAV_HUMAN]	29128253	77090456	54628943	1,41
Q96LB9	Peptidoglycan recognition protein 3 OS=Homo sapiens GN=PGLYRP3 PE=1 SV=1 - [PGRP3_HUMAN]	100857914	92228812	75096059	1,23
Q9HCB6	Spondin-1 OS=Homo sapiens GN=SPON1 PE=1 SV=2 - [SPON1_HUMAN]	132096817	108111169	141015001	0,77
P06681	Complement C2 OS=Homo sapiens GN=C2 PE=1 SV=2 - [CO2_HUMAN]	4033194	4172678	5625678	0,74
P49589	Cysteine--tRNA ligase, cytoplasmic OS=Homo sapiens GN=CARS PE=1 SV=3 - [SYCC_HUMAN]	6180970	7396977	10268436	0,72
P0C0L4	Complement C4-A OS=Homo sapiens GN=C4A PE=1 SV=2 - [CO4A_HUMAN]	213168444	193135678	268518808	0,72
Q6I9Y2	THO complex subunit 7 homolog OS=Homo sapiens GN=THOC7 PE=1 SV=3 - [THOC7_HUMAN]	22659150	26689996	38288944	0,70
P00751	Complement factor B OS=Homo sapiens GN=CFB PE=1 SV=2 - [CFAB_HUMAN]	73334533	77634619	112032082	0,69
Q92611	ER degradation-enhancing alpha-mannosidase-like protein 1 OS=Homo sapiens GN=EDEM1 PE=1 SV=1 - [EDEM1_H	2736914	1992962	2933501	0,68
P01024	Complement C3 OS=Homo sapiens GN=C3 PE=1 SV=2 - [CO3_HUMAN]	214705954	204192921	306869834	0,67
P01622	Ig kappa chain V-III region Ti OS=Homo sapiens PE=1 SV=1 - [KV304_HUMAN]	5117978	4809507	7231463	0,67
P08185	Corticosteroid-binding globulin OS=Homo sapiens GN=SERPINA6 PE=1 SV=1 - [CBG_HUMAN]	11273920	9808540	15457606	0,63
P25092	Heat-stable enterotoxin receptor OS=Homo sapiens GN=GUCY2C PE=1 SV=2 - [GUC2C_HUMAN]	16467404	29803585	47667557	0,63
Q2PPJ7	Ral GTPase-activating protein subunit alpha-2 OS=Homo sapiens GN=RALGAPA2 PE=1 SV=2 - [RGPA2_HUMAN]	1107763	1852063	3006316	0,62
Q9Y2U5	Mitogen-activated protein kinase kinase kinase 2 OS=Homo sapiens GN=MAP3K2 PE=1 SV=2 - [M3K2_HUMAN]	169650379	198088037	322670625	0,61
A8MPX8	Protein phosphatase 2C-like domain-containing protein 1 OS=Homo sapiens GN=PP2D1 PE=2 SV=2 - [PP2D1_HUMA	2989993	3446440	5669932	0,61
Q9HC62	Sentrin-specific protease 2 OS=Homo sapiens GN=SENP2 PE=1 SV=3 - [SENP2_HUMAN]	24566630	19544834	32230330	0,61
Q2WGJ9	Fer-1-like protein 6 OS=Homo sapiens GN=FER1L6 PE=2 SV=2 - [FR1L6_HUMAN]	139366498	165153416	275816956	0,60
Q86VZ6	Juxtaposed with another zinc finger protein 1 OS=Homo sapiens GN=JAZF1 PE=1 SV=2 - [JAZF1_HUMAN]	2716052287	1810425378	3039985889	0,60
Q5FWE3	Proline-rich transmembrane protein 3 OS=Homo sapiens GN=PRRT3 PE=1 SV=3 - [PRRT3_HUMAN]	316768123	259953746	448219751	0,58
P00747	Plasminogen OS=Homo sapiens GN=PLG PE=1 SV=2 - [PLMN_HUMAN]	53683767	64740691	113958036	0,57
Q15582	Transforming growth factor-beta-induced protein ig-h3 OS=Homo sapiens GN=TGFBI PE=1 SV=1 - [BGH3_HUMAN]	2416909	2376919	4255789	0,56
P07360	Complement component C8 gamma chain OS=Homo sapiens GN=C8G PE=1 SV=3 - [CO8G_HUMAN]	1907874	2201776	3970766	0,55
P02751	Fibronectin OS=Homo sapiens GN=FN1 PE=1 SV=4 - [FINC_HUMAN]	36573293	65555579	119838606	0,55
P02748	Complement component C9 OS=Homo sapiens GN=C9 PE=1 SV=2 - [CO9_HUMAN]	15127942	19035124	34989747	0,54
Q86V88	Magnesium-dependent phosphatase 1 OS=Homo sapiens GN=MDP1 PE=1 SV=1 - [MGDP1_HUMAN]	419643	142954	263057	0,54
P07858	Cathepsin B OS=Homo sapiens GN=CTSB PE=1 SV=3 - [CATB_HUMAN]	770641	555090	1030785	0,54
Q9H497	Torsin-3A OS=Homo sapiens GN=TOR3A PE=1 SV=1 - [TOR3A_HUMAN]	3106844	2364707	4420768	0,53
O43435	T-box transcription factor TBX1 OS=Homo sapiens GN=TBX1 PE=1 SV=1 - [TBX1_HUMAN]	547305	901663	1693157	0,53
Q8IUR5	Transmembrane and TPR repeat-containing protein 1 OS=Homo sapiens GN=TMTC1 PE=1 SV=3 - [TMTC1_HUMAN]	8932101	11568837	21872622	0,53
Q9UPU5	Ubiquitin carboxyl-terminal hydrolase 24 OS=Homo sapiens GN=USP24 PE=1 SV=3 - [UBP24_HUMAN]	10617252	10552749	20461872	0,52
Q12841	Follistatin-related protein 1 OS=Homo sapiens GN=FSTL1 PE=1 SV=1 - [FSTL1_HUMAN]	26043744	51648101	100742484	0,51
Q53RD9	Fibulin-7 OS=Homo sapiens GN=FBLN7 PE=2 SV=1 - [FBLN7_HUMAN]	26774512	5019928	9828165	0,51
P02765	Alpha-2-HS-glycoprotein OS=Homo sapiens GN=AHSG PE=1 SV=1 - [FETUA_HUMAN]	138444305	118005366	231591614	0,51
P00734	Prothrombin OS=Homo sapiens GN=F2 PE=1 SV=2 - [THRB_HUMAN]	24043988	32216375	63237379	0,51
Q15185	Prostaglandin E synthase 3 OS=Homo sapiens GN=PTGES3 PE=1 SV=1 - [TEBP_HUMAN]	1132285	1106913	2233891	0,50
Q9BY67	Cell adhesion molecule 1 OS=Homo sapiens GN=CADM1 PE=1 SV=2 - [CADM1_HUMAN]	1729528	1964022	3978562	0,49
Q14624	Inter-alpha-trypsin inhibitor heavy chain H4 OS=Homo sapiens GN=ITIH4 PE=1 SV=4 - [ITIH4_HUMAN]	9790530	7757702	16037370	0,48
Q92743	Serine protease HTRA1 OS=Homo sapiens GN=HTRA1 PE=1 SV=1 - [HTRA1_HUMAN]	584543	580916	1201225	0,48
Q7RTZ1	Ovochymase-2 OS=Homo sapiens GN=OVCH2 PE=3 SV=2 - [OVCH2_HUMAN]	2253252	1485789	3199565	0,46
O15195	Villin-like protein OS=Homo sapiens GN=VILL PE=2 SV=3 - [VILL_HUMAN]	64054082	37755995	81769390	0,46
P01042	Kininogen-1 OS=Homo sapiens GN=KNG1 PE=1 SV=2 - [KNG1_HUMAN]	48817639	43589414	94797310	0,46
P01876	Ig alpha-1 chain C region OS=Homo sapiens GN=IGHA1 PE=1 SV=2 - [IGHA1_HUMAN]	36542909	48083717	104616219	0,46
P02749	Beta-2-glycoprotein 1 OS=Homo sapiens GN=APOH PE=1 SV=3 - [APOH_HUMAN]	38509501	41906736	92297205	0,45
Q9BU40	Chordin-like protein 1 OS=Homo sapiens GN=CHRDL1 PE=1 SV=1 - [CRDL1_HUMAN]	394675	357167	815945	0,44
Q9UJX3	Anaphase-promoting complex subunit 7 OS=Homo sapiens GN=ANAPC7 PE=1 SV=4 - [APC7_HUMAN]	1384986	1155703	2644451	0,44
Q01484	Ankyrin-2 OS=Homo sapiens GN=ANK2 PE=1 SV=4 - [ANK2_HUMAN]	159263502	138111323	324402896	0,43
Q68D51	DENN domain-containing protein 2C OS=Homo sapiens GN=DENND2C PE=1 SV=2 - [DEN2C_HUMAN]	511991	363876	880327	0,41
O95251	Histone acetyltransferase KAT7 OS=Homo sapiens GN=KAT7 PE=1 SV=1 - [KAT7_HUMAN]	4083350	3818117	9603053	0,40
Q2VY69	Zinc finger protein 284 OS=Homo sapiens GN=ZNF284 PE=2 SV=1 - [ZN284_HUMAN]	360839	237250	605338	0,39
Q9UGM5	Fetuin-B OS=Homo sapiens GN=FETUB PE=1 SV=2 - [FETUB_HUMAN]	347893	455885	1163765	0,39
O43174	Cytochrome P450 26A1 OS=Homo sapiens GN=CYP26A1 PE=2 SV=2 - [CP26A_HUMAN]	108011	272473	707278	0,39
P02760	Protein AMBP OS=Homo sapiens GN=AMBP PE=1 SV=1 - [AMBP_HUMAN]	6211489	9438425	24570836	0,38
P05090	Apolipoprotein D OS=Homo sapiens GN=APOD PE=1 SV=1 - [APOD_HUMAN]	477131	561351	1482977	0,38
P01611	Ig kappa chain V-I region Wes OS=Homo sapiens PE=1 SV=1 - [KV119_HUMAN]	531439	1018899	2698117	0,38
Q86UK0	ATP-binding cassette sub-family A member 12 OS=Homo sapiens GN=ABCA12 PE=1 SV=3 - [ABCAC_HUMAN]	2070741	2021925	5355389	0,38
Q8N392	Rho GTPase-activating protein 18 OS=Homo sapiens GN=ARHGAP18 PE=1 SV=3 - [RHG18_HUMAN]	465234	420545	1135455	0,37
Q9C0B1	Alpha-ketoglutarate-dependent dioxygenase FTO OS=Homo sapiens GN=FTO PE=1 SV=3 - [FTO_HUMAN]	5222953	4817730	13021370	0,37

O75882	Attractin OS=Homo sapiens GN=ATRN PE=1 SV=2 - [ATRN_HUMAN]	42756	50875	138604	0,37
Q02386	Zinc finger protein 45 OS=Homo sapiens GN=ZNF45 PE=2 SV=2 - [ZNF45_HUMAN]	1041158	663040	1836787	0,36
P19827	Inter-alpha-trypsin inhibitor heavy chain H1 OS=Homo sapiens GN=ITIH1 PE=1 SV=3 - [ITIH1_HUMAN]	12263795	8014836	22543183	0,36
P06312	Ig kappa chain V-IV region (Fragment) OS=Homo sapiens GN=IGKV4-1 PE=4 SV=1 - [KV401_HUMAN]	50974	67913	195550	0,35
P01033	Metalloproteinase inhibitor 1 OS=Homo sapiens GN=TIMP1 PE=1 SV=1 - [TIMP1_HUMAN]	984406	642435	1868741	0,34
Q03591	Complement factor H-related protein 1 OS=Homo sapiens GN=CFHR1 PE=1 SV=2 - [FHR1_HUMAN]	2621027	3045161	8952355	0,34
Q6PL18	ATPase family AAA domain-containing protein 2 OS=Homo sapiens GN=ATAD2 PE=1 SV=1 - [ATAD2_HUMAN]	15997	33183	98194	0,34
Q5VZE5	N-alpha-acetyltransferase 35, NatC auxiliary subunit OS=Homo sapiens GN=NAA35 PE=1 SV=1 - [NAA35_HUMAN]	3133523	3261113	9695524	0,34
Q5T7N2	LINE-1 type transposase domain-containing protein 1 OS=Homo sapiens GN=L1TD1 PE=1 SV=1 - [LITD1_HUMAN]	39201868	60430749	180830646	0,33
Q96IY4	Carboxypeptidase B2 OS=Homo sapiens GN=CPB2 PE=1 SV=2 - [CBPB2_HUMAN]	511625	425822	1275283	0,33
Q15911	Zinc finger homeobox protein 3 OS=Homo sapiens GN=ZFHX3 PE=1 SV=2 - [ZFHX3_HUMAN]	59974	111970	335825	0,33
P01764	Ig heavy chain V-III region 23 OS=Homo sapiens GN=IGHV3-23 PE=1 SV=2 - [HV303_HUMAN]	117424	214392	655346	0,33
P53804	E3 ubiquitin-protein ligase TTC3 OS=Homo sapiens GN=TTC3 PE=1 SV=2 - [TTC3_HUMAN]	1747013	616662	1896409	0,33
Q9UM82	Spermatogenesis-associated protein 2 OS=Homo sapiens GN=SPATA2 PE=1 SV=2 - [SPAT2_HUMAN]	53583	43296	133637	0,32
Q8NCT3	Uncharacterized protein KIAA0895 OS=Homo sapiens GN=KIAA0895 PE=2 SV=4 - [K0895_HUMAN]	7451168	10143524	31499897	0,32
P61160	Actin-related protein 2 OS=Homo sapiens GN=ACTR2 PE=1 SV=1 - [ARP2_HUMAN]	27525386	33896755	105460593	0,32
O94915	Protein furry homolog-like OS=Homo sapiens GN=FRYL PE=1 SV=2 - [FRYL_HUMAN]	45105200	30200144	95849305	0,32
Q9UPT9	Ubiquitin carboxyl-terminal hydrolase 22 OS=Homo sapiens GN=USP22 PE=1 SV=2 - [UBP22_HUMAN]	4506525	5063150	16386539	0,31
P10643	Complement component C7 OS=Homo sapiens GN=C7 PE=1 SV=2 - [CO7_HUMAN]	1726957	1563257	5084386	0,31
Q9BYW2	Histone-lysine N-methyltransferase SETD2 OS=Homo sapiens GN=SETD2 PE=1 SV=3 - [SETD2_HUMAN]	2729824	6245095	20354142	0,31
Q96MI9	Cytosolic carboxypeptidase 4 OS=Homo sapiens GN=AGBL1 PE=1 SV=2 - [CBPC4_HUMAN]	1265305	115153	385610	0,30
Q9NS87	Kinesin-like protein KIF15 OS=Homo sapiens GN=KIF15 PE=1 SV=1 - [KIF15_HUMAN]	2037984	2311712	8020411	0,29
Q9BYJ0	Fibroblast growth factor-binding protein 2 OS=Homo sapiens GN=FGFBP2 PE=1 SV=1 - [FGFP2_HUMAN]	90311	135714	479261	0,28
Q96PY5	Formin-like protein 2 OS=Homo sapiens GN=FMNL2 PE=1 SV=3 - [FMNL2_HUMAN]	1752579	1173551	4181980	0,28
P06865	Beta-hexosaminidase subunit alpha OS=Homo sapiens GN=HEXA PE=1 SV=2 - [HEXA_HUMAN]	47186269	47224227	169785781	0,28
P02679	Fibrinogen gamma chain OS=Homo sapiens GN=FGG PE=1 SV=3 - [FIBG_HUMAN]	19790041	11616118	42118814	0,28
Q92541	RNA polymerase-associated protein RTF1 homolog OS=Homo sapiens GN=RTF1 PE=1 SV=4 - [RTF1_HUMAN]	352848	294757	1095850	0,27
Q8NEL0	Coiled-coil domain-containing protein 54 OS=Homo sapiens GN=CCDC54 PE=1 SV=2 - [CCD54_HUMAN]	1411242	1511339	5848602	0,26
Q8N945	PRELI domain-containing protein 2 OS=Homo sapiens GN=PRELID2 PE=2 SV=1 - [PRLD2_HUMAN]	92221901	41309964	163344177	0,25
Q9H2F5	Enhancer of polycomb homolog 1 OS=Homo sapiens GN=EPC1 PE=1 SV=1 - [EPC1_HUMAN]	612308	519246	2115838	0,25
P00740	Coagulation factor IX OS=Homo sapiens GN=F9 PE=1 SV=2 - [FA9_HUMAN]	67410	64567	264160	0,24
P62736	Actin, aortic smooth muscle OS=Homo sapiens GN=ACTA2 PE=1 SV=1 - [ACTA_HUMAN]	691018	337722	1414754	0,24
P02671	Fibrinogen alpha chain OS=Homo sapiens GN=FGA PE=1 SV=2 - [FIBA_HUMAN]	4192344	3546865	14904251	0,24
Q5T9S5	Coiled-coil domain-containing protein 18 OS=Homo sapiens GN=CCDC18 PE=2 SV=1 - [CCD18_HUMAN]	998772	729461	3099642	0,24
Q96N87	Sodium-dependent neutral amino acid transporter B(0)AT3 OS=Homo sapiens GN=SLC6A18 PE=2 SV=2 - [S6A18_HU	1280404	930736	4078457	0,23
P09486	SPARC OS=Homo sapiens GN=SPARC PE=1 SV=1 - [SPRC_HUMAN]	320296	172323	755598	0,23
P03952	Plasma kallikrein OS=Homo sapiens GN=KLKB1 PE=1 SV=1 - [KLKB1_HUMAN]	84732	73038	325372	0,22
Q8IY92	Structure-specific endonuclease subunit SLX4 OS=Homo sapiens GN=SLX4 PE=1 SV=3 - [SLX4_HUMAN]	569010	1028135	4684532	0,22
Q9NQX4	Unconventional myosin-Vc OS=Homo sapiens GN=MYO5C PE=1 SV=2 - [MYO5C_HUMAN]	181693	39805	183835	0,22
Q6ZTR5	Cilia- and flagella-associated protein 47 OS=Homo sapiens GN=CFAP47 PE=2 SV=4 - [CFA47_HUMAN]	328383	245609	1134461	0,22
O75445	Usherin OS=Homo sapiens GN=USH2A PE=1 SV=3 - [USH2A_HUMAN]	310258	434342	2027622	0,21
Q8TDJ6	DmX-like protein 2 OS=Homo sapiens GN=DMXL2 PE=1 SV=2 - [DMXL2_HUMAN]	9725	21912	104835	0,21
P42262	Glutamate receptor 2 OS=Homo sapiens GN=GRIA2 PE=1 SV=3 - [GRIA2_HUMAN]	171661	117243	561970	0,21
P05813	Beta-crystallin A3 OS=Homo sapiens GN=CRYBA1 PE=1 SV=4 - [CRBA1_HUMAN]	51599	22050	106301	0,21
Q8WZ42	Titin OS=Homo sapiens GN=TTN PE=1 SV=4 - [TITIN_HUMAN]	25038847	26923662	130465382	0,21
O00512	B-cell CLL/lymphoma 9 protein OS=Homo sapiens GN=BCL9 PE=1 SV=4 - [BCL9_HUMAN]	64989	87279	428300	0,20
P01344	Insulin-like growth factor II OS=Homo sapiens GN=IGF2 PE=1 SV=1 - [IGF2_HUMAN]	70840	90706	467774	0,19
P36980	Complement factor H-related protein 2 OS=Homo sapiens GN=CFHR2 PE=1 SV=1 - [FHR2_HUMAN]	65499	97495	505118	0,19
Q8IUG5	Unconventional myosin-XVIIIb OS=Homo sapiens GN=MYO18B PE=1 SV=1 - [MY18B_HUMAN]	64077	22841	118636	0,19
A1L0T0	Acetolactate synthase-like protein OS=Homo sapiens GN=ILVBL PE=1 SV=2 - [ILVBL_HUMAN]	1050778	821188	4291438	0,19
Q86VY4	Testis-specific Y-encoded-like protein 5 OS=Homo sapiens GN=TSPYL5 PE=1 SV=2 - [TSYL5_HUMAN]	393739	247345	1302462	0,19
Q13219	Pappalysin-1 OS=Homo sapiens GN=PAPPA PE=1 SV=3 - [PAPP1_HUMAN]	182998	111989	590291	0,19
P0C7V8	DDB1- and CUL4-associated factor 8-like protein 2 OS=Homo sapiens GN=DCAF8L2 PE=2 SV=2 - [DC8L2_HUMAN]	2561309	1279511	6764568	0,19
P0DJI9	Serum amyloid A-2 protein OS=Homo sapiens GN=SAA2 PE=1 SV=1 - [SAA2_HUMAN]	56676	14042	78119	0,18
Q15746	Myosin light chain kinase, smooth muscle OS=Homo sapiens GN=MYLK PE=1 SV=4 - [MYLK_HUMAN]	447407	191986	1088989	0,18
O95995	Growth arrest-specific protein 8 OS=Homo sapiens GN=GAS8 PE=1 SV=1 - [GAS8_HUMAN]	20500	21551	126280	0,17
Q8TF05	Serine/threonine-protein phosphatase 4 regulatory subunit 1 OS=Homo sapiens GN=PPP4R1 PE=1 SV=1 - [PP4R1_H	178798	126967	777754	0,16
P02675	Fibrinogen beta chain OS=Homo sapiens GN=FGB PE=1 SV=2 - [FIBB_HUMAN]	6745870	5032237	31952949	0,16
P49448	Glutamate dehydrogenase 2, mitochondrial OS=Homo sapiens GN=GLUD2 PE=1 SV=2 - [DHE4_HUMAN]	178367	28595	188552	0,15
Q5JVG2	Zinc finger protein 484 OS=Homo sapiens GN=ZNF484 PE=1 SV=1 - [ZN484_HUMAN]	22762660	7180295	48081892	0,15
P60709	Actin, cytoplasmic 1 OS=Homo sapiens GN=ACTB PE=1 SV=1 - [ACTB_HUMAN]	331892	119430	838424	0,14
Q8N1B4	Vacuolar protein sorting-associated protein 52 homolog OS=Homo sapiens GN=VPS52 PE=1 SV=1 - [VPS52_HUMAN	94101	54880	388732	0,14
P05160	Coagulation factor XIII B chain OS=Homo sapiens GN=F13B PE=1 SV=3 - [F13B_HUMAN]	36105	7809	57352	0,14
Q68CZ2	Tensin-3 OS=Homo sapiens GN=TNS3 PE=1 SV=2 - [TENS3_HUMAN]	105557	157478	1169060	0,13
Q8IWP9	Coiled-coil domain-containing protein 28A OS=Homo sapiens GN=CCDC28A PE=1 SV=1 - [CC28A_HUMAN]	10334179	2347563	19222375	0,12
Q14699	Raftlin OS=Homo sapiens GN=RFTN1 PE=1 SV=4 - [RFTN1_HUMAN]	10134	28148	237319	0,12
Q9NZJ4	Sacsin OS=Homo sapiens GN=SACS PE=1 SV=2 - [SACS_HUMAN]	7514	21747	184784	0,12
Q9UKN7	Unconventional myosin-XV OS=Homo sapiens GN=MYO15A PE=1 SV=2 - [MYO15_HUMAN]	127664	432033	3889663	0,11
P06310	Ig kappa chain V-II region RPMI 6410 OS=Homo sapiens PE=4 SV=1 - [KV206_HUMAN]	15216	5358	50073	0,11
P35542	Serum amyloid A-4 protein OS=Homo sapiens GN=SAA4 PE=1 SV=2 - [SAA4_HUMAN]	64033	20599	197438	0,10
Q9H706	GRB2-associated and regulator of MAPK protein 1 OS=Homo sapiens GN=GAREM1 PE=1 SV=2 - [GARE1_HUMAN]	308120	115941	1174665	0,10
Q9UPS8	Ankyrin repeat domain-containing protein 26 OS=Homo sapiens GN=ANKRD26 PE=1 SV=3 - [ANR26_HUMAN]	1308605	2706651	28934735	0,09
Q96L92	Sorting nexin-27 OS=Homo sapiens GN=SNX27 PE=1 SV=2 - [SNX27_HUMAN]	1275112	103225	1122922	0,09
Q15828	Cystatin-M OS=Homo sapiens GN=CST6 PE=1 SV=1 - [CYTM_HUMAN]	25458	11709	132349	0,09
Q9Y4F3	Meiosis arrest female protein 1 OS=Homo sapiens GN=KIAA0430 PE=1 SV=6 - [MARF1_HUMAN]	2408	12260	147521	0,08
O14628	Zinc finger protein 195 OS=Homo sapiens GN=ZNF195 PE=1 SV=2 - [ZN195_HUMAN]	8656923	7366716	92950626	0,08
Q15468	SCL-interrupting locus protein OS=Homo sapiens GN=STIL PE=1 SV=2 - [STIL_HUMAN]	265496	80136	1034868	0,08
Q8TF20	Zinc finger protein 721 OS=Homo sapiens GN=ZNF721 PE=2 SV=2 - [ZN721_HUMAN]	94576	118035	1642880	0,07
Q8NF50	Dedicator of cytokinesis protein 8 OS=Homo sapiens GN=DOCK8 PE=1 SV=3 - [DOCK8_HUMAN]	9504	34517	522527	0,07
Q96MT1	RING finger protein 145 OS=Homo sapiens GN=RNF145 PE=2 SV=2 - [RN145_HUMAN]	24811	31488	516777	0,06
Q9UGJ0	5'-AMP-activated protein kinase subunit gamma-2 OS=Homo sapiens GN=PRKAG2 PE=1 SV=1 - [AAKG2_HUMAN]	13243	2660	48010	0,06
Q8NGQ3	Olfactory receptor 1S2 OS=Homo sapiens GN=OR1S2 PE=3 SV=2 - [OR1S2_HUMAN]	69548	486	9005	0,05
Q7Z478	ATP-dependent RNA helicase DHX29 OS=Homo sapiens GN=DHX29 PE=1 SV=2 - [DHX29_HUMAN]	2445714	348618	6539846	0,05
Q7RTW8	Otoancorin OS=Homo sapiens GN=OTOA PE=1 SV=1 - [OTOAN_HUMAN]	20364	7068	134465	0,05
Q93050	V-type proton ATPase 116 kDa subunit a isoform 1 OS=Homo sapiens GN=ATP6V0A1 PE=1 SV=3 - [VPP1_HUMAN]	142482	43640	895208	0,05
P98161	Polycystin-1 OS=Homo sapiens GN=PKD1 PE=1 SV=3 - [PKD1_HUMAN]	10322	528	10985	0,05

P20396	Pro-thyrotropin-releasing hormone OS=Homo sapiens GN=TRH PE=1 SV=1 - [TRH_HUMAN]	2459	12297	272381	0,05
P60022	Beta-defensin 1 OS=Homo sapiens GN=DEFB1 PE=1 SV=1 - [DEFB1_HUMAN]	2869	1113	26186	0,04
Q9H5N1	Rab GTPase-binding effector protein 2 OS=Homo sapiens GN=RABEP2 PE=1 SV=2 - [RABE2_HUMAN]	15247	13450	318079	0,04
P24539	ATP synthase F(0) complex subunit B1, mitochondrial OS=Homo sapiens GN=ATP5F1 PE=1 SV=2 - [AT5F1_HUMAN]	28313	16155	451024	0,04
P61812	Transforming growth factor beta-2 OS=Homo sapiens GN=TGFB2 PE=1 SV=1 - [TGFB2_HUMAN]	1110	547	21289	0,03
Q86Z14	Beta-klotho OS=Homo sapiens GN=KLB PE=1 SV=1 - [KLOTB_HUMAN]	661	297	12263	0,02
Q7LGC8	Carbohydrate sulfotransferase 3 OS=Homo sapiens GN=CHST3 PE=1 SV=3 - [CHST3_HUMAN]	93233	24369	1080296	0,02
P10523	S-arrestin OS=Homo sapiens GN=SAG PE=1 SV=3 - [ARRS_HUMAN]	754	1242	62103	0,02
P68871	Hemoglobin subunit beta OS=Homo sapiens GN=HBB PE=1 SV=2 - [HBB_HUMAN]	4893086	3601152	188088670	0,02
Q9NYB0	Telomeric repeat-binding factor 2-interacting protein 1 OS=Homo sapiens GN=TERF2IP PE=1 SV=1 - [TE2IP_HUMAN	7819	2111	131412	0,02
Q96N64	PWWP domain-containing protein 2A OS=Homo sapiens GN=PWWP2A PE=1 SV=2 - [PWP2A_HUMAN]	49212	20414	2231265	0,01
P69905	Hemoglobin subunit alpha OS=Homo sapiens GN=HBA1 PE=1 SV=2 - [HBA_HUMAN]	1216046	376605	42122572	0,01
Q69YH5	Cell division cycle-associated protein 2 OS=Homo sapiens GN=CDCA2 PE=1 SV=2 - [CDCA2_HUMAN]	30573	2566	322195	0,01
P62873	Guanine nucleotide-binding protein G(I)/G(S)/G(T) subunit beta-1 OS=Homo sapiens GN=GNB1 PE=1 SV=3 - [GBB1_	2581	667	86134	0,01
