# Supplementary material for: Systems pathology analysis identifies neurodegenerative nature of age‐related vitreoretinal interface diseases
Source: Aging Cell. 2018 Jul 2;17(5):e12809. doi: 10.1111/acel.12809 (PMC6156470; doi:10.1111/acel.12809)
Supplement: Supplementary file 10 [file ACEL-17-e12809-s010.pdf]

Supplemental Table S7: 37 neuronal proteins were present at higher level in iERM and/or MH samples compared to DME and their 90 interactors found in our vitreous proteome analysis.
Type: MH = found only in MH samples, iERM = found only in iERM samples, iERM+MH = found MH and iERM samples, X = interactors

Entry	Short protein name	Type	Protein names
O00533	NCHL1	MH	Neural cell adhesion molecule L1-like protein (Close homolog of L1) [Cleaved into: Processed neural cell adhesion molecule L1-like protein]
O14594	NCAN	MH	Neurocan core protein (Chondroitin sulfate proteoglycan 3)
O43157	PLXB1	MH	Plexin-B1 (Semaphorin receptor SEP)
P05408	7B2	MH	Neuroendocrine protein 7B2 (Pituitary polypeptide) (Secretogranin V) (Secretogranin-5) (Secretory granule endocrine protein I) [Cleaved into: N-terminal peptide; C-terminal peptide]
P10451	OSTP	MH	Osteopontin (Bone sialoprotein 1) (Nephropontin) (Secreted phosphoprotein 1) (SPP-1) (Urinary stone protein) (Uropontin)
P22304	IDS	MH	Iduronate 2-sulfatase (EC 3.1.6.13) (Alpha-L-iduronate sulfate sulfatase) (Idursulfase) [Cleaved into: Iduronate 2-sulfatase 42 kDa chain; Iduronate 2-sulfatase 14 kDa chain]
P42356	PI4KA	MH	Phosphatidylinositol 4-kinase alpha (PI4-kinase alpha) (PI4K-alpha) (PtdIns-4-kinase alpha) (EC 2.7.1.67)
P98164	LRP2	MH	Low-density lipoprotein receptor-related protein 2 (LRP-2) (Glycoprotein 330) (gp330) (Megalin)
Q08629	TICN1	MH	Testican-1 (Protein SPOCK)
Q13214	SEM3B	MH	Semaphorin-3B (Sema A(V)) (Semaphorin-V) (Sema V)
Q15173	2A5B	MH	Serine/threonine-protein phosphatase 2A 56 kDa regulatory subunit beta isoform (PP2A B subunit isoform B'-beta) (PP2A B subunit isoform B56-beta)
Q53EL9	SEZ6	MH	Seizure protein 6 homolog (SEZ-6) (hSEZ-6)
Q59EK9	RUN3A	MH	RUN domain-containing protein 3A (Rap2-interacting protein 8) (RPIP-8)
Q8IXJ6	SIR2	MH	NAD-dependent protein deacetylase sirtuin-2 (EC 3.5.1.-) (Regulatory protein SIR2 homolog 2) (SIR2-like protein 2)
Q8WXD2	SCG3	MH	Secretogranin-3 (Secretogranin III) (SgIII)
Q96F63	CCD97	MH	Coiled-coil domain-containing protein 97
Q99574	NEUS	MH	Neuroserpin (Peptidase inhibitor 12) (PI-12) (Serpin I1)
Q9UBX1	CATF	MH	Cathepsin F (CATSF) (EC 3.4.22.41)
Q9ULB1	NRX1A	MH	Neurexin-1 (Neurexin I-alpha) (Neurexin-1-alpha)
Q9ULD0	OGDHL	MH	2-oxoglutarate dehydrogenase-like, mitochondrial (EC 1.2.4.-) (2-oxoglutarate dehydrogenase complex component E1-like) (OGDC-E1-like) (Alpha-ketoglutarate dehydrogenase-like)
Q9Y4I1	MYO5A	MH	Unconventional myosin-Va (Dilute myosin heavy chain, non-muscle) (Myosin heavy chain 12) (Myosin-12) (Myoxin)
Q14679	TTLL4	iERM+MH	Tubulin polyglutamylase TTLL4
O15240	VGF	iERM+MH	Neurosecretory protein VGF [Cleaved into: Neuroendocrine regulatory peptide-1 (NERP-1); Neuroendocrine regulatory peptide-2 (NERP-2); Antimicrobial peptide VGF[554-577]]
O94985	CSTN1	iERM+MH	Calsyntenin-1 (Alcadein-alpha) (Alc-alpha) (Alzheimer-related cadherin-like protein) (Non-classical cadherin XB31alpha) [Cleaved into: Soluble Alc-alpha (SAlc-alpha)
P02649	APOE	iERM+MH	Apolipoprotein E (Apo-E)
P16519	NEC2	iERM+MH	Neuroendocrine convertase 2 (NEC 2) (EC 3.4.21.94) (KEX2-like endoprotease 2) (Prohormone convertase 2) (Proprotein convertase 2) (PC2)
P16870	CBPE	iERM+MH	Carboxypeptidase E (CPE) (EC 3.4.17.10) (Carboxypeptidase H) (CPH) (Enkephalin convertase) (Prohormone-processing carboxypeptidase)
P19022	CADH2	iERM+MH	Cadherin-2 (CDw325) (Neural cadherin) (N-cadherin) (CD antigen CD325)
P51693	APLP1	iERM+MH	Amyloid-like protein 1 (APLP) (APLP-1) [Cleaved into: C30]
Q14995	NR1D2	iERM+MH	Nuclear receptor subfamily 1 group D member 2 (Orphan nuclear hormone receptor BD73) (Rev-erb alpha-related receptor) (RVR) (Rev-erb-beta) (V-erbA-related protein 1-related) (EAR-1R)
Q5T5U3	RHG21	iERM+MH	Rho GTPase-activating protein 21 (Rho GTPase-activating protein 10) (Rho-type GTPase-activating protein 21)
Q92823	NRCAM	iERM+MH	Neuronal cell adhesion molecule (Nr-CAM) (Neuronal surface protein Bravo) (hBravo) (NgCAM-related cell adhesion molecule) (Ng-CAM-related)
Q9H1K4	GHC2	iERM+MH	Mitochondrial glutamate carrier 2 (GC-2) (Glutamate/H(+) symporter 2) (Solute carrier family 25 member 18)
Q9UQB3	CTND2	iERM+MH	Catenin delta-2 (Delta-catenin) (GT24) (Neural plakophilin-related ARM-repeat protein) (NPRAP) (Neurojungin)
Q9Y5W5	WIF1	iERM+MH	Wnt inhibitory factor 1 (WIF-1)
Q8N1I0	DOCK4	iERM	Dedicator of cytokinesis protein 4
Q9NSY0	NRBP2	iERM	Nuclear receptor-binding protein 2 (Transformation-related gene 16 protein) (TRG-16)
O43166	SI1L1	x	Signal-induced proliferation-associated 1-like protein 1 (SIPA1-like protein 1) (High-risk human papilloma viruses E6 oncoproteins targeted protein 1) (E6-targeted protein 1)
P06396	GELS	x	Gelsolin (AGEL) (Actin-depolymerizing factor) (ADF) (Brevin)
P05067	A4	x	Amyloid beta A4 protein (ABPP) (APPI) (APP) (Alzheimer disease amyloid protein) (Amyloid precursor protein) (Beta-amyloid precursor protein) (Cerebral vascular amyloid peptide) (CVAP)
P02652	APOA2	x	Apolipoprotein A-II (Apo-AII) (ApoA-II) (Apolipoprotein A2) [Cleaved into: Proapolipoprotein A-II (ProapoA-II); Truncated apolipoprotein A-II (Apolipoprotein A-II(1-76))]
P02751	FINC	x	Fibronectin (FN) (Cold-insoluble globulin) (CIG) [Cleaved into: Anastellin; Ugl-Y1; Ugl-Y2; Ugl-Y3]
P00734	THRB	x	Prothrombin (EC 3.4.21.5) (Coagulation factor II) [Cleaved into: Activation peptide fragment 1; Activation peptide fragment 2; Thrombin light chain; Thrombin heavy chain]
P00738	HPT	x	Haptoglobin (Zonulin) [Cleaved into: Haptoglobin alpha chain; Haptoglobin beta chain]
P02765	FETUA	x	Alpha-2-HS-glycoprotein (Alpha-2-Z-globulin) (Ba-alpha-2-glycoprotein) (Fetuin-A) [Cleaved into: Alpha-2-HS-glycoprotein chain A; Alpha-2-HS-glycoprotein chain B]
P07711	CATL1	x	Cathepsin L1 (EC 3.4.22.15) (Cathepsin L) (Major excreted protein) (MEP) [Cleaved into: Cathepsin L1 heavy chain; Cathepsin L1 light chain]
P02671	FIBA	x	Fibrinogen alpha chain [Cleaved into: Fibrinopeptide A; Fibrinogen alpha chain]

P35222	CTNB1	x	Catenin beta-1 (Beta-catenin)
P02647	APOA1	x	Apolipoprotein A-I (Apo-AI) (ApoA-I) (Apolipoprotein A1) [Cleaved into: Proapolipoprotein A-I (ProapoA-I); Truncated apolipoprotein A-I (Apolipoprotein A-I(1-242))]
P10645	CMGA	x	Chromogranin-A (CgA) (Pituitary secretory protein I) (SP-I) [Cleaved into: Vasostatin-1 (Vasostatin I); Vasostatin-2 (Vasostatin II); EA-92; ES-43; Pancreastatin; SS-18; WA-8; WE-14; LF-19
P20396	TRH	x	Pro-thyrotropin-releasing hormone (Pro-TRH) (Prothyroliberin) [Cleaved into: Thyrotropin-releasing hormone (TRH) (Protirelin) (TSH-releasing factor) (Thyroliberin) (Thyrotropin-releasing factor) (TRF)]
Q9UBN7	HDAC6	x	Histone deacetylase 6 (HD6) (EC 3.5.1.98)
Q07954	LRP1	x	Prolow-density lipoprotein receptor-related protein 1 (LRP-1) (Alpha-2-macroglobulin receptor) (A2MR) (Apolipoprotein E receptor) (APOER) (CD antigen CD91)
P05155	IC1	x	Plasma protease C1 inhibitor (C1 Inh) (C1Inh) (C1 esterase inhibitor) (C1-inhibiting factor) (Serpin G1)
P08779	K1C16	x	Keratin, type I cytoskeletal 16 (Cytokeratin-16) (CK-16) (Keratin-16) (K16)
P24593	IBP5	x	Insulin-like growth factor-binding protein 5 (IBP-5) (IGF-binding protein 5) (IGFBP-5)
P02533	K1C14	x	Keratin, type I cytoskeletal 14 (Cytokeratin-14) (CK-14) (Keratin-14) (K14)
P08253	MMP2	x	72 kDa type IV collagenase (EC 3.4.24.24) (72 kDa gelatinase) (Gelatinase A) (Matrix metalloproteinase-2) (MMP-2) (TBE-1) [Cleaved into: PEX]
P10909	CLUS	x	Clusterin (Aging-associated gene 4 protein) (Apolipoprotein J) (Apo-J) (Complement cytolysis inhibitor) (CLI) (Complement-associated protein SP-40,40)
P81605	DCD	x	Dermcidin (EC 3.4.-.-) (Preproteolysin) [Cleaved into: Survival-promoting peptide; DCD-1]
Q14563	SEM3A	x	Semaphorin-3A (Semaphorin III) (Sema III)
P01034	CYTC	x	Cystatin-C (Cystatin-3) (Gamma-trace) (Neuroendocrine basic polypeptide) (Post-gamma-globulin)
Q9GZU2	PEG3	x	Paternally-expressed gene 3 protein (Zinc finger and SCAN domain-containing protein 24)
P04114	APOB	x	Apolipoprotein B-100 (Apo B-100) [Cleaved into: Apolipoprotein B-48 (Apo B-48)]
Q92752	TENR	x	Tenascin-R (TN-R) (Janusin) (Restrictin)
P24539	AT5F1	x	ATP synthase F(0) complex subunit B1, mitochondrial (ATP synthase proton-transporting mitochondrial F(0) complex subunit B1) (ATP synthase subunit b) (ATPase subunit b)
Q01484	ANK2	x	Ankyrin-2 (ANK-2) (Ankyrin-B) (Brain ankyrin) (Non-erythroid ankyrin)
P13591	NCAM1	x	Neural cell adhesion molecule 1 (N-CAM-1) (NCAM-1) (CD antigen CD56)
O94856	NFASC	x	Neurofascin
Q00169	PIPNA	x	Phosphatidylinositol transfer protein alpha isoform (PI-TP-alpha) (PtdIns transfer protein alpha) (PtdInsTP alpha)
P01834	IGKC	x	Immunoglobulin kappa constant (Ig kappa chain C region) (Ig kappa chain C region AG) (Ig kappa chain C region CUM) (Ig kappa chain C region EU) (Ig kappa chain C region OU)
P01857	IGHG1	x	Immunoglobulin heavy constant gamma 1 (Ig gamma-1 chain C region) (Ig gamma-1 chain C region EU) (Ig gamma-1 chain C region KOL) (Ig gamma-1 chain C region NIE)
P69905	HBA	x	Hemoglobin subunit alpha (Alpha-globin) (Hemoglobin alpha chain)
P02768	ALBU	x	Serum albumin
P00747	PLMN	x	Plasminogen (EC 3.4.21.7) [Cleaved into: Plasmin heavy chain A; Activation peptide; Angiostatin; Plasmin heavy chain A, short form; Plasmin light chain B]
P01023	A2MG	x	Alpha-2-macroglobulin (Alpha-2-M) (C3 and PZP-like alpha-2-macroglobulin domain-containing protein 5)
P02749	APOH	x	Beta-2-glycoprotein 1 (APC inhibitor) (Activated protein C-binding protein) (Anticardiolipin cofactor) (Apolipoprotein H) (Apo-H) (Beta-2-glycoprotein I) (B2GPI) (Beta(2)GPI)
P21246	PTN	x	Pleiotrophin (PTN) (Heparin-binding brain mitogen) (HBBM) (Heparin-binding growth factor 8) (HBGF-8) (Heparin-binding growth-associated molecule) (HB-GAM)
Q13315	ATM	x	Serine-protein kinase ATM (EC 2.7.11.1) (Ataxia telangiectasia mutated) (A-T mutated)
P24821	TENA	x	Tenascin (TN) (Cytotactin) (GMEM) (GP 150-225) (Glioma-associated-extracellular matrix antigen) (Hexabrachion) (JI) (Myotendinous antigen) (Neuronectin) (Tenascin-C) (TN-C)
Q8WZ42	TITIN	x	Titin (EC 2.7.11.1) (Connectin) (Rhabdomyosarcoma antigen MU-RMS-40.14)
P10253	LYAG	x	Lysosomal alpha-glucosidase (EC 3.2.1.20) (Acid maltase) (Aglucosidase alfa) [Cleaved into: 76 kDa lysosomal alpha-glucosidase; 70 kDa lysosomal alpha-glucosidase]
P16070	CD44	x	CD44 antigen (CDw44) (Epican) (Extracellular matrix receptor III) (ECMR-III) (GP90 lymphocyte homing/adhesion receptor) (HUTCH-I) (Heparan sulfate proteoglycan)
P04004	VTNC	x	Vitronectin (VN) (S-protein) (Serum-spreading factor) (V75) [Cleaved into: Vitronectin V65 subunit; Vitronectin V10 subunit; Somatomedin-B]
Q9HCB6	SPON1	x	Spondin-1 (F-spondin) (Vascular smooth muscle cell growth-promoting factor)
Q92831	KAT2B	x	Histone acetyltransferase KAT2B (EC 2.3.1.48) (Histone acetyltransferase PCAF) (Histone acetylase PCAF) (Lysine acetyltransferase 2B) (P300/CBP-associated factor) (P/CAF)
P02538	K2C6A	x	Keratin, type II cytoskeletal 6A (Cytokeratin-6A) (CK-6A) (Cytokeratin-6D) (CK-6D) (Keratin-6A) (K6A) (Type-II keratin Kb6) (allergen Hom s 5)
Q9NPR2	SEM4B	x	Semaphorin-4B
P40818	UBP8	x	Ubiquitin carboxyl-terminal hydrolase 8 (EC 3.4.19.12) (Deubiquitinating enzyme 8) (Ubiquitin isopeptidase Y) (hUBPy) (Ubiquitin thioesterase 8) (Ubiquitin-specific-processing protease 8)
Q8NFZ4	NLGN2	x	Neuroligin-2
P26006	ITA3	x	Integrin alpha-3 (CD49 antigen-like family member C) (FRP-2) (Galactoprotein B3) (GAPB3) (VLA-3 subunit alpha) (CD antigen CD49c) [Cleaved into: Integrin alpha-3 heavy chain; Integrin alpha-3 light chain]
P02790	HEMO	x	Hemopexin (Beta-1B-glycoprotein)
P06727	APOA4	x	Apolipoprotein A-IV (Apo-AIV) (ApoA-IV) (Apolipoprotein A4)
P02766	TTHY	x	Transthyretin (ATTR) (Prealbumin) (TBPA)
Q92547	TOPB1	x	DNA topoisomerase 2-binding protein 1 (DNA topoisomerase II-beta-binding protein 1) (TopBP1) (DNA topoisomerase II-binding protein 1)
P13646	K1C13	x	Keratin, type I cytoskeletal 13 (Cytokeratin-13) (CK-13) (Keratin-13) (K13)
P07858	CATB	x	Cathepsin B (EC 3.4.22.1) (APP secretase) (APPS) (Cathepsin B1) [Cleaved into: Cathepsin B light chain; Cathepsin B heavy chain]
P42262	GRIA2	x	Glutamate receptor 2 (GluR-2) (AMPA-selective glutamate receptor 2) (GluR-B) (GluR-K2) (Glutamate receptor ionotropic, AMPA 2) (GluA2)
P02655	APOC2	x	Apolipoprotein C-II (Apo-CII) (ApoC-II) (Apolipoprotein C2) [Cleaved into: Proapolipoprotein C-II (ProapoC-II)]
P02656	APOC3	x	Apolipoprotein C-III (Apo-CIII) (ApoC-III) (Apolipoprotein C3)
P02654	APOC1	x	Apolipoprotein C-I (Apo-CI) (ApoC-I) (Apolipoprotein C1) [Cleaved into: Truncated apolipoprotein C-I]
Q92743	HTRA1	x	Serine protease HTRA1 (EC 3.4.21.-) (High-temperature requirement A serine peptidase 1) (L56) (Serine protease 11)
Q13129	RLF	x	Zinc finger protein Rlf (Rearranged L-myc fusion gene protein) (Zn-15-related protein)
P00746	CFAD	x	Complement factor D (EC 3.4.21.46) (Adipsin) (C3 convertase activator) (Properdin factor D)

P00751	CFAB	x	Complement factor B (EC 3.4.21.47) (C3/C5 convertase) (Glycine-rich beta glycoprotein) (GBG) (PBF2) (Properdin factor B) [Cleaved into: Complement factor B Ba fragment; Complement factor B Bb fragment]
P08603	CFAH	x	Complement factor H (H factor 1)
Q9NZP8	C1RL	x	Complement C1r subcomponent-like protein (C1r-LP) (C1r-like protein) (EC 3.4.21.-) (C1r-like serine protease analog protein) (CLSPa)
P04264	K2C1	x	Keratin, type II cytoskeletal 1 (67 kDa cytokeratin) (Cytokeratin-1) (CK-1) (Hair alpha protein) (Keratin-1) (K1) (Type-II keratin Kb1)
P04259	K2C6B	x	Keratin, type II cytoskeletal 6B (Cytokeratin-6B) (CK-6B) (Keratin-6B) (K6B) (Type-II keratin Kb10)
P13647	K2C5	x	Keratin, type II cytoskeletal 5 (58 kDa cytokeratin) (Cytokeratin-5) (CK-5) (Keratin-5) (K5) (Type-II keratin Kb5)
P35527	K1C9	x	Keratin, type I cytoskeletal 9 (Cytokeratin-9) (CK-9) (Keratin-9) (K9)
P13645	K1C10	x	Keratin, type I cytoskeletal 10 (Cytokeratin-10) (CK-10) (Keratin-10) (K10)
A5YKK6	CNOT1	x	CCR4-NOT transcription complex subunit 1 (CCR4-associated factor 1) (Negative regulator of transcription subunit 1 homolog) (NOT1H) (hNOT1)
P55058	PLTP	x	Phospholipid transfer protein (Lipid transfer protein II)
Q9UPN3	MACF1	x	Microtubule-actin cross-linking factor 1, isoforms 1/2/3/5 (620 kDa actin-binding protein) (ABP620) (Actin cross-linking family protein 7) (Macrophin-1) (Trabeculin-alpha)
Q9NZN5	ARHGC	x	Rho guanine nucleotide exchange factor 12 (Leukemia-associated RhoGEF)
Q06481	APLP2	x	Amyloid-like protein 2 (APLP-2) (APPH) (Amyloid protein homolog) (CDEI box-binding protein) (CDEBP)
P19827	ITIH1	x	Inter-alpha-trypsin inhibitor heavy chain H1 (ITI heavy chain H1) (ITI-HC1) (Inter-alpha-inhibitor heavy chain 1) (Inter-alpha-trypsin inhibitor complex component III) (Serum-derived hyaluronan-associated protein) (SHAP)
Q13405	RM49	x	39S ribosomal protein L49, mitochondrial (L49mt) (MRP-L49) (Mitochondrial large ribosomal subunit protein mL49) (Neighbor of FAU) (NOF) (Protein NOF1)
Q9NZJ4	SACS	x	Sacsin (DnaJ homolog subfamily C member 29) (DNAJC29)
Q4V328	GRAP1	x	GRIP1-associated protein 1 (GRASP-1)
P14136	GFAP	x	Glial fibrillary acidic protein (GFAP)
Q7Z6G8	ANS1B	x	Ankyrin repeat and sterile alpha motif domain-containing protein 1B (Amyloid-beta protein intracellular domain-associated protein 1) (AIDA-1) (E2A-PBX1-associated protein) (EB-1)
P02774	VTDB	x	Vitamin D-binding protein (DBP) (VDB) (Gc protein-derived macrophage activating factor) (Gc-MAF) (GcMAF) (Gc-globulin) (Group-specific component) (Gc)
P41219	PERI	x	Peripherin (Neurofilament 4)
Q92628	K0232	x	Uncharacterized protein KIAA0232
Q9P2M1	LR2BP	x	LRP2-binding protein (Megalin-binding protein) (MegBP)
