# Supplementary material for: Systems pathology analysis identifies neurodegenerative nature of age‐related vitreoretinal interface diseases
Source: Aging Cell. 2018 Jul 2;17(5):e12809. doi: 10.1111/acel.12809 (PMC6156470; doi:10.1111/acel.12809)
Supplement: Supplementary file 11 [file ACEL-17-e12809-s011.pdf]

Supplemental Table S8: Normalized SWATH peak areas of the 404 quantified proteins.

Accession	Entry name	Protein names	iERM_1	iERM_14	iERM_17	iERM_20	iERM_26	MH_34	MH_36	MH_38	MH_45	MH_50	DME_59	DME_60	DME_61	DME_62	RRD_1	RRD_2
P05067	A4_HUMAN	Amyloid-beta A4 protein (ABPP) (APPI) (APP) (Alzheimer disease amyloid pro	89217,22	179342,3	342269,2	446984	323114,2	126887,6	298146,7	267518,4	234889	100496,2	60202,28	82051,54	109304,6	78872,96	140521	41657,06
P51693	APLP1_HUMAN	Amyloid-like protein 1 (APLP) (APLP-1) [Cleaved into: C30]	54111,89	75317,35	150124,2	84741,7	89962,52	39571,31	74759,78	88428,83	97585,64	33764,49	15316,16	23648,97	49293,95	71803,01	30688,45	15616,39
Q06481	APLP2_HUMAN	Amyloid-like protein 2 (APLP-2) (APPH) (Amyloid protein homolog) (CDEI box	157125,4	249807,8	642723,8	383157	325807,5	202382,7	383828,5	343033,6	277499,6	161725,6	72254,83	79933,76	102433,4	69866,97	227351,9	70543,38
P02649	APOE_HUMAN	Apolipoprotein E (Apo-E)	1228674	2342077	3575308	3429075	4548402	1747616	2324588	2573042	4251985	1421502	949442	1623965	1686791	1198053	1331768	899314,3
P19022	CADH2_HUMAN	Cadherin-2 (CDw325) (Neural cadherin) (N-cadherin) (CD antigen CD325)	29509,13	48133,6	75495,14	66552,83	67076,12	25317,77	69200,18	32101,68	54645,51	48655,37	43163	21004,38	21467,2	18059,51	28377,93	8461,308
O94985	CSTN1_HUMAN	Calsyntenin-1 (Alcadein-alpha) (Alc-alpha) (Alzheimer-related cadherin-like p	782302	888284	1457649	1257905	717080,9	738074,8	1409410	1408836	1132000	596753,4	241167,9	297182,5	293119,4	239342,5	873582,8	131068
P16870	CBPE_HUMAN	Carboxypeptidase E (CPE) (EC 3.4.17.10) (Carboxypeptidase H) (CPH) (Enkeph	251544,3	336915,5	502671,9	378507,1	281268,1	200626	549578	377310,9	277890,7	194953,5	110045,5	145019,1	120834,9	89013,36	296507,4	87425,12
Q9UBX1	CATF_HUMAN	Cathepsin F (CATSF) (EC 3.4.22.41)	32506,25	33263,84	53619,99	70237,83	41059,57	13267,22	34207,8	53331,2	39652,37	35606,57	31137,22	21076,03	17762,21	16543,75	31440,28	16313,13
P22304	IDS_HUMAN	Iduronate 2-sulfatase (EC 3.1.6.13) (Alpha-L-iduronate sulfate sulfatase) (Idu	2505,077	4040,386	7284,141	9603,94	7130,968	2681,403	1657,455	8872,575	5806,876	1239,236	1602,518	7073,716	3614,921	5062,147	8572,323	2576,634
Q9Y287	ITM2B_HUMAN	Integral membrane protein 2B (Immature BRI2) (imBRI2) (Protein E25B) (Tra	17414,21	11119,58	22840,05	28585,93	14127,83	9324,807	30909,12	8691,104	6666,815	11055,74	4046,904	12163,34	9575,715	32727,61	16003,65	19003,92
O00533	NCHL1_HUMAN	Neural cell adhesion molecule L1-like protein (Close homolog of L1) [Cleaved	20265,78	41640,24	68079,38	66285,02	42759,02	35339,8	46007,59	36840,64	36004,26	25229,64	15242,5	17395,92	17016,58	12067,24	20482,39	9647,168
O14594	NCAN_HUMAN	Neurocan core protein (Chondroitin sulfate proteoglycan 3)	28846,95	95532,76	104551,2	85483,97	69274,83	8564,586	81930,75	57162,91	43978,41	54191,44	8755,462	20912,94	73893,89	41025,72	16307,06	29948,29
Q92823	NRCAM_HUMAN	Neuronal cell adhesion molecule (Nr-CAM) (Neuronal surface protein Bravo)	44120,2	100339,7	128569,8	141663,9	82042,13	72135,01	102108,5	93934,39	93120,39	81978,32	69994,2	79648,71	100458,1	32996,57	64961,56	25757,35
O15240	VGF_HUMAN	Neurosecretory protein VGF [Cleaved into: Neuroendocrine regulatory pepti	37854,49	42951,53	60535,44	50778,05	59636,98	47453,63	76717,99	53421,02	62777,15	28539,54	53659,52	41354,79	27640,43	22411,09	58891,35	6374,481
Q99574	NEUS_HUMAN	Neuroserpin (Peptidase inhibitor 12) (PI-12) (Serpin I1)	17394,27	11067,74	22008,7	17165,06	13192,58	10592,93	11938,36	25275,46	16282,1	13822,76	5583,627	8879,188	6828,228	24673,79	21715,16	8337,055
P10451	OSTP_HUMAN	Osteopontin (Bone sialoprotein 1) (Nephropontin) (Secreted phosphoprotei	357423,1	1117701	1118223	1131200	966218,1	557392,7	1239430	878151,5	913519,3	386611,9	253031,8	276488,7	251723,8	239646,7	788615,7	105342,4
Q59EK9	RUN3A_HUMAN	RUN domain-containing protein 3A (Rap2-interacting protein 8) (RPIP-8)	15507,6	844,2815	34058,23	15551,98	31752,99	29678,55	34725,04	30797,32	1323,759	21507,27	16776,4	2745,871	20427,7	18626,63	12583,02	317,7448
Q8WXD2	SCG3_HUMAN	Secretogranin-3 (Secretogranin III) (SgIII)	63154,13	86888,72	164256,5	140068,3	96143,48	75321,56	117521,6	109582,9	98248	47448,95	35703,44	42566,32	44698,58	34821,28	67463,21	19300,15
Q08629	TICN1_HUMAN	Testican-1 (Protein SPOCK)	98566,67	205291,4	261672,2	319030,7	277266,9	144260,2	318444,2	269422,3	231895,2	146637	30496,67	54210,43	58220,84	24567,98	93401,83	23590,92
O14773	TPP1_HUMAN	Tripeptidyl-peptidase 1 (TPP-1) (EC 3.4.14.9) (Cell growth-inhibiting gene 1 p	100097,9	145174	170155,8	228558,1	193693	108099,7	259617,8	203611,5	185072,3	122156,8	84458,71	59769,24	51852,58	30443,32	143850,3	30112,85
Q9Y5W5	WIF1_HUMAN	Wnt inhibitory factor 1 (WIF-1)	134532,7	189931,3	300953,6	274942,7	105487,3	92684,82	205163,7	169754,7	127912,4	92088,33	47003,86	77024,08	43624,39	46329,06	159742,9	32066,9
P98164	LRP2_HUMAN	Low-density lipoprotein receptor-related protein 2 (LRP-2) (Glycoprotein 330	103086,1	103893,3	91721,21	78664,35	61876,75	81037,68	59608,56	103934,3	43996,97	150252,6	120707,3	114672,4	103903,6	129754,4	26162,78	18758,56
P16519	NEC2_HUMAN	Neuroendocrine convertase 2 (NEC 2) (EC 3.4.21.94) (KEX2-like endoprotease	9796,876	43362,26	444018,9	922122,9	858081,6	526825,2	14154,91	22645,89	21223,85	192675,9	36316,93	886994,4	893181,5	743963,7	399147,8	37779,37
Q53EL9	SEZ6_HUMAN	Seizure protein 6 homolog (SEZ-6) (hSEZ-6)	36278,67	18879,71	29098,9	18264,33	17722,66	21642,86	35511,7	32016,7	18448,72	18815,94	24691,97	26581,2	31548,54	6579,826	11879,68	21831,4
P63104	1433Z_HUMAN	14-3-3 protein zeta/delta (Protein kinase C inhibitor protein 1) (KCIP-1)	8551,628	3602,62	10459	10337,09	9386,638	1255,279	6597,522	959,979	3577,434	2168,321	16642,02	8834,388	12703,64	1558,296	21548,26	878,9617
Q9BRK5	CAB45_HUMAN	45 kDa calcium-binding protein (Cab45) (Stromal cell-derived factor 4) (SDF-4	313,657	9115,314	13073,14	2736,995	11713,29	2075,885	18302,25	15386,19	10580,27	7316,835	6017,235	337,4533	11404,32	3596,822	1347,449	1502,809
P08253	MMP2_HUMAN	72 kDa type IV collagenase (EC 3.4.24.24) (72 kDa gelatinase) (Gelatinase A) (	75642,61	35733,65	62638,72	29715,85	65054,01	22640	53628,82	42494,47	36249,11	34815,3	38715,47	25566,53	35858,24	68964,04	58525,39	49504,59
Q9UHI8	ATS1_HUMAN	A disintegrin and metalloproteinase with thrombospondin motifs 1 (ADAM-T	9314,234	3811,804	7863,58	5899,711	1676,869	1304,899	4345,291	5246,163	7211,386	289,4939	5743,282	2532,469	1710,478	2946,11	4899,181	5680,468
Q13510	ASAH1_HUMAN	Acid ceramidase (AC) (ACDase) (Acid CDase) (EC 3.5.1.23) (Acylsphingosine d	23664,3	51104,8	86307,36	83264,73	67768,37	31583,65	96991,83	49818,83	55210,94	51316,62	28262,26	9285,379	11896,28	38906,39	57945,33	19837,53
P63261	ACTG_HUMAN	Actin, cytoplasmic 2 (Gamma-actin) [Cleaved into: Actin, cytoplasmic 2, N-ter	93050,23	46586,98	66929,52	69861,29	82918,25	55750,49	59022,65	56181,36	37296,72	50625,24	257169,9	35640,54	69632,51	71895,26	441614,8	88732,01
P43652	AFAM_HUMAN	Afamin (Alpha-albumin) (Alpha-Alb)	481358	396982,5	334068,2	450867,9	499729,9	902842,8	512246,4	444617,3	383147,4	574560,5	381030,7	257411,8	742573,2	719958,4	354253,5	378694,6
O00468	AGRIN_HUMAN	Agrin [Cleaved into: Agrin N-terminal 110 kDa subunit; Agrin C-terminal 110	43714,62	43658,14	55702,86	60668,27	59222,57	24584,12	37473,88	25815,4	37751,19	45604,72	46624,63	15437,55	35245,55	24977,22	24720,03	33941,84
P26572	MGAT1_HUMAN	Alpha-1,3-mannosyl-glycoprotein 2-beta-N-acetylglucosaminyltransferase (E	3571,56	3281,768	6328,872	5594,096	5369,471	7012,34	3736,03	6230,818	5141,437	530,9766	264,134	1096,898	829,5074	818,0122	1837,27	750,7462
Q09328	MGT5A_HUMAN	Alpha-1,6-mannosylglycoprotein 6-beta-N-acetylglucosaminyltransferase A (	1183,998	5502,229	4575,526	2300,888	1291,655	1116,488	1619,342	1146,356	4395,712	1628,658	1326,408	423,2419	3630,609	1281,632	2763,83	1730,969
P02763	A1AG1_HUMAN	Alpha-1-acid glycoprotein 1 (AGP 1) (Orosomucoid-1) (OMD 1)	1169295	1823371	954565,7	1063351	4771656	4983627	3556787	3182869	6122577	6452433	9211406	4725713	7615467	6231808	4249222	6183386
P19652	A1AG2_HUMAN	Alpha-1-acid glycoprotein 2 (AGP 2) (Orosomucoid-2) (OMD 2)	105341,6	94325,52	67274,21	94348,45	529669,1	331286,4	388169,5	445656,3	452481	569113,2	663988,8	829913	1095519	1134898	747121,9	483540
P01011	AACT_HUMAN	Alpha-1-antichymotrypsin (ACT) (Cell growth-inhibiting gene 24/25 protein)	1858187	2071107	1274645	922658,8	1666796	2061317	1280972	770440,8	1629660	1703047	3784595	2331351	2314126	2686690	3603062	3755524
P01009	A1AT_HUMAN	Alpha-1-antitrypsin (Alpha-1 protease inhibitor) (Alpha-1-antiproteinase) (Se	534512,6	458859,7	544785,4	348759,9	652293,3	643270,8	442052,2	512102,2	494961,3	479592,6	579619,5	609854,4	657710,4	623370,3	340756,1	510562,5
P04217	A1BG_HUMAN	Alpha-1B-glycoprotein (Alpha-1-B glycoprotein)	731504,5	526078,8	669360,7	454552,9	686310,8	1116335	553397,9	579540,7	1047484	1149199	1074816	1018885	1314878	1258633	748051,9	855308,5
P08697	A2AP_HUMAN	Alpha-2-antiplasmin (Alpha-2-AP) (Alpha-2-plasmin inhibitor) (Alpha-2-PI) (Se	108037,4	85567,19	118004,2	53661,61	64499	157916,4	118612,4	118858,4	23568,43	68046,33	114444,8	126770,6	222946	112650,6	77325,14	130165,4
P02765	FETUA_HUMAN	Alpha-2-HS-glycoprotein (Alpha-2-Z-globulin) (Ba-alpha-2-glycoprotein) (Fetu	1917755	1665103	1600026	1283807	1929451	2921262	1163210	1950205	1548152	1940853	1726093	1438073	2632904	2132870	1333803	1804653
P01023	A2MG_HUMAN	Alpha-2-macroglobulin (Alpha-2-M) (C3 and PZP-like alpha-2-macroglobulin c	930122,7	844950	2324185	1942382	1683338	1317934	1985468	975862,1	1588773	1219668	907297,5	1609643	1254510	693101,9	2450226	610043,4
P49641	MA2A2_HUMAN	Alpha-mannosidase 2x (EC 3.2.1.114) (Alpha-mannosidase IIx) (Man IIx) (Mar	27717,29	17589,89	13626,8	16234,22	24138,65	7243,297	770,5629	795,4318	1769,773	1734,749	3407,555	2393,769	18055,72	2199,426	367,0705	1458,845
P17050	NAGAB_HUMAN	Alpha-N-acetylgalactosaminidase (EC 3.2.1.49) (Alpha-galactosidase B)	2731,801	11707,17	15422,76	11371,74	10915,93	4241,975	11380,15	10827,52	10471,06	10689,37	11444,02	13642,27	27475,42	20072,95	3657,262	16406,56
P37840	SYUA_HUMAN	Alpha-synuclein (Non-A beta component of AD amyloid) (Non-A4 componen	14208,84	567,9858	925,0562	2195,576	710,3258	3907,541	15942,85	896,1126	687,7956	631,4214	824,951	13667,42	10062,27	556,5825	47078,12	2581,604
P01019	ANGT_HUMAN	Angiotensinogen (Serpin A8) [Cleaved into: Angiotensin-1 (Angiotensin 1-10)	496496,4	526260,1	392370,4	466827,5	481580,4	471666,9	536608,8	375419,8	450621,7	700801,8	499288,3	569757,3	737274,3	792890	321103,5	368814,2
P01008	ANT3_HUMAN	Antithrombin-III (ATIII) (Serpin C1)	778669,3	530681,1	526412,9	344606,9	682052,4	666638,2	688785,9	736418,8	667627,9	1106687	818819,4	1210220	1155391	1230231	580926,1	1243753
P02647	APOA1_HUMAN	Apolipoprotein A-I (Apo-AI) (ApoA-I) (Apolipoprotein A1) [Cleaved into: Proa	3169229	2528183	1747526	1746924	2685426	1503517	1647022	2610241	4438032	3517246	3233746	3074453	3139463	2582169	2491460	4094293
P02652	APOA2_HUMAN	Apolipoprotein A-II (Apo-AII) (ApoA-II) (Apolipoprotein A2) [Cleaved into: Pr	975838,9	506459,2	489842,1	757130,9	881291,9	713060,8	692894,3	766610,1	774502,9	1496397	1158234	990964,5	1452431	917202,9	759851	1355534
P06727	APOA4_HUMAN	Apolipoprotein A-IV (Apo-AIV) (ApoA-IV) (Apolipoprotein A4)	1811154	1730681	1617649	668919,6	1231979	1725137	916360,7	1327882	1707206	1232358	1479024	1873586	2982921	3624239	608585,8	1335133
P02654	APOC1_HUMAN	Apolipoprotein C-I (Apo-CI) (ApoC-I) (Apolipoprotein C1) [Cleaved into: Trun	7739,621	4304,157	14418,77	7640,21	16907,5	94926,33	51634,8	64873,72	7969,049	16741,23	13904,68	50550,37	46853,43	11118,56	11099,91	24166,03
P02655	APOC2_HUMAN	Apolipoprotein C-II (Apo-CII) (ApoC-II) (Apolipoprotein C2) [Cleaved into: Pr	15177,92	5206,086	9210,327	9249,76	10879,53	1835,709	12949,26	6535,566	7469,589	2191,616	7499,611	45077,88	31246,46	22461,56	3750,419	29663,04
P02656	APOC3_HUMAN	Apolipoprotein C-III (Apo-CIII) (ApoC-III) (Apolipoprotein C3)	20216,64	14839,64	19649,79	6744,26	26482,98	23563,82	20858,51	19749,44	34215	29853,4	48363,56	96656,82	122634,4	102705,1	15196,92	33365,75

P05090	APOD_HUMAN	Apolipoprotein D (Apo-D) (ApoD)	7842,469	1435,176	833,0516	4258,408	1037,88	1667,224	4371,104	9977,334	6423,517	10816,47	1335,868	40097,99	4133,017	26172,9	6918,562	22110,34
Q13790	APOF_HUMAN	Apolipoprotein F (Apo-F) (Lipid transfer inhibitor protein) (LTIP)	5175,864	2213,482	894,7043	1103,676	4222,138	5148,14	952,7704	2557,29	3637,557	4527,959	1484,226	4625,589	6051,716	576,8174	2111,965	4793,116
O14791	APOL1_HUMAN	Apolipoprotein L1 (Apolipoprotein L) (Apo-L) (ApoL) (Apolipoprotein L-I) (Ap	52400,28	70109,01	8234,23	3491447	29755,38	36837,54	56715,26	66736,21	15300,17	29424,12	33990,03	27658,59	78395,95	6876,813	12088,85	47980,58
O95445	APOM_HUMAN	Apolipoprotein M (Apo-M) (ApoM) (Protein G3a)	10786,14	1381,808	6049,157	9078,775	5773,074	5361,261	12253,06	7078,241	5010,055	7708,22	6148,786	10294,93	12716,46	4701,91	9076,33	5913,317
P15848	ARSB_HUMAN	Arylsulfatase B (ASB) (EC 3.1.6.12) (N-acetylgalactosamine-4-sulfatase) (G4S)	1216,735	2130,032	2378,74	25410,88	349,7979	1602,713	3049,982	3394,513	625,1933	627,8934	619,8791	575,2791	2867,465	860,5642	3089,908	818,8916
Q12797	ASPH_HUMAN	Aspartyl/asparaginyl beta-hydroxylase (EC 1.14.11.16) (Aspartate beta-hydro	1780,68	2138,321	1747,329	6941,867	7690,449	822,5989	710,7017	958,7255	768,3079	540,3219	969,8121	712,2382	1341,693	986,0067	3068,392	2835,115
Q8NE71	ABCF1_HUMAN	ATP-binding cassette sub-family F member 1 (ATP-binding cassette 50) (TNF-	27900,74	20694,53	21181,83	11200,18	87854,12	115574,8	45353,61	122547,8	54791,5	66288,58	123779,6	140453	79363,23	81404,52	142468	189406,1
O75882	ATRN_HUMAN	Attractin (DPPT-L) (Mahogany homolog)	18271,28	6895,951	9023,743	9214,749	29236,71	22101,86	22738,43	24471,54	21628,08	22504,52	18858,74	15026,76	12229	6844,552	41666,88	15303,4
P02730	B3AT_HUMAN	Band 3 anion transport protein (Anion exchange protein 1) (AE 1) (Anion exc	1495,871	903,3319	1746,637	599,8696	1266,282	792,7528	1010,678	919,6291	759,3438	402,3704	815,2097	1289,748	1138,824	2144,3	2589,72	1084,405
P98160	PGBM_HUMAN	Basement membrane-specific heparan sulfate proteoglycan core protein (HS	49881,06	36601,42	46023,75	49481,01	34663,85	45940,2	50964,14	76419,29	31446,19	43344,99	22880,73	67043,12	37557,74	73775,25	47290,64	251245
P15291	B4GT1_HUMAN	Beta-1,4-galactosyltransferase 1 (Beta-1,4-GalTase 1) (Beta4Gal-T1) (b4Gal-T1	10023,44	26848,39	45508,14	31960,14	23993,38	19273,39	23715,13	14198,28	27435,54	10769,32	17207,11	7458,361	11906,6	13139,37	20905,3	7813,705
O43505	B4GA1_HUMAN	Beta-1,4-glucuronyltransferase 1 (EC 2.4.1.-) (I-beta-1,3-N-acetylglucosaminy	123669,4	179875,6	310555,1	271189,7	243836,3	132034,1	217522	192182,1	220259,8	110781,8	66292,47	72628,72	100649,7	20105,13	97949,26	35332,81
P02749	APOH_HUMAN	Beta-2-glycoprotein 1 (APC inhibitor) (Activated protein C-binding protein) (/	54672,26	47506,93	50111,39	29589,8	144368,9	100547,1	92137,6	96527,07	75082,19	76575,34	168879	192887,2	271970,6	234173,4	113025,5	98571
P61769	B2MG_HUMAN	Beta-2-microglobulin [Cleaved into: Beta-2-microglobulin form pI 5.3]	110984,2	76106,95	73312,63	64932,33	42431,22	54640,79	43489,46	46374,7	59476,52	50419,79	78851,85	49482,48	52629,51	53724,18	68917,39	22784,75
Q96KN2	CNDP1_HUMAN	Beta-Ala-His dipeptidase (EC 3.4.13.20) (CNDP dipeptidase 1) (Carnosine dipe	46140,76	88580,66	25490,51	57513,59	58608,64	49424,21	62047,49	27173,83	36757,57	42773,07	61720,63	42807,03	35593,36	58505,64	65904,38	19881,95
P43320	CRBB2_HUMAN	Beta-crystallin B2 (Beta-B2 crystallin) (Beta-crystallin Bp)	10442,55	979,805	10791,56	972,3953	2349,15	54160,44	2136,586	2244,961	2865,22	3568,923	25612,06	1145,967	1416,369	653,965	4888,082	2101,758
P22914	CRBS_HUMAN	Beta-crystallin S (Gamma-S-crystallin) (Gamma-crystallin S)	895,0742	4348,624	3200,173	1405,345	1110,126	2232,946	2694,815	6813,606	842,1724	3856,516	1122,859	6311,463	8833,778	6637,448	2306,175	549,2146
P06865	HEXA_HUMAN	Beta-hexosaminidase subunit alpha (EC 3.2.1.52) (Beta-N-acetylhexosaminid	14435,73	44072,58	55660,88	7815,389	41046,07	10851,12	10167,55	11178,84	12203,72	41821,49	27311,65	14800,93	13636,07	2752,513	23266,35	11018,38
P07686	HEXB_HUMAN	Beta-hexosaminidase subunit beta (EC 3.2.1.52) (Beta-N-acetylhexosaminida	6127,198	13820,82	16763,58	6663,342	20221,66	11110,24	9572,248	17176,52	10015,76	10532,49	21318,24	59189,94	24767,11	37938,11	11075,36	10827,94
O00462	MANBA_HUMAN	Beta-mannosidase (EC 3.2.1.25) (Lysosomal beta A mannosidase) (Mannanas	7622,171	4804,016	1976,64	3415,84	1646,157	733,2389	9057,062	3018,527	1873,419	690,2863	1810,775	543,8039	15076,3	11989,88	2695,406	1709,614
P43251	BTD_HUMAN	Biotinidase (Biotinase) (EC 3.5.1.12)	77236,93	117782,9	165627,9	179452,8	128499,3	71332,32	130973,6	143661,4	99189,03	86927,73	27998,42	40778,28	43501,38	33035,34	74777,77	25732,7
P07738	PMGE_HUMAN	Bisphosphoglycerate mutase (BPGM) (EC 5.4.2.4) (2,3-bisphosphoglycerate n	2269,603	2421,584	1789,343	1262,801	4595,416	5321,791	809,6936	3346,182	1816,367	405,7934	1877,161	7276,006	958,5958	1235,578	5150,395	829,482
Q96GW7	PGCB_HUMAN	Brevican core protein (Brain-enriched hyaluronan-binding protein) (BEHAB) (	9373,316	37902,15	32392,04	70572,59	72968,45	20962,55	14523,89	48245,96	24107,81	45598,23	8250,733	2597,58	11075,25	8117,33	17909,12	4992,673
Q8IZJ3	CPMD8_HUMAN	C3 and PZP-like alpha-2-macroglobulin domain-containing protein 8	82639,65	36295,88	44128,32	72607,68	93565,95	56538,58	76104,06	71883,03	32434,55	63296,05	52738,37	35126,94	75551,85	126611,2	114026,1	62690,32
P33151	CADH5_HUMAN	Cadherin-5 (7B4 antigen) (Vascular endothelial cadherin) (VE-cadherin) (CD a	5562,347	1921,385	5386,533	3559,221	9946,274	5442,445	6025,802	2844,902	4235,709	3317,462	2688,651	2135,435	4203,956	2328,291	2203,81	4813,97
Q96JP9	CDHR1_HUMAN	Cadherin-related family member 1 (Photoreceptor cadherin) (prCAD) (Protoc	8797,831	22673,2	40321,25	34577,83	23936,96	16676,16	30391,69	24859,37	24227,46	13317,34	29495,82	8996,136	5845,322	14615	24184,3	6936,17
Q9BQT9	CSTN3_HUMAN	Calsyntenin-3 (Alcadein-beta) (Alc-beta)	4601,305	9612,181	10567,66	10271,18	6458,363	5256,416	13287,66	6494,725	3582,311	3422,491	1450,876	3249,903	4135,421	4505,411	11271,89	2798,446
P00915	CAH1_HUMAN	Carbonic anhydrase 1 (EC 4.2.1.1) (Carbonate dehydratase I) (Carbonic anhyc	7932,041	3192,639	5871,622	3696,489	5970,044	8105,133	5228,104	3765,917	5870,983	4910,324	4962,881	4011,625	2780,295	8413,412	1417,639	3532,466
Q96IY4	CBPB2_HUMAN	Carboxypeptidase B2 (EC 3.4.17.20) (Carboxypeptidase U) (CPU) (Plasma carb	32621,06	32764,16	29976,19	36100,91	33569,84	33063,23	42267,37	20649,44	39704,25	53322,44	44068,29	83123,9	79158,03	91412,89	33729,76	89101,86
Q9Y646	CBPQ_HUMAN	Carboxypeptidase Q (EC 3.4.17.-) (Lysosomal dipeptidase) (Plasma glutamate	30019,81	42331,26	41851,08	36597,98	29197,35	20327,94	15667,43	29719,26	28710,52	17005,63	22404,73	44936,16	22131,81	79428,58	43147,69	10241,02
Q9NQ79	CRAC1_HUMAN	Cartilage acidic protein 1 (68 kDa chondrocyte-expressed protein) (CEP-68) (/	202001,6	260802,1	274907,8	285241,9	269635,3	185419,9	308767,3	214013,7	256386,3	169738,8	177468	100386,1	110918,4	140987,6	185483	52143,65
P04040	CATA_HUMAN	Catalase (EC 1.11.1.6)	8797,271	4611,401	5648,201	8433,07	21317,77	3254,114	25933,95	32221,1	6520,306	17285,78	25122,37	8985,957	11884,56	14491,5	10663,98	18248,95
P07858	CATB_HUMAN	Cathepsin B (EC 3.4.22.1) (APP secretase) (APPS) (Cathepsin B1) [Cleaved intc	17171,11	26885,42	18335,3	29217,53	5629,213	19933,48	34816,33	21210,74	27480,02	8000,972	20524,56	8157,816	10437,42	5571,137	41257,29	65369,43
P07339	CATD_HUMAN	Cathepsin D (EC 3.4.23.5) [Cleaved into: Cathepsin D light chain; Cathepsin D	1180218	2353406	3581953	2271017	1583647	1076262	1582067	1317973	1930133	905295	1658308	725830,5	754102	606734,4	2636725	670444,3
P07711	CATL1_HUMAN	Cathepsin L1 (EC 3.4.22.15) (Cathepsin L) (Major excreted protein) (MEP) [Cle	55680,11	75516,16	96829,81	95757,17	83351,93	45645,16	107537,3	75133,06	64180,51	51742,04	11422,88	21746,81	29617,5	2600,186	62635,39	28305,44
Q9UBR2	CATZ_HUMAN	Cathepsin Z (EC 3.4.18.1) (Cathepsin P) (Cathepsin X)	27209,33	34392,68	45824,2	43277,56	37856,04	20881,11	42060,69	28886,28	27974,39	29998,1	21566,64	15950,66	8468,981	16622,43	39418,1	13057,82
P16070	CD44_HUMAN	CD44 antigen (CDw44) (Epican) (Extracellular matrix receptor III) (ECMR-III) (	18387,2	11356,97	11617,61	7446,473	9397,605	7951,609	7791,637	5910,345	7199,024	7700,962	10295,37	7398,097	9140,692	4355,287	2229,215	3464,006
P13987	CD59_HUMAN	CD59 glycoprotein (1F5 antigen) (20 kDa homologous restriction factor) (HRF	2807,847	9700,951	12581,74	1131,584	1209,71	9948,726	16238,81	10991,75	930,7041	1283,335	7641,207	1459,087	4047,699	2038,66	28637,21	2670,072
Q9BY67	CADM1_HUMAN	Cell adhesion molecule 1 (Immunoglobulin superfamily member 4) (IgSF4) (N	17350,29	41470,42	14857,57	21607,73	8276,119	11092,5	42674,03	32642,43	9871,495	4617,753	17883,15	11659,06	37914,52	2570,036	22447,66	4598,725
Q8N3J6	CADM2_HUMAN	Cell adhesion molecule 2 (Immunoglobulin superfamily member 4D) (IgSF4D	4977,195	2405,677	5358,623	4425,474	1512,524	3609,509	4981,059	3879,504	1441,005	6986,09	3531,569	8466,405	11055,73	7488,886	9876,482	4907,621
O75503	CLN5_HUMAN	Ceroid-lipofuscinosis neuronal protein 5 (Protein CLN5) [Cleaved into: Ceroic	6141,773	1406,414	2842,482	7135,164	3908,161	3800,55	12994,71	4747,574	7129,473	4219,606	1952,225	4932,676	1398,414	1267,004	7544,779	1528,86
P00450	CERU_HUMAN	Ceruloplasmin (EC 1.16.3.1) (Ferroxidase)	3477302	4256197	3482948	2941965	2800123	3705337	2996924	1803111	3880044	3596939	3869671	3254898	2961568	2765053	4019697	2308430
P36222	CH3L1_HUMAN	Chitinase-3-like protein 1 (39 kDa synovial protein) (Cartilage glycoprotein 39	35287,07	47667,52	23073,77	51140,18	82697,35	146795,1	111114,1	71519,74	92631,22	60626,54	235279,3	137543,9	81504,02	96131,7	603986,1	60470,58
P10645	CMGA_HUMAN	Chromogranin-A (CgA) (Pituitary secretory protein I) (SP-I) [Cleaved into: Vas	18895,48	22474,71	44365,89	30522,49	34979,85	20272,01	34416,19	39206,93	22249,39	10569,18	8453,985	14878,15	9153,753	5641,708	7357,85	5918,831
P10909	CLUS_HUMAN	Clusterin (Aging-associated gene 4 protein) (Apolipoprotein J) (Apo-J) (Comp	7407181	13685137	14257299	13598963	9032702	6766444	7677165	7034295	8174261	5270162	8162849	4843137	4763098	4966302	5918712	3440021
Q15846	CLUL1_HUMAN	Clusterin-like protein 1 (Retinal-specific clusterin-like protein)	11484,96	20202,21	26961,68	29120,82	13805,64	17653,26	5156,852	30957,88	6306,771	5790,086	34576,09	21315,43	13068,74	14883,62	14413,59	9407,685
P00740	FA9_HUMAN	Coagulation factor IX (EC 3.4.21.22) (Christmas factor) (Plasma thromboplast	18197,12	12189,63	11435,78	8881,208	9122,127	14403,53	20088,64	18778,76	10696,62	13376,39	9507,586	25607,03	24981	23207,31	10059,7	10056,27
P12259	FA5_HUMAN	Coagulation factor V (Activated protein C cofactor) (Proaccelerin, labile facto	26471,37	19843,93	21813,16	17023,41	19852,14	15888,59	31246,06	22374,98	15807,07	24961,23	11702,5	12155,63	7215,23	5634,887	18020,79	21744,23
P00742	FA10_HUMAN	Coagulation factor X (EC 3.4.21.6) (Stuart factor) (Stuart-Prower factor) [Clea	642,4563	4485,008	2871,695	1648,135	758,26	1020,952	2769,749	4531,411	500,6069	6897,233	6254,252	1382,764	3002,781	3781,473	1941,607	1640,182
P00748	FA12_HUMAN	Coagulation factor XII (EC 3.4.21.38) (Hageman factor) (HAF) [Cleaved into: C	80891,74	37025,68	48802,04	22923,9	84418,31	92085,78	85643,74	57365,92	58719,78	119426,1	97338,48	67440,19	73360,32	55673,01	59731,24	58620,84
P23528	COF1_HUMAN	Cofilin-1 (18 kDa phosphoprotein) (p18) (Cofilin, non-muscle isoform)	1505,808	639,443	1529,102	3563,166	881,143	2634,742	4921,535	1063,179	650,5613	438,4303	4783,314	980,038	1140,897	1180,196	7655,941	1935,396
P02452	CO1A1_HUMAN	Collagen alpha-1(I) chain (Alpha-1 type I collagen)	16561,45	9147,662	11553,46	16768,72	40686,86	58325,71	6354,303	16630,59	36453,32	45649,1	29560,98	27606,73	24351,46	47050,09	23055,9	47018,06
P02458	CO2A1_HUMAN	Collagen alpha-1(II) chain (Alpha-1 type II collagen) [Cleaved into: Collagen al	20177	52037	24848,82	52511,16	27506,07	17987,88	45883,7	24135,92	34431,64	29432,44	9338,888	11189,17	19812,31	5367,884	12811,65	2901,185
P02461	CO3A1_HUMAN	Collagen alpha-1(III) chain	645,2684	2400,55	2836,255	2653,046	2307,196	727,566	3067,925	670,7909	1850,398	1287,226	1702,354	3413,771	4851,526	1943,35	1447,601	1328,665
P12109	CO6A1_HUMAN	Collagen alpha-1(VI) chain	20910,2	36286,41	64116,72	56886,23	46640,46	25969,18	53803,35	37818,97	37691,31	23799,4	15209,73	14839,04	17300,98	9173,959	19859,53	11244,18
P39060	COIA1_HUMAN	Collagen alpha-1(XVIII) chain [Cleaved into: Endostatin; Non-collagenous dor	35844,53	15956,14	111539,2	91455,32	17337	10057,1	22836,27	116017,1	15052,43	18534,71	11763,96	19002,04	17321,16	20135,24	10686,99	11693,84
P08123	CO1A2_HUMAN	Collagen alpha-2(I) chain (Alpha-2 type I collagen)	2302,529	6323,907	7581,396	6886,013	4903,689	2146,308	6378,617	3695,87	8019,401	6718,499	4912,845	9208,203	11863,59	2447,34	9141,183	2465,518
Q14055	CO9A2_HUMAN	Collagen alpha-2(IX) chain	22299,5	15434,82	1225,076	2171,678	51621,04	1406,279	6315,974	1946,591	49084,01	4913,128	4218,155	105490,3	3827,617	132503,8	4645,76	593,1637
Q5KU26	COL12_HUMAN	Collectin-12 (Collectin placenta protein 1) (CL-P1) (hCL-P1) (Nurse cell scaven	31745,69	1494,988	2060,571	9093,556	29691,8	6531,831	2156,216	14618,69	4556,948	10253,7	15618,42	24834,12	24570,26	1168,972	2472,18	17550

P02746	C1QB_HUMAN	Complement C1q subcomponent subunit B	24805,21	29794,26	15650,27	13549,65	16559,42	12729,75	4566,705	5788,389	14390,22	3652,754	15843,19	4443,705	15313,78	16856,72	24306,08	7094,131
P02747	C1QC_HUMAN	Complement C1q subcomponent subunit C	42795,65	75783,72	54212,01	27222,49	41143,7	32867,55	22241,32	22984,11	42030,56	28272,96	43921,1	48762,48	34993,41	24056,81	56325,13	23698,64
Q9BXJ4	C1QT3_HUMAN	Complement C1q tumor necrosis factor-related protein 3 (Collagenous repea	434,3331	33222,92	46844,67	2362,484	33543,12	21938,35	45178,85	31194,51	35183,87	22483,87	9797,791	14629,53	1999,896	12811,52	25172,22	14949,2
P00736	C1R_HUMAN	Complement C1r subcomponent (EC 3.4.21.41) (Complement component 1 :	60466,11	44280,69	57037,03	38248,15	75077,79	33516	60628,57	25831,18	16934,09	39839,4	58850,35	56019,45	32349,02	67755,32	90237,19	34181,16
Q9NZP8	C1RL_HUMAN	Complement C1r subcomponent-like protein (C1r-LP) (C1r-like protein) (EC 3	17045,1	15088,46	11116,57	4797,121	15793,87	19186,53	12110,37	5282,961	11996,11	16350,43	12522,35	11546,82	17855,19	22229,83	6027,182	14792,59
P09871	C1S_HUMAN	Complement C1s subcomponent (EC 3.4.21.42) (C1 esterase) (Complement c	133642,1	85638,44	76522,57	61122,09	46708,77	68799,08	97766,91	67143,17	80311,89	25584,41	68648,57	75330,22	115698,4	45322,92	105689,6	49084,82
P06681	CO2_HUMAN	Complement C2 (EC 3.4.21.43) (C3/C5 convertase) [Cleaved into: Compleme	29228,2	18933,88	21392,23	25195	51055,11	73975,84	59887,81	54033,6	50851,92	67034,82	35428,78	104107	104800,8	82580,16	28288,25	59110,34
P01024	CO3_HUMAN	Complement C3 (C3 and PZP-like alpha-2-macroglobulin domain-containing p	1839007	1986198	1677558	2024525	2658988	3070861	1808505	1725980	2983927	3035871	2261904	3370707	3423314	2446237	2604489	2409477
P0C0L4	CO4A_HUMAN	Complement C4-A (Acidic complement C4) (C3 and PZP-like alpha-2-macrogl	1637,861	411,7501	2034,936	0	304,709	1852,739	2684,689	443,2458	3226,419	619,6488	3273,386	1082,26	1104,094	6738,428	979,5168	2257,685
P0C0L5	CO4B_HUMAN	Complement C4-B (Basic complement C4) (C3 and PZP-like alpha-2-macroglo	163452	393645,8	313928	105083,9	213725,8	179505,9	146615,5	111358,5	269956,3	186273,3	164786,2	228822,4	222111,1	211597,7	251989,9	111907,1
P01031	CO5_HUMAN	Complement C5 (C3 and PZP-like alpha-2-macroglobulin domain-containing p	133837,7	90487,71	98711,94	59357,79	139347,4	118500,8	83631,4	82961,65	154000,8	153664,8	88769,91	158642,5	169516,2	141662,6	89219,69	100678,1
P13671	CO6_HUMAN	Complement component C6	34477,05	15950,1	14781,27	6148,681	24385,37	15270,32	24124,58	31283,88	32954,53	19285,79	20454,81	52509,58	40235,42	24960,47	13839,11	28233,31
P10643	CO7_HUMAN	Complement component C7	112126,9	61893,08	114908,8	32570,27	51879,28	186624,9	40900,43	24299,09	45506,72	47579,57	59108,24	126798	66272,06	65930,41	36606,64	44080,14
P07357	CO8A_HUMAN	Complement component C8 alpha chain (Complement component 8 subunit	68585,22	28211,1	71197,23	62099,03	111805,2	45639,48	66661,88	26243,77	59220,33	70120,69	66970,29	69301,29	50176,3	43962,23	51822,49	44110,4
P07358	CO8B_HUMAN	Complement component C8 beta chain (Complement component 8 subunit	90533,2	68172,04	57769,77	47479,06	62168,8	68364,33	63326,55	46217,5	105633,3	98981,57	96789,19	176058,2	99114,28	118473,6	57819,14	65334,27
P07360	CO8G_HUMAN	Complement component C8 gamma chain	25221,75	22030,62	22794,66	23693,66	45984,74	48506,14	29929,99	37171,93	73787,09	62063,6	53844,54	84877,95	74141,28	46756,11	28548,15	63662,04
P02748	CO9_HUMAN	Complement component C9 [Cleaved into: Complement component C9a; Cc	256486,5	213844,1	200871,5	121891,5	235874,4	231858,1	171845,6	146015,2	336700,9	265998	276369,5	317691,6	293166,2	363492,9	85544,31	222175,9
P00751	CFAB_HUMAN	Complement factor B (EC 3.4.21.47) (C3/C5 convertase) (Glycine-rich beta gl	728426,4	629273,8	678104,7	785592,5	1054658	970141,3	817465,2	621220,5	600374	878422	770424,3	954351,3	1066791	935978,2	815065,5	655707,1
P00746	CFAD_HUMAN	Complement factor D (EC 3.4.21.46) (Adipsin) (C3 convertase activator) (Prop	124846,7	51398,56	89076,23	34808,35	14943,54	94294,18	97197,2	43279,54	49190,08	53834,34	84122,31	64489,58	69128,13	105750,8	47272,05	29950,07
P08603	CFAH_HUMAN	Complement factor H (H factor 1)	74421,76	52469,59	106074,5	88182,12	151071,2	110989,5	111371,8	95354,28	150742,1	149072,7	101957,7	222112,1	196416,6	107472,7	113715,7	95992,49
Q03591	FHR1_HUMAN	Complement factor H-related protein 1 (FHR-1) (H factor-like protein 1) (H-fa	40390,66	43479,77	30951,08	36183,09	51017,88	57078,82	71135,19	52771,49	57573,78	51447,95	57509,55	54367,12	83486,31	69550,85	52788,76	19164,39
P05156	CFAI_HUMAN	Complement factor I (EC 3.4.21.45) (C3B/C4B inactivator) [Cleaved into: Con	255736,4	394075,7	322369,1	190864,6	236294,1	362949,6	291759	206343,1	350828	323459,4	296361,3	303762,7	368272,2	401924,3	436513,1	272029,7
Q12860	CNTN1_HUMAN	Contactin-1 (Glycoprotein gp135) (Neural cell surface protein F3)	37225,66	53340,78	94745,86	93632,22	71045,31	60440,73	57515,41	46967,53	38873,42	13410,92	14591,27	16070,31	20647,44	11635,35	28741,21	18411,05
Q02246	CNTN2_HUMAN	Contactin-2 (Axonal glycoprotein TAG-1) (Axonin-1) (Transient axonal glycopr	3768,456	6333,058	6082,918	13555,42	6590,713	7013,174	10026,6	3223,988	6071,775	5093,242	2236,883	2668,686	7902,582	2299,366	6012,968	7171,387
Q8IWV2	CNTN4_HUMAN	Contactin-4 (Brain-derived immunoglobulin superfamily protein 2) (BIG-2)	36988,67	8034,597	15230,21	16548,14	11841,68	9394,795	12551,66	10900,71	5361,403	4662,084	6916,29	5809,095	9520,526	6818,266	6648,274	3552,799
P08185	CBG_HUMAN	Corticosteroid-binding globulin (CBG) (Serpin A6) (Transcortin)	101756,1	77686,63	46839,5	29047,37	48600,54	34404,03	38959,94	42202,25	59529,37	68877,66	92520,39	100183,3	148617	121277,4	62662,67	86280,66
P02741	CRP_HUMAN	C-reactive protein [Cleaved into: C-reactive protein(1-205)]	11289,03	3609,335	1441,231	10041,21	7289,336	7156,529	2066,542	4891,494	589,9667	7881,149	5400,586	7252,342	1401,339	10804,25	600,1874	351,4131
P12277	KCRB_HUMAN	Creatine kinase B-type (EC 2.7.3.2) (Brain creatine kinase) (B-CK) (Creatine kir	1371,363	2404,963	10599,96	26829,62	57603,01	20335,34	29676,85	21835,17	8250,585	14179,94	5684,224	2853,413	15262,18	16745,79	20551,46	13959,3
P01034	CYTC_HUMAN	Cystatin-C (Cystatin-3) (Gamma-trace) (Neuroendocrine basic polypeptide) (I	1741987	3014485	4340117	1983011	3972581	1133969	2026321	3425274	3286132	1028258	2797746	1267460	1169946	1222595	4467213	491083,9
O00115	DNS2A_HUMAN	Deoxyribonuclease-2-alpha (EC 3.1.22.1) (Acid DNase) (Deoxyribonuclease II	6177,123	3281,836	14784,23	8820,075	13375,93	9093,973	21711,3	14669,64	9588,295	2045,914	1643,184	3297,688	3647,174	7756,755	9139,209	6753,8
Q9UBP4	DKK3_HUMAN	Dickkopf-related protein 3 (Dickkopf-3) (Dkk-3) (hDkk-3)	658401,1	1718851	1900332	2188234	1683443	779222,8	1643625	1290939	1575268	707186,9	562033,2	467831,1	581843,1	434845,5	922513,8	205325,6
Q01459	DIAC_HUMAN	Di-N-acetylchitobiase (EC 3.2.1.-)	8397,048	4530,763	5911,464	10581,35	6224,418	7708,003	7675,34	688,2209	3608,347	9728,143	4717,318	4583,249	4577,314	6087,239	7491,367	2809,591
Q9UHL4	DPP2_HUMAN	Dipeptidyl peptidase 2 (EC 3.4.14.2) (Dipeptidyl aminopeptidase II) (Dipeptic	27584,22	37748,96	44342,67	24071,47	59689,81	23880,82	34308,61	36843,13	13909,44	19133,55	11338,42	135937,5	22179	39733,25	44201,89	9358,327
P49917	DNLI4_HUMAN	DNA ligase 4 (EC 6.5.1.1) (DNA ligase IV) (Polydeoxyribonucleotide synthase		9669,801	722,8227	4437,98	1316,311	3630,816	4015,123	1372,942	4992,232	1756,175	1399,717	2956,111	5625,2	35546,65	23667,6	815,182	1380,687
Q9Y673	ALG5_HUMAN	Dolichyl-phosphate beta-glucosyltransferase (DolP-glucosyltransferase) (EC 2	28318,8	3492,529	7740,236	2941,384	846,1086	10014,69	13679,69	11206,69	15421,66	4987,235	13421,87	10874,13	29722,84	10866,9	6060,902	613,8644
Q14118	DAG1_HUMAN	Dystroglycan (Dystrophin-associated glycoprotein 1) [Cleaved into: Alpha-dy:	8543,881	17092,23	24147,88	3579,159	6003,76	5011,036	10439,16	3867,705	6520,684	3203,622	16855,08	32537,16	6183,557	5432,26	7863,688	12571,67
Q13822	ENPP2_HUMAN	Ectonucleotide pyrophosphatase/phosphodiesterase family member 2 (E-NF	369868,7	288196,5	267012,6	297650,7	186090,7	322858	504046,6	293947,7	205278,1	244201,2	416266,3	114343,1	127772,1	90395,74	368187,9	106998,1
Q12805	FBLN3_HUMAN	EGF-containing fibulin-like extracellular matrix protein 1 (Extracellular protei	44028,63	74519,04	96961,57	87380,23	150549,9	167031,5	173163,6	182965,3	319813	124847,4	114001,7	487106,3	125534,9	84756,94	189758	242725,8
P11021	BIP_HUMAN	Endoplasmic reticulum chaperone BiP (EC 3.6.4.10) (78 kDa glucose-regulate	6214,965	2010,468	1173,017	2379,203	362,1076	791,0896	883,0337	2336,038	2372,327	1117,771	936,4375	1871,707	1209,366	509,8229	1446,433	767,5097
Q16610	ECM1_HUMAN	Extracellular matrix protein 1 (Secretory component p85)	86766,25	37064,08	47940,72	18899,58	50634,98	59961,18	43455,43	77787,94	37887,61	44628,75	16404,74	35950,69	77757,89	32472,56	59033,72	50869,15
Q8IWU5	SULF2_HUMAN	Extracellular sulfatase Sulf-2 (hSulf-2) (EC 3.1.6.-)	515,3795	2932,631	1229,912	2604,129	1223,349	2279,219	767,0781	5479,825	1204,552	1691,829	1345,745	791,2771	5554,089	2637,941	2629,746	1433,032
P08294	SODE_HUMAN	Extracellular superoxide dismutase [Cu-Zn] (EC-SOD) (EC 1.15.1.1)	28895,22	70739,29	54368,09	36557,58	42092,57	35950,86	57835,74	53517,71	31782,17	30163,12	33305,08	9267,543	8635,6	11065,16	56521,97	27101,31
P35555	FBN1_HUMAN	Fibrillin-1 [Cleaved into: Asprosin]	4691,251	8500,882	7172,975	5282,482	4707,992	17262,55	32140,95	16890,09	5242,567	56090,34	13773,95	6003,968	8573,548	7400,933	11566,84	7913,956
P02671	FIBA_HUMAN	Fibrinogen alpha chain [Cleaved into: Fibrinopeptide A; Fibrinogen alpha cha	101638,1	68109,29	339804,5	161016,1	274268,4	237883,7	89817,84	143489,9	246063,4	130257,9	92661,84	1516469	927958,8	329891,9	82580,78	209438,5
P02675	FIBB_HUMAN	Fibrinogen beta chain [Cleaved into: Fibrinopeptide B; Fibrinogen beta chain	188376,4	126813,5	191036,2	165516,7	792876,9	353662	181576,7	240910,4	607569,9	362795,1	132105,8	2423169	1661881	422076,5	65032,47	285559,1
P02679	FIBG_HUMAN	Fibrinogen gamma chain	137078,4	72546,52	179185,1	101737,1	343666,8	213448,2	128943,5	167149,3	379036,9	242451,4	148963,3	1541940	1031893	366653,7	60223,05	252165,4
P02751	FINC_HUMAN	Fibronectin (FN) (Cold-insoluble globulin) (CIG) [Cleaved into: Anastellin; Ugl	120511,2	89190,07	454269,1	139764,7	167895,7	115590,7	122294,6	107352,1	214488,6	115001,8	172457,4	189076,1	240246,5	134764,1	102063,9	78971,2
P23142	FBLN1_HUMAN	Fibulin-1 (FIBL-1)	108784,9	79566,42	124890	102393,6	97046,23	73907,3	124004,8	119176,6	115058,2	79127,96	41681,33	47163,97	50544,87	44297,1	83086,3	46223,77
P30043	BLVRB_HUMAN	Flavin reductase (NADPH) (FR) (EC 1.5.1.30) (Biliverdin reductase B) (BVR-B) (	559,7715	215,8747	1055,625	2035,306	475,4883	362,9154	1258,854	638,4121	603,5279	1051,808	636,0067	1268,914	1159,257	572,0999	21289	716,3904
Q12841	FSTL1_HUMAN	Follistatin-related protein 1 (Follistatin-like protein 1)	49467,94	129964,2	158566,9	76694,62	68977,73	62186,02	243425,5	85760,33	64038,28	35005,48	29816,99	32404,06	40166,44	35182,32	139282,6	16358,63
Q6MZW2	FSTL4_HUMAN	Follistatin-related protein 4 (Follistatin-like protein 4)	16572,95	12484,89	16780,05	31641,73	11411,68	5865,52	14636,25	26291,58	18002,25	16372,68	6446,774	11943,93	9209,862	7437,243	17142,53	3379,942
Q8N475	FSTL5_HUMAN	Follistatin-related protein 5 (Follistatin-like protein 5)	52695,24	74446,57	116368,2	111046,2	86655,67	41104,01	84235,54	90860,15	84931,17	51436,92	36919	51090,29	70143,4	29475,66	93867,15	18118,84
P09972	ALDOC_HUMAN	Fructose-bisphosphate aldolase C (EC 4.1.2.13) (Brain-type aldolase)	1388,748	570,5771	2963,282	899,6323	986,2134	568,1296	4698,701	2354,078	3346,745	2371,836	4347,543	528,2951	1647,726	10258,53	6330,848	1289,4
Q08380	LG3BP_HUMAN	Galectin-3-binding protein (Basement membrane autoantigen p105) (Lectin	77701,93	72988,58	127728,6	157196,4	77582,5	63518,77	117649,3	155323,8	55234,82	88452,39	61438,56	27888,31	18618,28	23577,17	262470,1	33710,41
P09104	ENOG_HUMAN	Gamma-enolase (EC 4.2.1.11) (2-phospho-D-glycerate hydro-lyase) (Enolase	14125,18	4462,882	7413,36	10604,09	8759,93	10271,94	31399,73	20571,27	15885,26	50537,2	68579,8	45449,48	49505,8	15139,03	70713,84	4933,588
P17900	SAP3_HUMAN	Ganglioside GM2 activator (Cerebroside sulfate activator protein) (GM2-AP)	25303,04	17289,96	31959,08	28985,5	26012,3	15318,64	70198,44	37284,69	22623,94	40426,3	39246,87	8468,927	12221,03	8910,932	8282,045	8816,428
P06396	GELS_HUMAN	Gelsolin (AGEL) (Actin-depolymerizing factor) (ADF) (Brevin)	1238192	1117762	1182804	582100,4	833271,1	771372,8	1018082	1221382	1185123	882011,7	936264	1048438	858400,4	802346	832963	591131,6
P48058	GRIA4_HUMAN	Glutamate receptor 4 (GluR-4) (GluR4) (AMPA-selective glutamate receptor 4	2546,968	9690,862	14210,61	15409,17	9360,676	5893,834	5733,227	9816,58	10720,85	5823,184	2919,888	2696,087	4849,506	6912,433	7411,692	4270,646
Q16769	QPCT_HUMAN	Glutaminyl-peptide cyclotransferase (EC 2.3.2.5) (Glutaminyl cyclase) (QC) (s	19668,46	14665,62	24945,93	34801,07	17093,92	8818,394	44418,42	24236,65	19513,96	22378,61	8736,172	4814,419	5731,569	1936,7	15258,58	2901,508

P22352	GPX3_HUMAN	Glutathione peroxidase 3 (GPx-3) (GSHPx-3) (EC 1.11.1.9) (Extracellular glutat	2384971	3223748	3868479	4167758	2432886	2231665	4579135	2419216	2728130	1753548	1197443	1175927	1115346	1240412	2343002	867748
P04406	G3P_HUMAN	Glyceraldehyde-3-phosphate dehydrogenase (GAPDH) (EC 1.2.1.12) (Peptidy	11895,05	9791,059	20532,82	16750,34	13215,27	15759,25	13514,73	16459,16	16006,92	12636,06	15200,41	4456,338	10521,01	4637,426	28005,91	4117,504
Q14393	GAS6_HUMAN	Growth arrest-specific protein 6 (GAS-6) (AXL receptor tyrosine kinase ligand	1526,643	681,6108	629,6161	1412,598	1019,933	2975,774	2716,677	4082,402	2479,471	1175,821	652,8001	1984,95	1582,199	4835,746	1305,451	2902,333
O14793	GDF8_HUMAN	Growth/differentiation factor 8 (GDF-8) (Myostatin)	754,1396	1768,725	666,4101	551,5671	3032,395	924,6872	11159,72	1483,598	655,9314	3703,445	789,6066	2415,96	5844,341	3771,507	1289,392	3403,423
P62873	GBB1_HUMAN	Guanine nucleotide-binding protein G(I)/G(S)/G(T) subunit beta-1 (Transduci	8942,507	12300,13	14649,53	10909,42	8962,116	5159,634	18552,28	17794,2	6751,313	9631,472	16831,59	13001,92	10978,06	27486,8	513739,1	20628,5
P11488	GNAT1_HUMAN	Guanine nucleotide-binding protein G(t) subunit alpha-1 (Transducin alpha-1	7505,434	3871,43	5645,093	3320,053	4763,011	6051,784	3596,273	3050,405	5110,605	5021,347	13990,62	6969,442	4035,054	10288,38	229431,1	21533,63
P63211	GBG1_HUMAN	Guanine nucleotide-binding protein G(T) subunit gamma-T1 (Transducin gam	21696,95	2647,329	27054,12	9340,402	24421,45	5774,976	19008,23	12181,52	15426,5	14783,62	24633,29	24645,57	21590,4	18859,17	182791,2	6243,106
P00738	HPT_HUMAN	Haptoglobin (Zonulin) [Cleaved into: Haptoglobin alpha chain; Haptoglobin b	69656,59	70046,44	135228,1	707357,2	1340421	121972	125269,3	433913	112222,8	885199,6	223122,5	1476008	1580415	146411,4	256659	1153680
P00739	HPTR_HUMAN	Haptoglobin-related protein	5021,214	11948,82	1292,697	4875,567	79958,63	3946,861	5113,506	16100,07	6522,024	16496,83	5778,647	56003,53	62768,94	2742,264	21439,99	253944,8
P48723	HSP13_HUMAN	Heat shock 70 kDa protein 13 (Microsomal stress-70 protein ATPase core) (St	31334	30161,97	25336,66	32042,21	27986,52	14274,91	30750,38	25176,32	22391,65	5902,986	8030,596	19115,24	21368,97	9050,306	44537,16	80293,87
P69905	HBA_HUMAN	Hemoglobin subunit alpha (Alpha-globin) (Hemoglobin alpha chain)	9916,897	1925,459	9966,291	2738,468	18936,43	7867,286	4193,362	2919,557	2077,453	2079,054	93835,38	8438,088	2295,013	95398,52	2737,771	1135,336
P68871	HBB_HUMAN	Hemoglobin subunit beta (Beta-globin) (Hemoglobin beta chain) [Cleaved int	15954,11	6507,379	7565,49	8918,822	8615,887	3906,489	17457,26	4508,318	12315,38	10424,5	956197,5	6918,257	15795,49	1235710	11613,83	3083,006
P02042	HBD_HUMAN	Hemoglobin subunit delta (Delta-globin) (Hemoglobin delta chain)	29448,34	4350,969	2372,852	9173,253	6725,096	9923,449	8596,473	6119,03	7269,774	14288,93	19468,57	9835,22	3007,278	32353,06	27991,65	1243,808
P02790	HEMO_HUMAN	Hemopexin (Beta-1B-glycoprotein)	6503568	6891893	5445107	5240722	6677106	9691351	7287674	6621120	7780878	10470721	6523479	7072243	9163517	9081171	3873292	4734406
P05546	HEP2_HUMAN	Heparin cofactor 2 (Heparin cofactor II) (HC-II) (Protease inhibitor leuserpin-2	193230,2	260807,4	228222,3	143820,5	261784,1	360838,7	240348	155511,1	227646,3	313899,8	130087,6	279021,6	328606,1	303163,5	119445,5	304517,1
P04196	HRG_HUMAN	Histidine-rich glycoprotein (Histidine-proline-rich glycoprotein) (HPRG)	278008,3	348902,2	340050	295258,9	346076,4	543433,3	694391,4	325492,2	398484,4	321896,7	633363,4	641577,8	546724,9	597267,8	248499,8	455474,7
Q9BTM1	H2AJ_HUMAN	Histone H2A.J (H2a/j)	4373,37	1015,647	9292,784	1073,99	20492,41	3406,137	672,6523	4908,165	3053,272	1047,913	9523,463	2623,404	5588,956	4377,358	31401,22	371,0008
Q14520	HABP2_HUMAN	Hyaluronan-binding protein 2 (EC 3.4.21.-) (Factor VII-activating protease) (F	53824,49	15501,78	13462,39	10114,91	24452,53	28388,59	24273,87	22997,77	20614,75	19220,76	23641,43	33915,93	52720,72	46376,95	22881,42	25996,84
Q9Y6R7	FCGBP_HUMAN	IgGFc-binding protein (Fcgamma-binding protein antigen) (FcgammaBP)	305342,7	556165,1	277183,1	221607,1	352098,1	311301	185952	163306,1	323715,3	276165,4	318763,1	155861,3	359972,6	196700,4	242267,7	174676,2
P0DOX2	IGA2_HUMAN	Immunoglobulin alpha-2 heavy chain (Immunoglobulin alpha-2 heavy chain I	7678,228	3465,865	1364,375	3563,786	11062,03	17899,35	18462,87	8966,496	19997,16	1981,411	19381,62	95621,79	42355,2	3147,481	7541,257	7891,84
P0DOX3	IGD_HUMAN	Immunoglobulin delta heavy chain (Immunoglobulin delta heavy chain WAH	15045,42	13244,45	14675,78	26016,04	19616,27	3139,194	13721,77	11436,06	14351,61	15601,89	11290,39	12815,77	20536,76	17178,53	2832,962	2947,071
P0DOX4	IGE_HUMAN	Immunoglobulin epsilon heavy chain (Immunoglobulin epsilon heavy chain N	14608,49	1804,49	3708,868	8960,328	3501,315	30690,94	26002,07	43475,91	22965,58	18414,27	14599,7	11384,43	13789,98	10634,32	15525,42	30269,8
P0DOX5	IGG1_HUMAN	Immunoglobulin gamma-1 heavy chain (Immunoglobulin gamma-1 heavy cha	178681,4	7571,743	5914,766	44274,86	11672,77	8518,735	9552,185	15325,07	10391,38	10711,39	6040,959	5204,204	5911,869	15725,09	10744,67	11878,99
P01876	IGHA1_HUMAN	Immunoglobulin heavy constant alpha 1 (Ig alpha-1 chain C region) (Ig alpha-	2056563	182012,7	554060,9	369662,2	839383	1378689	583968,7	539084,5	1309642	274557,5	533115,8	1493730	675216,4	323919,6	97985,96	393190,2
P01859	IGHG2_HUMAN	Immunoglobulin heavy constant gamma 2 (Ig gamma-2 chain C region) (Ig ga	1237952	882095,3	814068,9	1076373	1298011	3037869	2846160	3712412	6127398	3536337	2605174	1921661	1679516	1385088	2891881	7833786
P01860	IGHG3_HUMAN	Immunoglobulin heavy constant gamma 3 (HDC) (Heavy chain disease protei	2009,354	4831,244	1514,843	5866,702	1935,64	1669,687	2753,4	5760,139	6592,016	5958,959	5967,159	13618,31	6789,65	5641,494	8717,532	4456,555
P01861	IGHG4_HUMAN	Immunoglobulin heavy constant gamma 4 (Ig gamma-4 chain C region)	88294,48	16055,35	52310,85	30666,63	368119,2	33317,76	363417,4	91679,3	231715,4	77881,21	30668,11	211297,8	63865,83	27726,53	68232,52	204716
P01871	IGHM_HUMAN	Immunoglobulin heavy constant mu (Ig mu chain C region) (Ig mu chain C re	4349,255	9394,748	31980,58	7275,179	4533,497	10286,04	6733,015	12935,14	4649,873	10623,72	5519,119	28477,79	14343,89	9318,739	11951,17	20946,42
A0A0B4J1	HV315_HUMAN	Immunoglobulin heavy variable 3-15	29606,21	31861,01	27624,48	34964,06	5021,405	53102,07	64786,65	62145,19	108670,7	57223,14	33109,46	70139,23	63113,7	65821,05	82271,61	180801,7
P0DP03	HV335_HUMAN	Immunoglobulin heavy variable 3-30-5	4573,345	3156,887	1641,796	3516,368	3518,743	6979,54	3158,599	5865,431	4475,019	4789,536	1846,356	4933,676	3707,538	3855,023	1200,85	12309,9
A0A0A0M	HV349_HUMAN	Immunoglobulin heavy variable 3-49	5755,449	4227,784	15436,67	3961,738	7079,353	9121,829	18807,6	13786,24	16801,11	10059,53	9672,087	18968,25	10719	8360,063	29471,69	31010,94
A0A0J9YX	HV64D_HUMAN	Immunoglobulin heavy variable 3-64D	23262,3	25495,08	34207,94	14892,11	27468,87	2872,85	91293,81	18861,34	9346,159	19198,36	13424,88	21376,66	87780,03	12905,33	32675,52	28417,55
P01780	HV307_HUMAN	Immunoglobulin heavy variable 3-7 (Ig heavy chain V-III region GAL) (Ig heav	3076,359	1414,684	898,2537	870,8054	4088,492	317,0914	2196,163	472,2801	6669,994	4230,474	3208,82	2094,416	2760,137	1415,917	4270,167	15593,7
A0A0J9YX	HV5X1_HUMAN	Immunoglobulin heavy variable 5-10-1	9363,675	2671,124	3329,106	5938,052	8209,755	5623,094	9584,451	8233,072	9322,297	3200,838	4345,863	6513,736	3318,805	1826,695	17404,16	1868,166
P01834	IGKC_HUMAN	Immunoglobulin kappa constant (Ig kappa chain C region) (Ig kappa chain C r	439305,9	62408,5	92417,8	39199,46	81051,93	97357,69	106310	105857,5	153127,4	119088,3	68552,39	136773,4	144073,5	106733,1	92481,93	170370,6
P0DOX7	IGK_HUMAN	Immunoglobulin kappa light chain (Immunoglobulin kappa light chain EU)	153387,3	19712,19	26577,25	3648,446	10775,2	54690,07	57353,94	56725,62	61759,21	48983,67	35556,82	42129,35	86824,69	59567,63	42828,81	76954,13
P01602	KV105_HUMAN	Immunoglobulin kappa variable 1-5 (Ig kappa chain V-I region CAR) (Ig kappa	154802,7	4411,186	7047,728	57831,02	18478,39	6389,251	4464,575	2618,737	92488,71	14862,82	200877,2	300135,9	23802,55	29142,21	9607,964	32588,08
A0A0C4DH	KV106_HUMAN	Immunoglobulin kappa variable 1-6	1762,385	3981,403	1443,541	4019,542	866,3174	5794,412	2897,488	1436,913	2000,024	1872,427	5613,515	7512,952	1510,023	2549,596	18069,06	6159,467
A0A0C4DH	KV109_HUMAN	Immunoglobulin kappa variable 1-9	4354,506	1099,472	1801,7	1088,182	1884,893	7018,892	1278,429	770,5823	4090,314	1511,16	2070,562	400,7036	4419,492	610,5963	8050,788	15289,12
A0A0C4DH	KV224_HUMAN	Immunoglobulin kappa variable 2-24	1692,733	3830,539	1068,817	3748,808	1145,839	1620,339	4998,566	2908,084	4151,906	9494,016	1071,764	1645,213	2527,672	1172,848	9862,11	19671,68
P04433	KV311_HUMAN	Immunoglobulin kappa variable 3-11 (Ig kappa chain V-III region VG)	528960,8	72000,71	52044,13	72408,08	94901,32	75561,88	74132,25	124293,1	119847,7	290147,5	45378,26	70190,42	67842,75	34189,48	120367	188416
P01619	KV320_HUMAN	Immunoglobulin kappa variable 3-20 (Ig kappa chain V-III region B6) (Ig kapp	137500,2	50558,13	53493,77	91065,04	106188,4	94449,07	61793,78	95784,17	129621,9	61828,2	63201,83	74813,88	84408,8	49720,48	110488,1	155022,2
A0A0C4DH	KVD20_HUMAN	Immunoglobulin kappa variable 3D-20	27455,82	5052,683	614,1477	12077,97	11985,78	9089,96	7544,087	24408,41	15535,86	20596,7	413,0986	14345,19	15255,26	8083,104	12278,09	37158,43
P06312	KV401_HUMAN	Immunoglobulin kappa variable 4-1 (Ig kappa chain V-IV region B17) (Ig kapp	616,4422	306,8179	1852,437	2148,074	871,5199	686,8947	511,3854	3021,024	2228,77	1399,252	1659,921	2861,674	3374,267	3221,086	4976,287	4037,222
P0DOY3	IGLC3_HUMAN	Immunoglobulin lambda constant 3 (Ig lambda chain C region DOT) (Ig lambo	7846,17	1847,457	1176,344	2003,007	3332,46	1746,449	3235,353	2113,78	3334,141	2086,812	3749,149	3664,447	3083,614	3578,814	1936,713	2291,914
A0M8Q6	IGLC7_HUMAN	Immunoglobulin lambda constant 7 (Ig lambda-7 chain C region)	10137,46	7579,377	7586,293	6178,694	2845,928	1184,814	44203,83	48674,21	404,9618	305,7963	958,1607	383,7069	78081,01	95947,35	34884,26	3485,119
P01700	LV147_HUMAN	Immunoglobulin lambda variable 1-47 (Ig lambda chain V-I region HA) (Ig lam	82001,97	12702,1	10584,19	16134,59	19547,94	26957,9	20500,99	27452,74	34712,56	18027,87	21101,26	28745,29	32886,46	11099,47	46540,23	16386,7
P01701	LV151_HUMAN	Immunoglobulin lambda variable 1-51 (Ig lambda chain V-I region BL2) (Ig lar	4650,582	3119,042	2295,549	4359,398	3649,243	7063,998	4139,987	630,9666	6420,263	2693,364	21184,36	4077,052	28529,42	5623,567	5116,745	7460,455
A0A075B6	LV218_HUMAN	Immunoglobulin lambda variable 2-18	11982,78	1124,578	322,4173	18303,6	6378,402	6122,434	1576,747	447,5031	639,3951	305,7881	3433,682	1499,047	1278,314	426,3944	1590,109	3914,166
P80748	LV321_HUMAN	Immunoglobulin lambda variable 3-21 (Ig lambda chain V-III region LOI) (Ig la	16608,69	13352,57	8393,965	1536,329	17030,75	12175,07	5182,421	9030,337	6192,073	6740,71	8453,782	16240,86	18096,46	8634,469	22178,82	15891,71
P01718	LV327_HUMAN	Immunoglobulin lambda variable 3-27 (Ig lambda chain V-IV region Kern)	3301,168	5519,717	2468,196	7260,509	3880,502	6066,038	4869,633	3923,876	4516,99	4762,47	4794,226	5912,498	13204,17	2653,713	7001,387	7102,816
A0A075B6	LV861_HUMAN	Immunoglobulin lambda variable 8-61	941,3841	3848,992	525,8594	4558,863	3766,281	1201,276	5758,042	1493,271	8869,068	2841,967	2626,656	4365,996	4516,364	1213,25	4426,297	6782,544
P0DOX8	IGL1_HUMAN	Immunoglobulin lambda-1 light chain (Immunoglobulin lambda-1 light chain	450442,1	77343,69	106184	182543,6	179618,5	292555	219736,8	226582,4	267412,6	152321,1	141457,3	147380,9	138620,7	129150	296796,2	452760,8
O14498	ISLR_HUMAN	Immunoglobulin superfamily containing leucine-rich repeat protein	7744,564	4767,998	3701,214	4596,568	1473,863	3854,004	7363,941	2386,477	2864,101	1858,433	6104,153	3830,731	2091,889	3112,111	1204,425	937,6078
Q8N3Z0	PRS35_HUMAN	Inactive serine protease 35	10582,99	7352,915	11262,83	6976,622	1908,177	6614,6	10161,39	7451,199	10815,1	1380,907	4225,369	3616,092	5513,329	5775,262	12412,33	2096,564
P18065	IBP2_HUMAN	Insulin-like growth factor-binding protein 2 (IBP-2) (IGF-binding protein 2) (I(	6738,613	8246,504	13367,83	6235,174	4793,42	7784,677	4818,813	4484,188	4129,285	2252,903	4984,542	10236,19	6386,201	12696,43	3892,953	5730,064
P22692	IBP4_HUMAN	Insulin-like growth factor-binding protein 4 (IBP-4) (IGF-binding protein 4) (I(	11630,73	8478,63	16215,93	16402,39	1679,09	12440,3	3707,417	10337,64	8724,837	1671,219	4659,71	5548,574	1663,334	851,7432	1917,652	384,9664
P24593	IBP5_HUMAN	Insulin-like growth factor-binding protein 5 (IBP-5) (IGF-binding protein 5) (I(	8141,982	7386,684	2154,968	15772,5	2908,179	6121,322	11367,13	8326,453	5517,793	2192,273	2432,984	1370,129	11731,4	3482,665	3935,384	1384,753
P24592	IBP6_HUMAN	Insulin-like growth factor-binding protein 6 (IBP-6) (IGF-binding protein 6) (I(	111187,7	69281,87	100148,5	76620,86	57025,31	72195,53	112061,8	109312,8	67473,27	59960,54	76395,85	51283,25	44259,96	40308,93	120964,5	35824,45

Q16270	IBP7_HUMAN	Insulin-like growth factor-binding protein 7 (IBP-7) (IGF-binding protein 7) (I(	151928,6	94104,74	129261,1	84916,91	55176,96	97741,05	201626	125015	98632,12	101612,5	88285,72	28840,11	69303,7	44378,29	171790,2	19221,76
P35858	ALS_HUMAN	Insulin-like growth factor-binding protein complex acid labile subunit (ALS)	28594,95	30093,18	30108,08	25917,32	28809,31	26031,47	29997,17	19011,47	38246,95	69023,19	20563,32	53982,98	62196,88	35540,32	32527,26	34603,4
P19827	ITIH1_HUMAN	Inter-alpha-trypsin inhibitor heavy chain H1 (ITI heavy chain H1) (ITI-HC1) (In	318527	296160,6	446937,2	325630,2	411431,9	247786,9	263924,4	232822,7	377710,5	622375,6	262923,7	781722,6	648569	538421,5	166920,6	390132,2
P19823	ITIH2_HUMAN	Inter-alpha-trypsin inhibitor heavy chain H2 (ITI heavy chain H2) (ITI-HC2) (In	548965,7	455734	752366,2	478241,7	696288,9	507555,9	468664,9	515008,7	794205,8	1033076	408478,6	995964,8	879338,1	564462,2	307793,8	586046,5
Q06033	ITIH3_HUMAN	Inter-alpha-trypsin inhibitor heavy chain H3 (ITI heavy chain H3) (ITI-HC3) (In	21247,37	15428,35	25879,13	20796,11	25841,95	25608,09	23951,03	21851,11	9258,403	14080,92	21293,97	18269,09	19171,19	32882,85	14680,13	21735,72
Q14624	ITIH4_HUMAN	Inter-alpha-trypsin inhibitor heavy chain H4 (ITI heavy chain H4) (ITI-HC4) (In	322677,3	210994	272482,9	275588,9	397376,2	352709,7	235795,3	273781,7	434939,4	502832	212648,9	493013	585838,6	397459,8	220008,1	285925,2
Q86UX2	ITIH5_HUMAN	Inter-alpha-trypsin inhibitor heavy chain H5 (ITI heavy chain H5) (ITI-HC5) (In	9578,962	15335,26	18454,68	27047,1	41923,64	18234,28	26494,65	27526,73	33674,73	20519,63	5562,075	14690,79	13098,31	13756,39	17541,56	7947,628
Q17R60	IMPG1_HUMAN	Interphotoreceptor matrix proteoglycan 1 (Interphotoreceptor matrix prote	42811,71	18814,81	46881,22	54991,69	51986,52	31573,79	43260,9	61414,4	29516,11	50276,59	236116,1	39584,67	76706,82	46297,01	180639,5	170875,5
Q9BZV3	IMPG2_HUMAN	Interphotoreceptor matrix proteoglycan 2 (Interphotoreceptor matrix prote	8462,139	12052,77	4916,906	17375,32	10272,72	7513,725	10392,95	11514,92	12707,19	10769,33	48416,71	21670,9	30577,74	13010,3	89175,73	42662,92
P29622	KAIN_HUMAN	Kallistatin (Kallikrein inhibitor) (Peptidase inhibitor 4) (PI-4) (Serpin A4)	11939,72	14070,97	13806,17	5824,728	13587,36	4235,799	9779,466	10570,15	4524,519	14507,18	6286,787	2782,302	6515,27	5665,646	2329,447	2994,631
P13645	K1C10_HUMAN	Keratin, type I cytoskeletal 10 (Cytokeratin-10) (CK-10) (Keratin-10) (K10)	355124,5	157182,9	275144,1	265451,3	575344,5	407931,3	225184,6	315682,5	301607,8	429768,5	60393,12	567652,4	324480	109707	284780,7	143431,8
P02533	K1C14_HUMAN	Keratin, type I cytoskeletal 14 (Cytokeratin-14) (CK-14) (Keratin-14) (K14)	8338,014	6128,479	11892,87	4623,282	12782,42	50642,37	8372,715	17233,8	8755,97	14248,91	1996,217	11567,64	2094,222	5046,086	14993,81	1002,581
P08779	K1C16_HUMAN	Keratin, type I cytoskeletal 16 (Cytokeratin-16) (CK-16) (Keratin-16) (K16)	2050,368	3430,823	701,2866	5584,383	3221,47	5548,869	6816,388	6374,523	891,8109	1273,801	10158,59	1069,176	4106,093	1169,098	2955,834	686,9275
P35527	K1C9_HUMAN	Keratin, type I cytoskeletal 9 (Cytokeratin-9) (CK-9) (Keratin-9) (K9)	310000,7	113010,4	226084,7	256462,9	321379,2	622893	170960,6	175085,4	129367,6	285884,7	40636,08	158485,7	57232,64	37040,17	227172,3	196499,5
P04264	K2C1_HUMAN	Keratin, type II cytoskeletal 1 (67 kDa cytokeratin) (Cytokeratin-1) (CK-1) (Hai	1599786	616674,1	1190913	1178172	1626991	2672369	664578,7	1004572	1071382	1489011	187126,5	758699,6	453325,5	193018,7	870263,9	378585
P35908	K22E_HUMAN	Keratin, type II cytoskeletal 2 epidermal (Cytokeratin-2e) (CK-2e) (Epithelial k	485035,2	96267,08	387387,5	112770,3	443424,7	418047,5	393649,7	385230,2	378589	612640,1	88131,64	230936,4	193446,4	101486,6	159562,1	115126,8
P13647	K2C5_HUMAN	Keratin, type II cytoskeletal 5 (58 kDa cytokeratin) (Cytokeratin-5) (CK-5) (Ker	65370,85	26194,09	50929,33	92473,85	56828,01	174896,5	45323,4	76350,25	37227,28	64079,97	16080,97	49230,42	36502,89	29767,77	47357,78	7618,165
P01042	KNG1_HUMAN	Kininogen-1 (Alpha-2-thiol proteinase inhibitor) (Fitzgerald factor) (High mol	661819,6	496464,2	481148,2	335309	601802,2	875522,6	441803,4	547829,7	607250	535239,3	1029459	766891,2	1573498	1440367	338314,8	751793,3
Q14767	LTBP2_HUMAN	Latent-transforming growth factor beta-binding protein 2 (LTBP-2)	704,8679	893,5879	5780,34	2772,069	1204,786	3061,966	10140,64	5328,748	5411,797	4429,626	1733,045	2291,256	348,6134	1230,694	8875,239	1308,812
P02750	A2GL_HUMAN	Leucine-rich alpha-2-glycoprotein (LRG)	618337,3	494442,7	518025	308668,6	415210,4	1139451	460098,4	267960,7	562022,4	882847,8	867739,4	407101,6	587127,8	757737,5	214804,6	390302
Q9P2V4	LRIT1_HUMAN	Leucine-rich repeat, immunoglobulin-like domain and transmembrane doma	12850,14	53874,09	19775,67	25774,06	14880,52	7776,279	22045,09	16305,27	17444,31	13151,41	6964,006	8639,43	5375,964	4258,624	11626,14	2203,454
Q13449	LSAMP_HUMAN	Limbic system-associated membrane protein (LSAMP) (IgLON family membe	31425,11	41036,52	49200,05	70224,26	40845,29	17545,48	79313,33	42822,89	52006,59	32333,72	9954,863	24775,45	32058,61	14992,52	41849,93	30400,54
P18428	LBP_HUMAN	Lipopolysaccharide-binding protein (LBP)	34135,17	13960,66	22154,92	9376,586	16121,17	40526,9	3608,661	8895,098	19391,73	14906,91	19230,87	14904,84	20961,29	15208,31	3568,13	8247,295
P00338	LDHA_HUMAN	L-lactate dehydrogenase A chain (LDH-A) (EC 1.1.1.27) (Cell proliferation-indu	1947,027	15034,32	937,5291	1908,111	5529,095	1835,379	1889,689	10439,92	3667,198	6016,639	7699,173	5682,069	7535,49	4896,52	3074,671	3980,851
P07195	LDHB_HUMAN	L-lactate dehydrogenase B chain (LDH-B) (EC 1.1.1.27) (LDH heart subunit) (L	1743,227	4156,432	7590,61	1872,639	2434,604	5691,158	3850,368	1339,897	2517,284	3203,514	1678,277	3643,232	1060,98	7091,533	1700,389	1555,391
P14151	LYAM1_HUMAN	L-selectin (CD62 antigen-like family member L) (Leukocyte adhesion molecul	3213,596	3624,747	3011,732	870,266	3944,57	4573,398	3336,474	2042,357	5815,338	2079,666	1378,364	4896,884	4637,291	787,0176	4366,883	6252,189
P51884	LUM_HUMAN	Lumican (Keratan sulfate proteoglycan lumican) (KSPG lumican)	134517,9	116870,4	153728,7	222751,9	176547	248400,9	117351,7	160783	179689,8	91582,49	57458,85	188437,1	136314,6	103116,2	87796,7	128788,6
P11117	PPAL_HUMAN	Lysosomal acid phosphatase (LAP) (EC 3.1.3.2)	2968,622	2781,77	6157,976	4034,663	5070,162	4545,027	3719,852	812,9992	6658,883	4755,494	4668,014	1658,916	6793,736	2171,091	2319,872	2407,414
P10253	LYAG_HUMAN	Lysosomal alpha-glucosidase (EC 3.2.1.20) (Acid maltase) (Aglucosidase alfa)	373,2053	3440,757	6067,151	1807,711	2736,911	1532,6	3335,451	919,6184	4187,508	2589,853	1685,317	492,8716	522,9969	1489,551	524,3116	527,0745
P10619	PPGB_HUMAN	Lysosomal protective protein (EC 3.4.16.5) (Carboxypeptidase C) (Carboxype	5198,29	1460,982	7183,231	6332,515	5079,656	1853,04	13938,1	6614,954	4231,636	4156,431	12123,79	27297,73	6329,793	7175,056	9053,545	5947,293
P61626	LYSC_HUMAN	Lysozyme C (EC 3.2.1.17) (1,4-beta-N-acetylmuramidase C)	7837,886	5021,016	3900,025	6669,382	1761,106	604,609	2520,165	4694,335	1781,223	3475,3	1544,23	1087,974	2351,513	969,1632	1513,015	3662,577
P40925	MDHC_HUMAN	Malate dehydrogenase, cytoplasmic (EC 1.1.1.37) (Cytosolic malate dehydrog	3043,879	3151,934	5022,967	8393,625	3396,564	1906,788	10475,27	5192,64	1314,899	3480,356	1710,348	3643,157	717,9174	6040,234	8327,758	1359,159
P33908	MA1A1_HUMAN	Mannosyl-oligosaccharide 1,2-alpha-mannosidase IA (EC 3.2.1.113) (Man(9)-a	3354,268	4353,846	11568,64	35139,52	2985,923	6455,684	11527,39	9527,621	30746,51	35983,36	4947,982	28435,75	21304,08	21512,52	4160,195	2186,27
Q9NR34	MA1C1_HUMAN	Mannosyl-oligosaccharide 1,2-alpha-mannosidase IC (EC 3.2.1.113) (HMIC) (N	790,1784	387,8889	786,4869	822,9935	340,8605	2108,456	6272,374	2206,597	1677,576	1928,116	2574,597	13054,87	1633,818	2511,778	7515,725	4149,971
P08493	MGP_HUMAN	Matrix Gla protein (MGP) (Cell growth-inhibiting gene 36 protein)	5846,39	5561,319	10747,33	3814,932	4690,014	4454,95	3649,212	4533,264	6029,935	1816,869	2987,03	3208,704	974,7488	12446,39	5111,46	1469,376
P01033	TIMP1_HUMAN	Metalloproteinase inhibitor 1 (Erythroid-potentiating activity) (EPA) (Fibrobl	66039,92	129360,4	176088,2	155195,7	112372,3	145541,6	41763,01	34380,9	77306,7	31647,67	50054,73	80334,79	22764,11	83724,76	416929,8	95236,93
P16035	TIMP2_HUMAN	Metalloproteinase inhibitor 2 (CSC-21K) (Tissue inhibitor of metalloproteinas	35596,9	24479,36	23482,64	39533,64	26748,93	23035,88	33214,41	32881,75	28649,27	20806,86	11017,9	8806,267	8246,54	11366,78	32205,19	3964,752
Q9UJH8	METRN_HUMAN	Meteorin	2355,79	763,723	2576,24	1746,637	2484,011	275,3687	3330,591	1853,014	996,8635	1734,657	1227,039	449,498	2063,984	1767,349	2896,401	1513,356
P55083	MFAP4_HUMAN	Microfibril-associated glycoprotein 4	53438,94	58857,28	81877,63	60943,89	127351,4	56123,45	110598,7	53239,53	72383,37	58858,64	22866,28	9070,528	38740,06	40505,57	38900,62	8253,057
P08571	CD14_HUMAN	Monocyte differentiation antigen CD14 (Myeloid cell-specific leucine-rich gly	74545,11	52191,92	51288,24	33715,97	34956,52	37497,34	31813,18	24143,38	38437,98	27988,69	61587,65	44223,87	131106,5	46529,03	42032,59	31494,78
Q7Z7M0	MEGF8_HUMAN	Multiple epidermal growth factor-like domains protein 8 (Multiple EGF-like c	3528,638	10522,16	40654,24	18390,54	26919,77	20871,97	13241,18	7377,259	24623,37	20966,22	19078,59	7098,971	21142,87	25566,23	14428,55	12595,7
Q9UNW1	MINP1_HUMAN	Multiple inositol polyphosphate phosphatase 1 (EC 3.1.3.62) (2,3-bisphosphc	11660,54	10240,58	32796,72	16609,51	13327,23	26784,59	20977,44	29620,53	12302,12	5363,978	18548,94	8654,654	12824,13	10720,95	5435,306	3990,957
Q99972	MYOC_HUMAN	Myocilin (Myocilin 55 kDa subunit) (Trabecular meshwork-induced glucocort	73393,75	43126,13	105317,9	49899,48	57064,39	40396,3	128221,5	110369	61149,29	60635,67	28851,56	24233,86	39530,23	59557,85	124120,6	7958,916
Q9Y2K3	MYH15_HUMAN	Myosin-15 (Myosin heavy chain 15)	13598,33	12770,14	12441,95	7776,729	19673,32	29685,5	9763,856	20933,23	15708,71	21145,96	17997,49	21179,38	32372,89	29881,97	8605,36	6730,513
P15586	GNS_HUMAN	N-acetylglucosamine-6-sulfatase (EC 3.1.6.14) (Glucosamine-6-sulfatase) (G6:	16442,18	211315,2	94309,01	168544,5	63619,82	38780,6	288404,1	72036,05	81492,4	41698,76	40156,84	64479,56	28764,27	18401,67	49574,57	31156,15
Q96PD5	PGRP2_HUMAN	N-acetylmuramoyl-L-alanine amidase (EC 3.5.1.28) (Peptidoglycan recognitio	79542,93	88821,08	196167,2	148828	113270,2	181636,2	64781,49	95592,34	115815,7	109687,4	68568,98	247839,4	178896	101526,1	35968,17	65538,19
Q92859	NEO1_HUMAN	Neogenin (Immunoglobulin superfamily DCC subclass member 2)	4078,324	2858,194	4447,894	870,8403	27875,45	2900,896	1928,655	2225,893	14764,5	2758,551	2370,245	2097,252	53010,89	851,7371	332,4907	47495,82
P13591	NCAM1_HUMAN	Neural cell adhesion molecule 1 (N-CAM-1) (NCAM-1) (CD antigen CD56)	11036,54	22390,08	31088,46	39945,25	26962,21	12512,34	26123,78	20516,82	15125,77	14139,56	8851,028	7114,416	11839,86	8115,331	12436,93	1110,294
P32004	L1CAM_HUMAN	Neural cell adhesion molecule L1 (N-CAM-L1) (NCAM-L1) (CD antigen CD171)	1868,138	1173,678	2774,183	15201,71	3776,757	1618,498	38298,14	820,3626	2423,54	2390,015	468,579	687,0507	495,8171	753,3941	583,9802	855,0665
Q9P2S2	NRX2A_HUMAN	Neurexin-2 (Neurexin II-alpha) (Neurexin-2-alpha)	6537,919	15854,9	24078,17	16230,82	9894,213	5780,292	24974,95	11164,09	1577,998	9702,73	2808,516	3284,363	7930,003	28305,59	7685,83	1894,028
Q9Y4C0	NRX3A_HUMAN	Neurexin-3 (Neurexin III-alpha) (Neurexin-3-alpha)	96500,38	114155	66654,38	73563,74	89979,73	98126	35047,58	96286,2	95048,22	100728,4	134437,1	191770,4	70125,06	196994,9	30040,21	46605,72
P41271	NBL1_HUMAN	Neuroblastoma suppressor of tumorigenicity 1 (DAN domain family member	65860,68	55667,4	76352,88	66509,71	52427,35	73694,32	132950,9	65843,09	63380,23	40602,44	34206,96	2496,992	19629,12	22124,93	36135,61	8903,886
O94856	NFASC_HUMAN	Neurofascin	31545,4	40398,24	44143,37	53280,76	41505,48	37878,53	60258,29	54722,62	35259,52	15314,29	20526,09	8566,056	6502,538	10762,63	14907,9	5930,987
Q7Z3B1	NEGR1_HUMAN	Neuronal growth regulator 1 (IgLON family member 4)	10739,52	18252,43	18588,71	19628,67	16432,36	3718,503	4409,04	2162,743	14709,94	8289,45	5105,815	1336,238	3897,743	4110,22	11087,22	1699,513
Q15818	NPTX1_HUMAN	Neuronal pentraxin-1 (NP1) (Neuronal pentraxin I) (NP-I)	2195,997	983,6065	802,039	1983,987	2191,306	1495,41	19868,72	6174,194	4859,315	651,187	717,0902	9296,455	4123,668	9958,585	8086,644	4406,412
Q9P121	NTRI_HUMAN	Neurotrimin (hNT) (IgLON family member 2)	10713,85	41849,76	27768,24	38500,21	37791,89	37121	46407,43	49008,61	31026,66	43470,56	47694,68	19557,71	44737,31	37305,42	17860,18	26842,83
Q99784	NOE1_HUMAN	Noelin (Neuronal olfactomedin-related ER localized protein) (Olfactomedin-1	25176,42	4521,382	9801,295	2857,575	27271,8	50605,04	21889,13	1988,933	2783,855	2293,551	1904,869	2588,42	8060,885	4939,147	17532,51	5205,535
O95897	NOE2_HUMAN	Noelin-2 (Olfactomedin-2)	4014,512	11899,4	10825,3	7572,659	7153,957	2641,013	6306,547	3072,183	10118,48	5979,764	4021,826	2983,857	4328,519	4909,691	13239,39	2023,43
P61916	NPC2_HUMAN	NPC intracellular cholesterol transporter 2 (Epididymal secretory protein E1)	9885,689	13881,33	6669,729	5268,023	2865,984	11198,88	13375,72	16394,13	14219,78	6499,884	14373,44	10115,16	3915,829	7263,59	10333,41	13707,02

Q02818	NUCB1_HUMAN	Nucleobindin-1 (CALNUC)	37137,34	55864,74	64486,95	91178,69	49648,04	30467,92	62307,01	45108,87	38795,96	37178,78	28284,93	36147,5	41235,27	32762,41	26870,07	27595,69
P23515	OMGP_HUMAN	Oligodendrocyte-myelin glycoprotein	7005,663	564,2994	1123,26	477,3391	24004,54	1417,688	42398,35	7148,868	15523,34	458,5717	8599,546	7764,597	871,5773	956,9452	2966,781	369,6853
Q9UBM4	OPT_HUMAN	Opticin (Oculoglycan)	572137,4	445277,7	590743,7	300155,1	343047,7	421212,8	1760268	824802,1	665740,5	291907,2	199714,9	202987,3	319117,5	704048,3	136210,2	
Q86UD1	OAF_HUMAN	Out at first protein homolog (HCV NS5A-transactivated protein 13 target pro	12928,38	42622,86	83412,13	69776,59	5436,295	23768,18	121770,3	92534,71	49520,05	29663,95	5946,436	30670,36	62145,4	31600,8	87746,36	16147,93
P50897	PPT1_HUMAN	Palmitoyl-protein thioesterase 1 (PPT-1) (EC 3.1.2.22) (Palmitoyl-protein hyd	8964,379	2847,148	15411,82	7325,837	10147,75	5305,663	2659,167	8102,136	7087,523	4886,763	4231,14	4041,348	2432,139	2179,36	9818,349	1415,37
O95428	PPN_HUMAN	Papilin	573,3721	286,8671	578,8738	406,4105	433,3534	1010,795	852,1943	678,617	935,3799	323,5256	413,0918	735,3864	1538,682	192,8652	483,8621	287,9384
Q9BXP8	PAPP2_HUMAN	Pappalysin-2 (EC 3.4.24.-) (Pregnancy-associated plasma protein A2) (PAPP-A	21175,12	31128,67	12589,66	21200,95	19953,14	9773,812	33101,34	11321,53	11516,82	13205,85	7454,371	8782,666	12818,93	6336,462	12321,58	5755,459
Q6UXB8	PI16_HUMAN	Peptidase inhibitor 16 (PI-16) (Cysteine-rich secretory protein 9) (CRISP-9) (P	18496,97	1985,864	2071,352	2628,158	8432,353	7287,417	2791,47	7283,6	11498,83	2368,569	4383,973	3575,086	10896,66	12562,72	3098,775	4136,744
P19021	AMD_HUMAN	Peptidyl-glycine alpha-amidating monooxygenase (PAM) [Includes: Peptidylg	1430,397	2740,155	4080,813	1977,973	840,9441	3519,31	1715,998	6391,875	2041,6	4027,553	1700,143	4660,864	784,4617	760,3441	1785,447	1543,21
P32119	PRDX2_HUMAN	Peroxiredoxin-2 (EC 1.11.1.15) (Natural killer cell-enhancing factor B) (NKEF-I	10948,83	12159,24	14936,67	32951,05	11929,27	11122,87	13368,72	12492,15	45126,58	15971,82	27224,9	19472,22	9754,735	20033,62	13026,2	11980,71
P30086	PEBP1_HUMAN	Phosphatidylethanolamine-binding protein 1 (PEBP-1) (HCNPpp) (Neuropoly	8291,646	2186,294	18206,81	1479,408	11138,69	4378,614	20786,56	583,8128	2296,161	7956,946	15256,26	2584,937	3635,68	481,4782	28904,78	8026,217
Q96S96	PEBP4_HUMAN	Phosphatidylethanolamine-binding protein 4 (PEBP-4) (hPEBP4) (Protein cou	8691,145	20425,56	7964,201	5777,099	7583,304	3066,052	27113,69	6605,079	13732,6	11198,68	19096,48	24482,65	16410,46	21738,15	3840,442	12813,9
P80108	PHLD_HUMAN	Phosphatidylinositol-glycan-specific phospholipase D (PI-G PLD) (EC 3.1.4.50)	2956,48	10590,06	3128,252	5755,635	6274,109	3324,567	6470,404	10177,89	5112,768	9323,265	7964,369	15359,6	13534,08	8132,737	6306,055	4659,862
Q96FE7	P3IP1_HUMAN	Phosphoinositide-3-kinase-interacting protein 1 (Kringle domain-containing	2816,524	5370,711	8753,183	5672,028	5774,523	4967,549	5809,494	504,8401	135,7779	3161,029	1472,162	2094,492	2106,772	1205,336	5259,127	2344,221
Q8IV08	PLD3_HUMAN	Phospholipase D3 (PLD 3) (EC 3.1.4.4) (Choline phosphatase 3) (HindIII K4L h	39316,97	45082,1	55979,22	42988,87	36363,5	23008,3	32289,81	33226,4	38391,6	15229,44	11587,46	10375,02	8327,01	8280,103	17268,03	5146,726
P55058	PLTP_HUMAN	Phospholipid transfer protein (Lipid transfer protein II)	22629,14	15978,37	22840,87	17980,83	29221,47	10247,77	32501,96	8171,688	8748,855	23969,1	65663,63	27626,81	92516,91	14230,71	4257,812	10831,1
P36955	PEDF_HUMAN	Pigment epithelium-derived factor (PEDF) (Cell proliferation-inducing gene 3	13644986	36730149	33241021	34369544	23909741	17056094	22376226	23458224	21820793	14744166	12823503	5677186	6712555	8280313	23006944	4036011
Q63HQ2	EGFLA_HUMAN	Pikachurin (Agrin-like protein) (EGF-like, fibronectin type-III and laminin G-lik	4067,385	3845,633	9122,306	7465,824	6617,267	6083,578	3944,179	2959,43	5531,854	7518,662	3495,967	2219,201	6072,328	2513,73	3210,954	3633,221
Q13835	PKP1_HUMAN	Plakophilin-1 (Band 6 protein) (B6P)	975,7061	1137,072	573,0704	3729,861	1634,203	3971,089	2141,337	1555,122	3226,869	1673,843	3163,549	2516,146	725,8377	2859,15	1428,056	327,1365
Q9BTY2	FUCO2_HUMAN	Plasma alpha-L-fucosidase (EC 3.2.1.51) (Alpha-L-fucoside fucohydrolase 2) (/	11132,51	10347,49	5994,092	17015,87	15383,89	6273,823	9523,715	13698,77	11201,72	3644,162	6114,625	18162,46	6884,228	8494,672	6948,97	1864,582
P03952	KLKB1_HUMAN	Plasma kallikrein (EC 3.4.21.34) (Fletcher factor) (Kininogenin) (Plasma prekal	2088,948	905,8273	1197,07	1086,488	2750,459	3956	3095,553	2279,914	1437,844	2841,946	1112,043	7577,348	1875,946	5836,887	5030,854	4283,796
P05155	IC1_HUMAN	Plasma protease C1 inhibitor (C1 Inh) (C1Inh) (C1 esterase inhibitor) (C1-inhil	1819932	2540804	1610507	1391854	2108015	1848290	1988891	1623101	2178583	1913811	1737723	1623786	1673009	1407990	1628321	1039161
P05154	IPSP_HUMAN	Plasma serine protease inhibitor (Acrosomal serine protease inhibitor) (Plasn	4490,536	1895,684	2082,088	3434,429	8954,179	3034,452	5086,159	6856,639	5551,293	8506,401	4149,423	10869,73	4370,333	10350,3	8151,205	8943,295
P00747	PLMN_HUMAN	Plasminogen (EC 3.4.21.7) [Cleaved into: Plasmin heavy chain A; Activation p	390609	281369,4	404193,1	351028,9	442756,1	733113,3	457328,5	305820,4	380927,8	533894	454920	514767,5	702270,5	615438,2	420740,9	512286,8
Q6UX71	PXDC2_HUMAN	Plexin domain-containing protein 2 (Tumor endothelial marker 7-related pro	43915,21	56070,1	44298,76	32478,95	77252,55	8918,569	19234,14	33513,62	30154,67	11976,62	5046,975	55502,17	20798,89	13918,28	12011,9	6454,256
O15031	PLXB2_HUMAN	Plexin-B2 (MM1)	11650,57	3290,482	7465,762	13864,93	9443,154	10216,89	11841,72	7933,568	6028,928	13695,61	1844,834	15003,68	12820,61	13256,02	6009,975	7385,097
Q10471	GALT2_HUMAN	Polypeptide N-acetylgalactosaminyltransferase 2 (EC 2.4.1.41) (Polypeptide (	909,6679	1434,372	7988,12	5635,384	8111,544	5048,179	13200,08	8367,483	3388,931	5923,71	2430,571	1126,23	724,8997	1901,228	2375,216	447,7741
Q8NA82	MARHA_HUMAN	Probable E3 ubiquitin-protein ligase MARCH10 (EC 2.3.2.27) (Membrane-ass(	333,8102	204,5582	456,1126	0	322,8094	667,6251	0	472,3067	279,3816	305,8005	789,8638	221,7108	1946,596	899,8347	311,1593	351,6348
Q9H3G5	CPVL_HUMAN	Probable serine carboxypeptidase CPVL (EC 3.4.16.-) (Carboxypeptidase, vite	1862,537	1905,069	804,5837	10538,52	1714,149	325805,1	2688,698	4962,74	836,0293	5963,868	570,0764	3042,618	2588,488	5180,809	3792,612	1166,154
Q02809	PLOD1_HUMAN	Procollagen-lysine,2-oxoglutarate 5-dioxygenase 1 (EC 1.14.11.4) (Lysyl hydr(	12064,69	8555,562	5150,285	12980,51	7790,494	4093,099	3111,268	8530,264	4602,126	3762,245	2120,624	8064,225	4115,725	3265,857	7859,363	2389,042
P07737	PROF1_HUMAN	Profilin-1 (Epididymis tissue protein Li 184a) (Profilin I)	4010,486	2471,049	15168,96	10613,54	8777,45	3405,026	3151,937	1450,388	5263,739	3449,462	13371,16	5237,613	4507,971	7411,086	15144,06	8472,78
P51888	PRELP_HUMAN	Prolargin (Proline-arginine-rich end leucine-rich repeat protein)	10064	13514,76	2821,675	7790,629	7397,379	8240,53	20657,04	8986,329	10238,55	11242,4	6128,738	4139,282	10871,17	8207,063	11748,29	5339,041
P0DMB1	P23D2_HUMAN	Proline-rich protein 23D2	122427	120076,4	69420,4	78109,44	4269,92	131118,2	122425,6	108886,2	77998,49	105580	3067,599	147355,1	158485,9	142243,1	49463,76	70268,25
Q07954	LRP1_HUMAN	Prolow-density lipoprotein receptor-related protein 1 (LRP-1) (Alpha-2-macr(	18117,2	7142,81	19716,03	8301,452	10221,86	6758,211	9123,392	7071,887	4104,263	11770,18	8474,208	5664,718	10676,75	2359,266	22645,06	7619,554
Q9UHG2	PCSK1_HUMAN	ProSAAS (Proprotein convertase subtilisin/kexin type 1 inhibitor) (Proproteir	40304,82	48089,14	69428,43	58154,16	43016,52	30103,53	61351,88	38602,33	49605,53	24568,61	32760,01	34964,83	25865,05	17874,04	36683,78	14000,38
P07602	SAP_HUMAN	Prosaposin (Proactivator polypeptide) [Cleaved into: Saposin-A (Protein A); S	22459,42	19003,42	14381,02	34047,56	211769,7	149597,4	297377,5	206934,6	242396,3	111101,5	130840	21741,28	83837,44	65605,53	163580,3	39255,21
P41222	PTGDS_HUMAN	Prostaglandin-H2 D-isomerase (EC 5.3.99.2) (Beta-trace protein) (Cerebrin-28	4099345	6214504	8101137	1056710	5006901	165595,2	301252,2	5119191	4316862	199080,1	2309864	85284,27	1480787	125442,3	8307597	77298,2
P02760	AMBP_HUMAN	Protein AMBP [Cleaved into: Alpha-1-microglobulin (Protein HC) (Alpha-1 mi(	25868,03	11430,75	12384,63	10317,37	25010,91	14198,64	15560,15	11630,54	32274,69	29056,11	48546,86	88858,53	71503,38	71301,14	16170,65	41600,6
O60888	CUTA_HUMAN	Protein CutA (Acetylcholinesterase-associated protein) (Brain acetylcholinest	34260,44	12121,93	14169,7	32494,35	9282,39	2549,711	4464,948	8201,371	8232,645	5581,515	12307,31	4198,932	6479,072	3848,949	12400,11	2853,478
Q92520	FAM3C_HUMAN	Protein FAM3C (Interleukin-like EMT inducer)	167697,2	57426,82	23455,98	65043,82	106619	31896,41	30443,23	60774,14	47142,26	112741	186911,5	6824,515	26100,95	17423,04	132417,6	61046,8
Q99435	NELL2_HUMAN	Protein kinase C-binding protein NELL2 (NEL-like protein 2) (Nel-related prot	3837,502	2184,655	2921,108	3077,344	3779,775	4610,005	8002,426	3778,62	1339,225	1883,69	1392,754	735,3539	1104,035	975,1717	7120,436	4434,072
Q9UPW8	UN13A_HUMAN	Protein unc-13 homolog A (Munc13-1)	8350,987	4915,432	10339,65	6905,425	8628,124	8573,764	10686,99	5181,571	11307,46	4572,696	7034,746	10904,6	10638,8	3092,518	3798,509	3966,516
Q9GZM5	YIPF3_HUMAN	Protein YIPF3 (Killer lineage protein 1) (Natural killer cell-specific antigen KLIF	28830,39	33139,01	50222,51	69230,11	31457,57	20506,04	50623,75	40846,38	29892,45	20869,38	10270,34	1204,824	813,1438	4514,821	4218,804	286,2576
P00734	THRB_HUMAN	Prothrombin (EC 3.4.21.5) (Coagulation factor II) [Cleaved into: Activation pe	173490,4	164379,6	206120,2	166289,6	355974,6	329167,8	312317,7	307732	336058	297345	406698,5	481780,2	650857,6	508487,7	228315	240307,1
Q8NHP8	PLBL2_HUMAN	Putative phospholipase B-like 2 (EC 3.1.1.-) (76 kDa protein) (p76) (LAMA-like	63484,79	10415,01	2537,725	22423,93	44043,44	8354,585	46053,16	11764,75	8993,241	6778,854	4177,144	8872,393	1821,725	8919,765	23130,44	12437,99
P14618	KPYM_HUMAN	Pyruvate kinase PKM (EC 2.7.1.40) (Cytosolic thyroid hormone-binding prote	49339,7	12525,32	26546,34	22330	33786,01	15572,46	40477,55	20497,07	16283,33	16463,49	23617,43	12936,11	15951,27	18623,3	56864,97	24122,9
Q13332	PTPRS_HUMAN	Receptor-type tyrosine-protein phosphatase S (R-PTP-S) (EC 3.1.3.48) (Recep	8726,844	9197,641	2200,623	13992,46	16175,26	16677,57	9771,52	12139,93	8260,806	11982,89	10543,53	6843,847	31835,47	8925,413	8022,506	8431,476
P23471	PTPRZ_HUMAN	Receptor-type tyrosine-protein phosphatase zeta (R-PTP-zeta) (EC 3.1.3.48) (	29149,45	68494,95	80989,9	67936,83	61766,8	30550,5	48959,47	42863,92	43624,2	33206,83	24761,83	10470,13	20610,81	7200,988	25909,52	7795,074
P78509	RELN_HUMAN	Reelin (EC 3.4.21.-)	28008,05	24845,53	33452,9	33579,7	12341,38	14663,62	11283,24	27111,53	18926,61	34896,55	15562,54	9655,067	11035,54	7578,901	34985,66	14091,42
O75787	RENR_HUMAN	Renin receptor (ATPase H(+)-transporting lysosomal accessory protein 2) (AT	21451,45	33799,82	53836,99	54064,45	33039,85	31954,15	43665,43	53657,06	37049,71	8436,294	83082,57	12709,94	25455,24	24217,72	34619,06	5979,284
Q6XPR3	RPTN_HUMAN	Repetin	38198,52	130679,9	41662,43	63202,52	52143,9	24137,49	25925,98	33466,24	22318,27	16119,41	27830,15	19300,32	23715,44	23333,29	13982,85	19483,77
P49788	TIG1_HUMAN	Retinoic acid receptor responder protein 1 (Phorbol ester-induced gene 1 pr	4115,481	825,008	3746,102	2539,021	16414,74	19401,08	6219,543	9548,87	766,3939	10806,87	652,8412	47841,51	72980,7	1027,409	3009,227	606,7105
Q99969	RARR2_HUMAN	Retinoic acid receptor responder protein 2 (Chemerin) (RAR-responsive prot	47084,91	24859,02	39809,33	29865,86	35366,07	36101,9	67053,35	51973,08	45777,6	30758,97	19612,88	30532	4309,364	22829,51	47617,09	8128,683
P10745	RET3_HUMAN	Retinol-binding protein 3 (Interphotoreceptor retinoid-binding protein) (IRB	3078047	6130916	8225221	9013409	5776581	5420477	9318762	5904878	7657340	4389579	5991214	2521395	2187860	2252281	25426768	3184608
P02753	RET4_HUMAN	Retinol-binding protein 4 (Plasma retinol-binding protein) (PRBP) (RBP) [Clea	30061,58	41495,75	61908,68	31386,33	82012,32	172147,5	202024,8	143382,8	82665,68	132599	307550,9	425503,1	322301	317272,5	245982	256324
O15537	XLRS1_HUMAN	Retinoschisin (X-linked juvenile retinoschisis protein)	9740,66	30600,43	39971,31	16789,99	78844,21	44584,44	81904,47	35703,51	69193,36	58050,48	21388,62	41271,58	23605,49	42585,71	77190,47	12057,13
P07998	RNAS1_HUMAN	Ribonuclease pancreatic (EC 3.1.27.5) (HP-RNase) (RIB-1) (RNase UpI-1) (Ribc	2017,635	2668,212	2289,814	1823,292	3937,491	9085,619	2996,243	3152,204	7056,42	4806,092	8939,293	5584,304	7923,636	8314,837	14426,09	4222,886
O00584	RNT2_HUMAN	Ribonuclease T2 (EC 3.1.27.-) (Ribonuclease 6)	15033,19	26800,52	28801,35	20978,13	9225,312	11844,14	22982,91	8299,659	11639,91	16608,15	10339,26	7223,608	12917,59	2510,386	13983,91	11496,32
P16499	PDE6A_HUMAN	Rod cGMP-specific 3',5'-cyclic phosphodiesterase subunit alpha (GMP-PDE al	393,7781	3052,201	438,8458	725,7096	1280,165	506,3326	338,1182	870,1124	1221,677	273,2671	247,2999	267,3709	697,327	342,2329	3777,105	234,402

P35913	PDE6B_HUMAN	Rod cGMP-specific 3',5'-cyclic phosphodiesterase subunit beta (GMP-PDE be	35096,6	21127,99	44579,12	14482,34	37314,55	11345,11	266717,9	12955,54	9859,432	935704,3	10888,21	8861,392	22021,23	13141,25	27333,65	133318,6
P10523	ARRS_HUMAN	S-arrestin (48 kDa protein) (Retinal S-antigen) (S-AG) (Rod photoreceptor arr	16373,22	33672,96	32593,12	25585,31	16005,3	19273,8	55522,68	17781,58	14416,01	21882,63	87066,25	80104,64	27397,61	21013,01	177929,1	27190,94
Q92765	SFRP3_HUMAN	Secreted frizzled-related protein 3 (sFRP-3) (Frezzled) (Fritz) (Frizzled-related	56950,38	71069,2	145794,1	73774,22	78529,14	72789,43	112656,5	116168	140126,7	52631,12	30269,91	49439,77	56463,22	40408,98	67754,72	12991,86
P05060	SCG1_HUMAN	Secretogranin-1 (Chromogranin-B) (CgB) (Secretogranin I) (SgI) [Cleaved into	12543,28	7187,519	11380,63	18375,85	16638,17	8408,662	16653,26	16147,41	17522,92	8244,831	7078,676	11004,41	16885,71	6092,782	20517,89	10214,84
P13521	SCG2_HUMAN	Secretogranin-2 (Chromogranin-C) (Secretogranin II) (SgII) [Cleaved into: Sec	24150,81	47273,36	77916,25	70600,74	50081,48	6533,352	67627,35	60758,97	49775,79	15708,22	6959,761	15147,02	24329,65	4786,065	22640,87	15907,53
P49908	SEPP1_HUMAN	Selenoprotein P (SeP)	23207,04	25368,33	32278,58	44209,98	31278,26	27197,19	31735,58	32767,77	27055,32	18401,02	13948,66	24200,2	11566,64	22696,21	12663,49	8822,177
Q14563	SEM3A_HUMAN	Semaphorin-3A (Semaphorin III) (Sema III)	4484,6	10101,06	23202,51	26145,96	72236,27	47042,01	8852,056	17064,82	20074,15	77023,76	62834,56	33784,66	59330,01	59651,74	34088,02	97286,52
Q9NPR2	SEM4B_HUMAN	Semaphorin-4B	17350,49	18830,3	23273,52	33781,13	19221	15779,81	18435,02	5149,236	14246,05	11339,97	5869,404	5807,172	6814,79	8008,056	8067,939	5169,455
O75326	SEM7A_HUMAN	Semaphorin-7A (CDw108) (JMH blood group antigen) (John-Milton-Hargen h	50832,82	133361,1	132751,1	182447,3	109751,2	88155,07	137846,6	93803,55	97667,81	88990,46	48236,89	59108,23	61067,32	63058,08	99651,61	30864,66
Q92743	HTRA1_HUMAN	Serine protease HTRA1 (EC 3.4.21.-) (High-temperature requirement A serine	18234,56	17744,55	37298,7	30671,18	15107,52	12342,07	22570,83	14531,71	25256,8	3579,278	16010,67	6353,7	4056,984	9048,321	19747,3	6751,219
P02787	TRFE_HUMAN	Serotransferrin (Transferrin) (Beta-1 metal-binding globulin) (Siderophilin)	16105128	28780272	24050701	48669735	13252468	5588940	8500371	7788532	6695422	4401651	5596714	4818972	4405499	5372394	8863801	4050145
A8MV23	SERP3_HUMAN	Serpin E3	12403,52	26001,16	16553,34	17166,36	12845,97	8245,76	32579,52	18530,24	28539,01	18171,87	9980,726	5587,39	7612,11	3652,76	7956,353	6345,392
P02768	ALBU_HUMAN	Serum albumin	1,51E+08	1,01E+08	96655237	86474647	1,25E+08	1,47E+08	1,38E+08	1,43E+08	1,24E+08	1,52E+08	1,52E+08	1,66E+08	1,58E+08	1,69E+08	1,19E+08	1,82E+08
P0DJI8	SAA1_HUMAN	Serum amyloid A-1 protein (SAA) [Cleaved into: Amyloid protein A (Amyloid	645,9456	2222,647	3990,742	927,699	4787,748	3048,19	1369,595	4608,235	406,9485	2953,647	4283,638	6466,013	2090,729	1157,174	1524,421	4937,126
P35542	SAA4_HUMAN	Serum amyloid A-4 protein (Constitutively expressed serum amyloid A prote	10134,46	8990,299	5800,516	26750,01	11121,78	3249,561	27201,24	18526,33	31697,65	32237,71	14664,93	46029,85	39747,59	25998,16	29510,87	53484,03
P27169	PON1_HUMAN	Serum paraoxonase/arylesterase 1 (PON 1) (EC 3.1.1.2) (EC 3.1.1.81) (EC 3.1.:	85559,62	29971,27	45707,88	84901,95	21350,11	48975,83	40600,43	58767,51	81791,41	83425,92	81421,12	178595,9	128245,9	55331,31	53273,56	116746,9
P04278	SHBG_HUMAN	Sex hormone-binding globulin (SHBG) (Sex steroid-binding protein) (SBP) (Te	25594,54	5598,676	20850,83	7652,629	12839,93	13495,69	6008,569	4433,638	14882,61	4992,603	8954,464	4024,738	7980,705	8692,005	1761,723	5050,139
Q9HAT2	SIAE_HUMAN	Sialate O-acetylesterase (EC 3.1.1.53) (H-Lse) (Sialic acid-specific 9-O-acetyles	1158,86	2136,274	2794,389	1681,528	1274,902	578,1923	1436,709	1345,84	398,9869	3815,248	503,0304	2853,83	348,6134	433,7402	5806,909	1673,802
Q99519	NEUR1_HUMAN	Sialidase-1 (EC 3.2.1.18) (Acetylneuraminyl hydrolase) (G9 sialidase) (Lysoson	8913,168	13240,64	16904,38	16099,59	14292,54	7443,662	11157,94	646,21	6060,457	8109,006	4352,078	289,6412	551,9947	897,7715	4355,412	597,1865
Q92543	SNX19_HUMAN	Sorting nexin-19	2298,787	562,4801	975,3153	590,8664	1438,152	1019,619	1917,67	1491,578	2313,942	941,6089	3048,169	3042,806	4166,387	3011,022	1863,372	650,5317
P09486	SPRC_HUMAN	SPARC (Basement-membrane protein 40) (BM-40) (Osteonectin) (ON) (Secre	9151,84	5997,452	36138,52	28788,26	4654,394	14631,06	14863,31	9224,541	6239,822	2726,984	8465,284	15779,23	11348,15	147980,5	5568,667	8713,94
Q14515	SPRL1_HUMAN	SPARC-like protein 1 (High endothelial venule protein) (Hevin) (MAST 9)	80425,72	118477,6	142342	158520,6	99086,71	91936,28	153080,6	121495,6	97234,45	84421,77	51887,08	37578	30765,48	55113,28	85902,57	33390
Q9HCB6	SPON1_HUMAN	Spondin-1 (F-spondin) (Vascular smooth muscle cell growth-promoting facto	174665	277083,3	354105,1	330800,2	243649,4	126630,8	273998,4	216969,3	227016,3	155724,9	69115	110458,1	132312,2	95430,47	111686,6	41462,4
O00391	QSOX1_HUMAN	Sulfhydryl oxidase 1 (hQSOX) (EC 1.8.3.2) (Quiescin Q6)	31609,58	28181,99	46570,65	35163,11	25060,03	38734,21	22986,46	33149,43	21396,1	11728,37	7509,814	26999,39	19709,41	16686,71	43713,09	11652,23
Q7Z7G0	TARSH_HUMAN	Target of Nesh-SH3 (Tarsh) (ABI gene family member 3-binding protein) (Nes	53438,39	119433,1	129846,6	252612,2	128741,3	37133,36	567089,2	250944,8	126168,3	123818,7	21489,16	26085,9	32399,9	31273,14	65059,52	67226,27
O60347	TBC12_HUMAN	TBC1 domain family member 12	1423,91	2950,522	3965,039	2351,26	484,0817	709,557	894,9299	1242,987	361,3758	247,6069	885,3048	891,2783	627,262	876,7231	900,4606	527,4432
P24821	TENA_HUMAN	Tenascin (TN) (Cytotactin) (GMEM) (GP 150-225) (Glioma-associated-extracel	1536,305	1584,205	3935,96	991,2054	7626,547	1349,389	1526,956	1547,596	9791,243	1963,864	1254,5	8254,926	9650,734	2424,055	3993,844	2579,968
Q92752	TENR_HUMAN	Tenascin-R (TN-R) (Janusin) (Restrictin)	14192,59	21401,25	41148,56	32676,84	48725,18	13672,55	25847,67	27600,6	23133,57	8919,743	11223,32	9343,147	15303,09	8306,287	23981,47	13323,27
Q92563	TICN2_HUMAN	Testican-2 (SPARC/osteonectin, CWCV, and Kazal-like domains proteoglycan :	17922,48	16702,5	38646,32	22421,32	20757,11	16313,07	33441,08	70862,61	19361,92	7317,851	15132,63	15133,7	19471,52	3874,048	19989,04	6476,539
Q9BQ16	TICN3_HUMAN	Testican-3 (SPARC/osteonectin, CWCV, and Kazal-like domains proteoglycan :	12188,16	8412,574	3141,024	3584,085	1667,56	8635,595	4941,001	5124,736	1380,149	9014,329	1564,124	795,3478	6991,1	4068,468	5254,39	351,9663
P05452	TETN_HUMAN	Tetranectin (TN) (C-type lectin domain family 3 member B) (Plasminogen krii	33719,89	36356,99	52321,13	17705,7	29709,83	30643,74	49411,6	44852,06	34807,98	47463,63	30603,81	28801,56	52076,98	20493,79	16773,23	25280,69
P10599	THIO_HUMAN	Thioredoxin (Trx) (ATL-derived factor) (ADF) (Surface-associated sulphydryl p	7464,441	3130,953	12720,99	5535,54	2804,579	6687,959	11940,12	3970,085	3826,918	3789,162	4482,036	18039,61	16838,08	4617,655	1061,231	6100,663
P35443	TSP4_HUMAN	Thrombospondin-4	4151,607	2419,472	5987,411	2773,55	1983,7	4552,253	27895,4	693,1914	2783,774	1749,806	6439,103	2383,162	2365,35	4899,426	973,6144	1968,357
P05543	THBG_HUMAN	Thyroxine-binding globulin (Serpin A7) (T4-binding globulin)	34250,72	38890,15	23358,67	51798,22	56653,79	27921,22	20472,94	26023,11	17550,48	49567,69	103343,2	56669,87	41723,04	24623,35	64908,38	99912,89
P04066	FUCO_HUMAN	Tissue alpha-L-fucosidase (EC 3.2.1.51) (Alpha-L-fucosidase I) (Alpha-L-fucosi	26835,44	29291,89	21737,97	17940,47	26309,75	19329,8	29108,69	19273,23	15145,87	14158	16051,64	19854,41	22265,04	4129,778	10096,53	10145,34
P61812	TGFB2_HUMAN	Transforming growth factor beta-2 (TGF-beta-2) (BSC-1 cell growth inhibitor)	6707,059	7213,324	9485,755	8710,389	4751,557	6150,818	10001,93	13840,65	6638,781	8475,608	604,1354	1896,682	2829,134	4071,281	11534,7	22312,65
Q15582	BGH3_HUMAN	Transforming growth factor-beta-induced protein ig-h3 (Beta ig-h3) (Kerato-(	46947,07	12783,41	40382,94	10362,66	9938,899	22898,99	22183,66	13625,2	17005,3	13545,86	40436,83	24854,31	18461,11	28510,22	16596,26	22005,17
P02766	TTHY_HUMAN	Transthyretin (ATTR) (Prealbumin) (TBPA)	1353840	3237679	4621677	1793890	1233235	984668,6	1747808	1419267	2208292	1357021	1980211	1331045	1170668	1099655	2060243	487770,6
P60174	TPIS_HUMAN	Triosephosphate isomerase (TIM) (EC 5.3.1.1) (Triose-phosphate isomerase)	5386,597	9131,75	28136,98	12790,04	7818,617	13356,22	22031,42	16917,82	4697,397	24891,59	7500,851	12420,57	6833,63	7590,401	37737,77	2397,431
P00761	TRYP_PIG	Trypsin (EC 3.4.21.4)	5906,439	1733,064	10139,54	7186,002	4929,613	2964,63	5563,484	1444,34	3035,742	5650,326	5794,506	5743,159	14062,76	3531,135	4342,082	3468,476
Q9GZX9	TWSG1_HUMAN	Twisted gastrulation protein homolog 1	9286,309	6627,851	9986,127	8591,973	8191,012	5861,946	6751,948	9529,822	6667,409	5173,169	4253,766	1782,398	2429,14	740,3216	7046,82	352,9142
Q6EMK4	VASN_HUMAN	Vasorin (Protein slit-like 2)	9362,119	29172,32	17246,11	29974,52	18643,94	17225,75	26965,14	22598,25	4415,834	25823,04	30882,02	15218,12	21374,5	20471,32	42314,65	15860,25
P13611	CSPG2_HUMAN	Versican core protein (Chondroitin sulfate proteoglycan core protein 2) (Cho	37754,79	150893	220843,8	218090,9	271912,9	47974,94	440139,8	323761	291982,9	195945,7	28873,94	65495,68	91903,52	61195,38	59030,45	33440,89
P08670	VIME_HUMAN	Vimentin	5786,099	6749,885	7950,042	2379,585	3130,989	1327,111	1320,976	1491,537	3309,052	2161,904	9333,2	1616,022	5010,093	1710,151	20157,55	1171,783
P02774	VTDB_HUMAN	Vitamin D-binding protein (DBP) (VDB) (Gc protein-derived macrophage activ	4052557	3169466	3054715	2556295	2872587	4631154	2378200	3229263	3902645	5084988	3830681	2929054	4154200	3064887	1794843	2189936
P07225	PROS_HUMAN	Vitamin K-dependent protein S	38796,78	55442,03	87023,52	58425,98	65900,31	48461,29	87834,14	6536,567	70022,14	46399,72	43603,06	60439,78	72577,28	60123,8	48601,05	25117,24
P04004	VTNC_HUMAN	Vitronectin (VN) (S-protein) (Serum-spreading factor) (V75) [Cleaved into: Vi	112539,5	136958	118910,6	90736,21	99910,94	195472	130815,4	107865,5	159569,6	112127,4	109310,4	162954,4	215191,3	227287,1	149533,7	222400,7
Q8WY21	SORC1_HUMAN	VPS10 domain-containing receptor SorCS1 (hSorCS)	2744,552	1403,073	1124,075	2849,116	2653,538	1430,532	1107,967	789,7694	3526,839	1723,865	1388,046	3480,181	835,5289	1874,291	672,8692	1992,713
Q8TAG5	VTM2A_HUMAN	V-set and transmembrane domain-containing protein 2A	17848,5	3270,442	6958,137	3721,369	3238,116	6452,249	5181,147	38235,86	871,202	4880,841	3273,755	9666,355	2292,904	2174,419	2423,474	31573,09
A6NLU5	VTM2B_HUMAN	V-set and transmembrane domain-containing protein 2B	2429,135	2369,067	4125,227	2998,83	1516,065	651,1947	1491,591	4783,354	838,5461	2526,026	2253,527	1700,662	1568,794	2961,718	509,8271	6221,366
Q15904	VAS1_HUMAN	V-type proton ATPase subunit S1 (V-ATPase subunit S1) (Protein XAP-3) (V-AT	39657,15	37573,84	62543,02	60510,4	38864,25	24431,37	32197,51	38193,6	31996,16	30359,52	52617,44	24674,83	20787,59	75972,24	21796,05	12669
Q5VU97	CAHD1_HUMAN	VWFA and cache domain-containing protein 1 (Cache domain-containing prc	3482,479	5060,86	6100,144	1886,031	2415,733	1497,248	6249,374	1367,189	2261,523	1689,76	2156,496	3142,894	2075,327	1052,963	1091,51	2155,175
Q8IZC7	ZN101_HUMAN	Zinc finger protein 101 (Zinc finger protein HZF12)	4423,119	5261,3	4532,661	1697,672	6707,64	5100,655	3346,618	8556,088	9614,569	997,7549	14121,45	11301,85	20264,55	9643,496	547,8469	2192,421
Q9H4I2	ZHX3_HUMAN	Zinc fingers and homeoboxes protein 3 (Triple homeobox protein 1) (Zinc fin	853,663	1251,708	2712,829	1863,954	8999,743	547,3149	11629,04	6384,18	7780,434	1383,361	381,5931	5303,061	3716,797	4808,612	10456,18	1183,487
P25311	ZA2G_HUMAN	Zinc-alpha-2-glycoprotein (Zn-alpha-2-GP) (Zn-alpha-2-glycoprotein)	24184,92	24478,67	15731,98	15602,4	22292,66	24222,16	64475,49	33245,77	35716,34	23975,19	38778,9	49275,61	41376,18	24382,79	152454,5	215591
