# Supplementary material for: Systems pathology analysis identifies neurodegenerative nature of age‐related vitreoretinal interface diseases
Source: Aging Cell. 2018 Jul 2;17(5):e12809. doi: 10.1111/acel.12809 (PMC6156470; doi:10.1111/acel.12809)
Supplement: Supplementary file 12 [file ACEL-17-e12809-s012.pdf]

Supplemental Table S9: Significantly differed proteins between iERM and MH proteomes, q-value < 0.05.
53 proteins were present at higher level (red) and 65 proteins at lower level (yellow) in the iERM proteome when compared to MH proteome, the abundance ratio >2.

		Average of MS1 Intensities			Fold differences
Accession	Description	iERM	MH	DME	iERM vs MH
Q99708	DNA endonuclease RBBP8 OS=Homo sapiens GN=RBBP8 PE=1 SV=2 - [COM1_HUMAN]	1407277	642	6708	2191,50
P14679	Tyrosinase OS=Homo sapiens GN=TYR PE=1 SV=3 - [TYRO_HUMAN]	201837	2796	4926	72,19
P18583	Protein SON OS=Homo sapiens GN=SON PE=1 SV=4 - [SON_HUMAN]	31518	1625	1935	19,40
Q9NNX1	Tuftelin OS=Homo sapiens GN=TUFT1 PE=1 SV=1 - [TUFT1_HUMAN]	486602980	33160534	4265973	14,67
Q9UFH2	Dynein heavy chain 17, axonemal OS=Homo sapiens GN=DNAH17 PE=1 SV=2 - [DYH17_HUMAN]	238653	19061	43965	12,52
Q96L92	Sorting nexin-27 OS=Homo sapiens GN=SNX27 PE=1 SV=2 - [SNX27_HUMAN]	1275112	103225	1122922	12,35
Q96MI9	Cytosolic carboxypeptidase 4 OS=Homo sapiens GN=AGBL1 PE=1 SV=2 - [CBPC4_HUMAN]	1265305	115153	385610	10,99
P51530	DNA replication ATP-dependent helicase/nuclease DNA2 OS=Homo sapiens GN=DNA2 PE=1 SV=3 - [DNA2	47842	5354	8671	8,94
Q9H707	Zinc finger protein 552 OS=Homo sapiens GN=ZNF552 PE=1 SV=2 - [ZN552_HUMAN]	455369141	68545638	29804070	6,64
A0AVI2	Fer-1-like protein 5 OS=Homo sapiens GN=FER1L5 PE=2 SV=2 - [FR1L5_HUMAN]	24866	3771	0	6,59
Q8N1I0	Dedicator of cytokinesis protein 4 OS=Homo sapiens GN=DOCK4 PE=1 SV=3 - [DOCK4_HUMAN]	4653239	737564	486392	6,31
P49448	Glutamate dehydrogenase 2, mitochondrial OS=Homo sapiens GN=GLUD2 PE=1 SV=2 - [DHE4_HUMAN]	178367	28595	188552	6,24
Q07002	Cyclin-dependent kinase 18 OS=Homo sapiens GN=CDK18 PE=1 SV=3 - [CDK18_HUMAN]	135425	23654	3205	5,73
Q9P2M1	LRP2-binding protein OS=Homo sapiens GN=LRP2BP PE=1 SV=2 - [LR2BP_HUMAN]	923272	161478	102809	5,72
Q53RD9	Fibulin-7 OS=Homo sapiens GN=FBLN7 PE=2 SV=1 - [FBLN7_HUMAN]	26774512	5019928	9828165	5,33
Q8WVM8	Sec1 family domain-containing protein 1 OS=Homo sapiens GN=SCFD1 PE=1 SV=4 - [SCFD1_HUMAN]	3281523	621465	53311	5,28
A0FGR9	Extended synaptotagmin-3 OS=Homo sapiens GN=ESYT3 PE=1 SV=1 - [ESYT3_HUMAN]	656600	125457	94220	5,23
Q9H069	Dynein regulatory complex subunit 3 OS=Homo sapiens GN=DRC3 PE=2 SV=2 - [DRC3_HUMAN]	320216	61954	84342	5,17
Q99996	A-kinase anchor protein 9 OS=Homo sapiens GN=AKAP9 PE=1 SV=3 - [AKAP9_HUMAN]	10826785	2123410	1258187	5,10
Q9BXG8	Spermatogenic leucine zipper protein 1 OS=Homo sapiens GN=SPZ1 PE=1 SV=2 - [SPZ1_HUMAN]	7827952	1704541	979850	4,59
Q9NQX4	Unconventional myosin-Vc OS=Homo sapiens GN=MYO5C PE=1 SV=2 - [MYO5C_HUMAN]	181693	39805	183835	4,56
Q8IWP9	Coiled-coil domain-containing protein 28A OS=Homo sapiens GN=CCDC28A PE=1 SV=1 - [CC28A_HUMAN	10334179	2347563	19222375	4,40
P51878	Caspase-5 OS=Homo sapiens GN=CASP5 PE=1 SV=3 - [CASP5_HUMAN]	941481	220558	149682	4,27
Q02817	Mucin-2 OS=Homo sapiens GN=MUC2 PE=1 SV=2 - [MUC2_HUMAN]	53439520	12623677	596829	4,23
P54756	Ephrin type-A receptor 5 OS=Homo sapiens GN=EPHA5 PE=1 SV=3 - [EPHA5_HUMAN]	91257544	21631146	23743593	4,22
Q96HY6	DDRGK domain-containing protein 1 OS=Homo sapiens GN=DDRGK1 PE=1 SV=2 - [DDRGK_HUMAN]	52799	13005	2808	4,06
Q7LGC8	Carbohydrate sulfotransferase 3 OS=Homo sapiens GN=CHST3 PE=1 SV=3 - [CHST3_HUMAN]	93233	24369	1080296	3,83
Q86X52	Chondroitin sulfate synthase 1 OS=Homo sapiens GN=CHSY1 PE=1 SV=3 - [CHSS1_HUMAN]	6254952	1714291	2876489	3,65
Q9UHB6	LIM domain and actin-binding protein 1 OS=Homo sapiens GN=LIMA1 PE=1 SV=1 - [LIMA1_HUMAN]	2268924	673378	228998	3,37
P53004	Biliverdin reductase A OS=Homo sapiens GN=BLVRA PE=1 SV=2 - [BIEA_HUMAN]	3560070	1085335	337316	3,28
Q8N137	Centrobin OS=Homo sapiens GN=CNTROB PE=1 SV=1 - [CNTRB_HUMAN]	8405583	2568220	1576459	3,27
P30086	Phosphatidylethanolamine-binding protein 1 OS=Homo sapiens GN=PEBP1 PE=1 SV=3 - [PEBP1_HUMAN]	75161	23600	93761	3,18
Q6A162	Keratin, type I cytoskeletal 40 OS=Homo sapiens GN=KRT40 PE=1 SV=2 - [K1C40_HUMAN]	18684961	6171587	3934075	3,03
Q03468	DNA excision repair protein ERCC-6 OS=Homo sapiens GN=ERCC6 PE=1 SV=1 - [ERCC6_HUMAN]	150955	50599	112290	2,98
Q9NYW0	Taste receptor type 2 member 10 OS=Homo sapiens GN=TAS2R10 PE=1 SV=3 - [T2R10_HUMAN]	2163851	729167	2184188	2,97
O75063	Glycosaminoglycan xylosylkinase OS=Homo sapiens GN=FAM20B PE=1 SV=1 - [XYLK_HUMAN]	854683	290223	48227	2,94
Q9UHB4	NADPH-dependent diflavin oxidoreductase 1 OS=Homo sapiens GN=NDOR1 PE=1 SV=1 - [NDOR1_HUMAI	33402723	11484948	13183708	2,91
P20929	Nebulin OS=Homo sapiens GN=NEB PE=1 SV=5 - [NEBU_HUMAN]	35937405	12357649	2104757	2,91
P12271	Retinaldehyde-binding protein 1 OS=Homo sapiens GN=RLBP1 PE=1 SV=2 - [RLBP1_HUMAN]	593376	209805	352979	2,83
Q8IUG5	Unconventional myosin-XVIIIb OS=Homo sapiens GN=MYO18B PE=1 SV=1 - [MY18B_HUMAN]	64077	22841	118636	2,81
P26006	Integrin alpha-3 OS=Homo sapiens GN=ITGA3 PE=1 SV=5 - [ITA3_HUMAN]	617077	220599	18529	2,80
Q96ES7	SAGA-associated factor 29 OS=Homo sapiens GN=SGF29 PE=1 SV=1 - [SGF29_HUMAN]	7712188	2793330	1344593	2,76
P62829	60S ribosomal protein L23 OS=Homo sapiens GN=RPL23 PE=1 SV=1 - [RL23_HUMAN]	928532	340907	167372	2,72
Q86WI1	Fibrocystin-L OS=Homo sapiens GN=PKHD1L1 PE=2 SV=2 - [PKHL1_HUMAN]	4713766	1734585	1630598	2,72
Q9NQ38	Serine protease inhibitor Kazal-type 5 OS=Homo sapiens GN=SPINK5 PE=1 SV=2 - [ISK5_HUMAN]	81468761	32322798	58596913	2,52
Q9NZP8	Complement C1r subcomponent-like protein OS=Homo sapiens GN=C1RL PE=1 SV=2 - [C1RL_HUMAN]	65413	26464	2635	2,47
O15031	Plexin-B2 OS=Homo sapiens GN=PLXNB2 PE=1 SV=3 - [PLXB2_HUMAN]	401539	162740	146028	2,47
Q15746	Myosin light chain kinase, smooth muscle OS=Homo sapiens GN=MYLK PE=1 SV=4 - [MYLK_HUMAN]	447407	191986	1088989	2,33
Q14746	Conserved oligomeric Golgi complex subunit 2 OS=Homo sapiens GN=COG2 PE=1 SV=1 - [COG2_HUMAN]	4063257	1754031	860562	2,32
Q8N945	PRELI domain-containing protein 2 OS=Homo sapiens GN=PRELID2 PE=2 SV=1 - [PRLD2_HUMAN]	92221901	41309964	163344177	2,23
P19021	Peptidyl-glycine alpha-amidating monooxygenase OS=Homo sapiens GN=PAM PE=1 SV=2 - [AMD_HUMAI	204498	93636	65779	2,18
P62736	Actin, aortic smooth muscle OS=Homo sapiens GN=ACTA2 PE=1 SV=1 - [ACTA_HUMAN]	691018	337722	1414754	2,05
P0C7V8	DDB1- and CUL4-associated factor 8-like protein 2 OS=Homo sapiens GN=DCAF8L2 PE=2 SV=2 - [DC8L2_H	2561309	1279511	6764568	2,00
Q9Y234	Lipoyltransferase 1, mitochondrial OS=Homo sapiens GN=LIPT1 PE=1 SV=1 - [LIPT_HUMAN]	5506264	2907853	3039260	1,89
P09486	SPARC OS=Homo sapiens GN=SPARC PE=1 SV=1 - [SPRC_HUMAN]	320296	172323	755598	1,86
Q5VST9	Obscurin OS=Homo sapiens GN=OBSCN PE=1 SV=3 - [OBSCN_HUMAN]	1545015	889729	819546	1,74
P02679	Fibrinogen gamma chain OS=Homo sapiens GN=FGG PE=1 SV=3 - [FIBG_HUMAN]	19790041	11616118	42118814	1,70
P02787	Serotransferrin OS=Homo sapiens GN=TF PE=1 SV=3 - [TRFE_HUMAN]	2991882936	1765912759	2097086346	1,69
P02042	Hemoglobin subunit delta OS=Homo sapiens GN=HBD PE=1 SV=2 - [HBD_HUMAN]	2338527	1399607	1754498	1,67
P07225	Vitamin K-dependent protein S OS=Homo sapiens GN=PROS1 PE=1 SV=1 - [PROS_HUMAN]	3093378	1854466	1591403	1,67
Q9Y6G9	Cytoplasmic dynein 1 light intermediate chain 1 OS=Homo sapiens GN=DYNC1LI1 PE=1 SV=3 - [DC1L1_HU	6155047	3807938	5267349	1,62
P40189	Interleukin-6 receptor subunit beta OS=Homo sapiens GN=IL6ST PE=1 SV=2 - [IL6RB_HUMAN]	487004	302921	424074	1,61
P51178	1-phosphatidylinositol 4,5-bisphosphate phosphodiesterase delta-1 OS=Homo sapiens GN=PLCD1 PE=1 S	866707	539368	729664	1,61
Q02386	Zinc finger protein 45 OS=Homo sapiens GN=ZNF45 PE=2 SV=2 - [ZNF45_HUMAN]	1041158	663040	1836787	1,57
P01033	Metalloproteinase inhibitor 1 OS=Homo sapiens GN=TIMP1 PE=1 SV=1 - [TIMP1_HUMAN]	984406	642435	1868741	1,53
P00736	Complement C1r subcomponent OS=Homo sapiens GN=C1R PE=1 SV=2 - [C1R_HUMAN]	2502929	1674257	3164003	1,49
Q9Y5X3	Sorting nexin-5 OS=Homo sapiens GN=SNX5 PE=1 SV=1 - [SNX5_HUMAN]	102952	70525	62417	1,46
P06396	Gelsolin OS=Homo sapiens GN=GSN PE=1 SV=1 - [GELS_HUMAN]	95647566	75151612	86612186	1,27
Q9P212	1-phosphatidylinositol 4,5-bisphosphate phosphodiesterase epsilon-1 OS=Homo sapiens GN=PLCE1 PE=1	5575551	7291016	3065990	0,76
Q9P2P6	StAR-related lipid transfer protein 9 OS=Homo sapiens GN=STARD9 PE=1 SV=3 - [STAR9_HUMAN]	2225654	3047024	1151132	0,73
Q24JP5	Transmembrane protein 132A OS=Homo sapiens GN=TMEM132A PE=1 SV=1 - [T132A_HUMAN]	5664553	7854394	7670946	0,72
P28290	Sperm-specific antigen 2 OS=Homo sapiens GN=SSFA2 PE=1 SV=3 - [SSFA2_HUMAN]	2271704	3162191	918250	0,72
Q92878	DNA repair protein RAD50 OS=Homo sapiens GN=RAD50 PE=1 SV=1 - [RAD50_HUMAN]	945816	1385599	1285826	0,68
Q14520	Hyaluronan-binding protein 2 OS=Homo sapiens GN=HABP2 PE=1 SV=1 - [HABP2_HUMAN]	104468	159481	101075	0,66
Q7Z2W4	Zinc finger CCCH-type antiviral protein 1 OS=Homo sapiens GN=ZC3HAV1 PE=1 SV=3 - [ZCCHV_HUMAN]	893640	1385891	405932	0,64
P24043	Laminin subunit alpha-2 OS=Homo sapiens GN=LAMA2 PE=1 SV=4 - [LAMA2_HUMAN]	7019363	11187509	8954953	0,63
Q9H3G5	Probable serine carboxypeptidase CPVL OS=Homo sapiens GN=CPVL PE=1 SV=2 - [CPVL_HUMAN]	1781747	2912808	582089	0,61
O00443	Phosphatidylinositol 4-phosphate 3-kinase C2 domain-containing subunit alpha OS=Homo sapiens GN=PI	5851954	9626104	3644453	0,61
O60216	Double-strand-break repair protein rad21 homolog OS=Homo sapiens GN=RAD21 PE=1 SV=2 - [RAD21_H	34546990	57749017	46782395	0,60
Q2PPJ7	Ral GTPase-activating protein subunit alpha-2 OS=Homo sapiens GN=RALGAPA2 PE=1 SV=2 - [RGPA2_HU	1107763	1852063	3006316	0,60
O96028	Histone-lysine N-methyltransferase NSD2 OS=Homo sapiens GN=WHSC1 PE=1 SV=1 - [NSD2_HUMAN]	590717	991269	302018	0,60
Q12805	EGF-containing fibulin-like extracellular matrix protein 1 OS=Homo sapiens GN=EFEMP1 PE=1 SV=2 - [FBL	36172522	64307594	41767332	0,56

P01766	Ig heavy chain V-III region BRO OS=Homo sapiens PE=1 SV=1 - [HV305_HUMAN]	2532771	4532643	3644537	0,56
P02751	Fibronectin OS=Homo sapiens GN=FN1 PE=1 SV=4 - [FINC_HUMAN]	36573293	65555579	119838606	0,56
P01859	Ig gamma-2 chain C region OS=Homo sapiens GN=IGHG2 PE=1 SV=2 - [IGHG2_HUMAN]	61518052	110977664	93466100	0,55
P25092	Heat-stable enterotoxin receptor OS=Homo sapiens GN=GUCY2C PE=1 SV=2 - [GUC2C_HUMAN]	16467404	29803585	47667557	0,55
Q9P2M7	Cingulin OS=Homo sapiens GN=CGN PE=1 SV=2 - [CING_HUMAN]	1147424	2099882	529615	0,55
Q9Y4F4	Protein FAM179B OS=Homo sapiens GN=FAM179B PE=1 SV=4 - [F179B_HUMAN]	1352790	2561655	577221	0,53
P35222	Catenin beta-1 OS=Homo sapiens GN=CTNNB1 PE=1 SV=1 - [CTNB1_HUMAN]	2039975	3899096	129234	0,52
Q6IQ26	DENN domain-containing protein 5A OS=Homo sapiens GN=DENND5A PE=1 SV=2 - [DEN5A_HUMAN]	2237114	4429906	2230130	0,51
Q12841	Follistatin-related protein 1 OS=Homo sapiens GN=FSTL1 PE=1 SV=1 - [FSTL1_HUMAN]	26043744	51648101	100742484	0,50
Q96F63	Coiled-coil domain-containing protein 97 OS=Homo sapiens GN=CCDC97 PE=1 SV=1 - [CCD97_HUMAN]	3360512	6735018	2803151	0,50
Q9HC35	Echinoderm microtubule-associated protein-like 4 OS=Homo sapiens GN=EML4 PE=1 SV=3 - [EMAL4_HUI	8637339	17332211	10119597	0,50
Q6UWY2	Serine protease 57 OS=Homo sapiens GN=PRSS57 PE=1 SV=2 - [PRS57_HUMAN]	123189	248593	3722	0,50
O43913	Origin recognition complex subunit 5 OS=Homo sapiens GN=ORC5 PE=1 SV=1 - [ORC5_HUMAN]	247199	499071	484558	0,50
Q00056	Homeobox protein Hox-A4 OS=Homo sapiens GN=HOXA4 PE=2 SV=3 - [HXA4_HUMAN]	93728	189509	2707	0,49
Q92621	Nuclear pore complex protein Nup205 OS=Homo sapiens GN=NUP205 PE=1 SV=3 - [NU205_HUMAN]	1388028	2860975	1465437	0,49
O15354	Prosaposin receptor GPR37 OS=Homo sapiens GN=GPR37 PE=1 SV=2 - [GPR37_HUMAN]	46875404	100084565	119182703	0,47
P26358	DNA (cytosine-5)-methyltransferase 1 OS=Homo sapiens GN=DNMT1 PE=1 SV=2 - [DNMT1_HUMAN]	3952428	8587929	6529996	0,46
Q9P266	Junctional protein associated with coronary artery disease OS=Homo sapiens GN=KIAA1462 PE=1 SV=3 -	499281	1084900	652741	0,46
P0C7P3	Schlafen family member 14 OS=Homo sapiens GN=SLFN14 PE=2 SV=2 - [SLN14_HUMAN]	4731746	10401692	8450106	0,45
Q96MR6	Cilia- and flagella-associated protein 57 OS=Homo sapiens GN=CFAP57 PE=2 SV=3 - [CFA57_HUMAN]	2179435	4867951	633691	0,45
Q8NI51	Transcriptional repressor CTCFL OS=Homo sapiens GN=CTCFL PE=1 SV=2 - [CTCFL_HUMAN]	2007176	4524294	5976859	0,44
P07602	Prosaposin OS=Homo sapiens GN=PSAP PE=1 SV=2 - [SAP_HUMAN]	2066934	4667868	3826122	0,44
Q8NE71	ATP-binding cassette sub-family F member 1 OS=Homo sapiens GN=ABCF1 PE=1 SV=2 - [ABCF1_HUMAN]	5873295	13299858	842580	0,44
Q9BYW2	Histone-lysine N-methyltransferase SETD2 OS=Homo sapiens GN=SETD2 PE=1 SV=3 - [SETD2_HUMAN]	2729824	6245095	20354142	0,44
Q99618	Cell division cycle-associated protein 3 OS=Homo sapiens GN=CDCA3 PE=1 SV=1 - [CDCA3_HUMAN]	85833	202271	42440	0,42
Q9UF33	Ephrin type-A receptor 6 OS=Homo sapiens GN=EPHA6 PE=2 SV=3 - [EPHA6_HUMAN]	3673786	9009759	14200826	0,41
P01714	Ig lambda chain V-III region SH OS=Homo sapiens PE=1 SV=1 - [LV301_HUMAN]	11272	27665	15578	0,41
Q96NL6	Sodium channel and clathrin linker 1 OS=Homo sapiens GN=SCLT1 PE=1 SV=2 - [SCLT1_HUMAN]	3721772	9142708	3299192	0,41
Q6PGQ7	Protein aurora borealis OS=Homo sapiens GN=BORA PE=1 SV=2 - [BORA_HUMAN]	146280	362305	36415	0,40
O60733	85/88 kDa calcium-independent phospholipase A2 OS=Homo sapiens GN=PLA2G6 PE=1 SV=2 - [PLPL9_HU	44512	111940	194889	0,40
Q5VZK9	Leucine-rich repeat-containing protein 16A OS=Homo sapiens GN=LRRC16A PE=1 SV=1 - [LR16A_HUMAN	354636	894206	340215	0,40
O43174	Cytochrome P450 26A1 OS=Homo sapiens GN=CYP26A1 PE=2 SV=2 - [CP26A_HUMAN]	108011	272473	707278	0,40
P48051	G protein-activated inward rectifier potassium channel 2 OS=Homo sapiens GN=KCNJ6 PE=1 SV=1 - [KCNJ	539608	1381760	741909	0,39
Q9UBN7	Histone deacetylase 6 OS=Homo sapiens GN=HDAC6 PE=1 SV=2 - [HDAC6_HUMAN]	1339885	3450512	32645	0,39
Q15413	Ryanodine receptor 3 OS=Homo sapiens GN=RYR3 PE=1 SV=3 - [RYR3_HUMAN]	9468459	24488080	18710972	0,39
P09488	Glutathione S-transferase Mu 1 OS=Homo sapiens GN=GSTM1 PE=1 SV=3 - [GSTM1_HUMAN]	508262	1334508	1834704	0,38
Q9UBX1	Cathepsin F OS=Homo sapiens GN=CTSF PE=1 SV=1 - [CATF_HUMAN]	11900122	31276874	4578743	0,38
P15498	Proto-oncogene vav OS=Homo sapiens GN=VAV1 PE=1 SV=4 - [VAV_HUMAN]	29128253	77090456	54628943	0,38
Q9Y5E7	Protocadherin beta-2 OS=Homo sapiens GN=PCDHB2 PE=1 SV=1 - [PCDB2_HUMAN]	2744221	7368655	1249171	0,37
Q8TCN5	Zinc finger protein 507 OS=Homo sapiens GN=ZNF507 PE=1 SV=2 - [ZN507_HUMAN]	6961748	18715960	13783857	0,37
P49368	T-complex protein 1 subunit gamma OS=Homo sapiens GN=CCT3 PE=1 SV=4 - [TCPG_HUMAN]	945646	2565663	740812	0,37
P48681	Nestin OS=Homo sapiens GN=NES PE=1 SV=2 - [NEST_HUMAN]	4984952	13553178	15878663	0,37
Q5T6C5	Ataxin-7-like protein 2 OS=Homo sapiens GN=ATXN7L2 PE=3 SV=1 - [AT7L2_HUMAN]	14963295	41385178	12919801	0,36
P49750	YLP motif-containing protein 1 OS=Homo sapiens GN=YLPM1 PE=1 SV=3 - [YLPM1_HUMAN]	60900	168630	11425	0,36
Q96LZ7	Regulator of microtubule dynamics protein 2 OS=Homo sapiens GN=RMDN2 PE=1 SV=2 - [RMD2_HUMAN	292309	810130	507564	0,36
Q86WZ6	Zinc finger protein 227 OS=Homo sapiens GN=ZNF227 PE=1 SV=1 - [ZN227_HUMAN]	2113675	5902922	1977203	0,36
Q13948	Protein CASP OS=Homo sapiens GN=CUX1 PE=1 SV=2 - [CASP_HUMAN]	559075	1573362	377103	0,36
Q8WXQ3	Putative uncharacterized protein encoded by LINC01599 OS=Homo sapiens GN=LINC01599 PE=2 SV=1 - [	325811	922170	84805	0,35
Q96PX9	Pleckstrin homology domain-containing family G member 4B OS=Homo sapiens GN=PLEKHG4B PE=2 SV=	1467218	4210157	2989672	0,35
Q8TCU4	Alstrom syndrome protein 1 OS=Homo sapiens GN=ALMS1 PE=1 SV=3 - [ALMS1_HUMAN]	12140544	35070875	45510096	0,35
Q6ZP82	Coiled-coil domain-containing protein 141 OS=Homo sapiens GN=CCDC141 PE=1 SV=2 - [CC141_HUMAN]	84345	245190	50502	0,34
P02549	Spectrin alpha chain, erythrocytic 1 OS=Homo sapiens GN=SPTA1 PE=1 SV=5 - [SPTA1_HUMAN]	14965135	44265807	3317173	0,34
Q96ME7	Zinc finger protein 512 OS=Homo sapiens GN=ZNF512 PE=1 SV=2 - [ZN512_HUMAN]	2281909	6822903	853431	0,33
Q969M1	Mitochondrial import receptor subunit TOM40B OS=Homo sapiens GN=TOMM40L PE=1 SV=1 - [TM40L_H	311250	953897	595309	0,33
Q2NKX8	DNA excision repair protein ERCC-6-like OS=Homo sapiens GN=ERCC6L PE=1 SV=1 - [ERC6L_HUMAN]	56117	174657	31338	0,32
Q8TDB6	E3 ubiquitin-protein ligase DTX3L OS=Homo sapiens GN=DTX3L PE=1 SV=1 - [DTX3L_HUMAN]	17005	53022	18135	0,32
Q9UPN3	Microtubule-actin cross-linking factor 1, isoforms 1/2/3/5 OS=Homo sapiens GN=MACF1 PE=1 SV=4 - [MA	29129	90832	34273	0,32
Q92628	Uncharacterized protein KIAA0232 OS=Homo sapiens GN=KIAA0232 PE=1 SV=5 - [K0232_HUMAN]	29562	95197	9176	0,31
Q96S79	Ras-like protein family member 10B OS=Homo sapiens GN=RASL10B PE=2 SV=1 - [RSLAB_HUMAN]	303757	984700	448384	0,31
Q96RL7	Vacuolar protein sorting-associated protein 13A OS=Homo sapiens GN=VPS13A PE=1 SV=2 - [VP13A_HUM	218190	709629	685528	0,31
Q15173	Serine/threonine-protein phosphatase 2A 56 kDa regulatory subunit beta isoform OS=Homo sapiens GN=	14944	48695	0	0,31
P21817	Ryanodine receptor 1 OS=Homo sapiens GN=RYR1 PE=1 SV=3 - [RYR1_HUMAN]	64834	212527	87524	0,31
Q86UN2	Reticulon-4 receptor-like 1 OS=Homo sapiens GN=RTN4RL1 PE=1 SV=1 - [R4RL1_HUMAN]	36017563	119876831	38581223	0,30
Q6ZT07	TBC1 domain family member 9 OS=Homo sapiens GN=TBC1D9 PE=2 SV=2 - [TBCD9_HUMAN]	253799	880723	218081	0,29
Q9Y4I1	Unconventional myosin-Va OS=Homo sapiens GN=MYO5A PE=1 SV=2 - [MYO5A_HUMAN]	314925	1093701	382147	0,29
Q53QZ3	Rho GTPase-activating protein 15 OS=Homo sapiens GN=ARHGAP15 PE=1 SV=2 - [RHG15_HUMAN]	52238	184767	0	0,28
Q9UBM8	Alpha-1,3-mannosyl-glycoprotein 4-beta-N-acetylglucosaminyltransferase C OS=Homo sapiens GN=MGAT	21850	77908	29157	0,28
Q8NF50	Dedicator of cytokinesis protein 8 OS=Homo sapiens GN=DOCK8 PE=1 SV=3 - [DOCK8_HUMAN]	9504	34517	522527	0,28
P42702	Leukemia inhibitory factor receptor OS=Homo sapiens GN=LIFR PE=1 SV=1 - [LIFR_HUMAN]	70400	258480	72166	0,27
Q9P2E2	Kinesin-like protein KIF17 OS=Homo sapiens GN=KIF17 PE=2 SV=3 - [KIF17_HUMAN]	47777	179214	358072	0,27
Q9H8L6	Multimerin-2 OS=Homo sapiens GN=MMRN2 PE=1 SV=2 - [MMRN2_HUMAN]	363319	1384464	436302	0,26
P42356	Phosphatidylinositol 4-kinase alpha OS=Homo sapiens GN=PI4KA PE=1 SV=4 - [PI4KA_HUMAN]	672176	2723851	150921	0,25
O43306	Adenylate cyclase type 6 OS=Homo sapiens GN=ADCY6 PE=1 SV=2 - [ADCY6_HUMAN]	213099	1136476	61303	0,19
Q2KHR3	Glutamine and serine-rich protein 1 OS=Homo sapiens GN=QSER1 PE=1 SV=3 - [QSER1_HUMAN]	3964	25087	30691	0,16
Q96N16	Janus kinase and microtubule-interacting protein 1 OS=Homo sapiens GN=JAKMIP1 PE=1 SV=1 - [JKIP1_H	267418	1793281	831787	0,15
P0CG05	Ig lambda-2 chain C regions OS=Homo sapiens GN=IGLC2 PE=1 SV=1 - [LAC2_HUMAN]	30530	215635	455035	0,14
Q8NG11	Tetraspanin-14 OS=Homo sapiens GN=TSPAN14 PE=1 SV=1 - [TSN14_HUMAN]	1268893	9584468	1757186	0,13
Q6P3R8	Serine/threonine-protein kinase Nek5 OS=Homo sapiens GN=NEK5 PE=2 SV=1 - [NEK5_HUMAN]	2528	26185	28860	0,10
